# Repeated observation of immune gene sets enrichment in women with non-small cell lung cancer

**DOI:** 10.18632/oncotarget.7943

**Published:** 2016-03-06

**Authors:** Jhajaira M. Araujo, Alexandra Prado, Nadezhda K. Cardenas, Mayer Zaharia, Richard Dyer, Franco Doimi, Leny Bravo, Luis Pinillos, Zaida Morante, Alfredo Aguilar, Luis A. Mas, Henry L. Gomez, Carlos S. Vallejos, Christian Rolfo, Joseph A. Pinto

**Affiliations:** ^1^ Unidad de Investigación Básica y Traslacional, Oncosalud-AUNA, San Borja, Lima 41, Peru; ^2^ Escuela de Medicina Humana, Universidad Privada San Juan Bautista, Chorrillos, Lima 09, Peru; ^3^ Departamento de Patología, Oncosalud-AUNA, San Borja, Lima 41, Peru; ^4^ Departamento de Medicina Oncológica, Instituto Nacional de Enfermedades Neoplásicas, Surquillo, Lima 41, Peru; ^5^ Phase I – Early Clinical Trials Unit, Antwerp University Hospital, Antwerp, Edegem 2650, Belgium

**Keywords:** non-small cell lung cancer, gender, GSEA, CIBERSORT, immune gene sets

## Abstract

There are different biological and clinical patterns of lung cancer between genders indicating intrinsic differences leading to increased sensitivity to cigarette smoke-induced DNA damage, mutational patterns of KRAS and better clinical outcomes in women while differences between genders at gene-expression levels was not previously reported. Here we show an enrichment of immune genes in NSCLC in women compared to men. We found in a GSEA analysis (by biological processes annotated from Gene Ontology) of six public datasets a repeated observation of immune gene sets enrichment in women. “Immune system process”, “immune response”, “defense response”, “cellular defense response” and “regulation of immune system process” were the gene sets most over-represented while *APOBEC3G, APOBEC3F, LAT, CD1D* and *CCL5* represented the top-five core genes. Characterization of immune cell composition with the platform CIBERSORT showed no differences between genders; however, there were differences when tumor tissues were compared to normal tissues. Our results suggest different immune responses in NSCLC between genders that could be related with the different clinical outcome.

## INTRODUCTION

Lung cancer is the most common malignancy and the leading cause of death worldwide where tobacco smoking is the main risk factor for the development of this cancer [1, 2].

It is well known that lung cancer shows different patterns of clinical characteristics and outcomes according to sex. Women have higher susceptibility to cigarette smoke-induced DNA damage and an increased risk for lung cancer, including higher levels of DNA adducts and higher frequency of the KRAS G12C mutation than men [3, 4]. In addition, female patients tend to present lung cancer at a younger age and in more advanced stages; however, women have better prognostic than men [5, 6].

Due to different susceptibility to DNA damage, according to sex, our research group previously evaluated differences between genders in gene expression and mutational status in genes involved in DNA repair without observing differences. In contrast, in a global evaluation, some gene sets were differentially enriched in women, including immune gene sets [7]. Despite the great advances in the knowledge of the genomic landscape of lung cancer, transcriptional differences between genders have not been previously explored.

With the aim of exploring transcriptional differences of Non-small cell lung cancer (NSCLC) between the genders, we performed a comprehensive analysis of differentially enriched immune gene sets annotated by Gene Ontology Biological Process (C5 BP).

## RESULTS

### Gene set enrichment analysis (GSEA) reveals enrichment of immune gene sets in NSCLC in women

**Figure 1:**
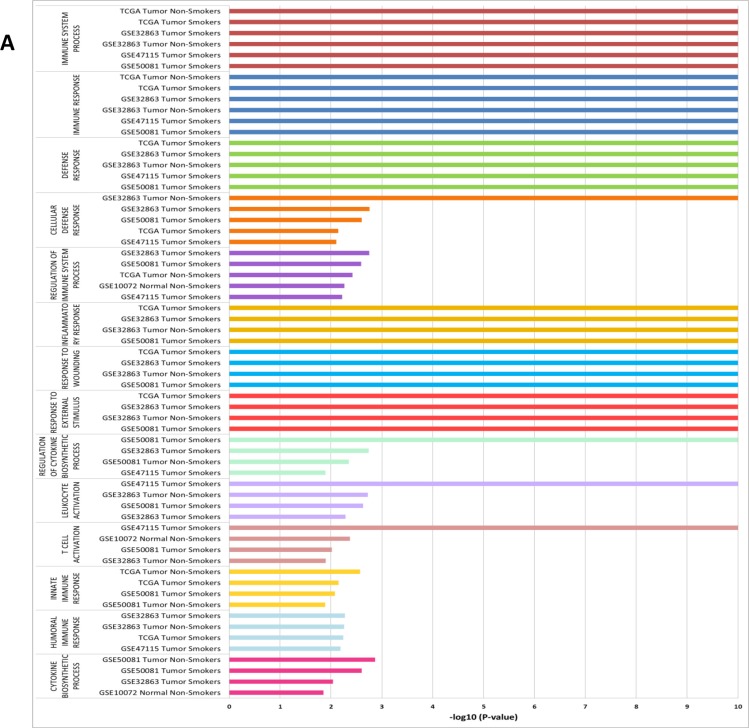

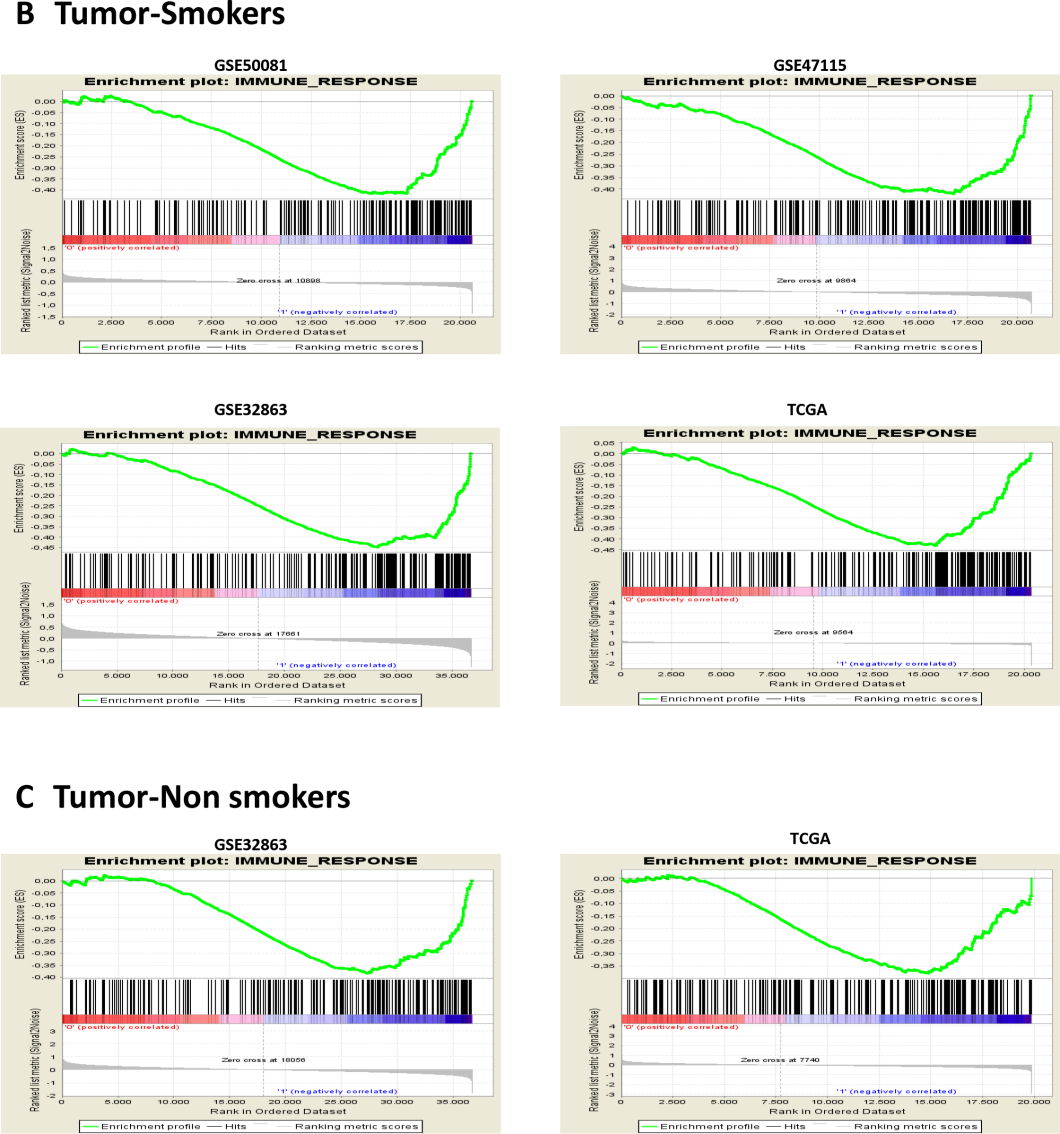
Immune gene sets enrichment more overrepresented in women –log10 of *P*-values are shown in different subsets (**A**). Enrichment profile generated with GSEA in the gene set “immune process” comparing NSCLC in men vs NSCLC in women shown enrichment in female in smokers (**B**) and in non-smokers (**C**).

**Figure 2:**
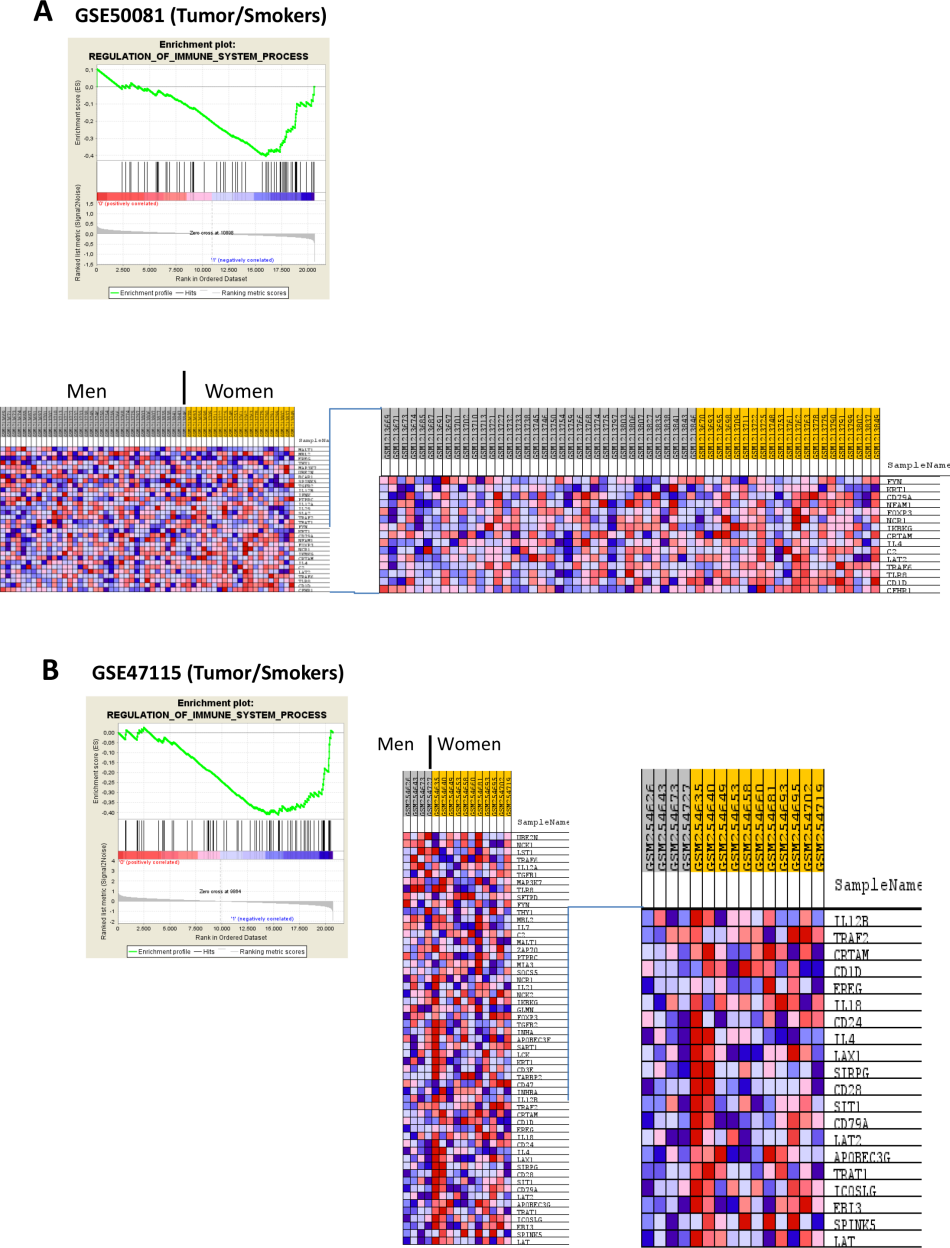

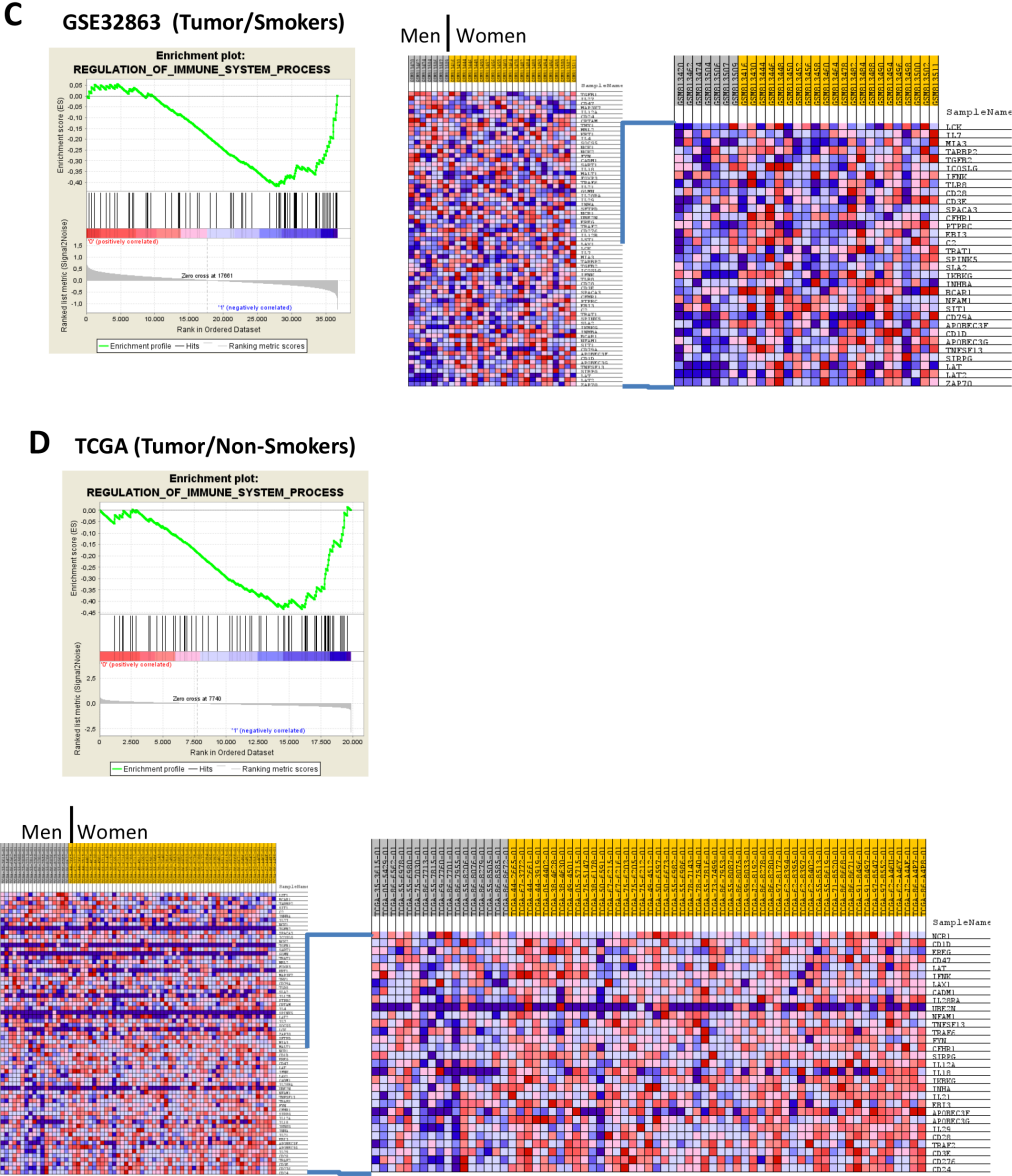

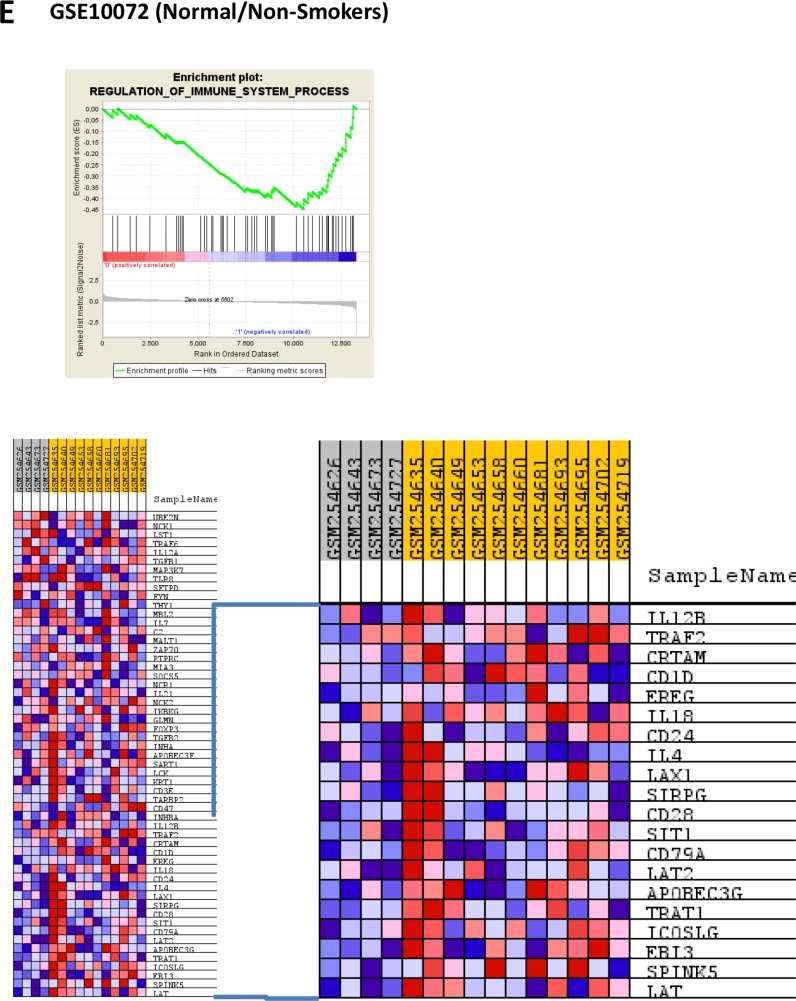
Enrichment profile and heatmaps generated with comparing the biological process: “REGULATION_OF IMMUNE_SYSTEM_PROCESS” between NSCLC in men vs NSCLC in women This entichment was observed in subsets of smokers (Figures **A**, **B** and **C**), non-smokers (**D**) and healthy tissue from non-smokers (**E**).

The structure of the data for GSEA analysis is shown in Table 1. Immune gene sets enrichment was not observed in men. In contrast, we found 40 immune gene sets enriched in women where the most over-represented where “Immune Response” ([GO:0006955]; *p*-values < 0.00000001; FDR's 0.4%–24.2%) and “Immune System Process” ([GO:0002376]; *p*-values < 0.00000001; FDR's: 0.4%–24.3%) in 06 subsets; “Defense Response” ([GO:0006952; *p*-values < 0.00000001; FDR's: 0.3%–13.6%), “Cellular Defense Response” ([GO:0006968]; *p*-values between < 0.00000001 to 0.00784929) and “Regulation of Immune System Process” ([GO:0002682]; *p*-values between 0.00176991 to 0.0060423) in 05 subsets (Figure 1 and Figure 2). List of all datasets with immune gene sets enrichment in ≥ 2 subsets are shown in Tables S1 and S2. We were not able to find differences in the dataset GSE7670 (without information about smoking status). It could be due to smoking is a potential confounding factor hiding differences.

**Table 1:** Characteristics and composition of subsets included in the GSEA analysis

GEO Accesion		GSE10072		GSE32863		TCGA	GSE50081	GSE47115	GSE7670
Array		Affymetrix Human Genome U133A		Illumina HumanWG-6 v3.0		IlluminaHiSeq_RNASeqV2	Affymetrix Human Genome U133 Plus 2.0	Illumina HumanHT-12 WG-DASL V4.0 R2	Affymetrix Human Genome U133A
**Histology**		Adenocarcinoma		Adenocarcinoma		Adenocarcinoma	NSCLC	NSCLC	Adenocarcinoma
**Stage**		IA–IV		IA–IV			IA–IIB	IB–IIB	
**Samples evaluated**		***n* = 71**		**116**		**447**	**81**	**16**	**52**
**Subsets in dataset**	-	4		4		2	2	1	2
**Never Smokers**	**Normal Tissue**	**31 (43.7%)**	**15 (48.4%)**	**59 (50.1%)**	**30 (50.8%)**				
	Male Female		4 (26.7%) 11 (73.3%)		7 (23.3%) 23 (76.7%)				
	**Tumoral Tissue**		**16 (51.6%)**		**29 (49.2%)**	**69 (15.4%)**	**24 (29.6%)**		
	Male Female		13 (81.2%) 3 (18.8%)		6 (20.7%) 23 (79.3%)	17 (24.6%) 52 (75.4%)	6 (25%) 18 (75%)		
**Current Smokers**	**Normal Tissue**	**40 (56.3%)**	**16 (40%)**	**57 (49.9%)**	**28 (49.1%)**				
	Male Female		4 (25%) 12 (75%)		6 (21.4%) 22 (78.6%)				
	**Tumoral Tissue**		**24 (60%)**		**29 (50.1%)**	**378 (84.6%)**	**57 (70.4%)**	**16 (100%)**	
	Male Female		16 (66.7%) 8 (33.3%)		7 (24.1%) 22 (75.9%)	187 (49.5%) 191(50.5%)	36 (63.2%) 21(36.8%)	9 (56.2%) 7 (43.8%)	
**Unknown**	**Normal Tissue**								**26 (50%)**
	Male Female								5 (19.2%) 21 (80.8%)
	**Tumoral Tissue**								**26 (50%)**
	Male Female								5 (19.2%) 21 (80.8%)

### Immune-related genes over-represented in several datasets

Due to the gene redundancy across immune gene sets, we identified “core genes” (genes associated with the enrichment signal) over-represented in different subsets for immune biological processes. The top-ten rank core genes included *APOBEC3G* (Apolipoprotein B mRNA editing enzyme, catalytic polypeptide-like 3G) that exerts innate immune activity against retroviruses and has shown tumor suppressive effects in human hepatocellular carcinoma and enhance cell radio resistance in lymphomas; [8, 9] *APOBEC3F* (Apolipoprotein B mRNA editing enzyme, catalytic polypeptide-like 3F) that showed to inhibit HIV-1 DNA Integration; [10] *CCL5* (Chemokine (C-C motif) ligand 5, a well characterized chemotactic chemokine; *CD1D* (CD1d molecule) mediate the presentation of self or microbial antigens to T cells; *LAT* (Linker for activation of T cells), a major transporter for essential amino acids into activated human T cells; [11] *TRAT1* (T cell receptor associated transmembrane adaptor 1), important to transport od CTLA-4 to the cell surface; [12] *IL32* (Interleukin 32), whose expression is increased after the activation of T-cells by mitogens or the activation of NK cells by IL-2; *CRTAM* (cytotoxic and regulatory T cell molecule), upregulated in CD4 and CD8 T cells; *CFHR1* (complement factor H-related 1), whose protein product binds to *Pseudomonas aeruginosa* elongation factor Tuf together with plasminogen and *CCR2* (chemokine [C-C motif] receptor 2) that mediates monocyte infiltration. The complete list of core genes involved in immune gene sets is shown in Table S3.

### Immune cell composition inferred from the transriptional background

**Figure 3:**
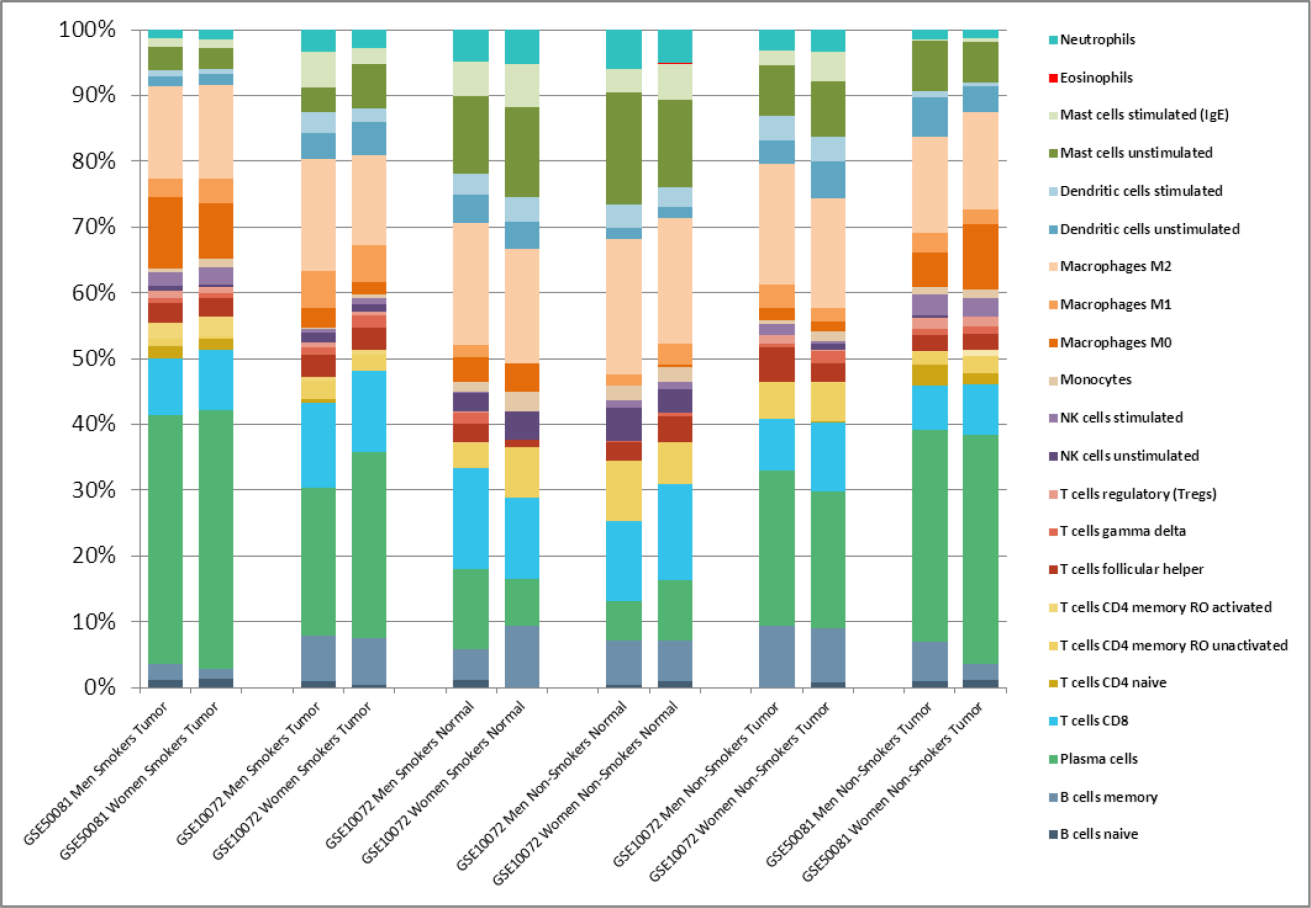
Relative leukocyte fractions evaluated by CIBERSORT in Affymetrix datasets to infer relative RNA fractions from 22 leukocyte subsets (LM22 signature) in each sample Shown are the average fractions in each dataset.

There were no differences in relative frequencies of immune cell composition in both genders according to the LM22 signature (Figure 3). An important difference was seen between healthy lung tissues vs NSCLC, regardless the smoking status or gender. The main component in tumors was plasma B cells and macrophages. In contrast, healthy lung tissues have a higher proportion of T cells CD8, mast cells and a lower proportion of plasma cells, compared to lung tumors. Detailed values of immune cell composition are described in the Table S3.

## DISCUSSION

Although gender is a prognostic factor in some malignancies, there is a lack of biological basis explaining this phenomenon [5, 13, 14]. On the other hand, the immune system plays an important role in the efficacy of the therapy, for example, tumor infiltration by lymphocytes is associated with better response to chemotherapy and trastuzumab, and also is associated with a better prognosis in breast cancer and other cancers [15, 16, 17].

There are few reports describing the value of tumor-infiltrating lymphocytes in NSCLC where a more significant prognostic factor is the presence of tumor-induced bronchus-associated lymphoid tissue (Ti-BALT) omposed by mature dendritic cell (DC)/T-cell clusters adjacent to B-cell follicles [18]. Despite of the controversial value of tumor-infiltrating lymphocytes in NSCLC, peripheral leukocytes have more prognostic relevance. A recent meta-analysis study described that a high neutrophils/lymphocyte ratio in peripheral blood is related with a poor prognosis [19]. Also, gene expression patterns of peripheral blood mononuclear cells are highly correlated with the tumor burden in NSCLC and this biological signal could disappear after tumor resection [20, 21].

A different composition of tumor infiltrating immune cells between genders might be a good explanation for our findings in the GSEA analyses and could be more easily linked to the outcomes in women patients with NSCLC; however, the similar composition of immune cells suggests differences in activation of immune pathways and interaction between immune and tumoral cells.

There are several methodologies for the analysis of various datasets to minimize the batch effect [22]. In this work we preferred to construct subsets and perform analyses in each subset individually instead of pooling data. An overall comparison in each dataset could lead to biased results due to smoking status is the main cofounder and difficult to control. Although we decide not to use a more stringent statistics (FDR < 5%), the strength of our analysis strategy is based on the multiple validation that lead to be able to identify subtle differences in biological signals that could be masked with the batch of samples or more stringent statistics.

Our results are supported by a recent report by Qu et al. (2015), providing the first evidence of gender differences of immune regulation by elements that escape X chromosome inactivation and trigger regulatory networks and activation of genes with immune function in other autosomes in a study done in primary humans T cells [23]. Our results suggest different activities of immune gene sets regardless the immune cell composition in the tumor. Genes such as *APOBEC3G* and *APOBEC3F* seem to play and important role in the immune response in NSCLC, while over-representation of *CCL5* (a gene widely studied in other malignancies), *CD1D*, *LAT*, *TRAT1*, *IL32* and others, suggest different regulatory activities of T lymphocytes in NSCLC in women compared to men. Although “Immune Response” and “Immune System Process” where the gene sets more over-represented, which is logical because include more genes that produce redundancy across gene sets, we found differences in more specific subsets such as “Regulation of Immune System Process” “T-Cell Activation”, “Regulation of Cytokine Biosynthetic Process” (Figure 2).

Recently, immune checkpoints inhibitors such as nivolumab and pembrolizumab are included in the National Comprehensive Cancer Network (NCCN) guidelines for the treatment of metastatic NSCLC subsequent to first line of chemotherapy [24]. A phase III study comparing nivolumab with docetaxel in patients with advanced non-squamous NSCLC showed that nivolumab not improved the overall survival in the sub group of women patients (HR:0.78, CI: 0.58–1.04) [25]. Likewise, the results of KEYNOTE-010 trial comparing pembrolizumab versus docetaxel in patients previously treated with platinum-doublet chemotherapy showed that pembrolizumab does not increase the progression free survival in women (HR: 1.02, CI: 0.78–1.32). Conversely, both of these immune checkpoint inhibitors improved the outcome of the subgroup of men patients [26]. This contrasting results might be explained by differences in expression of immune genes.

Biological differences of lung tumors among genders should be deeply explored in order to improve the immunotherapeutic approaches. Our study provides evidence of biological differences of NSCLC between genders and the basis for the distinct clinical outcome.

## MATERIALS AND METHODS

### NSCLC datasets

With the aim of explore differences in immune gene sets in NSCLC between genders in a functional approach, we retrieved normalized gene expression data of five NSCLC datasets (GSE10072, GSE32863, GSE50081, GSE47115, and GSE7670) from the NCBI GEO website (http://www.ncbi.nlm.nih.gov/geo/). One RNASeq Level 3 dataset of lung adenocarcinoma was downloaded from the TCGA website (https://tcga-data.nci.nih.gov/docs/publications/luad_2014/).

### Data preprocessing

Values from datasets downloaded from the NCBI GEO were log_2_ transformed and median centered. In the TCGA dataset, expression values of “zero” were set to the overall minimum value and all data were log_2_ transformed and median centered.

### GSEA analysis

We evaluated 825 gene sets in biological processes annotated from Gene Ontology (C5 BP) in the Molecular Signature Database (MSigDB; http://www.broad.mit.edu/gsea/msigdb/msigdb_index.html). Because of the small sample size of the subsets, the GSEA was conducted with 1000 gene set permutations. The GSEA analyses were performed using the Java GSEA implementation downloaded from www.broad.mit.edu/gsea/msigdb/. Gene sets from samples of men vs women were compared. A gene set was considered enriched when it was included in the top 50 rank in at least two subsets with a *p*-value < 0.05 and a False Discovery Rate (FDR) < 25% and when was represented in only 1 gender.

To avoid the confounding effect of smoking and possible batch effects with the pooling of samples, we decided to divide each datasets in subsets according to smoking status (smokers vs no smokers) and type of tissue (normal vs tumoral) when these samples were available. Patients with unknown smoking status or former smokers were excluded from this analysis, except for patients from the dataset GSE7670 whose samples lack this information (Table 1).

### Analysis of immune cells composition from gene expression data

To investigate if signal of enrichment of immune gene sets in women is related to a different background of immune cells in the samples, gene expression analysis with the online analytical platform CIBERSORT (https://cibersort.stanford.edu/) was done. CIBERSORT quantify relative levels of the abundances of distinct cell types in a mixed cell population [27].

We evaluated our subsets profiled with Affymetrix platforms (GSE10072 and GSE50081) with the LM22 gene signature that is able to identify 22 immune cell types (LM22 signature is only validated for Affymetrix microarrays data). For this analysis, labels of affymetrix probes were replaced with gene names. Due to more than one affymetrix probe could represent a gene, genes were collapsed to the highest value. After this step, the data were quintile normalized. Analyses were done with 100 permutations with default statistical parameters. The results were filtered by a maximum *p*-value of 0.05.

## SUPPLEMENTARY MATERIALS TABLES






