# Supplementary material for: Repeated observation of immune gene sets enrichment in women with non-small cell lung cancer
Source: Oncotarget. 2016 Mar 6;7(15):20282–92. doi: 10.18632/oncotarget.7943 (PMC4991454; doi:10.18632/oncotarget.7943)
Supplement: Supplementary file 1 [file oncotarget-07-20282-s001.pdf]

Repeated observation of immune gene sets enrichment in women with non-small cell lung cancer

Supplementary Materials

Supplementry Table S1: Immune gene sets according to GO biological processes enriched in women

Dataset	Gene Ontoly Term	GO Term	NES	NOM *p*−val	FDR *q*−val	Tissue	Description
GSE50081	IMMUNE_ RESPONSE	GO:0006955	−2.141816	0	0.00495013	TUMOR	Smokers
GSE47115			−2.0355432	0	0.05654139	TUMOR	Smokers
GSE32863			−1.8873783	0	0.04999797	TUMOR	Non-Smokers
GSE32863			−2.187301	0	0.00407708	TUMOR	Smokers
TCGA			−2.1322112	0	0.110324	TUMOR	Smokers
TCGA			−1.6349066	0	0.24177992	TUMOR	Non-Smokers
GSE50081	IMMUNE_ SYSTEM_PROCESS	GO:0002376	−2.027211	0	0.01091849	TUMOR	Smokers
GSE47115			−1.9008057	0	0.08669692	TUMOR	Smokers
GSE32863			−2.025273	0	0.01954763	TUMOR	Non-Smokers
GSE32863			−2.1147306	0	0.00373852	TUMOR	Smokers
TCGA			−1.9406003	0	0.07627276	TUMOR	Smokers
TCGA			−1.644219	0	0.24277365	TUMOR	Non-Smokers
GSE50081	CELLULAR_ DEFENSE_ RESPONSE	GO:0006968	−1.8251283	0.00248756	0.05018073	TUMOR	Smokers
GSE47115			−1.6079495	0.00784929	0.19234282	TUMOR	Smokers
GSE32863			−1.9247202	0	0.04471115	TUMOR	Non-Smokers
GSE32863			−1.9325396	0.00175131	0.04304432	TUMOR	Smokers
TCGA			−1.7326139	0.00715564	0.07209199	TUMOR	Smokers
GSE50081	DEFENSE_ RESPONSE	GO:0006952	−2.2449086	0	0.0026197	TUMOR	Smokers
GSE47115			−1.746567	0	0.13567683	TUMOR	Smokers
GSE32863			−1.9226836	0	0.04044157	TUMOR	Non-Smokers
GSE32863			−2.1175356	0	0.00560778	TUMOR	Smokers
TCGA			−2.112328	0	0.06187405	TUMOR	Smokers
GSE50081	REGULATION_ OF_IMMUNE_ SYSTEM_PROCESS	GO:0002682	−1.7209327	0.00253165	0.08929511	TUMOR	Smokers
GSE47115			−1.6360501	0.0060423	0.2184723	TUMOR	Smokers
GSE10072			−1.6696321	0.00543478	0.11862037	NORMAL	Non-Smokers
GSE32863			−1.6818429	0.00176991	0.1548676	TUMOR	Smokers
TCGA			−1.6231375	0.00374532	0.23759808	TUMOR	Non-Smokers
GSE50081	CYTOKINE_ BIOSYNTHETIC_ PROCESS	GO:0042089	−1.9806778	0.00246914	0.01618297	TUMOR	Smokers
GSE50081			−1.6930139	0.00135685	0.16829406	TUMOR	Non-Smokers
GSE10072			−1.6159624	0.01408451	0.1357506	NORMAL	Non-Smokers
GSE32863			−1.55206	0.00915751	0.23396383	TUMOR	Smokers
GSE47115	HUMORAL_ IMMUNE_ RESPONSE	GO:0006959	−1.677544	0.00649351	0.19688377	TUMOR	Smokers
GSE32863			−1.6820558	0.00551471	0.16195275	TUMOR	Non-Smokers
GSE32863			−1.7178334	0.0053286	0.1281885	TUMOR	Smokers
TCGA			−1.7834922	0.00568182	0.07305726	TUMOR	Smokers

GSE50081	INFLAMMATORY_RESPONSE	GO:0006954	−2.1959457	0	0.00394936	TUMOR	Smokers
GSE32863			−1.6383127	0	0.17906307	TUMOR	Non-Smokers
GSE32863			−1.8924664	0	0.05366586	TUMOR	Smokers
TCGA			−1.9676802	0	0.09290282	TUMOR	Smokers
GSE50081	INNATE_IMMUNE_RESPONSE	GO:004508	−1.8000206	0.00833333	0.05371597	TUMOR	Smokers
GSE50081			−1.6167494	0.0130246	0.22084205	TUMOR	Non-Smokers
TCGA			−1.9162349	0.00705467	0.06925158	TUMOR	Smokers
TCGA			−1.7101098	0.00269179	0.21096279	TUMOR	Non-Smokers
GSE50081	LEUKOCYTE_ACTIVATION	GO:0045321	−1.6960053	0.002331	0.09904125	TUMOR	Smokers
GSE47115			−1.9095325	0	0.10273132	TUMOR	Smokers
GSE32863			−1.6817983	0.00189036	0.1565814	TUMOR	Non-Smokers
GSE32863			−1.569123	0.00513699	0.22044884	TUMOR	Smokers
GSE50081	REGULATION_OF_CYTOKINE_BIOSYNTHETIC_PROCESS	GO:0042035	−1.9554288	0	0.01938074	TUMOR	Smokers
GSE50081			−1.7510277	0.00442478	0.15727207	TUMOR	Non-Smokers
GSE47115			−1.6818967	0.01286174	0.21014212	TUMOR	Smokers
GSE32863			−1.6633716	0.00181159	0.16754858	TUMOR	Smokers
GSE50081	RESPONSE_TO_EXTERNAL_STIMULUS	GO:0009605	−1.8335316	0	0.04974858	TUMOR	Smokers
GSE32863			−1.8440604	0	0.06911543	TUMOR	Non-Smokers
GSE32863			−1.7571218	0	0.10927048	TUMOR	Smokers
TCGA			−1.8645161	0	0.06991231	TUMOR	Smokers
GSE50081	RESPONSE_TO_WOUNDING	GO:0009611	−1.8946304	0	0.03428209	TUMOR	Smokers
GSE32863			−1.6511477	0	0.18542273	TUMOR	Non-Smokers
GSE32863			−1.843025	0	0.07233741	TUMOR	Smokers
TCGA			−1.8400553	0	0.07388143	TUMOR	Smokers
GSE50081	T_CELL_ACTIVATION	GO:0042110	−1.699743	0.00954654	0.09822804	TUMOR	Smokers
GSE47115			−1.8975077	0	0.07193277	TUMOR	Smokers
GSE10072			−1.6644487	0.00420168	0.11739676	NORMAL	Non-Smokers
GSE32863			−1.6042974	0.01275046	0.19601727	TUMOR	Non-Smokers
GSE47115	CELL_ACTIVATION	GO:0001775	−1.9293643	0	0.12010915	TUMOR	Smokers
GSE32863			−1.5814444	0.00700525	0.22325641	TUMOR	Non-Smokers
GSE32863			−1.5563774	0.00521739	0.23158695	TUMOR	Smokers
GSE47115	CELL_CELL_SIGNALING	GO:0007267	−1.5803511	0	0.21147372	TUMOR	Smokers
GSE10072			−1.8602321	0	0.06949831	NORMAL	Non-Smokers
GSE10072			−1.8501779	0	0.00797819	TUMOR	Non−Smokers
GSE50081	CYTOKINE_METABOLIC_PROCESS	GO:0042107	−2.0086787	0	0.01374719	TUMOR	Smokers
GSE50081			−1.6498977	0.00412655	0.21309516	TUMOR	Non-Smokers
GSE10072			−1.6593872	0.00842697	0.11989322	NORMAL	Non-Smokers
GSE47115	LYMPHOCYTE_ACTIVATION	GO:0046649	−1.875324	0	0.08219407	TUMOR	Smokers
GSE10072			−1.6515247	0.0056101	0.11965143	NORMAL	Non-Smokers
GSE32863			−1.7409751	0.00174825	0.10906782	TUMOR	Non-Smokers
GSE50081	POSITIVE_REGULATION_OF_CYTOKINE_BIOSYNTHETIC_PROCESS	GO:0042108	−1.9891311	0	0.01680089	TUMOR	Smokers
GSE50081			−1.6402935	0.01424502	0.21301033	TUMOR	Non-Smokers
GSE47115			−1.7606636	0.00656814	0.16079095	TUMOR	Smokers
GSE10072	POSITIVE_REGULATION_OF_T_CELL_PROLIFERATION	GO:0042102	−1.6158615	0.02419355	0.13315406	NORMAL	Non-Smokers
GSE32863			−1.6191905	0.01325758	0.18311827	TUMOR	Non-Smokers
TCGA			−1.9364585	0	0.13259144	TUMOR	Non-Smokers

GSE50081	REGULATION_ OF_DEFENSE_ RESPONSE	GO:0031347	−2.1020842	0	0.00734997	TUMOR	Smokers
TCGA			−1.741655	0.00544465	0.07487477	TUMOR	Smokers
TCGA			−1.6764903	0.00571429	0.1941017	TUMOR	Non-Smokers
GSE47115	REGULATION_OF_ LYMPHOCYTE_ ACTIVATION	GO:0051249	−1.6254781	0.01151316	0.20639502	TUMOR	Smokers
GSE10072			−1.7079116	0.00851064	0.10882808	NORMAL	Non-Smokers
GSE32863			−1.5609378	0.02317291	0.24410667	TUMOR	Non-Smokers
GSE50081	RESPONSE_ TO_OTHER_ ORGANISM	GO:0051707	−1.8548871	0	0.04535616	TUMOR	Smokers
GSE10072			−1.7059491	0.00266312	0.10713203	NORMAL	Non-Smokers
GSE32863			−1.7908653	0	0.09373694	TUMOR	Smokers
GSE50081	ADAPTIVE_ IMMUNE_ RESPONSE _GO_0002460	GO:0002460	−1.9808283	0.00225734	0.01717944	TUMOR	Smokers
GSE47115			−1.6431261	0.01372213	0.23233375	TUMOR	Smokers
GSE10072	CELL_SURFACE_ RECEPTOR_ LINKED_SIGNAL_ TRANSDUCTION	GO:0007166	−1.5847944	0	0.12821138	TUMOR	Non-Smokers
TCGA			−2.0441449	0	0.08256079	TUMOR	Smokers
GSE47115	DEFENSE_ RESPONSE_TO_ VIRUS	GO:0051607	−1.6288024	0.02360877	0.21492128	TUMOR	Smokers
GSE32863			−1.575936	0.0291439	0.22438057	TUMOR	Smokers
GSE50081	IMMUNE_ EFFECTOR_ PROCESS	GO:0002252	−1.709698	0.00980392	0.09298511	TUMOR	Smokers
GSE50081			−1.6035817	0.01014493	0.2239497	TUMOR	Non-Smokers
GSE10072	IMMUNE_ SYSTEM_ DEVELOPMENT	GO:0002520	−1.7130586	0	0.11296329	NORMAL	Non-Smokers
GSE32863			−1.8994037	0	0.04719496	TUMOR	Non-Smokers
GSE50081	INTERLEUKIN_8_ PRODUCTION	GO:0032637	−1.7731749	0.00860215	0.06707466	TUMOR	Smokers
GSE32863			−1.738181	0.00369004	0.11667174	TUMOR	Smokers
GSE50081	LEUKOCYTE_ CHEMOTAXIS	GO:0030595	−2.0317585	0	0.01258163	TUMOR	Smokers
GSE32863			−1.9470302	0	0.04376491	TUMOR	Smokers
GSE10072	LEUKOCYTE_ DIFFERENTIATION	GO:0002521	−1.6486496	0.00983146	0.1197386	NORMAL	Non-Smokers
GSE32863			−1.6513519	0.00902527	0.16752724	TUMOR	Smokers
GSE32863	POSITIVE_ REGULATION_OF_ LYMPHOCYTE_ ACTIVATION	GO:0051251	−1.6488091	0.015625	0.17774025	TUMOR	Non-Smokers
TCGA			−1.8128265	0.00135685	0.17338015	TUMOR	Non-Smokers
GSE50081	POSITIVE_ REGULATION_OF_ RESPONSE_TO_ STIMULUS	GO:0048584	−2.0761623	0	0.00875345	TUMOR	Smokers
GSE32863			−1.5888414	0.01241135	0.20886502	TUMOR	Smokers
GSE32863	POSITIVE_ REGULATION_ OF_T_CELL_ ACTIVATION	GO:0050870	−1.6226203	0.02010969	0.18688846	TUMOR	Non-Smokers
TCGA			−1.8057642	0.00141844	0.15253495	TUMOR	Non-Smokers
GSE50081	REGULATION_ OF_IMMUNE_ RESPONSE	GO:0050776	−1.7685797	0.00227273	0.06802931	TUMOR	Smokers
TCGA			−1.5875516	0.01312336	0.24561612	TUMOR	Non-Smokers
GSE50081	REGULATION_ OF_RESPONSE_ TO_EXTERNAL_ STIMULUS	GO:0032101	−1.8560536	0.00421053	0.04670648	TUMOR	Smokers
GSE32863			−1.6045786	0.03345725	0.19964395	TUMOR	Smokers

GSE50081	REGULATION_OF_ RESPONSE_TO_ STIMULUS	GO:0048583	−2.0516105	0	0.0110049	TUMOR	Smokers
GSE32863			−1.7097533	0	0.13092537	TUMOR	Smokers
GSE47115	REGULATION_ OF_T_CELL_ ACTIVATION	GO:0050863	−1.7484587	0.00519031	0.14171112	TUMOR	Smokers
GSE10072			−1.8635097	0.00297177	0.07846844	NORMAL	Non-Smokers
GSE50081	RESPONSE_TO_ BIOTIC_STIMULUS	GO:0009607	−1.6900933	0	0.10112421	TUMOR	Smokers
GSE10072			−1.6458721	0	0.12015209	NORMAL	Non-Smokers
GSE50081	RESPONSE_TO_ VIRUS	GO:0009615	−1.81758	0.00251889	0.04865548	TUMOR	Smokers
GSE32863			−1.8700213	0.0018315	0.05679273	TUMOR	Smokers

Supplementry Table S2: Results of gsea analysis in all cohorts
Supplementry Table S2.1: GSE10072: Never smokers, normal tissue, enriched in male

Supplementry Table S3: Overrepresented genes related to immune system in all cohorts

Supplementry Table S4:

CIBERSORT-inferred fractions of tumor-associated leukocytes in NSCLC

Data set	Platform	No. samples shown (P<0.05)	Total samples analyzed	B cells naive	B cells memory	Plasma cells	T cells CD8	T cells CD4 naive	T cells CD4 memory RO unactivated	T cells CD4 memory RO activated	T cells follicular helper	T cells gamma delta	T cells regulatory (Tregs)	NK cells unstimulated	NK cells stimulated	Monocytes	Macrophages M0	Macrophages M1	Macrophages M2	Dendritic cells unstimulated	Dendritic cells stimulated	Mast cells unstimulated	Mast cells stimulated (IgE)	Eosinophils	Neutrophils
GSE50081 Men Smokers Tumor	HGU133Plus2	36	36	0,01	0,03	0,38	0,09	0,02	0,01	0,02	0,03	0,01	0,01	0,01	0,02	0,01	0,11	0,03	0,14	0,01	0,01	0,04	0,01	0,00	0,01
GSE50081 Women Smokers Tumor	HGU133Plus2	20	21	0,01	0,01	0,39	0,09	0,02	0,01	0,03	0,03	0,01	0,01	0,00	0,03	0,01	0,08	0,04	0,14	0,02	0,01	0,03	0,01	0,00	0,02
GSE10072 Men Smokers Tumor	HGU133A	16	16	0,01	0,07	0,22	0,13	0,01	0,03	0,00	0,03	0,01	0,01	0,01	0,00	0,00	0,03	0,06	0,17	0,04	0,03	0,04	0,05	0,00	0,03
GSE10072 Women Smokers Tumor	HGU133A	8	8	0,00	0,07	0,28	0,12	0,00	0,02	0,01	0,03	0,02	0,01	0,01	0,01	0,01	0,02	0,05	0,14	0,05	0,02	0,07	0,02	0,00	0,03
GSE10072 Men Smokers Normal	HGU133A	12	12	0,01	0,05	0,12	0,15	0,00	0,04	0,00	0,03	0,02	0,00	0,03	0,00	0,01	0,04	0,02	0,19	0,04	0,03	0,12	0,05	0,00	0,05
GSE10072 Women Smokers Normal	HGU133A	4	4	0,00	0,09	0,07	0,12	0,00	0,08	0,00	0,01	0,00	0,00	0,04	0,00	0,03	0,04	0,00	0,17	0,04	0,04	0,14	0,07	0,00	0,05
GSE10072 Men Non-Smokers Normal	HGU133A	4	4	0,00	0,07	0,06	0,12	0,00	0,09	0,00	0,03	0,00	0,00	0,05	0,01	0,02	0,00	0,02	0,21	0,02	0,04	0,17	0,04	0,00	0,06
GSE10072 Women Non-Smokers Normal	HGU133A	11	11	0,01	0,06	0,09	0,15	0,00	0,06	0,00	0,04	0,01	0,00	0,03	0,01	0,02	0,00	0,03	0,19	0,02	0,03	0,13	0,05	0,00	0,05
GSE10072 Men Non-Smokers Tumor	HGU133A	3	3	0,00	0,09	0,24	0,08	0,00	0,06	0,00	0,05	0,01	0,01	0,00	0,02	0,01	0,02	0,04	0,18	0,04	0,04	0,08	0,02	0,00	0,03
GSE10072 Women Non-Smokers Tumor	HGU133A	13	13	0,01	0,08	0,21	0,10	0,00	0,06	0,00	0,03	0,02	0,00	0,01	0,00	0,01	0,02	0,02	0,17	0,06	0,04	0,08	0,04	0,00	0,03
GSE50081 Men Non-Smokers Tumor	HGU133Plus2	6	6	0,01	0,06	0,32	0,07	0,03	0,02	0,00	0,03	0,01	0,02	0,00	0,03	0,01	0,05	0,03	0,15	0,06	0,01	0,08	0,00	0,00	0,01
GSE50081 Women Non-Smokers Tumor	HGU133Plus2	18	18	0,01	0,02	0,35	0,08	0,02	0,03	0,01	0,02	0,01	0,01	0,00	0,03	0,01	0,10	0,02	0,15	0,04	0,01	0,06	0,01	0,00	0,01
