# Supplementary material for: Repeated observation of immune gene sets enrichment in women with non-small cell lung cancer
Source: Oncotarget. 2016 Mar 6;7(15):20282–92. doi: 10.18632/oncotarget.7943 (PMC4991454; doi:10.18632/oncotarget.7943)
Supplement: Supplementary file 2 [file oncotarget-07-20282-s002.docx]

**TABLE S2.- RESULTS OF GSEA ANALYSIS IN ALL COHORTS**

**Table S2.1.- GSE10072: never Smokers, normal tissue, enriched in male.**

| **NAME** | **GS<br> follow link to MSigDB** | **GS DETAILS** | **SIZE** | **ES** | **NES** | **NOM p-val** | **FDR q-val** | **FWER p-val** | **RANK AT MAX** | **LEADING EDGE** |
| --- | --- | --- | --- | --- | --- | --- | --- | --- | --- | --- |
| **CELLULAR_RESPIRATION** | **CELLULAR_RESPIRATION** | **Details ...** | **19** | **0.6032349** | **1.9506924** | **0.009375** | **0.8323668** | **0.552** | **4123** | **tags=79%, list=31%, signal=114%** |
| **AMYLOID_PRECURSOR_PROTEIN_METABOLIC_PROCESS** | **AMYLOID_PRECURSOR_PROTEIN_METABOLIC_PROCESS** | **Details ...** | **8** | **0.7756919** | **1.9368434** | **0.00265957** | **0.47103718** | **0.593** | **1632** | **tags=50%, list=12%, signal=57%** |
| **MRNA_PROCESSING_GO_0006397** | **MRNA_PROCESSING_GO_0006397** | **Details ...** | **45** | **0.46537387** | **1.8728447** | **0** | **0.5356186** | **0.785** | **1493** | **tags=31%, list=11%, signal=35%** |
| **MRNA_METABOLIC_PROCESS** | **MRNA_METABOLIC_PROCESS** | **Details ...** | **56** | **0.415511** | **1.7804103** | **0** | **0.73691225** | **0.937** | **3464** | **tags=46%, list=26%, signal=62%** |
| **NOTCH_SIGNALING_PATHWAY** | **NOTCH_SIGNALING_PATHWAY** | **Details ...** | **8** | **0.6934396** | **1.7741327** | **0.0166205** | **0.61339647** | **0.944** | **2748** | **tags=88%, list=21%, signal=110%** |
| **GLYCOPROTEIN_CATABOLIC_PROCESS** | **GLYCOPROTEIN_CATABOLIC_PROCESS** | **Details ...** | **11** | **0.6331621** | **1.7718463** | **0.01907357** | **0.51751554** | **0.947** | **1632** | **tags=45%, list=12%, signal=52%** |
| **RNA_3END_PROCESSING** | **RNA_3END_PROCESSING** | **Details ...** | **8** | **0.68080443** | **1.7224174** | **0.01511335** | **0.6076836** | **0.989** | **3937** | **tags=88%, list=30%, signal=124%** |
| **AEROBIC_RESPIRATION** | **AEROBIC_RESPIRATION** | **Details ...** | **15** | **0.5631429** | **1.701751** | **0.0252809** | **0.6053941** | **0.992** | **4123** | **tags=73%, list=31%, signal=106%** |
| **RNA_SPLICING** | **RNA_SPLICING** | **Details ...** | **59** | **0.38924283** | **1.6979047** | **0.00369004** | **0.54836255** | **0.992** | **1470** | **tags=27%, list=11%, signal=30%** |
| **POSITIVE_REGULATION_OF_I_KAPPAB_KINASE_NF_KAPPAB_CASCADE** | **POSITIVE_REGULATION_OF_I_KAPPAB_KINASE_NF_KAPPAB_CASCADE** | **Details ...** | **66** | **0.38829508** | **1.6966021** | **0** | **0.4973977** | **0.992** | **3760** | **tags=47%, list=28%, signal=65%** |
| **NEGATIVE_REGULATION_OF_CELLULAR_COMPONENT_ORGANIZATION_AND_BIOGENESIS** | **NEGATIVE_REGULATION_OF_CELLULAR_COMPONENT_ORGANIZATION_AND_BIOGENESIS** | **Details ...** | **25** | **0.48815614** | **1.6950103** | **0.01311475** | **0.4549289** | **0.992** | **1247** | **tags=28%, list=9%, signal=31%** |
| **ER_TO_GOLGI_VESICLE_MEDIATED_TRANSPORT** | **ER_TO_GOLGI_VESICLE_MEDIATED_TRANSPORT** | **Details ...** | **12** | **0.5882183** | **1.677906** | **0.02571429** | **0.4580839** | **0.996** | **3534** | **tags=67%, list=27%, signal=91%** |
| **RNA_SPLICINGVIA_TRANSESTERIFICATION_REACTIONS** | **RNA_SPLICINGVIA_TRANSESTERIFICATION_REACTIONS** | **Details ...** | **17** | **0.53902537** | **1.6669756** | **0.0375** | **0.4492014** | **0.996** | **1239** | **tags=29%, list=9%, signal=32%** |
| **ISOPRENOID_METABOLIC_PROCESS** | **ISOPRENOID_METABOLIC_PROCESS** | **Details ...** | **10** | **0.5864362** | **1.6279838** | **0.0458221** | **0.511409** | **0.999** | **71** | **tags=20%, list=1%, signal=20%** |
| **REGULATION_OF_SYNAPSE_STRUCTURE_AND_ACTIVITY** | **REGULATION_OF_SYNAPSE_STRUCTURE_AND_ACTIVITY** | **Details ...** | **7** | **0.68199414** | **1.6157954** | **0.04359673** | **0.50564784** | **1** | **1976** | **tags=43%, list=15%, signal=50%** |
| **SPLICEOSOME_ASSEMBLY** | **SPLICEOSOME_ASSEMBLY** | **Details ...** | **10** | **0.5947261** | **1.5934817** | **0.04407714** | **0.5317008** | **1** | **3041** | **tags=60%, list=23%, signal=78%** |
| **REGULATION_OF_I_KAPPAB_KINASE_NF_KAPPAB_CASCADE** | **REGULATION_OF_I_KAPPAB_KINASE_NF_KAPPAB_CASCADE** | **Details ...** | **71** | **0.36350206** | **1.5847014** | **0.00930233** | **0.5230526** | **1** | **2143** | **tags=28%, list=16%, signal=33%** |
| **PROTEIN_MATURATION** | **PROTEIN_MATURATION** | **Details ...** | **8** | **0.63262653** | **1.560227** | **0.06434316** | **0.55665505** | **1** | **2625** | **tags=75%, list=20%, signal=93%** |
| **VESICLE_LOCALIZATION** | **VESICLE_LOCALIZATION** | **Details ...** | **10** | **0.5833664** | **1.5586698** | **0.05555556** | **0.531539** | **1** | **2406** | **tags=50%, list=18%, signal=61%** |
| **GOLGI_VESICLE_TRANSPORT** | **GOLGI_VESICLE_TRANSPORT** | **Details ...** | **41** | **0.3825507** | **1.5548733** | **0.02173913** | **0.5140541** | **1** | **2708** | **tags=41%, list=20%, signal=52%** |
| **PHOSPHOINOSITIDE_BIOSYNTHETIC_PROCESS** | **PHOSPHOINOSITIDE_BIOSYNTHETIC_PROCESS** | **Details ...** | **18** | **0.46671572** | **1.5474521** | **0.05198777** | **0.5080757** | **1** | **2093** | **tags=44%, list=16%, signal=53%** |
| **RESPONSE_TO_HEAT** | **RESPONSE_TO_HEAT** | **Details ...** | **10** | **0.558734** | **1.5216589** | **0.07046071** | **0.5493478** | **1** | **2427** | **tags=60%, list=18%, signal=73%** |
| **POST_GOLGI_VESICLE_MEDIATED_TRANSPORT** | **POST_GOLGI_VESICLE_MEDIATED_TRANSPORT** | **Details ...** | **13** | **0.50447804** | **1.4937294** | **0.0661157** | **0.59857094** | **1** | **1552** | **tags=38%, list=12%, signal=43%** |
| **RNA_PROCESSING** | **RNA_PROCESSING** | **Details ...** | **117** | **0.30442324** | **1.4926126** | **0.01117318** | **0.5764428** | **1** | **1493** | **tags=22%, list=11%, signal=25%** |
| **PROTEIN_AMINO_ACID_LIPIDATION** | **PROTEIN_AMINO_ACID_LIPIDATION** | **Details ...** | **21** | **0.44178653** | **1.4850131** | **0.06382979** | **0.5741798** | **1** | **2093** | **tags=43%, list=16%, signal=51%** |
| **COFACTOR_METABOLIC_PROCESS** | **COFACTOR_METABOLIC_PROCESS** | **Details ...** | **47** | **0.36025944** | **1.4674639** | **0.05019305** | **0.6008135** | **1** | **2990** | **tags=40%, list=22%, signal=52%** |
| **REGULATION_OF_CELLULAR_COMPONENT_ORGANIZATION_AND_BIOGENESIS** | **REGULATION_OF_CELLULAR_COMPONENT_ORGANIZATION_AND_BIOGENESIS** | **Details ...** | **93** | **0.3166266** | **1.4656674** | **0.0201005** | **0.58406216** | **1** | **2288** | **tags=27%, list=17%, signal=32%** |
| **NEGATIVE_REGULATION_OF_CYTOSKELETON_ORGANIZATION_AND_BIOGENESIS** | **NEGATIVE_REGULATION_OF_CYTOSKELETON_ORGANIZATION_AND_BIOGENESIS** | **Details ...** | **10** | **0.5297905** | **1.4592046** | **0.07969151** | **0.58069783** | **1** | **1247** | **tags=30%, list=9%, signal=33%** |
| **COENZYME_METABOLIC_PROCESS** | **COENZYME_METABOLIC_PROCESS** | **Details ...** | **33** | **0.38011432** | **1.4505428** | **0.06397306** | **0.58374953** | **1** | **2990** | **tags=45%, list=22%, signal=58%** |
| **ESTABLISHMENT_OF_VESICLE_LOCALIZATION** | **ESTABLISHMENT_OF_VESICLE_LOCALIZATION** | **Details ...** | **9** | **0.5491539** | **1.4424891** | **0.09972299** | **0.5852962** | **1** | **2406** | **tags=44%, list=18%, signal=54%** |
| **COFACTOR_BIOSYNTHETIC_PROCESS** | **COFACTOR_BIOSYNTHETIC_PROCESS** | **Details ...** | **21** | **0.4330472** | **1.4353946** | **0.08411215** | **0.5860086** | **1** | **1851** | **tags=29%, list=14%, signal=33%** |
| **APOPTOTIC_NUCLEAR_CHANGES** | **APOPTOTIC_NUCLEAR_CHANGES** | **Details ...** | **16** | **0.45845312** | **1.4317943** | **0.09941521** | **0.578034** | **1** | **3320** | **tags=69%, list=25%, signal=91%** |
| **REGULATION_OF_CHROMOSOME_ORGANIZATION_AND_BIOGENESIS** | **REGULATION_OF_CHROMOSOME_ORGANIZATION_AND_BIOGENESIS** | **Details ...** | **9** | **0.5627373** | **1.4311317** | **0.11053985** | **0.5619607** | **1** | **1984** | **tags=33%, list=15%, signal=39%** |
| **ESTABLISHMENT_AND_OR_MAINTENANCE_OF_CHROMATIN_ARCHITECTURE** | **ESTABLISHMENT_AND_OR_MAINTENANCE_OF_CHROMATIN_ARCHITECTURE** | **Details ...** | **60** | **0.31943125** | **1.4011508** | **0.04583333** | **0.6242056** | **1** | **1734** | **tags=28%, list=13%, signal=32%** |
| **POSITIVE_REGULATION_OF_SIGNAL_TRANSDUCTION** | **POSITIVE_REGULATION_OF_SIGNAL_TRANSDUCTION** | **Details ...** | **93** | **0.3045802** | **1.393945** | **0.04639175** | **0.62530637** | **1** | **2485** | **tags=28%, list=19%, signal=34%** |
| **COENZYME_BIOSYNTHETIC_PROCESS** | **COENZYME_BIOSYNTHETIC_PROCESS** | **Details ...** | **10** | **0.5207538** | **1.39305** | **0.11917099** | **0.6106118** | **1** | **1851** | **tags=40%, list=14%, signal=46%** |
| **RHO_PROTEIN_SIGNAL_TRANSDUCTION** | **RHO_PROTEIN_SIGNAL_TRANSDUCTION** | **Details ...** | **30** | **0.36960694** | **1.3894073** | **0.05862069** | **0.60322076** | **1** | **1476** | **tags=27%, list=11%, signal=30%** |
| **FATTY_ACID_BIOSYNTHETIC_PROCESS** | **FATTY_ACID_BIOSYNTHETIC_PROCESS** | **Details ...** | **14** | **0.4703242** | **1.3885747** | **0.11041009** | **0.5899712** | **1** | **3151** | **tags=64%, list=24%, signal=84%** |
| **PROTEIN_TARGETING_TO_MEMBRANE** | **PROTEIN_TARGETING_TO_MEMBRANE** | **Details ...** | **8** | **0.5484385** | **1.3773754** | **0.11471321** | **0.6026955** | **1** | **2554** | **tags=63%, list=19%, signal=77%** |
| **I_KAPPAB_KINASE_NF_KAPPAB_CASCADE** | **I_KAPPAB_KINASE_NF_KAPPAB_CASCADE** | **Details ...** | **88** | **0.29147005** | **1.3768934** | **0.03225806** | **0.58892965** | **1** | **2143** | **tags=24%, list=16%, signal=28%** |
| **ACTIN_POLYMERIZATION_AND_OR_DEPOLYMERIZATION** | **ACTIN_POLYMERIZATION_AND_OR_DEPOLYMERIZATION** | **Details ...** | **18** | **0.4275744** | **1.3765998** | **0.11641791** | **0.5752509** | **1** | **789** | **tags=28%, list=6%, signal=29%** |
| **PROTEIN_DNA_COMPLEX_ASSEMBLY** | **PROTEIN_DNA_COMPLEX_ASSEMBLY** | **Details ...** | **36** | **0.35323688** | **1.3565419** | **0.0942029** | **0.6143719** | **1** | **1734** | **tags=31%, list=13%, signal=35%** |
| **RESPONSE_TO_OXIDATIVE_STRESS** | **RESPONSE_TO_OXIDATIVE_STRESS** | **Details ...** | **38** | **0.3436045** | **1.3472952** | **0.07936508** | **0.6243808** | **1** | **2544** | **tags=39%, list=19%, signal=49%** |
| **PROTEIN_POLYUBIQUITINATION** | **PROTEIN_POLYUBIQUITINATION** | **Details ...** | **10** | **0.4919195** | **1.3327789** | **0.14076246** | **0.65208346** | **1** | **2625** | **tags=40%, list=20%, signal=50%** |
| **PROTEIN_POLYMERIZATION** | **PROTEIN_POLYMERIZATION** | **Details ...** | **16** | **0.42257243** | **1.3304523** | **0.13636364** | **0.64450836** | **1** | **1326** | **tags=31%, list=10%, signal=35%** |
| **PHOSPHOINOSITIDE_METABOLIC_PROCESS** | **PHOSPHOINOSITIDE_METABOLIC_PROCESS** | **Details ...** | **22** | **0.38952655** | **1.3300027** | **0.11280488** | **0.6319664** | **1** | **3249** | **tags=50%, list=24%, signal=66%** |
| **ACTIN_FILAMENT_POLYMERIZATION** | **ACTIN_FILAMENT_POLYMERIZATION** | **Details ...** | **11** | **0.46927577** | **1.3261825** | **0.11904762** | **0.6281134** | **1** | **789** | **tags=27%, list=6%, signal=29%** |
| **NEGATIVE_REGULATION_OF_CELL_ADHESION** | **NEGATIVE_REGULATION_OF_CELL_ADHESION** | **Details ...** | **16** | **0.42306325** | **1.3204807** | **0.12394366** | **0.6301821** | **1** | **1582** | **tags=44%, list=12%, signal=50%** |
| **REGULATION_OF_ORGANELLE_ORGANIZATION_AND_BIOGENESIS** | **REGULATION_OF_ORGANELLE_ORGANIZATION_AND_BIOGENESIS** | **Details ...** | **34** | **0.34018517** | **1.3102143** | **0.12772585** | **0.64489985** | **1** | **2047** | **tags=26%, list=15%, signal=31%** |
| **NUCLEOTIDE_EXCISION_REPAIR** | **NUCLEOTIDE_EXCISION_REPAIR** | **Details ...** | **19** | **0.3897292** | **1.308552** | **0.12426036** | **0.63660294** | **1** | **2930** | **tags=37%, list=22%, signal=47%** |
| **DNA_DAMAGE_RESPONSESIGNAL_TRANSDUCTION_BY_P53_CLASS_MEDIATOR** | **DNA_DAMAGE_RESPONSESIGNAL_TRANSDUCTION_BY_P53_CLASS_MEDIATOR** | | **12** | **0.45652056** | **1.3078904** | **0.1589041** | **0.6260329** | **1** | **1641** | **tags=33%, list=12%, signal=38%** |
| **REGULATION_OF_RHO_PROTEIN_SIGNAL_TRANSDUCTION** | **REGULATION_OF_RHO_PROTEIN_SIGNAL_TRANSDUCTION** | | **7** | **0.53051555** | **1.300607** | **0.16445623** | **0.63287264** | **1** | **4101** | **tags=57%, list=31%, signal=83%** |
| **REGULATION_OF_ACTIN_POLYMERIZATION_AND_OR_DEPOLYMERIZATION** | **REGULATION_OF_ACTIN_POLYMERIZATION_AND_OR_DEPOLYMERIZATION** | | **11** | **0.45706454** | **1.2856545** | **0.1522388** | **0.6618381** | **1** | **789** | **tags=27%, list=6%, signal=29%** |
| **REGULATION_OF_CELLULAR_COMPONENT_SIZE** | **REGULATION_OF_CELLULAR_COMPONENT_SIZE** | | **12** | **0.44299677** | **1.2680178** | **0.17553191** | **0.6993906** | **1** | **789** | **tags=25%, list=6%, signal=27%** |
| **ACTIN_CYTOSKELETON_ORGANIZATION_AND_BIOGENESIS** | **ACTIN_CYTOSKELETON_ORGANIZATION_AND_BIOGENESIS** | | **86** | **0.27465716** | **1.2631173** | **0.0952381** | **0.70158786** | **1** | **2406** | **tags=27%, list=18%, signal=32%** |
| **PROTEIN_RNA_COMPLEX_ASSEMBLY** | **PROTEIN_RNA_COMPLEX_ASSEMBLY** | | **37** | **0.32890996** | **1.2594436** | **0.14716981** | **0.6996276** | **1** | **1239** | **tags=19%, list=9%, signal=21%** |
| **TRANSFORMING_GROWTH_FACTOR_BETA_RECEPTOR_SIGNALING_PATHWAY** | **TRANSFORMING_GROWTH_FACTOR_BETA_RECEPTOR_SIGNALING_PATHWAY** | | **34** | **0.33133724** | **1.2553306** | **0.16724738** | **0.6981577** | **1** | **3653** | **tags=50%, list=27%, signal=69%** |
| **CELL_SUBSTRATE_ADHESION** | **CELL_SUBSTRATE_ADHESION** | | **35** | **0.3264157** | **1.2543229** | **0.15523465** | **0.68933076** | **1** | **1841** | **tags=31%, list=14%, signal=36%** |
| **RESPONSE_TO_TEMPERATURE_STIMULUS** | **RESPONSE_TO_TEMPERATURE_STIMULUS** | | **16** | **0.4065771** | **1.2494869** | **0.17589577** | **0.6923567** | **1** | **2496** | **tags=50%, list=19%, signal=61%** |
| **REGULATION_OF_ACTIN_FILAMENT_LENGTH** | **REGULATION_OF_ACTIN_FILAMENT_LENGTH** | | **12** | **0.44299677** | **1.244264** | **0.21578947** | **0.6946744** | **1** | **789** | **tags=25%, list=6%, signal=27%** |
| **RESPONSE_TO_HORMONE_STIMULUS** | **RESPONSE_TO_HORMONE_STIMULUS** | | **25** | **0.34817645** | **1.2404002** | **0.18333334** | **0.6943229** | **1** | **2255** | **tags=32%, list=17%, signal=38%** |
| **REGULATION_OF_ENDOCYTOSIS** | **REGULATION_OF_ENDOCYTOSIS** | | **14** | **0.41338158** | **1.225362** | **0.21022727** | **0.72694314** | **1** | **1323** | **tags=29%, list=10%, signal=32%** |
| **TRANSCRIPTION_INITIATION_FROM_RNA_POLYMERASE_II_PROMOTER** | **TRANSCRIPTION_INITIATION_FROM_RNA_POLYMERASE_II_PROMOTER** | | **18** | **0.39113113** | **1.2226378** | **0.21165644** | **0.7236822** | **1** | **1635** | **tags=22%, list=12%, signal=25%** |
| **TRANSMEMBRANE_RECEPTOR_PROTEIN_SERINE_THREONINE_KINASE_SIGNALING_PATHWAY** | **TRANSMEMBRANE_RECEPTOR_PROTEIN_SERINE_THREONINE_KINASE_SIGNALING_PATHWAY** | | **42** | **0.30693603** | **1.2157998** | **0.16494845** | **0.73262674** | **1** | **3653** | **tags=50%, list=27%, signal=69%** |
| **AXON_GUIDANCE** | **AXON_GUIDANCE** | | **19** | **0.3701552** | **1.2041652** | **0.19104478** | **0.75653523** | **1** | **735** | **tags=21%, list=6%, signal=22%** |
| **GLIOGENESIS** | **GLIOGENESIS** | | **11** | **0.4281399** | **1.1977847** | **0.2431694** | **0.7645465** | **1** | **2427** | **tags=45%, list=18%, signal=56%** |
| **ACTIN_FILAMENT_BASED_PROCESS** | **ACTIN_FILAMENT_BASED_PROCESS** | | **96** | **0.2580127** | **1.193661** | **0.17277487** | **0.76637036** | **1** | **2406** | **tags=28%, list=18%, signal=34%** |
| **LIPOPROTEIN_BIOSYNTHETIC_PROCESS** | **LIPOPROTEIN_BIOSYNTHETIC_PROCESS** | | **23** | **0.34508604** | **1.1906493** | **0.21902017** | **0.7645127** | **1** | **2093** | **tags=39%, list=16%, signal=46%** |
| **INTRACELLULAR_TRANSPORT** | **INTRACELLULAR_TRANSPORT** | | **240** | **0.2169604** | **1.1893598** | **0.0462963** | **0.7573114** | **1** | **2708** | **tags=27%, list=20%, signal=33%** |
| **STEROID_HORMONE_RECEPTOR_SIGNALING_PATHWAY** | **STEROID_HORMONE_RECEPTOR_SIGNALING_PATHWAY** | | **10** | **0.42605948** | **1.1888262** | **0.23243243** | **0.748263** | **1** | **2052** | **tags=40%, list=15%, signal=47%** |
| **CELL_MATRIX_ADHESION** | **CELL_MATRIX_ADHESION** | | **34** | **0.3131438** | **1.1856959** | **0.18245614** | **0.7475952** | **1** | **1790** | **tags=29%, list=13%, signal=34%** |
| **AGING** | **AGING** |  | **11** | **0.4293621** | **1.1797236** | **0.25153375** | **0.7554504** | **1** | **3692** | **tags=64%, list=28%, signal=88%** |
| **POSITIVE_REGULATION_OF_PROTEIN_SECRETION** | **POSITIVE_REGULATION_OF_PROTEIN_SECRETION** | | **6** | **0.5165205** | **1.1779499** | **0.25737265** | **0.7501076** | **1** | **67** | **tags=17%, list=1%, signal=17%** |
| **RNA_EXPORT_FROM_NUCLEUS** | **RNA_EXPORT_FROM_NUCLEUS** | | **16** | **0.3705912** | **1.1731566** | **0.22392638** | **0.7537247** | **1** | **2641** | **tags=38%, list=20%, signal=47%** |
| **NEGATIVE_REGULATION_OF_PROGRAMMED_CELL_DEATH** | **NEGATIVE_REGULATION_OF_PROGRAMMED_CELL_DEATH** | | **131** | **0.23419589** | **1.167183** | **0.1573604** | **0.7612504** | **1** | **2983** | **tags=34%, list=22%, signal=44%** |
| **INTRACELLULAR_RECEPTOR_MEDIATED_SIGNALING_PATHWAY** | **INTRACELLULAR_RECEPTOR_MEDIATED_SIGNALING_PATHWAY** | | **10** | **0.42605948** | **1.1665229** | **0.24250682** | **0.7531171** | **1** | **2052** | **tags=40%, list=15%, signal=47%** |
| **ANTI_APOPTOSIS** | **ANTI_APOPTOSIS** | | **103** | **0.24433848** | **1.1633596** | **0.1557789** | **0.7527632** | **1** | **2983** | **tags=36%, list=22%, signal=46%** |
| **MESODERM_DEVELOPMENT** | **MESODERM_DEVELOPMENT** | | **22** | **0.33456165** | **1.1614017** | **0.2310231** | **0.7486964** | **1** | **1735** | **tags=32%, list=13%, signal=37%** |
| **POSITIVE_REGULATION_OF_CYTOKINE_SECRETION** | **POSITIVE_REGULATION_OF_CYTOKINE_SECRETION** | | **5** | **0.54360443** | **1.157826** | **0.3043478** | **0.7501345** | **1** | **67** | **tags=20%, list=1%, signal=20%** |
| **GLUTAMINE_FAMILY_AMINO_ACID_METABOLIC_PROCESS** | **GLUTAMINE_FAMILY_AMINO_ACID_METABOLIC_PROCESS** | | **14** | **0.38641798** | **1.1556339** | **0.25212464** | **0.7472287** | **1** | **1807** | **tags=29%, list=14%, signal=33%** |
| **ELECTRON_TRANSPORT_GO_0006118** | **ELECTRON_TRANSPORT_GO_0006118** | | **50** | **0.27946073** | **1.1545725** | **0.2265625** | **0.740907** | **1** | **2080** | **tags=26%, list=16%, signal=31%** |
| **PROTEIN_FOLDING** | **PROTEIN_FOLDING** | | **55** | **0.27315605** | **1.1536429** | **0.20689656** | **0.7346432** | **1** | **2988** | **tags=40%, list=22%, signal=51%** |
| **REGULATION_OF_GTPASE_ACTIVITY** | **REGULATION_OF_GTPASE_ACTIVITY** | | **9** | **0.43429664** | **1.1363362** | **0.2837838** | **0.77754205** | **1** | **4160** | **tags=56%, list=31%, signal=81%** |
| **REGULATION_OF_SMALL_GTPASE_MEDIATED_SIGNAL_TRANSDUCTION** | **REGULATION_OF_SMALL_GTPASE_MEDIATED_SIGNAL_TRANSDUCTION** | | **14** | **0.38051942** | **1.1316382** | **0.26815644** | **0.7836299** | **1** | **331** | **tags=14%, list=2%, signal=15%** |
| **PEPTIDE_METABOLIC_PROCESS** | **PEPTIDE_METABOLIC_PROCESS** | | **9** | **0.43163776** | **1.1263162** | **0.29787233** | **0.7904587** | **1** | **1891** | **tags=33%, list=14%, signal=39%** |
| **NEGATIVE_REGULATION_OF_APOPTOSIS** | **NEGATIVE_REGULATION_OF_APOPTOSIS** | | **130** | **0.22622639** | **1.1253242** | **0.1897436** | **0.7843869** | **1** | **2983** | **tags=34%, list=22%, signal=43%** |
| **REGULATION_OF_NEUROGENESIS** | **REGULATION_OF_NEUROGENESIS** | | **12** | **0.38449776** | **1.1248027** | **0.315** | **0.7770347** | **1** | **916** | **tags=25%, list=7%, signal=27%** |
| **ESTABLISHMENT_OF_ORGANELLE_LOCALIZATION** | **ESTABLISHMENT_OF_ORGANELLE_LOCALIZATION** | | **15** | **0.36276323** | **1.1244897** | **0.2748538** | **0.76922035** | **1** | **2406** | **tags=33%, list=18%, signal=41%** |
| **REGULATION_OF_RAS_PROTEIN_SIGNAL_TRANSDUCTION** | **REGULATION_OF_RAS_PROTEIN_SIGNAL_TRANSDUCTION** | | **11** | **0.40570444** | **1.1232276** | **0.29427794** | **0.764289** | **1** | **331** | **tags=18%, list=2%, signal=19%** |
| **ICOSANOID_METABOLIC_PROCESS** | **ICOSANOID_METABOLIC_PROCESS** | | **17** | **0.35006398** | **1.1146203** | **0.31092438** | **0.7814275** | **1** | **1811** | **tags=35%, list=14%, signal=41%** |
| **CELL_STRUCTURE_DISASSEMBLY_DURING_APOPTOSIS** | **CELL_STRUCTURE_DISASSEMBLY_DURING_APOPTOSIS** | | **16** | **0.3582362** | **1.1107987** | **0.32463768** | **0.78403205** | **1** | **3320** | **tags=69%, list=25%, signal=91%** |
| **DNA_CATABOLIC_PROCESS** | **DNA_CATABOLIC_PROCESS** | | **20** | **0.33733398** | **1.1103841** | **0.278481** | **0.77677935** | **1** | **3320** | **tags=50%, list=25%, signal=66%** |
| **NUCLEOBASENUCLEOSIDENUCLEOTIDE_AND_NUCLEIC_ACID_TRANSPORT** | **NUCLEOBASENUCLEOSIDENUCLEOTIDE_AND_NUCLEIC_ACID_TRANSPORT** | | **24** | **0.31908843** | **1.1032574** | **0.31111112** | **0.7888924** | **1** | **3523** | **tags=46%, list=26%, signal=62%** |
| **FATTY_ACID_METABOLIC_PROCESS** | **FATTY_ACID_METABOLIC_PROCESS** | | **53** | **0.25991514** | **1.0996817** | **0.28057554** | **0.79104847** | **1** | **1521** | **tags=23%, list=11%, signal=25%** |
| **GLYCEROPHOSPHOLIPID_METABOLIC_PROCESS** | **GLYCEROPHOSPHOLIPID_METABOLIC_PROCESS** | | **36** | **0.28136727** | **1.0941534** | **0.2908497** | **0.8002962** | **1** | **2093** | **tags=33%, list=16%, signal=39%** |
| **CELL_RECOGNITION** | **CELL_RECOGNITION** | | **14** | **0.3652984** | **1.0901684** | **0.32743362** | **0.8042876** | **1** | **1206** | **tags=36%, list=9%, signal=39%** |
| **MICROTUBULE_BASED_PROCESS** | **MICROTUBULE_BASED_PROCESS** | | **72** | **0.24419367** | **1.086981** | **0.29186603** | **0.80577886** | **1** | **1247** | **tags=17%, list=9%, signal=18%** |
| **LIPOPROTEIN_METABOLIC_PROCESS** | **LIPOPROTEIN_METABOLIC_PROCESS** | | **30** | **0.28978023** | **1.0743378** | **0.32507738** | **0.8370784** | **1** | **2093** | **tags=37%, list=16%, signal=43%** |
| **GLYCEROPHOSPHOLIPID_BIOSYNTHETIC_PROCESS** | **GLYCEROPHOSPHOLIPID_BIOSYNTHETIC_PROCESS** | | **24** | **0.3087519** | **1.069886** | **0.34042552** | **0.8430544** | **1** | **2093** | **tags=33%, list=16%, signal=39%** |
| **NLS_BEARING_SUBSTRATE_IMPORT_INTO_NUCLEUS** | **NLS_BEARING_SUBSTRATE_IMPORT_INTO_NUCLEUS** | | **11** | **0.37630373** | **1.0596979** | **0.37931034** | **0.86840045** | **1** | **1729** | **tags=36%, list=13%, signal=42%** |
| **REGULATION_OF_SIGNAL_TRANSDUCTION** | **REGULATION_OF_SIGNAL_TRANSDUCTION** | | **168** | **0.20692748** | **1.0572525** | **0.34108528** | **0.8672493** | **1** | **2143** | **tags=21%, list=16%, signal=25%** |
| **NUCLEAR_ORGANIZATION_AND_BIOGENESIS** | **NUCLEAR_ORGANIZATION_AND_BIOGENESIS** | | **21** | **0.31269** | **1.055164** | **0.3522388** | **0.8658256** | **1** | **3320** | **tags=52%, list=25%, signal=70%** |
| **ACTIN_FILAMENT_BUNDLE_FORMATION** | **ACTIN_FILAMENT_BUNDLE_FORMATION** | | **10** | **0.39389008** | **1.0525093** | **0.37179488** | **0.86575484** | **1** | **2406** | **tags=40%, list=18%, signal=49%** |
| **ESTABLISHMENT_OF_CELLULAR_LOCALIZATION** | **ESTABLISHMENT_OF_CELLULAR_LOCALIZATION** | | **297** | **0.18937163** | **1.0498923** | **0.25531915** | **0.8659788** | **1** | **1729** | **tags=18%, list=13%, signal=20%** |
| **PROTEOLYSIS** | **PROTEOLYSIS** | | **160** | **0.2025486** | **1.0487497** | **0.3148148** | **0.861625** | **1** | **2833** | **tags=30%, list=21%, signal=38%** |
| **BASE_EXCISION_REPAIR** | **BASE_EXCISION_REPAIR** | | **15** | **0.34682527** | **1.048517** | **0.37260273** | **0.8541711** | **1** | **3670** | **tags=47%, list=28%, signal=64%** |
| **RECEPTOR_MEDIATED_ENDOCYTOSIS** | **RECEPTOR_MEDIATED_ENDOCYTOSIS** | | **30** | **0.2853331** | **1.0480129** | **0.34674922** | **0.84785897** | **1** | **1076** | **tags=20%, list=8%, signal=22%** |
| **CYTOKINESIS** | **CYTOKINESIS** | | **16** | **0.3327548** | **1.0475355** | **0.39385474** | **0.84150124** | **1** | **2073** | **tags=25%, list=16%, signal=30%** |
| **ANDROGEN_RECEPTOR_SIGNALING_PATHWAY** | **ANDROGEN_RECEPTOR_SIGNALING_PATHWAY** | | **5** | **0.49327344** | **1.0474837** | **0.4101942** | **0.8339147** | **1** | **1635** | **tags=40%, list=12%, signal=46%** |
| **CHROMATIN_ASSEMBLY_OR_DISASSEMBLY** | **CHROMATIN_ASSEMBLY_OR_DISASSEMBLY** | | **25** | **0.29906678** | **1.043196** | **0.37096775** | **0.83870244** | **1** | **1734** | **tags=24%, list=13%, signal=28%** |
| **VESICLE_MEDIATED_TRANSPORT** | **VESICLE_MEDIATED_TRANSPORT** | | **169** | **0.19706047** | **1.0391662** | **0.39333335** | **0.8430779** | **1** | **1849** | **tags=20%, list=14%, signal=23%** |
| **NUCLEOTIDE_SUGAR_METABOLIC_PROCESS** | **NUCLEOTIDE_SUGAR_METABOLIC_PROCESS** | | **8** | **0.41772354** | **1.0373598** | **0.39012346** | **0.8413008** | **1** | **2658** | **tags=38%, list=20%, signal=47%** |
| **FATTY_ACID_OXIDATION** | **FATTY_ACID_OXIDATION** | | **16** | **0.33978388** | **1.0349083** | **0.39184952** | **0.84101313** | **1** | **1251** | **tags=31%, list=9%, signal=34%** |
| **NUCLEAR_EXPORT** | **NUCLEAR_EXPORT** | | **25** | **0.29631495** | **1.0318912** | **0.36607143** | **0.84327686** | **1** | **2641** | **tags=32%, list=20%, signal=40%** |
| **TRANSLATION** | **TRANSLATION** | | **136** | **0.20246167** | **1.0306937** | **0.35714287** | **0.83931684** | **1** | **1307** | **tags=11%, list=10%, signal=12%** |
| **OVULATION_CYCLE** | **OVULATION_CYCLE** | | **9** | **0.38552004** | **1.029733** | **0.39790577** | **0.8352225** | **1** | **3252** | **tags=44%, list=24%, signal=59%** |
| **CELLULAR_BIOSYNTHETIC_PROCESS** | **CELLULAR_BIOSYNTHETIC_PROCESS** | | **259** | **0.18755195** | **1.0240244** | **0.35353535** | **0.84505916** | **1** | **1347** | **tags=12%, list=10%, signal=13%** |
| **ENERGY_DERIVATION_BY_OXIDATION_OF_ORGANIC_COMPOUNDS** | **ENERGY_DERIVATION_BY_OXIDATION_OF_ORGANIC_COMPOUNDS** | | **37** | **0.27097172** | **1.0226712** | **0.42142856** | **0.84199566** | **1** | **3790** | **tags=46%, list=28%, signal=64%** |
| **HOMOPHILIC_CELL_ADHESION** | **HOMOPHILIC_CELL_ADHESION** | | **12** | **0.36074424** | **1.0209578** | **0.4082192** | **0.84064776** | **1** | **562** | **tags=17%, list=4%, signal=17%** |
| **RIBONUCLEOPROTEIN_COMPLEX_BIOGENESIS_AND_ASSEMBLY** | **RIBONUCLEOPROTEIN_COMPLEX_BIOGENESIS_AND_ASSEMBLY** | | **47** | **0.25075674** | **1.0202073** | **0.39051095** | **0.83585835** | **1** | **1310** | **tags=19%, list=10%, signal=21%** |
| **MICROTUBULE_POLYMERIZATION_OR_DEPOLYMERIZATION** | **MICROTUBULE_POLYMERIZATION_OR_DEPOLYMERIZATION** | | **11** | **0.3710311** | **1.0200031** | **0.41359773** | **0.82961076** | **1** | **2288** | **tags=36%, list=17%, signal=44%** |
| **FOCAL_ADHESION_FORMATION** | **FOCAL_ADHESION_FORMATION** | | **10** | **0.36900476** | **1.0183076** | **0.4273743** | **0.8277529** | **1** | **4253** | **tags=60%, list=32%, signal=88%** |
| **REGULATION_OF_NEURON_APOPTOSIS** | **REGULATION_OF_NEURON_APOPTOSIS** | | **10** | **0.38171145** | **1.0175289** | **0.41253263** | **0.8234342** | **1** | **2427** | **tags=30%, list=18%, signal=37%** |
| **NITROGEN_COMPOUND_BIOSYNTHETIC_PROCESS** | **NITROGEN_COMPOUND_BIOSYNTHETIC_PROCESS** | | **25** | **0.29023665** | **1.015343** | **0.39933994** | **0.82347465** | **1** | **2976** | **tags=40%, list=22%, signal=51%** |
| **POSITIVE_REGULATION_OF_PROTEIN_MODIFICATION_PROCESS** | **POSITIVE_REGULATION_OF_PROTEIN_MODIFICATION_PROCESS** | | **23** | **0.2977819** | **1.0126344** | **0.40184048** | **0.82529956** | **1** | **2625** | **tags=30%, list=20%, signal=38%** |
| **REGULATION_OF_PROTEIN_POLYMERIZATION** | **REGULATION_OF_PROTEIN_POLYMERIZATION** | | **10** | **0.38210896** | **1.009935** | **0.41919193** | **0.8271803** | **1** | **789** | **tags=30%, list=6%, signal=32%** |
| **GENERATION_OF_PRECURSOR_METABOLITES_AND_ENERGY** | **GENERATION_OF_PRECURSOR_METABOLITES_AND_ENERGY** | | **118** | **0.20687334** | **1.0065618** | **0.4** | **0.8301124** | **1** | **2080** | **tags=22%, list=16%, signal=26%** |
| **CELLULAR_LOCALIZATION** | **CELLULAR_LOCALIZATION** | | **310** | **0.18165967** | **1.0041157** | **0.42696628** | **0.8307839** | **1** | **1729** | **tags=17%, list=13%, signal=20%** |
| **MEMBRANE_FUSION** | **MEMBRANE_FUSION** | | **26** | **0.2794496** | **1.0031042** | **0.43225807** | **0.8273963** | **1** | **2511** | **tags=31%, list=19%, signal=38%** |
| **REGULATION_OF_CYTOSKELETON_ORGANIZATION_AND_BIOGENESIS** | **REGULATION_OF_CYTOSKELETON_ORGANIZATION_AND_BIOGENESIS** | | **25** | **0.2880298** | **1.0019372** | **0.4397394** | **0.82446283** | **1** | **1247** | **tags=20%, list=9%, signal=22%** |
| **BIOPOLYMER_CATABOLIC_PROCESS** | **BIOPOLYMER_CATABOLIC_PROCESS** | | **101** | **0.20953812** | **1.0011206** | **0.4170616** | **0.82072234** | **1** | **3320** | **tags=37%, list=25%, signal=48%** |
| **GLUCOSE_METABOLIC_PROCESS** | **GLUCOSE_METABOLIC_PROCESS** | | **26** | **0.28846037** | **1.0003841** | **0.42857143** | **0.81673354** | **1** | **2193** | **tags=27%, list=16%, signal=32%** |
| **DOUBLE_STRAND_BREAK_REPAIR** | **DOUBLE_STRAND_BREAK_REPAIR** | | **21** | **0.3002051** | **0.99803776** | **0.41520467** | **0.8172476** | **1** | **3918** | **tags=48%, list=29%, signal=67%** |
| **CYTOSKELETON_ORGANIZATION_AND_BIOGENESIS** | **CYTOSKELETON_ORGANIZATION_AND_BIOGENESIS** | | **177** | **0.19294241** | **0.9977512** | **0.4861111** | **0.81187457** | **1** | **2427** | **tags=23%, list=18%, signal=27%** |
| **CELL_DIVISION** | **CELL_DIVISION** | | **18** | **0.30311668** | **0.99445343** | **0.43712574** | **0.8151512** | **1** | **2073** | **tags=22%, list=16%, signal=26%** |
| **CYTOSKELETON_DEPENDENT_INTRACELLULAR_TRANSPORT** | **CYTOSKELETON_DEPENDENT_INTRACELLULAR_TRANSPORT** | | **26** | **0.27555966** | **0.99318194** | **0.443038** | **0.8127446** | **1** | **3153** | **tags=35%, list=24%, signal=45%** |
| **PHOSPHOLIPID_BIOSYNTHETIC_PROCESS** | **PHOSPHOLIPID_BIOSYNTHETIC_PROCESS** | | **30** | **0.26694506** | **0.9911276** | **0.47682118** | **0.8127456** | **1** | **2093** | **tags=27%, list=16%, signal=32%** |
| **OXYGEN_AND_REACTIVE_OXYGEN_SPECIES_METABOLIC_PROCESS** | **OXYGEN_AND_REACTIVE_OXYGEN_SPECIES_METABOLIC_PROCESS** | | **18** | **0.3008419** | **0.9836518** | **0.45721924** | **0.8279669** | **1** | **2427** | **tags=44%, list=18%, signal=54%** |
| **PROTEIN_MODIFICATION_BY_SMALL_PROTEIN_CONJUGATION** | **PROTEIN_MODIFICATION_BY_SMALL_PROTEIN_CONJUGATION** | | **36** | **0.2517384** | **0.98312557** | **0.47697368** | **0.8233476** | **1** | **2625** | **tags=31%, list=20%, signal=38%** |
| **MAINTENANCE_OF_CELLULAR_PROTEIN_LOCALIZATION** | **MAINTENANCE_OF_CELLULAR_PROTEIN_LOCALIZATION** | | **9** | **0.37445834** | **0.9775787** | **0.4326425** | **0.8335281** | **1** | **3274** | **tags=56%, list=25%, signal=74%** |
| **AMINO_ACID_DERIVATIVE_METABOLIC_PROCESS** | **AMINO_ACID_DERIVATIVE_METABOLIC_PROCESS** | | **23** | **0.2795738** | **0.9734352** | **0.47682118** | **0.8397491** | **1** | **3282** | **tags=39%, list=25%, signal=52%** |
| **REGULATION_OF_RHO_GTPASE_ACTIVITY** | **REGULATION_OF_RHO_GTPASE_ACTIVITY** | | **5** | **0.45148695** | **0.9711133** | **0.49311927** | **0.84027064** | **1** | **7308** | **tags=100%, list=55%, signal=221%** |
| **LIPID_BIOSYNTHETIC_PROCESS** | **LIPID_BIOSYNTHETIC_PROCESS** | | **78** | **0.21311642** | **0.9691751** | **0.48497853** | **0.84016323** | **1** | **1601** | **tags=19%, list=12%, signal=22%** |
| **TRICARBOXYLIC_ACID_CYCLE_INTERMEDIATE_METABOLIC_PROCESS** | **TRICARBOXYLIC_ACID_CYCLE_INTERMEDIATE_METABOLIC_PROCESS** | | **10** | **0.34760734** | **0.96659696** | **0.4735376** | **0.8413283** | **1** | **8693** | **tags=100%, list=65%, signal=288%** |
| **CELLULAR_PROTEIN_CATABOLIC_PROCESS** | **CELLULAR_PROTEIN_CATABOLIC_PROCESS** | | **49** | **0.24228288** | **0.96638876** | **0.48375452** | **0.83601606** | **1** | **3302** | **tags=43%, list=25%, signal=57%** |
| **PROTEIN_UBIQUITINATION** | **PROTEIN_UBIQUITINATION** | | **34** | **0.25672787** | **0.9655436** | **0.49103943** | **0.8329655** | **1** | **4186** | **tags=50%, list=31%, signal=73%** |
| **DNA_FRAGMENTATION_DURING_APOPTOSIS** | **DNA_FRAGMENTATION_DURING_APOPTOSIS** | | **11** | **0.3419113** | **0.963923** | **0.48541114** | **0.8318814** | **1** | **3320** | **tags=64%, list=25%, signal=85%** |
| **PROTEIN_MODIFICATION_PROCESS** | **PROTEIN_MODIFICATION_PROCESS** | | **531** | **0.1651978** | **0.96168256** | **0.6216216** | **0.83223236** | **1** | **2630** | **tags=25%, list=20%, signal=30%** |
| **ACTIN_FILAMENT_ORGANIZATION** | **ACTIN_FILAMENT_ORGANIZATION** | | **20** | **0.2848881** | **0.96085906** | **0.48474577** | **0.828843** | **1** | **2967** | **tags=35%, list=22%, signal=45%** |
| **SULFUR_METABOLIC_PROCESS** | **SULFUR_METABOLIC_PROCESS** | | **30** | **0.2642969** | **0.95742935** | **0.46461537** | **0.8326165** | **1** | **3080** | **tags=43%, list=23%, signal=56%** |
| **NEGATIVE_REGULATION_OF_DEVELOPMENTAL_PROCESS** | **NEGATIVE_REGULATION_OF_DEVELOPMENTAL_PROCESS** | | **169** | **0.18571223** | **0.95572317** | **0.6083916** | **0.8318982** | **1** | **2541** | **tags=27%, list=19%, signal=32%** |
| **BIOSYNTHETIC_PROCESS** | **BIOSYNTHETIC_PROCESS** | | **379** | **0.1681298** | **0.9555963** | **0.7105263** | **0.8267256** | **1** | **2005** | **tags=17%, list=15%, signal=20%** |
| **MACROMOLECULE_LOCALIZATION** | **MACROMOLECULE_LOCALIZATION** | | **190** | **0.18004** | **0.95492166** | **0.5968992** | **0.8233484** | **1** | **2554** | **tags=24%, list=19%, signal=29%** |
| **APOPTOTIC_PROGRAM** | **APOPTOTIC_PROGRAM** | | **53** | **0.22639968** | **0.9523665** | **0.4982079** | **0.82485753** | **1** | **3320** | **tags=47%, list=25%, signal=63%** |
| **CELLULAR_COMPONENT_DISASSEMBLY** | **CELLULAR_COMPONENT_DISASSEMBLY** | | **30** | **0.26280397** | **0.9513106** | **0.5236593** | **0.8224191** | **1** | **3320** | **tags=47%, list=25%, signal=62%** |
| **MICROTUBULE_BASED_MOVEMENT** | **MICROTUBULE_BASED_MOVEMENT** | | **16** | **0.30636418** | **0.95030135** | **0.53869045** | **0.81990635** | **1** | **29** | **tags=6%, list=0%, signal=6%** |
| **HISTONE_MODIFICATION** | **HISTONE_MODIFICATION** | | **18** | **0.30362213** | **0.9470343** | **0.5264706** | **0.8231706** | **1** | **1212** | **tags=28%, list=9%, signal=31%** |
| **AMINO_ACID_DERIVATIVE_BIOSYNTHETIC_PROCESS** | **AMINO_ACID_DERIVATIVE_BIOSYNTHETIC_PROCESS** | | **10** | **0.35431525** | **0.94065315** | **0.5294118** | **0.8349255** | **1** | **589** | **tags=20%, list=4%, signal=21%** |
| **MAINTENANCE_OF_PROTEIN_LOCALIZATION** | **MAINTENANCE_OF_PROTEIN_LOCALIZATION** | | **10** | **0.35084757** | **0.9387686** | **0.52645504** | **0.834608** | **1** | **3875** | **tags=60%, list=29%, signal=85%** |
| **POST_TRANSLATIONAL_PROTEIN_MODIFICATION** | **POST_TRANSLATIONAL_PROTEIN_MODIFICATION** | | **399** | **0.1587678** | **0.93858784** | **0.8363636** | **0.829885** | **1** | **2655** | **tags=24%, list=20%, signal=29%** |
| **NUCLEAR_TRANSPORT** | **NUCLEAR_TRANSPORT** | | **72** | **0.20919342** | **0.93488383** | **0.5656109** | **0.8345266** | **1** | **2491** | **tags=25%, list=19%, signal=31%** |
| **PROTEIN_AMINO_ACID_N_LINKED_GLYCOSYLATION** | **PROTEIN_AMINO_ACID_N_LINKED_GLYCOSYLATION** | | **26** | **0.26887783** | **0.9347788** | **0.55417955** | **0.8297006** | **1** | **2177** | **tags=35%, list=16%, signal=41%** |
| **N_ACETYLGLUCOSAMINE_METABOLIC_PROCESS** | **N_ACETYLGLUCOSAMINE_METABOLIC_PROCESS** | | **11** | **0.34000063** | **0.9346038** | **0.53435117** | **0.8250738** | **1** | **2658** | **tags=45%, list=20%, signal=57%** |
| **BIOPOLYMER_MODIFICATION** | **BIOPOLYMER_MODIFICATION** | | **546** | **0.1618909** | **0.93284273** | **0.74285716** | **0.8244644** | **1** | **2630** | **tags=24%, list=20%, signal=29%** |
| **TRANSCRIPTION_INITIATION** | **TRANSCRIPTION_INITIATION** | | **24** | **0.26282382** | **0.9312976** | **0.507837** | **0.82352626** | **1** | **1635** | **tags=21%, list=12%, signal=24%** |
| **CHROMATIN_MODIFICATION** | **CHROMATIN_MODIFICATION** | | **41** | **0.22998115** | **0.92888254** | **0.5951557** | **0.82476795** | **1** | **1642** | **tags=22%, list=12%, signal=25%** |
| **COVALENT_CHROMATIN_MODIFICATION** | **COVALENT_CHROMATIN_MODIFICATION** | | **19** | **0.28490856** | **0.9269871** | **0.5360231** | **0.82497555** | **1** | **1212** | **tags=26%, list=9%, signal=29%** |
| **NUCLEOSOME_ASSEMBLY** | **NUCLEOSOME_ASSEMBLY** | | **10** | **0.34791866** | **0.9262211** | **0.539726** | **0.8218607** | **1** | **3024** | **tags=50%, list=23%, signal=65%** |
| **MACROMOLECULE_CATABOLIC_PROCESS** | **MACROMOLECULE_CATABOLIC_PROCESS** | | **118** | **0.19274567** | **0.9209916** | **0.61142856** | **0.8304435** | **1** | **3320** | **tags=35%, list=25%, signal=46%** |
| **INTRACELLULAR_PROTEIN_TRANSPORT** | **INTRACELLULAR_PROTEIN_TRANSPORT** | | **122** | **0.18651763** | **0.9199968** | **0.6368715** | **0.82799846** | **1** | **2554** | **tags=25%, list=19%, signal=30%** |
| **SMALL_GTPASE_MEDIATED_SIGNAL_TRANSDUCTION** | **SMALL_GTPASE_MEDIATED_SIGNAL_TRANSDUCTION** | | **73** | **0.20357566** | **0.90866613** | **0.68899524** | **0.8519881** | **1** | **1515** | **tags=18%, list=11%, signal=20%** |
| **NUCLEOCYTOPLASMIC_TRANSPORT** | **NUCLEOCYTOPLASMIC_TRANSPORT** | | **72** | **0.20919342** | **0.9075049** | **0.5813953** | **0.85011214** | **1** | **2491** | **tags=25%, list=19%, signal=31%** |
| **CHROMATIN_REMODELING** | **CHROMATIN_REMODELING** | | **19** | **0.28057712** | **0.9047668** | **0.5855856** | **0.85162306** | **1** | **3024** | **tags=37%, list=23%, signal=48%** |
| **MAINTENANCE_OF_LOCALIZATION** | **MAINTENANCE_OF_LOCALIZATION** | | **19** | **0.28149882** | **0.90382385** | **0.5903614** | **0.84907657** | **1** | **3274** | **tags=37%, list=25%, signal=49%** |
| **POLYSACCHARIDE_METABOLIC_PROCESS** | **POLYSACCHARIDE_METABOLIC_PROCESS** | | **13** | **0.30957207** | **0.90285057** | **0.58126724** | **0.84661645** | **1** | **1037** | **tags=23%, list=8%, signal=25%** |
| **ORGANELLE_ORGANIZATION_AND_BIOGENESIS** | **ORGANELLE_ORGANIZATION_AND_BIOGENESIS** | | **386** | **0.15686123** | **0.8950784** | **0.8518519** | **0.8602569** | **1** | **2641** | **tags=24%, list=20%, signal=29%** |
| **NUCLEAR_IMPORT** | **NUCLEAR_IMPORT** | | **43** | **0.22645605** | **0.8950002** | **0.65637064** | **0.85555416** | **1** | **1729** | **tags=21%, list=13%, signal=24%** |
| **RAS_PROTEIN_SIGNAL_TRANSDUCTION** | **RAS_PROTEIN_SIGNAL_TRANSDUCTION** | | **54** | **0.2150656** | **0.8938389** | **0.6535433** | **0.8535291** | **1** | **1476** | **tags=19%, list=11%, signal=21%** |
| **PROTEIN_LOCALIZATION** | **PROTEIN_LOCALIZATION** | | **174** | **0.16849242** | **0.8906464** | **0.796748** | **0.8564313** | **1** | **2554** | **tags=23%, list=19%, signal=28%** |
| **NEURON_APOPTOSIS** | **NEURON_APOPTOSIS** | | **14** | **0.29552495** | **0.881354** | **0.625** | **0.87359387** | **1** | **2427** | **tags=36%, list=18%, signal=44%** |
| **REGULATION_OF_RAS_GTPASE_ACTIVITY** | **REGULATION_OF_RAS_GTPASE_ACTIVITY** | | **6** | **0.3879835** | **0.8797961** | **0.5690073** | **0.8721072** | **1** | **8154** | **tags=100%, list=61%, signal=258%** |
| **PROTEIN_AMINO_ACID_ADP_RIBOSYLATION** | **PROTEIN_AMINO_ACID_ADP_RIBOSYLATION** | | **10** | **0.32181838** | **0.8790283** | **0.6025641** | **0.86914825** | **1** | **952** | **tags=20%, list=7%, signal=22%** |
| **RESPONSE_TO_CARBOHYDRATE_STIMULUS** | **RESPONSE_TO_CARBOHYDRATE_STIMULUS** | | **10** | **0.3205422** | **0.8731963** | **0.6011396** | **0.8774307** | **1** | **2104** | **tags=40%, list=16%, signal=47%** |
| **ESTABLISHMENT_OF_PROTEIN_LOCALIZATION** | **ESTABLISHMENT_OF_PROTEIN_LOCALIZATION** | | **156** | **0.17137666** | **0.8715136** | **0.8113208** | **0.87678635** | **1** | **2554** | **tags=23%, list=19%, signal=28%** |
| **BIOGENIC_AMINE_METABOLIC_PROCESS** | **BIOGENIC_AMINE_METABOLIC_PROCESS** | | **16** | **0.28316033** | **0.86926854** | **0.61461794** | **0.87705517** | **1** | **430** | **tags=13%, list=3%, signal=13%** |
| **PROTEIN_CATABOLIC_PROCESS** | **PROTEIN_CATABOLIC_PROCESS** | | **58** | **0.19773524** | **0.8626124** | **0.7180451** | **0.88700664** | **1** | **3302** | **tags=38%, list=25%, signal=50%** |
| **MAINTENANCE_OF_CELLULAR_LOCALIZATION** | **MAINTENANCE_OF_CELLULAR_LOCALIZATION** | | **10** | **0.313987** | **0.8596546** | **0.6246575** | **0.8887023** | **1** | **3274** | **tags=50%, list=25%, signal=66%** |
| **EPIDERMAL_GROWTH_FACTOR_RECEPTOR_SIGNALING_PATHWAY** | **EPIDERMAL_GROWTH_FACTOR_RECEPTOR_SIGNALING_PATHWAY** | | **18** | **0.267935** | **0.85902965** | **0.64927536** | **0.8854014** | **1** | **1585** | **tags=28%, list=12%, signal=31%** |
| **REGULATION_OF_AXONOGENESIS** | **REGULATION_OF_AXONOGENESIS** | | **8** | **0.35239366** | **0.8581109** | **0.6308901** | **0.88282883** | **1** | **916** | **tags=25%, list=7%, signal=27%** |
| **CELLULAR_MACROMOLECULE_CATABOLIC_PROCESS** | **CELLULAR_MACROMOLECULE_CATABOLIC_PROCESS** | | **90** | **0.18047598** | **0.8578767** | **0.8063063** | **0.8785895** | **1** | **2833** | **tags=30%, list=21%, signal=38%** |
| **REGIONALIZATION** | **REGIONALIZATION** | | **12** | **0.30187425** | **0.8551267** | **0.6350975** | **0.8802113** | **1** | **1735** | **tags=25%, list=13%, signal=29%** |
| **REGULATION_OF_PROTEIN_MODIFICATION_PROCESS** | **REGULATION_OF_PROTEIN_MODIFICATION_PROCESS** | | **34** | **0.22562693** | **0.85463965** | **0.722973** | **0.8766005** | **1** | **2625** | **tags=24%, list=20%, signal=29%** |
| **MACROMOLECULAR_COMPLEX_ASSEMBLY** | **MACROMOLECULAR_COMPLEX_ASSEMBLY** | | **219** | **0.158896** | **0.85345083** | **0.9137931** | **0.87456346** | **1** | **1759** | **tags=17%, list=13%, signal=19%** |
| **PROTEIN_SECRETION** | **PROTEIN_SECRETION** | | **22** | **0.24883533** | **0.8528717** | **0.69005847** | **0.8712489** | **1** | **925** | **tags=14%, list=7%, signal=15%** |
| **INTERLEUKIN_8_BIOSYNTHETIC_PROCESS** | **INTERLEUKIN_8_BIOSYNTHETIC_PROCESS** | | **7** | **0.34218177** | **0.84154564** | **0.64444447** | **0.890243** | **1** | **64** | **tags=14%, list=0%, signal=14%** |
| **FATTY_ACID_BETA_OXIDATION** | **FATTY_ACID_BETA_OXIDATION** | | **10** | **0.31214175** | **0.8356687** | **0.6566416** | **0.8972328** | **1** | **1251** | **tags=30%, list=9%, signal=33%** |
| **POSITIVE_REGULATION_OF_DNA_METABOLIC_PROCESS** | **POSITIVE_REGULATION_OF_DNA_METABOLIC_PROCESS** | | **10** | **0.31187618** | **0.82979023** | **0.707124** | **0.9039078** | **1** | **527** | **tags=20%, list=4%, signal=21%** |
| **RESPIRATORY_GASEOUS_EXCHANGE** | **RESPIRATORY_GASEOUS_EXCHANGE** | | **14** | **0.26954702** | **0.8254896** | **0.72753626** | **0.9072941** | **1** | **2149** | **tags=29%, list=16%, signal=34%** |
| **GLUCOSAMINE_METABOLIC_PROCESS** | **GLUCOSAMINE_METABOLIC_PROCESS** | | **12** | **0.2919085** | **0.82318825** | **0.68221575** | **0.90696037** | **1** | **1037** | **tags=25%, list=8%, signal=27%** |
| **POSITIVE_REGULATION_OF_CELLULAR_COMPONENT_ORGANIZATION_AND_BIOGENESIS** | **POSITIVE_REGULATION_OF_CELLULAR_COMPONENT_ORGANIZATION_AND_BIOGENESIS** | | **29** | **0.22200435** | **0.8218003** | **0.72789115** | **0.9051564** | **1** | **1076** | **tags=17%, list=8%, signal=19%** |
| **APOPTOTIC_MITOCHONDRIAL_CHANGES** | **APOPTOTIC_MITOCHONDRIAL_CHANGES** | | **10** | **0.30205294** | **0.81973714** | **0.6584022** | **0.904613** | **1** | **2864** | **tags=40%, list=21%, signal=51%** |
| **POSITIVE_REGULATION_OF_NUCLEOBASENUCLEOSIDENUCLEOTIDE_AND_NUCLEIC_ACID_METABOLIC_PROCESS** | **POSITIVE_REGULATION_OF_NUCLEOBASENUCLEOSIDENUCLEOTIDE_AND_NUCLEIC_ACID_METABOLIC_PROCESS** | | **118** | **0.1689833** | **0.8098731** | **0.9100529** | **0.9180391** | **1** | **3371** | **tags=36%, list=25%, signal=47%** |
| **PHOSPHORYLATION** | **PHOSPHORYLATION** | | **254** | **0.15278575** | **0.80897975** | **0.97321427** | **0.91520643** | **1** | **2655** | **tags=23%, list=20%, signal=28%** |
| **CELLULAR_RESPONSE_TO_STIMULUS** | **CELLULAR_RESPONSE_TO_STIMULUS** | | **15** | **0.26300243** | **0.8087837** | **0.7057143** | **0.91103244** | **1** | **511** | **tags=13%, list=4%, signal=14%** |
| **ORGANELLE_LOCALIZATION** | **ORGANELLE_LOCALIZATION** | | **21** | **0.239671** | **0.7933414** | **0.7584098** | **0.93387383** | **1** | **2406** | **tags=29%, list=18%, signal=35%** |
| **PYRIMIDINE_NUCLEOTIDE_METABOLIC_PROCESS** | **PYRIMIDINE_NUCLEOTIDE_METABOLIC_PROCESS** | | **8** | **0.30882266** | **0.7690022** | **0.7153846** | **0.9691956** | **1** | **205** | **tags=13%, list=2%, signal=13%** |
| **CELLULAR_COMPONENT_ASSEMBLY** | **CELLULAR_COMPONENT_ASSEMBLY** | | **236** | **0.1450836** | **0.7689792** | **1** | **0.9645389** | **1** | **2166** | **tags=19%, list=16%, signal=22%** |
| **SECONDARY_METABOLIC_PROCESS** | **SECONDARY_METABOLIC_PROCESS** | | **23** | **0.21868151** | **0.7650693** | **0.8078078** | **0.9658946** | **1** | **580** | **tags=9%, list=4%, signal=9%** |
| **NEGATIVE_REGULATION_OF_INTRACELLULAR_TRANSPORT** | **NEGATIVE_REGULATION_OF_INTRACELLULAR_TRANSPORT** | | **9** | **0.29184192** | **0.76133263** | **0.7534626** | **0.96707875** | **1** | **9435** | **tags=100%, list=71%, signal=343%** |
| **POSITIVE_REGULATION_OF_RNA_METABOLIC_PROCESS** | **POSITIVE_REGULATION_OF_RNA_METABOLIC_PROCESS** | | **95** | **0.1618266** | **0.7549703** | **0.92626727** | **0.9720607** | **1** | **3437** | **tags=36%, list=26%, signal=48%** |
| **RESPONSE_TO_IONIZING_RADIATION** | **RESPONSE_TO_IONIZING_RADIATION** | | **7** | **0.32117602** | **0.754859** | **0.7772277** | **0.96762586** | **1** | **2930** | **tags=57%, list=22%, signal=73%** |
| **INORGANIC_ANION_TRANSPORT** | **INORGANIC_ANION_TRANSPORT** | | **14** | **0.24995199** | **0.75260556** | **0.808** | **0.9664709** | **1** | **1226** | **tags=21%, list=9%, signal=24%** |
| **LYSOSOMAL_TRANSPORT** | **LYSOSOMAL_TRANSPORT** | | **8** | **0.30319682** | **0.7523666** | **0.72527474** | **0.96227866** | **1** | **478** | **tags=13%, list=4%, signal=13%** |
| **ER_NUCLEAR_SIGNALING_PATHWAY** | **ER_NUCLEAR_SIGNALING_PATHWAY** | | **13** | **0.25741845** | **0.7488375** | **0.79479766** | **0.9629444** | **1** | **3670** | **tags=46%, list=28%, signal=64%** |
| **VITAMIN_METABOLIC_PROCESS** | **VITAMIN_METABOLIC_PROCESS** | | **15** | **0.24276175** | **0.74424213** | **0.84823847** | **0.9649407** | **1** | **71** | **tags=7%, list=1%, signal=7%** |
| **TRNA_PROCESSING** | **TRNA_PROCESSING** | | **8** | **0.2922491** | **0.73640716** | **0.8034826** | **0.97134805** | **1** | **1203** | **tags=25%, list=9%, signal=27%** |
| **NEGATIVE_REGULATION_OF_SIGNAL_TRANSDUCTION** | **NEGATIVE_REGULATION_OF_SIGNAL_TRANSDUCTION** | | **30** | **0.19569434** | **0.7290671** | **0.8833333** | **0.97649825** | **1** | **712** | **tags=10%, list=5%, signal=11%** |
| **INDUCTION_OF_APOPTOSIS_BY_INTRACELLULAR_SIGNALS** | **INDUCTION_OF_APOPTOSIS_BY_INTRACELLULAR_SIGNALS** | | **21** | **0.21815687** | **0.72783256** | **0.8445748** | **0.97364813** | **1** | **360** | **tags=10%, list=3%, signal=10%** |
| **GLUCAN_METABOLIC_PROCESS** | **GLUCAN_METABOLIC_PROCESS** | | **9** | **0.2751019** | **0.71408623** | **0.8372703** | **0.98639727** | **1** | **274** | **tags=11%, list=2%, signal=11%** |
| **REGULATION_OF_TRANSFORMING_GROWTH_FACTOR_BETA_RECEPTOR_SIGNALING_PATHWAY** | **REGULATION_OF_TRANSFORMING_GROWTH_FACTOR_BETA_RECEPTOR_SIGNALING_PATHWAY** | | **12** | **0.24971236** | **0.7034264** | **0.87784094** | **0.9940159** | **1** | **4014** | **tags=50%, list=30%, signal=71%** |
| **HEME_BIOSYNTHETIC_PROCESS** | **HEME_BIOSYNTHETIC_PROCESS** | | **10** | **0.25877443** | **0.69705105** | **0.8473054** | **0.99637705** | **1** | **580** | **tags=10%, list=4%, signal=10%** |
| **CELLULAR_PROTEIN_COMPLEX_DISASSEMBLY** | **CELLULAR_PROTEIN_COMPLEX_DISASSEMBLY** | | **13** | **0.23820956** | **0.687471** | **0.8847185** | **1** | **1** | **2195** | **tags=23%, list=16%, signal=28%** |
| **TRANSLATIONAL_INITIATION** | **TRANSLATIONAL_INITIATION** | | **22** | **0.20146498** | **0.68552023** | **0.9245283** | **0.99946934** | **1** | **511** | **tags=9%, list=4%, signal=9%** |
| **VACUOLAR_TRANSPORT** | **VACUOLAR_TRANSPORT** | | **9** | **0.2606471** | **0.67276245** | **0.86197186** | **1** | **1** | **478** | **tags=11%, list=4%, signal=12%** |
| **REGULATION_OF_TRANSLATIONAL_INITIATION** | **REGULATION_OF_TRANSLATIONAL_INITIATION** | | **15** | **0.22029467** | **0.66940475** | **0.8981723** | **1** | **1** | **3794** | **tags=40%, list=28%, signal=56%** |
| **ACTIVATION_OF_NF_KAPPAB_TRANSCRIPTION_FACTOR** | **ACTIVATION_OF_NF_KAPPAB_TRANSCRIPTION_FACTOR** | | **14** | **0.2266599** | **0.6680843** | **0.91930836** | **1** | **1** | **672** | **tags=14%, list=5%, signal=15%** |
| **AMINE_BIOSYNTHETIC_PROCESS** | **AMINE_BIOSYNTHETIC_PROCESS** | | **15** | **0.21508293** | **0.66145337** | **0.9101124** | **1** | **1** | **2976** | **tags=33%, list=22%, signal=43%** |
| **MYOBLAST_DIFFERENTIATION** | **MYOBLAST_DIFFERENTIATION** | | **16** | **0.21085858** | **0.65510255** | **0.90936553** | **1** | **1** | **2697** | **tags=25%, list=20%, signal=31%** |
| **PHAGOCYTOSIS** | **PHAGOCYTOSIS** | | **16** | **0.20957756** | **0.6410845** | **0.90434784** | **1** | **1** | **1206** | **tags=13%, list=9%, signal=14%** |
| **MITOTIC_CELL_CYCLE_CHECKPOINT** | **MITOTIC_CELL_CYCLE_CHECKPOINT** | | **19** | **0.1984663** | **0.64027184** | **0.9147059** | **1** | **1** | **10680** | **tags=100%, list=80%, signal=504%** |
| **MACROMOLECULAR_COMPLEX_DISASSEMBLY** | **MACROMOLECULAR_COMPLEX_DISASSEMBLY** | | **14** | **0.21028413** | **0.6293332** | **0.915942** | **1** | **1** | **1068** | **tags=14%, list=8%, signal=16%** |
| **PROTEIN_COMPLEX_DISASSEMBLY** | **PROTEIN_COMPLEX_DISASSEMBLY** | | **14** | **0.21028413** | **0.6275235** | **0.92777777** | **1** | **1** | **1068** | **tags=14%, list=8%, signal=16%** |
| **POSITIVE_REGULATION_OF_BINDING** | **POSITIVE_REGULATION_OF_BINDING** | | **18** | **0.19224492** | **0.62528247** | **0.937677** | **1** | **1** | **672** | **tags=11%, list=5%, signal=12%** |
| **DNA_REPLICATION_INITIATION** | **DNA_REPLICATION_INITIATION** | | **11** | **0.22024496** | **0.618734** | **0.92134833** | **1** | **1** | **2056** | **tags=27%, list=15%, signal=32%** |
| **RNA_ELONGATION** | **RNA_ELONGATION** | | **10** | **0.22645257** | **0.61483014** | **0.9111111** | **1** | **1** | **1367** | **tags=20%, list=10%, signal=22%** |
| **POSITIVE_REGULATION_OF_DNA_BINDING** | **POSITIVE_REGULATION_OF_DNA_BINDING** | | **18** | **0.19224492** | **0.6115101** | **0.9690141** | **1** | **1** | **672** | **tags=11%, list=5%, signal=12%** |
| **GLUCOSE_CATABOLIC_PROCESS** | **GLUCOSE_CATABOLIC_PROCESS** | | **11** | **0.2110874** | **0.6109066** | **0.9285714** | **0.9961288** | **1** | **2004** | **tags=18%, list=15%, signal=21%** |
| **CHROMOSOME_CONDENSATION** | **CHROMOSOME_CONDENSATION** | | **7** | **0.2516413** | **0.6080382** | **0.9510309** | **0.9936028** | **1** | **2528** | **tags=43%, list=19%, signal=53%** |
| **POSITIVE_REGULATION_OF_TRANSCRIPTION_FACTOR_ACTIVITY** | **POSITIVE_REGULATION_OF_TRANSCRIPTION_FACTOR_ACTIVITY** | | **16** | **0.20106992** | **0.60421425** | **0.94586897** | **0.9914835** | **1** | **672** | **tags=13%, list=5%, signal=13%** |
| **REGULATION_OF_CELL_CELL_ADHESION** | **REGULATION_OF_CELL_CELL_ADHESION** | | **9** | **0.23264992** | **0.6015729** | **0.9232804** | **0.98873967** | **1** | **1582** | **tags=22%, list=12%, signal=25%** |
| **NUCLEOBASENUCLEOSIDE_AND_NUCLEOTIDE_METABOLIC_PROCESS** | **NUCLEOBASENUCLEOSIDE_AND_NUCLEOTIDE_METABOLIC_PROCESS** | | **44** | **0.15056477** | **0.5925341** | **0.98571426** | **0.9890394** | **1** | **1124** | **tags=9%, list=8%, signal=10%** |
| **ENDOSOME_TRANSPORT** | **ENDOSOME_TRANSPORT** | | **22** | **0.16843185** | **0.5799452** | **0.95859873** | **0.9908081** | **1** | **3125** | **tags=27%, list=23%, signal=36%** |
| **HEME_METABOLIC_PROCESS** | **HEME_METABOLIC_PROCESS** | | **11** | **0.20660014** | **0.57805467** | **0.9611111** | **0.98762983** | **1** | **580** | **tags=9%, list=4%, signal=9%** |
| **REGULATION_OF_ENDOTHELIAL_CELL_PROLIFERATION** | **REGULATION_OF_ENDOTHELIAL_CELL_PROLIFERATION** | | **7** | **0.23944716** | **0.5688954** | **0.9457364** | **0.9870803** | **1** | **10132** | **tags=100%, list=76%, signal=417%** |
| **REGULATION_OF_JAK_STAT_CASCADE** | **REGULATION_OF_JAK_STAT_CASCADE** | | **7** | **0.22825597** | **0.55072963** | **0.9636871** | **0.98934114** | **1** | **10281** | **tags=100%, list=77%, signal=438%** |
| **DNA_DAMAGE_RESPONSESIGNAL_TRANSDUCTION_RESULTING_IN_INDUCTION_OF_APOPTOSIS** | **DNA_DAMAGE_RESPONSESIGNAL_TRANSDUCTION_RESULTING_IN_INDUCTION_OF_APOPTOSIS** | | **13** | **0.17555928** | **0.5107946** | **0.98** | **0.99499273** | **1** | **274** | **tags=8%, list=2%, signal=8%** |
| **REGULATION_OF_TYROSINE_PHOSPHORYLATION_OF_STAT_PROTEIN** | **REGULATION_OF_TYROSINE_PHOSPHORYLATION_OF_STAT_PROTEIN** | | **6** | **0.22823891** | **0.5096363** | **0.975** | **0.9911828** | **1** | **10281** | **tags=100%, list=77%, signal=438%** |

**Table S2.2.- GSE10072: Never smokers, normal tissue, enriched in women**

| **NAME** | **GS<br> follow link to MSigDB** | **GS DETAILS** | **SIZE** | **ES** | **NES** | **NOM p-val** | **FDR q-val** | **FWER p-val** | **RANK AT MAX** | **LEADING EDGE** |
| --- | --- | --- | --- | --- | --- | --- | --- | --- | --- | --- |
| **G_PROTEIN_COUPLED_RECEPTOR_PROTEIN_SIGNALING_PATHWAY** | **G_PROTEIN_COUPLED_RECEPTOR_PROTEIN_SIGNALING_PATHWAY** | **Details ...** | **290** | **-0.42472085** | **-1.9437582** | **0** | **0.15535158** | **0.144** | **3721** | **tags=47%, list=28%, signal=63%** |
| **SENSORY_PERCEPTION** | **SENSORY_PERCEPTION** | **Details ...** | **161** | **-0.4347215** | **-1.8975408** | **0** | **0.14368454** | **0.247** | **4566** | **tags=58%, list=34%, signal=88%** |
| **MONOVALENT_INORGANIC_CATION_TRANSPORT** | **MONOVALENT_INORGANIC_CATION_TRANSPORT** | **Details ...** | **78** | **-0.4824819** | **-1.8968755** | **0** | **0.09645083** | **0.248** | **3600** | **tags=50%, list=27%, signal=68%** |
| **NEUROLOGICAL_SYSTEM_PROCESS** | **NEUROLOGICAL_SYSTEM_PROCESS** | **Details ...** | **324** | **-0.40879884** | **-1.8875083** | **0** | **0.0845698** | **0.29** | **4566** | **tags=55%, list=34%, signal=81%** |
| **DETECTION_OF_STIMULUS** | **DETECTION_OF_STIMULUS** | **Details ...** | **33** | **-0.55719143** | **-1.8667177** | **0** | **0.08867265** | **0.356** | **5193** | **tags=82%, list=39%, signal=134%** |
| **REGULATION_OF_T_CELL_ACTIVATION** | **REGULATION_OF_T_CELL_ACTIVATION** | **Details ...** | **26** | **-0.596079** | **-1.8635097** | **0.00297177** | **0.07846844** | **0.376** | **1937** | **tags=42%, list=15%, signal=49%** |
| **CELL_CELL_SIGNALING** | **CELL_CELL_SIGNALING** | **Details ...** | **366** | **-0.39850014** | **-1.8602321** | **0** | **0.06949831** | **0.382** | **3518** | **tags=41%, list=26%, signal=55%** |
| **SECOND_MESSENGER_MEDIATED_SIGNALING** | **SECOND_MESSENGER_MEDIATED_SIGNALING** | **Details ...** | **141** | **-0.43560594** | **-1.8550715** | **0** | **0.0639936** | **0.396** | **3687** | **tags=49%, list=28%, signal=67%** |
| **RESPONSE_TO_BACTERIUM** | **RESPONSE_TO_BACTERIUM** | **Details ...** | **19** | **-0.629679** | **-1.844894** | **0.0014881** | **0.06405734** | **0.433** | **2756** | **tags=58%, list=21%, signal=73%** |
| **POTASSIUM_ION_TRANSPORT** | **POTASSIUM_ION_TRANSPORT** | **Details ...** | **51** | **-0.5004298** | **-1.8429016** | **0** | **0.05931841** | **0.441** | **3600** | **tags=51%, list=27%, signal=70%** |
| **SYSTEM_PROCESS** | **SYSTEM_PROCESS** | **Details ...** | **492** | **-0.3800441** | **-1.8164271** | **0** | **0.07492767** | **0.559** | **3645** | **tags=42%, list=27%, signal=56%** |
| **G_PROTEIN_SIGNALING_COUPLED_TO_CYCLIC_NUCLEOTIDE_SECOND_MESSENGER** | **G_PROTEIN_SIGNALING_COUPLED_TO_CYCLIC_NUCLEOTIDE_SECOND_MESSENGER** | **Details ...** | **97** | **-0.43632683** | **-1.8035338** | **0** | **0.07954621** | **0.611** | **3687** | **tags=52%, list=28%, signal=71%** |
| **SYNAPTIC_TRANSMISSION** | **SYNAPTIC_TRANSMISSION** | **Details ...** | **152** | **-0.41909286** | **-1.8029126** | **0** | **0.07432749** | **0.617** | **3589** | **tags=45%, list=27%, signal=61%** |
| **DEFENSE_RESPONSE_TO_BACTERIUM** | **DEFENSE_RESPONSE_TO_BACTERIUM** | **Details ...** | **13** | **-0.6676567** | **-1.7821575** | **0** | **0.08652107** | **0.697** | **2756** | **tags=62%, list=21%, signal=78%** |
| **MULTI_ORGANISM_PROCESS** | **MULTI_ORGANISM_PROCESS** | **Details ...** | **130** | **-0.41991773** | **-1.7733767** | **0** | **0.09190136** | **0.747** | **2756** | **tags=35%, list=21%, signal=43%** |
| **METAL_ION_TRANSPORT** | **METAL_ION_TRANSPORT** | **Details ...** | **98** | **-0.43391803** | **-1.7730279** | **0** | **0.08646055** | **0.748** | **3600** | **tags=45%, list=27%, signal=61%** |
| **PHOSPHOINOSITIDE_MEDIATED_SIGNALING** | **PHOSPHOINOSITIDE_MEDIATED_SIGNALING** | **Details ...** | **41** | **-0.5024014** | **-1.7692648** | **0.00140449** | **0.08500879** | **0.761** | **2931** | **tags=44%, list=22%, signal=56%** |
| **CATION_TRANSPORT** | **CATION_TRANSPORT** | **Details ...** | **124** | **-0.4186433** | **-1.7546684** | **0.0012285** | **0.09569922** | **0.822** | **3600** | **tags=44%, list=27%, signal=59%** |
| **G_PROTEIN_SIGNALING_COUPLED_TO_IP3_SECOND_MESSENGERPHOSPHOLIPASE_C_ACTIVATING** | **G_PROTEIN_SIGNALING_COUPLED_TO_IP3_SECOND_MESSENGERPHOSPHOLIPASE_C_ACTIVATING** | **Details ...** | **38** | **-0.5160546** | **-1.7517534** | **0** | **0.0933416** | **0.83** | **2931** | **tags=45%, list=22%, signal=57%** |
| **G_PROTEIN_SIGNALING_COUPLED_TO_CAMP_NUCLEOTIDE_SECOND_MESSENGER** | **G_PROTEIN_SIGNALING_COUPLED_TO_CAMP_NUCLEOTIDE_SECOND_MESSENGER** | **Details ...** | **63** | **-0.45762548** | **-1.7463638** | **0.00135135** | **0.09292819** | **0.843** | **3687** | **tags=54%, list=28%, signal=74%** |
| **GENERATION_OF_A_SIGNAL_INVOLVED_IN_CELL_CELL_SIGNALING** | **GENERATION_OF_A_SIGNAL_INVOLVED_IN_CELL_CELL_SIGNALING** | **Details ...** | **24** | **-0.5521153** | **-1.725015** | **0.01004304** | **0.1095103** | **0.901** | **3473** | **tags=58%, list=26%, signal=79%** |
| **TRANSMISSION_OF_NERVE_IMPULSE** | **TRANSMISSION_OF_NERVE_IMPULSE** | **Details ...** | **165** | **-0.39220494** | **-1.7153177** | **0** | **0.11540601** | **0.928** | **3518** | **tags=42%, list=26%, signal=56%** |
| **IMMUNE_SYSTEM_DEVELOPMENT** | **IMMUNE_SYSTEM_DEVELOPMENT** | **Details ...** | **71** | **-0.44302753** | **-1.7130586** | **0** | **0.11296329** | **0.931** | **3201** | **tags=38%, list=24%, signal=50%** |
| **CAMP_MEDIATED_SIGNALING** | **CAMP_MEDIATED_SIGNALING** | **Details ...** | **64** | **-0.44498152** | **-1.7092271** | **0** | **0.11238307** | **0.938** | **3687** | **tags=53%, list=28%, signal=73%** |
| **REGULATION_OF_LYMPHOCYTE_ACTIVATION** | **REGULATION_OF_LYMPHOCYTE_ACTIVATION** | **Details ...** | **31** | **-0.5040159** | **-1.7079116** | **0.00851064** | **0.10882808** | **0.941** | **1937** | **tags=35%, list=15%, signal=41%** |
| **RESPONSE_TO_OTHER_ORGANISM** | **RESPONSE_TO_OTHER_ORGANISM** | **Details ...** | **64** | **-0.44943854** | **-1.7059491** | **0.00266312** | **0.10713203** | **0.946** | **3117** | **tags=39%, list=23%, signal=51%** |
| **ECTODERM_DEVELOPMENT** | **ECTODERM_DEVELOPMENT** | **Details ...** | **73** | **-0.4362132** | **-1.7025429** | **0.00131062** | **0.10668892** | **0.952** | **2272** | **tags=40%, list=17%, signal=48%** |
| **CYCLIC_NUCLEOTIDE_MEDIATED_SIGNALING** | **CYCLIC_NUCLEOTIDE_MEDIATED_SIGNALING** | **Details ...** | **99** | **-0.42308158** | **-1.6997876** | **0.00121655** | **0.10577472** | **0.959** | **3687** | **tags=51%, list=28%, signal=69%** |
| **REGULATION_OF_MULTICELLULAR_ORGANISMAL_PROCESS** | **REGULATION_OF_MULTICELLULAR_ORGANISMAL_PROCESS** | **Details ...** | **127** | **-0.40612945** | **-1.6940553** | **0.00124069** | **0.1085569** | **0.965** | **2772** | **tags=35%, list=21%, signal=43%** |
| **CYTOKINE_PRODUCTION** | **CYTOKINE_PRODUCTION** | **Details ...** | **55** | **-0.45245853** | **-1.6851566** | **0.00269542** | **0.11530359** | **0.975** | **3162** | **tags=51%, list=24%, signal=66%** |
| **LIPID_CATABOLIC_PROCESS** | **LIPID_CATABOLIC_PROCESS** | **Details ...** | **31** | **-0.51052505** | **-1.6841774** | **0.01154401** | **0.11266202** | **0.975** | **2104** | **tags=48%, list=16%, signal=57%** |
| **NEGATIVE_REGULATION_OF_SECRETION** | **NEGATIVE_REGULATION_OF_SECRETION** | **Details ...** | **9** | **-0.70239383** | **-1.6832951** | **0.01092044** | **0.11021047** | **0.977** | **3194** | **tags=78%, list=24%, signal=102%** |
| **DETECTION_OF_EXTERNAL_STIMULUS** | **DETECTION_OF_EXTERNAL_STIMULUS** | **Details ...** | **16** | **-0.6029246** | **-1.6756773** | **0.01217656** | **0.11536314** | **0.982** | **4850** | **tags=81%, list=36%, signal=128%** |
| **REGULATION_OF_IMMUNE_SYSTEM_PROCESS** | **REGULATION_OF_IMMUNE_SYSTEM_PROCESS** | **Details ...** | **55** | **-0.4474856** | **-1.6696321** | **0.00543478** | **0.11862037** | **0.989** | **2772** | **tags=36%, list=21%, signal=46%** |
| **REGULATION_OF_HORMONE_SECRETION** | **REGULATION_OF_HORMONE_SECRETION** | **Details ...** | **12** | **-0.63337344** | **-1.6686413** | **0.00944882** | **0.11604265** | **0.99** | **3194** | **tags=67%, list=24%, signal=88%** |
| **T_CELL_ACTIVATION** | **T_CELL_ACTIVATION** | **Details ...** | **39** | **-0.48482788** | **-1.6644487** | **0.00420168** | **0.11739676** | **0.99** | **2772** | **tags=38%, list=21%, signal=48%** |
| **CYTOKINE_METABOLIC_PROCESS** | **CYTOKINE_METABOLIC_PROCESS** | **Details ...** | **34** | **-0.49436504** | **-1.6593872** | **0.00842697** | **0.11989322** | **0.992** | **3459** | **tags=59%, list=26%, signal=79%** |
| **HEMOPOIETIC_OR_LYMPHOID_ORGAN_DEVELOPMENT** | **HEMOPOIETIC_OR_LYMPHOID_ORGAN_DEVELOPMENT** | **Details ...** | **68** | **-0.43058264** | **-1.6541325** | **0.00398936** | **0.12247166** | **0.994** | **3201** | **tags=37%, list=24%, signal=48%** |
| **CHEMICAL_HOMEOSTASIS** | **CHEMICAL_HOMEOSTASIS** | **Details ...** | **133** | **-0.39325175** | **-1.6530005** | **0** | **0.12073793** | **0.994** | **4805** | **tags=53%, list=36%, signal=82%** |
| **LYMPHOCYTE_ACTIVATION** | **LYMPHOCYTE_ACTIVATION** | **Details ...** | **53** | **-0.4483425** | **-1.6515247** | **0.0056101** | **0.11965143** | **0.994** | **3162** | **tags=40%, list=24%, signal=52%** |
| **LEUKOCYTE_DIFFERENTIATION** | **LEUKOCYTE_DIFFERENTIATION** | **Details ...** | **34** | **-0.48945227** | **-1.6486496** | **0.00983146** | **0.1197386** | **0.994** | **3201** | **tags=38%, list=24%, signal=50%** |
| **RESPONSE_TO_BIOTIC_STIMULUS** | **RESPONSE_TO_BIOTIC_STIMULUS** | **Details ...** | **97** | **-0.4112538** | **-1.6458721** | **0** | **0.12015209** | **0.994** | **2772** | **tags=32%, list=21%, signal=40%** |
| **ION_TRANSPORT** | **ION_TRANSPORT** | **Details ...** | **159** | **-0.37869576** | **-1.6443461** | **0** | **0.11902755** | **0.994** | **3600** | **tags=41%, list=27%, signal=55%** |
| **HEART_DEVELOPMENT** | **HEART_DEVELOPMENT** | **Details ...** | **32** | **-0.4972953** | **-1.6424997** | **0.00870827** | **0.11864647** | **0.994** | **1561** | **tags=38%, list=12%, signal=42%** |
| **HEMOPOIESIS** | **HEMOPOIESIS** | **Details ...** | **66** | **-0.42320734** | **-1.6254933** | **0.00266312** | **0.13578159** | **0.999** | **3201** | **tags=36%, list=24%, signal=48%** |
| **CELL_FATE_COMMITMENT** | **CELL_FATE_COMMITMENT** | **Details ...** | **13** | **-0.6286998** | **-1.6236808** | **0.01664145** | **0.13479699** | **0.999** | **1223** | **tags=54%, list=9%, signal=59%** |
| **SYSTEM_DEVELOPMENT** | **SYSTEM_DEVELOPMENT** | **Details ...** | **736** | **-0.3319654** | **-1.6190419** | **0** | **0.13761187** | **0.999** | **3562** | **tags=37%, list=27%, signal=48%** |
| **REGULATION_OF_CELL_SHAPE** | **REGULATION_OF_CELL_SHAPE** | **Details ...** | **8** | **-0.70275086** | **-1.6173549** | **0.02106969** | **0.13692789** | **0.999** | **850** | **tags=25%, list=6%, signal=27%** |
| **CYTOKINE_BIOSYNTHETIC_PROCESS** | **CYTOKINE_BIOSYNTHETIC_PROCESS** | **Details ...** | **33** | **-0.48777562** | **-1.6159624** | **0.01408451** | **0.1357506** | **0.999** | **3459** | **tags=58%, list=26%, signal=78%** |
| **POSITIVE_REGULATION_OF_T_CELL_PROLIFERATION** | **POSITIVE_REGULATION_OF_T_CELL_PROLIFERATION** | **Details ...** | **12** | **-0.6027894** | **-1.6158615** | **0.02419355** | **0.13315406** | **0.999** | **2772** | **tags=58%, list=21%, signal=74%** |
| **ANATOMICAL_STRUCTURE_DEVELOPMENT** | **ANATOMICAL_STRUCTURE_DEVELOPMENT** | | **868** | **-0.3274822** | **-1.5991975** | **0** | **0.15197913** | **1** | **3562** | **tags=37%, list=27%, signal=47%** |
| **POSITIVE_REGULATION_OF_MULTICELLULAR_ORGANISMAL_PROCESS** | **POSITIVE_REGULATION_OF_MULTICELLULAR_ORGANISMAL_PROCESS** | | **52** | **-0.4383512** | **-1.5970265** | **0.01198402** | **0.1519937** | **1** | **2772** | **tags=37%, list=21%, signal=46%** |
| **REGULATION_OF_CYTOKINE_PRODUCTION** | **REGULATION_OF_CYTOKINE_PRODUCTION** | | **20** | **-0.53557444** | **-1.593515** | **0.02049781** | **0.15405388** | **1** | **2772** | **tags=50%, list=21%, signal=63%** |
| **CELL_ACTIVATION** | **CELL_ACTIVATION** | | **63** | **-0.4174704** | **-1.5926677** | **0.01749664** | **0.15259428** | **1** | **2920** | **tags=38%, list=22%, signal=49%** |
| **ION_HOMEOSTASIS** | **ION_HOMEOSTASIS** | | **110** | **-0.38371852** | **-1.5895035** | **0.00124069** | **0.15414087** | **1** | **4805** | **tags=52%, list=36%, signal=80%** |
| **CELLULAR_LIPID_CATABOLIC_PROCESS** | **CELLULAR_LIPID_CATABOLIC_PROCESS** | | **28** | **-0.4951376** | **-1.5878175** | **0.01601164** | **0.15348302** | **1** | **2104** | **tags=46%, list=16%, signal=55%** |
| **MULTICELLULAR_ORGANISMAL_DEVELOPMENT** | **MULTICELLULAR_ORGANISMAL_DEVELOPMENT** | | **899** | **-0.3247173** | **-1.5868226** | **0** | **0.15204662** | **1** | **3562** | **tags=36%, list=27%, signal=46%** |
| **FEEDING_BEHAVIOR** | **FEEDING_BEHAVIOR** | | **19** | **-0.53565913** | **-1.5830921** | **0.02799378** | **0.1541141** | **1** | **2549** | **tags=53%, list=19%, signal=65%** |
| **DETECTION_OF_ABIOTIC_STIMULUS** | **DETECTION_OF_ABIOTIC_STIMULUS** | | **16** | **-0.56743115** | **-1.5774208** | **0.02199413** | **0.1591373** | **1** | **5073** | **tags=75%, list=38%, signal=121%** |
| **FEMALE_PREGNANCY** | **FEMALE_PREGNANCY** | | **41** | **-0.4511655** | **-1.5724405** | **0.01525659** | **0.16319005** | **1** | **3609** | **tags=46%, list=27%, signal=63%** |
| **NEUROTRANSMITTER_SECRETION** | **NEUROTRANSMITTER_SECRETION** | | **11** | **-0.612859** | **-1.5649868** | **0.03599374** | **0.17156887** | **1** | **2193** | **tags=45%, list=16%, signal=54%** |
| **EPIDERMIS_DEVELOPMENT** | **EPIDERMIS_DEVELOPMENT** | | **65** | **-0.40826017** | **-1.5611477** | **0.01086957** | **0.17474696** | **1** | **2272** | **tags=37%, list=17%, signal=44%** |
| **REGULATION_OF_CYTOKINE_BIOSYNTHETIC_PROCESS** | **REGULATION_OF_CYTOKINE_BIOSYNTHETIC_PROCESS** | | **31** | **-0.46674076** | **-1.5603786** | **0.02161383** | **0.17298542** | **1** | **3459** | **tags=55%, list=26%, signal=74%** |
| **BEHAVIOR** | **BEHAVIOR** |  | **132** | **-0.3691984** | **-1.5599685** | **0.00116959** | **0.17098933** | **1** | **5313** | **tags=58%, list=40%, signal=95%** |
| **CATION_HOMEOSTASIS** | **CATION_HOMEOSTASIS** | | **93** | **-0.38692072** | **-1.5594599** | **0.01034929** | **0.16905648** | **1** | **3833** | **tags=41%, list=29%, signal=57%** |
| **NERVOUS_SYSTEM_DEVELOPMENT** | **NERVOUS_SYSTEM_DEVELOPMENT** | | **314** | **-0.33730876** | **-1.5592012** | **0.00219298** | **0.16678** | **1** | **3590** | **tags=40%, list=27%, signal=53%** |
| **REPRODUCTIVE_PROCESS** | **REPRODUCTIVE_PROCESS** | | **134** | **-0.36670342** | **-1.5533968** | **0.00120337** | **0.17294502** | **1** | **3800** | **tags=41%, list=29%, signal=57%** |
| **LEUKOCYTE_ACTIVATION** | **LEUKOCYTE_ACTIVATION** | | **58** | **-0.4203703** | **-1.5526726** | **0.01460823** | **0.17166029** | **1** | **2920** | **tags=38%, list=22%, signal=48%** |
| **DETECTION_OF_STIMULUS_INVOLVED_IN_SENSORY_PERCEPTION** | **DETECTION_OF_STIMULUS_INVOLVED_IN_SENSORY_PERCEPTION** | | **15** | **-0.5656358** | **-1.5408313** | **0.0477658** | **0.18733035** | **1** | **4850** | **tags=73%, list=36%, signal=115%** |
| **REGULATION_OF_HEART_CONTRACTION** | **REGULATION_OF_HEART_CONTRACTION** | | **24** | **-0.5007534** | **-1.5386531** | **0.02737752** | **0.1876995** | **1** | **3600** | **tags=50%, list=27%, signal=68%** |
| **G_PROTEIN_SIGNALING_ADENYLATE_CYCLASE_ACTIVATING_PATHWAY** | **G_PROTEIN_SIGNALING_ADENYLATE_CYCLASE_ACTIVATING_PATHWAY** | | **25** | **-0.4892637** | **-1.5336074** | **0.02857143** | **0.1932214** | **1** | **3670** | **tags=64%, list=28%, signal=88%** |
| **ADAPTIVE_IMMUNE_RESPONSE_GO_0002460** | **ADAPTIVE_IMMUNE_RESPONSE_GO_0002460** | | **19** | **-0.5152334** | **-1.5315472** | **0.02481752** | **0.19364192** | **1** | **2772** | **tags=42%, list=21%, signal=53%** |
| **EXCRETION** | **EXCRETION** |  | **35** | **-0.45942506** | **-1.5287776** | **0.02753623** | **0.19515926** | **1** | **4009** | **tags=54%, list=30%, signal=77%** |
| **POSITIVE_REGULATION_OF_IMMUNE_SYSTEM_PROCESS** | **POSITIVE_REGULATION_OF_IMMUNE_SYSTEM_PROCESS** | | **41** | **-0.4418397** | **-1.5270731** | **0.01790634** | **0.19519673** | **1** | **2772** | **tags=37%, list=21%, signal=46%** |
| **ADAPTIVE_IMMUNE_RESPONSE** | **ADAPTIVE_IMMUNE_RESPONSE** | | **20** | **-0.5105161** | **-1.5242641** | **0.03453454** | **0.19668032** | **1** | **2772** | **tags=40%, list=21%, signal=50%** |
| **NEGATIVE_REGULATION_OF_MULTICELLULAR_ORGANISMAL_PROCESS** | **NEGATIVE_REGULATION_OF_MULTICELLULAR_ORGANISMAL_PROCESS** | | **26** | **-0.47906634** | **-1.5242018** | **0.03813559** | **0.19419447** | **1** | **1794** | **tags=31%, list=13%, signal=35%** |
| **HORMONE_SECRETION** | **HORMONE_SECRETION** | | **14** | **-0.56836826** | **-1.5189927** | **0.03736921** | **0.19924667** | **1** | **3194** | **tags=64%, list=24%, signal=84%** |
| **CELL_SURFACE_RECEPTOR_LINKED_SIGNAL_TRANSDUCTION_GO_0007166** | **CELL_SURFACE_RECEPTOR_LINKED_SIGNAL_TRANSDUCTION_GO_0007166** | | **554** | **-0.31552845** | **-1.5183394** | **0** | **0.19777404** | **1** | **3533** | **tags=35%, list=27%, signal=45%** |
| **HOMEOSTATIC_PROCESS** | **HOMEOSTATIC_PROCESS** | | **175** | **-0.34311104** | **-1.5151511** | **0.00232829** | **0.19999692** | **1** | **4805** | **tags=48%, list=36%, signal=74%** |
| **CELLULAR_CATION_HOMEOSTASIS** | **CELLULAR_CATION_HOMEOSTASIS** | | **90** | **-0.37740982** | **-1.5116974** | **0.01620948** | **0.20267104** | **1** | **3488** | **tags=37%, list=26%, signal=49%** |
| **BODY_FLUID_SECRETION** | **BODY_FLUID_SECRETION** | | **10** | **-0.61360043** | **-1.5097643** | **0.05354331** | **0.20333637** | **1** | **2889** | **tags=60%, list=22%, signal=77%** |
| **HUMORAL_IMMUNE_RESPONSE** | **HUMORAL_IMMUNE_RESPONSE** | | **27** | **-0.46387345** | **-1.5088723** | **0.0463097** | **0.2021258** | **1** | **3772** | **tags=48%, list=28%, signal=67%** |
| **DEFENSE_RESPONSE** | **DEFENSE_RESPONSE** | | **222** | **-0.33389214** | **-1.5052376** | **0.00685714** | **0.20499958** | **1** | **3165** | **tags=32%, list=24%, signal=42%** |
| **REGULATION_OF_MAPKKK_CASCADE** | **REGULATION_OF_MAPKKK_CASCADE** | | **18** | **-0.5254574** | **-1.5045482** | **0.04388715** | **0.20361966** | **1** | **5979** | **tags=89%, list=45%, signal=161%** |
| **POSITIVE_REGULATION_OF_T_CELL_ACTIVATION** | **POSITIVE_REGULATION_OF_T_CELL_ACTIVATION** | | **20** | **-0.5064297** | **-1.502756** | **0.04268293** | **0.204069** | **1** | **1937** | **tags=35%, list=15%, signal=41%** |
| **T_CELL_PROLIFERATION** | **T_CELL_PROLIFERATION** | | **16** | **-0.5361827** | **-1.502241** | **0.03230769** | **0.20249183** | **1** | **2772** | **tags=50%, list=21%, signal=63%** |
| **POSITIVE_REGULATION_OF_TRANSLATION** | **POSITIVE_REGULATION_OF_TRANSLATION** | | **28** | **-0.4634086** | **-1.5011647** | **0.0295421** | **0.20172845** | **1** | **3054** | **tags=50%, list=23%, signal=65%** |
| **RESPONSE_TO_XENOBIOTIC_STIMULUS** | **RESPONSE_TO_XENOBIOTIC_STIMULUS** | | **9** | **-0.63503736** | **-1.4985801** | **0.05172414** | **0.20319442** | **1** | **2844** | **tags=56%, list=21%, signal=71%** |
| **REGULATION_OF_BODY_FLUID_LEVELS** | **REGULATION_OF_BODY_FLUID_LEVELS** | | **54** | **-0.40261236** | **-1.4982054** | **0.02557201** | **0.2016629** | **1** | **3728** | **tags=41%, list=28%, signal=56%** |
| **STEROID_METABOLIC_PROCESS** | **STEROID_METABOLIC_PROCESS** | | **63** | **-0.38762364** | **-1.4957464** | **0.02281879** | **0.20298795** | **1** | **4013** | **tags=46%, list=30%, signal=66%** |
| **CIRCADIAN_RHYTHM** | **CIRCADIAN_RHYTHM** | | **13** | **-0.56781006** | **-1.4934841** | **0.04166667** | **0.20379947** | **1** | **1318** | **tags=38%, list=10%, signal=43%** |
| **REGULATION_OF_T_CELL_PROLIFERATION** | **REGULATION_OF_T_CELL_PROLIFERATION** | | **14** | **-0.56107986** | **-1.4908973** | **0.0488959** | **0.20532688** | **1** | **2772** | **tags=50%, list=21%, signal=63%** |
| **TISSUE_DEVELOPMENT** | **TISSUE_DEVELOPMENT** | | **122** | **-0.3544942** | **-1.484755** | **0.0060024** | **0.21264265** | **1** | **2272** | **tags=32%, list=17%, signal=38%** |
| **MYELOID_LEUKOCYTE_DIFFERENTIATION** | **MYELOID_LEUKOCYTE_DIFFERENTIATION** | | **14** | **-0.5592109** | **-1.4832789** | **0.05333334** | **0.21241169** | **1** | **4210** | **tags=64%, list=32%, signal=94%** |
| **CENTRAL_NERVOUS_SYSTEM_DEVELOPMENT** | **CENTRAL_NERVOUS_SYSTEM_DEVELOPMENT** | | **99** | **-0.36569464** | **-1.4827734** | **0.01477833** | **0.21103391** | **1** | **2939** | **tags=38%, list=22%, signal=49%** |
| **POSITIVE_REGULATION_OF_EPITHELIAL_CELL_PROLIFERATION** | **POSITIVE_REGULATION_OF_EPITHELIAL_CELL_PROLIFERATION** | | **7** | **-0.67014045** | **-1.4796857** | **0.05797102** | **0.2136958** | **1** | **2931** | **tags=43%, list=22%, signal=55%** |
| **CELLULAR_HOMEOSTASIS** | **CELLULAR_HOMEOSTASIS** | | **119** | **-0.35498664** | **-1.4777457** | **0.00889454** | **0.21467793** | **1** | **4805** | **tags=49%, list=36%, signal=76%** |
| **DIGESTION** | **DIGESTION** |  | **40** | **-0.42739406** | **-1.4771779** | **0.0264993** | **0.21322845** | **1** | **2549** | **tags=40%, list=19%, signal=49%** |
| **LYMPHOCYTE_DIFFERENTIATION** | **LYMPHOCYTE_DIFFERENTIATION** | | **23** | **-0.47867507** | **-1.4735498** | **0.04405286** | **0.21677281** | **1** | **3162** | **tags=35%, list=24%, signal=46%** |
| **PRODUCTION_OF_MOLECULAR_MEDIATOR_OF_IMMUNE_RESPONSE** | **PRODUCTION_OF_MOLECULAR_MEDIATOR_OF_IMMUNE_RESPONSE** | | **11** | **-0.5718203** | **-1.4731673** | **0.05188679** | **0.21528418** | **1** | **2766** | **tags=45%, list=21%, signal=57%** |
| **EXTRACELLULAR_STRUCTURE_ORGANIZATION_AND_BIOGENESIS** | **EXTRACELLULAR_STRUCTURE_ORGANIZATION_AND_BIOGENESIS** | | **22** | **-0.48905814** | **-1.4716078** | **0.05743741** | **0.21561512** | **1** | **2155** | **tags=41%, list=16%, signal=49%** |
| **REGULATION_OF_SECRETION** | **REGULATION_OF_SECRETION** | | **29** | **-0.4554353** | **-1.4704312** | **0.03290415** | **0.21529698** | **1** | **3194** | **tags=45%, list=24%, signal=59%** |
| **MORPHOGENESIS_OF_AN_EPITHELIUM** | **MORPHOGENESIS_OF_AN_EPITHELIUM** | | **14** | **-0.55173206** | **-1.4695302** | **0.07153729** | **0.21455215** | **1** | **3252** | **tags=50%, list=24%, signal=66%** |
| **PHOTOTRANSDUCTION** | **PHOTOTRANSDUCTION** | | **11** | **-0.57778996** | **-1.468076** | **0.05714286** | **0.2146461** | **1** | **4850** | **tags=73%, list=36%, signal=114%** |
| **EMBRYONIC_MORPHOGENESIS** | **EMBRYONIC_MORPHOGENESIS** | | **14** | **-0.53500533** | **-1.4662191** | **0.05952381** | **0.21543148** | **1** | **1259** | **tags=36%, list=9%, signal=39%** |
| **XENOBIOTIC_METABOLIC_PROCESS** | **XENOBIOTIC_METABOLIC_PROCESS** | | **8** | **-0.6438566** | **-1.4636722** | **0.04574132** | **0.21765794** | **1** | **2844** | **tags=63%, list=21%, signal=79%** |
| **NEGATIVE_REGULATION_OF_RESPONSE_TO_STIMULUS** | **NEGATIVE_REGULATION_OF_RESPONSE_TO_STIMULUS** | | **9** | **-0.61165243** | **-1.4560753** | **0.06362154** | **0.22746606** | **1** | **1318** | **tags=33%, list=10%, signal=37%** |
| **TRIACYLGLYCEROL_METABOLIC_PROCESS** | **TRIACYLGLYCEROL_METABOLIC_PROCESS** | | **6** | **-0.6762106** | **-1.4525262** | **0.06132879** | **0.23092625** | **1** | **666** | **tags=50%, list=5%, signal=53%** |
| **SODIUM_ION_TRANSPORT** | **SODIUM_ION_TRANSPORT** | | **15** | **-0.53472805** | **-1.451215** | **0.08206687** | **0.2309741** | **1** | **3532** | **tags=60%, list=27%, signal=82%** |
| **ORGAN_DEVELOPMENT** | **ORGAN_DEVELOPMENT** | | **493** | **-0.30343896** | **-1.4508855** | **0** | **0.22939013** | **1** | **3560** | **tags=35%, list=27%, signal=47%** |
| **REGULATION_OF_IMMUNE_RESPONSE** | **REGULATION_OF_IMMUNE_RESPONSE** | | **26** | **-0.4634391** | **-1.4499395** | **0.04081633** | **0.2291301** | **1** | **2772** | **tags=38%, list=21%, signal=48%** |
| **MITOTIC_SISTER_CHROMATID_SEGREGATION** | **MITOTIC_SISTER_CHROMATID_SEGREGATION** | | **12** | **-0.56427467** | **-1.4493352** | **0.05451714** | **0.22809651** | **1** | **1133** | **tags=33%, list=9%, signal=36%** |
| **IMMUNE_SYSTEM_PROCESS** | **IMMUNE_SYSTEM_PROCESS** | | **286** | **-0.317001** | **-1.4479502** | **0.00110254** | **0.22816199** | **1** | **3201** | **tags=31%, list=24%, signal=41%** |
| **REGULATED_SECRETORY_PATHWAY** | **REGULATED_SECRETORY_PATHWAY** | | **12** | **-0.54977655** | **-1.4457959** | **0.08682171** | **0.22952603** | **1** | **2193** | **tags=42%, list=16%, signal=50%** |
| **POSITIVE_REGULATION_OF_TRANSFERASE_ACTIVITY** | **POSITIVE_REGULATION_OF_TRANSFERASE_ACTIVITY** | | **68** | **-0.37571907** | **-1.4432244** | **0.03942181** | **0.23178339** | **1** | **4075** | **tags=47%, list=31%, signal=67%** |
| **REGULATION_OF_BIOLOGICAL_QUALITY** | **REGULATION_OF_BIOLOGICAL_QUALITY** | | **351** | **-0.30808944** | **-1.4413059** | **0.00108696** | **0.23280995** | **1** | **3503** | **tags=33%, list=26%, signal=44%** |
| **DETECTION_OF_CHEMICAL_STIMULUS** | **DETECTION_OF_CHEMICAL_STIMULUS** | | **13** | **-0.5338619** | **-1.4332721** | **0.06821705** | **0.24428838** | **1** | **4688** | **tags=77%, list=35%, signal=119%** |
| **REPRODUCTION** | **REPRODUCTION** | | **212** | **-0.32219526** | **-1.431284** | **0.00918485** | **0.24575175** | **1** | **2945** | **tags=33%, list=22%, signal=42%** |
| **RESPONSE_TO_EXTERNAL_STIMULUS** | **RESPONSE_TO_EXTERNAL_STIMULUS** | | **265** | **-0.31280667** | **-1.4294912** | **0.00333704** | **0.24666584** | **1** | **3872** | **tags=37%, list=29%, signal=51%** |
| **LOCOMOTORY_BEHAVIOR** | **LOCOMOTORY_BEHAVIOR** | | **83** | **-0.3603576** | **-1.4294386** | **0.02635046** | **0.24469955** | **1** | **3858** | **tags=41%, list=29%, signal=57%** |
| **EPITHELIAL_CELL_DIFFERENTIATION** | **EPITHELIAL_CELL_DIFFERENTIATION** | | **9** | **-0.61375326** | **-1.4290017** | **0.07704403** | **0.24345484** | **1** | **4739** | **tags=89%, list=36%, signal=138%** |
| **MITOCHONDRIAL_MEMBRANE_ORGANIZATION_AND_BIOGENESIS** | **MITOCHONDRIAL_MEMBRANE_ORGANIZATION_AND_BIOGENESIS** | | **9** | **-0.5898514** | **-1.4232436** | **0.09430894** | **0.2513888** | **1** | **1538** | **tags=44%, list=12%, signal=50%** |
| **POSITIVE_REGULATION_OF_LYMPHOCYTE_ACTIVATION** | **POSITIVE_REGULATION_OF_LYMPHOCYTE_ACTIVATION** | | **22** | **-0.46338773** | **-1.423084** | **0.0639881** | **0.24960871** | **1** | **1937** | **tags=32%, list=15%, signal=37%** |
| **B_CELL_ACTIVATION** | **B_CELL_ACTIVATION** | | **16** | **-0.52327704** | **-1.4224749** | **0.07621951** | **0.24862237** | **1** | **3162** | **tags=44%, list=24%, signal=57%** |
| **SYNAPSE_ORGANIZATION_AND_BIOGENESIS** | **SYNAPSE_ORGANIZATION_AND_BIOGENESIS** | | **14** | **-0.529043** | **-1.4211893** | **0.07843138** | **0.24874224** | **1** | **2155** | **tags=50%, list=16%, signal=60%** |
| **REGULATION_OF_CELL_MORPHOGENESIS** | **REGULATION_OF_CELL_MORPHOGENESIS** | | **9** | **-0.5872135** | **-1.4201097** | **0.08945687** | **0.24852549** | **1** | **850** | **tags=22%, list=6%, signal=24%** |
| **SISTER_CHROMATID_SEGREGATION** | **SISTER_CHROMATID_SEGREGATION** | | **13** | **-0.5388877** | **-1.4125801** | **0.07956319** | **0.25937966** | **1** | **4347** | **tags=69%, list=33%, signal=103%** |
| **NEGATIVE_REGULATION_OF_CYTOKINE_BIOSYNTHETIC_PROCESS** | **NEGATIVE_REGULATION_OF_CYTOKINE_BIOSYNTHETIC_PROCESS** | | **9** | **-0.5897814** | **-1.4079624** | **0.10048623** | **0.26549488** | **1** | **3162** | **tags=56%, list=24%, signal=73%** |
| **IMMUNE_RESPONSE** | **IMMUNE_RESPONSE** | | **203** | **-0.31706914** | **-1.4049346** | **0.00340909** | **0.2691313** | **1** | **3858** | **tags=36%, list=29%, signal=51%** |
| **MALE_GONAD_DEVELOPMENT** | **MALE_GONAD_DEVELOPMENT** | | **11** | **-0.5510033** | **-1.4008589** | **0.093702** | **0.27457857** | **1** | **3800** | **tags=64%, list=29%, signal=89%** |
| **POSITIVE_REGULATION_OF_CYTOKINE_PRODUCTION** | **POSITIVE_REGULATION_OF_CYTOKINE_PRODUCTION** | | **12** | **-0.5367654** | **-1.3993806** | **0.09508716** | **0.2749838** | **1** | **2772** | **tags=50%, list=21%, signal=63%** |
| **REGULATION_OF_KINASE_ACTIVITY** | **REGULATION_OF_KINASE_ACTIVITY** | | **131** | **-0.33115622** | **-1.3892758** | **0.02200489** | **0.2913668** | **1** | **4075** | **tags=40%, list=31%, signal=58%** |
| **ADENYLATE_CYCLASE_ACTIVATION** | **ADENYLATE_CYCLASE_ACTIVATION** | | **19** | **-0.47834063** | **-1.3892705** | **0.09985097** | **0.28919777** | **1** | **3670** | **tags=68%, list=28%, signal=94%** |
| **MYELOID_CELL_DIFFERENTIATION** | **MYELOID_CELL_DIFFERENTIATION** | | **32** | **-0.41664842** | **-1.3884814** | **0.08345324** | **0.2885528** | **1** | **3201** | **tags=38%, list=24%, signal=49%** |
| **IMMUNE_EFFECTOR_PROCESS** | **IMMUNE_EFFECTOR_PROCESS** | | **31** | **-0.41878253** | **-1.3874372** | **0.07439199** | **0.2883447** | **1** | **3117** | **tags=35%, list=23%, signal=46%** |
| **SPHINGOLIPID_BIOSYNTHETIC_PROCESS** | **SPHINGOLIPID_BIOSYNTHETIC_PROCESS** | | **5** | **-0.6893551** | **-1.3867248** | **0.07807309** | **0.2875736** | **1** | **2273** | **tags=60%, list=17%, signal=72%** |
| **REGULATION_OF_PROTEIN_KINASE_ACTIVITY** | **REGULATION_OF_PROTEIN_KINASE_ACTIVITY** | | **130** | **-0.3283148** | **-1.3845302** | **0.04005006** | **0.2896695** | **1** | **4075** | **tags=40%, list=31%, signal=57%** |
| **T_CELL_DIFFERENTIATION** | **T_CELL_DIFFERENTIATION** | | **14** | **-0.51348126** | **-1.3814749** | **0.10625** | **0.2935589** | **1** | **2772** | **tags=29%, list=21%, signal=36%** |
| **RRNA_PROCESSING** | **RRNA_PROCESSING** | | **8** | **-0.60071355** | **-1.3812513** | **0.1101549** | **0.29189828** | **1** | **625** | **tags=25%, list=5%, signal=26%** |
| **POSITIVE_REGULATION_OF_MAP_KINASE_ACTIVITY** | **POSITIVE_REGULATION_OF_MAP_KINASE_ACTIVITY** | | **39** | **-0.3953882** | **-1.381248** | **0.07876231** | **0.28984812** | **1** | **4075** | **tags=51%, list=31%, signal=74%** |
| **CARBOXYLIC_ACID_TRANSPORT** | **CARBOXYLIC_ACID_TRANSPORT** | | **41** | **-0.3917675** | **-1.374151** | **0.08732394** | **0.30108616** | **1** | **4363** | **tags=44%, list=33%, signal=65%** |
| **REGULATION_OF_BLOOD_PRESSURE** | **REGULATION_OF_BLOOD_PRESSURE** | | **20** | **-0.45865002** | **-1.3728275** | **0.09185185** | **0.30160502** | **1** | **2927** | **tags=40%, list=22%, signal=51%** |
| **ANATOMICAL_STRUCTURE_MORPHOGENESIS** | **ANATOMICAL_STRUCTURE_MORPHOGENESIS** | | **324** | **-0.29568318** | **-1.3727844** | **0.0075431** | **0.29958367** | **1** | **3641** | **tags=36%, list=27%, signal=49%** |
| **REGULATION_OF_TRANSFERASE_ACTIVITY** | **REGULATION_OF_TRANSFERASE_ACTIVITY** | | **133** | **-0.3208637** | **-1.3711561** | **0.0323741** | **0.30079916** | **1** | **4075** | **tags=40%, list=31%, signal=57%** |
| **REGULATION_OF_CELL_MIGRATION** | **REGULATION_OF_CELL_MIGRATION** | | **23** | **-0.44483387** | **-1.3667026** | **0.10730253** | **0.30741325** | **1** | **1722** | **tags=35%, list=13%, signal=40%** |
| **VIRAL_REPRODUCTION** | **VIRAL_REPRODUCTION** | | **38** | **-0.39983308** | **-1.3650992** | **0.08836524** | **0.3084831** | **1** | **1670** | **tags=26%, list=13%, signal=30%** |
| **ORGANIC_ACID_TRANSPORT** | **ORGANIC_ACID_TRANSPORT** | | **41** | **-0.3917675** | **-1.357617** | **0.07865169** | **0.32036892** | **1** | **4363** | **tags=44%, list=33%, signal=65%** |
| **NEGATIVE_REGULATION_OF_CELL_MIGRATION** | **NEGATIVE_REGULATION_OF_CELL_MIGRATION** | | **14** | **-0.50580317** | **-1.3567146** | **0.12443778** | **0.3200332** | **1** | **1409** | **tags=36%, list=11%, signal=40%** |
| **RIBOSOME_BIOGENESIS_AND_ASSEMBLY** | **RIBOSOME_BIOGENESIS_AND_ASSEMBLY** | | **10** | **-0.54261935** | **-1.3557825** | **0.12824675** | **0.3194902** | **1** | **2440** | **tags=40%, list=18%, signal=49%** |
| **GLYCOSPHINGOLIPID_METABOLIC_PROCESS** | **GLYCOSPHINGOLIPID_METABOLIC_PROCESS** | | **11** | **-0.536466** | **-1.354807** | **0.12440191** | **0.31935945** | **1** | **2273** | **tags=45%, list=17%, signal=55%** |
| **DEVELOPMENTAL_MATURATION** | **DEVELOPMENTAL_MATURATION** | | **17** | **-0.47194088** | **-1.3507388** | **0.10852713** | **0.32510284** | **1** | **4430** | **tags=53%, list=33%, signal=79%** |
| **REGULATION_OF_DEFENSE_RESPONSE** | **REGULATION_OF_DEFENSE_RESPONSE** | | **14** | **-0.49422833** | **-1.3482264** | **0.11346445** | **0.3277839** | **1** | **2772** | **tags=43%, list=21%, signal=54%** |
| **REGULATION_OF_RESPONSE_TO_STIMULUS** | **REGULATION_OF_RESPONSE_TO_STIMULUS** | | **47** | **-0.37406468** | **-1.3481245** | **0.07712766** | **0.32585928** | **1** | **2772** | **tags=32%, list=21%, signal=40%** |
| **CALCIUM_MEDIATED_SIGNALING** | **CALCIUM_MEDIATED_SIGNALING** | | **13** | **-0.51415473** | **-1.3479782** | **0.11961722** | **0.32401714** | **1** | **956** | **tags=31%, list=7%, signal=33%** |
| **REGULATION_OF_JNK_CASCADE** | **REGULATION_OF_JNK_CASCADE** | | **11** | **-0.5374068** | **-1.3477619** | **0.11987381** | **0.3223054** | **1** | **5979** | **tags=91%, list=45%, signal=165%** |
| **POSITIVE_REGULATION_OF_CYTOKINE_BIOSYNTHETIC_PROCESS** | **POSITIVE_REGULATION_OF_CYTOKINE_BIOSYNTHETIC_PROCESS** | | **21** | **-0.44707155** | **-1.3457726** | **0.10441177** | **0.3240659** | **1** | **3054** | **tags=52%, list=23%, signal=68%** |
| **LIPID_HOMEOSTASIS** | **LIPID_HOMEOSTASIS** | | **15** | **-0.47784433** | **-1.3414955** | **0.12648809** | **0.33045888** | **1** | **1881** | **tags=40%, list=14%, signal=47%** |
| **RRNA_METABOLIC_PROCESS** | **RRNA_METABOLIC_PROCESS** | | **9** | **-0.5474038** | **-1.3333726** | **0.14790997** | **0.34442872** | **1** | **625** | **tags=22%, list=5%, signal=23%** |
| **S_PHASE_OF_MITOTIC_CELL_CYCLE** | **S_PHASE_OF_MITOTIC_CELL_CYCLE** | | **8** | **-0.5791782** | **-1.3306248** | **0.12965964** | **0.34796628** | **1** | **2355** | **tags=50%, list=18%, signal=61%** |
| **RESPONSE_TO_VIRUS** | **RESPONSE_TO_VIRUS** | | **43** | **-0.37340495** | **-1.3289374** | **0.10881542** | **0.34939307** | **1** | **1737** | **tags=21%, list=13%, signal=24%** |
| **HEMOSTASIS** | **HEMOSTASIS** | | **45** | **-0.37190163** | **-1.32737** | **0.08516484** | **0.3505528** | **1** | **3728** | **tags=38%, list=28%, signal=52%** |
| **AMINO_ACID_TRANSPORT** | **AMINO_ACID_TRANSPORT** | | **26** | **-0.41570976** | **-1.3269404** | **0.12753624** | **0.34943706** | **1** | **5272** | **tags=54%, list=40%, signal=89%** |
| **G2_M_TRANSITION_OF_MITOTIC_CELL_CYCLE** | **G2_M_TRANSITION_OF_MITOTIC_CELL_CYCLE** | | **12** | **-0.5124828** | **-1.3236014** | **0.15975423** | **0.35400963** | **1** | **1090** | **tags=33%, list=8%, signal=36%** |
| **NEGATIVE_REGULATION_OF_BIOSYNTHETIC_PROCESS** | **NEGATIVE_REGULATION_OF_BIOSYNTHETIC_PROCESS** | | **24** | **-0.42503402** | **-1.3223256** | **0.14032497** | **0.35448715** | **1** | **3295** | **tags=42%, list=25%, signal=55%** |
| **G_PROTEIN_SIGNALING_ADENYLATE_CYCLASE_INHIBITING_PATHWAY** | **G_PROTEIN_SIGNALING_ADENYLATE_CYCLASE_INHIBITING_PATHWAY** | | **10** | **-0.5349055** | **-1.3218483** | **0.12954186** | **0.35328916** | **1** | **1670** | **tags=40%, list=13%, signal=46%** |
| **SYNAPTOGENESIS** | **SYNAPTOGENESIS** | | **10** | **-0.5493849** | **-1.3214823** | **0.12439419** | **0.352005** | **1** | **2155** | **tags=60%, list=16%, signal=72%** |
| **SECRETION** | **SECRETION** |  | **149** | **-0.30927977** | **-1.318882** | **0.0430622** | **0.3556022** | **1** | **3578** | **tags=35%, list=27%, signal=47%** |
| **GLYCOLIPID_METABOLIC_PROCESS** | **GLYCOLIPID_METABOLIC_PROCESS** | | **13** | **-0.5059901** | **-1.3178872** | **0.1482036** | **0.355413** | **1** | **2273** | **tags=46%, list=17%, signal=56%** |
| **NEGATIVE_REGULATION_OF_CELLULAR_BIOSYNTHETIC_PROCESS** | **NEGATIVE_REGULATION_OF_CELLULAR_BIOSYNTHETIC_PROCESS** | | **23** | **-0.4293882** | **-1.3156886** | **0.13664596** | **0.35777208** | **1** | **3295** | **tags=43%, list=25%, signal=58%** |
| **ACUTE_INFLAMMATORY_RESPONSE** | **ACUTE_INFLAMMATORY_RESPONSE** | | **9** | **-0.54333043** | **-1.3143313** | **0.14239483** | **0.3584468** | **1** | **834** | **tags=22%, list=6%, signal=24%** |
| **REGULATION_OF_MYELOID_CELL_DIFFERENTIATION** | **REGULATION_OF_MYELOID_CELL_DIFFERENTIATION** | | **18** | **-0.45853806** | **-1.3090175** | **0.13636364** | **0.36748043** | **1** | **3162** | **tags=44%, list=24%, signal=58%** |
| **S_PHASE** | **S_PHASE** |  | **12** | **-0.50811297** | **-1.3082342** | **0.16747968** | **0.36696678** | **1** | **2355** | **tags=42%, list=18%, signal=51%** |
| **CENTROSOME_CYCLE** | **CENTROSOME_CYCLE** | | **7** | **-0.5930529** | **-1.3062829** | **0.16166666** | **0.3687975** | **1** | **2790** | **tags=57%, list=21%, signal=72%** |
| **MAPKKK_CASCADE_GO_0000165** | **MAPKKK_CASCADE_GO_0000165** | | **90** | **-0.3273441** | **-1.3044748** | **0.07779172** | **0.37055302** | **1** | **5198** | **tags=53%, list=39%, signal=87%** |
| **REGULATION_OF_MITOTIC_CELL_CYCLE** | **REGULATION_OF_MITOTIC_CELL_CYCLE** | | **16** | **-0.47691655** | **-1.3042556** | **0.14461538** | **0.36894503** | **1** | **5933** | **tags=81%, list=45%, signal=146%** |
| **ACTIVATION_OF_PROTEIN_KINASE_ACTIVITY** | **ACTIVATION_OF_PROTEIN_KINASE_ACTIVITY** | | **21** | **-0.43255705** | **-1.3036691** | **0.15316643** | **0.3681391** | **1** | **4591** | **tags=52%, list=34%, signal=80%** |
| **CELL_CYCLE_PHASE** | **CELL_CYCLE_PHASE** | | **140** | **-0.30593175** | **-1.3013122** | **0.06755127** | **0.37113658** | **1** | **4884** | **tags=44%, list=37%, signal=68%** |
| **VITAMIN_TRANSPORT** | **VITAMIN_TRANSPORT** | | **11** | **-0.51352364** | **-1.3008947** | **0.16055046** | **0.36995044** | **1** | **6487** | **tags=100%, list=49%, signal=195%** |
| **INFLAMMATORY_RESPONSE** | **INFLAMMATORY_RESPONSE** | | **107** | **-0.31918544** | **-1.3004373** | **0.07402761** | **0.36875996** | **1** | **3872** | **tags=37%, list=29%, signal=52%** |
| **SPINDLE_ORGANIZATION_AND_BIOGENESIS** | **SPINDLE_ORGANIZATION_AND_BIOGENESIS** | | **10** | **-0.5319231** | **-1.299831** | **0.16507937** | **0.36799383** | **1** | **2069** | **tags=30%, list=16%, signal=35%** |
| **G1_PHASE** | **G1_PHASE** |  | **13** | **-0.49511945** | **-1.2979261** | **0.17014447** | **0.36985308** | **1** | **2960** | **tags=38%, list=22%, signal=49%** |
| **POSITIVE_REGULATION_OF_MAPKKK_CASCADE** | **POSITIVE_REGULATION_OF_MAPKKK_CASCADE** | | **9** | **-0.55561894** | **-1.2940205** | **0.17609046** | **0.37603682** | **1** | **5098** | **tags=78%, list=38%, signal=126%** |
| **PROTEOGLYCAN_METABOLIC_PROCESS** | **PROTEOGLYCAN_METABOLIC_PROCESS** | | **14** | **-0.4789886** | **-1.2938485** | **0.15576324** | **0.3742868** | **1** | **4098** | **tags=64%, list=31%, signal=93%** |
| **RESPONSE_TO_WOUNDING** | **RESPONSE_TO_WOUNDING** | | **161** | **-0.2987336** | **-1.2928916** | **0.06096131** | **0.37407735** | **1** | **3872** | **tags=35%, list=29%, signal=48%** |
| **RESPONSE_TO_EXTRACELLULAR_STIMULUS** | **RESPONSE_TO_EXTRACELLULAR_STIMULUS** | | **27** | **-0.39844564** | **-1.2918802** | **0.1426492** | **0.37421635** | **1** | **2147** | **tags=30%, list=16%, signal=35%** |
| **INTERPHASE_OF_MITOTIC_CELL_CYCLE** | **INTERPHASE_OF_MITOTIC_CELL_CYCLE** | | **53** | **-0.3522932** | **-1.28892** | **0.12225275** | **0.37884828** | **1** | **4192** | **tags=40%, list=31%, signal=58%** |
| **LEARNING_AND_OR_MEMORY** | **LEARNING_AND_OR_MEMORY** | | **14** | **-0.4797647** | **-1.2865868** | **0.1612426** | **0.3815569** | **1** | **5209** | **tags=71%, list=39%, signal=117%** |
| **POSITIVE_REGULATION_OF_CELL_PROLIFERATION** | **POSITIVE_REGULATION_OF_CELL_PROLIFERATION** | | **124** | **-0.3053335** | **-1.281589** | **0.08711656** | **0.39035136** | **1** | **2458** | **tags=26%, list=18%, signal=31%** |
| **MITOTIC_SPINDLE_ORGANIZATION_AND_BIOGENESIS** | **MITOTIC_SPINDLE_ORGANIZATION_AND_BIOGENESIS** | | **9** | **-0.5496137** | **-1.2807623** | **0.15224358** | **0.3901711** | **1** | **2069** | **tags=33%, list=16%, signal=39%** |
| **MITOTIC_CELL_CYCLE** | **MITOTIC_CELL_CYCLE** | | **124** | **-0.30716172** | **-1.2806007** | **0.06748466** | **0.38840735** | **1** | **4884** | **tags=44%, list=37%, signal=69%** |
| **NEGATIVE_REGULATION_OF_MYELOID_CELL_DIFFERENTIATION** | **NEGATIVE_REGULATION_OF_MYELOID_CELL_DIFFERENTIATION** | | **10** | **-0.5122868** | **-1.2800851** | **0.19309261** | **0.3875503** | **1** | **3826** | **tags=60%, list=29%, signal=84%** |
| **MEIOTIC_CELL_CYCLE** | **MEIOTIC_CELL_CYCLE** | | **28** | **-0.3986345** | **-1.2793617** | **0.15484804** | **0.38722005** | **1** | **2945** | **tags=32%, list=22%, signal=41%** |
| **EXOCYTOSIS** | **EXOCYTOSIS** |  | **21** | **-0.41868055** | **-1.2776332** | **0.15373352** | **0.38905853** | **1** | **637** | **tags=19%, list=5%, signal=20%** |
| **G1_PHASE_OF_MITOTIC_CELL_CYCLE** | **G1_PHASE_OF_MITOTIC_CELL_CYCLE** | | **12** | **-0.50415856** | **-1.2762237** | **0.17524116** | **0.3901056** | **1** | **2960** | **tags=42%, list=22%, signal=54%** |
| **REGULATION_OF_CATALYTIC_ACTIVITY** | **REGULATION_OF_CATALYTIC_ACTIVITY** | | **231** | **-0.28401437** | **-1.2732655** | **0.04869762** | **0.3946917** | **1** | **3958** | **tags=38%, list=30%, signal=53%** |
| **REGULATION_OF_MOLECULAR_FUNCTION** | **REGULATION_OF_MOLECULAR_FUNCTION** | | **265** | **-0.27659306** | **-1.2700509** | **0.04861879** | **0.39986002** | **1** | **3958** | **tags=37%, list=30%, signal=51%** |
| **STRESS_ACTIVATED_PROTEIN_KINASE_SIGNALING_PATHWAY** | **STRESS_ACTIVATED_PROTEIN_KINASE_SIGNALING_PATHWAY** | | **45** | **-0.36192012** | **-1.266673** | **0.1396648** | **0.40510944** | **1** | **4482** | **tags=49%, list=34%, signal=73%** |
| **CDC42_PROTEIN_SIGNAL_TRANSDUCTION** | **CDC42_PROTEIN_SIGNAL_TRANSDUCTION** | | **8** | **-0.54645705** | **-1.2623395** | **0.2064** | **0.41242582** | **1** | **3075** | **tags=38%, list=23%, signal=49%** |
| **RESPONSE_TO_RADIATION** | **RESPONSE_TO_RADIATION** | | **52** | **-0.34678942** | **-1.2607567** | **0.14150943** | **0.41410297** | **1** | **4377** | **tags=46%, list=33%, signal=68%** |
| **BLOOD_COAGULATION** | **BLOOD_COAGULATION** | | **40** | **-0.36222082** | **-1.2599182** | **0.14985995** | **0.41387504** | **1** | **3728** | **tags=38%, list=28%, signal=52%** |
| **CELL_CYCLE_GO_0007049** | **CELL_CYCLE_GO_0007049** | | **260** | **-0.27385256** | **-1.2598455** | **0.05641593** | **0.41195226** | **1** | **4349** | **tags=37%, list=33%, signal=53%** |
| **COAGULATION** | **COAGULATION** | | **40** | **-0.36222082** | **-1.2598075** | **0.13861386** | **0.40998098** | **1** | **3728** | **tags=38%, list=28%, signal=52%** |
| **POSITIVE_REGULATION_OF_IMMUNE_RESPONSE** | **POSITIVE_REGULATION_OF_IMMUNE_RESPONSE** | | **22** | **-0.4112447** | **-1.2594101** | **0.16448326** | **0.40897995** | **1** | **2772** | **tags=36%, list=21%, signal=46%** |
| **CELL_MATURATION** | **CELL_MATURATION** | | **15** | **-0.4587053** | **-1.25363** | **0.19106318** | **0.4195047** | **1** | **4430** | **tags=53%, list=33%, signal=80%** |
| **CALCIUM_INDEPENDENT_CELL_CELL_ADHESION** | **CALCIUM_INDEPENDENT_CELL_CELL_ADHESION** | | **16** | **-0.44720483** | **-1.2522198** | **0.18098159** | **0.42050102** | **1** | **4241** | **tags=69%, list=32%, signal=101%** |
| **NEGATIVE_REGULATION_OF_METABOLIC_PROCESS** | **NEGATIVE_REGULATION_OF_METABOLIC_PROCESS** | | **212** | **-0.27771473** | **-1.2519451** | **0.08390023** | **0.41914332** | **1** | **3475** | **tags=31%, list=26%, signal=41%** |
| **REGULATION_OF_MAP_KINASE_ACTIVITY** | **REGULATION_OF_MAP_KINASE_ACTIVITY** | | **57** | **-0.33542234** | **-1.251792** | **0.12851405** | **0.41745028** | **1** | **4167** | **tags=47%, list=31%, signal=69%** |
| **M_PHASE** | **M_PHASE** |  | **90** | **-0.30670878** | **-1.2517825** | **0.12242268** | **0.41546232** | **1** | **4741** | **tags=43%, list=36%, signal=67%** |
| **REGULATION_OF_DNA_REPLICATION** | **REGULATION_OF_DNA_REPLICATION** | | **17** | **-0.43016863** | **-1.2497957** | **0.19248827** | **0.41803616** | **1** | **3041** | **tags=47%, list=23%, signal=61%** |
| **JNK_CASCADE** | **JNK_CASCADE** | | **44** | **-0.35309958** | **-1.2458844** | **0.15620641** | **0.42501348** | **1** | **4482** | **tags=48%, list=34%, signal=72%** |
| **AROMATIC_COMPOUND_METABOLIC_PROCESS** | **AROMATIC_COMPOUND_METABOLIC_PROCESS** | | **25** | **-0.39148113** | **-1.2396015** | **0.17043741** | **0.43770418** | **1** | **2066** | **tags=28%, list=16%, signal=33%** |
| **REGULATION_OF_CELL_CYCLE** | **REGULATION_OF_CELL_CYCLE** | | **153** | **-0.29108435** | **-1.2383592** | **0.10332542** | **0.43857276** | **1** | **4031** | **tags=35%, list=30%, signal=49%** |
| **WOUND_HEALING** | **WOUND_HEALING** | | **48** | **-0.34299925** | **-1.2374653** | **0.16870749** | **0.43836632** | **1** | **5494** | **tags=54%, list=41%, signal=92%** |
| **REGULATION_OF_CELL_GROWTH** | **REGULATION_OF_CELL_GROWTH** | | **37** | **-0.36526746** | **-1.2356229** | **0.15804195** | **0.44061196** | **1** | **3607** | **tags=41%, list=27%, signal=55%** |
| **INTERPHASE** | **INTERPHASE** | | **58** | **-0.32974795** | **-1.2330451** | **0.1701847** | **0.4444098** | **1** | **4192** | **tags=38%, list=31%, signal=55%** |
| **NEUTRAL_AMINO_ACID_TRANSPORT** | **NEUTRAL_AMINO_ACID_TRANSPORT** | | **10** | **-0.49967077** | **-1.2310212** | **0.21717991** | **0.44708145** | **1** | **5272** | **tags=70%, list=40%, signal=116%** |
| **POSITIVE_REGULATION_OF_CATALYTIC_ACTIVITY** | **POSITIVE_REGULATION_OF_CATALYTIC_ACTIVITY** | | **135** | **-0.29010877** | **-1.2308284** | **0.12378641** | **0.44548747** | **1** | **3958** | **tags=41%, list=30%, signal=57%** |
| **REGULATION_OF_HOMEOSTATIC_PROCESS** | **REGULATION_OF_HOMEOSTATIC_PROCESS** | | **12** | **-0.46585003** | **-1.2288092** | **0.22364217** | **0.44802338** | **1** | **1318** | **tags=33%, list=10%, signal=37%** |
| **CELL_CYCLE_PROCESS** | **CELL_CYCLE_PROCESS** | | **156** | **-0.28627658** | **-1.226963** | **0.11269277** | **0.45023292** | **1** | **4884** | **tags=42%, list=37%, signal=66%** |
| **RESPONSE_TO_LIGHT_STIMULUS** | **RESPONSE_TO_LIGHT_STIMULUS** | | **40** | **-0.35616645** | **-1.225949** | **0.17952314** | **0.45049378** | **1** | **4850** | **tags=52%, list=36%, signal=82%** |
| **MONOVALENT_INORGANIC_CATION_HOMEOSTASIS** | **MONOVALENT_INORGANIC_CATION_HOMEOSTASIS** | | **13** | **-0.46762162** | **-1.2246658** | **0.23354232** | **0.4516239** | **1** | **3795** | **tags=46%, list=28%, signal=64%** |
| **REGULATION_OF_PH** | **REGULATION_OF_PH** | | **12** | **-0.46852264** | **-1.2231951** | **0.19508448** | **0.45304662** | **1** | **3795** | **tags=50%, list=28%, signal=70%** |
| **VIRAL_REPRODUCTIVE_PROCESS** | **VIRAL_REPRODUCTIVE_PROCESS** | | **33** | **-0.37119493** | **-1.222098** | **0.17083947** | **0.45343798** | **1** | **1670** | **tags=24%, list=13%, signal=28%** |
| **ESTABLISHMENT_AND_OR_MAINTENANCE_OF_CELL_POLARITY** | **ESTABLISHMENT_AND_OR_MAINTENANCE_OF_CELL_POLARITY** | | **19** | **-0.41693863** | **-1.2160596** | **0.23423423** | **0.46583194** | **1** | **3422** | **tags=58%, list=26%, signal=78%** |
| **SPERM_MOTILITY** | **SPERM_MOTILITY** | | **9** | **-0.51283866** | **-1.2132701** | **0.2359736** | **0.47035283** | **1** | **5077** | **tags=78%, list=38%, signal=126%** |
| **NEGATIVE_REGULATION_OF_CELLULAR_METABOLIC_PROCESS** | **NEGATIVE_REGULATION_OF_CELLULAR_METABOLIC_PROCESS** | | **209** | **-0.2723666** | **-1.2107965** | **0.12082854** | **0.47450763** | **1** | **3475** | **tags=30%, list=26%, signal=40%** |
| **RESPONSE_TO_CHEMICAL_STIMULUS** | **RESPONSE_TO_CHEMICAL_STIMULUS** | | **266** | **-0.26493785** | **-1.2103415** | **0.09598214** | **0.47349212** | **1** | **1903** | **tags=19%, list=14%, signal=21%** |
| **NEGATIVE_REGULATION_OF_GROWTH** | **NEGATIVE_REGULATION_OF_GROWTH** | | **33** | **-0.36500534** | **-1.208053** | **0.20084865** | **0.4767507** | **1** | **3607** | **tags=42%, list=27%, signal=58%** |
| **EMBRYONIC_DEVELOPMENT** | **EMBRYONIC_DEVELOPMENT** | | **46** | **-0.33720073** | **-1.2077051** | **0.17432432** | **0.475534** | **1** | **1259** | **tags=22%, list=9%, signal=24%** |
| **POSITIVE_REGULATION_OF_CELL_DIFFERENTIATION** | **POSITIVE_REGULATION_OF_CELL_DIFFERENTIATION** | | **19** | **-0.40737823** | **-1.2046249** | **0.23006135** | **0.48084012** | **1** | **1603** | **tags=21%, list=12%, signal=24%** |
| **STRIATED_MUSCLE_CONTRACTION_GO_0006941** | **STRIATED_MUSCLE_CONTRACTION_GO_0006941** | | **14** | **-0.45096576** | **-1.2031147** | **0.23458646** | **0.4825014** | **1** | **2914** | **tags=43%, list=22%, signal=55%** |
| **GLAND_DEVELOPMENT** | **GLAND_DEVELOPMENT** | | **12** | **-0.46416852** | **-1.2029635** | **0.25038403** | **0.4807057** | **1** | **1316** | **tags=33%, list=10%, signal=37%** |
| **ACTIVATION_OF_IMMUNE_RESPONSE** | **ACTIVATION_OF_IMMUNE_RESPONSE** | | **13** | **-0.45343238** | **-1.2013264** | **0.23937008** | **0.48254332** | **1** | **1087** | **tags=23%, list=8%, signal=25%** |
| **NEGATIVE_REGULATION_OF_CELL_CYCLE** | **NEGATIVE_REGULATION_OF_CELL_CYCLE** | | **67** | **-0.3082325** | **-1.2003915** | **0.18985696** | **0.48273134** | **1** | **4867** | **tags=46%, list=37%, signal=73%** |
| **ANATOMICAL_STRUCTURE_FORMATION** | **ANATOMICAL_STRUCTURE_FORMATION** | | **48** | **-0.33725244** | **-1.2001604** | **0.1997187** | **0.48127726** | **1** | **1223** | **tags=23%, list=9%, signal=25%** |
| **REGULATION_OF_MEMBRANE_POTENTIAL** | **REGULATION_OF_MEMBRANE_POTENTIAL** | | **13** | **-0.45362052** | **-1.1990349** | **0.2326284** | **0.48173288** | **1** | **1239** | **tags=23%, list=9%, signal=25%** |
| **AMINE_TRANSPORT** | **AMINE_TRANSPORT** | | **35** | **-0.3527706** | **-1.1984378** | **0.2034384** | **0.48113903** | **1** | **4428** | **tags=43%, list=33%, signal=64%** |
| **TISSUE_MORPHOGENESIS** | **TISSUE_MORPHOGENESIS** | | **13** | **-0.45528036** | **-1.1973572** | **0.22706766** | **0.48163587** | **1** | **2015** | **tags=46%, list=15%, signal=54%** |
| **REGULATION_OF_TRANSLATION** | **REGULATION_OF_TRANSLATION** | | **64** | **-0.31605196** | **-1.1959324** | **0.17496635** | **0.48314482** | **1** | **3162** | **tags=33%, list=24%, signal=43%** |
| **ESTABLISHMENT_OF_LOCALIZATION** | **ESTABLISHMENT_OF_LOCALIZATION** | | **752** | **-0.24711592** | **-1.194696** | **0.03163265** | **0.48408833** | **1** | **3600** | **tags=30%, list=27%, signal=39%** |
| **DNA_RECOMBINATION** | **DNA_RECOMBINATION** | | **41** | **-0.33763188** | **-1.1930346** | **0.21118881** | **0.48595968** | **1** | **1555** | **tags=22%, list=12%, signal=25%** |
| **CELL_PROLIFERATION_GO_0008283** | **CELL_PROLIFERATION_GO_0008283** | | **448** | **-0.253539** | **-1.1895753** | **0.0685654** | **0.49231157** | **1** | **3447** | **tags=29%, list=26%, signal=38%** |
| **POSITIVE_REGULATION_OF_ANGIOGENESIS** | **POSITIVE_REGULATION_OF_ANGIOGENESIS** | | **9** | **-0.5037946** | **-1.1888082** | **0.25955415** | **0.4922096** | **1** | **3395** | **tags=33%, list=25%, signal=45%** |
| **NEGATIVE_REGULATION_OF_TRANSCRIPTION_FROM_RNA_POLYMERASE_II_PROMOTER** | **NEGATIVE_REGULATION_OF_TRANSCRIPTION_FROM_RNA_POLYMERASE_II_PROMOTER** | | **70** | **-0.3084836** | **-1.1883857** | **0.21038616** | **0.49114448** | **1** | **1472** | **tags=19%, list=11%, signal=21%** |
| **DNA_REPLICATION** | **DNA_REPLICATION** | | **85** | **-0.29546738** | **-1.1869612** | **0.1965924** | **0.4926062** | **1** | **3041** | **tags=28%, list=23%, signal=36%** |
| **MITOCHONDRIAL_TRANSPORT** | **MITOCHONDRIAL_TRANSPORT** | | **18** | **-0.4038437** | **-1.1867797** | **0.24639769** | **0.49100178** | **1** | **2145** | **tags=28%, list=16%, signal=33%** |
| **TISSUE_REMODELING** | **TISSUE_REMODELING** | | **28** | **-0.36598954** | **-1.1847472** | **0.22401172** | **0.4935974** | **1** | **3826** | **tags=43%, list=29%, signal=60%** |
| **GLUTAMATE_SIGNALING_PATHWAY** | **GLUTAMATE_SIGNALING_PATHWAY** | | **17** | **-0.41282853** | **-1.1832457** | **0.2462006** | **0.49525025** | **1** | **4075** | **tags=41%, list=31%, signal=59%** |
| **SPHINGOLIPID_METABOLIC_PROCESS** | **SPHINGOLIPID_METABOLIC_PROCESS** | | **21** | **-0.38912433** | **-1.1830177** | **0.23900293** | **0.49381274** | **1** | **2328** | **tags=38%, list=17%, signal=46%** |
| **REGULATION_OF_NEUROTRANSMITTER_LEVELS** | **REGULATION_OF_NEUROTRANSMITTER_LEVELS** | | **22** | **-0.3923955** | **-1.1820911** | **0.24** | **0.49410626** | **1** | **1318** | **tags=23%, list=10%, signal=25%** |
| **VIRAL_INFECTIOUS_CYCLE** | **VIRAL_INFECTIOUS_CYCLE** | | **29** | **-0.36596465** | **-1.180262** | **0.25258493** | **0.49653575** | **1** | **967** | **tags=21%, list=7%, signal=22%** |
| **NEGATIVE_REGULATION_OF_NUCLEOBASENUCLEOSIDENUCLEOTIDE_AND_NUCLEIC_ACID_METABOLIC_PROCESS** | **NEGATIVE_REGULATION_OF_NUCLEOBASENUCLEOSIDENUCLEOTIDE_AND_NUCLEIC_ACID_METABOLIC_PROCESS** | | **167** | **-0.270993** | **-1.1757159** | **0.16627078** | **0.505955** | **1** | **4140** | **tags=37%, list=31%, signal=53%** |
| **NEGATIVE_REGULATION_OF_PROTEIN_METABOLIC_PROCESS** | **NEGATIVE_REGULATION_OF_PROTEIN_METABOLIC_PROCESS** | | **42** | **-0.33763626** | **-1.1748976** | **0.21468145** | **0.50606066** | **1** | **3201** | **tags=29%, list=24%, signal=37%** |
| **PROTEOGLYCAN_BIOSYNTHETIC_PROCESS** | **PROTEOGLYCAN_BIOSYNTHETIC_PROCESS** | | **10** | **-0.48739916** | **-1.1743325** | **0.26592356** | **0.5054198** | **1** | **4098** | **tags=60%, list=31%, signal=87%** |
| **NEGATIVE_REGULATION_OF_TRANSLATION** | **NEGATIVE_REGULATION_OF_TRANSLATION** | | **17** | **-0.41395605** | **-1.1731029** | **0.2670623** | **0.5066085** | **1** | **927** | **tags=24%, list=7%, signal=25%** |
| **REGULATION_OF_CELL_PROLIFERATION** | **REGULATION_OF_CELL_PROLIFERATION** | | **262** | **-0.25767466** | **-1.1691226** | **0.13676149** | **0.51416117** | **1** | **3194** | **tags=27%, list=24%, signal=35%** |
| **DI___TRI_VALENT_INORGANIC_CATION_TRANSPORT** | **DI___TRI_VALENT_INORGANIC_CATION_TRANSPORT** | | **27** | **-0.3647444** | **-1.1631981** | **0.24517088** | **0.52694726** | **1** | **4663** | **tags=52%, list=35%, signal=80%** |
| **NEGATIVE_REGULATION_OF_CELLULAR_PROTEIN_METABOLIC_PROCESS** | **NEGATIVE_REGULATION_OF_CELLULAR_PROTEIN_METABOLIC_PROCESS** | | **39** | **-0.33776695** | **-1.1631875** | **0.25694445** | **0.5249424** | **1** | **3162** | **tags=28%, list=24%, signal=37%** |
| **MICROTUBULE_CYTOSKELETON_ORGANIZATION_AND_BIOGENESIS** | **MICROTUBULE_CYTOSKELETON_ORGANIZATION_AND_BIOGENESIS** | | **31** | **-0.35622594** | **-1.1622002** | **0.24404761** | **0.52528834** | **1** | **2100** | **tags=23%, list=16%, signal=27%** |
| **TRANSPORT** | **TRANSPORT** |  | **691** | **-0.23942034** | **-1.161246** | **0.07091469** | **0.52561843** | **1** | **3104** | **tags=25%, list=23%, signal=31%** |
| **ANGIOGENESIS** | **ANGIOGENESIS** | | **40** | **-0.33382896** | **-1.1602368** | **0.24611032** | **0.52614766** | **1** | **945** | **tags=20%, list=7%, signal=21%** |
| **MICROTUBULE_ORGANIZING_CENTER_ORGANIZATION_AND_BIOGENESIS** | **MICROTUBULE_ORGANIZING_CENTER_ORGANIZATION_AND_BIOGENESIS** | | **12** | **-0.44154334** | **-1.1593056** | **0.27531645** | **0.52648735** | **1** | **3287** | **tags=42%, list=25%, signal=55%** |
| **ONE_CARBON_COMPOUND_METABOLIC_PROCESS** | **ONE_CARBON_COMPOUND_METABOLIC_PROCESS** | | **24** | **-0.37340418** | **-1.1582328** | **0.25942028** | **0.5271058** | **1** | **3056** | **tags=42%, list=23%, signal=54%** |
| **RNA_CATABOLIC_PROCESS** | **RNA_CATABOLIC_PROCESS** | | **21** | **-0.38809675** | **-1.1580572** | **0.28096676** | **0.52549267** | **1** | **2635** | **tags=24%, list=20%, signal=30%** |
| **ORGAN_MORPHOGENESIS** | **ORGAN_MORPHOGENESIS** | | **125** | **-0.2731081** | **-1.1552607** | **0.20731707** | **0.53044945** | **1** | **2299** | **tags=25%, list=17%, signal=30%** |
| **AXONOGENESIS** | **AXONOGENESIS** | | **35** | **-0.34588474** | **-1.1538141** | **0.24686192** | **0.53218216** | **1** | **2000** | **tags=29%, list=15%, signal=34%** |
| **CELL_CYCLE_ARREST_GO_0007050** | **CELL_CYCLE_ARREST_GO_0007050** | | **50** | **-0.31978026** | **-1.1502328** | **0.26086956** | **0.53902555** | **1** | **5596** | **tags=56%, list=42%, signal=96%** |
| **SEXUAL_REPRODUCTION** | **SEXUAL_REPRODUCTION** | | **107** | **-0.27990365** | **-1.1499856** | **0.22331288** | **0.5375697** | **1** | **2945** | **tags=32%, list=22%, signal=40%** |
| **RESPONSE_TO_DRUG** | **RESPONSE_TO_DRUG** | | **20** | **-0.3894741** | **-1.1478312** | **0.28915662** | **0.54091966** | **1** | **1428** | **tags=25%, list=11%, signal=28%** |
| **LIPID_METABOLIC_PROCESS** | **LIPID_METABOLIC_PROCESS** | | **266** | **-0.25037688** | **-1.1474222** | **0.15871254** | **0.5399321** | **1** | **2376** | **tags=25%, list=18%, signal=30%** |
| **DNA_MODIFICATION** | **DNA_MODIFICATION** | | **9** | **-0.48588678** | **-1.1446841** | **0.2969984** | **0.5448677** | **1** | **2920** | **tags=44%, list=22%, signal=57%** |
| **M_PHASE_OF_MITOTIC_CELL_CYCLE** | **M_PHASE_OF_MITOTIC_CELL_CYCLE** | | **68** | **-0.29595238** | **-1.1439105** | **0.265252** | **0.5446132** | **1** | **4884** | **tags=44%, list=37%, signal=69%** |
| **CELLULAR_LIPID_METABOLIC_PROCESS** | **CELLULAR_LIPID_METABOLIC_PROCESS** | | **207** | **-0.25656626** | **-1.1407455** | **0.19767442** | **0.55068916** | **1** | **2376** | **tags=25%, list=18%, signal=30%** |
| **POSITIVE_REGULATION_OF_DEFENSE_RESPONSE** | **POSITIVE_REGULATION_OF_DEFENSE_RESPONSE** | | **8** | **-0.49296767** | **-1.1401902** | **0.30546623** | **0.55015284** | **1** | **2772** | **tags=50%, list=21%, signal=63%** |
| **MEMBRANE_LIPID_METABOLIC_PROCESS** | **MEMBRANE_LIPID_METABOLIC_PROCESS** | | **80** | **-0.28483838** | **-1.1388029** | **0.29268292** | **0.5515885** | **1** | **2376** | **tags=26%, list=18%, signal=32%** |
| **VIRAL_GENOME_REPLICATION** | **VIRAL_GENOME_REPLICATION** | | **20** | **-0.38437647** | **-1.1383584** | **0.3020231** | **0.55076975** | **1** | **1670** | **tags=25%, list=13%, signal=29%** |
| **REGULATION_OF_G_PROTEIN_COUPLED_RECEPTOR_PROTEIN_SIGNALING_PATHWAY** | **REGULATION_OF_G_PROTEIN_COUPLED_RECEPTOR_PROTEIN_SIGNALING_PATHWAY** | | **23** | **-0.3749644** | **-1.1362967** | **0.28820962** | **0.55419135** | **1** | **2253** | **tags=39%, list=17%, signal=47%** |
| **NEUROPEPTIDE_SIGNALING_PATHWAY** | **NEUROPEPTIDE_SIGNALING_PATHWAY** | | **12** | **-0.44542176** | **-1.1347665** | **0.30191693** | **0.55609185** | **1** | **6817** | **tags=83%, list=51%, signal=171%** |
| **INTERFERON_GAMMA_BIOSYNTHETIC_PROCESS** | **INTERFERON_GAMMA_BIOSYNTHETIC_PROCESS** | | **9** | **-0.47682205** | **-1.1345747** | **0.3207856** | **0.5546494** | **1** | **3162** | **tags=44%, list=24%, signal=58%** |
| **CELLULAR_RESPONSE_TO_EXTRACELLULAR_STIMULUS** | **CELLULAR_RESPONSE_TO_EXTRACELLULAR_STIMULUS** | | **8** | **-0.49006236** | **-1.1339905** | **0.32419354** | **0.5542309** | **1** | **5933** | **tags=88%, list=45%, signal=158%** |
| **INTERLEUKIN_2_PRODUCTION** | **INTERLEUKIN_2_PRODUCTION** | | **10** | **-0.45949978** | **-1.133003** | **0.3262519** | **0.5548009** | **1** | **2766** | **tags=40%, list=21%, signal=50%** |
| **REGULATION_OF_GROWTH** | **REGULATION_OF_GROWTH** | | **47** | **-0.31832263** | **-1.13053** | **0.2841678** | **0.55898625** | **1** | **3607** | **tags=38%, list=27%, signal=52%** |
| **MITOSIS** | **MITOSIS** |  | **66** | **-0.292185** | **-1.1238563** | **0.275** | **0.57387555** | **1** | **4884** | **tags=44%, list=37%, signal=69%** |
| **ENDOTHELIAL_CELL_MIGRATION** | **ENDOTHELIAL_CELL_MIGRATION** | | **11** | **-0.44604844** | **-1.1226333** | **0.3124019** | **0.5749439** | **1** | **3395** | **tags=45%, list=25%, signal=61%** |
| **GAMETE_GENERATION** | **GAMETE_GENERATION** | | **88** | **-0.27972296** | **-1.1173115** | **0.2763819** | **0.58622277** | **1** | **2945** | **tags=33%, list=22%, signal=42%** |
| **TUBE_MORPHOGENESIS** | **TUBE_MORPHOGENESIS** | | **13** | **-0.41918567** | **-1.1151329** | **0.32519084** | **0.5896698** | **1** | **1313** | **tags=23%, list=10%, signal=26%** |
| **TRANSCRIPTION_FROM_RNA_POLYMERASE_II_PROMOTER** | **TRANSCRIPTION_FROM_RNA_POLYMERASE_II_PROMOTER** | | **399** | **-0.2375037** | **-1.1146505** | **0.2010582** | **0.5887975** | **1** | **3200** | **tags=26%, list=24%, signal=34%** |
| **REGULATION_OF_PEPTIDYL_TYROSINE_PHOSPHORYLATION** | **REGULATION_OF_PEPTIDYL_TYROSINE_PHOSPHORYLATION** | | **13** | **-0.41774535** | **-1.114552** | **0.3207262** | **0.58704454** | **1** | **4104** | **tags=38%, list=31%, signal=56%** |
| **DEFENSE_RESPONSE_TO_VIRUS** | **DEFENSE_RESPONSE_TO_VIRUS** | | **10** | **-0.45403686** | **-1.1121367** | **0.32018927** | **0.5912146** | **1** | **758** | **tags=20%, list=6%, signal=21%** |
| **BRAIN_DEVELOPMENT** | **BRAIN_DEVELOPMENT** | | **35** | **-0.3341627** | **-1.1102834** | **0.30869564** | **0.593867** | **1** | **2793** | **tags=34%, list=21%, signal=43%** |
| **BONE_REMODELING** | **BONE_REMODELING** | | **27** | **-0.3488496** | **-1.1093684** | **0.3266187** | **0.5941459** | **1** | **3826** | **tags=41%, list=29%, signal=57%** |
| **GROWTH** | **GROWTH** |  | **56** | **-0.30105302** | **-1.1085048** | **0.31878307** | **0.59444505** | **1** | **3607** | **tags=38%, list=27%, signal=51%** |
| **CASPASE_ACTIVATION** | **CASPASE_ACTIVATION** | | **24** | **-0.36177078** | **-1.1039871** | **0.3230994** | **0.6045948** | **1** | **1751** | **tags=29%, list=13%, signal=34%** |
| **NEGATIVE_REGULATION_OF_BIOLOGICAL_PROCESS** | **NEGATIVE_REGULATION_OF_BIOLOGICAL_PROCESS** | | **563** | **-0.22924629** | **-1.1021785** | **0.20349434** | **0.60740006** | **1** | **3201** | **tags=26%, list=24%, signal=33%** |
| **NEGATIVE_REGULATION_OF_TRANSCRIPTION** | **NEGATIVE_REGULATION_OF_TRANSCRIPTION** | | **148** | **-0.25758147** | **-1.1018678** | **0.2815534** | **0.6061469** | **1** | **4140** | **tags=36%, list=31%, signal=51%** |
| **CENTROSOME_ORGANIZATION_AND_BIOGENESIS** | **CENTROSOME_ORGANIZATION_AND_BIOGENESIS** | | **11** | **-0.44443262** | **-1.1013637** | **0.34923077** | **0.6054818** | **1** | **3287** | **tags=45%, list=25%, signal=60%** |
| **MEIOTIC_RECOMBINATION** | **MEIOTIC_RECOMBINATION** | | **14** | **-0.4047128** | **-1.0995996** | **0.3323124** | **0.60811275** | **1** | **740** | **tags=14%, list=6%, signal=15%** |
| **GENE_SILENCING** | **GENE_SILENCING** | | **9** | **-0.45616546** | **-1.0981213** | **0.3429487** | **0.609772** | **1** | **3973** | **tags=56%, list=30%, signal=79%** |
| **CYCLIC_NUCLEOTIDE_METABOLIC_PROCESS** | **CYCLIC_NUCLEOTIDE_METABOLIC_PROCESS** | | **9** | **-0.4604557** | **-1.0974226** | **0.3524721** | **0.609509** | **1** | **4865** | **tags=56%, list=37%, signal=87%** |
| **TUBE_DEVELOPMENT** | **TUBE_DEVELOPMENT** | | **15** | **-0.39459753** | **-1.0961533** | **0.3414264** | **0.6107043** | **1** | **1313** | **tags=20%, list=10%, signal=22%** |
| **PROTEIN_AMINO_ACID_O_LINKED_GLYCOSYLATION** | **PROTEIN_AMINO_ACID_O_LINKED_GLYCOSYLATION** | | **18** | **-0.37736145** | **-1.0859013** | **0.3476923** | **0.6356423** | **1** | **4769** | **tags=44%, list=36%, signal=69%** |
| **CARBOHYDRATE_METABOLIC_PROCESS** | **CARBOHYDRATE_METABOLIC_PROCESS** | | **150** | **-0.25260472** | **-1.0854558** | **0.3110048** | **0.63468206** | **1** | **4173** | **tags=38%, list=31%, signal=55%** |
| **VASCULATURE_DEVELOPMENT** | **VASCULATURE_DEVELOPMENT** | | **46** | **-0.30331576** | **-1.0852025** | **0.3402778** | **0.6332699** | **1** | **945** | **tags=17%, list=7%, signal=19%** |
| **SMOOTH_MUSCLE_CONTRACTION_GO_0006939** | **SMOOTH_MUSCLE_CONTRACTION_GO_0006939** | | **11** | **-0.43165898** | **-1.0838846** | **0.36949685** | **0.63482493** | **1** | **3488** | **tags=36%, list=26%, signal=49%** |
| **ACTIVATION_OF_MAPK_ACTIVITY** | **ACTIVATION_OF_MAPK_ACTIVITY** | | **33** | **-0.32518473** | **-1.0834407** | **0.36075035** | **0.6338055** | **1** | **4075** | **tags=45%, list=31%, signal=65%** |
| **RHYTHMIC_PROCESS** | **RHYTHMIC_PROCESS** | | **22** | **-0.35812253** | **-1.0791389** | **0.35311145** | **0.64304227** | **1** | **1318** | **tags=23%, list=10%, signal=25%** |
| **POSITIVE_REGULATION_OF_TRANSPORT** | **POSITIVE_REGULATION_OF_TRANSPORT** | | **18** | **-0.37727934** | **-1.0776685** | **0.36090225** | **0.64495057** | **1** | **2749** | **tags=33%, list=21%, signal=42%** |
| **GENERATION_OF_NEURONS** | **GENERATION_OF_NEURONS** | | **66** | **-0.28198358** | **-1.0756751** | **0.34620336** | **0.64805585** | **1** | **2057** | **tags=26%, list=15%, signal=30%** |
| **VACUOLE_ORGANIZATION_AND_BIOGENESIS** | **VACUOLE_ORGANIZATION_AND_BIOGENESIS** | | **12** | **-0.41265678** | **-1.0745674** | **0.3894081** | **0.64890844** | **1** | **4781** | **tags=58%, list=36%, signal=91%** |
| **PROTEIN_TETRAMERIZATION** | **PROTEIN_TETRAMERIZATION** | | **13** | **-0.40884748** | **-1.0728079** | **0.36834094** | **0.65120935** | **1** | **1292** | **tags=23%, list=10%, signal=26%** |
| **OLIGOSACCHARIDE_METABOLIC_PROCESS** | **OLIGOSACCHARIDE_METABOLIC_PROCESS** | | **10** | **-0.44242486** | **-1.0715277** | **0.37558687** | **0.6524432** | **1** | **2273** | **tags=40%, list=17%, signal=48%** |
| **REGULATION_OF_TRANSCRIPTION_FROM_RNA_POLYMERASE_II_PROMOTER** | **REGULATION_OF_TRANSCRIPTION_FROM_RNA_POLYMERASE_II_PROMOTER** | | **246** | **-0.2349642** | **-1.0705861** | **0.32694477** | **0.6526346** | **1** | **3236** | **tags=26%, list=24%, signal=34%** |
| **CARBOHYDRATE_BIOSYNTHETIC_PROCESS** | **CARBOHYDRATE_BIOSYNTHETIC_PROCESS** | | **34** | **-0.3148061** | **-1.0704381** | **0.38450703** | **0.65097314** | **1** | **2239** | **tags=29%, list=17%, signal=35%** |
| **REGULATION_OF_GENE_EXPRESSION** | **REGULATION_OF_GENE_EXPRESSION** | | **532** | **-0.22464496** | **-1.0703882** | **0.28050053** | **0.6490401** | **1** | **3236** | **tags=26%, list=24%, signal=32%** |
| **NEGATIVE_REGULATION_OF_CELL_PROLIFERATION** | **NEGATIVE_REGULATION_OF_CELL_PROLIFERATION** | | **138** | **-0.25045446** | **-1.0701544** | **0.33293557** | **0.6475957** | **1** | **3274** | **tags=28%, list=25%, signal=37%** |
| **NEGATIVE_REGULATION_OF_TRANSCRIPTION_DNA_DEPENDENT** | **NEGATIVE_REGULATION_OF_TRANSCRIPTION_DNA_DEPENDENT** | | **103** | **-0.25813043** | **-1.0662013** | **0.32961586** | **0.6561691** | **1** | **3429** | **tags=29%, list=26%, signal=39%** |
| **REGULATION_OF_CYCLIN_DEPENDENT_PROTEIN_KINASE_ACTIVITY** | **REGULATION_OF_CYCLIN_DEPENDENT_PROTEIN_KINASE_ACTIVITY** | | **39** | **-0.31383523** | **-1.0623627** | **0.35164836** | **0.66447014** | **1** | **5122** | **tags=46%, list=38%, signal=75%** |
| **POSITIVE_REGULATION_OF_PHOSPHORYLATION** | **POSITIVE_REGULATION_OF_PHOSPHORYLATION** | | **19** | **-0.3675911** | **-1.0621622** | **0.3804511** | **0.66290003** | **1** | **4464** | **tags=42%, list=34%, signal=63%** |
| **NEGATIVE_REGULATION_OF_RNA_METABOLIC_PROCESS** | **NEGATIVE_REGULATION_OF_RNA_METABOLIC_PROCESS** | | **103** | **-0.25813043** | **-1.0591882** | **0.37157756** | **0.66882074** | **1** | **3429** | **tags=29%, list=26%, signal=39%** |
| **NEGATIVE_REGULATION_OF_CELLULAR_PROCESS** | **NEGATIVE_REGULATION_OF_CELLULAR_PROCESS** | | **538** | **-0.22248317** | **-1.0582521** | **0.317757** | **0.6692106** | **1** | **3201** | **tags=26%, list=24%, signal=32%** |
| **LIPID_TRANSPORT** | **LIPID_TRANSPORT** | | **27** | **-0.3287063** | **-1.0578179** | **0.3748212** | **0.6683278** | **1** | **1881** | **tags=30%, list=14%, signal=34%** |
| **CELL_MIGRATION** | **CELL_MIGRATION** | | **82** | **-0.27145773** | **-1.0554932** | **0.37323037** | **0.67240363** | **1** | **3041** | **tags=29%, list=23%, signal=38%** |
| **PROTEIN_EXPORT_FROM_NUCLEUS** | **PROTEIN_EXPORT_FROM_NUCLEUS** | | **8** | **-0.45679116** | **-1.0542085** | **0.41185898** | **0.67362463** | **1** | **113** | **tags=13%, list=1%, signal=13%** |
| **CELL_CELL_ADHESION** | **CELL_CELL_ADHESION** | | **69** | **-0.2755149** | **-1.0540556** | **0.3671562** | **0.6719438** | **1** | **3460** | **tags=35%, list=26%, signal=47%** |
| **RESPONSE_TO_NUTRIENT_LEVELS** | **RESPONSE_TO_NUTRIENT_LEVELS** | | **25** | **-0.33264053** | **-1.0521303** | **0.40088105** | **0.67495173** | **1** | **2147** | **tags=24%, list=16%, signal=29%** |
| **POSITIVE_REGULATION_OF_CELL_CYCLE** | **POSITIVE_REGULATION_OF_CELL_CYCLE** | | **15** | **-0.38231802** | **-1.0509298** | **0.4064915** | **0.67600673** | **1** | **3555** | **tags=40%, list=27%, signal=54%** |
| **STEROID_BIOSYNTHETIC_PROCESS** | **STEROID_BIOSYNTHETIC_PROCESS** | | **22** | **-0.34493017** | **-1.0503134** | **0.40207714** | **0.6754663** | **1** | **4202** | **tags=50%, list=32%, signal=73%** |
| **LEUKOCYTE_MIGRATION** | **LEUKOCYTE_MIGRATION** | | **14** | **-0.38708618** | **-1.049571** | **0.43171805** | **0.6754593** | **1** | **873** | **tags=14%, list=7%, signal=15%** |
| **SENSORY_PERCEPTION_OF_TASTE** | **SENSORY_PERCEPTION_OF_TASTE** | | **5** | **-0.5318632** | **-1.043951** | **0.43361345** | **0.6882559** | **1** | **2969** | **tags=40%, list=22%, signal=51%** |
| **PHOSPHOLIPID_METABOLIC_PROCESS** | **PHOSPHOLIPID_METABOLIC_PROCESS** | | **59** | **-0.27948245** | **-1.0414989** | **0.39487872** | **0.6928106** | **1** | **2376** | **tags=24%, list=18%, signal=29%** |
| **NEGATIVE_REGULATION_OF_CATALYTIC_ACTIVITY** | **NEGATIVE_REGULATION_OF_CATALYTIC_ACTIVITY** | | **62** | **-0.27527142** | **-1.040848** | **0.40957448** | **0.692523** | **1** | **4855** | **tags=45%, list=36%, signal=71%** |
| **INTERACTION_WITH_HOST** | **INTERACTION_WITH_HOST** | | **15** | **-0.37951034** | **-1.0406768** | **0.42121685** | **0.6909356** | **1** | **1839** | **tags=27%, list=14%, signal=31%** |
| **REGULATION_OF_METABOLIC_PROCESS** | **REGULATION_OF_METABOLIC_PROCESS** | | **634** | **-0.21636821** | **-1.0395198** | **0.3587629** | **0.6919936** | **1** | **3212** | **tags=25%, list=24%, signal=31%** |
| **POSITIVE_REGULATION_OF_RESPONSE_TO_STIMULUS** | **POSITIVE_REGULATION_OF_RESPONSE_TO_STIMULUS** | | **33** | **-0.31437904** | **-1.0381273** | **0.4138929** | **0.6935411** | **1** | **2772** | **tags=30%, list=21%, signal=38%** |
| **CATABOLIC_PROCESS** | **CATABOLIC_PROCESS** | | **193** | **-0.23241161** | **-1.0373873** | **0.39580908** | **0.6933226** | **1** | **2117** | **tags=20%, list=16%, signal=24%** |
| **POSITIVE_REGULATION_OF_CELL_MIGRATION** | **POSITIVE_REGULATION_OF_CELL_MIGRATION** | | **8** | **-0.44475394** | **-1.034857** | **0.44821733** | **0.6980336** | **1** | **2931** | **tags=38%, list=22%, signal=48%** |
| **TRANSCRIPTION_DNA_DEPENDENT** | **TRANSCRIPTION_DNA_DEPENDENT** | | **538** | **-0.21416982** | **-1.0337507** | **0.3952282** | **0.69875836** | **1** | **3236** | **tags=25%, list=24%, signal=31%** |
| **B_CELL_DIFFERENTIATION** | **B_CELL_DIFFERENTIATION** | | **10** | **-0.4153592** | **-1.0330209** | **0.4347158** | **0.698665** | **1** | **3162** | **tags=40%, list=24%, signal=52%** |
| **REGULATION_OF_PROTEIN_METABOLIC_PROCESS** | **REGULATION_OF_PROTEIN_METABOLIC_PROCESS** | | **134** | **-0.24253508** | **-1.0322227** | **0.4091456** | **0.6987884** | **1** | **3201** | **tags=27%, list=24%, signal=35%** |
| **REGULATION_OF_CELL_DIFFERENTIATION** | **REGULATION_OF_CELL_DIFFERENTIATION** | | **45** | **-0.29592288** | **-1.0301118** | **0.4132581** | **0.70249397** | **1** | **2299** | **tags=27%, list=17%, signal=32%** |
| **REGULATION_OF_CELLULAR_METABOLIC_PROCESS** | **REGULATION_OF_CELLULAR_METABOLIC_PROCESS** | | **624** | **-0.21305543** | **-1.0291574** | **0.4018595** | **0.70288974** | **1** | **3212** | **tags=25%, list=24%, signal=31%** |
| **HOMEOSTASIS_OF_NUMBER_OF_CELLS** | **HOMEOSTASIS_OF_NUMBER_OF_CELLS** | | **19** | **-0.34697047** | **-1.0286862** | **0.4323144** | **0.7021473** | **1** | **3162** | **tags=32%, list=24%, signal=41%** |
| **REGULATION_OF_NUCLEOBASENUCLEOSIDENUCLEOTIDE_AND_NUCLEIC_ACID_METABOLIC_PROCESS** | **REGULATION_OF_NUCLEOBASENUCLEOSIDENUCLEOTIDE_AND_NUCLEIC_ACID_METABOLIC_PROCESS** | | **495** | **-0.21564908** | **-1.026492** | **0.4105263** | **0.70585567** | **1** | **3236** | **tags=25%, list=24%, signal=32%** |
| **MEIOSIS_I** | **MEIOSIS_I** |  | **16** | **-0.3687862** | **-1.0232426** | **0.44364163** | **0.7122584** | **1** | **1412** | **tags=19%, list=11%, signal=21%** |
| **LEUKOCYTE_CHEMOTAXIS** | **LEUKOCYTE_CHEMOTAXIS** | | **12** | **-0.39778265** | **-1.0193685** | **0.42056075** | **0.7201732** | **1** | **873** | **tags=17%, list=7%, signal=18%** |
| **CALCIUM_ION_TRANSPORT** | **CALCIUM_ION_TRANSPORT** | | **23** | **-0.33514407** | **-1.0189493** | **0.45414847** | **0.7192527** | **1** | **4663** | **tags=52%, list=35%, signal=80%** |
| **RESPONSE_TO_ABIOTIC_STIMULUS** | **RESPONSE_TO_ABIOTIC_STIMULUS** | | **79** | **-0.26076585** | **-1.018914** | **0.44916344** | **0.71729285** | **1** | **3606** | **tags=33%, list=27%, signal=45%** |
| **RNA_BIOSYNTHETIC_PROCESS** | **RNA_BIOSYNTHETIC_PROCESS** | | **539** | **-0.21376283** | **-1.0184153** | **0.43343654** | **0.7164453** | **1** | **3236** | **tags=25%, list=24%, signal=31%** |
| **POSITIVE_REGULATION_OF_CELLULAR_PROTEIN_METABOLIC_PROCESS** | **POSITIVE_REGULATION_OF_CELLULAR_PROTEIN_METABOLIC_PROCESS** | | **59** | **-0.27187905** | **-1.0181401** | **0.4359313** | **0.71521455** | **1** | **2772** | **tags=29%, list=21%, signal=36%** |
| **CELLULAR_RESPONSE_TO_NUTRIENT_LEVELS** | **CELLULAR_RESPONSE_TO_NUTRIENT_LEVELS** | | **7** | **-0.46437186** | **-1.0177007** | **0.45561138** | **0.7141796** | **1** | **5933** | **tags=86%, list=45%, signal=154%** |
| **REGULATION_OF_TRANSCRIPTION** | **REGULATION_OF_TRANSCRIPTION** | | **456** | **-0.21598373** | **-1.0166336** | **0.45058447** | **0.7147589** | **1** | **3236** | **tags=25%, list=24%, signal=32%** |
| **NEURITE_DEVELOPMENT** | **NEURITE_DEVELOPMENT** | | **43** | **-0.29177323** | **-1.0165309** | **0.43157893** | **0.71300703** | **1** | **2000** | **tags=26%, list=15%, signal=30%** |
| **TRANSCRIPTION** | **TRANSCRIPTION** | | **627** | **-0.20896664** | **-1.013463** | **0.4614594** | **0.71899813** | **1** | **3212** | **tags=24%, list=24%, signal=30%** |
| **POSITIVE_REGULATION_OF_PHOSPHATE_METABOLIC_PROCESS** | **POSITIVE_REGULATION_OF_PHOSPHATE_METABOLIC_PROCESS** | | **21** | **-0.3329206** | **-1.00702** | **0.477208** | **0.73339313** | **1** | **4464** | **tags=38%, list=34%, signal=57%** |
| **NEURON_DIFFERENTIATION** | **NEURON_DIFFERENTIATION** | | **59** | **-0.27318424** | **-1.0041937** | **0.47567567** | **0.7388251** | **1** | **2057** | **tags=25%, list=15%, signal=30%** |
| **MUSCLE_DEVELOPMENT** | **MUSCLE_DEVELOPMENT** | | **85** | **-0.25325704** | **-1.0028766** | **0.4839125** | **0.740079** | **1** | **3622** | **tags=32%, list=27%, signal=43%** |
| **CELLULAR_CATABOLIC_PROCESS** | **CELLULAR_CATABOLIC_PROCESS** | | **182** | **-0.22687633** | **-1.0006641** | **0.47086248** | **0.7438536** | **1** | **2117** | **tags=20%, list=16%, signal=24%** |
| **EMBRYO_IMPLANTATION** | **EMBRYO_IMPLANTATION** | | **9** | **-0.42886123** | **-0.9981363** | **0.48032787** | **0.74851274** | **1** | **1472** | **tags=22%, list=11%, signal=25%** |
| **PHOSPHOLIPASE_C_ACTIVATION** | **PHOSPHOLIPASE_C_ACTIVATION** | | **11** | **-0.3955572** | **-0.9972901** | **0.46515396** | **0.74860793** | **1** | **2931** | **tags=27%, list=22%, signal=35%** |
| **CELLULAR_MORPHOGENESIS_DURING_DIFFERENTIATION** | **CELLULAR_MORPHOGENESIS_DURING_DIFFERENTIATION** | | **40** | **-0.28594974** | **-0.9970219** | **0.47534245** | **0.7471742** | **1** | **2000** | **tags=25%, list=15%, signal=29%** |
| **POSITIVE_REGULATION_OF_PROTEIN_METABOLIC_PROCESS** | **POSITIVE_REGULATION_OF_PROTEIN_METABOLIC_PROCESS** | | **61** | **-0.26369014** | **-0.9960187** | **0.47207448** | **0.74779695** | **1** | **2772** | **tags=28%, list=21%, signal=35%** |
| **INACTIVATION_OF_MAPK_ACTIVITY** | **INACTIVATION_OF_MAPK_ACTIVITY** | | **12** | **-0.3805027** | **-0.995923** | **0.45383412** | **0.7459881** | **1** | **2093** | **tags=33%, list=16%, signal=40%** |
| **REGULATION_OF_RESPONSE_TO_EXTERNAL_STIMULUS** | **REGULATION_OF_RESPONSE_TO_EXTERNAL_STIMULUS** | | **12** | **-0.3887488** | **-0.992686** | **0.46855345** | **0.7524255** | **1** | **1318** | **tags=25%, list=10%, signal=28%** |
| **RESPONSE_TO_STRESS** | **RESPONSE_TO_STRESS** | | **444** | **-0.20878375** | **-0.9918139** | **0.5036726** | **0.7526022** | **1** | **3774** | **tags=28%, list=28%, signal=38%** |
| **REGULATION_OF_TRANSCRIPTIONDNA_DEPENDENT** | **REGULATION_OF_TRANSCRIPTIONDNA_DEPENDENT** | | **379** | **-0.21199937** | **-0.9916601** | **0.51182795** | **0.75096214** | **1** | **3236** | **tags=24%, list=24%, signal=31%** |
| **NEUROGENESIS** | **NEUROGENESIS** | | **76** | **-0.25538749** | **-0.99057144** | **0.4774536** | **0.75172657** | **1** | **3560** | **tags=37%, list=27%, signal=50%** |
| **REGULATION_OF_TRANSPORT** | **REGULATION_OF_TRANSPORT** | | **55** | **-0.26680228** | **-0.9898738** | **0.5019815** | **0.75133306** | **1** | **4408** | **tags=40%, list=33%, signal=60%** |
| **DNA_METABOLIC_PROCESS** | **DNA_METABOLIC_PROCESS** | | **221** | **-0.22251861** | **-0.98964816** | **0.5045045** | **0.74976593** | **1** | **3712** | **tags=30%, list=28%, signal=41%** |
| **POSITIVE_REGULATION_OF_CELL_ADHESION** | **POSITIVE_REGULATION_OF_CELL_ADHESION** | | **13** | **-0.3682428** | **-0.9885027** | **0.49304482** | **0.75074047** | **1** | **2976** | **tags=38%, list=22%, signal=49%** |
| **RESPONSE_TO_HYPOXIA** | **RESPONSE_TO_HYPOXIA** | | **25** | **-0.3149475** | **-0.98745257** | **0.4682081** | **0.7515837** | **1** | **2594** | **tags=28%, list=19%, signal=35%** |
| **PROTEIN_TARGETING_TO_MITOCHONDRION** | **PROTEIN_TARGETING_TO_MITOCHONDRION** | | **9** | **-0.4190838** | **-0.98698014** | **0.48217055** | **0.75074846** | **1** | **1823** | **tags=33%, list=14%, signal=39%** |
| **EPITHELIAL_TO_MESENCHYMAL_TRANSITION** | **EPITHELIAL_TO_MESENCHYMAL_TRANSITION** | | **9** | **-0.41133142** | **-0.98406374** | **0.49763033** | **0.75601244** | **1** | **1561** | **tags=22%, list=12%, signal=25%** |
| **INTERFERON_GAMMA_PRODUCTION** | **INTERFERON_GAMMA_PRODUCTION** | | **11** | **-0.38681534** | **-0.98205477** | **0.48382127** | **0.7589542** | **1** | **3162** | **tags=36%, list=24%, signal=48%** |
| **G1_S_TRANSITION_OF_MITOTIC_CELL_CYCLE** | **G1_S_TRANSITION_OF_MITOTIC_CELL_CYCLE** | | **24** | **-0.3113842** | **-0.98176247** | **0.49928057** | **0.75764024** | **1** | **3162** | **tags=25%, list=24%, signal=33%** |
| **GLYCOPROTEIN_BIOSYNTHETIC_PROCESS** | **GLYCOPROTEIN_BIOSYNTHETIC_PROCESS** | | **65** | **-0.25911924** | **-0.974728** | **0.51409394** | **0.7729918** | **1** | **3929** | **tags=37%, list=29%, signal=52%** |
| **REGULATION_OF_INTERFERON_GAMMA_BIOSYNTHETIC_PROCESS** | **REGULATION_OF_INTERFERON_GAMMA_BIOSYNTHETIC_PROCESS** | | **8** | **-0.42017302** | **-0.97381365** | **0.49275362** | **0.7730169** | **1** | **3162** | **tags=38%, list=24%, signal=49%** |
| **DEVELOPMENT_OF_PRIMARY_SEXUAL_CHARACTERISTICS** | **DEVELOPMENT_OF_PRIMARY_SEXUAL_CHARACTERISTICS** | | **25** | **-0.3136935** | **-0.9728435** | **0.5241581** | **0.7733611** | **1** | **2907** | **tags=36%, list=22%, signal=46%** |
| **INNATE_IMMUNE_RESPONSE** | **INNATE_IMMUNE_RESPONSE** | | **17** | **-0.3350988** | **-0.9720967** | **0.49608764** | **0.773192** | **1** | **4446** | **tags=41%, list=33%, signal=62%** |
| **MONOCARBOXYLIC_ACID_TRANSPORT** | **MONOCARBOXYLIC_ACID_TRANSPORT** | | **10** | **-0.39337227** | **-0.9720364** | **0.5007752** | **0.7712867** | **1** | **5896** | **tags=70%, list=44%, signal=125%** |
| **NUCLEOTIDE_BIOSYNTHETIC_PROCESS** | **NUCLEOTIDE_BIOSYNTHETIC_PROCESS** | | **16** | **-0.35367736** | **-0.97181076** | **0.49237806** | **0.76978207** | **1** | **6117** | **tags=56%, list=46%, signal=104%** |
| **REGULATION_OF_RNA_METABOLIC_PROCESS** | **REGULATION_OF_RNA_METABOLIC_PROCESS** | | **384** | **-0.20602788** | **-0.97163147** | **0.568254** | **0.76824474** | **1** | **3236** | **tags=24%, list=24%, signal=31%** |
| **CELL_CYCLE_CHECKPOINT_GO_0000075** | **CELL_CYCLE_CHECKPOINT_GO_0000075** | | **42** | **-0.28457764** | **-0.9712391** | **0.5144033** | **0.7672083** | **1** | **4802** | **tags=48%, list=36%, signal=74%** |
| **POSITIVE_REGULATION_OF_CASPASE_ACTIVITY** | **POSITIVE_REGULATION_OF_CASPASE_ACTIVITY** | | **26** | **-0.3054677** | **-0.9706155** | **0.506647** | **0.7666851** | **1** | **1751** | **tags=27%, list=13%, signal=31%** |
| **CHROMOSOME_SEGREGATION** | **CHROMOSOME_SEGREGATION** | | **24** | **-0.3125114** | **-0.97045565** | **0.5165945** | **0.76505595** | **1** | **1133** | **tags=17%, list=9%, signal=18%** |
| **PEPTIDYL_TYROSINE_MODIFICATION** | **PEPTIDYL_TYROSINE_MODIFICATION** | | **23** | **-0.31464878** | **-0.96973413** | **0.491404** | **0.7649271** | **1** | **4165** | **tags=39%, list=31%, signal=57%** |
| **PROTEIN_HOMOOLIGOMERIZATION** | **PROTEIN_HOMOOLIGOMERIZATION** | | **17** | **-0.34401205** | **-0.9690632** | **0.505295** | **0.7645104** | **1** | **1389** | **tags=18%, list=10%, signal=20%** |
| **REGULATION_OF_ANATOMICAL_STRUCTURE_MORPHOGENESIS** | **REGULATION_OF_ANATOMICAL_STRUCTURE_MORPHOGENESIS** | | **18** | **-0.3410543** | **-0.96869797** | **0.5259842** | **0.76334554** | **1** | **1661** | **tags=22%, list=12%, signal=25%** |
| **POSITIVE_REGULATION_OF_PEPTIDYL_TYROSINE_PHOSPHORYLATION** | **POSITIVE_REGULATION_OF_PEPTIDYL_TYROSINE_PHOSPHORYLATION** | | **9** | **-0.40914735** | **-0.96833235** | **0.5024155** | **0.76226157** | **1** | **1856** | **tags=22%, list=14%, signal=26%** |
| **ANION_TRANSPORT** | **ANION_TRANSPORT** | | **27** | **-0.30251935** | **-0.9677997** | **0.52387846** | **0.76162696** | **1** | **1467** | **tags=22%, list=11%, signal=25%** |
| **NEGATIVE_REGULATION_OF_DNA_METABOLIC_PROCESS** | **NEGATIVE_REGULATION_OF_DNA_METABOLIC_PROCESS** | | **16** | **-0.3409859** | **-0.96728486** | **0.51891077** | **0.76093054** | **1** | **3041** | **tags=38%, list=23%, signal=49%** |
| **MITOCHONDRION_ORGANIZATION_AND_BIOGENESIS** | **MITOCHONDRION_ORGANIZATION_AND_BIOGENESIS** | | **43** | **-0.27532938** | **-0.96685934** | **0.52414775** | **0.760106** | **1** | **2347** | **tags=21%, list=18%, signal=25%** |
| **INTRACELLULAR_SIGNALING_CASCADE** | **INTRACELLULAR_SIGNALING_CASCADE** | | **555** | **-0.20124236** | **-0.9665165** | **0.60102564** | **0.75893176** | **1** | **3202** | **tags=25%, list=24%, signal=32%** |
| **NEURON_DEVELOPMENT** | **NEURON_DEVELOPMENT** | | **50** | **-0.26680177** | **-0.96557724** | **0.5074425** | **0.7592598** | **1** | **2000** | **tags=24%, list=15%, signal=28%** |
| **POSITIVE_REGULATION_OF_DEVELOPMENTAL_PROCESS** | **POSITIVE_REGULATION_OF_DEVELOPMENTAL_PROCESS** | | **186** | **-0.22144614** | **-0.9655616** | **0.54875284** | **0.75737995** | **1** | **1753** | **tags=17%, list=13%, signal=19%** |
| **SENSORY_ORGAN_DEVELOPMENT** | **SENSORY_ORGAN_DEVELOPMENT** | | **14** | **-0.35885525** | **-0.9653676** | **0.5098336** | **0.755892** | **1** | **417** | **tags=14%, list=3%, signal=15%** |
| **CYTOKINE_SECRETION** | **CYTOKINE_SECRETION** | | **10** | **-0.39577803** | **-0.96237755** | **0.50158733** | **0.76115537** | **1** | **3075** | **tags=50%, list=23%, signal=65%** |
| **INSULIN_RECEPTOR_SIGNALING_PATHWAY** | **INSULIN_RECEPTOR_SIGNALING_PATHWAY** | | **16** | **-0.3523937** | **-0.96203834** | **0.5271318** | **0.760076** | **1** | **268** | **tags=13%, list=2%, signal=13%** |
| **REGULATION_OF_ANGIOGENESIS** | **REGULATION_OF_ANGIOGENESIS** | | **23** | **-0.3099556** | **-0.96011734** | **0.5** | **0.7624683** | **1** | **1603** | **tags=22%, list=12%, signal=25%** |
| **REGULATION_OF_CELLULAR_PROTEIN_METABOLIC_PROCESS** | **REGULATION_OF_CELLULAR_PROTEIN_METABOLIC_PROCESS** | | **123** | **-0.22799943** | **-0.9559542** | **0.5683814** | **0.7707222** | **1** | **3162** | **tags=26%, list=24%, signal=34%** |
| **MEMBRANE_LIPID_BIOSYNTHETIC_PROCESS** | **MEMBRANE_LIPID_BIOSYNTHETIC_PROCESS** | | **35** | **-0.28283188** | **-0.95482343** | **0.5166426** | **0.77154016** | **1** | **2273** | **tags=23%, list=17%, signal=27%** |
| **SECRETORY_PATHWAY** | **SECRETORY_PATHWAY** | | **70** | **-0.24418783** | **-0.94752795** | **0.5529716** | **0.7865188** | **1** | **637** | **tags=10%, list=5%, signal=10%** |
| **REGULATION_OF_CYTOKINE_SECRETION** | **REGULATION_OF_CYTOKINE_SECRETION** | | **9** | **-0.4010241** | **-0.94622594** | **0.56692916** | **0.7877462** | **1** | **2544** | **tags=44%, list=19%, signal=55%** |
| **RESPONSE_TO_NUTRIENT** | **RESPONSE_TO_NUTRIENT** | | **16** | **-0.34154868** | **-0.9441407** | **0.546875** | **0.7906325** | **1** | **2147** | **tags=25%, list=16%, signal=30%** |
| **PERIPHERAL_NERVOUS_SYSTEM_DEVELOPMENT** | **PERIPHERAL_NERVOUS_SYSTEM_DEVELOPMENT** | | **12** | **-0.3609949** | **-0.94191045** | **0.5385852** | **0.7937524** | **1** | **3147** | **tags=42%, list=24%, signal=55%** |
| **MONOCARBOXYLIC_ACID_METABOLIC_PROCESS** | **MONOCARBOXYLIC_ACID_METABOLIC_PROCESS** | | **74** | **-0.24246661** | **-0.94018507** | **0.58643615** | **0.7956512** | **1** | **2265** | **tags=26%, list=17%, signal=31%** |
| **REGULATION_OF_LIPID_METABOLIC_PROCESS** | **REGULATION_OF_LIPID_METABOLIC_PROCESS** | | **11** | **-0.37344354** | **-0.9382223** | **0.55198777** | **0.7981375** | **1** | **1688** | **tags=27%, list=13%, signal=31%** |
| **NEGATIVE_REGULATION_OF_MAP_KINASE_ACTIVITY** | **NEGATIVE_REGULATION_OF_MAP_KINASE_ACTIVITY** | | **15** | **-0.34103405** | **-0.93745154** | **0.5538922** | **0.79785967** | **1** | **2093** | **tags=27%, list=16%, signal=32%** |
| **REGULATION_OF_IMMUNE_EFFECTOR_PROCESS** | **REGULATION_OF_IMMUNE_EFFECTOR_PROCESS** | | **13** | **-0.35275885** | **-0.9369949** | **0.5366242** | **0.79704213** | **1** | **2772** | **tags=31%, list=21%, signal=39%** |
| **NEGATIVE_REGULATION_OF_ANGIOGENESIS** | **NEGATIVE_REGULATION_OF_ANGIOGENESIS** | | **11** | **-0.36245528** | **-0.93262184** | **0.54967946** | **0.80503464** | **1** | **945** | **tags=27%, list=7%, signal=29%** |
| **NEGATIVE_REGULATION_OF_DNA_REPLICATION** | **NEGATIVE_REGULATION_OF_DNA_REPLICATION** | | **11** | **-0.36449814** | **-0.9302944** | **0.5388349** | **0.8084665** | **1** | **3041** | **tags=45%, list=23%, signal=59%** |
| **FEMALE_GAMETE_GENERATION** | **FEMALE_GAMETE_GENERATION** | | **15** | **-0.33791384** | **-0.92843646** | **0.545045** | **0.8107265** | **1** | **2442** | **tags=33%, list=18%, signal=41%** |
| **AMINO_SUGAR_METABOLIC_PROCESS** | **AMINO_SUGAR_METABOLIC_PROCESS** | | **15** | **-0.3351627** | **-0.92775476** | **0.56429654** | **0.81026304** | **1** | **1971** | **tags=33%, list=15%, signal=39%** |
| **REGULATION_OF_HYDROLASE_ACTIVITY** | **REGULATION_OF_HYDROLASE_ACTIVITY** | | **63** | **-0.24607769** | **-0.9204529** | **0.59244794** | **0.8249055** | **1** | **1232** | **tags=14%, list=9%, signal=16%** |
| **NEGATIVE_REGULATION_OF_IMMUNE_SYSTEM_PROCESS** | **NEGATIVE_REGULATION_OF_IMMUNE_SYSTEM_PROCESS** | | **13** | **-0.3590096** | **-0.9203447** | **0.5731707** | **0.82318544** | **1** | **5797** | **tags=62%, list=44%, signal=109%** |
| **REGULATION_OF_CATABOLIC_PROCESS** | **REGULATION_OF_CATABOLIC_PROCESS** | | **15** | **-0.334221** | **-0.92034143** | **0.56042296** | **0.8212091** | **1** | **1688** | **tags=20%, list=13%, signal=23%** |
| **SKELETAL_DEVELOPMENT** | **SKELETAL_DEVELOPMENT** | | **91** | **-0.22984557** | **-0.9188285** | **0.63084704** | **0.82271** | **1** | **4865** | **tags=43%, list=37%, signal=67%** |
| **RESPONSE_TO_UV** | **RESPONSE_TO_UV** | | **22** | **-0.30467308** | **-0.91791993** | **0.5752608** | **0.82267207** | **1** | **3170** | **tags=36%, list=24%, signal=48%** |
| **PEPTIDYL_TYROSINE_PHOSPHORYLATION** | **PEPTIDYL_TYROSINE_PHOSPHORYLATION** | | **21** | **-0.30312717** | **-0.9176457** | **0.5800866** | **0.82135975** | **1** | **4165** | **tags=38%, list=31%, signal=55%** |
| **NEGATIVE_REGULATION_OF_HYDROLASE_ACTIVITY** | **NEGATIVE_REGULATION_OF_HYDROLASE_ACTIVITY** | | **14** | **-0.33438882** | **-0.9171903** | **0.5762195** | **0.8205136** | **1** | **693** | **tags=14%, list=5%, signal=15%** |
| **AMINO_ACID_METABOLIC_PROCESS** | **AMINO_ACID_METABOLIC_PROCESS** | | **72** | **-0.23838076** | **-0.91711855** | **0.6025641** | **0.8187436** | **1** | **5052** | **tags=51%, list=38%, signal=82%** |
| **SULFUR_COMPOUND_BIOSYNTHETIC_PROCESS** | **SULFUR_COMPOUND_BIOSYNTHETIC_PROCESS** | | **13** | **-0.3453451** | **-0.9147342** | **0.58942455** | **0.8220226** | **1** | **1720** | **tags=23%, list=13%, signal=26%** |
| **POSITIVE_REGULATION_OF_METABOLIC_PROCESS** | **POSITIVE_REGULATION_OF_METABOLIC_PROCESS** | | **182** | **-0.20812562** | **-0.91263515** | **0.65130025** | **0.8247777** | **1** | **3236** | **tags=27%, list=24%, signal=36%** |
| **REGULATION_OF_PROTEIN_SECRETION** | **REGULATION_OF_PROTEIN_SECRETION** | | **13** | **-0.34754765** | **-0.91094804** | **0.5943838** | **0.8264947** | **1** | **2544** | **tags=31%, list=19%, signal=38%** |
| **NEGATIVE_REGULATION_OF_CELL_DIFFERENTIATION** | **NEGATIVE_REGULATION_OF_CELL_DIFFERENTIATION** | | **23** | **-0.2994233** | **-0.909915** | **0.60921246** | **0.8268049** | **1** | **3162** | **tags=39%, list=24%, signal=51%** |
| **REGULATION_OF_INTRACELLULAR_TRANSPORT** | **REGULATION_OF_INTRACELLULAR_TRANSPORT** | | **21** | **-0.30040914** | **-0.9074755** | **0.6111111** | **0.8301513** | **1** | **4077** | **tags=38%, list=31%, signal=55%** |
| **TRANSMEMBRANE_RECEPTOR_PROTEIN_TYROSINE_KINASE_SIGNALING_PATHWAY** | **TRANSMEMBRANE_RECEPTOR_PROTEIN_TYROSINE_KINASE_SIGNALING_PATHWAY** | | **75** | **-0.23311573** | **-0.9066684** | **0.6453265** | **0.8299809** | **1** | **3377** | **tags=28%, list=25%, signal=37%** |
| **POSITIVE_REGULATION_OF_HYDROLASE_ACTIVITY** | **POSITIVE_REGULATION_OF_HYDROLASE_ACTIVITY** | | **44** | **-0.25683972** | **-0.90648115** | **0.62172776** | **0.8284914** | **1** | **1232** | **tags=16%, list=9%, signal=17%** |
| **CARBOHYDRATE_TRANSPORT** | **CARBOHYDRATE_TRANSPORT** | | **17** | **-0.32159638** | **-0.9046662** | **0.61290324** | **0.8305263** | **1** | **1785** | **tags=24%, list=13%, signal=27%** |
| **TYROSINE_PHOSPHORYLATION_OF_STAT_PROTEIN** | **TYROSINE_PHOSPHORYLATION_OF_STAT_PROTEIN** | | **9** | **-0.3748449** | **-0.9036597** | **0.5845411** | **0.8306795** | **1** | **3041** | **tags=33%, list=23%, signal=43%** |
| **REGULATION_OF_PHOSPHORYLATION** | **REGULATION_OF_PHOSPHORYLATION** | | **38** | **-0.2642314** | **-0.9036001** | **0.6211268** | **0.82888854** | **1** | **3372** | **tags=29%, list=25%, signal=39%** |
| **SECRETION_BY_CELL** | **SECRETION_BY_CELL** | | **92** | **-0.22254083** | **-0.9005973** | **0.6641791** | **0.8334492** | **1** | **2544** | **tags=20%, list=19%, signal=24%** |
| **REGULATION_OF_MITOSIS** | **REGULATION_OF_MITOSIS** | | **34** | **-0.26859513** | **-0.89984936** | **0.6122449** | **0.83315456** | **1** | **4741** | **tags=44%, list=36%, signal=68%** |
| **LYSOSOME_ORGANIZATION_AND_BIOGENESIS** | **LYSOSOME_ORGANIZATION_AND_BIOGENESIS** | | **11** | **-0.35560477** | **-0.89789444** | **0.61586314** | **0.8354769** | **1** | **4781** | **tags=55%, list=36%, signal=85%** |
| **INTERCELLULAR_JUNCTION_ASSEMBLY_AND_MAINTENANCE** | **INTERCELLULAR_JUNCTION_ASSEMBLY_AND_MAINTENANCE** | | **11** | **-0.35739344** | **-0.89423156** | **0.6012176** | **0.8413272** | **1** | **1808** | **tags=27%, list=14%, signal=32%** |
| **RNA_METABOLIC_PROCESS** | **RNA_METABOLIC_PROCESS** | | **683** | **-0.18447357** | **-0.8921314** | **0.8205912** | **0.84391576** | **1** | **3212** | **tags=23%, list=24%, signal=28%** |
| **RESPONSE_TO_TOXIN** | **RESPONSE_TO_TOXIN** | | **9** | **-0.372856** | **-0.8906221** | **0.6200318** | **0.84518725** | **1** | **3295** | **tags=44%, list=25%, signal=59%** |
| **ENZYME_LINKED_RECEPTOR_PROTEIN_SIGNALING_PATHWAY** | **ENZYME_LINKED_RECEPTOR_PROTEIN_SIGNALING_PATHWAY** | | **127** | **-0.21085207** | **-0.8903424** | **0.69570553** | **0.8437813** | **1** | **3037** | **tags=24%, list=23%, signal=31%** |
| **REGULATION_OF_VIRAL_REPRODUCTION** | **REGULATION_OF_VIRAL_REPRODUCTION** | | **10** | **-0.3636209** | **-0.8902596** | **0.6022013** | **0.84202987** | **1** | **758** | **tags=20%, list=6%, signal=21%** |
| **POSITIVE_REGULATION_OF_BIOLOGICAL_PROCESS** | **POSITIVE_REGULATION_OF_BIOLOGICAL_PROCESS** | | **570** | **-0.18333401** | **-0.88280535** | **0.81516093** | **0.8557435** | **1** | **3236** | **tags=23%, list=24%, signal=30%** |
| **MACROMOLECULE_BIOSYNTHETIC_PROCESS** | **MACROMOLECULE_BIOSYNTHETIC_PROCESS** | | **251** | **-0.19383** | **-0.8797472** | **0.745838** | **0.8599259** | **1** | **2299** | **tags=20%, list=17%, signal=24%** |
| **DNA_INTEGRITY_CHECKPOINT** | **DNA_INTEGRITY_CHECKPOINT** | | **19** | **-0.29993334** | **-0.87894607** | **0.6319759** | **0.85956633** | **1** | **3337** | **tags=37%, list=25%, signal=49%** |
| **CARBOXYLIC_ACID_METABOLIC_PROCESS** | **CARBOXYLIC_ACID_METABOLIC_PROCESS** | | **156** | **-0.20182276** | **-0.8782142** | **0.71893847** | **0.85917985** | **1** | **4229** | **tags=38%, list=32%, signal=55%** |
| **CELL_PROJECTION_BIOGENESIS** | **CELL_PROJECTION_BIOGENESIS** | | **19** | **-0.29942834** | **-0.8776655** | **0.6438953** | **0.8583706** | **1** | **850** | **tags=16%, list=6%, signal=17%** |
| **ACTIN_FILAMENT_BASED_MOVEMENT** | **ACTIN_FILAMENT_BASED_MOVEMENT** | | **10** | **-0.35894862** | **-0.8758171** | **0.62171054** | **0.86010426** | **1** | **580** | **tags=20%, list=4%, signal=21%** |
| **ORGANIC_ACID_METABOLIC_PROCESS** | **ORGANIC_ACID_METABOLIC_PROCESS** | | **158** | **-0.19936757** | **-0.87468666** | **0.7293844** | **0.8604038** | **1** | **5052** | **tags=47%, list=38%, signal=75%** |
| **PIGMENT_METABOLIC_PROCESS** | **PIGMENT_METABOLIC_PROCESS** | | **18** | **-0.30394226** | **-0.8719449** | **0.62814814** | **0.86393565** | **1** | **4750** | **tags=44%, list=36%, signal=69%** |
| **DNA_DAMAGE_CHECKPOINT** | **DNA_DAMAGE_CHECKPOINT** | | **16** | **-0.3074649** | **-0.87060845** | **0.652439** | **0.86446077** | **1** | **4722** | **tags=50%, list=35%, signal=77%** |
| **PEROXISOME_ORGANIZATION_AND_BIOGENESIS** | **PEROXISOME_ORGANIZATION_AND_BIOGENESIS** | | **15** | **-0.30874568** | **-0.87033975** | **0.65426356** | **0.8630784** | **1** | **1389** | **tags=13%, list=10%, signal=15%** |
| **PROTEIN_TARGETING** | **PROTEIN_TARGETING** | | **92** | **-0.21649103** | **-0.8691094** | **0.72866243** | **0.8636363** | **1** | **1838** | **tags=16%, list=14%, signal=19%** |
| **NEGATIVE_REGULATION_OF_TRANSCRIPTION_FACTOR_ACTIVITY** | **NEGATIVE_REGULATION_OF_TRANSCRIPTION_FACTOR_ACTIVITY** | | **12** | **-0.3337183** | **-0.86889946** | **0.6506024** | **0.8621911** | **1** | **3475** | **tags=33%, list=26%, signal=45%** |
| **INDUCTION_OF_APOPTOSIS_BY_EXTRACELLULAR_SIGNALS** | **INDUCTION_OF_APOPTOSIS_BY_EXTRACELLULAR_SIGNALS** | | **25** | **-0.28022364** | **-0.86807275** | **0.65552324** | **0.86191875** | **1** | **569** | **tags=12%, list=4%, signal=13%** |
| **ENDOTHELIAL_CELL_PROLIFERATION** | **ENDOTHELIAL_CELL_PROLIFERATION** | | **9** | **-0.36353445** | **-0.86728984** | **0.64154106** | **0.8615265** | **1** | **5168** | **tags=67%, list=39%, signal=109%** |
| **CELL_DEVELOPMENT** | **CELL_DEVELOPMENT** | | **498** | **-0.18177888** | **-0.86660546** | **0.8446298** | **0.8608229** | **1** | **3560** | **tags=27%, list=27%, signal=35%** |
| **KERATINOCYTE_DIFFERENTIATION** | **KERATINOCYTE_DIFFERENTIATION** | | **13** | **-0.32741582** | **-0.86378074** | **0.6438356** | **0.8644983** | **1** | **3280** | **tags=46%, list=25%, signal=61%** |
| **CELLULAR_MONOVALENT_INORGANIC_CATION_HOMEOSTASIS** | **CELLULAR_MONOVALENT_INORGANIC_CATION_HOMEOSTASIS** | | **10** | **-0.35435355** | **-0.8624414** | **0.646434** | **0.8651866** | **1** | **143** | **tags=10%, list=1%, signal=10%** |
| **NEGATIVE_REGULATION_OF_PHOSPHORYLATION** | **NEGATIVE_REGULATION_OF_PHOSPHORYLATION** | | **11** | **-0.34055778** | **-0.86081976** | **0.64649683** | **0.8665816** | **1** | **5342** | **tags=55%, list=40%, signal=91%** |
| **PROTEIN_IMPORT_INTO_NUCLEUS_TRANSLOCATION** | **PROTEIN_IMPORT_INTO_NUCLEUS_TRANSLOCATION** | | **11** | **-0.34179664** | **-0.8539873** | **0.6484751** | **0.8779037** | **1** | **2682** | **tags=27%, list=20%, signal=34%** |
| **REGULATION_OF_DNA_METABOLIC_PROCESS** | **REGULATION_OF_DNA_METABOLIC_PROCESS** | | **37** | **-0.25420642** | **-0.8538492** | **0.6878531** | **0.87625426** | **1** | **3041** | **tags=30%, list=23%, signal=38%** |
| **PATTERN_SPECIFICATION_PROCESS** | **PATTERN_SPECIFICATION_PROCESS** | | **27** | **-0.27075988** | **-0.85272187** | **0.68550724** | **0.8765483** | **1** | **1259** | **tags=19%, list=9%, signal=20%** |
| **INTERCELLULAR_JUNCTION_ASSEMBLY** | **INTERCELLULAR_JUNCTION_ASSEMBLY** | | **9** | **-0.3629828** | **-0.8525049** | **0.67601246** | **0.87505627** | **1** | **1808** | **tags=33%, list=14%, signal=39%** |
| **AMINE_METABOLIC_PROCESS** | **AMINE_METABOLIC_PROCESS** | | **125** | **-0.2005991** | **-0.84681624** | **0.7748503** | **0.8837857** | **1** | **4229** | **tags=38%, list=32%, signal=56%** |
| **CELLULAR_PROTEIN_COMPLEX_ASSEMBLY** | **CELLULAR_PROTEIN_COMPLEX_ASSEMBLY** | | **28** | **-0.26158366** | **-0.84639466** | **0.71428573** | **0.88269174** | **1** | **1592** | **tags=14%, list=12%, signal=16%** |
| **REGULATION_OF_DEVELOPMENTAL_PROCESS** | **REGULATION_OF_DEVELOPMENTAL_PROCESS** | | **369** | **-0.18096004** | **-0.84480983** | **0.8690096** | **0.8839535** | **1** | **3201** | **tags=23%, list=24%, signal=30%** |
| **SUPEROXIDE_METABOLIC_PROCESS** | **SUPEROXIDE_METABOLIC_PROCESS** | | **8** | **-0.37078726** | **-0.8444696** | **0.6650083** | **0.8826908** | **1** | **5593** | **tags=75%, list=42%, signal=129%** |
| **PROTEIN_AMINO_ACID_DEPHOSPHORYLATION** | **PROTEIN_AMINO_ACID_DEPHOSPHORYLATION** | | **60** | **-0.22610942** | **-0.84392786** | **0.7252011** | **0.8817836** | **1** | **2137** | **tags=18%, list=16%, signal=22%** |
| **ALCOHOL_METABOLIC_PROCESS** | **ALCOHOL_METABOLIC_PROCESS** | | **81** | **-0.21238475** | **-0.84316576** | **0.7546419** | **0.88131124** | **1** | **1920** | **tags=17%, list=14%, signal=20%** |
| **REGULATION_OF_CELLULAR_PH** | **REGULATION_OF_CELLULAR_PH** | | **9** | **-0.35499734** | **-0.84316266** | **0.6795491** | **0.8794284** | **1** | **143** | **tags=11%, list=1%, signal=11%** |
| **POSITIVE_REGULATION_OF_CELLULAR_METABOLIC_PROCESS** | **POSITIVE_REGULATION_OF_CELLULAR_METABOLIC_PROCESS** | | **177** | **-0.1934346** | **-0.84002066** | **0.82272726** | **0.8834197** | **1** | **3236** | **tags=27%, list=24%, signal=35%** |
| **MEMBRANE_ORGANIZATION_AND_BIOGENESIS** | **MEMBRANE_ORGANIZATION_AND_BIOGENESIS** | | **117** | **-0.20069279** | **-0.837394** | **0.7894737** | **0.886342** | **1** | **2082** | **tags=17%, list=16%, signal=20%** |
| **NEGATIVE_REGULATION_OF_PHOSPHATE_METABOLIC_PROCESS** | **NEGATIVE_REGULATION_OF_PHOSPHATE_METABOLIC_PROCESS** | | **12** | **-0.31803873** | **-0.8341894** | **0.68292683** | **0.8902345** | **1** | **5342** | **tags=50%, list=40%, signal=83%** |
| **REGULATION_OF_ACTION_POTENTIAL** | **REGULATION_OF_ACTION_POTENTIAL** | | **16** | **-0.30457577** | **-0.83414084** | **0.68575853** | **0.8884189** | **1** | **1472** | **tags=19%, list=11%, signal=21%** |
| **CYTOPLASM_ORGANIZATION_AND_BIOGENESIS** | **CYTOPLASM_ORGANIZATION_AND_BIOGENESIS** | | **13** | **-0.31227642** | **-0.8280499** | **0.6885759** | **0.89733726** | **1** | **1808** | **tags=23%, list=14%, signal=27%** |
| **TRANSITION_METAL_ION_TRANSPORT** | **TRANSITION_METAL_ION_TRANSPORT** | | **10** | **-0.33512056** | **-0.8270507** | **0.68659127** | **0.89710295** | **1** | **131** | **tags=10%, list=1%, signal=10%** |
| **ENERGY_RESERVE_METABOLIC_PROCESS** | **ENERGY_RESERVE_METABOLIC_PROCESS** | | **15** | **-0.29405966** | **-0.82670534** | **0.69736844** | **0.895851** | **1** | **4070** | **tags=40%, list=31%, signal=58%** |
| **NEGATIVE_REGULATION_OF_DNA_BINDING** | **NEGATIVE_REGULATION_OF_DNA_BINDING** | | **14** | **-0.30189213** | **-0.81585705** | **0.71428573** | **0.91283417** | **1** | **5705** | **tags=57%, list=43%, signal=100%** |
| **NITROGEN_COMPOUND_METABOLIC_PROCESS** | **NITROGEN_COMPOUND_METABOLIC_PROCESS** | | **137** | **-0.19171257** | **-0.81573886** | **0.84800965** | **0.91113144** | **1** | **4363** | **tags=39%, list=33%, signal=57%** |
| **RESPONSE_TO_ORGANIC_SUBSTANCE** | **RESPONSE_TO_ORGANIC_SUBSTANCE** | | **27** | **-0.25405395** | **-0.81323826** | **0.7056213** | **0.91344935** | **1** | **247** | **tags=11%, list=2%, signal=11%** |
| **POSITIVE_REGULATION_OF_CELLULAR_PROCESS** | **POSITIVE_REGULATION_OF_CELLULAR_PROCESS** | | **540** | **-0.16976103** | **-0.81319815** | **0.953125** | **0.91161823** | **1** | **3236** | **tags=23%, list=24%, signal=29%** |
| **HETEROPHILIC_CELL_ADHESION** | **HETEROPHILIC_CELL_ADHESION** | | **6** | **-0.38028723** | **-0.81311655** | **0.7024** | **0.9098519** | **1** | **1777** | **tags=33%, list=13%, signal=38%** |
| **NEGATIVE_REGULATION_OF_TRANSFERASE_ACTIVITY** | **NEGATIVE_REGULATION_OF_TRANSFERASE_ACTIVITY** | | **28** | **-0.25105444** | **-0.81302655** | **0.74408287** | **0.9080931** | **1** | **4802** | **tags=43%, list=36%, signal=67%** |
| **POSITIVE_REGULATION_OF_SECRETION** | **POSITIVE_REGULATION_OF_SECRETION** | | **13** | **-0.3139349** | **-0.81254137** | **0.73878205** | **0.90696603** | **1** | **3162** | **tags=38%, list=24%, signal=50%** |
| **RESPONSE_TO_DNA_DAMAGE_STIMULUS** | **RESPONSE_TO_DNA_DAMAGE_STIMULUS** | | **144** | **-0.18939368** | **-0.80951744** | **0.85988027** | **0.91019094** | **1** | **3705** | **tags=28%, list=28%, signal=39%** |
| **POSITIVE_REGULATION_OF_JNK_ACTIVITY** | **POSITIVE_REGULATION_OF_JNK_ACTIVITY** | | **16** | **-0.28937966** | **-0.8094797** | **0.7332317** | **0.9083992** | **1** | **3958** | **tags=44%, list=30%, signal=62%** |
| **CELLULAR_RESPONSE_TO_STRESS** | **CELLULAR_RESPONSE_TO_STRESS** | | **8** | **-0.35226923** | **-0.80716175** | **0.712** | **0.91042286** | **1** | **5933** | **tags=63%, list=45%, signal=113%** |
| **REGULATION_OF_BINDING** | **REGULATION_OF_BINDING** | | **42** | **-0.22732472** | **-0.80122924** | **0.76836157** | **0.91817695** | **1** | **4154** | **tags=33%, list=31%, signal=48%** |
| **PROTEIN_COMPLEX_ASSEMBLY** | **PROTEIN_COMPLEX_ASSEMBLY** | | **149** | **-0.18749052** | **-0.80006224** | **0.86658794** | **0.9181828** | **1** | **4442** | **tags=31%, list=33%, signal=46%** |
| **REGULATION_OF_PROTEIN_STABILITY** | **REGULATION_OF_PROTEIN_STABILITY** | | **14** | **-0.29195994** | **-0.7997156** | **0.72** | **0.91685677** | **1** | **3487** | **tags=50%, list=26%, signal=68%** |
| **CELLULAR_CARBOHYDRATE_METABOLIC_PROCESS** | **CELLULAR_CARBOHYDRATE_METABOLIC_PROCESS** | | **104** | **-0.1918083** | **-0.7950501** | **0.8525** | **0.9223016** | **1** | **4173** | **tags=34%, list=31%, signal=49%** |
| **PROTEIN_IMPORT_INTO_NUCLEUS** | **PROTEIN_IMPORT_INTO_NUCLEUS** | | **42** | **-0.22785246** | **-0.7936438** | **0.7923729** | **0.9227331** | **1** | **2744** | **tags=26%, list=21%, signal=33%** |
| **REGULATION_OF_PROTEIN_AMINO_ACID_PHOSPHORYLATION** | **REGULATION_OF_PROTEIN_AMINO_ACID_PHOSPHORYLATION** | | **20** | **-0.26888734** | **-0.79102093** | **0.75** | **0.9249463** | **1** | **4464** | **tags=35%, list=34%, signal=53%** |
| **APOPTOSIS_GO** | **APOPTOSIS_GO** | | **373** | **-0.1671449** | **-0.78841364** | **0.9361702** | **0.9271985** | **1** | **1753** | **tags=14%, list=13%, signal=15%** |
| **BIOPOLYMER_BIOSYNTHETIC_PROCESS** | **BIOPOLYMER_BIOSYNTHETIC_PROCESS** | | **7** | **-0.35916463** | **-0.7855762** | **0.7608347** | **0.929748** | **1** | **3248** | **tags=43%, list=24%, signal=57%** |
| **CHROMOSOME_ORGANIZATION_AND_BIOGENESIS** | **CHROMOSOME_ORGANIZATION_AND_BIOGENESIS** | | **97** | **-0.19365266** | **-0.7851888** | **0.84019977** | **0.9284477** | **1** | **4449** | **tags=38%, list=33%, signal=57%** |
| **CELLULAR_POLYSACCHARIDE_METABOLIC_PROCESS** | **CELLULAR_POLYSACCHARIDE_METABOLIC_PROCESS** | | **11** | **-0.3116309** | **-0.7846072** | **0.76282054** | **0.92748433** | **1** | **3248** | **tags=36%, list=24%, signal=48%** |
| **DEPHOSPHORYLATION** | **DEPHOSPHORYLATION** | | **67** | **-0.20611356** | **-0.78333336** | **0.83355886** | **0.92751205** | **1** | **3360** | **tags=24%, list=25%, signal=32%** |
| **SPERMATID_DIFFERENTIATION** | **SPERMATID_DIFFERENTIATION** | | **8** | **-0.34845102** | **-0.7823267** | **0.7467742** | **0.92725796** | **1** | **4768** | **tags=63%, list=36%, signal=97%** |
| **GLYCOPROTEIN_METABOLIC_PROCESS** | **GLYCOPROTEIN_METABOLIC_PROCESS** | | **79** | **-0.19776803** | **-0.7791803** | **0.8505155** | **0.9299387** | **1** | **3780** | **tags=32%, list=28%, signal=44%** |
| **PROGRAMMED_CELL_DEATH** | **PROGRAMMED_CELL_DEATH** | | **374** | **-0.16561173** | **-0.7789379** | **0.95182014** | **0.92843825** | **1** | **1753** | **tags=14%, list=13%, signal=15%** |
| **REGULATION_OF_CELL_ADHESION** | **REGULATION_OF_CELL_ADHESION** | | **32** | **-0.23682569** | **-0.77508986** | **0.7841226** | **0.93203014** | **1** | **3116** | **tags=34%, list=23%, signal=45%** |
| **AMINO_ACID_AND_DERIVATIVE_METABOLIC_PROCESS** | **AMINO_ACID_AND_DERIVATIVE_METABOLIC_PROCESS** | | **95** | **-0.1945213** | **-0.7744371** | **0.8748404** | **0.9311305** | **1** | **4363** | **tags=39%, list=33%, signal=58%** |
| **PROTEIN_TRANSPORT** | **PROTEIN_TRANSPORT** | | **134** | **-0.18032543** | **-0.772872** | **0.8956109** | **0.93162763** | **1** | **1838** | **tags=15%, list=14%, signal=17%** |
| **REGULATION_OF_JNK_ACTIVITY** | **REGULATION_OF_JNK_ACTIVITY** | | **18** | **-0.2649828** | **-0.7724328** | **0.76197606** | **0.9303243** | **1** | **3958** | **tags=39%, list=30%, signal=55%** |
| **NEGATIVE_REGULATION_OF_BINDING** | **NEGATIVE_REGULATION_OF_BINDING** | | **15** | **-0.27688044** | **-0.77128094** | **0.7802198** | **0.93008006** | **1** | **3890** | **tags=33%, list=29%, signal=47%** |
| **PURINE_RIBONUCLEOTIDE_METABOLIC_PROCESS** | **PURINE_RIBONUCLEOTIDE_METABOLIC_PROCESS** | | **7** | **-0.34550497** | **-0.7691739** | **0.75993377** | **0.9311573** | **1** | **3791** | **tags=43%, list=28%, signal=60%** |
| **DNA_PACKAGING** | **DNA_PACKAGING** | | **27** | **-0.2412041** | **-0.76897347** | **0.79015917** | **0.9296159** | **1** | **4214** | **tags=37%, list=32%, signal=54%** |
| **DNA_DAMAGE_RESPONSESIGNAL_TRANSDUCTION** | **DNA_DAMAGE_RESPONSESIGNAL_TRANSDUCTION** | | **29** | **-0.23609826** | **-0.7677688** | **0.80588233** | **0.92942375** | **1** | **65** | **tags=7%, list=0%, signal=7%** |
| **PROTEIN_KINASE_CASCADE** | **PROTEIN_KINASE_CASCADE** | | **238** | **-0.1707042** | **-0.76687986** | **0.938258** | **0.928819** | **1** | **4004** | **tags=29%, list=30%, signal=41%** |
| **REGULATION_OF_NUCLEOCYTOPLASMIC_TRANSPORT** | **REGULATION_OF_NUCLEOCYTOPLASMIC_TRANSPORT** | | **18** | **-0.26684582** | **-0.7582218** | **0.7910906** | **0.9385249** | **1** | **4044** | **tags=33%, list=30%, signal=48%** |
| **PEPTIDYL_AMINO_ACID_MODIFICATION** | **PEPTIDYL_AMINO_ACID_MODIFICATION** | | **46** | **-0.21003631** | **-0.7572711** | **0.86565095** | **0.93790483** | **1** | **3041** | **tags=24%, list=23%, signal=31%** |
| **PIGMENT_BIOSYNTHETIC_PROCESS** | **PIGMENT_BIOSYNTHETIC_PROCESS** | | **17** | **-0.2700405** | **-0.7572557** | **0.78526646** | **0.93609667** | **1** | **4750** | **tags=41%, list=36%, signal=64%** |
| **PROTEIN_OLIGOMERIZATION** | **PROTEIN_OLIGOMERIZATION** | | **32** | **-0.23080418** | **-0.75621545** | **0.8193642** | **0.93567675** | **1** | **1389** | **tags=13%, list=10%, signal=14%** |
| **RESPONSE_TO_ENDOGENOUS_STIMULUS** | **RESPONSE_TO_ENDOGENOUS_STIMULUS** | | **172** | **-0.17274033** | **-0.7542435** | **0.9255319** | **0.93646616** | **1** | **2825** | **tags=21%, list=21%, signal=26%** |
| **BILE_ACID_METABOLIC_PROCESS** | **BILE_ACID_METABOLIC_PROCESS** | | **9** | **-0.31722745** | **-0.7537139** | **0.792393** | **0.93534976** | **1** | **4011** | **tags=44%, list=30%, signal=64%** |
| **COFACTOR_TRANSPORT** | **COFACTOR_TRANSPORT** | | **9** | **-0.3172381** | **-0.7510167** | **0.79614764** | **0.9371097** | **1** | **6487** | **tags=89%, list=49%, signal=173%** |
| **REGULATION_OF_TRANSCRIPTION_FACTOR_ACTIVITY** | **REGULATION_OF_TRANSCRIPTION_FACTOR_ACTIVITY** | | **27** | **-0.23283143** | **-0.75096935** | **0.80494905** | **0.93534315** | **1** | **5705** | **tags=56%, list=43%, signal=97%** |
| **INTERLEUKIN_8_PRODUCTION** | **INTERLEUKIN_8_PRODUCTION** | | **8** | **-0.32680094** | **-0.7475304** | **0.81566066** | **0.93797755** | **1** | **2682** | **tags=38%, list=20%, signal=47%** |
| **REGULATION_OF_GENE_EXPRESSION_EPIGENETIC** | **REGULATION_OF_GENE_EXPRESSION_EPIGENETIC** | | **27** | **-0.23062108** | **-0.74516493** | **0.8455882** | **0.93900347** | **1** | **3105** | **tags=30%, list=23%, signal=39%** |
| **POSITIVE_REGULATION_OF_CYTOSKELETON_ORGANIZATION_AND_BIOGENESIS** | **POSITIVE_REGULATION_OF_CYTOSKELETON_ORGANIZATION_AND_BIOGENESIS** | | **9** | **-0.31270882** | **-0.7425424** | **0.77483445** | **0.9403516** | **1** | **3041** | **tags=33%, list=23%, signal=43%** |
| **PROTEIN_PROCESSING** | **PROTEIN_PROCESSING** | | **40** | **-0.21483701** | **-0.7417546** | **0.8777174** | **0.9395011** | **1** | **2076** | **tags=20%, list=16%, signal=24%** |
| **POSITIVE_REGULATION_OF_PROTEIN_AMINO_ACID_PHOSPHORYLATION** | **POSITIVE_REGULATION_OF_PROTEIN_AMINO_ACID_PHOSPHORYLATION** | | **14** | **-0.27317616** | **-0.7398304** | **0.8297546** | **0.9399771** | **1** | **4464** | **tags=36%, list=34%, signal=54%** |
| **INTRA_GOLGI_VESICLE_MEDIATED_TRANSPORT** | **INTRA_GOLGI_VESICLE_MEDIATED_TRANSPORT** | | **11** | **-0.2936221** | **-0.73767847** | **0.81219906** | **0.940785** | **1** | **333** | **tags=9%, list=2%, signal=9%** |
| **CELLULAR_DEFENSE_RESPONSE** | **CELLULAR_DEFENSE_RESPONSE** | | **50** | **-0.20101516** | **-0.7338367** | **0.8852459** | **0.94334555** | **1** | **2756** | **tags=20%, list=21%, signal=25%** |
| **REGULATION_OF_PROTEIN_IMPORT_INTO_NUCLEUS** | **REGULATION_OF_PROTEIN_IMPORT_INTO_NUCLEUS** | | **13** | **-0.27739283** | **-0.72980434** | **0.81219906** | **0.94616663** | **1** | **2682** | **tags=23%, list=20%, signal=29%** |
| **PROTEIN_STABILIZATION** | **PROTEIN_STABILIZATION** | | **8** | **-0.3229597** | **-0.72701484** | **0.83362216** | **0.9474301** | **1** | **3263** | **tags=50%, list=24%, signal=66%** |
| **HETEROCYCLE_METABOLIC_PROCESS** | **HETEROCYCLE_METABOLIC_PROCESS** | | **25** | **-0.23101556** | **-0.7214598** | **0.8460389** | **0.9518273** | **1** | **3215** | **tags=24%, list=24%, signal=32%** |
| **PROTEIN_IMPORT** | **PROTEIN_IMPORT** | | **55** | **-0.19308317** | **-0.72106344** | **0.9123288** | **0.9504395** | **1** | **2744** | **tags=22%, list=21%, signal=27%** |
| **POSITIVE_REGULATION_OF_TRANSCRIPTION_FROM_RNA_POLYMERASE_II_PROMOTER** | **POSITIVE_REGULATION_OF_TRANSCRIPTION_FROM_RNA_POLYMERASE_II_PROMOTER** | | **53** | **-0.19566762** | **-0.7161232** | **0.8897638** | **0.95386547** | **1** | **4625** | **tags=42%, list=35%, signal=63%** |
| **POSITIVE_REGULATION_OF_TRANSCRIPTION** | **POSITIVE_REGULATION_OF_TRANSCRIPTION** | | **109** | **-0.17470264** | **-0.7147453** | **0.94823235** | **0.95348805** | **1** | **3236** | **tags=25%, list=24%, signal=32%** |
| **REGULATION_OF_APOPTOSIS** | **REGULATION_OF_APOPTOSIS** | | **295** | **-0.15662566** | **-0.7104221** | **0.98674035** | **0.9559926** | **1** | **1753** | **tags=13%, list=13%, signal=15%** |
| **REGULATION_OF_PROGRAMMED_CELL_DEATH** | **REGULATION_OF_PROGRAMMED_CELL_DEATH** | | **296** | **-0.15478007** | **-0.7101903** | **0.98801744** | **0.95442975** | **1** | **1753** | **tags=13%, list=13%, signal=15%** |
| **DNA_DEPENDENT_DNA_REPLICATION** | **DNA_DEPENDENT_DNA_REPLICATION** | | **46** | **-0.19603412** | **-0.70972174** | **0.9126876** | **0.9530812** | **1** | **3041** | **tags=24%, list=23%, signal=31%** |
| **SENSORY_PERCEPTION_OF_CHEMICAL_STIMULUS** | **SENSORY_PERCEPTION_OF_CHEMICAL_STIMULUS** | | **12** | **-0.27859768** | **-0.7077601** | **0.8632075** | **0.95327073** | **1** | **4760** | **tags=50%, list=36%, signal=78%** |
| **STRIATED_MUSCLE_DEVELOPMENT** | **STRIATED_MUSCLE_DEVELOPMENT** | | **37** | **-0.20463528** | **-0.69476336** | **0.9026063** | **0.9637342** | **1** | **4703** | **tags=41%, list=35%, signal=62%** |
| **PROTEIN_AMINO_ACID_PHOSPHORYLATION** | **PROTEIN_AMINO_ACID_PHOSPHORYLATION** | | **225** | **-0.1538071** | **-0.69412315** | **0.99538106** | **0.96255296** | **1** | **4262** | **tags=32%, list=32%, signal=46%** |
| **REGULATION_OF_GENE_SPECIFIC_TRANSCRIPTION** | **REGULATION_OF_GENE_SPECIFIC_TRANSCRIPTION** | | **9** | **-0.29199505** | **-0.6897256** | **0.8419405** | **0.96449566** | **1** | **1561** | **tags=22%, list=12%, signal=25%** |
| **PURINE_NUCLEOTIDE_METABOLIC_PROCESS** | **PURINE_NUCLEOTIDE_METABOLIC_PROCESS** | | **9** | **-0.2841925** | **-0.68483007** | **0.8665568** | **0.9671255** | **1** | **3791** | **tags=33%, list=28%, signal=47%** |
| **DEVELOPMENTAL_GROWTH** | **DEVELOPMENTAL_GROWTH** | | **7** | **-0.3070574** | **-0.678796** | **0.877551** | **0.97032917** | **1** | **209** | **tags=14%, list=2%, signal=15%** |
| **ACTIVATION_OF_JNK_ACTIVITY** | **ACTIVATION_OF_JNK_ACTIVITY** | | **14** | **-0.25674048** | **-0.6771579** | **0.86846274** | **0.9698775** | **1** | **3958** | **tags=43%, list=30%, signal=61%** |
| **NUCLEOTIDE_METABOLIC_PROCESS** | **NUCLEOTIDE_METABOLIC_PROCESS** | | **34** | **-0.20189373** | **-0.6749346** | **0.91865355** | **0.96994656** | **1** | **4044** | **tags=26%, list=30%, signal=38%** |
| **SPERMATID_DEVELOPMENT** | **SPERMATID_DEVELOPMENT** | | **7** | **-0.29995435** | **-0.6718389** | **0.884106** | **0.97070044** | **1** | **4768** | **tags=57%, list=36%, signal=89%** |
| **CHROMATIN_ASSEMBLY** | **CHROMATIN_ASSEMBLY** | | **16** | **-0.23411305** | **-0.6679481** | **0.8786482** | **0.97178257** | **1** | **4214** | **tags=44%, list=32%, signal=64%** |
| **HORMONE_METABOLIC_PROCESS** | **HORMONE_METABOLIC_PROCESS** | | **27** | **-0.20625345** | **-0.6557624** | **0.9085714** | **0.97870374** | **1** | **4014** | **tags=33%, list=30%, signal=48%** |
| **AMINO_ACID_CATABOLIC_PROCESS** | **AMINO_ACID_CATABOLIC_PROCESS** | | **22** | **-0.20595224** | **-0.63672394** | **0.9426934** | **0.9892323** | **1** | **4229** | **tags=50%, list=32%, signal=73%** |
| **DNA_REPAIR** | **DNA_REPAIR** | | **115** | **-0.15499413** | **-0.63558906** | **0.9842615** | **0.9880619** | **1** | **3705** | **tags=26%, list=28%, signal=36%** |
| **TRANSCRIPTION_FROM_RNA_POLYMERASE_III_PROMOTER** | **TRANSCRIPTION_FROM_RNA_POLYMERASE_III_PROMOTER** | | **18** | **-0.21413092** | **-0.6216625** | **0.923676** | **0.9941213** | **1** | **1099** | **tags=11%, list=8%, signal=12%** |
| **POSITIVE_REGULATION_OF_TRANSCRIPTIONDNA_DEPENDENT** | **POSITIVE_REGULATION_OF_TRANSCRIPTIONDNA_DEPENDENT** | | **93** | **-0.1523964** | **-0.61445415** | **0.9835443** | **0.9959291** | **1** | **3236** | **tags=24%, list=24%, signal=31%** |
| **REGULATION_OF_MUSCLE_CONTRACTION** | **REGULATION_OF_MUSCLE_CONTRACTION** | | **17** | **-0.21304888** | **-0.6088027** | **0.9485294** | **0.9970186** | **1** | **3629** | **tags=35%, list=27%, signal=48%** |
| **SKELETAL_MUSCLE_DEVELOPMENT** | **SKELETAL_MUSCLE_DEVELOPMENT** | | **30** | **-0.18433003** | **-0.602768** | **0.95487624** | **0.9980438** | **1** | **4703** | **tags=40%, list=35%, signal=62%** |
| **UBIQUITIN_CYCLE** | **UBIQUITIN_CYCLE** | | **42** | **-0.17230217** | **-0.601861** | **0.9775596** | **0.9966383** | **1** | **3199** | **tags=21%, list=24%, signal=28%** |
| **NITROGEN_COMPOUND_CATABOLIC_PROCESS** | **NITROGEN_COMPOUND_CATABOLIC_PROCESS** | | **26** | **-0.18931349** | **-0.59824795** | **0.94871795** | **0.99638855** | **1** | **4318** | **tags=46%, list=32%, signal=68%** |
| **RIBONUCLEOTIDE_METABOLIC_PROCESS** | **RIBONUCLEOTIDE_METABOLIC_PROCESS** | | **11** | **-0.23698042** | **-0.5971849** | **0.95548487** | **0.9950482** | **1** | **5439** | **tags=45%, list=41%, signal=77%** |
| **REGULATION_OF_DNA_BINDING** | **REGULATION_OF_DNA_BINDING** | | **34** | **-0.17689727** | **-0.591179** | **0.9709945** | **0.9956091** | **1** | **4477** | **tags=35%, list=34%, signal=53%** |
| **RESPONSE_TO_STEROID_HORMONE_STIMULUS** | **RESPONSE_TO_STEROID_HORMONE_STIMULUS** | | **8** | **-0.2555036** | **-0.5907597** | **0.94391024** | **0.9939767** | **1** | **4077** | **tags=38%, list=31%, signal=54%** |
| **JAK_STAT_CASCADE** | **JAK_STAT_CASCADE** | | **26** | **-0.18416919** | **-0.58339167** | **0.96910113** | **0.9949989** | **1** | **4419** | **tags=31%, list=33%, signal=46%** |
| **PROTEIN_AMINO_ACID_AUTOPHOSPHORYLATION** | **PROTEIN_AMINO_ACID_AUTOPHOSPHORYLATION** | | **24** | **-0.1875526** | **-0.57894486** | **0.9583333** | **0.99488944** | **1** | **4386** | **tags=33%, list=33%, signal=50%** |
| **PROTEIN_AUTOPROCESSING** | **PROTEIN_AUTOPROCESSING** | | **24** | **-0.1875526** | **-0.5756065** | **0.96511626** | **0.9942899** | **1** | **4386** | **tags=33%, list=33%, signal=50%** |
| **CELLULAR_CARBOHYDRATE_CATABOLIC_PROCESS** | **CELLULAR_CARBOHYDRATE_CATABOLIC_PROCESS** | | **20** | **-0.19463891** | **-0.5746898** | **0.9623494** | **0.9928449** | **1** | **4995** | **tags=40%, list=37%, signal=64%** |
| **CARBOHYDRATE_CATABOLIC_PROCESS** | **CARBOHYDRATE_CATABOLIC_PROCESS** | | **20** | **-0.19463891** | **-0.5712317** | **0.9714715** | **0.99220985** | **1** | **4995** | **tags=40%, list=37%, signal=64%** |
| **CERAMIDE_METABOLIC_PROCESS** | **CERAMIDE_METABOLIC_PROCESS** | | **8** | **-0.24477993** | **-0.5651054** | **0.9575163** | **0.992441** | **1** | **4926** | **tags=50%, list=37%, signal=79%** |
| **AMINE_CATABOLIC_PROCESS** | **AMINE_CATABOLIC_PROCESS** | | **24** | **-0.18299228** | **-0.5645273** | **0.98384726** | **0.9908386** | **1** | **4229** | **tags=46%, list=32%, signal=67%** |
| **COFACTOR_CATABOLIC_PROCESS** | **COFACTOR_CATABOLIC_PROCESS** | | **9** | **-0.23671894** | **-0.5534675** | **0.95859873** | **0.99223787** | **1** | **2650** | **tags=22%, list=20%, signal=28%** |
| **UNFOLDED_PROTEIN_RESPONSE** | **UNFOLDED_PROTEIN_RESPONSE** | | **8** | **-0.24080344** | **-0.5476651** | **0.9718076** | **0.9920209** | **1** | **3952** | **tags=25%, list=30%, signal=36%** |
| **MUSCLE_CELL_DIFFERENTIATION** | **MUSCLE_CELL_DIFFERENTIATION** | | **19** | **-0.18370648** | **-0.544746** | **0.97462684** | **0.9910255** | **1** | **6045** | **tags=58%, list=45%, signal=106%** |
| **CYTOKINE_AND_CHEMOKINE_MEDIATED_SIGNALING_PATHWAY** | **CYTOKINE_AND_CHEMOKINE_MEDIATED_SIGNALING_PATHWAY** | | **18** | **-0.18657234** | **-0.5415939** | **0.9862805** | **0.99003536** | **1** | **4146** | **tags=28%, list=31%, signal=40%** |
| **SPHINGOID_METABOLIC_PROCESS** | **SPHINGOID_METABOLIC_PROCESS** | | **9** | **-0.22293174** | **-0.5387406** | **0.96988904** | **0.988964** | **1** | **4926** | **tags=44%, list=37%, signal=70%** |
| **TRNA_METABOLIC_PROCESS** | **TRNA_METABOLIC_PROCESS** | | **15** | **-0.18987115** | **-0.52904403** | **0.98033285** | **0.989253** | **1** | **5302** | **tags=53%, list=40%, signal=88%** |
| **NEGATIVE_REGULATION_OF_TRANSPORT** | **NEGATIVE_REGULATION_OF_TRANSPORT** | | **16** | **-0.15396872** | **-0.42240766** | **0.9969325** | **0.9987698** | **1** | **6168** | **tags=63%, list=46%, signal=116%** |

**Table S2.3.- GSE10072: Never smokers, tumor, enriched in men**

| **NAME** | **GS<br> follow link to MSigDB** | **GS DETAILS** | **SIZE** | **ES** | **NES** | **NOM p-val** | **FDR q-val** | **FWER p-val** | **RANK AT MAX** | **LEADING EDGE** |
| --- | --- | --- | --- | --- | --- | --- | --- | --- | --- | --- |
| **I_KAPPAB_KINASE_NF_KAPPAB_CASCADE** | **I_KAPPAB_KINASE_NF_KAPPAB_CASCADE** | **Details ...** | **88** | **0.41039208** | **2.0494995** | **0** | **0.33732894** | **0.175** | **2080** | **tags=35%, list=16%, signal=41%** |
| **JAK_STAT_CASCADE** | **JAK_STAT_CASCADE** | **Details ...** | **26** | **0.51016515** | **1.9282993** | **0** | **0.4650545** | **0.395** | **2957** | **tags=58%, list=22%, signal=74%** |
| **REGULATION_OF_I_KAPPAB_KINASE_NF_KAPPAB_CASCADE** | **REGULATION_OF_I_KAPPAB_KINASE_NF_KAPPAB_CASCADE** | **Details ...** | **71** | **0.39051318** | **1.8955784** | **0.01111111** | **0.40589818** | **0.495** | **3901** | **tags=56%, list=29%, signal=79%** |
| **POSITIVE_REGULATION_OF_I_KAPPAB_KINASE_NF_KAPPAB_CASCADE** | **POSITIVE_REGULATION_OF_I_KAPPAB_KINASE_NF_KAPPAB_CASCADE** | **Details ...** | **66** | **0.38918003** | **1.8851334** | **0** | **0.32359967** | **0.516** | **3901** | **tags=58%, list=29%, signal=81%** |
| **INACTIVATION_OF_MAPK_ACTIVITY** | **INACTIVATION_OF_MAPK_ACTIVITY** | **Details ...** | **12** | **0.5910063** | **1.8188345** | **0.01060071** | **0.43198204** | **0.705** | **2579** | **tags=67%, list=19%, signal=83%** |
| **IMMUNE_RESPONSE** | **IMMUNE_RESPONSE** | **Details ...** | **203** | **0.2972197** | **1.7938412** | **0** | **0.4263623** | **0.766** | **2141** | **tags=34%, list=16%, signal=40%** |
| **POST_GOLGI_VESICLE_MEDIATED_TRANSPORT** | **POST_GOLGI_VESICLE_MEDIATED_TRANSPORT** | **Details ...** | **13** | **0.58086866** | **1.782229** | **0.01098901** | **0.39620456** | **0.793** | **720** | **tags=31%, list=5%, signal=32%** |
| **CELLULAR_RESPIRATION** | **CELLULAR_RESPIRATION** | **Details ...** | **19** | **0.5067012** | **1.7819663** | **0.00480769** | **0.3475956** | **0.794** | **3192** | **tags=53%, list=24%, signal=69%** |
| **POSITIVE_REGULATION_OF_SIGNAL_TRANSDUCTION** | **POSITIVE_REGULATION_OF_SIGNAL_TRANSDUCTION** | **Details ...** | **93** | **0.33570296** | **1.6887124** | **0** | **0.54996246** | **0.939** | **3901** | **tags=56%, list=29%, signal=79%** |
| **RESPONSE_TO_VIRUS** | **RESPONSE_TO_VIRUS** | **Details ...** | **43** | **0.38184425** | **1.6649565** | **0.01709402** | **0.5672258** | **0.966** | **3353** | **tags=51%, list=25%, signal=68%** |
| **VIRAL_GENOME_REPLICATION** | **VIRAL_GENOME_REPLICATION** | **Details ...** | **20** | **0.46760195** | **1.6629618** | **0.01229508** | **0.52287734** | **0.967** | **3268** | **tags=60%, list=25%, signal=79%** |
| **PROTEIN_KINASE_CASCADE** | **PROTEIN_KINASE_CASCADE** | **Details ...** | **238** | **0.2825336** | **1.6426741** | **0** | **0.5395339** | **0.979** | **2957** | **tags=39%, list=22%, signal=49%** |
| **NEGATIVE_REGULATION_OF_TRANSFERASE_ACTIVITY** | **NEGATIVE_REGULATION_OF_TRANSFERASE_ACTIVITY** | **Details ...** | **28** | **0.41433302** | **1.6179575** | **0.02777778** | **0.5788742** | **0.988** | **2579** | **tags=50%, list=19%, signal=62%** |
| **T_CELL_DIFFERENTIATION** | **T_CELL_DIFFERENTIATION** | **Details ...** | **14** | **0.5043232** | **1.5986798** | **0.03149606** | **0.5980288** | **0.992** | **402** | **tags=29%, list=3%, signal=29%** |
| **AGING** | **AGING** | **Details ...** | **11** | **0.53370094** | **1.5922546** | **0.04411765** | **0.57718563** | **0.993** | **2606** | **tags=55%, list=20%, signal=68%** |
| **REGULATION_OF_GTPASE_ACTIVITY** | **REGULATION_OF_GTPASE_ACTIVITY** | **Details ...** | **9** | **0.5832787** | **1.5634665** | **0.05629139** | **0.6365087** | **0.996** | **979** | **tags=44%, list=7%, signal=48%** |
| **NEGATIVE_REGULATION_OF_TRANSCRIPTION** | **NEGATIVE_REGULATION_OF_TRANSCRIPTION** | **Details ...** | **148** | **0.2819772** | **1.561026** | **0** | **0.60656345** | **0.997** | **1480** | **tags=24%, list=11%, signal=26%** |
| **PEPTIDE_METABOLIC_PROCESS** | **PEPTIDE_METABOLIC_PROCESS** | **Details ...** | **9** | **0.58715194** | **1.554362** | **0.04651163** | **0.59395456** | **0.998** | **2597** | **tags=56%, list=19%, signal=69%** |
| **AEROBIC_RESPIRATION** | **AEROBIC_RESPIRATION** | **Details ...** | **15** | **0.4651564** | **1.5515096** | **0.03137255** | **0.571249** | **0.998** | **3100** | **tags=47%, list=23%, signal=61%** |
| **IMMUNE_SYSTEM_PROCESS** | **IMMUNE_SYSTEM_PROCESS** | **Details ...** | **286** | **0.25173745** | **1.5491731** | **0** | **0.5489272** | **0.998** | **2257** | **tags=31%, list=17%, signal=37%** |
| **NEGATIVE_REGULATION_OF_MAP_KINASE_ACTIVITY** | **NEGATIVE_REGULATION_OF_MAP_KINASE_ACTIVITY** | **Details ...** | **15** | **0.4636269** | **1.5024538** | **0.04938272** | **0.65767366** | **1** | **2579** | **tags=53%, list=19%, signal=66%** |
| **MAINTENANCE_OF_LOCALIZATION** | **MAINTENANCE_OF_LOCALIZATION** | **Details ...** | **19** | **0.41580206** | **1.4777123** | **0.05833333** | **0.7082213** | **1** | **2284** | **tags=42%, list=17%, signal=51%** |
| **REGULATION_OF_AXONOGENESIS** | **REGULATION_OF_AXONOGENESIS** | **Details ...** | **8** | **0.5615086** | **1.4724373** | **0.07028754** | **0.6974142** | **1** | **1511** | **tags=63%, list=11%, signal=70%** |
| **SPLICEOSOME_ASSEMBLY** | **SPLICEOSOME_ASSEMBLY** | **Details ...** | **10** | **0.51184267** | **1.4492576** | **0.08163265** | **0.7498213** | **1** | **1987** | **tags=50%, list=15%, signal=59%** |
| **CELL_CYCLE_CHECKPOINT_GO_0000075** | **CELL_CYCLE_CHECKPOINT_GO_0000075** | **Details ...** | **42** | **0.33888662** | **1.4417005** | **0.02702703** | **0.7443669** | **1** | **2558** | **tags=36%, list=19%, signal=44%** |
| **NEGATIVE_REGULATION_OF_CYTOSKELETON_ORGANIZATION_AND_BIOGENESIS** | **NEGATIVE_REGULATION_OF_CYTOSKELETON_ORGANIZATION_AND_BIOGENESIS** | **Details ...** | **10** | **0.512083** | **1.4414849** | **0.07633588** | **0.71627504** | **1** | **1333** | **tags=30%, list=10%, signal=33%** |
| **NEGATIVE_REGULATION_OF_TRANSCRIPTION_FACTOR_ACTIVITY** | **NEGATIVE_REGULATION_OF_TRANSCRIPTION_FACTOR_ACTIVITY** | **Details ...** | **12** | **0.45526314** | **1.4085156** | **0.10037175** | **0.8054182** | **1** | **1379** | **tags=42%, list=10%, signal=46%** |
| **REGULATION_OF_SIGNAL_TRANSDUCTION** | **REGULATION_OF_SIGNAL_TRANSDUCTION** | **Details ...** | **168** | **0.24162702** | **1.3987794** | **0** | **0.8144419** | **1** | **3901** | **tags=47%, list=29%, signal=66%** |
| **T_CELL_ACTIVATION** | **T_CELL_ACTIVATION** | **Details ...** | **39** | **0.33091912** | **1.3928971** | **0.04458599** | **0.8079239** | **1** | **1126** | **tags=26%, list=8%, signal=28%** |
| **PURINE_NUCLEOTIDE_METABOLIC_PROCESS** | **PURINE_NUCLEOTIDE_METABOLIC_PROCESS** | **Details ...** | **9** | **0.4977778** | **1.3875376** | **0.10784314** | **0.8005011** | **1** | **2773** | **tags=67%, list=21%, signal=84%** |
| **NEGATIVE_REGULATION_OF_NUCLEOBASENUCLEOSIDENUCLEOTIDE_AND_NUCLEIC_ACID_METABOLIC_PROCESS** | **NEGATIVE_REGULATION_OF_NUCLEOBASENUCLEOSIDENUCLEOTIDE_AND_NUCLEIC_ACID_METABOLIC_PROCESS** | **Details ...** | **167** | **0.25194013** | **1.3869779** | **0** | **0.77645874** | **1** | **1480** | **tags=22%, list=11%, signal=24%** |
| **REGULATION_OF_RHO_PROTEIN_SIGNAL_TRANSDUCTION** | **REGULATION_OF_RHO_PROTEIN_SIGNAL_TRANSDUCTION** | **Details ...** | **7** | **0.549471** | **1.3868848** | **0.11111111** | **0.7525223** | **1** | **130** | **tags=29%, list=1%, signal=29%** |
| **REGULATION_OF_RAS_GTPASE_ACTIVITY** | **REGULATION_OF_RAS_GTPASE_ACTIVITY** | **Details ...** | **6** | **0.58914834** | **1.3790513** | **0.14529915** | **0.75617164** | **1** | **130** | **tags=33%, list=1%, signal=34%** |
| **REGULATION_OF_NEUROGENESIS** | **REGULATION_OF_NEUROGENESIS** | **Details ...** | **12** | **0.46785823** | **1.3704306** | **0.12686567** | **0.76326674** | **1** | **1511** | **tags=42%, list=11%, signal=47%** |
| **RNA_3END_PROCESSING** | **RNA_3END_PROCESSING** | **Details ...** | **8** | **0.5294272** | **1.3597567** | **0.12418301** | **0.77945876** | **1** | **4685** | **tags=88%, list=35%, signal=135%** |
| **POSITIVE_REGULATION_OF_CYTOKINE_SECRETION** | **POSITIVE_REGULATION_OF_CYTOKINE_SECRETION** | **Details ...** | **5** | **0.6258529** | **1.3568392** | **0.14088398** | **0.76923555** | **1** | **333** | **tags=40%, list=2%, signal=41%** |
| **VIRAL_REPRODUCTIVE_PROCESS** | **VIRAL_REPRODUCTIVE_PROCESS** | **Details ...** | **33** | **0.3256749** | **1.348376** | **0.08450704** | **0.7764775** | **1** | **2533** | **tags=36%, list=19%, signal=45%** |
| **REGULATION_OF_RAS_PROTEIN_SIGNAL_TRANSDUCTION** | **REGULATION_OF_RAS_PROTEIN_SIGNAL_TRANSDUCTION** | **Details ...** | **11** | **0.4637959** | **1.3444798** | **0.125** | **0.7690861** | **1** | **130** | **tags=18%, list=1%, signal=18%** |
| **RNA_SPLICING** | **RNA_SPLICING** | **Details ...** | **59** | **0.28545767** | **1.3435912** | **0.04761905** | **0.752878** | **1** | **3308** | **tags=37%, list=25%, signal=49%** |
| **TYROSINE_PHOSPHORYLATION_OF_STAT_PROTEIN** | **TYROSINE_PHOSPHORYLATION_OF_STAT_PROTEIN** | **Details ...** | **9** | **0.48874438** | **1.3433872** | **0.1369863** | **0.7347189** | **1** | **2871** | **tags=56%, list=22%, signal=71%** |
| **REGULATION_OF_RHO_GTPASE_ACTIVITY** | **REGULATION_OF_RHO_GTPASE_ACTIVITY** | **Details ...** | **5** | **0.6044218** | **1.3366723** | **0.15406163** | **0.7389478** | **1** | **130** | **tags=40%, list=1%, signal=40%** |
| **ENDOSOME_TRANSPORT** | **ENDOSOME_TRANSPORT** | **Details ...** | **22** | **0.35897613** | **1.3353614** | **0.1** | **0.7253807** | **1** | **2272** | **tags=36%, list=17%, signal=44%** |
| **PROTEOLYSIS** | **PROTEOLYSIS** | **Details ...** | **160** | **0.23933683** | **1.3331604** | **0** | **0.71604246** | **1** | **2432** | **tags=29%, list=18%, signal=36%** |
| **REGULATION_OF_SMALL_GTPASE_MEDIATED_SIGNAL_TRANSDUCTION** | **REGULATION_OF_SMALL_GTPASE_MEDIATED_SIGNAL_TRANSDUCTION** | **Details ...** | **14** | **0.40708867** | **1.3310803** | **0.09677419** | **0.70577294** | **1** | **130** | **tags=14%, list=1%, signal=14%** |
| **RNA_PROCESSING** | **RNA_PROCESSING** | **Details ...** | **117** | **0.25840554** | **1.3266748** | **0.05128205** | **0.70376885** | **1** | **3063** | **tags=35%, list=23%, signal=45%** |
| **PROGRAMMED_CELL_DEATH** | **PROGRAMMED_CELL_DEATH** | **Details ...** | **374** | **0.21071973** | **1.3173395** | **0** | **0.71757716** | **1** | **3042** | **tags=34%, list=23%, signal=43%** |
| **NEGATIVE_REGULATION_OF_SIGNAL_TRANSDUCTION** | **NEGATIVE_REGULATION_OF_SIGNAL_TRANSDUCTION** | **Details ...** | **30** | **0.33535695** | **1.3159027** | **0.125** | **0.7065388** | **1** | **1035** | **tags=20%, list=8%, signal=22%** |
| **SULFUR_COMPOUND_BIOSYNTHETIC_PROCESS** | **SULFUR_COMPOUND_BIOSYNTHETIC_PROCESS** | **Details ...** | **13** | **0.42401275** | **1.3062264** | **0.14341085** | **0.7221804** | **1** | **725** | **tags=31%, list=5%, signal=33%** |
| **RESPONSE_TO_BIOTIC_STIMULUS** | **RESPONSE_TO_BIOTIC_STIMULUS** | **Details ...** | **97** | **0.25864145** | **1.2961371** | **0.03508772** | **0.7397036** | **1** | **3236** | **tags=41%, list=24%, signal=54%** |
| **VIRAL_REPRODUCTION** | **VIRAL_REPRODUCTION** | **Details ...** | **38** | **0.29933926** | **1.2933747** | **0.04375** | **0.7333713** | **1** | **3724** | **tags=50%, list=28%, signal=69%** |
| **CELLULAR_PROTEIN_CATABOLIC_PROCESS** | **CELLULAR_PROTEIN_CATABOLIC_PROCESS** | | **49** | **0.2885482** | **1.2921487** | **0.10679612** | **0.72205645** | **1** | **994** | **tags=18%, list=7%, signal=20%** |
| **CELLULAR_DEFENSE_RESPONSE** | **CELLULAR_DEFENSE_RESPONSE** | | **50** | **0.28669322** | **1.2893914** | **0.06923077** | **0.7154045** | **1** | **2236** | **tags=36%, list=17%, signal=43%** |
| **NEGATIVE_REGULATION_OF_METABOLIC_PROCESS** | **NEGATIVE_REGULATION_OF_METABOLIC_PROCESS** | | **212** | **0.22578496** | **1.2858863** | **0** | **0.7129886** | **1** | **1480** | **tags=20%, list=11%, signal=22%** |
| **MRNA_METABOLIC_PROCESS** | **MRNA_METABOLIC_PROCESS** | | **56** | **0.27880883** | **1.2846472** | **0.07** | **0.70348793** | **1** | **3063** | **tags=39%, list=23%, signal=51%** |
| **DEFENSE_RESPONSE_TO_VIRUS** | **DEFENSE_RESPONSE_TO_VIRUS** | | **10** | **0.44715482** | **1.2765595** | **0.17921147** | **0.7156803** | **1** | **364** | **tags=20%, list=3%, signal=21%** |
| **REGIONALIZATION** | **REGIONALIZATION** | | **12** | **0.41950083** | **1.2731713** | **0.16370107** | **0.713129** | **1** | **1562** | **tags=33%, list=12%, signal=38%** |
| **CYTOKINE_AND_CHEMOKINE_MEDIATED_SIGNALING_PATHWAY** | **CYTOKINE_AND_CHEMOKINE_MEDIATED_SIGNALING_PATHWAY** | | **18** | **0.37625837** | **1.2718463** | **0.14410481** | **0.7042326** | **1** | **2449** | **tags=44%, list=18%, signal=54%** |
| **MRNA_PROCESSING_GO_0006397** | **MRNA_PROCESSING_GO_0006397** | | **45** | **0.29051468** | **1.2711133** | **0.08730159** | **0.69430727** | **1** | **4320** | **tags=56%, list=32%, signal=82%** |
| **COFACTOR_CATABOLIC_PROCESS** | **COFACTOR_CATABOLIC_PROCESS** | | **9** | **0.44439435** | **1.2701843** | **0.18627451** | **0.68559045** | **1** | **1215** | **tags=33%, list=9%, signal=37%** |
| **TRANSITION_METAL_ION_TRANSPORT** | **TRANSITION_METAL_ION_TRANSPORT** | | **10** | **0.44873655** | **1.2684814** | **0.19064748** | **0.679948** | **1** | **2437** | **tags=40%, list=18%, signal=49%** |
| **POSITIVE_REGULATION_OF_EPITHELIAL_CELL_PROLIFERATION** | **POSITIVE_REGULATION_OF_EPITHELIAL_CELL_PROLIFERATION** | | **7** | **0.49774042** | **1.2620693** | **0.19417475** | **0.6885547** | **1** | **785** | **tags=29%, list=6%, signal=30%** |
| **NEGATIVE_REGULATION_OF_CELLULAR_METABOLIC_PROCESS** | **NEGATIVE_REGULATION_OF_CELLULAR_METABOLIC_PROCESS** | | **209** | **0.23635678** | **1.2553366** | **0** | **0.6970815** | **1** | **2371** | **tags=28%, list=18%, signal=33%** |
| **NEGATIVE_REGULATION_OF_BINDING** | **NEGATIVE_REGULATION_OF_BINDING** | | **15** | **0.3930185** | **1.245122** | **0.18007663** | **0.71616304** | **1** | **1379** | **tags=33%, list=10%, signal=37%** |
| **POSITIVE_REGULATION_OF_DEVELOPMENTAL_PROCESS** | **POSITIVE_REGULATION_OF_DEVELOPMENTAL_PROCESS** | | **186** | **0.20734406** | **1.2444677** | **0** | **0.70683116** | **1** | **2454** | **tags=27%, list=18%, signal=33%** |
| **PROTEIN_CATABOLIC_PROCESS** | **PROTEIN_CATABOLIC_PROCESS** | | **58** | **0.2725999** | **1.2418532** | **0.09821428** | **0.703916** | **1** | **994** | **tags=17%, list=7%, signal=19%** |
| **CELL_RECOGNITION** | **CELL_RECOGNITION** | | **14** | **0.38405883** | **1.240269** | **0.18421052** | **0.6977321** | **1** | **1386** | **tags=36%, list=10%, signal=40%** |
| **NEGATIVE_REGULATION_OF_DNA_BINDING** | **NEGATIVE_REGULATION_OF_DNA_BINDING** | | **14** | **0.39705938** | **1.2395386** | **0.2109375** | **0.6896972** | **1** | **1379** | **tags=36%, list=10%, signal=40%** |
| **INFLAMMATORY_RESPONSE** | **INFLAMMATORY_RESPONSE** | | **107** | **0.23495199** | **1.2390004** | **0.02777778** | **0.6812227** | **1** | **2041** | **tags=26%, list=15%, signal=31%** |
| **POSITIVE_REGULATION_OF_PROTEIN_SECRETION** | **POSITIVE_REGULATION_OF_PROTEIN_SECRETION** | | **6** | **0.51846176** | **1.2324661** | **0.20858896** | **0.689683** | **1** | **333** | **tags=33%, list=2%, signal=34%** |
| **POSITIVE_REGULATION_OF_CASPASE_ACTIVITY** | **POSITIVE_REGULATION_OF_CASPASE_ACTIVITY** | | **26** | **0.325914** | **1.2310416** | **0.15816326** | **0.6838675** | **1** | **2798** | **tags=42%, list=21%, signal=53%** |
| **APOPTOSIS_GO** | **APOPTOSIS_GO** | | **373** | **0.20916583** | **1.2256202** | **0** | **0.6890169** | **1** | **3042** | **tags=34%, list=23%, signal=43%** |
| **LOCOMOTORY_BEHAVIOR** | **LOCOMOTORY_BEHAVIOR** | | **83** | **0.24485792** | **1.2250942** | **0.09090909** | **0.68097633** | **1** | **2297** | **tags=36%, list=17%, signal=43%** |
| **REGULATION_OF_T_CELL_ACTIVATION** | **REGULATION_OF_T_CELL_ACTIVATION** | | **26** | **0.31511608** | **1.218578** | **0.11627907** | **0.68982226** | **1** | **1126** | **tags=23%, list=8%, signal=25%** |
| **GLYCOPROTEIN_CATABOLIC_PROCESS** | **GLYCOPROTEIN_CATABOLIC_PROCESS** | | **11** | **0.42701757** | **1.2154995** | **0.22834645** | **0.6891517** | **1** | **4922** | **tags=82%, list=37%, signal=130%** |
| **NEGATIVE_REGULATION_OF_RNA_METABOLIC_PROCESS** | **NEGATIVE_REGULATION_OF_RNA_METABOLIC_PROCESS** | | **103** | **0.23662633** | **1.2147596** | **0.05357143** | **0.68188435** | **1** | **1446** | **tags=20%, list=11%, signal=23%** |
| **REGULATION_OF_PROGRAMMED_CELL_DEATH** | **REGULATION_OF_PROGRAMMED_CELL_DEATH** | | **296** | **0.20173895** | **1.2124931** | **0** | **0.67923385** | **1** | **3042** | **tags=34%, list=23%, signal=43%** |
| **LYSOSOMAL_TRANSPORT** | **LYSOSOMAL_TRANSPORT** | | **8** | **0.45037198** | **1.2113601** | **0.22903226** | **0.67310053** | **1** | **2841** | **tags=50%, list=21%, signal=64%** |
| **NEGATIVE_REGULATION_OF_TRANSCRIPTION_DNA_DEPENDENT** | **NEGATIVE_REGULATION_OF_TRANSCRIPTION_DNA_DEPENDENT** | | **103** | **0.23662628** | **1.209978** | **0.05769231** | **0.6682024** | **1** | **1446** | **tags=20%, list=11%, signal=23%** |
| **CYTOPLASM_ORGANIZATION_AND_BIOGENESIS** | **CYTOPLASM_ORGANIZATION_AND_BIOGENESIS** | | **13** | **0.3934375** | **1.2052082** | **0.24444444** | **0.6737589** | **1** | **2829** | **tags=46%, list=21%, signal=59%** |
| **RIBONUCLEOTIDE_METABOLIC_PROCESS** | **RIBONUCLEOTIDE_METABOLIC_PROCESS** | | **11** | **0.42369726** | **1.200379** | **0.23282443** | **0.6789189** | **1** | **2773** | **tags=55%, list=21%, signal=69%** |
| **LYMPHOCYTE_DIFFERENTIATION** | **LYMPHOCYTE_DIFFERENTIATION** | | **23** | **0.32281032** | **1.1979603** | **0.17258883** | **0.6771683** | **1** | **402** | **tags=17%, list=3%, signal=18%** |
| **NEGATIVE_REGULATION_OF_TRANSCRIPTION_FROM_RNA_POLYMERASE_II_PROMOTER** | **NEGATIVE_REGULATION_OF_TRANSCRIPTION_FROM_RNA_POLYMERASE_II_PROMOTER** | | **70** | **0.25052482** | **1.1932992** | **0.11904762** | **0.6817289** | **1** | **1446** | **tags=21%, list=11%, signal=24%** |
| **LEUKOCYTE_CHEMOTAXIS** | **LEUKOCYTE_CHEMOTAXIS** | | **12** | **0.38380507** | **1.1932108** | **0.18014705** | **0.6738829** | **1** | **2257** | **tags=58%, list=17%, signal=70%** |
| **NUCLEOCYTOPLASMIC_TRANSPORT** | **NUCLEOCYTOPLASMIC_TRANSPORT** | | **72** | **0.23660709** | **1.1803659** | **0.09210526** | **0.7019535** | **1** | **1961** | **tags=21%, list=15%, signal=24%** |
| **REGULATION_OF_CELL_GROWTH** | **REGULATION_OF_CELL_GROWTH** | | **37** | **0.273285** | **1.1766456** | **0.19259259** | **0.7040676** | **1** | **1294** | **tags=22%, list=10%, signal=24%** |
| **HOMOPHILIC_CELL_ADHESION** | **HOMOPHILIC_CELL_ADHESION** | | **12** | **0.37785247** | **1.1702045** | **0.22939068** | **0.71447116** | **1** | **1866** | **tags=33%, list=14%, signal=39%** |
| **TRANSCRIPTION_INITIATION_FROM_RNA_POLYMERASE_II_PROMOTER** | **TRANSCRIPTION_INITIATION_FROM_RNA_POLYMERASE_II_PROMOTER** | | **18** | **0.34365678** | **1.1693485** | **0.21860465** | **0.7084129** | **1** | **1456** | **tags=28%, list=11%, signal=31%** |
| **NUCLEAR_TRANSPORT** | **NUCLEAR_TRANSPORT** | | **72** | **0.23660709** | **1.1676394** | **0.12359551** | **0.7050656** | **1** | **1961** | **tags=21%, list=15%, signal=24%** |
| **REGULATION_OF_CELL_CELL_ADHESION** | **REGULATION_OF_CELL_CELL_ADHESION** | | **9** | **0.4207427** | **1.1675298** | **0.29054055** | **0.6974068** | **1** | **962** | **tags=22%, list=7%, signal=24%** |
| **INTRACELLULAR_PROTEIN_TRANSPORT** | **INTRACELLULAR_PROTEIN_TRANSPORT** | | **122** | **0.2186027** | **1.1668929** | **0.175** | **0.691628** | **1** | **1849** | **tags=17%, list=14%, signal=20%** |
| **ANDROGEN_RECEPTOR_SIGNALING_PATHWAY** | **ANDROGEN_RECEPTOR_SIGNALING_PATHWAY** | | **5** | **0.5301132** | **1.1662444** | **0.29411766** | **0.6857156** | **1** | **271** | **tags=20%, list=2%, signal=20%** |
| **REGULATION_OF_VIRAL_REPRODUCTION** | **REGULATION_OF_VIRAL_REPRODUCTION** | | **10** | **0.415155** | **1.1645238** | **0.23387097** | **0.6830191** | **1** | **3724** | **tags=60%, list=28%, signal=83%** |
| **REGULATION_OF_LYMPHOCYTE_ACTIVATION** | **REGULATION_OF_LYMPHOCYTE_ACTIVATION** | | **31** | **0.29600066** | **1.1610485** | **0.2173913** | **0.6844925** | **1** | **3849** | **tags=52%, list=29%, signal=72%** |
| **BIOPOLYMER_CATABOLIC_PROCESS** | **BIOPOLYMER_CATABOLIC_PROCESS** | | **101** | **0.21449777** | **1.1610137** | **0.14** | **0.6772476** | **1** | **1059** | **tags=16%, list=8%, signal=17%** |
| **RESPONSE_TO_IONIZING_RADIATION** | **RESPONSE_TO_IONIZING_RADIATION** | | **7** | **0.45964912** | **1.1568425** | **0.28143713** | **0.68152916** | **1** | **489** | **tags=29%, list=4%, signal=30%** |
| **NLS_BEARING_SUBSTRATE_IMPORT_INTO_NUCLEUS** | **NLS_BEARING_SUBSTRATE_IMPORT_INTO_NUCLEUS** | | **11** | **0.39163056** | **1.1567636** | **0.2518797** | **0.67473644** | **1** | **1611** | **tags=27%, list=12%, signal=31%** |
| **VACUOLAR_TRANSPORT** | **VACUOLAR_TRANSPORT** | | **9** | **0.43690363** | **1.1545076** | **0.25503355** | **0.6735818** | **1** | **2841** | **tags=44%, list=21%, signal=56%** |
| **LYMPHOCYTE_ACTIVATION** | **LYMPHOCYTE_ACTIVATION** | | **53** | **0.2518516** | **1.1518453** | **0.2038835** | **0.6735957** | **1** | **1379** | **tags=21%, list=10%, signal=23%** |
| **DEFENSE_RESPONSE** | **DEFENSE_RESPONSE** | | **222** | **0.209136** | **1.1517543** | **0.09090909** | **0.6670167** | **1** | **2351** | **tags=29%, list=18%, signal=34%** |
| **INTRACELLULAR_TRANSPORT** | **INTRACELLULAR_TRANSPORT** | | **240** | **0.19677994** | **1.1494706** | **0** | **0.6663463** | **1** | **2272** | **tags=19%, list=17%, signal=23%** |
| **ESTABLISHMENT_OF_VESICLE_LOCALIZATION** | **ESTABLISHMENT_OF_VESICLE_LOCALIZATION** | | **9** | **0.4183771** | **1.1482948** | **0.26878613** | **0.6631045** | **1** | **118** | **tags=11%, list=1%, signal=11%** |
| **PHOSPHOINOSITIDE_BIOSYNTHETIC_PROCESS** | **PHOSPHOINOSITIDE_BIOSYNTHETIC_PROCESS** | | **18** | **0.3339342** | **1.141254** | **0.24090908** | **0.6752921** | **1** | **2403** | **tags=22%, list=18%, signal=27%** |
| **LEUKOCYTE_MIGRATION** | **LEUKOCYTE_MIGRATION** | | **14** | **0.36416942** | **1.1378361** | **0.25910932** | **0.6778488** | **1** | **2517** | **tags=57%, list=19%, signal=70%** |
| **PURINE_RIBONUCLEOTIDE_METABOLIC_PROCESS** | **PURINE_RIBONUCLEOTIDE_METABOLIC_PROCESS** | | **7** | **0.45407555** | **1.1360002** | **0.2905983** | **0.6762466** | **1** | **2773** | **tags=71%, list=21%, signal=90%** |
| **REGULATION_OF_APOPTOSIS** | **REGULATION_OF_APOPTOSIS** | | **295** | **0.19975393** | **1.1298201** | **0** | **0.6853249** | **1** | **3042** | **tags=34%, list=23%, signal=43%** |
| **VIRAL_INFECTIOUS_CYCLE** | **VIRAL_INFECTIOUS_CYCLE** | | **29** | **0.28415734** | **1.1270351** | **0.26256984** | **0.6858819** | **1** | **2533** | **tags=34%, list=19%, signal=42%** |
| **CASPASE_ACTIVATION** | **CASPASE_ACTIVATION** | | **24** | **0.2980788** | **1.1245153** | **0.25247526** | **0.6862447** | **1** | **2798** | **tags=42%, list=21%, signal=53%** |
| **SULFUR_METABOLIC_PROCESS** | **SULFUR_METABOLIC_PROCESS** | | **30** | **0.274523** | **1.1228237** | **0.2580645** | **0.6844162** | **1** | **2285** | **tags=33%, list=17%, signal=40%** |
| **POSITIVE_REGULATION_OF_T_CELL_ACTIVATION** | **POSITIVE_REGULATION_OF_T_CELL_ACTIVATION** | | **20** | **0.31611946** | **1.1172615** | **0.3018018** | **0.69361454** | **1** | **1126** | **tags=25%, list=8%, signal=27%** |
| **APOPTOTIC_PROGRAM** | **APOPTOTIC_PROGRAM** | | **53** | **0.2389747** | **1.1110566** | **0.25619835** | **0.70454466** | **1** | **3026** | **tags=38%, list=23%, signal=49%** |
| **APOPTOTIC_MITOCHONDRIAL_CHANGES** | **APOPTOTIC_MITOCHONDRIAL_CHANGES** | | **10** | **0.38995436** | **1.1108229** | **0.31487888** | **0.69866973** | **1** | **2908** | **tags=40%, list=22%, signal=51%** |
| **REGULATION_OF_CELL_CYCLE** | **REGULATION_OF_CELL_CYCLE** | | **153** | **0.19915266** | **1.1041561** | **0.22727273** | **0.71010536** | **1** | **2399** | **tags=24%, list=18%, signal=28%** |
| **PHAGOCYTOSIS** | **PHAGOCYTOSIS** | | **16** | **0.34373894** | **1.1022378** | **0.28688523** | **0.70885015** | **1** | **1090** | **tags=25%, list=8%, signal=27%** |
| **REGULATION_OF_MAP_KINASE_ACTIVITY** | **REGULATION_OF_MAP_KINASE_ACTIVITY** | | **57** | **0.23755407** | **1.1013274** | **0.33333334** | **0.7051406** | **1** | **2949** | **tags=35%, list=22%, signal=45%** |
| **MESODERM_DEVELOPMENT** | **MESODERM_DEVELOPMENT** | | **22** | **0.29788703** | **1.0976503** | **0.29591838** | **0.70869285** | **1** | **2081** | **tags=41%, list=16%, signal=48%** |
| **DNA_DAMAGE_RESPONSESIGNAL_TRANSDUCTION_BY_P53_CLASS_MEDIATOR** | **DNA_DAMAGE_RESPONSESIGNAL_TRANSDUCTION_BY_P53_CLASS_MEDIATOR** | | **12** | **0.36119074** | **1.0915942** | **0.31407943** | **0.7193686** | **1** | **3216** | **tags=50%, list=24%, signal=66%** |
| **RNA_SPLICINGVIA_TRANSESTERIFICATION_REACTIONS** | **RNA_SPLICINGVIA_TRANSESTERIFICATION_REACTIONS** | | **17** | **0.32428584** | **1.084308** | **0.3105023** | **0.73298925** | **1** | **1987** | **tags=29%, list=15%, signal=35%** |
| **CELL_STRUCTURE_DISASSEMBLY_DURING_APOPTOSIS** | **CELL_STRUCTURE_DISASSEMBLY_DURING_APOPTOSIS** | | **16** | **0.33431274** | **1.0726793** | **0.3846154** | **0.75942725** | **1** | **822** | **tags=25%, list=6%, signal=27%** |
| **LEUKOCYTE_ACTIVATION** | **LEUKOCYTE_ACTIVATION** | | **58** | **0.22363694** | **1.0711173** | **0.31632653** | **0.75757974** | **1** | **1379** | **tags=21%, list=10%, signal=23%** |
| **RESPONSE_TO_OTHER_ORGANISM** | **RESPONSE_TO_OTHER_ORGANISM** | | **64** | **0.22559927** | **1.0696268** | **0.29347825** | **0.75559837** | **1** | **1590** | **tags=23%, list=12%, signal=26%** |
| **RESPIRATORY_GASEOUS_EXCHANGE** | **RESPIRATORY_GASEOUS_EXCHANGE** | | **14** | **0.3233816** | **1.0664935** | **0.3457249** | **0.7583328** | **1** | **2782** | **tags=43%, list=21%, signal=54%** |
| **PROTEIN_TARGETING** | **PROTEIN_TARGETING** | | **92** | **0.2114465** | **1.065741** | **0.23728813** | **0.75433975** | **1** | **1849** | **tags=18%, list=14%, signal=21%** |
| **POSITIVE_REGULATION_OF_LYMPHOCYTE_ACTIVATION** | **POSITIVE_REGULATION_OF_LYMPHOCYTE_ACTIVATION** | | **22** | **0.2955552** | **1.065194** | **0.3233831** | **0.74976635** | **1** | **1126** | **tags=23%, list=8%, signal=25%** |
| **POSITIVE_REGULATION_OF_CELL_CYCLE** | **POSITIVE_REGULATION_OF_CELL_CYCLE** | | **15** | **0.3256417** | **1.0640311** | **0.3529412** | **0.747135** | **1** | **4347** | **tags=53%, list=33%, signal=79%** |
| **NUCLEAR_IMPORT** | **NUCLEAR_IMPORT** | | **43** | **0.23925675** | **1.0618166** | **0.26086956** | **0.7469824** | **1** | **1849** | **tags=21%, list=14%, signal=24%** |
| **REGULATION_OF_TRANSFERASE_ACTIVITY** | **REGULATION_OF_TRANSFERASE_ACTIVITY** | | **133** | **0.19292754** | **1.0603619** | **0.32142857** | **0.74509656** | **1** | **2984** | **tags=29%, list=22%, signal=37%** |
| **PROTEIN_IMPORT_INTO_NUCLEUS** | **PROTEIN_IMPORT_INTO_NUCLEUS** | | **42** | **0.24300624** | **1.0599** | **0.3283582** | **0.7404741** | **1** | **1849** | **tags=21%, list=14%, signal=25%** |
| **COENZYME_BIOSYNTHETIC_PROCESS** | **COENZYME_BIOSYNTHETIC_PROCESS** | | **10** | **0.3832087** | **1.0548873** | **0.36206895** | **0.74829596** | **1** | **786** | **tags=20%, list=6%, signal=21%** |
| **INDUCTION_OF_APOPTOSIS_BY_INTRACELLULAR_SIGNALS** | **INDUCTION_OF_APOPTOSIS_BY_INTRACELLULAR_SIGNALS** | | **21** | **0.29670647** | **1.0547469** | **0.3761468** | **0.7427798** | **1** | **2390** | **tags=38%, list=18%, signal=46%** |
| **N_ACETYLGLUCOSAMINE_METABOLIC_PROCESS** | **N_ACETYLGLUCOSAMINE_METABOLIC_PROCESS** | | **11** | **0.35005355** | **1.0510253** | **0.3556338** | **0.74750626** | **1** | **478** | **tags=18%, list=4%, signal=19%** |
| **NUCLEAR_EXPORT** | **NUCLEAR_EXPORT** | | **25** | **0.29004484** | **1.0489042** | **0.374269** | **0.7477693** | **1** | **3308** | **tags=40%, list=25%, signal=53%** |
| **PEPTIDYL_TYROSINE_MODIFICATION** | **PEPTIDYL_TYROSINE_MODIFICATION** | | **23** | **0.28136718** | **1.0474225** | **0.36633664** | **0.74577713** | **1** | **2871** | **tags=43%, list=22%, signal=55%** |
| **CHROMOSOME_CONDENSATION** | **CHROMOSOME_CONDENSATION** | | **7** | **0.40780407** | **1.0402876** | **0.37333333** | **0.7595481** | **1** | **2482** | **tags=43%, list=19%, signal=53%** |
| **REGULATION_OF_HYDROLASE_ACTIVITY** | **REGULATION_OF_HYDROLASE_ACTIVITY** | | **63** | **0.2152856** | **1.040118** | **0.38297874** | **0.7542717** | **1** | **979** | **tags=16%, list=7%, signal=17%** |
| **ISOPRENOID_METABOLIC_PROCESS** | **ISOPRENOID_METABOLIC_PROCESS** | | **10** | **0.3652679** | **1.0346384** | **0.39716312** | **0.7640033** | **1** | **903** | **tags=20%, list=7%, signal=21%** |
| **PROTEOGLYCAN_METABOLIC_PROCESS** | **PROTEOGLYCAN_METABOLIC_PROCESS** | | **14** | **0.33225724** | **1.0314536** | **0.38518518** | **0.7664471** | **1** | **999** | **tags=29%, list=7%, signal=31%** |
| **POSITIVE_REGULATION_OF_HYDROLASE_ACTIVITY** | **POSITIVE_REGULATION_OF_HYDROLASE_ACTIVITY** | | **44** | **0.23977089** | **1.0291057** | **0.3923077** | **0.7666735** | **1** | **2798** | **tags=39%, list=21%, signal=49%** |
| **MEMBRANE_ORGANIZATION_AND_BIOGENESIS** | **MEMBRANE_ORGANIZATION_AND_BIOGENESIS** | | **117** | **0.18799263** | **1.0277418** | **0.3255814** | **0.764904** | **1** | **1186** | **tags=13%, list=9%, signal=14%** |
| **G1_S_TRANSITION_OF_MITOTIC_CELL_CYCLE** | **G1_S_TRANSITION_OF_MITOTIC_CELL_CYCLE** | | **24** | **0.27870378** | **1.0200922** | **0.3768116** | **0.7803189** | **1** | **3167** | **tags=42%, list=24%, signal=55%** |
| **PROTEIN_AMINO_ACID_DEPHOSPHORYLATION** | **PROTEIN_AMINO_ACID_DEPHOSPHORYLATION** | | **60** | **0.21358545** | **1.013454** | **0.46226415** | **0.7934092** | **1** | **2635** | **tags=32%, list=20%, signal=39%** |
| **REGULATION_OF_TRANSCRIPTION_FROM_RNA_POLYMERASE_II_PROMOTER** | **REGULATION_OF_TRANSCRIPTION_FROM_RNA_POLYMERASE_II_PROMOTER** | | **246** | **0.17857543** | **1.012045** | **0.5** | **0.79195493** | **1** | **1446** | **tags=16%, list=11%, signal=18%** |
| **GOLGI_VESICLE_TRANSPORT** | **GOLGI_VESICLE_TRANSPORT** | | **41** | **0.23387185** | **1.0114522** | **0.48427674** | **0.78825444** | **1** | **2788** | **tags=27%, list=21%, signal=34%** |
| **ESTABLISHMENT_OF_PROTEIN_LOCALIZATION** | **ESTABLISHMENT_OF_PROTEIN_LOCALIZATION** | | **156** | **0.18075119** | **1.0075479** | **0.39285713** | **0.79336274** | **1** | **1849** | **tags=17%, list=14%, signal=19%** |
| **REGULATION_OF_PROTEIN_KINASE_ACTIVITY** | **REGULATION_OF_PROTEIN_KINASE_ACTIVITY** | | **130** | **0.1897444** | **1.0046884** | **0.48387095** | **0.7961407** | **1** | **2984** | **tags=29%, list=22%, signal=37%** |
| **REGULATION_OF_NUCLEOCYTOPLASMIC_TRANSPORT** | **REGULATION_OF_NUCLEOCYTOPLASMIC_TRANSPORT** | | **18** | **0.30032524** | **1.0015173** | **0.43555555** | **0.7998305** | **1** | **1849** | **tags=22%, list=14%, signal=26%** |
| **NEGATIVE_REGULATION_OF_IMMUNE_SYSTEM_PROCESS** | **NEGATIVE_REGULATION_OF_IMMUNE_SYSTEM_PROCESS** | | **13** | **0.32411718** | **0.9954954** | **0.4375** | **0.811451** | **1** | **979** | **tags=23%, list=7%, signal=25%** |
| **REGULATION_OF_NUCLEOBASENUCLEOSIDENUCLEOTIDE_AND_NUCLEIC_ACID_METABOLIC_PROCESS** | **REGULATION_OF_NUCLEOBASENUCLEOSIDENUCLEOTIDE_AND_NUCLEIC_ACID_METABOLIC_PROCESS** | | **495** | **0.15613821** | **0.9946645** | **1** | **0.8082859** | **1** | **1480** | **tags=15%, list=11%, signal=17%** |
| **REGULATION_OF_JAK_STAT_CASCADE** | **REGULATION_OF_JAK_STAT_CASCADE** | | **7** | **0.39609185** | **0.98920065** | **0.42559522** | **0.8185512** | **1** | **3564** | **tags=57%, list=27%, signal=78%** |
| **MITOCHONDRIAL_TRANSPORT** | **MITOCHONDRIAL_TRANSPORT** | | **18** | **0.2938435** | **0.9889576** | **0.45348838** | **0.81380826** | **1** | **9411** | **tags=100%, list=71%, signal=340%** |
| **UNFOLDED_PROTEIN_RESPONSE** | **UNFOLDED_PROTEIN_RESPONSE** | | **8** | **0.3818456** | **0.98742205** | **0.44193548** | **0.8123459** | **1** | **888** | **tags=25%, list=7%, signal=27%** |
| **PROTEIN_TRANSPORT** | **PROTEIN_TRANSPORT** | | **134** | **0.18177803** | **0.9873843** | **0.4347826** | **0.80700415** | **1** | **1849** | **tags=16%, list=14%, signal=18%** |
| **RESPONSE_TO_HORMONE_STIMULUS** | **RESPONSE_TO_HORMONE_STIMULUS** | | **25** | **0.2545313** | **0.9867802** | **0.486911** | **0.80302036** | **1** | **1588** | **tags=24%, list=12%, signal=27%** |
| **ER_NUCLEAR_SIGNALING_PATHWAY** | **ER_NUCLEAR_SIGNALING_PATHWAY** | | **13** | **0.32267368** | **0.9864979** | **0.4347826** | **0.798471** | **1** | **2339** | **tags=31%, list=18%, signal=37%** |
| **MICROTUBULE_BASED_MOVEMENT** | **MICROTUBULE_BASED_MOVEMENT** | | **16** | **0.29411453** | **0.9809839** | **0.46120688** | **0.80853367** | **1** | **1153** | **tags=25%, list=9%, signal=27%** |
| **REGULATION_OF_KINASE_ACTIVITY** | **REGULATION_OF_KINASE_ACTIVITY** | | **131** | **0.18952772** | **0.9788652** | **0.5** | **0.8089828** | **1** | **2984** | **tags=29%, list=22%, signal=37%** |
| **MICROTUBULE_BASED_PROCESS** | **MICROTUBULE_BASED_PROCESS** | | **72** | **0.20293759** | **0.97766316** | **0.54285717** | **0.8070631** | **1** | **1333** | **tags=14%, list=10%, signal=15%** |
| **TRICARBOXYLIC_ACID_CYCLE_INTERMEDIATE_METABOLIC_PROCESS** | **TRICARBOXYLIC_ACID_CYCLE_INTERMEDIATE_METABOLIC_PROCESS** | | **10** | **0.35785556** | **0.9765194** | **0.46478873** | **0.8045861** | **1** | **3447** | **tags=50%, list=26%, signal=67%** |
| **NEGATIVE_REGULATION_OF_CELLULAR_PROTEIN_METABOLIC_PROCESS** | **NEGATIVE_REGULATION_OF_CELLULAR_PROTEIN_METABOLIC_PROCESS** | | **39** | **0.22975563** | **0.9737296** | **0.4935065** | **0.80692697** | **1** | **2562** | **tags=31%, list=19%, signal=38%** |
| **RESPONSE_TO_OXIDATIVE_STRESS** | **RESPONSE_TO_OXIDATIVE_STRESS** | | **38** | **0.23023328** | **0.97054935** | **0.4964029** | **0.81006134** | **1** | **1895** | **tags=24%, list=14%, signal=28%** |
| **MACROMOLECULE_LOCALIZATION** | **MACROMOLECULE_LOCALIZATION** | | **190** | **0.1767002** | **0.96734077** | **0.6923077** | **0.8131846** | **1** | **1849** | **tags=16%, list=14%, signal=18%** |
| **NEGATIVE_REGULATION_OF_CATALYTIC_ACTIVITY** | **NEGATIVE_REGULATION_OF_CATALYTIC_ACTIVITY** | | **62** | **0.20999455** | **0.96628076** | **0.46** | **0.811097** | **1** | **2579** | **tags=35%, list=19%, signal=44%** |
| **GLUCOSAMINE_METABOLIC_PROCESS** | **GLUCOSAMINE_METABOLIC_PROCESS** | | **12** | **0.31547722** | **0.9632611** | **0.49615383** | **0.8142757** | **1** | **478** | **tags=17%, list=4%, signal=17%** |
| **CELLULAR_MACROMOLECULE_CATABOLIC_PROCESS** | **CELLULAR_MACROMOLECULE_CATABOLIC_PROCESS** | | **90** | **0.18951604** | **0.96262205** | **0.6** | **0.81108207** | **1** | **1693** | **tags=20%, list=13%, signal=23%** |
| **PROTEOGLYCAN_BIOSYNTHETIC_PROCESS** | **PROTEOGLYCAN_BIOSYNTHETIC_PROCESS** | | **10** | **0.34340987** | **0.9558145** | **0.49659863** | **0.8237726** | **1** | **725** | **tags=30%, list=5%, signal=32%** |
| **REGULATION_OF_TYROSINE_PHOSPHORYLATION_OF_STAT_PROTEIN** | **REGULATION_OF_TYROSINE_PHOSPHORYLATION_OF_STAT_PROTEIN** | | **6** | **0.40756434** | **0.9550258** | **0.5133531** | **0.8205256** | **1** | **2871** | **tags=50%, list=22%, signal=64%** |
| **PROTEIN_LOCALIZATION** | **PROTEIN_LOCALIZATION** | | **174** | **0.17668355** | **0.95260197** | **0.5** | **0.8219867** | **1** | **1849** | **tags=16%, list=14%, signal=18%** |
| **REGULATION_OF_IMMUNE_EFFECTOR_PROCESS** | **REGULATION_OF_IMMUNE_EFFECTOR_PROCESS** | | **13** | **0.30512872** | **0.95178777** | **0.5034965** | **0.81907** | **1** | **364** | **tags=15%, list=3%, signal=16%** |
| **POSITIVE_REGULATION_OF_RESPONSE_TO_STIMULUS** | **POSITIVE_REGULATION_OF_RESPONSE_TO_STIMULUS** | | **33** | **0.238495** | **0.9453685** | **0.52662724** | **0.8310656** | **1** | **3443** | **tags=48%, list=26%, signal=65%** |
| **PROTEIN_DNA_COMPLEX_ASSEMBLY** | **PROTEIN_DNA_COMPLEX_ASSEMBLY** | | **36** | **0.22262298** | **0.9446641** | **0.56395346** | **0.8278955** | **1** | **2371** | **tags=28%, list=18%, signal=34%** |
| **CELLULAR_COMPONENT_DISASSEMBLY** | **CELLULAR_COMPONENT_DISASSEMBLY** | | **30** | **0.2363382** | **0.94441384** | **0.5764706** | **0.82346785** | **1** | **1041** | **tags=20%, list=8%, signal=22%** |
| **POSITIVE_REGULATION_OF_DEFENSE_RESPONSE** | **POSITIVE_REGULATION_OF_DEFENSE_RESPONSE** | | **8** | **0.35640484** | **0.9410956** | **0.5062893** | **0.8266021** | **1** | **3377** | **tags=63%, list=25%, signal=84%** |
| **PROTEIN_IMPORT** | **PROTEIN_IMPORT** | | **55** | **0.19926636** | **0.93707776** | **0.6146789** | **0.83155334** | **1** | **1849** | **tags=18%, list=14%, signal=21%** |
| **CYTOSKELETON_DEPENDENT_INTRACELLULAR_TRANSPORT** | **CYTOSKELETON_DEPENDENT_INTRACELLULAR_TRANSPORT** | | **26** | **0.24129413** | **0.9336613** | **0.6145833** | **0.83512634** | **1** | **1153** | **tags=19%, list=9%, signal=21%** |
| **COFACTOR_BIOSYNTHETIC_PROCESS** | **COFACTOR_BIOSYNTHETIC_PROCESS** | | **21** | **0.25090644** | **0.93322986** | **0.5695067** | **0.8314568** | **1** | **1164** | **tags=19%, list=9%, signal=21%** |
| **MEMBRANE_FUSION** | **MEMBRANE_FUSION** | | **26** | **0.23890176** | **0.9268595** | **0.5399061** | **0.84300697** | **1** | **732** | **tags=12%, list=5%, signal=12%** |
| **COENZYME_METABOLIC_PROCESS** | **COENZYME_METABOLIC_PROCESS** | | **33** | **0.2299061** | **0.9174506** | **0.654321** | **0.8617809** | **1** | **1215** | **tags=18%, list=9%, signal=20%** |
| **PATTERN_SPECIFICATION_PROCESS** | **PATTERN_SPECIFICATION_PROCESS** | | **27** | **0.24225241** | **0.9162441** | **0.6** | **0.8600492** | **1** | **2062** | **tags=33%, list=15%, signal=39%** |
| **PEPTIDYL_TYROSINE_PHOSPHORYLATION** | **PEPTIDYL_TYROSINE_PHOSPHORYLATION** | | **21** | **0.2462111** | **0.9128968** | **0.5849057** | **0.8629157** | **1** | **1943** | **tags=29%, list=15%, signal=33%** |
| **INTERFERON_GAMMA_BIOSYNTHETIC_PROCESS** | **INTERFERON_GAMMA_BIOSYNTHETIC_PROCESS** | | **9** | **0.33763** | **0.9122207** | **0.5017544** | **0.85962427** | **1** | **3640** | **tags=67%, list=27%, signal=92%** |
| **MONOCARBOXYLIC_ACID_TRANSPORT** | **MONOCARBOXYLIC_ACID_TRANSPORT** | | **10** | **0.32001454** | **0.91112953** | **0.58862877** | **0.8580806** | **1** | **474** | **tags=20%, list=4%, signal=21%** |
| **RESPONSE_TO_STEROID_HORMONE_STIMULUS** | **RESPONSE_TO_STEROID_HORMONE_STIMULUS** | | **8** | **0.34800574** | **0.9102265** | **0.5695793** | **0.85559356** | **1** | **8687** | **tags=100%, list=65%, signal=287%** |
| **TRNA_METABOLIC_PROCESS** | **TRNA_METABOLIC_PROCESS** | | **15** | **0.27940488** | **0.909551** | **0.57258064** | **0.85251296** | **1** | **911** | **tags=13%, list=7%, signal=14%** |
| **DEPHOSPHORYLATION** | **DEPHOSPHORYLATION** | | **67** | **0.1919242** | **0.9089317** | **0.75609756** | **0.8496534** | **1** | **2635** | **tags=28%, list=20%, signal=35%** |
| **INDUCTION_OF_APOPTOSIS_BY_EXTRACELLULAR_SIGNALS** | **INDUCTION_OF_APOPTOSIS_BY_EXTRACELLULAR_SIGNALS** | | **25** | **0.23759018** | **0.90876955** | **0.65581393** | **0.84536374** | **1** | **10161** | **tags=100%, list=76%, signal=421%** |
| **REGULATION_OF_CELLULAR_COMPONENT_ORGANIZATION_AND_BIOGENESIS** | **REGULATION_OF_CELLULAR_COMPONENT_ORGANIZATION_AND_BIOGENESIS** | | **93** | **0.17458828** | **0.9079067** | **0.6885246** | **0.8429627** | **1** | **2371** | **tags=25%, list=18%, signal=30%** |
| **NOTCH_SIGNALING_PATHWAY** | **NOTCH_SIGNALING_PATHWAY** | | **8** | **0.35301754** | **0.90639955** | **0.5498282** | **0.84190094** | **1** | **2380** | **tags=50%, list=18%, signal=61%** |
| **COFACTOR_METABOLIC_PROCESS** | **COFACTOR_METABOLIC_PROCESS** | | **47** | **0.20112152** | **0.9013124** | **0.6515151** | **0.8495625** | **1** | **1215** | **tags=17%, list=9%, signal=19%** |
| **NEGATIVE_REGULATION_OF_ANGIOGENESIS** | **NEGATIVE_REGULATION_OF_ANGIOGENESIS** | | **11** | **0.31204423** | **0.9001649** | **0.56643355** | **0.8479453** | **1** | **255** | **tags=18%, list=2%, signal=19%** |
| **POSITIVE_REGULATION_OF_IMMUNE_SYSTEM_PROCESS** | **POSITIVE_REGULATION_OF_IMMUNE_SYSTEM_PROCESS** | | **41** | **0.2091688** | **0.8992681** | **0.6451613** | **0.84566003** | **1** | **3813** | **tags=49%, list=29%, signal=68%** |
| **CELLULAR_LOCALIZATION** | **CELLULAR_LOCALIZATION** | | **310** | **0.14376804** | **0.88777494** | **1** | **0.8676574** | **1** | **1989** | **tags=16%, list=15%, signal=19%** |
| **REGULATION_OF_IMMUNE_SYSTEM_PROCESS** | **REGULATION_OF_IMMUNE_SYSTEM_PROCESS** | | **55** | **0.1938261** | **0.88663113** | **0.72131145** | **0.8656675** | **1** | **3849** | **tags=45%, list=29%, signal=64%** |
| **CYTOKINESIS** | **CYTOKINESIS** | | **16** | **0.2675107** | **0.88073957** | **0.6571429** | **0.8748565** | **1** | **3833** | **tags=50%, list=29%, signal=70%** |
| **NEGATIVE_REGULATION_OF_CELL_PROLIFERATION** | **NEGATIVE_REGULATION_OF_CELL_PROLIFERATION** | | **138** | **0.16126205** | **0.88045806** | **0.8** | **0.87097687** | **1** | **2274** | **tags=22%, list=17%, signal=26%** |
| **MITOTIC_CELL_CYCLE_CHECKPOINT** | **MITOTIC_CELL_CYCLE_CHECKPOINT** | | **19** | **0.25246847** | **0.8798463** | **0.60262007** | **0.8678647** | **1** | **3116** | **tags=42%, list=23%, signal=55%** |
| **MACROMOLECULE_CATABOLIC_PROCESS** | **MACROMOLECULE_CATABOLIC_PROCESS** | | **118** | **0.16949713** | **0.8787334** | **0.8055556** | **0.86585546** | **1** | **1405** | **tags=16%, list=11%, signal=18%** |
| **REGULATION_OF_PROTEIN_IMPORT_INTO_NUCLEUS** | **REGULATION_OF_PROTEIN_IMPORT_INTO_NUCLEUS** | | **13** | **0.28068623** | **0.87811136** | **0.61627907** | **0.8629171** | **1** | **1849** | **tags=23%, list=14%, signal=27%** |
| **PROTEIN_RNA_COMPLEX_ASSEMBLY** | **PROTEIN_RNA_COMPLEX_ASSEMBLY** | | **37** | **0.20781481** | **0.8770655** | **0.7176471** | **0.86079425** | **1** | **1987** | **tags=24%, list=15%, signal=29%** |
| **TRNA_PROCESSING** | **TRNA_PROCESSING** | | **8** | **0.3300858** | **0.8705849** | **0.5889571** | **0.8705947** | **1** | **3850** | **tags=63%, list=29%, signal=88%** |
| **REGULATION_OF_MITOSIS** | **REGULATION_OF_MITOSIS** | | **34** | **0.20649567** | **0.86318564** | **0.70198673** | **0.88233** | **1** | **3116** | **tags=38%, list=23%, signal=50%** |
| **ESTABLISHMENT_OF_ORGANELLE_LOCALIZATION** | **ESTABLISHMENT_OF_ORGANELLE_LOCALIZATION** | | **15** | **0.25973243** | **0.8611124** | **0.65863454** | **0.8827945** | **1** | **9864** | **tags=100%, list=74%, signal=385%** |
| **ACTIN_POLYMERIZATION_AND_OR_DEPOLYMERIZATION** | **ACTIN_POLYMERIZATION_AND_OR_DEPOLYMERIZATION** | | **18** | **0.24855904** | **0.8605859** | **0.7105263** | **0.87943774** | **1** | **1454** | **tags=22%, list=11%, signal=25%** |
| **RECEPTOR_MEDIATED_ENDOCYTOSIS** | **RECEPTOR_MEDIATED_ENDOCYTOSIS** | | **30** | **0.21338865** | **0.8354236** | **0.7839506** | **0.9275495** | **1** | **1186** | **tags=13%, list=9%, signal=15%** |
| **LEUKOCYTE_DIFFERENTIATION** | **LEUKOCYTE_DIFFERENTIATION** | | **34** | **0.20247345** | **0.83276135** | **0.775641** | **0.9286024** | **1** | **402** | **tags=12%, list=3%, signal=12%** |
| **RNA_EXPORT_FROM_NUCLEUS** | **RNA_EXPORT_FROM_NUCLEUS** | | **16** | **0.2557141** | **0.82893187** | **0.7037037** | **0.9317964** | **1** | **3308** | **tags=38%, list=25%, signal=50%** |
| **NEGATIVE_REGULATION_OF_CELLULAR_COMPONENT_ORGANIZATION_AND_BIOGENESIS** | **NEGATIVE_REGULATION_OF_CELLULAR_COMPONENT_ORGANIZATION_AND_BIOGENESIS** | | **25** | **0.22322693** | **0.8262333** | **0.73364484** | **0.9327828** | **1** | **2371** | **tags=32%, list=18%, signal=39%** |
| **CELL_CYCLE_GO_0007049** | **CELL_CYCLE_GO_0007049** | | **260** | **0.14767043** | **0.82438856** | **0.8** | **0.93212205** | **1** | **3121** | **tags=27%, list=23%, signal=34%** |
| **ACTIVATION_OF_IMMUNE_RESPONSE** | **ACTIVATION_OF_IMMUNE_RESPONSE** | | **13** | **0.26720354** | **0.8231818** | **0.67634857** | **0.92995334** | **1** | **1995** | **tags=31%, list=15%, signal=36%** |
| **RHO_PROTEIN_SIGNAL_TRANSDUCTION** | **RHO_PROTEIN_SIGNAL_TRANSDUCTION** | | **30** | **0.20784748** | **0.82177067** | **0.76744187** | **0.92837423** | **1** | **1592** | **tags=20%, list=12%, signal=23%** |
| **INTERCELLULAR_JUNCTION_ASSEMBLY_AND_MAINTENANCE** | **INTERCELLULAR_JUNCTION_ASSEMBLY_AND_MAINTENANCE** | | **11** | **0.2804897** | **0.8197136** | **0.7183099** | **0.9281806** | **1** | **2643** | **tags=36%, list=20%, signal=45%** |
| **SPERMATID_DIFFERENTIATION** | **SPERMATID_DIFFERENTIATION** | | **8** | **0.30941454** | **0.8189419** | **0.6743421** | **0.92526937** | **1** | **45** | **tags=13%, list=0%, signal=13%** |
| **RAS_PROTEIN_SIGNAL_TRANSDUCTION** | **RAS_PROTEIN_SIGNAL_TRANSDUCTION** | | **54** | **0.17693979** | **0.81643575** | **0.8545455** | **0.92586213** | **1** | **1592** | **tags=15%, list=12%, signal=17%** |
| **NUCLEOSOME_ASSEMBLY** | **NUCLEOSOME_ASSEMBLY** | | **10** | **0.27256098** | **0.80947196** | **0.72043014** | **0.9347493** | **1** | **2371** | **tags=30%, list=18%, signal=36%** |
| **INTRA_GOLGI_VESICLE_MEDIATED_TRANSPORT** | **INTRA_GOLGI_VESICLE_MEDIATED_TRANSPORT** | | **11** | **0.27196664** | **0.8089124** | **0.74703556** | **0.9314395** | **1** | **3138** | **tags=45%, list=24%, signal=59%** |
| **VESICLE_LOCALIZATION** | **VESICLE_LOCALIZATION** | | **10** | **0.28576** | **0.8061285** | **0.6815069** | **0.9322905** | **1** | **118** | **tags=10%, list=1%, signal=10%** |
| **INTERCELLULAR_JUNCTION_ASSEMBLY** | **INTERCELLULAR_JUNCTION_ASSEMBLY** | | **9** | **0.30059826** | **0.8057729** | **0.6944444** | **0.92870545** | **1** | **2643** | **tags=44%, list=20%, signal=55%** |
| **REGULATION_OF_ENDOTHELIAL_CELL_PROLIFERATION** | **REGULATION_OF_ENDOTHELIAL_CELL_PROLIFERATION** | | **7** | **0.3246957** | **0.79730445** | **0.69325155** | **0.93951243** | **1** | **8997** | **tags=100%, list=68%, signal=308%** |
| **OXYGEN_AND_REACTIVE_OXYGEN_SPECIES_METABOLIC_PROCESS** | **OXYGEN_AND_REACTIVE_OXYGEN_SPECIES_METABOLIC_PROCESS** | | **18** | **0.23259501** | **0.79638255** | **0.81124496** | **0.9367236** | **1** | **833** | **tags=22%, list=6%, signal=24%** |
| **PROTEIN_TARGETING_TO_MITOCHONDRION** | **PROTEIN_TARGETING_TO_MITOCHONDRION** | | **9** | **0.29364476** | **0.7895103** | **0.737931** | **0.94430625** | **1** | **9411** | **tags=100%, list=71%, signal=340%** |
| **AMINO_SUGAR_METABOLIC_PROCESS** | **AMINO_SUGAR_METABOLIC_PROCESS** | | **15** | **0.24796048** | **0.78804684** | **0.75330395** | **0.9427918** | **1** | **478** | **tags=13%, list=4%, signal=14%** |
| **REGULATION_OF_ORGANELLE_ORGANIZATION_AND_BIOGENESIS** | **REGULATION_OF_ORGANELLE_ORGANIZATION_AND_BIOGENESIS** | | **34** | **0.18549481** | **0.77632403** | **0.86** | **0.9584447** | **1** | **1333** | **tags=15%, list=10%, signal=16%** |
| **NUCLEOTIDE_METABOLIC_PROCESS** | **NUCLEOTIDE_METABOLIC_PROCESS** | | **34** | **0.18924202** | **0.7737868** | **0.88414633** | **0.95818835** | **1** | **2773** | **tags=29%, list=21%, signal=37%** |
| **EPIDERMAL_GROWTH_FACTOR_RECEPTOR_SIGNALING_PATHWAY** | **EPIDERMAL_GROWTH_FACTOR_RECEPTOR_SIGNALING_PATHWAY** | | **18** | **0.22985582** | **0.7664867** | **0.8214286** | **0.96545625** | **1** | **3449** | **tags=39%, list=26%, signal=52%** |
| **REGULATION_OF_TRANSLATIONAL_INITIATION** | **REGULATION_OF_TRANSLATIONAL_INITIATION** | | **15** | **0.23721807** | **0.7622282** | **0.78333336** | **0.9679864** | **1** | **2339** | **tags=27%, list=18%, signal=32%** |
| **DNA_DAMAGE_CHECKPOINT** | **DNA_DAMAGE_CHECKPOINT** | | **16** | **0.22934161** | **0.76192856** | **0.8313253** | **0.9641217** | **1** | **2986** | **tags=38%, list=22%, signal=48%** |
| **RIBONUCLEOPROTEIN_COMPLEX_BIOGENESIS_AND_ASSEMBLY** | **RIBONUCLEOPROTEIN_COMPLEX_BIOGENESIS_AND_ASSEMBLY** | | **47** | **0.16862799** | **0.7609552** | **0.88785046** | **0.96146756** | **1** | **1648** | **tags=19%, list=12%, signal=22%** |
| **VESICLE_MEDIATED_TRANSPORT** | **VESICLE_MEDIATED_TRANSPORT** | | **169** | **0.14127214** | **0.75715125** | **0.9285714** | **0.9630516** | **1** | **3001** | **tags=27%, list=23%, signal=34%** |
| **SPERMATID_DEVELOPMENT** | **SPERMATID_DEVELOPMENT** | | **7** | **0.31004706** | **0.7571341** | **0.787037** | **0.9588264** | **1** | **45** | **tags=14%, list=0%, signal=14%** |
| **REGULATION_OF_HOMEOSTATIC_PROCESS** | **REGULATION_OF_HOMEOSTATIC_PROCESS** | | **12** | **0.25187075** | **0.7545343** | **0.7789855** | **0.9588607** | **1** | **979** | **tags=17%, list=7%, signal=18%** |
| **INNATE_IMMUNE_RESPONSE** | **INNATE_IMMUNE_RESPONSE** | | **17** | **0.22404392** | **0.75173855** | **0.84549356** | **0.9588099** | **1** | **1472** | **tags=24%, list=11%, signal=26%** |
| **REGULATION_OF_CYTOSKELETON_ORGANIZATION_AND_BIOGENESIS** | **REGULATION_OF_CYTOSKELETON_ORGANIZATION_AND_BIOGENESIS** | | **25** | **0.19951446** | **0.7474605** | **0.8556701** | **0.961208** | **1** | **1333** | **tags=16%, list=10%, signal=18%** |
| **PEPTIDYL_AMINO_ACID_MODIFICATION** | **PEPTIDYL_AMINO_ACID_MODIFICATION** | | **46** | **0.1674166** | **0.7437791** | **0.91240877** | **0.962358** | **1** | **2949** | **tags=28%, list=22%, signal=36%** |
| **REGULATION_OF_TRANSFORMING_GROWTH_FACTOR_BETA_RECEPTOR_SIGNALING_PATHWAY** | **REGULATION_OF_TRANSFORMING_GROWTH_FACTOR_BETA_RECEPTOR_SIGNALING_PATHWAY** | | **12** | **0.2467436** | **0.7431541** | **0.79422385** | **0.9591286** | **1** | **3991** | **tags=67%, list=30%, signal=95%** |
| **NEGATIVE_REGULATION_OF_PHOSPHATE_METABOLIC_PROCESS** | **NEGATIVE_REGULATION_OF_PHOSPHATE_METABOLIC_PROCESS** | | **12** | **0.24499197** | **0.7422582** | **0.8151815** | **0.95610386** | **1** | **3015** | **tags=42%, list=23%, signal=54%** |
| **REGULATION_OF_INTRACELLULAR_TRANSPORT** | **REGULATION_OF_INTRACELLULAR_TRANSPORT** | | **21** | **0.20307802** | **0.73820835** | **0.85714287** | **0.9575192** | **1** | **1849** | **tags=19%, list=14%, signal=22%** |
| **SMALL_GTPASE_MEDIATED_SIGNAL_TRANSDUCTION** | **SMALL_GTPASE_MEDIATED_SIGNAL_TRANSDUCTION** | | **73** | **0.15106733** | **0.73581934** | **0.9480519** | **0.95672935** | **1** | **1592** | **tags=15%, list=12%, signal=17%** |
| **INTERFERON_GAMMA_PRODUCTION** | **INTERFERON_GAMMA_PRODUCTION** | | **11** | **0.25023797** | **0.7339834** | **0.80144405** | **0.9550273** | **1** | **3640** | **tags=64%, list=27%, signal=87%** |
| **NUCLEOBASENUCLEOSIDE_AND_NUCLEOTIDE_METABOLIC_PROCESS** | **NUCLEOBASENUCLEOSIDE_AND_NUCLEOTIDE_METABOLIC_PROCESS** | | **44** | **0.16454801** | **0.7219666** | **0.92253524** | **0.9661762** | **1** | **2834** | **tags=27%, list=21%, signal=35%** |
| **ACTIN_FILAMENT_BASED_MOVEMENT** | **ACTIN_FILAMENT_BASED_MOVEMENT** | | **10** | **0.2612126** | **0.71724117** | **0.81395346** | **0.968255** | **1** | **9843** | **tags=100%, list=74%, signal=383%** |
| **ACTIVATION_OF_MAPK_ACTIVITY** | **ACTIVATION_OF_MAPK_ACTIVITY** | | **33** | **0.1810203** | **0.7133751** | **0.9177215** | **0.9690017** | **1** | **3892** | **tags=42%, list=29%, signal=60%** |
| **NEGATIVE_REGULATION_OF_DNA_REPLICATION** | **NEGATIVE_REGULATION_OF_DNA_REPLICATION** | | **11** | **0.24996243** | **0.711502** | **0.8333333** | **0.9672172** | **1** | **9993** | **tags=100%, list=75%, signal=400%** |
| **REGULATION_OF_INTERFERON_GAMMA_BIOSYNTHETIC_PROCESS** | **REGULATION_OF_INTERFERON_GAMMA_BIOSYNTHETIC_PROCESS** | | **8** | **0.26677826** | **0.7044955** | **0.8363636** | **0.97161686** | **1** | **3640** | **tags=63%, list=27%, signal=86%** |
| **PROTEIN_TARGETING_TO_MEMBRANE** | **PROTEIN_TARGETING_TO_MEMBRANE** | | **8** | **0.26575524** | **0.7036066** | **0.8654434** | **0.9685288** | **1** | **9782** | **tags=100%, list=73%, signal=376%** |
| **INTRACELLULAR_RECEPTOR_MEDIATED_SIGNALING_PATHWAY** | **INTRACELLULAR_RECEPTOR_MEDIATED_SIGNALING_PATHWAY** | | **10** | **0.23969072** | **0.6920625** | **0.8869258** | **0.9774265** | **1** | **271** | **tags=10%, list=2%, signal=10%** |
| **MAINTENANCE_OF_PROTEIN_LOCALIZATION** | **MAINTENANCE_OF_PROTEIN_LOCALIZATION** | | **10** | **0.25182176** | **0.6891674** | **0.8689655** | **0.976574** | **1** | **9968** | **tags=100%, list=75%, signal=397%** |
| **POSITIVE_REGULATION_OF_IMMUNE_RESPONSE** | **POSITIVE_REGULATION_OF_IMMUNE_RESPONSE** | | **22** | **0.19087508** | **0.6871395** | **0.90384614** | **0.97463346** | **1** | **3729** | **tags=45%, list=28%, signal=63%** |
| **MAINTENANCE_OF_CELLULAR_PROTEIN_LOCALIZATION** | **MAINTENANCE_OF_CELLULAR_PROTEIN_LOCALIZATION** | | **9** | **0.25180286** | **0.6858906** | **0.8900344** | **0.97215986** | **1** | **9968** | **tags=100%, list=75%, signal=397%** |
| **CELL_DIVISION** | **CELL_DIVISION** | | **18** | **0.20591559** | **0.6817037** | **0.93191487** | **0.9728637** | **1** | **3301** | **tags=39%, list=25%, signal=52%** |
| **STEROID_HORMONE_RECEPTOR_SIGNALING_PATHWAY** | **STEROID_HORMONE_RECEPTOR_SIGNALING_PATHWAY** | | **10** | **0.23969072** | **0.6793559** | **0.87762237** | **0.9712818** | **1** | **271** | **tags=10%, list=2%, signal=10%** |
| **PROTEIN_EXPORT_FROM_NUCLEUS** | **PROTEIN_EXPORT_FROM_NUCLEUS** | | **8** | **0.259438** | **0.6742104** | **0.873065** | **0.9720159** | **1** | **2238** | **tags=25%, list=17%, signal=30%** |
| **INTERLEUKIN_2_PRODUCTION** | **INTERLEUKIN_2_PRODUCTION** | | **10** | **0.24062766** | **0.6701272** | **0.86315787** | **0.9720997** | **1** | **1961** | **tags=30%, list=15%, signal=35%** |
| **NUCLEOTIDE_SUGAR_METABOLIC_PROCESS** | **NUCLEOTIDE_SUGAR_METABOLIC_PROCESS** | | **8** | **0.25493875** | **0.6683147** | **0.86378735** | **0.96979475** | **1** | **9926** | **tags=100%, list=75%, signal=392%** |
| **ER_TO_GOLGI_VESICLE_MEDIATED_TRANSPORT** | **ER_TO_GOLGI_VESICLE_MEDIATED_TRANSPORT** | | **12** | **0.21271312** | **0.64238536** | **0.9222222** | **0.9870613** | **1** | **10489** | **tags=100%, list=79%, signal=470%** |
| **DNA_INTEGRITY_CHECKPOINT** | **DNA_INTEGRITY_CHECKPOINT** | | **19** | **0.18316524** | **0.6306129** | **0.9583333** | **0.9917607** | **1** | **2390** | **tags=26%, list=18%, signal=32%** |
| **POSITIVE_REGULATION_OF_MAP_KINASE_ACTIVITY** | **POSITIVE_REGULATION_OF_MAP_KINASE_ACTIVITY** | | **39** | **0.15273929** | **0.628441** | **0.9932432** | **0.98932266** | **1** | **3892** | **tags=41%, list=29%, signal=58%** |
| **NEGATIVE_REGULATION_OF_PHOSPHORYLATION** | **NEGATIVE_REGULATION_OF_PHOSPHORYLATION** | | **11** | **0.20341933** | **0.5839802** | **0.969112** | **1** | **1** | **3015** | **tags=36%, list=23%, signal=47%** |
| **MITOCHONDRION_ORGANIZATION_AND_BIOGENESIS** | **MITOCHONDRION_ORGANIZATION_AND_BIOGENESIS** | | **43** | **0.13076618** | **0.5804773** | **0.99264705** | **1** | **1** | **10396** | **tags=98%, list=78%, signal=443%** |
| **REGULATION_OF_GENE_SPECIFIC_TRANSCRIPTION** | **REGULATION_OF_GENE_SPECIFIC_TRANSCRIPTION** | | **9** | **0.20883407** | **0.57054204** | **0.92068964** | **1** | **1** | **10540** | **tags=100%, list=79%, signal=479%** |
| **REGULATION_OF_CHROMOSOME_ORGANIZATION_AND_BIOGENESIS** | **REGULATION_OF_CHROMOSOME_ORGANIZATION_AND_BIOGENESIS** | | **9** | **0.20091967** | **0.5573396** | **0.9756098** | **1** | **1** | **3443** | **tags=33%, list=26%, signal=45%** |
| **REGULATION_OF_DNA_REPLICATION** | **REGULATION_OF_DNA_REPLICATION** | | **17** | **0.16874628** | **0.55531746** | **0.9906977** | **1** | **1** | **2986** | **tags=29%, list=22%, signal=38%** |
| **ELECTRON_TRANSPORT_GO_0006118** | **ELECTRON_TRANSPORT_GO_0006118** | | **50** | **0.12003458** | **0.54612005** | **1** | **1** | **1** | **3262** | **tags=30%, list=24%, signal=40%** |
| **DNA_MODIFICATION** | **DNA_MODIFICATION** | | **9** | **0.19681492** | **0.5378671** | **0.97651005** | **1** | **1** | **10700** | **tags=100%, list=80%, signal=508%** |
| **ENDOTHELIAL_CELL_PROLIFERATION** | **ENDOTHELIAL_CELL_PROLIFERATION** | | **9** | **0.18930279** | **0.5203445** | **0.9877301** | **1** | **1** | **10800** | **tags=100%, list=81%, signal=528%** |
| **ACTIVATION_OF_JNK_ACTIVITY** | **ACTIVATION_OF_JNK_ACTIVITY** | | **14** | **0.14999709** | **0.47990263** | **0.9915254** | **1** | **1** | **2949** | **tags=36%, list=22%, signal=46%** |
| **LYSOSOME_ORGANIZATION_AND_BIOGENESIS** | **LYSOSOME_ORGANIZATION_AND_BIOGENESIS** | | **11** | **0.1627359** | **0.4775907** | **0.9968553** | **1** | **1** | **1950** | **tags=18%, list=15%, signal=21%** |
| **VACUOLE_ORGANIZATION_AND_BIOGENESIS** | **VACUOLE_ORGANIZATION_AND_BIOGENESIS** | | **12** | **0.1547824** | **0.46147513** | **0.99590164** | **0.99838257** | **1** | **11260** | **tags=100%, list=85%, signal=646%** |
| **RNA_METABOLIC_PROCESS** | **RNA_METABOLIC_PROCESS** | | **683** | **0.14740267** |  |  | **1** | **0** | **1484** | **tags=15%, list=11%, signal=16%** |
| **ESTABLISHMENT_OF_CELLULAR_LOCALIZATION** | **ESTABLISHMENT_OF_CELLULAR_LOCALIZATION** | | **297** | **0.14377902** |  |  | **1** | **0** | **2035** | **tags=16%, list=15%, signal=19%** |
| **NEGATIVE_REGULATION_OF_CELLULAR_PROCESS** | **NEGATIVE_REGULATION_OF_CELLULAR_PROCESS** | | **538** | **0.16656235** |  |  | **1** | **0** | **1550** | **tags=17%, list=12%, signal=18%** |
| **REGULATION_OF_DEVELOPMENTAL_PROCESS** | **REGULATION_OF_DEVELOPMENTAL_PROCESS** | | **369** | **0.17598392** |  |  | **1** | **0** | **2390** | **tags=25%, list=18%, signal=30%** |
| **RESPONSE_TO_STRESS** | **RESPONSE_TO_STRESS** | | **444** | **0.15081592** |  |  | **1** | **0** | **2393** | **tags=23%, list=18%, signal=27%** |
| **POSITIVE_REGULATION_OF_CELLULAR_PROCESS** | **POSITIVE_REGULATION_OF_CELLULAR_PROCESS** | | **540** | **0.14748625** |  |  | **1** | **0** | **2473** | **tags=24%, list=19%, signal=28%** |
| **NEGATIVE_REGULATION_OF_BIOLOGICAL_PROCESS** | **NEGATIVE_REGULATION_OF_BIOLOGICAL_PROCESS** | | **563** | **0.16627108** |  |  | **1** | **0** | **1641** | **tags=17%, list=12%, signal=19%** |
| **REGULATION_OF_TRANSCRIPTION** | **REGULATION_OF_TRANSCRIPTION** | | **456** | **0.16689916** |  |  | **1** | **0** | **1480** | **tags=16%, list=11%, signal=17%** |

**Table S2.4.- GSE10072: Never smokers, tumor, enriched in women**

| **NAME** | **GS<br> follow link to MSigDB** | **GS DETAILS** | **SIZE** | **ES** | **NES** | **NOM p-val** | **FDR q-val** | **FWER p-val** | **RANK AT MAX** | **LEADING EDGE** |
| --- | --- | --- | --- | --- | --- | --- | --- | --- | --- | --- |
| **SYNAPTIC_TRANSMISSION** | **SYNAPTIC_TRANSMISSION** | **Details ...** | **152** | **-0.6140814** | **-2.3484855** | **0** | **0** | **0** | **2447** | **tags=54%, list=18%, signal=65%** |
| **TRANSMISSION_OF_NERVE_IMPULSE** | **TRANSMISSION_OF_NERVE_IMPULSE** | **Details ...** | **165** | **-0.60542166** | **-2.3446777** | **0** | **0** | **0** | **2447** | **tags=52%, list=18%, signal=63%** |
| **NEUROLOGICAL_SYSTEM_PROCESS** | **NEUROLOGICAL_SYSTEM_PROCESS** | **Details ...** | **324** | **-0.5579385** | **-2.2206452** | **0** | **0** | **0** | **3721** | **tags=58%, list=28%, signal=79%** |
| **SYSTEM_PROCESS** | **SYSTEM_PROCESS** | **Details ...** | **492** | **-0.51091015** | **-2.0686402** | **0** | **1.34E-04** | **0.002** | **3775** | **tags=52%, list=28%, signal=70%** |
| **SENSORY_PERCEPTION** | **SENSORY_PERCEPTION** | **Details ...** | **161** | **-0.53093594** | **-2.0306063** | **0** | **4.35E-04** | **0.007** | **4372** | **tags=64%, list=33%, signal=94%** |
| **G_PROTEIN_COUPLED_RECEPTOR_PROTEIN_SIGNALING_PATHWAY** | **G_PROTEIN_COUPLED_RECEPTOR_PROTEIN_SIGNALING_PATHWAY** | **Details ...** | **290** | **-0.50902253** | **-2.0267584** | **0** | **4.04E-04** | **0.007** | **3404** | **tags=51%, list=26%, signal=67%** |
| **SECOND_MESSENGER_MEDIATED_SIGNALING** | **SECOND_MESSENGER_MEDIATED_SIGNALING** | **Details ...** | **141** | **-0.5099848** | **-1.9475483** | **0** | **0.00253919** | **0.046** | **2585** | **tags=45%, list=19%, signal=56%** |
| **METAL_ION_TRANSPORT** | **METAL_ION_TRANSPORT** | **Details ...** | **98** | **-0.5255114** | **-1.9393737** | **0** | **0.00278632** | **0.054** | **2351** | **tags=44%, list=18%, signal=53%** |
| **G_PROTEIN_SIGNALING_COUPLED_TO_CYCLIC_NUCLEOTIDE_SECOND_MESSENGER** | **G_PROTEIN_SIGNALING_COUPLED_TO_CYCLIC_NUCLEOTIDE_SECOND_MESSENGER** | **Details ...** | **97** | **-0.5273099** | **-1.9367644** | **0** | **0.00276599** | **0.056** | **2585** | **tags=53%, list=19%, signal=65%** |
| **MONOVALENT_INORGANIC_CATION_TRANSPORT** | **MONOVALENT_INORGANIC_CATION_TRANSPORT** | **Details ...** | **78** | **-0.5347439** | **-1.9291455** | **0** | **0.00306295** | **0.065** | **2351** | **tags=45%, list=18%, signal=54%** |
| **CYCLIC_NUCLEOTIDE_MEDIATED_SIGNALING** | **CYCLIC_NUCLEOTIDE_MEDIATED_SIGNALING** | **Details ...** | **99** | **-0.52451044** | **-1.9277773** | **0** | **0.00298777** | **0.067** | **2585** | **tags=52%, list=19%, signal=63%** |
| **POTASSIUM_ION_TRANSPORT** | **POTASSIUM_ION_TRANSPORT** | **Details ...** | **51** | **-0.57338595** | **-1.923634** | **0** | **0.0029177** | **0.069** | **4409** | **tags=69%, list=33%, signal=102%** |
| **CATION_TRANSPORT** | **CATION_TRANSPORT** | **Details ...** | **124** | **-0.50670594** | **-1.9127631** | **0** | **0.00336039** | **0.082** | **2351** | **tags=40%, list=18%, signal=49%** |
| **ION_TRANSPORT** | **ION_TRANSPORT** | **Details ...** | **159** | **-0.48786017** | **-1.8705652** | **0** | **0.00620186** | **0.148** | **2500** | **tags=40%, list=19%, signal=48%** |
| **DETECTION_OF_STIMULUS** | **DETECTION_OF_STIMULUS** | **Details ...** | **33** | **-0.59576106** | **-1.8510481** | **0** | **0.00818555** | **0.201** | **3139** | **tags=58%, list=24%, signal=75%** |
| **CELL_CELL_SIGNALING** | **CELL_CELL_SIGNALING** | **Details ...** | **366** | **-0.46070346** | **-1.8501779** | **0** | **0.00797819** | **0.203** | **3555** | **tags=48%, list=27%, signal=63%** |
| **DETECTION_OF_STIMULUS_INVOLVED_IN_SENSORY_PERCEPTION** | **DETECTION_OF_STIMULUS_INVOLVED_IN_SENSORY_PERCEPTION** | **Details ...** | **15** | **-0.6697625** | **-1.7734745** | **0.00134048** | **0.02531813** | **0.542** | **2378** | **tags=60%, list=18%, signal=73%** |
| **DETECTION_OF_EXTERNAL_STIMULUS** | **DETECTION_OF_EXTERNAL_STIMULUS** | **Details ...** | **16** | **-0.65535235** | **-1.7392827** | **0.00390625** | **0.03943457** | **0.707** | **576** | **tags=44%, list=4%, signal=46%** |
| **NEUROPEPTIDE_SIGNALING_PATHWAY** | **NEUROPEPTIDE_SIGNALING_PATHWAY** | **Details ...** | **12** | **-0.68667006** | **-1.7380254** | **0.00681199** | **0.03842327** | **0.712** | **3305** | **tags=92%, list=25%, signal=122%** |
| **FEEDING_BEHAVIOR** | **FEEDING_BEHAVIOR** | **Details ...** | **19** | **-0.611118** | **-1.7164495** | **0.0013245** | **0.04984865** | **0.819** | **2753** | **tags=63%, list=21%, signal=79%** |
| **SEXUAL_REPRODUCTION** | **SEXUAL_REPRODUCTION** | **Details ...** | **107** | **-0.46257848** | **-1.7145666** | **0** | **0.04897233** | **0.824** | **2725** | **tags=39%, list=20%, signal=49%** |
| **DETECTION_OF_ABIOTIC_STIMULUS** | **DETECTION_OF_ABIOTIC_STIMULUS** | **Details ...** | **16** | **-0.6436028** | **-1.7113012** | **0.0063857** | **0.04947479** | **0.838** | **2251** | **tags=56%, list=17%, signal=68%** |
| **REGULATION_OF_JNK_CASCADE** | **REGULATION_OF_JNK_CASCADE** | **Details ...** | **11** | **-0.6802908** | **-1.6760927** | **0.00282087** | **0.07323068** | **0.942** | **2153** | **tags=55%, list=16%, signal=65%** |
| **SODIUM_ION_TRANSPORT** | **SODIUM_ION_TRANSPORT** | **Details ...** | **15** | **-0.6446389** | **-1.67391** | **0.00526316** | **0.07253804** | **0.946** | **2111** | **tags=67%, list=16%, signal=79%** |
| **REGULATION_OF_NEUROTRANSMITTER_LEVELS** | **REGULATION_OF_NEUROTRANSMITTER_LEVELS** | **Details ...** | **22** | **-0.56826013** | **-1.6529877** | **0.0075662** | **0.08913752** | **0.971** | **3650** | **tags=59%, list=27%, signal=81%** |
| **PROTEIN_AMINO_ACID_ADP_RIBOSYLATION** | **PROTEIN_AMINO_ACID_ADP_RIBOSYLATION** | **Details ...** | **10** | **-0.69101614** | **-1.6502982** | **0.00706215** | **0.089396** | **0.974** | **2591** | **tags=50%, list=19%, signal=62%** |
| **EXCRETION** | **EXCRETION** | **Details ...** | **35** | **-0.52112275** | **-1.6465489** | **0.00357569** | **0.09059408** | **0.976** | **3837** | **tags=49%, list=29%, signal=68%** |
| **REGULATED_SECRETORY_PATHWAY** | **REGULATED_SECRETORY_PATHWAY** | **Details ...** | **12** | **-0.6507542** | **-1.6357207** | **0.01470588** | **0.10007808** | **0.989** | **2722** | **tags=58%, list=20%, signal=73%** |
| **HORMONE_METABOLIC_PROCESS** | **HORMONE_METABOLIC_PROCESS** | **Details ...** | **27** | **-0.54856116** | **-1.6314927** | **0.01322115** | **0.10170916** | **0.99** | **2134** | **tags=41%, list=16%, signal=48%** |
| **RESPONSE_TO_LIGHT_STIMULUS** | **RESPONSE_TO_LIGHT_STIMULUS** | **Details ...** | **40** | **-0.4933069** | **-1.6177248** | **0.00578035** | **0.11518613** | **0.996** | **4084** | **tags=58%, list=31%, signal=83%** |
| **PHOSPHOINOSITIDE_MEDIATED_SIGNALING** | **PHOSPHOINOSITIDE_MEDIATED_SIGNALING** | **Details ...** | **41** | **-0.489986** | **-1.60742** | **0.00584795** | **0.12553366** | **0.998** | **2076** | **tags=37%, list=16%, signal=43%** |
| **CENTROSOME_CYCLE** | **CENTROSOME_CYCLE** | **Details ...** | **7** | **-0.7427624** | **-1.6039596** | **0.02040816** | **0.12711805** | **0.999** | **1285** | **tags=29%, list=10%, signal=32%** |
| **G_PROTEIN_SIGNALING_COUPLED_TO_IP3_SECOND_MESSENGERPHOSPHOLIPASE_C_ACTIVATING** | **G_PROTEIN_SIGNALING_COUPLED_TO_IP3_SECOND_MESSENGERPHOSPHOLIPASE_C_ACTIVATING** | **Details ...** | **38** | **-0.49796033** | **-1.6028098** | **0.00821596** | **0.12540545** | **0.999** | **3135** | **tags=45%, list=24%, signal=58%** |
| **DETECTION_OF_CHEMICAL_STIMULUS** | **DETECTION_OF_CHEMICAL_STIMULUS** | **Details ...** | **13** | **-0.629344** | **-1.6002069** | **0.01355014** | **0.12562373** | **0.999** | **3139** | **tags=62%, list=24%, signal=80%** |
| **G_PROTEIN_SIGNALING_COUPLED_TO_CAMP_NUCLEOTIDE_SECOND_MESSENGER** | **G_PROTEIN_SIGNALING_COUPLED_TO_CAMP_NUCLEOTIDE_SECOND_MESSENGER** | **Details ...** | **63** | **-0.45019108** | **-1.598435** | **0.0032967** | **0.12504111** | **0.999** | **2585** | **tags=46%, list=19%, signal=57%** |
| **G_PROTEIN_SIGNALING_ADENYLATE_CYCLASE_ACTIVATING_PATHWAY** | **G_PROTEIN_SIGNALING_ADENYLATE_CYCLASE_ACTIVATING_PATHWAY** | **Details ...** | **25** | **-0.5330791** | **-1.5949942** | **0.01743462** | **0.12654734** | **0.999** | **2522** | **tags=52%, list=19%, signal=64%** |
| **FEMALE_GAMETE_GENERATION** | **FEMALE_GAMETE_GENERATION** | **Details ...** | **15** | **-0.5971757** | **-1.5943254** | **0.02136182** | **0.1245641** | **0.999** | **3250** | **tags=67%, list=24%, signal=88%** |
| **ANION_TRANSPORT** | **ANION_TRANSPORT** | **Details ...** | **27** | **-0.5277842** | **-1.5912076** | **0.01824817** | **0.1255809** | **0.999** | **3274** | **tags=48%, list=25%, signal=64%** |
| **NEUROTRANSMITTER_SECRETION** | **NEUROTRANSMITTER_SECRETION** | **Details ...** | **11** | **-0.65899456** | **-1.5887923** | **0.01494565** | **0.12553452** | **0.999** | **2722** | **tags=64%, list=20%, signal=80%** |
| **CELL_SURFACE_RECEPTOR_LINKED_SIGNAL_TRANSDUCTION_GO_0007166** | **CELL_SURFACE_RECEPTOR_LINKED_SIGNAL_TRANSDUCTION_GO_0007166** | **Details ...** | **554** | **-0.39094016** | **-1.5847944** | **0** | **0.12821138** | **1** | **3305** | **tags=37%, list=25%, signal=48%** |
| **FATTY_ACID_OXIDATION** | **FATTY_ACID_OXIDATION** | **Details ...** | **16** | **-0.5889042** | **-1.5820928** | **0.02389937** | **0.12949985** | **1** | **2461** | **tags=38%, list=18%, signal=46%** |
| **REGULATION_OF_LIPID_METABOLIC_PROCESS** | **REGULATION_OF_LIPID_METABOLIC_PROCESS** | **Details ...** | **11** | **-0.65797836** | **-1.581156** | **0.02489627** | **0.12807578** | **1** | **2402** | **tags=55%, list=18%, signal=66%** |
| **PHOTOTRANSDUCTION** | **PHOTOTRANSDUCTION** | **Details ...** | **11** | **-0.6401672** | **-1.5792383** | **0.02253521** | **0.12798385** | **1** | **2251** | **tags=55%, list=17%, signal=66%** |
| **STRIATED_MUSCLE_CONTRACTION_GO_0006941** | **STRIATED_MUSCLE_CONTRACTION_GO_0006941** | **Details ...** | **14** | **-0.6148049** | **-1.5787624** | **0.01619433** | **0.12631942** | **1** | **2896** | **tags=50%, list=22%, signal=64%** |
| **REGULATION_OF_MAPKKK_CASCADE** | **REGULATION_OF_MAPKKK_CASCADE** | **Details ...** | **18** | **-0.5596291** | **-1.5649925** | **0.02933674** | **0.14173596** | **1** | **2727** | **tags=44%, list=20%, signal=56%** |
| **GLUTAMATE_SIGNALING_PATHWAY** | **GLUTAMATE_SIGNALING_PATHWAY** | **Details ...** | **17** | **-0.57409865** | **-1.5564071** | **0.01728723** | **0.15129922** | **1** | **3650** | **tags=65%, list=27%, signal=89%** |
| **CAMP_MEDIATED_SIGNALING** | **CAMP_MEDIATED_SIGNALING** | **Details ...** | **64** | **-0.44618782** | **-1.5481001** | **0.00553097** | **0.16140115** | **1** | **2585** | **tags=45%, list=19%, signal=56%** |
| **REPRODUCTION** | **REPRODUCTION** | **Details ...** | **212** | **-0.3963756** | **-1.5471638** | **0** | **0.16012298** | **1** | **2725** | **tags=34%, list=20%, signal=42%** |
| **REPRODUCTIVE_PROCESS** | **REPRODUCTIVE_PROCESS** | **Details ...** | **134** | **-0.405399** | **-1.5425692** | **0.0030896** | **0.16481955** | **1** | **2725** | **tags=35%, list=20%, signal=44%** |
| **CALCIUM_ION_TRANSPORT** | **CALCIUM_ION_TRANSPORT** | **Details ...** | **23** | **-0.5384701** | **-1.5422264** | **0.02114428** | **0.1624368** | **1** | **4064** | **tags=61%, list=31%, signal=87%** |
| **NERVOUS_SYSTEM_DEVELOPMENT** | **NERVOUS_SYSTEM_DEVELOPMENT** | | **314** | **-0.38520402** | **-1.5372907** | **0** | **0.16719994** | **1** | **3483** | **tags=38%, list=26%, signal=51%** |
| **INORGANIC_ANION_TRANSPORT** | **INORGANIC_ANION_TRANSPORT** | | **14** | **-0.5862188** | **-1.5350641** | **0.03703704** | **0.16747995** | **1** | **2990** | **tags=50%, list=22%, signal=64%** |
| **AMINE_TRANSPORT** | **AMINE_TRANSPORT** | | **35** | **-0.47809055** | **-1.5330176** | **0.01902497** | **0.16784593** | **1** | **4444** | **tags=51%, list=33%, signal=77%** |
| **REGULATION_OF_CELL_MIGRATION** | **REGULATION_OF_CELL_MIGRATION** | | **23** | **-0.51175165** | **-1.5241783** | **0.0302267** | **0.17932066** | **1** | **1626** | **tags=26%, list=12%, signal=30%** |
| **EPITHELIAL_CELL_DIFFERENTIATION** | **EPITHELIAL_CELL_DIFFERENTIATION** | | **9** | **-0.6556792** | **-1.5227876** | **0.03506311** | **0.17868607** | **1** | **980** | **tags=33%, list=7%, signal=36%** |
| **AROMATIC_COMPOUND_METABOLIC_PROCESS** | **AROMATIC_COMPOUND_METABOLIC_PROCESS** | | **25** | **-0.51004195** | **-1.5221397** | **0.02442003** | **0.17686903** | **1** | **3551** | **tags=40%, list=27%, signal=54%** |
| **DI___TRI_VALENT_INORGANIC_CATION_TRANSPORT** | **DI___TRI_VALENT_INORGANIC_CATION_TRANSPORT** | | **27** | **-0.508046** | **-1.5172191** | **0.01330109** | **0.18214926** | **1** | **4064** | **tags=52%, list=31%, signal=74%** |
| **SPERM_MOTILITY** | **SPERM_MOTILITY** | | **9** | **-0.6464998** | **-1.5118448** | **0.04539007** | **0.1880424** | **1** | **4215** | **tags=89%, list=32%, signal=130%** |
| **GAMETE_GENERATION** | **GAMETE_GENERATION** | | **88** | **-0.4165897** | **-1.5107358** | **0.00845666** | **0.18722901** | **1** | **2725** | **tags=36%, list=20%, signal=45%** |
| **ENERGY_RESERVE_METABOLIC_PROCESS** | **ENERGY_RESERVE_METABOLIC_PROCESS** | | **15** | **-0.5632675** | **-1.5060537** | **0.03050398** | **0.19214013** | **1** | **1647** | **tags=33%, list=12%, signal=38%** |
| **CELLULAR_MORPHOGENESIS_DURING_DIFFERENTIATION** | **CELLULAR_MORPHOGENESIS_DURING_DIFFERENTIATION** | | **40** | **-0.46139252** | **-1.5041742** | **0.02097902** | **0.19245666** | **1** | **2020** | **tags=33%, list=15%, signal=38%** |
| **CENTRAL_NERVOUS_SYSTEM_DEVELOPMENT** | **CENTRAL_NERVOUS_SYSTEM_DEVELOPMENT** | | **99** | **-0.4116801** | **-1.5020785** | **0.00844773** | **0.19301072** | **1** | **2701** | **tags=33%, list=20%, signal=42%** |
| **RESPONSE_TO_NUTRIENT_LEVELS** | **RESPONSE_TO_NUTRIENT_LEVELS** | | **25** | **-0.49789742** | **-1.499771** | **0.03362391** | **0.19433764** | **1** | **5049** | **tags=68%, list=38%, signal=109%** |
| **XENOBIOTIC_METABOLIC_PROCESS** | **XENOBIOTIC_METABOLIC_PROCESS** | | **8** | **-0.67017853** | **-1.497913** | **0.04011461** | **0.1947816** | **1** | **3824** | **tags=88%, list=29%, signal=123%** |
| **STEROID_METABOLIC_PROCESS** | **STEROID_METABOLIC_PROCESS** | | **63** | **-0.42143714** | **-1.4799249** | **0.0173913** | **0.22418164** | **1** | **2929** | **tags=32%, list=22%, signal=41%** |
| **ADENYLATE_CYCLASE_ACTIVATION** | **ADENYLATE_CYCLASE_ACTIVATION** | | **19** | **-0.5278822** | **-1.4715409** | **0.0465718** | **0.23738766** | **1** | **2522** | **tags=53%, list=19%, signal=65%** |
| **HEART_DEVELOPMENT** | **HEART_DEVELOPMENT** | | **32** | **-0.4592296** | **-1.4555762** | **0.03026635** | **0.26744244** | **1** | **3498** | **tags=41%, list=26%, signal=55%** |
| **MORPHOGENESIS_OF_AN_EPITHELIUM** | **MORPHOGENESIS_OF_AN_EPITHELIUM** | | **14** | **-0.5572582** | **-1.4526571** | **0.06258693** | **0.2703011** | **1** | **980** | **tags=21%, list=7%, signal=23%** |
| **G1_PHASE_OF_MITOTIC_CELL_CYCLE** | **G1_PHASE_OF_MITOTIC_CELL_CYCLE** | | **12** | **-0.56812793** | **-1.4447293** | **0.05986395** | **0.28432572** | **1** | **2450** | **tags=42%, list=18%, signal=51%** |
| **PHOSPHOLIPASE_C_ACTIVATION** | **PHOSPHOLIPASE_C_ACTIVATION** | | **11** | **-0.58583546** | **-1.4412116** | **0.04807692** | **0.2882193** | **1** | **1987** | **tags=45%, list=15%, signal=53%** |
| **AXONOGENESIS** | **AXONOGENESIS** | | **35** | **-0.4581437** | **-1.4411633** | **0.04486423** | **0.28467238** | **1** | **2020** | **tags=34%, list=15%, signal=40%** |
| **POSITIVE_REGULATION_OF_MAPKKK_CASCADE** | **POSITIVE_REGULATION_OF_MAPKKK_CASCADE** | | **9** | **-0.6117797** | **-1.4409373** | **0.06478873** | **0.2816109** | **1** | **2153** | **tags=44%, list=16%, signal=53%** |
| **CELL_FATE_COMMITMENT** | **CELL_FATE_COMMITMENT** | | **13** | **-0.5559672** | **-1.4338322** | **0.07368421** | **0.29390702** | **1** | **2589** | **tags=54%, list=19%, signal=67%** |
| **ECTODERM_DEVELOPMENT** | **ECTODERM_DEVELOPMENT** | | **73** | **-0.4021513** | **-1.4335679** | **0.02835333** | **0.29092407** | **1** | **3701** | **tags=45%, list=28%, signal=62%** |
| **SECRETION** | **SECRETION** |  | **149** | **-0.36946815** | **-1.4196118** | **0.00514933** | **0.32021978** | **1** | **3837** | **tags=39%, list=29%, signal=54%** |
| **RESPONSE_TO_RADIATION** | **RESPONSE_TO_RADIATION** | | **52** | **-0.41842538** | **-1.4136078** | **0.04446979** | **0.33068565** | **1** | **4084** | **tags=50%, list=31%, signal=72%** |
| **STEROID_BIOSYNTHETIC_PROCESS** | **STEROID_BIOSYNTHETIC_PROCESS** | | **22** | **-0.4924803** | **-1.4134574** | **0.06741573** | **0.32714865** | **1** | **4940** | **tags=64%, list=37%, signal=101%** |
| **REGULATION_OF_CELL_MORPHOGENESIS** | **REGULATION_OF_CELL_MORPHOGENESIS** | | **9** | **-0.6204368** | **-1.4014769** | **0.0466761** | **0.3536619** | **1** | **2786** | **tags=56%, list=21%, signal=70%** |
| **REGULATION_OF_CATABOLIC_PROCESS** | **REGULATION_OF_CATABOLIC_PROCESS** | | **15** | **-0.5339711** | **-1.400551** | **0.07362784** | **0.3518714** | **1** | **5158** | **tags=73%, list=39%, signal=120%** |
| **EPIDERMIS_DEVELOPMENT** | **EPIDERMIS_DEVELOPMENT** | | **65** | **-0.39574558** | **-1.4003135** | **0.04225352** | **0.34852704** | **1** | **3701** | **tags=45%, list=28%, signal=61%** |
| **RESPONSE_TO_XENOBIOTIC_STIMULUS** | **RESPONSE_TO_XENOBIOTIC_STIMULUS** | | **9** | **-0.6144117** | **-1.3978617** | **0.09116809** | **0.35091856** | **1** | **3824** | **tags=78%, list=29%, signal=109%** |
| **BODY_FLUID_SECRETION** | **BODY_FLUID_SECRETION** | | **10** | **-0.5821004** | **-1.3964804** | **0.07460545** | **0.35045633** | **1** | **4743** | **tags=80%, list=36%, signal=124%** |
| **AMINE_CATABOLIC_PROCESS** | **AMINE_CATABOLIC_PROCESS** | | **24** | **-0.47375396** | **-1.3851619** | **0.0730198** | **0.37552643** | **1** | **5009** | **tags=58%, list=38%, signal=93%** |
| **REGULATION_OF_HEART_CONTRACTION** | **REGULATION_OF_HEART_CONTRACTION** | | **24** | **-0.46780112** | **-1.382304** | **0.09228824** | **0.37929407** | **1** | **4154** | **tags=54%, list=31%, signal=79%** |
| **NEURITE_DEVELOPMENT** | **NEURITE_DEVELOPMENT** | | **43** | **-0.42216566** | **-1.3803389** | **0.06053551** | **0.38021865** | **1** | **2020** | **tags=30%, list=15%, signal=36%** |
| **GENE_SILENCING** | **GENE_SILENCING** | | **9** | **-0.5914876** | **-1.3801675** | **0.09916201** | **0.37665683** | **1** | **1656** | **tags=33%, list=12%, signal=38%** |
| **ALCOHOL_METABOLIC_PROCESS** | **ALCOHOL_METABOLIC_PROCESS** | | **81** | **-0.38070938** | **-1.3767893** | **0.03407881** | **0.38179845** | **1** | **3426** | **tags=30%, list=26%, signal=40%** |
| **TISSUE_DEVELOPMENT** | **TISSUE_DEVELOPMENT** | | **122** | **-0.36448467** | **-1.3750172** | **0.03448276** | **0.382305** | **1** | **3757** | **tags=43%, list=28%, signal=60%** |
| **REGULATION_OF_MYELOID_CELL_DIFFERENTIATION** | **REGULATION_OF_MYELOID_CELL_DIFFERENTIATION** | | **18** | **-0.49444157** | **-1.3742174** | **0.09819122** | **0.38057598** | **1** | **2522** | **tags=39%, list=19%, signal=48%** |
| **GLUCOSE_METABOLIC_PROCESS** | **GLUCOSE_METABOLIC_PROCESS** | | **26** | **-0.45614013** | **-1.3724933** | **0.08301887** | **0.38129234** | **1** | **5318** | **tags=54%, list=40%, signal=89%** |
| **RESPONSE_TO_EXTRACELLULAR_STIMULUS** | **RESPONSE_TO_EXTRACELLULAR_STIMULUS** | | **27** | **-0.4603539** | **-1.3723476** | **0.09282178** | **0.37780124** | **1** | **5119** | **tags=67%, list=38%, signal=108%** |
| **MONOVALENT_INORGANIC_CATION_HOMEOSTASIS** | **MONOVALENT_INORGANIC_CATION_HOMEOSTASIS** | | **13** | **-0.53592724** | **-1.3666843** | **0.10519125** | **0.3887802** | **1** | **2062** | **tags=31%, list=15%, signal=36%** |
| **LIPID_CATABOLIC_PROCESS** | **LIPID_CATABOLIC_PROCESS** | | **31** | **-0.43573987** | **-1.3627981** | **0.08460634** | **0.39538747** | **1** | **2461** | **tags=35%, list=18%, signal=43%** |
| **NEUTRAL_AMINO_ACID_TRANSPORT** | **NEUTRAL_AMINO_ACID_TRANSPORT** | | **10** | **-0.56832254** | **-1.3622183** | **0.10578279** | **0.39295062** | **1** | **4206** | **tags=70%, list=32%, signal=102%** |
| **DEVELOPMENT_OF_PRIMARY_SEXUAL_CHARACTERISTICS** | **DEVELOPMENT_OF_PRIMARY_SEXUAL_CHARACTERISTICS** | | **25** | **-0.4537678** | **-1.3572565** | **0.09938651** | **0.4023928** | **1** | **3636** | **tags=52%, list=27%, signal=71%** |
| **EXOCYTOSIS** | **EXOCYTOSIS** |  | **21** | **-0.47077522** | **-1.3563924** | **0.10237204** | **0.40080523** | **1** | **2897** | **tags=38%, list=22%, signal=49%** |
| **DIGESTION** | **DIGESTION** |  | **40** | **-0.41153896** | **-1.3440264** | **0.08244994** | **0.43212655** | **1** | **3372** | **tags=40%, list=25%, signal=53%** |
| **NEGATIVE_REGULATION_OF_CELL_MIGRATION** | **NEGATIVE_REGULATION_OF_CELL_MIGRATION** | | **14** | **-0.5202685** | **-1.3396672** | **0.10728477** | **0.4405287** | **1** | **936** | **tags=21%, list=7%, signal=23%** |
| **MALE_GONAD_DEVELOPMENT** | **MALE_GONAD_DEVELOPMENT** | | **11** | **-0.5468218** | **-1.3393742** | **0.12707183** | **0.43722194** | **1** | **5024** | **tags=73%, list=38%, signal=117%** |
| **AMINO_ACID_CATABOLIC_PROCESS** | **AMINO_ACID_CATABOLIC_PROCESS** | | **22** | **-0.4642775** | **-1.33662** | **0.11304348** | **0.44098043** | **1** | **4564** | **tags=50%, list=34%, signal=76%** |
| **ANATOMICAL_STRUCTURE_DEVELOPMENT** | **ANATOMICAL_STRUCTURE_DEVELOPMENT** | | **868** | **-0.32528087** | **-1.336119** | **0** | **0.43846765** | **1** | **3505** | **tags=34%, list=26%, signal=43%** |
| **RESPONSE_TO_ABIOTIC_STIMULUS** | **RESPONSE_TO_ABIOTIC_STIMULUS** | | **79** | **-0.36678725** | **-1.325769** | **0.07221007** | **0.46492255** | **1** | **4084** | **tags=44%, list=31%, signal=64%** |
| **NEUROGENESIS** | **NEUROGENESIS** | | **76** | **-0.36548316** | **-1.3254553** | **0.07011867** | **0.46174198** | **1** | **2061** | **tags=26%, list=15%, signal=31%** |
| **NEURON_DIFFERENTIATION** | **NEURON_DIFFERENTIATION** | | **59** | **-0.38797322** | **-1.3240299** | **0.07078651** | **0.46178746** | **1** | **2020** | **tags=27%, list=15%, signal=32%** |
| **TISSUE_REMODELING** | **TISSUE_REMODELING** | | **28** | **-0.4271727** | **-1.3236558** | **0.12392638** | **0.45893586** | **1** | **2522** | **tags=36%, list=19%, signal=44%** |
| **REGULATION_OF_PH** | **REGULATION_OF_PH** | | **12** | **-0.5364316** | **-1.3189204** | **0.12845305** | **0.46898854** | **1** | **2062** | **tags=33%, list=15%, signal=39%** |
| **SYSTEM_DEVELOPMENT** | **SYSTEM_DEVELOPMENT** | | **736** | **-0.32382706** | **-1.3179138** | **0** | **0.4680597** | **1** | **3505** | **tags=34%, list=26%, signal=44%** |
| **BONE_REMODELING** | **BONE_REMODELING** | | **27** | **-0.43547857** | **-1.3172013** | **0.11590629** | **0.466143** | **1** | **2522** | **tags=37%, list=19%, signal=46%** |
| **REGULATION_OF_CELL_SHAPE** | **REGULATION_OF_CELL_SHAPE** | | **8** | **-0.5869134** | **-1.3162252** | **0.13969572** | **0.46506533** | **1** | **2786** | **tags=50%, list=21%, signal=63%** |
| **PIGMENT_BIOSYNTHETIC_PROCESS** | **PIGMENT_BIOSYNTHETIC_PROCESS** | | **17** | **-0.4816741** | **-1.3161415** | **0.11238825** | **0.46140584** | **1** | **3551** | **tags=47%, list=27%, signal=64%** |
| **PROTEIN_STABILIZATION** | **PROTEIN_STABILIZATION** | | **8** | **-0.6087569** | **-1.3156157** | **0.13772455** | **0.45905423** | **1** | **15** | **tags=13%, list=0%, signal=13%** |
| **RESPONSE_TO_NUTRIENT** | **RESPONSE_TO_NUTRIENT** | | **16** | **-0.4870451** | **-1.3154299** | **0.13499345** | **0.4557276** | **1** | **5034** | **tags=69%, list=38%, signal=110%** |
| **GENERATION_OF_A_SIGNAL_INVOLVED_IN_CELL_CELL_SIGNALING** | **GENERATION_OF_A_SIGNAL_INVOLVED_IN_CELL_CELL_SIGNALING** | | **24** | **-0.44139913** | **-1.3091145** | **0.13740458** | **0.47024137** | **1** | **3139** | **tags=46%, list=24%, signal=60%** |
| **REGULATION_OF_BLOOD_PRESSURE** | **REGULATION_OF_BLOOD_PRESSURE** | | **20** | **-0.45995992** | **-1.3090625** | **0.12724936** | **0.46659252** | **1** | **4802** | **tags=60%, list=36%, signal=94%** |
| **LIPOPROTEIN_METABOLIC_PROCESS** | **LIPOPROTEIN_METABOLIC_PROCESS** | | **30** | **-0.42722493** | **-1.3087521** | **0.12864077** | **0.46366104** | **1** | **718** | **tags=13%, list=5%, signal=14%** |
| **NEGATIVE_REGULATION_OF_SECRETION** | **NEGATIVE_REGULATION_OF_SECRETION** | | **9** | **-0.5750878** | **-1.306078** | **0.1422652** | **0.4673951** | **1** | **2935** | **tags=56%, list=22%, signal=71%** |
| **GLUCAN_METABOLIC_PROCESS** | **GLUCAN_METABOLIC_PROCESS** | | **9** | **-0.5607032** | **-1.304033** | **0.13986014** | **0.4697151** | **1** | **3139** | **tags=44%, list=24%, signal=58%** |
| **FATTY_ACID_BETA_OXIDATION** | **FATTY_ACID_BETA_OXIDATION** | | **10** | **-0.5397877** | **-1.3006557** | **0.13610315** | **0.47610417** | **1** | **2461** | **tags=30%, list=18%, signal=37%** |
| **MULTICELLULAR_ORGANISMAL_DEVELOPMENT** | **MULTICELLULAR_ORGANISMAL_DEVELOPMENT** | | **899** | **-0.31730536** | **-1.2974914** | **0** | **0.481929** | **1** | **3961** | **tags=37%, list=30%, signal=49%** |
| **G_PROTEIN_SIGNALING_ADENYLATE_CYCLASE_INHIBITING_PATHWAY** | **G_PROTEIN_SIGNALING_ADENYLATE_CYCLASE_INHIBITING_PATHWAY** | | **10** | **-0.53693086** | **-1.2959707** | **0.17191978** | **0.4826874** | **1** | **2585** | **tags=70%, list=19%, signal=87%** |
| **GENERATION_OF_NEURONS** | **GENERATION_OF_NEURONS** | | **66** | **-0.36373082** | **-1.2929485** | **0.09945355** | **0.4881528** | **1** | **2020** | **tags=26%, list=15%, signal=30%** |
| **BEHAVIOR** | **BEHAVIOR** |  | **132** | **-0.34377247** | **-1.292124** | **0.06542056** | **0.48677313** | **1** | **2061** | **tags=28%, list=15%, signal=33%** |
| **BRAIN_DEVELOPMENT** | **BRAIN_DEVELOPMENT** | | **35** | **-0.40410322** | **-1.2871579** | **0.117506** | **0.49737918** | **1** | **3146** | **tags=37%, list=24%, signal=48%** |
| **RESPONSE_TO_UV** | **RESPONSE_TO_UV** | | **22** | **-0.44748572** | **-1.2856266** | **0.15424837** | **0.49846357** | **1** | **4084** | **tags=55%, list=31%, signal=79%** |
| **CELL_PROJECTION_BIOGENESIS** | **CELL_PROJECTION_BIOGENESIS** | | **19** | **-0.46106154** | **-1.2846819** | **0.14800514** | **0.49749616** | **1** | **2991** | **tags=42%, list=22%, signal=54%** |
| **FEMALE_PREGNANCY** | **FEMALE_PREGNANCY** | | **41** | **-0.3951008** | **-1.2837393** | **0.12788019** | **0.49634987** | **1** | **2540** | **tags=34%, list=19%, signal=42%** |
| **REGULATION_OF_MUSCLE_CONTRACTION** | **REGULATION_OF_MUSCLE_CONTRACTION** | | **17** | **-0.4633347** | **-1.2829043** | **0.163827** | **0.49539647** | **1** | **2272** | **tags=35%, list=17%, signal=42%** |
| **NITROGEN_COMPOUND_CATABOLIC_PROCESS** | **NITROGEN_COMPOUND_CATABOLIC_PROCESS** | | **26** | **-0.42480597** | **-1.2828403** | **0.1402214** | **0.4919334** | **1** | **5009** | **tags=54%, list=38%, signal=86%** |
| **REGULATION_OF_PROTEIN_POLYMERIZATION** | **REGULATION_OF_PROTEIN_POLYMERIZATION** | | **10** | **-0.5432453** | **-1.2763368** | **0.17621145** | **0.50766253** | **1** | **781** | **tags=20%, list=6%, signal=21%** |
| **GLUCOSE_CATABOLIC_PROCESS** | **GLUCOSE_CATABOLIC_PROCESS** | | **11** | **-0.5124844** | **-1.2686706** | **0.18907563** | **0.52742773** | **1** | **5805** | **tags=73%, list=44%, signal=129%** |
| **ACTIVATION_OF_NF_KAPPAB_TRANSCRIPTION_FACTOR** | **ACTIVATION_OF_NF_KAPPAB_TRANSCRIPTION_FACTOR** | | **14** | **-0.4832907** | **-1.2615265** | **0.19086021** | **0.5458703** | **1** | **4084** | **tags=50%, list=31%, signal=72%** |
| **MONOCARBOXYLIC_ACID_METABOLIC_PROCESS** | **MONOCARBOXYLIC_ACID_METABOLIC_PROCESS** | | **74** | **-0.34843943** | **-1.2594523** | **0.12662338** | **0.548353** | **1** | **4083** | **tags=35%, list=31%, signal=50%** |
| **SENSORY_PERCEPTION_OF_CHEMICAL_STIMULUS** | **SENSORY_PERCEPTION_OF_CHEMICAL_STIMULUS** | | **12** | **-0.50342983** | **-1.2585992** | **0.20862308** | **0.5472361** | **1** | **1137** | **tags=42%, list=9%, signal=46%** |
| **SENSORY_PERCEPTION_OF_TASTE** | **SENSORY_PERCEPTION_OF_TASTE** | | **5** | **-0.65084887** | **-1.2576646** | **0.190625** | **0.54624164** | **1** | **3853** | **tags=80%, list=29%, signal=113%** |
| **SPHINGOLIPID_BIOSYNTHETIC_PROCESS** | **SPHINGOLIPID_BIOSYNTHETIC_PROCESS** | | **5** | **-0.6463767** | **-1.257632** | **0.18478261** | **0.54253006** | **1** | **1070** | **tags=40%, list=8%, signal=43%** |
| **PIGMENT_METABOLIC_PROCESS** | **PIGMENT_METABOLIC_PROCESS** | | **18** | **-0.4561562** | **-1.2564801** | **0.17254902** | **0.5425378** | **1** | **3551** | **tags=44%, list=27%, signal=61%** |
| **PRODUCTION_OF_MOLECULAR_MEDIATOR_OF_IMMUNE_RESPONSE** | **PRODUCTION_OF_MOLECULAR_MEDIATOR_OF_IMMUNE_RESPONSE** | | **11** | **-0.51052606** | **-1.2528967** | **0.18130311** | **0.5502264** | **1** | **1344** | **tags=27%, list=10%, signal=30%** |
| **DEVELOPMENTAL_MATURATION** | **DEVELOPMENTAL_MATURATION** | | **17** | **-0.46066868** | **-1.2488735** | **0.19587629** | **0.55905366** | **1** | **3323** | **tags=41%, list=25%, signal=55%** |
| **AMINO_ACID_TRANSPORT** | **AMINO_ACID_TRANSPORT** | | **26** | **-0.42023325** | **-1.248399** | **0.16029593** | **0.55686826** | **1** | **4444** | **tags=46%, list=33%, signal=69%** |
| **NEURON_DEVELOPMENT** | **NEURON_DEVELOPMENT** | | **50** | **-0.36785614** | **-1.2481045** | **0.145977** | **0.5541643** | **1** | **2020** | **tags=26%, list=15%, signal=31%** |
| **NEGATIVE_REGULATION_OF_RESPONSE_TO_STIMULUS** | **NEGATIVE_REGULATION_OF_RESPONSE_TO_STIMULUS** | | **9** | **-0.5306184** | **-1.247588** | **0.19087137** | **0.55221903** | **1** | **1767** | **tags=44%, list=13%, signal=51%** |
| **RESPONSE_TO_TOXIN** | **RESPONSE_TO_TOXIN** | | **9** | **-0.54701954** | **-1.2452879** | **0.19912152** | **0.5558184** | **1** | **3761** | **tags=44%, list=28%, signal=62%** |
| **G1_PHASE** | **G1_PHASE** |  | **13** | **-0.48580223** | **-1.2419106** | **0.19556172** | **0.56302273** | **1** | **2450** | **tags=38%, list=18%, signal=47%** |
| **CELLULAR_LIPID_METABOLIC_PROCESS** | **CELLULAR_LIPID_METABOLIC_PROCESS** | | **207** | **-0.31978378** | **-1.2417725** | **0.05342742** | **0.5597339** | **1** | **4165** | **tags=33%, list=31%, signal=47%** |
| **CALCIUM_INDEPENDENT_CELL_CELL_ADHESION** | **CALCIUM_INDEPENDENT_CELL_CELL_ADHESION** | | **16** | **-0.45582756** | **-1.2406416** | **0.20387097** | **0.5596296** | **1** | **3501** | **tags=50%, list=26%, signal=68%** |
| **PROTEIN_TETRAMERIZATION** | **PROTEIN_TETRAMERIZATION** | | **13** | **-0.48093477** | **-1.2362145** | **0.21143617** | **0.5700359** | **1** | **2206** | **tags=31%, list=17%, signal=37%** |
| **GLYCOLIPID_METABOLIC_PROCESS** | **GLYCOLIPID_METABOLIC_PROCESS** | | **13** | **-0.48424566** | **-1.2355798** | **0.21486486** | **0.56841034** | **1** | **3311** | **tags=54%, list=25%, signal=72%** |
| **DEFENSE_RESPONSE_TO_BACTERIUM** | **DEFENSE_RESPONSE_TO_BACTERIUM** | | **13** | **-0.47748977** | **-1.2300111** | **0.20327422** | **0.58257043** | **1** | **3011** | **tags=54%, list=23%, signal=70%** |
| **REGULATION_OF_MULTICELLULAR_ORGANISMAL_PROCESS** | **REGULATION_OF_MULTICELLULAR_ORGANISMAL_PROCESS** | | **127** | **-0.32491922** | **-1.2295227** | **0.12029289** | **0.58054316** | **1** | **3709** | **tags=34%, list=28%, signal=46%** |
| **TRIACYLGLYCEROL_METABOLIC_PROCESS** | **TRIACYLGLYCEROL_METABOLIC_PROCESS** | | **6** | **-0.5888741** | **-1.2260344** | **0.22904192** | **0.587795** | **1** | **4248** | **tags=67%, list=32%, signal=98%** |
| **SPHINGOLIPID_METABOLIC_PROCESS** | **SPHINGOLIPID_METABOLIC_PROCESS** | | **21** | **-0.4286238** | **-1.223296** | **0.22403003** | **0.5933725** | **1** | **2561** | **tags=33%, list=19%, signal=41%** |
| **PERIPHERAL_NERVOUS_SYSTEM_DEVELOPMENT** | **PERIPHERAL_NERVOUS_SYSTEM_DEVELOPMENT** | | **12** | **-0.48084685** | **-1.2201002** | **0.2260274** | **0.6004976** | **1** | **2061** | **tags=42%, list=15%, signal=49%** |
| **ANATOMICAL_STRUCTURE_MORPHOGENESIS** | **ANATOMICAL_STRUCTURE_MORPHOGENESIS** | | **324** | **-0.3067653** | **-1.2156534** | **0.04914744** | **0.6111658** | **1** | **3168** | **tags=30%, list=24%, signal=38%** |
| **NEURON_APOPTOSIS** | **NEURON_APOPTOSIS** | | **14** | **-0.4744841** | **-1.2123983** | **0.21944809** | **0.61849135** | **1** | **3650** | **tags=36%, list=27%, signal=49%** |
| **REGULATION_OF_ACTION_POTENTIAL** | **REGULATION_OF_ACTION_POTENTIAL** | | **16** | **-0.45322952** | **-1.2120202** | **0.22739018** | **0.61586374** | **1** | **2061** | **tags=25%, list=15%, signal=30%** |
| **PROTEIN_POLYUBIQUITINATION** | **PROTEIN_POLYUBIQUITINATION** | | **10** | **-0.50094366** | **-1.2118844** | **0.23520924** | **0.61255795** | **1** | **4708** | **tags=50%, list=35%, signal=77%** |
| **POSITIVE_REGULATION_OF_CELLULAR_PROTEIN_METABOLIC_PROCESS** | **POSITIVE_REGULATION_OF_CELLULAR_PROTEIN_METABOLIC_PROCESS** | | **59** | **-0.34874278** | **-1.2093589** | **0.1809101** | **0.61716634** | **1** | **1104** | **tags=17%, list=8%, signal=18%** |
| **POSITIVE_REGULATION_OF_PROTEIN_MODIFICATION_PROCESS** | **POSITIVE_REGULATION_OF_PROTEIN_MODIFICATION_PROCESS** | | **23** | **-0.4072488** | **-1.2037686** | **0.21182266** | **0.63201797** | **1** | **1104** | **tags=17%, list=8%, signal=19%** |
| **GLYCOSPHINGOLIPID_METABOLIC_PROCESS** | **GLYCOSPHINGOLIPID_METABOLIC_PROCESS** | | **11** | **-0.49130693** | **-1.2022263** | **0.25342464** | **0.63368005** | **1** | **2561** | **tags=45%, list=19%, signal=56%** |
| **ORGANIC_ACID_TRANSPORT** | **ORGANIC_ACID_TRANSPORT** | | **41** | **-0.36627215** | **-1.2007612** | **0.21222606** | **0.63474244** | **1** | **4444** | **tags=44%, list=33%, signal=66%** |
| **CENTROSOME_ORGANIZATION_AND_BIOGENESIS** | **CENTROSOME_ORGANIZATION_AND_BIOGENESIS** | | **11** | **-0.49033612** | **-1.1990879** | **0.25** | **0.63660574** | **1** | **1285** | **tags=27%, list=10%, signal=30%** |
| **POSITIVE_REGULATION_OF_CELL_MIGRATION** | **POSITIVE_REGULATION_OF_CELL_MIGRATION** | | **8** | **-0.53833133** | **-1.1980509** | **0.24743778** | **0.63602865** | **1** | **1626** | **tags=25%, list=12%, signal=28%** |
| **SMOOTH_MUSCLE_CONTRACTION_GO_0006939** | **SMOOTH_MUSCLE_CONTRACTION_GO_0006939** | | **11** | **-0.49878013** | **-1.1974705** | **0.24071527** | **0.6343185** | **1** | **3481** | **tags=45%, list=26%, signal=61%** |
| **CELL_MATURATION** | **CELL_MATURATION** | | **15** | **-0.45069566** | **-1.1930907** | **0.24830393** | **0.64539605** | **1** | **3245** | **tags=40%, list=24%, signal=53%** |
| **POSITIVE_REGULATION_OF_ANGIOGENESIS** | **POSITIVE_REGULATION_OF_ANGIOGENESIS** | | **9** | **-0.5135011** | **-1.1915717** | **0.2631579** | **0.6468859** | **1** | **1626** | **tags=33%, list=12%, signal=38%** |
| **RESPONSE_TO_BACTERIUM** | **RESPONSE_TO_BACTERIUM** | | **19** | **-0.42070076** | **-1.1909009** | **0.21815889** | **0.6455085** | **1** | **674** | **tags=26%, list=5%, signal=28%** |
| **NEGATIVE_REGULATION_OF_MULTICELLULAR_ORGANISMAL_PROCESS** | **NEGATIVE_REGULATION_OF_MULTICELLULAR_ORGANISMAL_PROCESS** | | **26** | **-0.4008219** | **-1.1890403** | **0.24187726** | **0.64838225** | **1** | **3689** | **tags=50%, list=28%, signal=69%** |
| **HETEROCYCLE_METABOLIC_PROCESS** | **HETEROCYCLE_METABOLIC_PROCESS** | | **25** | **-0.39663574** | **-1.1856015** | **0.24968633** | **0.65646756** | **1** | **3551** | **tags=36%, list=27%, signal=49%** |
| **CARBOXYLIC_ACID_TRANSPORT** | **CARBOXYLIC_ACID_TRANSPORT** | | **41** | **-0.36627215** | **-1.1846242** | **0.21809745** | **0.6560943** | **1** | **4444** | **tags=44%, list=33%, signal=66%** |
| **SECRETION_BY_CELL** | **SECRETION_BY_CELL** | | **92** | **-0.32311645** | **-1.1843455** | **0.20362474** | **0.65341103** | **1** | **3771** | **tags=35%, list=28%, signal=48%** |
| **CELL_MATRIX_ADHESION** | **CELL_MATRIX_ADHESION** | | **34** | **-0.3680712** | **-1.1840236** | **0.2386091** | **0.65093166** | **1** | **2029** | **tags=26%, list=15%, signal=31%** |
| **POSITIVE_REGULATION_OF_PROTEIN_METABOLIC_PROCESS** | **POSITIVE_REGULATION_OF_PROTEIN_METABOLIC_PROCESS** | | **61** | **-0.33710685** | **-1.1837164** | **0.19252548** | **0.64822924** | **1** | **1104** | **tags=16%, list=8%, signal=18%** |
| **MICROTUBULE_ORGANIZING_CENTER_ORGANIZATION_AND_BIOGENESIS** | **MICROTUBULE_ORGANIZING_CENTER_ORGANIZATION_AND_BIOGENESIS** | | **12** | **-0.4698053** | **-1.1836812** | **0.24353741** | **0.6447552** | **1** | **1285** | **tags=25%, list=10%, signal=28%** |
| **MYELOID_LEUKOCYTE_DIFFERENTIATION** | **MYELOID_LEUKOCYTE_DIFFERENTIATION** | | **14** | **-0.45202023** | **-1.1754049** | **0.26196808** | **0.669587** | **1** | **3505** | **tags=43%, list=26%, signal=58%** |
| **ORGAN_DEVELOPMENT** | **ORGAN_DEVELOPMENT** | | **493** | **-0.2898785** | **-1.1711105** | **0.051** | **0.68084294** | **1** | **3759** | **tags=34%, list=28%, signal=46%** |
| **LIPID_HOMEOSTASIS** | **LIPID_HOMEOSTASIS** | | **15** | **-0.43543428** | **-1.1707419** | **0.28289473** | **0.67839754** | **1** | **278** | **tags=13%, list=2%, signal=14%** |
| **CHEMICAL_HOMEOSTASIS** | **CHEMICAL_HOMEOSTASIS** | | **133** | **-0.30824146** | **-1.1659111** | **0.20889348** | **0.6917159** | **1** | **2062** | **tags=22%, list=15%, signal=26%** |
| **SKELETAL_DEVELOPMENT** | **SKELETAL_DEVELOPMENT** | | **91** | **-0.3158714** | **-1.1650505** | **0.21360256** | **0.69086915** | **1** | **3498** | **tags=30%, list=26%, signal=40%** |
| **PROTEIN_AMINO_ACID_AUTOPHOSPHORYLATION** | **PROTEIN_AMINO_ACID_AUTOPHOSPHORYLATION** | | **24** | **-0.3899212** | **-1.1649809** | **0.274359** | **0.68742204** | **1** | **4529** | **tags=46%, list=34%, signal=69%** |
| **PROTEIN_POLYMERIZATION** | **PROTEIN_POLYMERIZATION** | | **16** | **-0.4306739** | **-1.1639163** | **0.28894806** | **0.68754745** | **1** | **1078** | **tags=19%, list=8%, signal=20%** |
| **MEMBRANE_LIPID_METABOLIC_PROCESS** | **MEMBRANE_LIPID_METABOLIC_PROCESS** | | **80** | **-0.32173687** | **-1.1629952** | **0.23109244** | **0.6870187** | **1** | **3842** | **tags=31%, list=29%, signal=44%** |
| **REGULATION_OF_TRANSPORT** | **REGULATION_OF_TRANSPORT** | | **55** | **-0.33840165** | **-1.1617421** | **0.255079** | **0.68767536** | **1** | **1770** | **tags=18%, list=13%, signal=21%** |
| **CELLULAR_LIPID_CATABOLIC_PROCESS** | **CELLULAR_LIPID_CATABOLIC_PROCESS** | | **28** | **-0.38348836** | **-1.1602943** | **0.24878049** | **0.6891396** | **1** | **3506** | **tags=39%, list=26%, signal=53%** |
| **POSITIVE_REGULATION_OF_TRANSLATION** | **POSITIVE_REGULATION_OF_TRANSLATION** | | **28** | **-0.3736972** | **-1.1547997** | **0.2693727** | **0.70446575** | **1** | **3391** | **tags=36%, list=25%, signal=48%** |
| **PROTEIN_AUTOPROCESSING** | **PROTEIN_AUTOPROCESSING** | | **24** | **-0.3899212** | **-1.1543064** | **0.26666668** | **0.7027013** | **1** | **4529** | **tags=46%, list=34%, signal=69%** |
| **REGULATION_OF_NEURON_APOPTOSIS** | **REGULATION_OF_NEURON_APOPTOSIS** | | **10** | **-0.4806456** | **-1.1538643** | **0.3042292** | **0.7006037** | **1** | **3650** | **tags=40%, list=27%, signal=55%** |
| **SISTER_CHROMATID_SEGREGATION** | **SISTER_CHROMATID_SEGREGATION** | | **13** | **-0.44481024** | **-1.1517698** | **0.27334234** | **0.70455647** | **1** | **5572** | **tags=69%, list=42%, signal=119%** |
| **POSITIVE_REGULATION_OF_PROTEIN_AMINO_ACID_PHOSPHORYLATION** | **POSITIVE_REGULATION_OF_PROTEIN_AMINO_ACID_PHOSPHORYLATION** | | **14** | **-0.43844715** | **-1.1512597** | **0.29506007** | **0.7026462** | **1** | **1104** | **tags=21%, list=8%, signal=23%** |
| **CATION_HOMEOSTASIS** | **CATION_HOMEOSTASIS** | | **93** | **-0.31187382** | **-1.1502073** | **0.23411018** | **0.7027172** | **1** | **2062** | **tags=24%, list=15%, signal=28%** |
| **SYNAPSE_ORGANIZATION_AND_BIOGENESIS** | **SYNAPSE_ORGANIZATION_AND_BIOGENESIS** | | **14** | **-0.44135857** | **-1.1445446** | **0.28668478** | **0.7186704** | **1** | **3926** | **tags=43%, list=29%, signal=61%** |
| **POSITIVE_REGULATION_OF_CYTOKINE_BIOSYNTHETIC_PROCESS** | **POSITIVE_REGULATION_OF_CYTOKINE_BIOSYNTHETIC_PROCESS** | | **21** | **-0.3970721** | **-1.1426847** | **0.28994846** | **0.7216148** | **1** | **460** | **tags=19%, list=3%, signal=20%** |
| **AMINE_BIOSYNTHETIC_PROCESS** | **AMINE_BIOSYNTHETIC_PROCESS** | | **15** | **-0.4352044** | **-1.1414276** | **0.29514825** | **0.7223201** | **1** | **4976** | **tags=47%, list=37%, signal=74%** |
| **POSITIVE_REGULATION_OF_TRANSPORT** | **POSITIVE_REGULATION_OF_TRANSPORT** | | **18** | **-0.41138482** | **-1.1391586** | **0.28702492** | **0.72654754** | **1** | **3689** | **tags=33%, list=28%, signal=46%** |
| **LIPID_TRANSPORT** | **LIPID_TRANSPORT** | | **27** | **-0.37389392** | **-1.137745** | **0.29588014** | **0.7279499** | **1** | **278** | **tags=11%, list=2%, signal=11%** |
| **INTERLEUKIN_8_PRODUCTION** | **INTERLEUKIN_8_PRODUCTION** | | **8** | **-0.5037998** | **-1.1316466** | **0.31240875** | **0.74669945** | **1** | **2522** | **tags=38%, list=19%, signal=46%** |
| **BIOGENIC_AMINE_METABOLIC_PROCESS** | **BIOGENIC_AMINE_METABOLIC_PROCESS** | | **16** | **-0.42137364** | **-1.130441** | **0.32345015** | **0.7477108** | **1** | **1509** | **tags=25%, list=11%, signal=28%** |
| **CELL_SUBSTRATE_ADHESION** | **CELL_SUBSTRATE_ADHESION** | | **35** | **-0.35539034** | **-1.1298858** | **0.30023363** | **0.7460303** | **1** | **2029** | **tags=26%, list=15%, signal=30%** |
| **GLIOGENESIS** | **GLIOGENESIS** | | **11** | **-0.46429163** | **-1.1282977** | **0.3215797** | **0.74826586** | **1** | **4208** | **tags=55%, list=32%, signal=80%** |
| **HISTONE_MODIFICATION** | **HISTONE_MODIFICATION** | | **18** | **-0.41425768** | **-1.1218948** | **0.3167785** | **0.76710933** | **1** | **5563** | **tags=67%, list=42%, signal=114%** |
| **ION_HOMEOSTASIS** | **ION_HOMEOSTASIS** | | **110** | **-0.29995495** | **-1.1161054** | **0.2679875** | **0.78408897** | **1** | **2062** | **tags=22%, list=15%, signal=26%** |
| **CELLULAR_HOMEOSTASIS** | **CELLULAR_HOMEOSTASIS** | | **119** | **-0.29583025** | **-1.1147101** | **0.25** | **0.7855137** | **1** | **2522** | **tags=25%, list=19%, signal=31%** |
| **PHOSPHOLIPID_METABOLIC_PROCESS** | **PHOSPHOLIPID_METABOLIC_PROCESS** | | **59** | **-0.3216962** | **-1.1141918** | **0.3063263** | **0.783651** | **1** | **3842** | **tags=31%, list=29%, signal=43%** |
| **LIPID_METABOLIC_PROCESS** | **LIPID_METABOLIC_PROCESS** | | **266** | **-0.28145248** | **-1.1133621** | **0.2118474** | **0.7830532** | **1** | **3311** | **tags=26%, list=25%, signal=34%** |
| **COVALENT_CHROMATIN_MODIFICATION** | **COVALENT_CHROMATIN_MODIFICATION** | | **19** | **-0.3948433** | **-1.1131309** | **0.31140938** | **0.78015983** | **1** | **5563** | **tags=63%, list=42%, signal=108%** |
| **SKELETAL_MUSCLE_DEVELOPMENT** | **SKELETAL_MUSCLE_DEVELOPMENT** | | **30** | **-0.36186445** | **-1.1086493** | **0.33172497** | **0.7922798** | **1** | **3245** | **tags=37%, list=24%, signal=48%** |
| **ESTABLISHMENT_OF_LOCALIZATION** | **ESTABLISHMENT_OF_LOCALIZATION** | | **752** | **-0.27182066** | **-1.1080652** | **0.117** | **0.7905691** | **1** | **3843** | **tags=31%, list=29%, signal=41%** |
| **PROTEIN_SECRETION** | **PROTEIN_SECRETION** | | **22** | **-0.38257864** | **-1.107927** | **0.3186951** | **0.78742397** | **1** | **3391** | **tags=41%, list=25%, signal=55%** |
| **ENDOTHELIAL_CELL_MIGRATION** | **ENDOTHELIAL_CELL_MIGRATION** | | **11** | **-0.45506445** | **-1.1060574** | **0.36134455** | **0.7905254** | **1** | **2522** | **tags=27%, list=19%, signal=34%** |
| **FATTY_ACID_METABOLIC_PROCESS** | **FATTY_ACID_METABOLIC_PROCESS** | | **53** | **-0.3212799** | **-1.1048834** | **0.3130734** | **0.79099977** | **1** | **4083** | **tags=32%, list=31%, signal=46%** |
| **TUBE_MORPHOGENESIS** | **TUBE_MORPHOGENESIS** | | **13** | **-0.43280277** | **-1.1046793** | **0.35185185** | **0.78815675** | **1** | **2768** | **tags=38%, list=21%, signal=49%** |
| **TRANSFORMING_GROWTH_FACTOR_BETA_RECEPTOR_SIGNALING_PATHWAY** | **TRANSFORMING_GROWTH_FACTOR_BETA_RECEPTOR_SIGNALING_PATHWAY** | | **34** | **-0.35093242** | **-1.1046774** | **0.32886723** | **0.7845578** | **1** | **2754** | **tags=29%, list=21%, signal=37%** |
| **SECRETORY_PATHWAY** | **SECRETORY_PATHWAY** | | **70** | **-0.31032556** | **-1.09688** | **0.32928178** | **0.8088644** | **1** | **3771** | **tags=33%, list=28%, signal=46%** |
| **REGULATION_OF_RESPONSE_TO_EXTERNAL_STIMULUS** | **REGULATION_OF_RESPONSE_TO_EXTERNAL_STIMULUS** | | **12** | **-0.43371236** | **-1.096085** | **0.36615813** | **0.8079499** | **1** | **1859** | **tags=33%, list=14%, signal=39%** |
| **UBIQUITIN_CYCLE** | **UBIQUITIN_CYCLE** | | **42** | **-0.33415124** | **-1.0929877** | **0.33025405** | **0.8153306** | **1** | **1725** | **tags=19%, list=13%, signal=22%** |
| **TRANSPORT** | **TRANSPORT** |  | **691** | **-0.26804155** | **-1.092655** | **0.168** | **0.8130305** | **1** | **3698** | **tags=29%, list=28%, signal=39%** |
| **CELLULAR_MONOVALENT_INORGANIC_CATION_HOMEOSTASIS** | **CELLULAR_MONOVALENT_INORGANIC_CATION_HOMEOSTASIS** | | **10** | **-0.4515793** | **-1.0867479** | **0.36834735** | **0.83073276** | **1** | **4215** | **tags=40%, list=32%, signal=58%** |
| **REGULATION_OF_BODY_FLUID_LEVELS** | **REGULATION_OF_BODY_FLUID_LEVELS** | | **54** | **-0.3186071** | **-1.0843639** | **0.34619752** | **0.83575505** | **1** | **3837** | **tags=41%, list=29%, signal=57%** |
| **CYTOKINE_BIOSYNTHETIC_PROCESS** | **CYTOKINE_BIOSYNTHETIC_PROCESS** | | **33** | **-0.34264058** | **-1.0824659** | **0.3823178** | **0.8390739** | **1** | **2028** | **tags=24%, list=15%, signal=29%** |
| **CELL_MIGRATION** | **CELL_MIGRATION** | | **82** | **-0.29741535** | **-1.0813344** | **0.3275862** | **0.83944285** | **1** | **2020** | **tags=21%, list=15%, signal=24%** |
| **HOMEOSTATIC_PROCESS** | **HOMEOSTATIC_PROCESS** | | **175** | **-0.27934164** | **-1.0795603** | **0.31135902** | **0.8419164** | **1** | **2522** | **tags=23%, list=19%, signal=28%** |
| **CELLULAR_POLYSACCHARIDE_METABOLIC_PROCESS** | **CELLULAR_POLYSACCHARIDE_METABOLIC_PROCESS** | | **11** | **-0.44290572** | **-1.0779046** | **0.37254903** | **0.84392184** | **1** | **1647** | **tags=27%, list=12%, signal=31%** |
| **VITAMIN_TRANSPORT** | **VITAMIN_TRANSPORT** | | **11** | **-0.44208473** | **-1.0765882** | **0.36925286** | **0.8452313** | **1** | **3513** | **tags=45%, list=26%, signal=62%** |
| **NEGATIVE_REGULATION_OF_CELL_DIFFERENTIATION** | **NEGATIVE_REGULATION_OF_CELL_DIFFERENTIATION** | | **23** | **-0.362034** | **-1.0746111** | **0.38385093** | **0.84884423** | **1** | **1413** | **tags=26%, list=11%, signal=29%** |
| **CYTOKINE_METABOLIC_PROCESS** | **CYTOKINE_METABOLIC_PROCESS** | | **34** | **-0.33914077** | **-1.073066** | **0.36754176** | **0.85066897** | **1** | **2028** | **tags=24%, list=15%, signal=28%** |
| **CIRCADIAN_RHYTHM** | **CIRCADIAN_RHYTHM** | | **13** | **-0.4141014** | **-1.0726147** | **0.38472223** | **0.8485396** | **1** | **2003** | **tags=31%, list=15%, signal=36%** |
| **REGULATION_OF_PROTEIN_SECRETION** | **REGULATION_OF_PROTEIN_SECRETION** | | **13** | **-0.41314018** | **-1.0724188** | **0.40106952** | **0.8456024** | **1** | **3391** | **tags=54%, list=25%, signal=72%** |
| **REGULATION_OF_BIOLOGICAL_QUALITY** | **REGULATION_OF_BIOLOGICAL_QUALITY** | | **351** | **-0.2672616** | **-1.0678794** | **0.31462926** | **0.8582142** | **1** | **2786** | **tags=24%, list=21%, signal=29%** |
| **REGULATION_OF_RESPONSE_TO_STIMULUS** | **REGULATION_OF_RESPONSE_TO_STIMULUS** | | **47** | **-0.316396** | **-1.067532** | **0.38856477** | **0.85577303** | **1** | **2028** | **tags=23%, list=15%, signal=28%** |
| **MYELOID_CELL_DIFFERENTIATION** | **MYELOID_CELL_DIFFERENTIATION** | | **32** | **-0.3438606** | **-1.0668268** | **0.38600725** | **0.8547032** | **1** | **2522** | **tags=31%, list=19%, signal=38%** |
| **DNA_DAMAGE_RESPONSESIGNAL_TRANSDUCTION_RESULTING_IN_INDUCTION_OF_APOPTOSIS** | **DNA_DAMAGE_RESPONSESIGNAL_TRANSDUCTION_RESULTING_IN_INDUCTION_OF_APOPTOSIS** | | **13** | **-0.42017606** | **-1.0660472** | **0.37702703** | **0.85381716** | **1** | **1574** | **tags=23%, list=12%, signal=26%** |
| **NEGATIVE_REGULATION_OF_HYDROLASE_ACTIVITY** | **NEGATIVE_REGULATION_OF_HYDROLASE_ACTIVITY** | | **14** | **-0.40729338** | **-1.0649059** | **0.39946738** | **0.8542429** | **1** | **796** | **tags=14%, list=6%, signal=15%** |
| **MEIOTIC_CELL_CYCLE** | **MEIOTIC_CELL_CYCLE** | | **28** | **-0.35162255** | **-1.064424** | **0.37468672** | **0.8525124** | **1** | **4305** | **tags=46%, list=32%, signal=68%** |
| **EXTRACELLULAR_STRUCTURE_ORGANIZATION_AND_BIOGENESIS** | **EXTRACELLULAR_STRUCTURE_ORGANIZATION_AND_BIOGENESIS** | | **22** | **-0.36718857** | **-1.0638092** | **0.39688715** | **0.8511906** | **1** | **547** | **tags=14%, list=4%, signal=14%** |
| **REGULATION_OF_SYNAPSE_STRUCTURE_AND_ACTIVITY** | **REGULATION_OF_SYNAPSE_STRUCTURE_AND_ACTIVITY** | | **7** | **-0.4905558** | **-1.0568537** | **0.42998585** | **0.8725489** | **1** | **1276** | **tags=29%, list=10%, signal=32%** |
| **S_PHASE_OF_MITOTIC_CELL_CYCLE** | **S_PHASE_OF_MITOTIC_CELL_CYCLE** | | **8** | **-0.46501648** | **-1.0565419** | **0.41248098** | **0.87001956** | **1** | **894** | **tags=25%, list=7%, signal=27%** |
| **JNK_CASCADE** | **JNK_CASCADE** | | **44** | **-0.31694138** | **-1.0563715** | **0.38505095** | **0.8670629** | **1** | **3114** | **tags=30%, list=23%, signal=38%** |
| **REGULATION_OF_SECRETION** | **REGULATION_OF_SECRETION** | | **29** | **-0.34549302** | **-1.0553697** | **0.39512196** | **0.8671061** | **1** | **3391** | **tags=38%, list=25%, signal=51%** |
| **CYTOKINE_PRODUCTION** | **CYTOKINE_PRODUCTION** | | **55** | **-0.31275028** | **-1.0549039** | **0.39732143** | **0.8651932** | **1** | **2551** | **tags=25%, list=19%, signal=31%** |
| **CELLULAR_CATION_HOMEOSTASIS** | **CELLULAR_CATION_HOMEOSTASIS** | | **90** | **-0.28973114** | **-1.0546205** | **0.39766702** | **0.86266077** | **1** | **2062** | **tags=22%, list=15%, signal=26%** |
| **TRANSMEMBRANE_RECEPTOR_PROTEIN_SERINE_THREONINE_KINASE_SIGNALING_PATHWAY** | **TRANSMEMBRANE_RECEPTOR_PROTEIN_SERINE_THREONINE_KINASE_SIGNALING_PATHWAY** | | **42** | **-0.32479537** | **-1.053136** | **0.3888889** | **0.864204** | **1** | **2754** | **tags=26%, list=21%, signal=33%** |
| **REGULATION_OF_CELLULAR_PH** | **REGULATION_OF_CELLULAR_PH** | | **9** | **-0.45243797** | **-1.0498133** | **0.41466856** | **0.8724033** | **1** | **4215** | **tags=44%, list=32%, signal=65%** |
| **ENZYME_LINKED_RECEPTOR_PROTEIN_SIGNALING_PATHWAY** | **ENZYME_LINKED_RECEPTOR_PROTEIN_SIGNALING_PATHWAY** | | **127** | **-0.27655026** | **-1.0482216** | **0.39031926** | **0.8741887** | **1** | **3515** | **tags=29%, list=26%, signal=39%** |
| **CELLULAR_CARBOHYDRATE_CATABOLIC_PROCESS** | **CELLULAR_CARBOHYDRATE_CATABOLIC_PROCESS** | | **20** | **-0.36892536** | **-1.0472006** | **0.41878173** | **0.87449425** | **1** | **2797** | **tags=30%, list=21%, signal=38%** |
| **CARBOHYDRATE_METABOLIC_PROCESS** | **CARBOHYDRATE_METABOLIC_PROCESS** | | **150** | **-0.27504587** | **-1.0468163** | **0.3836608** | **0.87248653** | **1** | **3426** | **tags=27%, list=26%, signal=35%** |
| **CARBOHYDRATE_CATABOLIC_PROCESS** | **CARBOHYDRATE_CATABOLIC_PROCESS** | | **20** | **-0.3689253** | **-1.0454007** | **0.39871794** | **0.87396944** | **1** | **2797** | **tags=30%, list=21%, signal=38%** |
| **REGULATION_OF_CYTOKINE_BIOSYNTHETIC_PROCESS** | **REGULATION_OF_CYTOKINE_BIOSYNTHETIC_PROCESS** | | **31** | **-0.33202112** | **-1.0429521** | **0.42409638** | **0.87893206** | **1** | **3391** | **tags=32%, list=25%, signal=43%** |
| **COFACTOR_TRANSPORT** | **COFACTOR_TRANSPORT** | | **9** | **-0.4521949** | **-1.0429287** | **0.43194443** | **0.8755743** | **1** | **2779** | **tags=33%, list=21%, signal=42%** |
| **WOUND_HEALING** | **WOUND_HEALING** | | **48** | **-0.30825868** | **-1.0417538** | **0.40753424** | **0.87617993** | **1** | **3709** | **tags=38%, list=28%, signal=52%** |
| **INTERLEUKIN_8_BIOSYNTHETIC_PROCESS** | **INTERLEUKIN_8_BIOSYNTHETIC_PROCESS** | | **7** | **-0.47702822** | **-1.0417451** | **0.4257576** | **0.8728056** | **1** | **2028** | **tags=29%, list=15%, signal=34%** |
| **ACUTE_INFLAMMATORY_RESPONSE** | **ACUTE_INFLAMMATORY_RESPONSE** | | **9** | **-0.44661084** | **-1.0382107** | **0.43453237** | **0.8811564** | **1** | **3689** | **tags=44%, list=28%, signal=61%** |
| **LIPOPROTEIN_BIOSYNTHETIC_PROCESS** | **LIPOPROTEIN_BIOSYNTHETIC_PROCESS** | | **23** | **-0.35826063** | **-1.0380567** | **0.42929935** | **0.8782933** | **1** | **3946** | **tags=26%, list=30%, signal=37%** |
| **CARBOHYDRATE_TRANSPORT** | **CARBOHYDRATE_TRANSPORT** | | **17** | **-0.3726356** | **-1.0357004** | **0.41848522** | **0.88281554** | **1** | **2894** | **tags=35%, list=22%, signal=45%** |
| **SECONDARY_METABOLIC_PROCESS** | **SECONDARY_METABOLIC_PROCESS** | | **23** | **-0.35400918** | **-1.0355233** | **0.44226044** | **0.88007796** | **1** | **3551** | **tags=35%, list=27%, signal=47%** |
| **MEIOTIC_RECOMBINATION** | **MEIOTIC_RECOMBINATION** | | **14** | **-0.39580637** | **-1.0334717** | **0.44192258** | **0.8839629** | **1** | **4290** | **tags=50%, list=32%, signal=74%** |
| **BIOPOLYMER_BIOSYNTHETIC_PROCESS** | **BIOPOLYMER_BIOSYNTHETIC_PROCESS** | | **7** | **-0.47851804** | **-1.0311301** | **0.45** | **0.8884116** | **1** | **3139** | **tags=43%, list=24%, signal=56%** |
| **AMINO_ACID_AND_DERIVATIVE_METABOLIC_PROCESS** | **AMINO_ACID_AND_DERIVATIVE_METABOLIC_PROCESS** | | **95** | **-0.27597067** | **-1.0274118** | **0.4459893** | **0.89747375** | **1** | **5009** | **tags=43%, list=38%, signal=69%** |
| **CHROMOSOME_ORGANIZATION_AND_BIOGENESIS** | **CHROMOSOME_ORGANIZATION_AND_BIOGENESIS** | | **97** | **-0.28023818** | **-1.0255878** | **0.44257113** | **0.9004141** | **1** | **4161** | **tags=32%, list=31%, signal=46%** |
| **CALCIUM_MEDIATED_SIGNALING** | **CALCIUM_MEDIATED_SIGNALING** | | **13** | **-0.39733064** | **-1.0230663** | **0.45380434** | **0.9056764** | **1** | **1826** | **tags=15%, list=14%, signal=18%** |
| **AXON_GUIDANCE** | **AXON_GUIDANCE** | | **19** | **-0.3629711** | **-1.0216918** | **0.44528303** | **0.90695137** | **1** | **2020** | **tags=26%, list=15%, signal=31%** |
| **MITOTIC_SISTER_CHROMATID_SEGREGATION** | **MITOTIC_SISTER_CHROMATID_SEGREGATION** | | **12** | **-0.40348294** | **-1.0195233** | **0.4496732** | **0.9110524** | **1** | **5572** | **tags=67%, list=42%, signal=115%** |
| **REGULATION_OF_PROTEIN_STABILITY** | **REGULATION_OF_PROTEIN_STABILITY** | | **14** | **-0.38931528** | **-1.0192715** | **0.46649146** | **0.9084299** | **1** | **15** | **tags=7%, list=0%, signal=7%** |
| **RESPONSE_TO_ORGANIC_SUBSTANCE** | **RESPONSE_TO_ORGANIC_SUBSTANCE** | | **27** | **-0.33777115** | **-1.0190359** | **0.4462299** | **0.9058085** | **1** | **4432** | **tags=37%, list=33%, signal=55%** |
| **CYTOKINE_SECRETION** | **CYTOKINE_SECRETION** | | **10** | **-0.4303201** | **-1.0173675** | **0.44729346** | **0.90787274** | **1** | **2249** | **tags=40%, list=17%, signal=48%** |
| **MICROTUBULE_POLYMERIZATION_OR_DEPOLYMERIZATION** | **MICROTUBULE_POLYMERIZATION_OR_DEPOLYMERIZATION** | | **11** | **-0.41937143** | **-1.0166372** | **0.46331522** | **0.90698034** | **1** | **1078** | **tags=18%, list=8%, signal=20%** |
| **LEARNING_AND_OR_MEMORY** | **LEARNING_AND_OR_MEMORY** | | **14** | **-0.39324838** | **-1.0162463** | **0.44657534** | **0.90499365** | **1** | **2004** | **tags=29%, list=15%, signal=34%** |
| **STRESS_ACTIVATED_PROTEIN_KINASE_SIGNALING_PATHWAY** | **STRESS_ACTIVATED_PROTEIN_KINASE_SIGNALING_PATHWAY** | | **45** | **-0.30804777** | **-1.0156261** | **0.4569378** | **0.90377766** | **1** | **3114** | **tags=29%, list=23%, signal=38%** |
| **LIPID_BIOSYNTHETIC_PROCESS** | **LIPID_BIOSYNTHETIC_PROCESS** | | **78** | **-0.28235078** | **-1.0142452** | **0.47121534** | **0.9050987** | **1** | **2303** | **tags=17%, list=17%, signal=20%** |
| **STRIATED_MUSCLE_DEVELOPMENT** | **STRIATED_MUSCLE_DEVELOPMENT** | | **37** | **-0.31659988** | **-1.0137289** | **0.4583836** | **0.9036047** | **1** | **4243** | **tags=38%, list=32%, signal=55%** |
| **DNA_RECOMBINATION** | **DNA_RECOMBINATION** | | **41** | **-0.31218097** | **-1.0135467** | **0.45369285** | **0.90096927** | **1** | **4492** | **tags=41%, list=34%, signal=62%** |
| **REGULATION_OF_CYTOKINE_PRODUCTION** | **REGULATION_OF_CYTOKINE_PRODUCTION** | | **20** | **-0.3528505** | **-1.0116832** | **0.45019406** | **0.9039891** | **1** | **3883** | **tags=40%, list=29%, signal=56%** |
| **IMMUNE_EFFECTOR_PROCESS** | **IMMUNE_EFFECTOR_PROCESS** | | **31** | **-0.32505587** | **-1.0104749** | **0.45005876** | **0.9045611** | **1** | **2028** | **tags=19%, list=15%, signal=23%** |
| **REGULATION_OF_CYTOKINE_SECRETION** | **REGULATION_OF_CYTOKINE_SECRETION** | | **9** | **-0.43250453** | **-1.0064455** | **0.48079658** | **0.9144131** | **1** | **2249** | **tags=44%, list=17%, signal=53%** |
| **PROTEIN_AMINO_ACID_O_LINKED_GLYCOSYLATION** | **PROTEIN_AMINO_ACID_O_LINKED_GLYCOSYLATION** | | **18** | **-0.36329147** | **-1.0060033** | **0.47820345** | **0.91274846** | **1** | **1711** | **tags=17%, list=13%, signal=19%** |
| **REGULATION_OF_BINDING** | **REGULATION_OF_BINDING** | | **42** | **-0.30478427** | **-1.0014466** | **0.48349056** | **0.9241865** | **1** | **1344** | **tags=17%, list=10%, signal=18%** |
| **REGULATION_OF_GENE_EXPRESSION_EPIGENETIC** | **REGULATION_OF_GENE_EXPRESSION_EPIGENETIC** | | **27** | **-0.32981515** | **-1.0005656** | **0.4864532** | **0.92389196** | **1** | **3391** | **tags=26%, list=25%, signal=35%** |
| **CELLULAR_RESPONSE_TO_NUTRIENT_LEVELS** | **CELLULAR_RESPONSE_TO_NUTRIENT_LEVELS** | | **7** | **-0.45517802** | **-0.9987027** | **0.4801902** | **0.9263876** | **1** | **5049** | **tags=57%, list=38%, signal=92%** |
| **CARBOXYLIC_ACID_METABOLIC_PROCESS** | **CARBOXYLIC_ACID_METABOLIC_PROCESS** | | **156** | **-0.25952372** | **-0.99793875** | **0.49638057** | **0.9257145** | **1** | **4592** | **tags=35%, list=34%, signal=52%** |
| **NEGATIVE_REGULATION_OF_MYELOID_CELL_DIFFERENTIATION** | **NEGATIVE_REGULATION_OF_MYELOID_CELL_DIFFERENTIATION** | | **10** | **-0.41959503** | **-0.9951621** | **0.4883402** | **0.9314651** | **1** | **1024** | **tags=30%, list=8%, signal=32%** |
| **DNA_PACKAGING** | **DNA_PACKAGING** | | **27** | **-0.32900724** | **-0.99432284** | **0.48877805** | **0.9309373** | **1** | **2839** | **tags=22%, list=21%, signal=28%** |
| **PROTEIN_HOMOOLIGOMERIZATION** | **PROTEIN_HOMOOLIGOMERIZATION** | | **17** | **-0.36127356** | **-0.9927206** | **0.4828496** | **0.93276244** | **1** | **4896** | **tags=47%, list=37%, signal=74%** |
| **PROTEIN_COMPLEX_ASSEMBLY** | **PROTEIN_COMPLEX_ASSEMBLY** | | **149** | **-0.26070407** | **-0.9919313** | **0.52012384** | **0.9320513** | **1** | **4902** | **tags=41%, list=37%, signal=64%** |
| **HETEROPHILIC_CELL_ADHESION** | **HETEROPHILIC_CELL_ADHESION** | | **6** | **-0.47746554** | **-0.99171346** | **0.49848485** | **0.92954475** | **1** | **4661** | **tags=67%, list=35%, signal=103%** |
| **NEGATIVE_REGULATION_OF_BIOSYNTHETIC_PROCESS** | **NEGATIVE_REGULATION_OF_BIOSYNTHETIC_PROCESS** | | **24** | **-0.3318445** | **-0.99113935** | **0.4975186** | **0.9282365** | **1** | **3139** | **tags=33%, list=24%, signal=44%** |
| **NEGATIVE_REGULATION_OF_CELLULAR_BIOSYNTHETIC_PROCESS** | **NEGATIVE_REGULATION_OF_CELLULAR_BIOSYNTHETIC_PROCESS** | | **23** | **-0.33655533** | **-0.9901277** | **0.49425286** | **0.928219** | **1** | **3139** | **tags=35%, list=24%, signal=45%** |
| **DEVELOPMENTAL_GROWTH** | **DEVELOPMENTAL_GROWTH** | | **7** | **-0.45934737** | **-0.98826265** | **0.49475262** | **0.93083495** | **1** | **83** | **tags=14%, list=1%, signal=14%** |
| **CHROMATIN_REMODELING** | **CHROMATIN_REMODELING** | | **19** | **-0.34996668** | **-0.98750186** | **0.505689** | **0.9299903** | **1** | **2920** | **tags=26%, list=22%, signal=34%** |
| **POSITIVE_REGULATION_OF_TRANSCRIPTION_FACTOR_ACTIVITY** | **POSITIVE_REGULATION_OF_TRANSCRIPTION_FACTOR_ACTIVITY** | | **16** | **-0.36332268** | **-0.9869796** | **0.4848101** | **0.92851746** | **1** | **4084** | **tags=44%, list=31%, signal=63%** |
| **POSITIVE_REGULATION_OF_PEPTIDYL_TYROSINE_PHOSPHORYLATION** | **POSITIVE_REGULATION_OF_PEPTIDYL_TYROSINE_PHOSPHORYLATION** | | **9** | **-0.42945474** | **-0.9848553** | **0.4923077** | **0.93185633** | **1** | **1067** | **tags=22%, list=8%, signal=24%** |
| **CHROMATIN_ASSEMBLY** | **CHROMATIN_ASSEMBLY** | | **16** | **-0.3612893** | **-0.9841273** | **0.4814815** | **0.93089074** | **1** | **1950** | **tags=19%, list=15%, signal=22%** |
| **POLYSACCHARIDE_METABOLIC_PROCESS** | **POLYSACCHARIDE_METABOLIC_PROCESS** | | **13** | **-0.38073936** | **-0.9777177** | **0.48962656** | **0.9474676** | **1** | **1647** | **tags=23%, list=12%, signal=26%** |
| **ORGANIC_ACID_METABOLIC_PROCESS** | **ORGANIC_ACID_METABOLIC_PROCESS** | | **158** | **-0.25333172** | **-0.975438** | **0.5474227** | **0.9514133** | **1** | **4592** | **tags=34%, list=34%, signal=52%** |
| **HOMEOSTASIS_OF_NUMBER_OF_CELLS** | **HOMEOSTASIS_OF_NUMBER_OF_CELLS** | | **19** | **-0.34820744** | **-0.974552** | **0.5063613** | **0.950995** | **1** | **2309** | **tags=26%, list=17%, signal=32%** |
| **POSITIVE_REGULATION_OF_METABOLIC_PROCESS** | **POSITIVE_REGULATION_OF_METABOLIC_PROCESS** | | **182** | **-0.2509968** | **-0.97396594** | **0.550152** | **0.9496262** | **1** | **1731** | **tags=15%, list=13%, signal=17%** |
| **POSITIVE_REGULATION_OF_PHOSPHORYLATION** | **POSITIVE_REGULATION_OF_PHOSPHORYLATION** | | **19** | **-0.34552163** | **-0.9735527** | **0.513089** | **0.9476284** | **1** | **1104** | **tags=16%, list=8%, signal=17%** |
| **NUCLEOTIDE_EXCISION_REPAIR** | **NUCLEOTIDE_EXCISION_REPAIR** | | **19** | **-0.3482771** | **-0.97320986** | **0.5212355** | **0.9456055** | **1** | **3701** | **tags=37%, list=28%, signal=51%** |
| **POSITIVE_REGULATION_OF_CELLULAR_COMPONENT_ORGANIZATION_AND_BIOGENESIS** | **POSITIVE_REGULATION_OF_CELLULAR_COMPONENT_ORGANIZATION_AND_BIOGENESIS** | | **29** | **-0.32037088** | **-0.9722111** | **0.51266587** | **0.9454883** | **1** | **3689** | **tags=34%, list=28%, signal=48%** |
| **REGULATION_OF_CYCLIN_DEPENDENT_PROTEIN_KINASE_ACTIVITY** | **REGULATION_OF_CYCLIN_DEPENDENT_PROTEIN_KINASE_ACTIVITY** | | **39** | **-0.30134964** | **-0.9721382** | **0.5213873** | **0.94261724** | **1** | **2989** | **tags=18%, list=22%, signal=23%** |
| **REGULATION_OF_ANGIOGENESIS** | **REGULATION_OF_ANGIOGENESIS** | | **23** | **-0.32936057** | **-0.9701658** | **0.515** | **0.94543725** | **1** | **1626** | **tags=22%, list=12%, signal=25%** |
| **CDC42_PROTEIN_SIGNAL_TRANSDUCTION** | **CDC42_PROTEIN_SIGNAL_TRANSDUCTION** | | **8** | **-0.42963588** | **-0.9676509** | **0.5156695** | **0.9497786** | **1** | **3213** | **tags=50%, list=24%, signal=66%** |
| **REGULATION_OF_IMMUNE_RESPONSE** | **REGULATION_OF_IMMUNE_RESPONSE** | | **26** | **-0.32538047** | **-0.96188277** | **0.5463535** | **0.9636394** | **1** | **2028** | **tags=23%, list=15%, signal=27%** |
| **OLIGOSACCHARIDE_METABOLIC_PROCESS** | **OLIGOSACCHARIDE_METABOLIC_PROCESS** | | **10** | **-0.4055488** | **-0.9608434** | **0.53481895** | **0.9636121** | **1** | **1006** | **tags=20%, list=8%, signal=22%** |
| **MEIOSIS_I** | **MEIOSIS_I** |  | **16** | **-0.3576744** | **-0.96055216** | **0.53918916** | **0.9613569** | **1** | **4290** | **tags=50%, list=32%, signal=74%** |
| **POSITIVE_REGULATION_OF_CELL_ADHESION** | **POSITIVE_REGULATION_OF_CELL_ADHESION** | | **13** | **-0.38209578** | **-0.9605058** | **0.526971** | **0.95835173** | **1** | **708** | **tags=23%, list=5%, signal=24%** |
| **DNA_FRAGMENTATION_DURING_APOPTOSIS** | **DNA_FRAGMENTATION_DURING_APOPTOSIS** | | **11** | **-0.38616136** | **-0.95703006** | **0.53938586** | **0.96531177** | **1** | **2061** | **tags=27%, list=15%, signal=32%** |
| **FOCAL_ADHESION_FORMATION** | **FOCAL_ADHESION_FORMATION** | | **10** | **-0.40375322** | **-0.9549652** | **0.5329513** | **0.96822685** | **1** | **3330** | **tags=50%, list=25%, signal=67%** |
| **POSITIVE_REGULATION_OF_CELLULAR_METABOLIC_PROCESS** | **POSITIVE_REGULATION_OF_CELLULAR_METABOLIC_PROCESS** | | **177** | **-0.24718787** | **-0.9533838** | **0.6038697** | **0.9695847** | **1** | **2782** | **tags=20%, list=21%, signal=25%** |
| **COAGULATION** | **COAGULATION** | | **40** | **-0.28850088** | **-0.9521596** | **0.55903614** | **0.9700321** | **1** | **3709** | **tags=38%, list=28%, signal=52%** |
| **SYNAPTOGENESIS** | **SYNAPTOGENESIS** | | **10** | **-0.3933099** | **-0.95130223** | **0.54008436** | **0.9694058** | **1** | **3859** | **tags=40%, list=29%, signal=56%** |
| **PROTEIN_OLIGOMERIZATION** | **PROTEIN_OLIGOMERIZATION** | | **32** | **-0.30627754** | **-0.94778365** | **0.56711006** | **0.976269** | **1** | **4896** | **tags=41%, list=37%, signal=64%** |
| **POSITIVE_REGULATION_OF_CATALYTIC_ACTIVITY** | **POSITIVE_REGULATION_OF_CATALYTIC_ACTIVITY** | | **135** | **-0.24881771** | **-0.94664186** | **0.6030928** | **0.9762381** | **1** | **2076** | **tags=19%, list=16%, signal=23%** |
| **REGULATION_OF_PROTEIN_AMINO_ACID_PHOSPHORYLATION** | **REGULATION_OF_PROTEIN_AMINO_ACID_PHOSPHORYLATION** | | **20** | **-0.33229068** | **-0.9451528** | **0.53797466** | **0.97741014** | **1** | **1104** | **tags=15%, list=8%, signal=16%** |
| **REGULATION_OF_PROTEIN_MODIFICATION_PROCESS** | **REGULATION_OF_PROTEIN_MODIFICATION_PROCESS** | | **34** | **-0.29186612** | **-0.9448576** | **0.55058825** | **0.97508967** | **1** | **1263** | **tags=15%, list=9%, signal=16%** |
| **CHROMATIN_MODIFICATION** | **CHROMATIN_MODIFICATION** | | **41** | **-0.285505** | **-0.94433606** | **0.5659277** | **0.9734766** | **1** | **5706** | **tags=49%, list=43%, signal=85%** |
| **REGULATION_OF_CELLULAR_PROTEIN_METABOLIC_PROCESS** | **REGULATION_OF_CELLULAR_PROTEIN_METABOLIC_PROCESS** | | **123** | **-0.25027823** | **-0.9414613** | **0.61875** | **0.97844756** | **1** | **1344** | **tags=13%, list=10%, signal=14%** |
| **KERATINOCYTE_DIFFERENTIATION** | **KERATINOCYTE_DIFFERENTIATION** | | **13** | **-0.37380302** | **-0.940345** | **0.56413615** | **0.97843945** | **1** | **2142** | **tags=23%, list=16%, signal=27%** |
| **CELL_CELL_ADHESION** | **CELL_CELL_ADHESION** | | **69** | **-0.26780945** | **-0.9399635** | **0.5929978** | **0.97648823** | **1** | **2754** | **tags=22%, list=21%, signal=27%** |
| **HEME_BIOSYNTHETIC_PROCESS** | **HEME_BIOSYNTHETIC_PROCESS** | | **10** | **-0.3904085** | **-0.9359103** | **0.56340057** | **0.9845077** | **1** | **3442** | **tags=40%, list=26%, signal=54%** |
| **RHYTHMIC_PROCESS** | **RHYTHMIC_PROCESS** | | **22** | **-0.32847187** | **-0.93483335** | **0.5640051** | **0.984464** | **1** | **2725** | **tags=32%, list=20%, signal=40%** |
| **AMYLOID_PRECURSOR_PROTEIN_METABOLIC_PROCESS** | **AMYLOID_PRECURSOR_PROTEIN_METABOLIC_PROCESS** | | **8** | **-0.42431006** | **-0.932608** | **0.57363254** | **0.9875524** | **1** | **2122** | **tags=25%, list=16%, signal=30%** |
| **AMINO_ACID_DERIVATIVE_METABOLIC_PROCESS** | **AMINO_ACID_DERIVATIVE_METABOLIC_PROCESS** | | **23** | **-0.3212574** | **-0.9323994** | **0.5816327** | **0.98505795** | **1** | **4990** | **tags=48%, list=37%, signal=76%** |
| **CHROMATIN_ASSEMBLY_OR_DISASSEMBLY** | **CHROMATIN_ASSEMBLY_OR_DISASSEMBLY** | | **25** | **-0.30805293** | **-0.93069166** | **0.57019705** | **0.98671365** | **1** | **1950** | **tags=16%, list=15%, signal=19%** |
| **BLOOD_COAGULATION** | **BLOOD_COAGULATION** | | **40** | **-0.28850088** | **-0.9261553** | **0.5953757** | **0.99586076** | **1** | **3709** | **tags=38%, list=28%, signal=52%** |
| **REGULATION_OF_ANATOMICAL_STRUCTURE_MORPHOGENESIS** | **REGULATION_OF_ANATOMICAL_STRUCTURE_MORPHOGENESIS** | | **18** | **-0.33244887** | **-0.92401266** | **0.5873418** | **0.99847907** | **1** | **2786** | **tags=33%, list=21%, signal=42%** |
| **TUBE_DEVELOPMENT** | **TUBE_DEVELOPMENT** | | **15** | **-0.35002512** | **-0.92231774** | **0.5676741** | **0.9998934** | **1** | **2768** | **tags=33%, list=21%, signal=42%** |
| **INTERPHASE_OF_MITOTIC_CELL_CYCLE** | **INTERPHASE_OF_MITOTIC_CELL_CYCLE** | | **53** | **-0.2726945** | **-0.9215722** | **0.6165158** | **0.9988584** | **1** | **3375** | **tags=26%, list=25%, signal=35%** |
| **REGULATION_OF_ACTIN_POLYMERIZATION_AND_OR_DEPOLYMERIZATION** | **REGULATION_OF_ACTIN_POLYMERIZATION_AND_OR_DEPOLYMERIZATION** | | **11** | **-0.3729533** | **-0.92082334** | **0.57342654** | **0.997735** | **1** | **149** | **tags=9%, list=1%, signal=9%** |
| **GENERATION_OF_PRECURSOR_METABOLITES_AND_ENERGY** | **GENERATION_OF_PRECURSOR_METABOLITES_AND_ENERGY** | | **118** | **-0.24419026** | **-0.9203645** | **0.6511387** | **0.99584264** | **1** | **3467** | **tags=25%, list=26%, signal=33%** |
| **HEMOSTASIS** | **HEMOSTASIS** | | **45** | **-0.27898467** | **-0.91629505** | **0.61899316** | **1** | **1** | **3837** | **tags=38%, list=29%, signal=53%** |
| **POSITIVE_REGULATION_OF_CYTOSKELETON_ORGANIZATION_AND_BIOGENESIS** | **POSITIVE_REGULATION_OF_CYTOSKELETON_ORGANIZATION_AND_BIOGENESIS** | | **9** | **-0.3889092** | **-0.91450214** | **0.5754986** | **1** | **1** | **4872** | **tags=44%, list=37%, signal=70%** |
| **MACROMOLECULAR_COMPLEX_DISASSEMBLY** | **MACROMOLECULAR_COMPLEX_DISASSEMBLY** | | **14** | **-0.34910828** | **-0.9125431** | **0.58710563** | **1** | **1** | **3450** | **tags=36%, list=26%, signal=48%** |
| **MYOBLAST_DIFFERENTIATION** | **MYOBLAST_DIFFERENTIATION** | | **16** | **-0.33234662** | **-0.90637916** | **0.5991957** | **1** | **1** | **3245** | **tags=31%, list=24%, signal=41%** |
| **MITOTIC_SPINDLE_ORGANIZATION_AND_BIOGENESIS** | **MITOTIC_SPINDLE_ORGANIZATION_AND_BIOGENESIS** | | **9** | **-0.39260086** | **-0.90529996** | **0.61167884** | **1** | **1** | **759** | **tags=11%, list=6%, signal=12%** |
| **POSITIVE_REGULATION_OF_PHOSPHATE_METABOLIC_PROCESS** | **POSITIVE_REGULATION_OF_PHOSPHATE_METABOLIC_PROCESS** | | **21** | **-0.31496975** | **-0.90459645** | **0.6066411** | **1** | **1** | **1104** | **tags=14%, list=8%, signal=16%** |
| **PROTEIN_UBIQUITINATION** | **PROTEIN_UBIQUITINATION** | | **34** | **-0.28643385** | **-0.90451765** | **0.6157407** | **1** | **1** | **1459** | **tags=15%, list=11%, signal=16%** |
| **REGULATION_OF_PROTEIN_METABOLIC_PROCESS** | **REGULATION_OF_PROTEIN_METABOLIC_PROCESS** | | **134** | **-0.23821008** | **-0.9024788** | **0.68508863** | **1** | **1** | **2335** | **tags=17%, list=18%, signal=21%** |
| **NEGATIVE_REGULATION_OF_APOPTOSIS** | **NEGATIVE_REGULATION_OF_APOPTOSIS** | | **130** | **-0.23515633** | **-0.9012045** | **0.7003122** | **1** | **1** | **2081** | **tags=17%, list=16%, signal=20%** |
| **RESPONSE_TO_CARBOHYDRATE_STIMULUS** | **RESPONSE_TO_CARBOHYDRATE_STIMULUS** | | **10** | **-0.37885988** | **-0.90094846** | **0.6027972** | **1** | **1** | **3472** | **tags=40%, list=26%, signal=54%** |
| **POSITIVE_REGULATION_OF_DNA_METABOLIC_PROCESS** | **POSITIVE_REGULATION_OF_DNA_METABOLIC_PROCESS** | | **10** | **-0.375273** | **-0.89977753** | **0.6216216** | **1** | **1** | **1263** | **tags=20%, list=9%, signal=22%** |
| **NEGATIVE_REGULATION_OF_DEVELOPMENTAL_PROCESS** | **NEGATIVE_REGULATION_OF_DEVELOPMENTAL_PROCESS** | | **169** | **-0.23322044** | **-0.8988254** | **0.7329949** | **1** | **1** | **2081** | **tags=17%, list=16%, signal=20%** |
| **CELLULAR_CARBOHYDRATE_METABOLIC_PROCESS** | **CELLULAR_CARBOHYDRATE_METABOLIC_PROCESS** | | **104** | **-0.24110603** | **-0.89757663** | **0.67432153** | **1** | **1** | **3426** | **tags=26%, list=26%, signal=35%** |
| **PROTEIN_COMPLEX_DISASSEMBLY** | **PROTEIN_COMPLEX_DISASSEMBLY** | | **14** | **-0.34910828** | **-0.8966279** | **0.6167582** | **1** | **1** | **3450** | **tags=36%, list=26%, signal=48%** |
| **POSITIVE_REGULATION_OF_T_CELL_PROLIFERATION** | **POSITIVE_REGULATION_OF_T_CELL_PROLIFERATION** | | **12** | **-0.3536224** | **-0.8957907** | **0.62415195** | **1** | **1** | **608** | **tags=17%, list=5%, signal=17%** |
| **RRNA_PROCESSING** | **RRNA_PROCESSING** | | **8** | **-0.39839652** | **-0.89482486** | **0.60755813** | **1** | **1** | **3973** | **tags=50%, list=30%, signal=71%** |
| **RESPONSE_TO_EXTERNAL_STIMULUS** | **RESPONSE_TO_EXTERNAL_STIMULUS** | | **265** | **-0.22501993** | **-0.89390165** | **0.7713998** | **1** | **1** | **2251** | **tags=19%, list=17%, signal=23%** |
| **ACTIN_FILAMENT_POLYMERIZATION** | **ACTIN_FILAMENT_POLYMERIZATION** | | **11** | **-0.36473116** | **-0.89315885** | **0.6286509** | **1** | **1** | **149** | **tags=9%, list=1%, signal=9%** |
| **REGULATION_OF_TRANSCRIPTION_FACTOR_ACTIVITY** | **REGULATION_OF_TRANSCRIPTION_FACTOR_ACTIVITY** | | **27** | **-0.29547453** | **-0.8892531** | **0.65609753** | **1** | **1** | **1344** | **tags=19%, list=10%, signal=21%** |
| **RESPONSE_TO_TEMPERATURE_STIMULUS** | **RESPONSE_TO_TEMPERATURE_STIMULUS** | | **16** | **-0.3317969** | **-0.88845235** | **0.6394102** | **1** | **1** | **3564** | **tags=38%, list=27%, signal=51%** |
| **GLAND_DEVELOPMENT** | **GLAND_DEVELOPMENT** | | **12** | **-0.35286987** | **-0.88715553** | **0.61657304** | **1** | **1** | **1941** | **tags=25%, list=15%, signal=29%** |
| **ENERGY_DERIVATION_BY_OXIDATION_OF_ORGANIC_COMPOUNDS** | **ENERGY_DERIVATION_BY_OXIDATION_OF_ORGANIC_COMPOUNDS** | | **37** | **-0.27508706** | **-0.8861409** | **0.6556992** | **1** | **1** | **794** | **tags=11%, list=6%, signal=11%** |
| **NEGATIVE_REGULATION_OF_PROGRAMMED_CELL_DEATH** | **NEGATIVE_REGULATION_OF_PROGRAMMED_CELL_DEATH** | | **131** | **-0.23229371** | **-0.88535005** | **0.72164947** | **1** | **1** | **2081** | **tags=17%, list=16%, signal=20%** |
| **MITOCHONDRIAL_MEMBRANE_ORGANIZATION_AND_BIOGENESIS** | **MITOCHONDRIAL_MEMBRANE_ORGANIZATION_AND_BIOGENESIS** | | **9** | **-0.37794754** | **-0.88489926** | **0.61174786** | **1** | **1** | **2926** | **tags=22%, list=22%, signal=28%** |
| **MULTI_ORGANISM_PROCESS** | **MULTI_ORGANISM_PROCESS** | | **130** | **-0.2351495** | **-0.88336843** | **0.725756** | **1** | **1** | **3011** | **tags=26%, list=23%, signal=33%** |
| **CARBOHYDRATE_BIOSYNTHETIC_PROCESS** | **CARBOHYDRATE_BIOSYNTHETIC_PROCESS** | | **34** | **-0.27807084** | **-0.8829747** | **0.64396656** | **1** | **1** | **3396** | **tags=29%, list=25%, signal=39%** |
| **POSITIVE_REGULATION_OF_DNA_BINDING** | **POSITIVE_REGULATION_OF_DNA_BINDING** | | **18** | **-0.32014942** | **-0.88296986** | **0.6535948** | **1** | **1** | **3413** | **tags=33%, list=26%, signal=45%** |
| **ESTABLISHMENT_AND_OR_MAINTENANCE_OF_CHROMATIN_ARCHITECTURE** | **ESTABLISHMENT_AND_OR_MAINTENANCE_OF_CHROMATIN_ARCHITECTURE** | | **60** | **-0.25021955** | **-0.8788772** | **0.6773128** | **1** | **1** | **2920** | **tags=22%, list=22%, signal=28%** |
| **POSITIVE_REGULATION_OF_BINDING** | **POSITIVE_REGULATION_OF_BINDING** | | **18** | **-0.32014942** | **-0.87783736** | **0.6238532** | **1** | **1** | **3413** | **tags=33%, list=26%, signal=45%** |
| **REGULATION_OF_CELLULAR_COMPONENT_SIZE** | **REGULATION_OF_CELLULAR_COMPONENT_SIZE** | | **12** | **-0.35256** | **-0.8775764** | **0.6550765** | **1** | **1** | **149** | **tags=8%, list=1%, signal=8%** |
| **PROTEIN_MODIFICATION_BY_SMALL_PROTEIN_CONJUGATION** | **PROTEIN_MODIFICATION_BY_SMALL_PROTEIN_CONJUGATION** | | **36** | **-0.2738869** | **-0.8758659** | **0.65088755** | **1** | **1** | **1459** | **tags=14%, list=11%, signal=16%** |
| **PROTEIN_IMPORT_INTO_NUCLEUS_TRANSLOCATION** | **PROTEIN_IMPORT_INTO_NUCLEUS_TRANSLOCATION** | | **11** | **-0.35927954** | **-0.875284** | **0.64909846** | **1** | **1** | **2911** | **tags=36%, list=22%, signal=46%** |
| **RESPONSE_TO_HEAT** | **RESPONSE_TO_HEAT** | | **10** | **-0.36947787** | **-0.8740013** | **0.62237763** | **1** | **1** | **3472** | **tags=40%, list=26%, signal=54%** |
| **BILE_ACID_METABOLIC_PROCESS** | **BILE_ACID_METABOLIC_PROCESS** | | **9** | **-0.3723537** | **-0.87344694** | **0.64525545** | **1** | **1** | **2134** | **tags=33%, list=16%, signal=40%** |
| **AMINO_ACID_DERIVATIVE_BIOSYNTHETIC_PROCESS** | **AMINO_ACID_DERIVATIVE_BIOSYNTHETIC_PROCESS** | | **10** | **-0.3639452** | **-0.8722053** | **0.64131993** | **1** | **1** | **3661** | **tags=40%, list=27%, signal=55%** |
| **REGULATION_OF_G_PROTEIN_COUPLED_RECEPTOR_PROTEIN_SIGNALING_PATHWAY** | **REGULATION_OF_G_PROTEIN_COUPLED_RECEPTOR_PROTEIN_SIGNALING_PATHWAY** | | **23** | **-0.29977775** | **-0.8717666** | **0.65125** | **1** | **1** | **1602** | **tags=17%, list=12%, signal=20%** |
| **REGULATION_OF_ACTIN_FILAMENT_LENGTH** | **REGULATION_OF_ACTIN_FILAMENT_LENGTH** | | **12** | **-0.35256** | **-0.87011343** | **0.629681** | **1** | **1** | **149** | **tags=8%, list=1%, signal=8%** |
| **NEGATIVE_REGULATION_OF_TRANSLATION** | **NEGATIVE_REGULATION_OF_TRANSLATION** | | **17** | **-0.31754568** | **-0.8693963** | **0.64341086** | **1** | **1** | **1767** | **tags=24%, list=13%, signal=27%** |
| **REGULATION_OF_MOLECULAR_FUNCTION** | **REGULATION_OF_MOLECULAR_FUNCTION** | | **265** | **-0.22023846** | **-0.86816525** | **0.82912034** | **1** | **1** | **2139** | **tags=17%, list=16%, signal=20%** |
| **MUSCLE_DEVELOPMENT** | **MUSCLE_DEVELOPMENT** | | **85** | **-0.24144545** | **-0.867768** | **0.7177849** | **1** | **1** | **4324** | **tags=36%, list=32%, signal=54%** |
| **RRNA_METABOLIC_PROCESS** | **RRNA_METABOLIC_PROCESS** | | **9** | **-0.37995568** | **-0.86579996** | **0.65680474** | **1** | **1** | **5364** | **tags=67%, list=40%, signal=112%** |
| **NITROGEN_COMPOUND_BIOSYNTHETIC_PROCESS** | **NITROGEN_COMPOUND_BIOSYNTHETIC_PROCESS** | | **25** | **-0.29265165** | **-0.8657698** | **0.67125** | **1** | **1** | **4976** | **tags=36%, list=37%, signal=57%** |
| **AMINO_ACID_METABOLIC_PROCESS** | **AMINO_ACID_METABOLIC_PROCESS** | | **72** | **-0.24310382** | **-0.8630683** | **0.7053669** | **1** | **1** | **4592** | **tags=36%, list=34%, signal=55%** |
| **MUSCLE_CELL_DIFFERENTIATION** | **MUSCLE_CELL_DIFFERENTIATION** | | **19** | **-0.30879188** | **-0.86164653** | **0.69419354** | **1** | **1** | **3391** | **tags=32%, list=25%, signal=42%** |
| **ANTI_APOPTOSIS** | **ANTI_APOPTOSIS** | | **103** | **-0.23374574** | **-0.8597001** | **0.7539432** | **1** | **1** | **2081** | **tags=17%, list=16%, signal=19%** |
| **REGULATION_OF_CATALYTIC_ACTIVITY** | **REGULATION_OF_CATALYTIC_ACTIVITY** | | **231** | **-0.21701889** | **-0.85660475** | **0.8230536** | **1** | **1** | **2139** | **tags=17%, list=16%, signal=20%** |
| **HEME_METABOLIC_PROCESS** | **HEME_METABOLIC_PROCESS** | | **11** | **-0.35342368** | **-0.85554534** | **0.65836793** | **1** | **1** | **3442** | **tags=36%, list=26%, signal=49%** |
| **POSITIVE_REGULATION_OF_NUCLEOBASENUCLEOSIDENUCLEOTIDE_AND_NUCLEIC_ACID_METABOLIC_PROCESS** | **POSITIVE_REGULATION_OF_NUCLEOBASENUCLEOSIDENUCLEOTIDE_AND_NUCLEIC_ACID_METABOLIC_PROCESS** | | **118** | **-0.22822979** | **-0.8553556** | **0.77211237** | **1** | **1** | **2920** | **tags=21%, list=22%, signal=27%** |
| **ADAPTIVE_IMMUNE_RESPONSE_GO_0002460** | **ADAPTIVE_IMMUNE_RESPONSE_GO_0002460** | | **19** | **-0.3040169** | **-0.8551987** | **0.6606684** | **1** | **1** | **2028** | **tags=21%, list=15%, signal=25%** |
| **TRANSMEMBRANE_RECEPTOR_PROTEIN_TYROSINE_KINASE_SIGNALING_PATHWAY** | **TRANSMEMBRANE_RECEPTOR_PROTEIN_TYROSINE_KINASE_SIGNALING_PATHWAY** | | **75** | **-0.23965073** | **-0.853514** | **0.7267876** | **1** | **1** | **5345** | **tags=45%, list=40%, signal=75%** |
| **GLYCOPROTEIN_BIOSYNTHETIC_PROCESS** | **GLYCOPROTEIN_BIOSYNTHETIC_PROCESS** | | **65** | **-0.24549967** | **-0.8499073** | **0.7350333** | **1** | **1** | **1711** | **tags=14%, list=13%, signal=16%** |
| **REGULATION_OF_MEMBRANE_POTENTIAL** | **REGULATION_OF_MEMBRANE_POTENTIAL** | | **13** | **-0.3313076** | **-0.849562** | **0.65847** | **1** | **1** | **2410** | **tags=23%, list=18%, signal=28%** |
| **CYCLIC_NUCLEOTIDE_METABOLIC_PROCESS** | **CYCLIC_NUCLEOTIDE_METABOLIC_PROCESS** | | **9** | **-0.3656198** | **-0.8486402** | **0.6752137** | **1** | **1** | **3194** | **tags=33%, list=24%, signal=44%** |
| **ADAPTIVE_IMMUNE_RESPONSE** | **ADAPTIVE_IMMUNE_RESPONSE** | | **20** | **-0.30360344** | **-0.84829605** | **0.7047497** | **1** | **1** | **2028** | **tags=20%, list=15%, signal=24%** |
| **NUCLEOBASENUCLEOSIDENUCLEOTIDE_AND_NUCLEIC_ACID_TRANSPORT** | **NUCLEOBASENUCLEOSIDENUCLEOTIDE_AND_NUCLEIC_ACID_TRANSPORT** | | **24** | **-0.2845263** | **-0.8474741** | **0.69433963** | **1** | **1** | **4168** | **tags=33%, list=31%, signal=48%** |
| **EMBRYONIC_MORPHOGENESIS** | **EMBRYONIC_MORPHOGENESIS** | | **14** | **-0.32932228** | **-0.84534377** | **0.6818792** | **1** | **1** | **3936** | **tags=36%, list=30%, signal=51%** |
| **ESTABLISHMENT_AND_OR_MAINTENANCE_OF_CELL_POLARITY** | **ESTABLISHMENT_AND_OR_MAINTENANCE_OF_CELL_POLARITY** | | **19** | **-0.30060095** | **-0.8426033** | **0.70298314** | **1** | **1** | **2137** | **tags=21%, list=16%, signal=25%** |
| **ONE_CARBON_COMPOUND_METABOLIC_PROCESS** | **ONE_CARBON_COMPOUND_METABOLIC_PROCESS** | | **24** | **-0.28219223** | **-0.8416254** | **0.68677795** | **1** | **1** | **2622** | **tags=21%, list=20%, signal=26%** |
| **SPINDLE_ORGANIZATION_AND_BIOGENESIS** | **SPINDLE_ORGANIZATION_AND_BIOGENESIS** | | **10** | **-0.35280958** | **-0.8412679** | **0.68151146** | **1** | **1** | **759** | **tags=10%, list=6%, signal=11%** |
| **CELLULAR_PROTEIN_COMPLEX_ASSEMBLY** | **CELLULAR_PROTEIN_COMPLEX_ASSEMBLY** | | **28** | **-0.27754465** | **-0.840399** | **0.7051597** | **1** | **1** | **5243** | **tags=50%, list=39%, signal=82%** |
| **GLYCEROPHOSPHOLIPID_METABOLIC_PROCESS** | **GLYCEROPHOSPHOLIPID_METABOLIC_PROCESS** | | **36** | **-0.26687655** | **-0.83967197** | **0.71664697** | **1** | **1** | **53** | **tags=6%, list=0%, signal=6%** |
| **GROWTH** | **GROWTH** |  | **56** | **-0.24470241** | **-0.8389812** | **0.737798** | **1** | **1** | **3802** | **tags=30%, list=29%, signal=42%** |
| **ORGAN_MORPHOGENESIS** | **ORGAN_MORPHOGENESIS** | | **125** | **-0.2218359** | **-0.83757526** | **0.8** | **1** | **1** | **3544** | **tags=28%, list=27%, signal=38%** |
| **POSITIVE_REGULATION_OF_TRANSCRIPTION** | **POSITIVE_REGULATION_OF_TRANSCRIPTION** | | **109** | **-0.2259551** | **-0.8357169** | **0.79748166** | **1** | **1** | **2920** | **tags=21%, list=22%, signal=27%** |
| **CELLULAR_COMPONENT_ASSEMBLY** | **CELLULAR_COMPONENT_ASSEMBLY** | | **236** | **-0.212192** | **-0.83405566** | **0.85439837** | **1** | **1** | **4918** | **tags=37%, list=37%, signal=57%** |
| **INTRACELLULAR_SIGNALING_CASCADE** | **INTRACELLULAR_SIGNALING_CASCADE** | | **555** | **-0.20547366** | **-0.83105564** | **0.934** | **1** | **1** | **2199** | **tags=17%, list=17%, signal=20%** |
| **MEMBRANE_LIPID_BIOSYNTHETIC_PROCESS** | **MEMBRANE_LIPID_BIOSYNTHETIC_PROCESS** | | **35** | **-0.25806105** | **-0.82942176** | **0.75357145** | **1** | **1** | **1070** | **tags=9%, list=8%, signal=9%** |
| **POSITIVE_REGULATION_OF_RNA_METABOLIC_PROCESS** | **POSITIVE_REGULATION_OF_RNA_METABOLIC_PROCESS** | | **95** | **-0.22509977** | **-0.827922** | **0.78723407** | **1** | **1** | **2920** | **tags=20%, list=22%, signal=25%** |
| **PROTEIN_PROCESSING** | **PROTEIN_PROCESSING** | | **40** | **-0.25600234** | **-0.8266899** | **0.7537923** | **1** | **1** | **4529** | **tags=33%, list=34%, signal=49%** |
| **REGULATION_OF_CELL_ADHESION** | **REGULATION_OF_CELL_ADHESION** | | **32** | **-0.26394337** | **-0.82662654** | **0.73741007** | **1** | **1** | **708** | **tags=13%, list=5%, signal=13%** |
| **PROTEIN_MATURATION** | **PROTEIN_MATURATION** | | **8** | **-0.37178436** | **-0.8257995** | **0.7136628** | **1** | **1** | **3843** | **tags=38%, list=29%, signal=53%** |
| **RESPONSE_TO_DRUG** | **RESPONSE_TO_DRUG** | | **20** | **-0.29223207** | **-0.82537234** | **0.7180784** | **1** | **1** | **2558** | **tags=25%, list=19%, signal=31%** |
| **REGULATION_OF_PHOSPHORYLATION** | **REGULATION_OF_PHOSPHORYLATION** | | **38** | **-0.25701484** | **-0.8239646** | **0.74112254** | **1** | **1** | **1104** | **tags=13%, list=8%, signal=14%** |
| **RESPONSE_TO_HYPOXIA** | **RESPONSE_TO_HYPOXIA** | | **25** | **-0.27501553** | **-0.82246786** | **0.73119605** | **1** | **1** | **1072** | **tags=16%, list=8%, signal=17%** |
| **CATABOLIC_PROCESS** | **CATABOLIC_PROCESS** | | **193** | **-0.21208526** | **-0.82238305** | **0.8446502** | **1** | **1** | **3418** | **tags=25%, list=26%, signal=34%** |
| **SUPEROXIDE_METABOLIC_PROCESS** | **SUPEROXIDE_METABOLIC_PROCESS** | | **8** | **-0.36353967** | **-0.8200146** | **0.7172996** | **1** | **1** | **3680** | **tags=50%, list=28%, signal=69%** |
| **IMMUNE_SYSTEM_DEVELOPMENT** | **IMMUNE_SYSTEM_DEVELOPMENT** | | **71** | **-0.2273227** | **-0.8159725** | **0.7903044** | **1** | **1** | **3883** | **tags=34%, list=29%, signal=47%** |
| **REGULATION_OF_PEPTIDYL_TYROSINE_PHOSPHORYLATION** | **REGULATION_OF_PEPTIDYL_TYROSINE_PHOSPHORYLATION** | | **13** | **-0.32167378** | **-0.8147038** | **0.7207703** | **1** | **1** | **1067** | **tags=15%, list=8%, signal=17%** |
| **SPHINGOID_METABOLIC_PROCESS** | **SPHINGOID_METABOLIC_PROCESS** | | **9** | **-0.3493775** | **-0.8107014** | **0.7136628** | **1** | **1** | **2062** | **tags=22%, list=15%, signal=26%** |
| **HEMOPOIETIC_OR_LYMPHOID_ORGAN_DEVELOPMENT** | **HEMOPOIETIC_OR_LYMPHOID_ORGAN_DEVELOPMENT** | | **68** | **-0.22835344** | **-0.80772144** | **0.79431075** | **1** | **1** | **3883** | **tags=34%, list=29%, signal=47%** |
| **ACTIVATION_OF_PROTEIN_KINASE_ACTIVITY** | **ACTIVATION_OF_PROTEIN_KINASE_ACTIVITY** | | **21** | **-0.2811166** | **-0.8045478** | **0.74129355** | **1** | **1** | **1807** | **tags=19%, list=14%, signal=22%** |
| **GLYCEROPHOSPHOLIPID_BIOSYNTHETIC_PROCESS** | **GLYCEROPHOSPHOLIPID_BIOSYNTHETIC_PROCESS** | | **24** | **-0.2731475** | **-0.8021889** | **0.75636363** | **1** | **1** | **15** | **tags=4%, list=0%, signal=4%** |
| **RIBOSOME_BIOGENESIS_AND_ASSEMBLY** | **RIBOSOME_BIOGENESIS_AND_ASSEMBLY** | | **10** | **-0.33547965** | **-0.80067295** | **0.7442857** | **1** | **1** | **6027** | **tags=70%, list=45%, signal=128%** |
| **PROTEIN_AMINO_ACID_N_LINKED_GLYCOSYLATION** | **PROTEIN_AMINO_ACID_N_LINKED_GLYCOSYLATION** | | **26** | **-0.26448092** | **-0.7962047** | **0.7714631** | **1** | **1** | **1358** | **tags=15%, list=10%, signal=17%** |
| **HEMOPOIESIS** | **HEMOPOIESIS** | | **66** | **-0.22417966** | **-0.7959022** | **0.8113839** | **1** | **1** | **3883** | **tags=33%, list=29%, signal=47%** |
| **SENSORY_ORGAN_DEVELOPMENT** | **SENSORY_ORGAN_DEVELOPMENT** | | **14** | **-0.3020039** | **-0.79430586** | **0.7536042** | **1** | **1** | **3759** | **tags=43%, list=28%, signal=60%** |
| **CELLULAR_CATABOLIC_PROCESS** | **CELLULAR_CATABOLIC_PROCESS** | | **182** | **-0.20272905** | **-0.794226** | **0.8840285** | **1** | **1** | **3418** | **tags=25%, list=26%, signal=33%** |
| **POSITIVE_REGULATION_OF_TRANSCRIPTIONDNA_DEPENDENT** | **POSITIVE_REGULATION_OF_TRANSCRIPTIONDNA_DEPENDENT** | | **93** | **-0.21501309** | **-0.79132193** | **0.84713376** | **1** | **1** | **2920** | **tags=19%, list=22%, signal=25%** |
| **OVULATION_CYCLE** | **OVULATION_CYCLE** | | **9** | **-0.34544265** | **-0.7902814** | **0.764539** | **1** | **1** | **4577** | **tags=56%, list=34%, signal=85%** |
| **HORMONE_SECRETION** | **HORMONE_SECRETION** | | **14** | **-0.29962447** | **-0.79002464** | **0.74834436** | **1** | **1** | **3139** | **tags=36%, list=24%, signal=47%** |
| **FATTY_ACID_BIOSYNTHETIC_PROCESS** | **FATTY_ACID_BIOSYNTHETIC_PROCESS** | | **14** | **-0.30310586** | **-0.7885601** | **0.7520325** | **1** | **1** | **395** | **tags=7%, list=3%, signal=7%** |
| **RESPONSE_TO_CHEMICAL_STIMULUS** | **RESPONSE_TO_CHEMICAL_STIMULUS** | | **266** | **-0.20078637** | **-0.786426** | **0.9264854** | **1** | **1** | **1971** | **tags=15%, list=15%, signal=18%** |
| **M_PHASE** | **M_PHASE** |  | **90** | **-0.21536516** | **-0.7852517** | **0.8370607** | **1** | **1** | **3458** | **tags=24%, list=26%, signal=33%** |
| **CERAMIDE_METABOLIC_PROCESS** | **CERAMIDE_METABOLIC_PROCESS** | | **8** | **-0.35394117** | **-0.784154** | **0.7643678** | **1** | **1** | **2062** | **tags=25%, list=15%, signal=30%** |
| **POSITIVE_REGULATION_OF_TRANSCRIPTION_FROM_RNA_POLYMERASE_II_PROMOTER** | **POSITIVE_REGULATION_OF_TRANSCRIPTION_FROM_RNA_POLYMERASE_II_PROMOTER** | | **53** | **-0.23101671** | **-0.7836878** | **0.83046967** | **1** | **1** | **1518** | **tags=15%, list=11%, signal=17%** |
| **INTERACTION_WITH_HOST** | **INTERACTION_WITH_HOST** | | **15** | **-0.29500192** | **-0.7832619** | **0.7689243** | **1** | **1** | **5245** | **tags=53%, list=39%, signal=88%** |
| **TRANSCRIPTION_FROM_RNA_POLYMERASE_II_PROMOTER** | **TRANSCRIPTION_FROM_RNA_POLYMERASE_II_PROMOTER** | | **399** | **-0.1947591** | **-0.78257644** | **0.95996** | **1** | **1** | **3008** | **tags=21%, list=23%, signal=26%** |
| **NEGATIVE_REGULATION_OF_GROWTH** | **NEGATIVE_REGULATION_OF_GROWTH** | | **33** | **-0.24641111** | **-0.78163874** | **0.78730905** | **1** | **1** | **796** | **tags=9%, list=6%, signal=10%** |
| **MICROTUBULE_CYTOSKELETON_ORGANIZATION_AND_BIOGENESIS** | **MICROTUBULE_CYTOSKELETON_ORGANIZATION_AND_BIOGENESIS** | | **31** | **-0.24835826** | **-0.7807559** | **0.801227** | **1** | **1** | **1078** | **tags=10%, list=8%, signal=11%** |
| **REGULATION_OF_CELL_DIFFERENTIATION** | **REGULATION_OF_CELL_DIFFERENTIATION** | | **45** | **-0.23796736** | **-0.780407** | **0.81114906** | **1** | **1** | **1795** | **tags=18%, list=13%, signal=20%** |
| **REGULATION_OF_HORMONE_SECRETION** | **REGULATION_OF_HORMONE_SECRETION** | | **12** | **-0.31361276** | **-0.77810925** | **0.77327937** | **1** | **1** | **3139** | **tags=33%, list=24%, signal=44%** |
| **TRANSCRIPTION_INITIATION** | **TRANSCRIPTION_INITIATION** | | **24** | **-0.26482123** | **-0.777084** | **0.78625953** | **1** | **1** | **955** | **tags=13%, list=7%, signal=13%** |
| **REGULATION_OF_T_CELL_PROLIFERATION** | **REGULATION_OF_T_CELL_PROLIFERATION** | | **14** | **-0.29150236** | **-0.7737615** | **0.7830688** | **1** | **1** | **608** | **tags=14%, list=5%, signal=15%** |
| **DNA_REPAIR** | **DNA_REPAIR** | | **115** | **-0.20534536** | **-0.7725075** | **0.87447697** | **1** | **1** | **4528** | **tags=34%, list=34%, signal=51%** |
| **RNA_ELONGATION** | **RNA_ELONGATION** | | **10** | **-0.32424703** | **-0.77106726** | **0.7763713** | **1** | **1** | **4060** | **tags=50%, list=30%, signal=72%** |
| **CELLULAR_PROTEIN_COMPLEX_DISASSEMBLY** | **CELLULAR_PROTEIN_COMPLEX_DISASSEMBLY** | | **13** | **-0.30037835** | **-0.7709505** | **0.7718579** | **1** | **1** | **3450** | **tags=31%, list=26%, signal=41%** |
| **T_CELL_PROLIFERATION** | **T_CELL_PROLIFERATION** | | **16** | **-0.28724322** | **-0.7705797** | **0.78125** | **1** | **1** | **608** | **tags=13%, list=5%, signal=13%** |
| **MACROMOLECULAR_COMPLEX_ASSEMBLY** | **MACROMOLECULAR_COMPLEX_ASSEMBLY** | | **219** | **-0.19629766** | **-0.76995385** | **0.909919** | **1** | **1** | **4918** | **tags=36%, list=37%, signal=56%** |
| **REGULATION_OF_DNA_BINDING** | **REGULATION_OF_DNA_BINDING** | | **34** | **-0.24085544** | **-0.76965415** | **0.8246225** | **1** | **1** | **1344** | **tags=15%, list=10%, signal=16%** |
| **REGULATION_OF_GROWTH** | **REGULATION_OF_GROWTH** | | **47** | **-0.22777277** | **-0.7676313** | **0.8344671** | **1** | **1** | **3802** | **tags=30%, list=29%, signal=42%** |
| **CELL_CYCLE_PHASE** | **CELL_CYCLE_PHASE** | | **140** | **-0.19883919** | **-0.7672647** | **0.8931141** | **1** | **1** | **2908** | **tags=20%, list=22%, signal=25%** |
| **NUCLEAR_ORGANIZATION_AND_BIOGENESIS** | **NUCLEAR_ORGANIZATION_AND_BIOGENESIS** | | **21** | **-0.26598912** | **-0.7618541** | **0.78045684** | **1** | **1** | **2061** | **tags=19%, list=15%, signal=22%** |
| **REGULATION_OF_TRANSLATION** | **REGULATION_OF_TRANSLATION** | | **64** | **-0.21750541** | **-0.7618332** | **0.86383927** | **1** | **1** | **2551** | **tags=19%, list=19%, signal=23%** |
| **TISSUE_MORPHOGENESIS** | **TISSUE_MORPHOGENESIS** | | **13** | **-0.3034636** | **-0.75983185** | **0.8013245** | **1** | **1** | **3701** | **tags=46%, list=28%, signal=64%** |
| **ACTIN_CYTOSKELETON_ORGANIZATION_AND_BIOGENESIS** | **ACTIN_CYTOSKELETON_ORGANIZATION_AND_BIOGENESIS** | | **86** | **-0.20565896** | **-0.74910444** | **0.8851931** | **1** | **1** | **2801** | **tags=20%, list=21%, signal=25%** |
| **G2_M_TRANSITION_OF_MITOTIC_CELL_CYCLE** | **G2_M_TRANSITION_OF_MITOTIC_CELL_CYCLE** | | **12** | **-0.29692376** | **-0.7460283** | **0.8076923** | **1** | **1** | **3916** | **tags=42%, list=29%, signal=59%** |
| **CELL_DEVELOPMENT** | **CELL_DEVELOPMENT** | | **498** | **-0.18354917** | **-0.7426245** | **0.991** | **1** | **1** | **2122** | **tags=15%, list=16%, signal=17%** |
| **S_PHASE** | **S_PHASE** |  | **12** | **-0.29369706** | **-0.74249405** | **0.79014087** | **1** | **1** | **894** | **tags=17%, list=7%, signal=18%** |
| **BIOSYNTHETIC_PROCESS** | **BIOSYNTHETIC_PROCESS** | | **379** | **-0.1839651** | **-0.7399336** | **0.9799197** | **1** | **1** | **4183** | **tags=26%, list=31%, signal=36%** |
| **CELL_ACTIVATION** | **CELL_ACTIVATION** | | **63** | **-0.21360391** | **-0.738513** | **0.875** | **1** | **1** | **2911** | **tags=22%, list=22%, signal=28%** |
| **POSITIVE_REGULATION_OF_SECRETION** | **POSITIVE_REGULATION_OF_SECRETION** | | **13** | **-0.29226977** | **-0.7369735** | **0.8070652** | **1** | **1** | **3391** | **tags=46%, list=25%, signal=62%** |
| **TRANSCRIPTION_DNA_DEPENDENT** | **TRANSCRIPTION_DNA_DEPENDENT** | | **538** | **-0.182077** | **-0.73442346** | **0.993** | **1** | **1** | **3008** | **tags=20%, list=23%, signal=25%** |
| **PROTEIN_AMINO_ACID_LIPIDATION** | **PROTEIN_AMINO_ACID_LIPIDATION** | | **21** | **-0.25916725** | **-0.7339114** | **0.82806575** | **1** | **1** | **3946** | **tags=24%, list=30%, signal=34%** |
| **INTERPHASE** | **INTERPHASE** | | **58** | **-0.21522388** | **-0.7336146** | **0.8815061** | **1** | **1** | **3375** | **tags=24%, list=25%, signal=32%** |
| **RNA_BIOSYNTHETIC_PROCESS** | **RNA_BIOSYNTHETIC_PROCESS** | | **539** | **-0.18163645** | **-0.7329842** | **0.997** | **1** | **1** | **3008** | **tags=20%, list=23%, signal=25%** |
| **POSITIVE_REGULATION_OF_MULTICELLULAR_ORGANISMAL_PROCESS** | **POSITIVE_REGULATION_OF_MULTICELLULAR_ORGANISMAL_PROCESS** | | **52** | **-0.21770604** | **-0.73239803** | **0.89431816** | **1** | **1** | **2522** | **tags=17%, list=19%, signal=21%** |
| **RESPONSE_TO_WOUNDING** | **RESPONSE_TO_WOUNDING** | | **161** | **-0.18987735** | **-0.7321771** | **0.9371782** | **1** | **1** | **3753** | **tags=29%, list=28%, signal=39%** |
| **VASCULATURE_DEVELOPMENT** | **VASCULATURE_DEVELOPMENT** | | **46** | **-0.2182583** | **-0.732071** | **0.8661234** | **1** | **1** | **1626** | **tags=15%, list=12%, signal=17%** |
| **MITOTIC_CELL_CYCLE** | **MITOTIC_CELL_CYCLE** | | **124** | **-0.19386974** | **-0.73132837** | **0.9217935** | **1** | **1** | **3509** | **tags=21%, list=26%, signal=28%** |
| **CELLULAR_BIOSYNTHETIC_PROCESS** | **CELLULAR_BIOSYNTHETIC_PROCESS** | | **259** | **-0.18519205** | **-0.7306574** | **0.97088355** | **1** | **1** | **4088** | **tags=26%, list=31%, signal=37%** |
| **GLYCOPROTEIN_METABOLIC_PROCESS** | **GLYCOPROTEIN_METABOLIC_PROCESS** | | **79** | **-0.20374459** | **-0.7305285** | **0.9139785** | **1** | **1** | **2122** | **tags=14%, list=16%, signal=16%** |
| **CELL_CYCLE_PROCESS** | **CELL_CYCLE_PROCESS** | | **156** | **-0.19015478** | **-0.7298835** | **0.9392379** | **1** | **1** | **2908** | **tags=19%, list=22%, signal=24%** |
| **MACROMOLECULE_BIOSYNTHETIC_PROCESS** | **MACROMOLECULE_BIOSYNTHETIC_PROCESS** | | **251** | **-0.18463267** | **-0.7285871** | **0.9667003** | **1** | **1** | **4183** | **tags=26%, list=31%, signal=37%** |
| **PHOSPHOINOSITIDE_METABOLIC_PROCESS** | **PHOSPHOINOSITIDE_METABOLIC_PROCESS** | | **22** | **-0.25299948** | **-0.72542477** | **0.8487805** | **1** | **1** | **53** | **tags=5%, list=0%, signal=5%** |
| **CELL_PROLIFERATION_GO_0008283** | **CELL_PROLIFERATION_GO_0008283** | | **448** | **-0.17830302** | **-0.7186521** | **0.992993** | **1** | **1** | **4237** | **tags=27%, list=32%, signal=39%** |
| **PROTEIN_MODIFICATION_PROCESS** | **PROTEIN_MODIFICATION_PROCESS** | | **531** | **-0.17719018** | **-0.71834004** | **0.993** | **1** | **1** | **3131** | **tags=20%, list=24%, signal=25%** |
| **RNA_CATABOLIC_PROCESS** | **RNA_CATABOLIC_PROCESS** | | **21** | **-0.24768768** | **-0.715893** | **0.84505206** | **1** | **1** | **1109** | **tags=14%, list=8%, signal=16%** |
| **CELLULAR_RESPONSE_TO_STRESS** | **CELLULAR_RESPONSE_TO_STRESS** | | **8** | **-0.31717592** | **-0.7156923** | **0.83829784** | **1** | **1** | **920** | **tags=13%, list=7%, signal=13%** |
| **DOUBLE_STRAND_BREAK_REPAIR** | **DOUBLE_STRAND_BREAK_REPAIR** | | **21** | **-0.24810329** | **-0.7121761** | **0.8593955** | **1** | **1** | **2459** | **tags=19%, list=18%, signal=23%** |
| **ACTIN_FILAMENT_BASED_PROCESS** | **ACTIN_FILAMENT_BASED_PROCESS** | | **96** | **-0.1932877** | **-0.7105603** | **0.93333334** | **1** | **1** | **2801** | **tags=19%, list=21%, signal=24%** |
| **POST_TRANSLATIONAL_PROTEIN_MODIFICATION** | **POST_TRANSLATIONAL_PROTEIN_MODIFICATION** | | **399** | **-0.17716552** | **-0.7102039** | **0.993994** | **1** | **1** | **3239** | **tags=22%, list=24%, signal=28%** |
| **ORGANELLE_ORGANIZATION_AND_BIOGENESIS** | **ORGANELLE_ORGANIZATION_AND_BIOGENESIS** | | **386** | **-0.17440262** | **-0.7042343** | **0.987** | **1** | **1** | **3479** | **tags=20%, list=26%, signal=27%** |
| **BIOPOLYMER_MODIFICATION** | **BIOPOLYMER_MODIFICATION** | | **546** | **-0.1734359** | **-0.69950163** | **0.997998** | **1** | **1** | **3131** | **tags=20%, list=24%, signal=25%** |
| **NEGATIVE_REGULATION_OF_CELL_ADHESION** | **NEGATIVE_REGULATION_OF_CELL_ADHESION** | | **16** | **-0.26076525** | **-0.69876766** | **0.8759791** | **1** | **1** | **2754** | **tags=19%, list=21%, signal=24%** |
| **DNA_REPLICATION** | **DNA_REPLICATION** | | **85** | **-0.19242373** | **-0.69848174** | **0.9409871** | **1** | **1** | **4208** | **tags=31%, list=32%, signal=44%** |
| **ANGIOGENESIS** | **ANGIOGENESIS** | | **40** | **-0.21493325** | **-0.69529337** | **0.89148194** | **1** | **1** | **1626** | **tags=15%, list=12%, signal=17%** |
| **POSITIVE_REGULATION_OF_CYTOKINE_PRODUCTION** | **POSITIVE_REGULATION_OF_CYTOKINE_PRODUCTION** | | **12** | **-0.27795476** | **-0.69249845** | **0.86885244** | **1** | **1** | **3883** | **tags=33%, list=29%, signal=47%** |
| **REGULATION_OF_ENDOCYTOSIS** | **REGULATION_OF_ENDOCYTOSIS** | | **14** | **-0.26631016** | **-0.6894169** | **0.8591731** | **1** | **1** | **3689** | **tags=29%, list=28%, signal=39%** |
| **CELLULAR_RESPONSE_TO_EXTRACELLULAR_STIMULUS** | **CELLULAR_RESPONSE_TO_EXTRACELLULAR_STIMULUS** | | **8** | **-0.31067568** | **-0.6879582** | **0.8713018** | **1** | **1** | **5049** | **tags=50%, list=38%, signal=80%** |
| **PHOSPHOLIPID_BIOSYNTHETIC_PROCESS** | **PHOSPHOLIPID_BIOSYNTHETIC_PROCESS** | | **30** | **-0.22248569** | **-0.68682593** | **0.9036861** | **1** | **1** | **15** | **tags=3%, list=0%, signal=3%** |
| **AMINE_METABOLIC_PROCESS** | **AMINE_METABOLIC_PROCESS** | | **125** | **-0.1817556** | **-0.68489987** | **0.960499** | **1** | **1** | **5191** | **tags=38%, list=39%, signal=62%** |
| **DNA_DAMAGE_RESPONSESIGNAL_TRANSDUCTION** | **DNA_DAMAGE_RESPONSESIGNAL_TRANSDUCTION** | | **29** | **-0.22157332** | **-0.68381137** | **0.9073405** | **1** | **1** | **1574** | **tags=14%, list=12%, signal=16%** |
| **TRANSCRIPTION** | **TRANSCRIPTION** | | **627** | **-0.16766731** | **-0.681456** | **1** | **1** | **1** | **3014** | **tags=20%, list=23%, signal=24%** |
| **RESPONSE_TO_DNA_DAMAGE_STIMULUS** | **RESPONSE_TO_DNA_DAMAGE_STIMULUS** | | **144** | **-0.17881876** | **-0.68019325** | **0.9670103** | **1** | **1** | **4528** | **tags=30%, list=34%, signal=45%** |
| **EMBRYONIC_DEVELOPMENT** | **EMBRYONIC_DEVELOPMENT** | | **46** | **-0.20402473** | **-0.67941964** | **0.90867054** | **1** | **1** | **3498** | **tags=28%, list=26%, signal=38%** |
| **EPITHELIAL_TO_MESENCHYMAL_TRANSITION** | **EPITHELIAL_TO_MESENCHYMAL_TRANSITION** | | **9** | **-0.29155293** | **-0.6787547** | **0.8611898** | **1** | **1** | **4242** | **tags=33%, list=32%, signal=49%** |
| **BASE_EXCISION_REPAIR** | **BASE_EXCISION_REPAIR** | | **15** | **-0.2520006** | **-0.6769173** | **0.86990803** | **1** | **1** | **4778** | **tags=40%, list=36%, signal=62%** |
| **NUCLEOTIDE_BIOSYNTHETIC_PROCESS** | **NUCLEOTIDE_BIOSYNTHETIC_PROCESS** | | **16** | **-0.25130427** | **-0.67076313** | **0.88384515** | **1** | **1** | **3052** | **tags=19%, list=23%, signal=24%** |
| **INSULIN_RECEPTOR_SIGNALING_PATHWAY** | **INSULIN_RECEPTOR_SIGNALING_PATHWAY** | | **16** | **-0.248328** | **-0.6703559** | **0.88743454** | **1** | **1** | **5573** | **tags=56%, list=42%, signal=97%** |
| **REGULATION_OF_METABOLIC_PROCESS** | **REGULATION_OF_METABOLIC_PROCESS** | | **634** | **-0.16450329** | **-0.6699211** | **0.999** | **1** | **1** | **2947** | **tags=18%, list=22%, signal=22%** |
| **CYTOSKELETON_ORGANIZATION_AND_BIOGENESIS** | **CYTOSKELETON_ORGANIZATION_AND_BIOGENESIS** | | **177** | **-0.17268807** | **-0.66948295** | **0.9775051** | **1** | **1** | **3479** | **tags=21%, list=26%, signal=28%** |
| **ANATOMICAL_STRUCTURE_FORMATION** | **ANATOMICAL_STRUCTURE_FORMATION** | | **48** | **-0.1981398** | **-0.6684504** | **0.9397042** | **1** | **1** | **3414** | **tags=25%, list=26%, signal=33%** |
| **RESPONSE_TO_ENDOGENOUS_STIMULUS** | **RESPONSE_TO_ENDOGENOUS_STIMULUS** | | **172** | **-0.1715498** | **-0.66571933** | **0.98458374** | **1** | **1** | **3486** | **tags=23%, list=26%, signal=30%** |
| **POSITIVE_REGULATION_OF_JNK_ACTIVITY** | **POSITIVE_REGULATION_OF_JNK_ACTIVITY** | | **16** | **-0.24657506** | **-0.66570693** | **0.9006536** | **1** | **1** | **1190** | **tags=13%, list=9%, signal=14%** |
| **REGULATION_OF_GENE_EXPRESSION** | **REGULATION_OF_GENE_EXPRESSION** | | **532** | **-0.16246085** | **-0.66299325** | **0.999** | **1** | **1** | **2947** | **tags=19%, list=22%, signal=23%** |
| **NEGATIVE_REGULATION_OF_CYTOKINE_BIOSYNTHETIC_PROCESS** | **NEGATIVE_REGULATION_OF_CYTOKINE_BIOSYNTHETIC_PROCESS** | | **9** | **-0.28994295** | **-0.662612** | **0.8830986** | **0.99923277** | **1** | **1767** | **tags=22%, list=13%, signal=26%** |
| **NEGATIVE_REGULATION_OF_CELL_CYCLE** | **NEGATIVE_REGULATION_OF_CELL_CYCLE** | | **67** | **-0.18690851** | **-0.6623023** | **0.945616** | **0.99746394** | **1** | **1759** | **tags=12%, list=13%, signal=14%** |
| **REGULATION_OF_JNK_ACTIVITY** | **REGULATION_OF_JNK_ACTIVITY** | | **18** | **-0.2443328** | **-0.6614006** | **0.90231365** | **0.996137** | **1** | **3114** | **tags=22%, list=23%, signal=29%** |
| **B_CELL_DIFFERENTIATION** | **B_CELL_DIFFERENTIATION** | | **10** | **-0.2744563** | **-0.6604492** | **0.8937063** | **0.99492085** | **1** | **2843** | **tags=20%, list=21%, signal=25%** |
| **VITAMIN_METABOLIC_PROCESS** | **VITAMIN_METABOLIC_PROCESS** | | **15** | **-0.24730182** | **-0.6532498** | **0.9109042** | **0.9982369** | **1** | **1512** | **tags=13%, list=11%, signal=15%** |
| **NEGATIVE_REGULATION_OF_PROTEIN_METABOLIC_PROCESS** | **NEGATIVE_REGULATION_OF_PROTEIN_METABOLIC_PROCESS** | | **42** | **-0.19840355** | **-0.65253115** | **0.9403509** | **0.9967783** | **1** | **2249** | **tags=17%, list=17%, signal=20%** |
| **PROTEIN_AMINO_ACID_PHOSPHORYLATION** | **PROTEIN_AMINO_ACID_PHOSPHORYLATION** | | **225** | **-0.16580625** | **-0.64994985** | **0.9899092** | **0.9967187** | **1** | **3330** | **tags=23%, list=25%, signal=30%** |
| **NITROGEN_COMPOUND_METABOLIC_PROCESS** | **NITROGEN_COMPOUND_METABOLIC_PROCESS** | | **137** | **-0.16787921** | **-0.6452349** | **0.9802905** | **0.9979558** | **1** | **5191** | **tags=37%, list=39%, signal=60%** |
| **PHOSPHORYLATION** | **PHOSPHORYLATION** | | **254** | **-0.16133048** | **-0.6436762** | **0.9939394** | **0.9970398** | **1** | **3433** | **tags=23%, list=26%, signal=30%** |
| **GLUTAMINE_FAMILY_AMINO_ACID_METABOLIC_PROCESS** | **GLUTAMINE_FAMILY_AMINO_ACID_METABOLIC_PROCESS** | | **14** | **-0.24541935** | **-0.64362454** | **0.90361446** | **0.9951228** | **1** | **4434** | **tags=36%, list=33%, signal=53%** |
| **APOPTOTIC_NUCLEAR_CHANGES** | **APOPTOTIC_NUCLEAR_CHANGES** | | **16** | **-0.23622578** | **-0.64042836** | **0.9073107** | **0.99528176** | **1** | **2061** | **tags=19%, list=15%, signal=22%** |
| **MITOSIS** | **MITOSIS** |  | **66** | **-0.18258631** | **-0.6401482** | **0.958613** | **0.9935328** | **1** | **2450** | **tags=17%, list=18%, signal=20%** |
| **NEGATIVE_REGULATION_OF_INTRACELLULAR_TRANSPORT** | **NEGATIVE_REGULATION_OF_INTRACELLULAR_TRANSPORT** | | **9** | **-0.2747172** | **-0.63820034** | **0.9075862** | **0.9928836** | **1** | **257** | **tags=11%, list=2%, signal=11%** |
| **REGULATION_OF_RNA_METABOLIC_PROCESS** | **REGULATION_OF_RNA_METABOLIC_PROCESS** | | **384** | **-0.15885308** | **-0.63636833** | **0.997998** | **0.9921586** | **1** | **2947** | **tags=18%, list=22%, signal=23%** |
| **M_PHASE_OF_MITOTIC_CELL_CYCLE** | **M_PHASE_OF_MITOTIC_CELL_CYCLE** | | **68** | **-0.18082376** | **-0.6350351** | **0.9756098** | **0.9910791** | **1** | **2450** | **tags=16%, list=18%, signal=20%** |
| **ACTIN_FILAMENT_BUNDLE_FORMATION** | **ACTIN_FILAMENT_BUNDLE_FORMATION** | | **10** | **-0.26984742** | **-0.63448966** | **0.9125** | **0.98952097** | **1** | **4729** | **tags=60%, list=36%, signal=93%** |
| **REGULATION_OF_CELLULAR_METABOLIC_PROCESS** | **REGULATION_OF_CELLULAR_METABOLIC_PROCESS** | | **624** | **-0.15682508** | **-0.6321377** | **1** | **0.98913705** | **1** | **2947** | **tags=18%, list=22%, signal=22%** |
| **REGULATION_OF_TRANSCRIPTIONDNA_DEPENDENT** | **REGULATION_OF_TRANSCRIPTIONDNA_DEPENDENT** | | **379** | **-0.15810974** | **-0.6317832** | **0.998997** | **0.9874577** | **1** | **2947** | **tags=18%, list=22%, signal=23%** |
| **NEGATIVE_REGULATION_OF_TRANSPORT** | **NEGATIVE_REGULATION_OF_TRANSPORT** | | **16** | **-0.2341948** | **-0.6299641** | **0.9295039** | **0.9866214** | **1** | **4807** | **tags=44%, list=36%, signal=68%** |
| **MAINTENANCE_OF_CELLULAR_LOCALIZATION** | **MAINTENANCE_OF_CELLULAR_LOCALIZATION** | | **10** | **-0.2649506** | **-0.6279068** | **0.91725105** | **0.98595816** | **1** | **4447** | **tags=50%, list=33%, signal=75%** |
| **MAPKKK_CASCADE_GO_0000165** | **MAPKKK_CASCADE_GO_0000165** | | **90** | **-0.16912915** | **-0.62531793** | **0.9852476** | **0.98558116** | **1** | **3114** | **tags=20%, list=23%, signal=26%** |
| **PROTEIN_FOLDING** | **PROTEIN_FOLDING** | | **55** | **-0.18263501** | **-0.6196728** | **0.96914285** | **0.9869414** | **1** | **3934** | **tags=27%, list=30%, signal=39%** |
| **CELLULAR_RESPONSE_TO_STIMULUS** | **CELLULAR_RESPONSE_TO_STIMULUS** | | **15** | **-0.23414996** | **-0.614908** | **0.9270557** | **0.9877348** | **1** | **5049** | **tags=40%, list=38%, signal=64%** |
| **ACTIN_FILAMENT_ORGANIZATION** | **ACTIN_FILAMENT_ORGANIZATION** | | **20** | **-0.21584389** | **-0.6119768** | **0.9395466** | **0.9873807** | **1** | **1787** | **tags=20%, list=13%, signal=23%** |
| **ICOSANOID_METABOLIC_PROCESS** | **ICOSANOID_METABOLIC_PROCESS** | | **17** | **-0.21815273** | **-0.6084688** | **0.94473684** | **0.9873695** | **1** | **395** | **tags=6%, list=3%, signal=6%** |
| **HUMORAL_IMMUNE_RESPONSE** | **HUMORAL_IMMUNE_RESPONSE** | | **27** | **-0.20265594** | **-0.60777116** | **0.95742095** | **0.9858007** | **1** | **608** | **tags=7%, list=5%, signal=8%** |
| **DNA_CATABOLIC_PROCESS** | **DNA_CATABOLIC_PROCESS** | | **20** | **-0.21049479** | **-0.60364455** | **0.9477041** | **0.98602206** | **1** | **2228** | **tags=20%, list=17%, signal=24%** |
| **TRANSCRIPTION_FROM_RNA_POLYMERASE_III_PROMOTER** | **TRANSCRIPTION_FROM_RNA_POLYMERASE_III_PROMOTER** | | **18** | **-0.21732877** | **-0.6017616** | **0.9373368** | **0.98510164** | **1** | **4052** | **tags=28%, list=30%, signal=40%** |
| **DNA_METABOLIC_PROCESS** | **DNA_METABOLIC_PROCESS** | | **221** | **-0.15264961** | **-0.59543586** | **0.9969849** | **0.98622596** | **1** | **4603** | **tags=29%, list=35%, signal=44%** |
| **REGULATION_OF_MITOTIC_CELL_CYCLE** | **REGULATION_OF_MITOTIC_CELL_CYCLE** | | **16** | **-0.21991195** | **-0.593684** | **0.94736844** | **0.9852017** | **1** | **3509** | **tags=19%, list=26%, signal=25%** |
| **POSITIVE_REGULATION_OF_BIOLOGICAL_PROCESS** | **POSITIVE_REGULATION_OF_BIOLOGICAL_PROCESS** | | **570** | **-0.14562938** | **-0.5924996** | **1** | **0.9838786** | **1** | **2551** | **tags=15%, list=19%, signal=18%** |
| **PYRIMIDINE_NUCLEOTIDE_METABOLIC_PROCESS** | **PYRIMIDINE_NUCLEOTIDE_METABOLIC_PROCESS** | | **8** | **-0.26121792** | **-0.58873475** | **0.94** | **0.9837152** | **1** | **2507** | **tags=25%, list=19%, signal=31%** |
| **POSITIVE_REGULATION_OF_CELL_DIFFERENTIATION** | **POSITIVE_REGULATION_OF_CELL_DIFFERENTIATION** | | **19** | **-0.21145505** | **-0.5844538** | **0.9530639** | **0.98355097** | **1** | **4243** | **tags=37%, list=32%, signal=54%** |
| **POSITIVE_REGULATION_OF_CELL_PROLIFERATION** | **POSITIVE_REGULATION_OF_CELL_PROLIFERATION** | | **124** | **-0.15454078** | **-0.58419496** | **0.997921** | **0.98183835** | **1** | **3962** | **tags=25%, list=30%, signal=35%** |
| **CHROMOSOME_SEGREGATION** | **CHROMOSOME_SEGREGATION** | | **24** | **-0.1976197** | **-0.5803942** | **0.96305734** | **0.98155075** | **1** | **1616** | **tags=13%, list=12%, signal=14%** |
| **CELL_CYCLE_ARREST_GO_0007050** | **CELL_CYCLE_ARREST_GO_0007050** | | **50** | **-0.17047477** | **-0.5738436** | **0.9842697** | **0.9822286** | **1** | **3302** | **tags=18%, list=25%, signal=24%** |
| **TRANSLATION** | **TRANSLATION** | | **136** | **-0.14813575** | **-0.5641014** | **0.9958635** | **0.9837925** | **1** | **4162** | **tags=24%, list=31%, signal=35%** |
| **DNA_DEPENDENT_DNA_REPLICATION** | **DNA_DEPENDENT_DNA_REPLICATION** | | **46** | **-0.1682023** | **-0.5592702** | **0.9825986** | **0.9835383** | **1** | **4208** | **tags=30%, list=32%, signal=44%** |
| **ORGANELLE_LOCALIZATION** | **ORGANELLE_LOCALIZATION** | | **21** | **-0.19335575** | **-0.5550596** | **0.97930145** | **0.9829301** | **1** | **5408** | **tags=43%, list=41%, signal=72%** |
| **EMBRYO_IMPLANTATION** | **EMBRYO_IMPLANTATION** | | **9** | **-0.24249476** | **-0.5520519** | **0.9480519** | **0.98204166** | **1** | **5730** | **tags=56%, list=43%, signal=97%** |
| **DNA_REPLICATION_INITIATION** | **DNA_REPLICATION_INITIATION** | | **11** | **-0.22378768** | **-0.55136824** | **0.9670782** | **0.98041344** | **1** | **5265** | **tags=55%, list=40%, signal=90%** |
| **POSITIVE_REGULATION_OF_TRANSFERASE_ACTIVITY** | **POSITIVE_REGULATION_OF_TRANSFERASE_ACTIVITY** | | **68** | **-0.15512913** | **-0.54887056** | **0.99010986** | **0.97938716** | **1** | **2061** | **tags=12%, list=15%, signal=14%** |
| **REGULATION_OF_CELL_PROLIFERATION** | **REGULATION_OF_CELL_PROLIFERATION** | | **262** | **-0.1381375** | **-0.5422446** | **1** | **0.9794101** | **1** | **3962** | **tags=24%, list=30%, signal=33%** |
| **REGULATION_OF_DEFENSE_RESPONSE** | **REGULATION_OF_DEFENSE_RESPONSE** | | **14** | **-0.20534556** | **-0.5379525** | **0.97357994** | **0.9786804** | **1** | **3883** | **tags=36%, list=29%, signal=50%** |
| **NEGATIVE_REGULATION_OF_DNA_METABOLIC_PROCESS** | **NEGATIVE_REGULATION_OF_DNA_METABOLIC_PROCESS** | | **16** | **-0.20148216** | **-0.5369319** | **0.98046875** | **0.9771243** | **1** | **5182** | **tags=38%, list=39%, signal=61%** |
| **REGULATION_OF_DNA_METABOLIC_PROCESS** | **REGULATION_OF_DNA_METABOLIC_PROCESS** | | **37** | **-0.16051559** | **-0.51641494** | **0.99311924** | **0.9797789** | **1** | **4208** | **tags=24%, list=32%, signal=35%** |
| **B_CELL_ACTIVATION** | **B_CELL_ACTIVATION** | | **16** | **-0.18916085** | **-0.513297** | **0.98955613** | **0.97858375** | **1** | **3709** | **tags=25%, list=28%, signal=35%** |
| **PEROXISOME_ORGANIZATION_AND_BIOGENESIS** | **PEROXISOME_ORGANIZATION_AND_BIOGENESIS** | | **15** | **-0.18300267** | **-0.48917904** | **0.98664886** | **0.98054945** | **1** | **4257** | **tags=27%, list=32%, signal=39%** |
| **TRANSLATIONAL_INITIATION** | **TRANSLATIONAL_INITIATION** | | **22** | **-0.16760898** | **-0.48324287** | **0.99620736** | **0.9794935** | **1** | **4088** | **tags=32%, list=31%, signal=46%** |

**Table S2.5.- GSE10072: Smokers, normal, enriched in men.**

| **NAME** | **GS<br> follow link to MSigDB** | **GS DETAILS** | **SIZE** | **ES** | **NES** | **NOM p-val** | **FDR q-val** | **FWER p-val** | **RANK AT MAX** | **LEADING EDGE** |
| --- | --- | --- | --- | --- | --- | --- | --- | --- | --- | --- |
| **TRANSCRIPTION_FROM_RNA_POLYMERASE_II_PROMOTER** | **TRANSCRIPTION_FROM_RNA_POLYMERASE_II_PROMOTER** | **Details ...** | **399** | **0.40847844** | **1.9663069** | **0** | **0.13581541** | **0.114** | **3035** | **tags=35%, list=23%, signal=44%** |
| **M_PHASE** | **M_PHASE** | **Details ...** | **90** | **0.4814081** | **1.9600667** | **0** | **0.07525913** | **0.126** | **4670** | **tags=57%, list=35%, signal=87%** |
| **PROTEIN_IMPORT_INTO_NUCLEUS** | **PROTEIN_IMPORT_INTO_NUCLEUS** | **Details ...** | **42** | **0.54584736** | **1.9190984** | **0** | **0.09618801** | **0.225** | **3279** | **tags=52%, list=25%, signal=69%** |
| **NUCLEAR_IMPORT** | **NUCLEAR_IMPORT** | **Details ...** | **43** | **0.5384462** | **1.9141191** | **0** | **0.07666318** | **0.237** | **3279** | **tags=51%, list=25%, signal=68%** |
| **REGULATION_OF_CYTOKINE_PRODUCTION** | **REGULATION_OF_CYTOKINE_PRODUCTION** | **Details ...** | **20** | **0.6451546** | **1.8890572** | **0** | **0.08927776** | **0.328** | **3331** | **tags=65%, list=25%, signal=87%** |
| **PYRIMIDINE_NUCLEOTIDE_METABOLIC_PROCESS** | **PYRIMIDINE_NUCLEOTIDE_METABOLIC_PROCESS** | **Details ...** | **8** | **0.812003** | **1.8702937** | **0.00167224** | **0.09157141** | **0.385** | **1001** | **tags=50%, list=8%, signal=54%** |
| **POSITIVE_REGULATION_OF_CYTOKINE_PRODUCTION** | **POSITIVE_REGULATION_OF_CYTOKINE_PRODUCTION** | **Details ...** | **12** | **0.7136918** | **1.8483368** | **0** | **0.10676364** | **0.494** | **3331** | **tags=83%, list=25%, signal=111%** |
| **MAINTENANCE_OF_PROTEIN_LOCALIZATION** | **MAINTENANCE_OF_PROTEIN_LOCALIZATION** | **Details ...** | **10** | **0.7367237** | **1.833638** | **0.00512821** | **0.11174095** | **0.552** | **3260** | **tags=80%, list=24%, signal=106%** |
| **CELL_CYCLE_PHASE** | **CELL_CYCLE_PHASE** | **Details ...** | **140** | **0.42619523** | **1.829657** | **0** | **0.10397042** | **0.576** | **4670** | **tags=51%, list=35%, signal=78%** |
| **CELL_CYCLE_PROCESS** | **CELL_CYCLE_PROCESS** | **Details ...** | **156** | **0.42060542** | **1.8178765** | **0** | **0.10895406** | **0.64** | **4670** | **tags=53%, list=35%, signal=80%** |
| **REGULATION_OF_TRANSCRIPTION_FROM_RNA_POLYMERASE_II_PROMOTER** | **REGULATION_OF_TRANSCRIPTION_FROM_RNA_POLYMERASE_II_PROMOTER** | **Details ...** | **246** | **0.39492533** | **1.8135374** | **0** | **0.10478254** | **0.658** | **2900** | **tags=33%, list=22%, signal=42%** |
| **TRANSCRIPTION** | **TRANSCRIPTION** | **Details ...** | **627** | **0.36565557** | **1.8010579** | **0** | **0.10709395** | **0.691** | **3486** | **tags=36%, list=26%, signal=47%** |
| **MEIOTIC_CELL_CYCLE** | **MEIOTIC_CELL_CYCLE** | **Details ...** | **28** | **0.55936176** | **1.7974674** | **0.0060698** | **0.10373084** | **0.708** | **3731** | **tags=57%, list=28%, signal=79%** |
| **REGULATION_OF_JNK_CASCADE** | **REGULATION_OF_JNK_CASCADE** | **Details ...** | **11** | **0.71171576** | **1.7947232** | **0.00337838** | **0.10003042** | **0.723** | **1610** | **tags=55%, list=12%, signal=62%** |
| **TRANSCRIPTION_DNA_DEPENDENT** | **TRANSCRIPTION_DNA_DEPENDENT** | **Details ...** | **538** | **0.3616522** | **1.7847344** | **0** | **0.10571799** | **0.764** | **3486** | **tags=35%, list=26%, signal=46%** |
| **MITOSIS** | **MITOSIS** | **Details ...** | **66** | **0.46945828** | **1.7797463** | **0.0028169** | **0.10484911** | **0.779** | **4670** | **tags=56%, list=35%, signal=86%** |
| **RNA_BIOSYNTHETIC_PROCESS** | **RNA_BIOSYNTHETIC_PROCESS** | **Details ...** | **539** | **0.36080477** | **1.7713641** | **0** | **0.1092042** | **0.808** | **3486** | **tags=35%, list=26%, signal=46%** |
| **NEGATIVE_REGULATION_OF_TRANSCRIPTION** | **NEGATIVE_REGULATION_OF_TRANSCRIPTION** | **Details ...** | **148** | **0.40760782** | **1.7660615** | **0.00129366** | **0.10885008** | **0.824** | **3615** | **tags=43%, list=27%, signal=58%** |
| **PROTEIN_DNA_COMPLEX_ASSEMBLY** | **PROTEIN_DNA_COMPLEX_ASSEMBLY** | **Details ...** | **36** | **0.5272312** | **1.7606413** | **0** | **0.10913226** | **0.845** | **2542** | **tags=39%, list=19%, signal=48%** |
| **M_PHASE_OF_MITOTIC_CELL_CYCLE** | **M_PHASE_OF_MITOTIC_CELL_CYCLE** | **Details ...** | **68** | **0.4611745** | **1.7579197** | **0.00143472** | **0.10685428** | **0.849** | **4670** | **tags=54%, list=35%, signal=83%** |
| **DOUBLE_STRAND_BREAK_REPAIR** | **DOUBLE_STRAND_BREAK_REPAIR** | **Details ...** | **21** | **0.583887** | **1.7537498** | **0.00815661** | **0.10607629** | **0.858** | **1442** | **tags=43%, list=11%, signal=48%** |
| **ACTIVATION_OF_PROTEIN_KINASE_ACTIVITY** | **ACTIVATION_OF_PROTEIN_KINASE_ACTIVITY** | **Details ...** | **21** | **0.5975102** | **1.7533755** | **0.00664452** | **0.10156315** | **0.859** | **3814** | **tags=57%, list=29%, signal=80%** |
| **MEIOTIC_RECOMBINATION** | **MEIOTIC_RECOMBINATION** | **Details ...** | **14** | **0.64930737** | **1.7517844** | **0.00985222** | **0.09867442** | **0.865** | **2939** | **tags=64%, list=22%, signal=82%** |
| **ANATOMICAL_STRUCTURE_FORMATION** | **ANATOMICAL_STRUCTURE_FORMATION** | **Details ...** | **48** | **0.48601624** | **1.7509971** | **0.00304878** | **0.09559941** | **0.869** | **3592** | **tags=52%, list=27%, signal=71%** |
| **NEGATIVE_REGULATION_OF_BINDING** | **NEGATIVE_REGULATION_OF_BINDING** | **Details ...** | **15** | **0.6296716** | **1.7499335** | **0.00818331** | **0.09285642** | **0.871** | **2811** | **tags=47%, list=21%, signal=59%** |
| **NUCLEAR_TRANSPORT** | **NUCLEAR_TRANSPORT** | **Details ...** | **72** | **0.448296** | **1.7471635** | **0.00143062** | **0.09172158** | **0.878** | **2612** | **tags=38%, list=20%, signal=46%** |
| **NUCLEOCYTOPLASMIC_TRANSPORT** | **NUCLEOCYTOPLASMIC_TRANSPORT** | **Details ...** | **72** | **0.448296** | **1.7423574** | **0** | **0.09248292** | **0.891** | **2612** | **tags=38%, list=20%, signal=46%** |
| **REGULATION_OF_TRANSCRIPTION** | **REGULATION_OF_TRANSCRIPTION** | **Details ...** | **456** | **0.35819846** | **1.740854** | **0** | **0.09062655** | **0.896** | **3486** | **tags=35%, list=26%, signal=46%** |
| **NEGATIVE_REGULATION_OF_DNA_BINDING** | **NEGATIVE_REGULATION_OF_DNA_BINDING** | **Details ...** | **14** | **0.64377064** | **1.7390912** | **0.01123596** | **0.08886008** | **0.9** | **2811** | **tags=50%, list=21%, signal=63%** |
| **MAINTENANCE_OF_CELLULAR_PROTEIN_LOCALIZATION** | **MAINTENANCE_OF_CELLULAR_PROTEIN_LOCALIZATION** | **Details ...** | **9** | **0.73057264** | **1.7373261** | **0.01079137** | **0.08747393** | **0.905** | **3260** | **tags=78%, list=24%, signal=103%** |
| **TYROSINE_PHOSPHORYLATION_OF_STAT_PROTEIN** | **TYROSINE_PHOSPHORYLATION_OF_STAT_PROTEIN** | **Details ...** | **9** | **0.7230127** | **1.7371916** | **0.00340716** | **0.08476195** | **0.905** | **1246** | **tags=44%, list=9%, signal=49%** |
| **MITOTIC_CELL_CYCLE** | **MITOTIC_CELL_CYCLE** | **Details ...** | **124** | **0.4087116** | **1.7291266** | **0.00134409** | **0.08886434** | **0.922** | **4670** | **tags=51%, list=35%, signal=78%** |
| **REGULATION_OF_NUCLEOBASENUCLEOSIDENUCLEOTIDE_AND_NUCLEIC_ACID_METABOLIC_PROCESS** | **REGULATION_OF_NUCLEOBASENUCLEOSIDENUCLEOTIDE_AND_NUCLEIC_ACID_METABOLIC_PROCESS** | **Details ...** | **495** | **0.35447422** | **1.7239708** | **0** | **0.09074701** | **0.935** | **3216** | **tags=34%, list=24%, signal=43%** |
| **DNA_RECOMBINATION** | **DNA_RECOMBINATION** | **Details ...** | **41** | **0.49567226** | **1.7195302** | **0.00458716** | **0.0920551** | **0.941** | **3144** | **tags=51%, list=24%, signal=67%** |
| **PRODUCTION_OF_MOLECULAR_MEDIATOR_OF_IMMUNE_RESPONSE** | **PRODUCTION_OF_MOLECULAR_MEDIATOR_OF_IMMUNE_RESPONSE** | **Details ...** | **11** | **0.6967103** | **1.7133002** | **0.00689655** | **0.09483681** | **0.953** | **3331** | **tags=73%, list=25%, signal=97%** |
| **CYTOKINE_PRODUCTION** | **CYTOKINE_PRODUCTION** | **Details ...** | **55** | **0.46076018** | **1.7105083** | **0.00441177** | **0.09462246** | **0.959** | **3366** | **tags=42%, list=25%, signal=56%** |
| **NEGATIVE_REGULATION_OF_TRANSCRIPTION_FROM_RNA_POLYMERASE_II_PROMOTER** | **NEGATIVE_REGULATION_OF_TRANSCRIPTION_FROM_RNA_POLYMERASE_II_PROMOTER** | **Details ...** | **70** | **0.4374118** | **1.7002916** | **0.00140449** | **0.10243567** | **0.969** | **3029** | **tags=39%, list=23%, signal=50%** |
| **REGULATION_OF_MAPKKK_CASCADE** | **REGULATION_OF_MAPKKK_CASCADE** | **Details ...** | **18** | **0.5863604** | **1.693931** | **0.01658375** | **0.1060462** | **0.979** | **1610** | **tags=44%, list=12%, signal=50%** |
| **CELL_CYCLE_ARREST_GO_0007050** | **CELL_CYCLE_ARREST_GO_0007050** | **Details ...** | **50** | **0.4616164** | **1.6928141** | **0.0057554** | **0.10440412** | **0.979** | **4698** | **tags=64%, list=35%, signal=98%** |
| **PROTEIN_IMPORT** | **PROTEIN_IMPORT** | **Details ...** | **55** | **0.45929322** | **1.6880018** | **0** | **0.10640016** | **0.985** | **3279** | **tags=44%, list=25%, signal=58%** |
| **COAGULATION** | **COAGULATION** | **Details ...** | **40** | **0.48451087** | **1.6848416** | **0.00152439** | **0.1073385** | **0.99** | **3042** | **tags=43%, list=23%, signal=55%** |
| **REGULATION_OF_CELLULAR_METABOLIC_PROCESS** | **REGULATION_OF_CELLULAR_METABOLIC_PROCESS** | **Details ...** | **624** | **0.34096465** | **1.6832999** | **0** | **0.10640765** | **0.99** | **3621** | **tags=36%, list=27%, signal=47%** |
| **BLOOD_COAGULATION** | **BLOOD_COAGULATION** | **Details ...** | **40** | **0.48451087** | **1.6818551** | **0.00786164** | **0.1050212** | **0.991** | **3042** | **tags=43%, list=23%, signal=55%** |
| **REGULATION_OF_BODY_FLUID_LEVELS** | **REGULATION_OF_BODY_FLUID_LEVELS** | **Details ...** | **54** | **0.44991842** | **1.6808746** | **0** | **0.10350259** | **0.992** | **3042** | **tags=43%, list=23%, signal=55%** |
| **NEGATIVE_REGULATION_OF_METABOLIC_PROCESS** | **NEGATIVE_REGULATION_OF_METABOLIC_PROCESS** | **Details ...** | **212** | **0.36853176** | **1.6779242** | **0** | **0.10384567** | **0.992** | **3621** | **tags=40%, list=27%, signal=54%** |
| **REGULATION_OF_TRANSCRIPTIONDNA_DEPENDENT** | **REGULATION_OF_TRANSCRIPTIONDNA_DEPENDENT** | **Details ...** | **379** | **0.3505749** | **1.6777319** | **0** | **0.1018589** | **0.992** | **2934** | **tags=30%, list=22%, signal=37%** |
| **NEGATIVE_REGULATION_OF_PHOSPHATE_METABOLIC_PROCESS** | **NEGATIVE_REGULATION_OF_PHOSPHATE_METABOLIC_PROCESS** | **Details ...** | **12** | **0.6430518** | **1.6765457** | **0.01449275** | **0.10089641** | **0.992** | **1302** | **tags=42%, list=10%, signal=46%** |
| **NEGATIVE_REGULATION_OF_CELLULAR_METABOLIC_PROCESS** | **NEGATIVE_REGULATION_OF_CELLULAR_METABOLIC_PROCESS** | **Details ...** | **209** | **0.3687784** | **1.6760354** | **0.00121359** | **0.09919367** | **0.992** | **3621** | **tags=40%, list=27%, signal=54%** |
| **MEIOSIS_I** | **MEIOSIS_I** | **Details ...** | **16** | **0.6002805** | **1.6751488** | **0.01284109** | **0.09801932** | **0.993** | **3586** | **tags=63%, list=27%, signal=85%** |
| **TRANSCRIPTION_INITIATION** | **TRANSCRIPTION_INITIATION** | **Details ...** | **24** | **0.5366701** | **1.6703225** | **0.01384615** | **0.1005493** | **0.995** | **3041** | **tags=42%, list=23%, signal=54%** |
| **POSITIVE_REGULATION_OF_CELLULAR_METABOLIC_PROCESS** | **POSITIVE_REGULATION_OF_CELLULAR_METABOLIC_PROCESS** | | **177** | **0.37621772** | **1.6686366** | **0** | **0.10004426** | **0.995** | **3124** | **tags=32%, list=23%, signal=42%** |
| **REGULATION_OF_GENE_EXPRESSION** | **REGULATION_OF_GENE_EXPRESSION** | | **532** | **0.34006408** | **1.6680436** | **0** | **0.09879816** | **0.995** | **3486** | **tags=33%, list=26%, signal=44%** |
| **POSITIVE_REGULATION_OF_CATALYTIC_ACTIVITY** | **POSITIVE_REGULATION_OF_CATALYTIC_ACTIVITY** | | **135** | **0.39290628** | **1.6679053** | **0.00134409** | **0.09702002** | **0.995** | **3814** | **tags=46%, list=29%, signal=64%** |
| **RNA_METABOLIC_PROCESS** | **RNA_METABOLIC_PROCESS** | | **683** | **0.3331135** | **1.6670511** | **0** | **0.0962092** | **0.995** | **3041** | **tags=29%, list=23%, signal=36%** |
| **CASPASE_ACTIVATION** | **CASPASE_ACTIVATION** | | **24** | **0.54703873** | **1.6648941** | **0.01757189** | **0.09617004** | **0.995** | **2722** | **tags=46%, list=20%, signal=58%** |
| **REGULATION_OF_METABOLIC_PROCESS** | **REGULATION_OF_METABOLIC_PROCESS** | | **634** | **0.33639273** | **1.6642382** | **0** | **0.09493531** | **0.995** | **3621** | **tags=36%, list=27%, signal=47%** |
| **REGULATION_OF_CELL_MIGRATION** | **REGULATION_OF_CELL_MIGRATION** | | **23** | **0.54449487** | **1.6641479** | **0.00947867** | **0.09334903** | **0.995** | **4120** | **tags=61%, list=31%, signal=88%** |
| **NEGATIVE_REGULATION_OF_NUCLEOBASENUCLEOSIDENUCLEOTIDE_AND_NUCLEIC_ACID_METABOLIC_PROCESS** | **NEGATIVE_REGULATION_OF_NUCLEOBASENUCLEOSIDENUCLEOTIDE_AND_NUCLEIC_ACID_METABOLIC_PROCESS** | | **167** | **0.37748128** | **1.6638355** | **0** | **0.09209756** | **0.996** | **3615** | **tags=42%, list=27%, signal=57%** |
| **PROTEIN_KINASE_CASCADE** | **PROTEIN_KINASE_CASCADE** | | **238** | **0.36167902** | **1.662966** | **0** | **0.0910354** | **0.996** | **2583** | **tags=30%, list=19%, signal=36%** |
| **RESPONSE_TO_IONIZING_RADIATION** | **RESPONSE_TO_IONIZING_RADIATION** | | **7** | **0.7489172** | **1.65884** | **0.01043478** | **0.09263319** | **0.997** | **197** | **tags=29%, list=1%, signal=29%** |
| **POSITIVE_REGULATION_OF_METABOLIC_PROCESS** | **POSITIVE_REGULATION_OF_METABOLIC_PROCESS** | | **182** | **0.3699526** | **1.6508493** | **0** | **0.09791987** | **0.998** | **2900** | **tags=30%, list=22%, signal=38%** |
| **POSITIVE_REGULATION_OF_NUCLEOBASENUCLEOSIDENUCLEOTIDE_AND_NUCLEIC_ACID_METABOLIC_PROCESS** | **POSITIVE_REGULATION_OF_NUCLEOBASENUCLEOSIDENUCLEOTIDE_AND_NUCLEIC_ACID_METABOLIC_PROCESS** | | **118** | **0.38868213** | **1.6505308** | **0** | **0.09661702** | **0.998** | **3124** | **tags=33%, list=23%, signal=43%** |
| **NEGATIVE_REGULATION_OF_CELL_CYCLE** | **NEGATIVE_REGULATION_OF_CELL_CYCLE** | | **67** | **0.42751122** | **1.6489793** | **0.00429185** | **0.09656335** | **0.998** | **4755** | **tags=61%, list=36%, signal=95%** |
| **NEGATIVE_REGULATION_OF_TRANSCRIPTION_FACTOR_ACTIVITY** | **NEGATIVE_REGULATION_OF_TRANSCRIPTION_FACTOR_ACTIVITY** | | **12** | **0.6327871** | **1.6450081** | **0.01861252** | **0.09823854** | **0.998** | **2811** | **tags=50%, list=21%, signal=63%** |
| **POSITIVE_REGULATION_OF_CELL_ADHESION** | **POSITIVE_REGULATION_OF_CELL_ADHESION** | | **13** | **0.62692195** | **1.6432091** | **0.02** | **0.09840117** | **0.998** | **2253** | **tags=38%, list=17%, signal=46%** |
| **REGULATION_OF_RNA_METABOLIC_PROCESS** | **REGULATION_OF_RNA_METABOLIC_PROCESS** | | **384** | **0.3416296** | **1.6382815** | **0** | **0.10148624** | **0.998** | **2934** | **tags=30%, list=22%, signal=37%** |
| **CELL_SUBSTRATE_ADHESION** | **CELL_SUBSTRATE_ADHESION** | | **35** | **0.479575** | **1.6290411** | **0.01382489** | **0.10812896** | **0.999** | **4155** | **tags=49%, list=31%, signal=70%** |
| **POSITIVE_REGULATION_OF_CASPASE_ACTIVITY** | **POSITIVE_REGULATION_OF_CASPASE_ACTIVITY** | | **26** | **0.52289426** | **1.6210438** | **0.01458671** | **0.11420457** | **1** | **3805** | **tags=54%, list=29%, signal=75%** |
| **CELL_MATRIX_ADHESION** | **CELL_MATRIX_ADHESION** | | **34** | **0.48408008** | **1.6177849** | **0.00903614** | **0.11531741** | **1** | **4155** | **tags=50%, list=31%, signal=72%** |
| **POSITIVE_REGULATION_OF_TRANSCRIPTION** | **POSITIVE_REGULATION_OF_TRANSCRIPTION** | | **109** | **0.38578022** | **1.6159085** | **0** | **0.11522075** | **1** | **3124** | **tags=33%, list=23%, signal=43%** |
| **NEGATIVE_REGULATION_OF_PHOSPHORYLATION** | **NEGATIVE_REGULATION_OF_PHOSPHORYLATION** | | **11** | **0.6477604** | **1.6147419** | **0.01785714** | **0.1144956** | **1** | **1302** | **tags=45%, list=10%, signal=50%** |
| **NEGATIVE_REGULATION_OF_RNA_METABOLIC_PROCESS** | **NEGATIVE_REGULATION_OF_RNA_METABOLIC_PROCESS** | | **103** | **0.38583374** | **1.6114694** | **0.00134228** | **0.11623888** | **1** | **3615** | **tags=40%, list=27%, signal=54%** |
| **ANGIOGENESIS** | **ANGIOGENESIS** | | **40** | **0.45923084** | **1.6104473** | **0.01040119** | **0.11582311** | **1** | **3588** | **tags=50%, list=27%, signal=68%** |
| **NEGATIVE_REGULATION_OF_SIGNAL_TRANSDUCTION** | **NEGATIVE_REGULATION_OF_SIGNAL_TRANSDUCTION** | | **30** | **0.48243076** | **1.6042693** | **0.02140673** | **0.12046491** | **1** | **3311** | **tags=43%, list=25%, signal=58%** |
| **WOUND_HEALING** | **WOUND_HEALING** | | **48** | **0.4476089** | **1.6018487** | **0.01017442** | **0.12096017** | **1** | **3124** | **tags=40%, list=23%, signal=52%** |
| **HEMOSTASIS** | **HEMOSTASIS** | | **45** | **0.45217237** | **1.5991616** | **0.00847458** | **0.1221158** | **1** | **3042** | **tags=42%, list=23%, signal=55%** |
| **CHROMOSOME_ORGANIZATION_AND_BIOGENESIS** | **CHROMOSOME_ORGANIZATION_AND_BIOGENESIS** | | **97** | **0.389417** | **1.594255** | **0.00273224** | **0.12557852** | **1** | **3665** | **tags=39%, list=28%, signal=54%** |
| **ADENYLATE_CYCLASE_ACTIVATION** | **ADENYLATE_CYCLASE_ACTIVATION** | | **19** | **0.54805654** | **1.5936301** | **0.02487562** | **0.12459236** | **1** | **1822** | **tags=47%, list=14%, signal=55%** |
| **REGULATION_OF_MOLECULAR_FUNCTION** | **REGULATION_OF_MOLECULAR_FUNCTION** | | **265** | **0.3416226** | **1.5906266** | **0** | **0.12652968** | **1** | **3962** | **tags=42%, list=30%, signal=59%** |
| **NEGATIVE_REGULATION_OF_TRANSCRIPTION_DNA_DEPENDENT** | **NEGATIVE_REGULATION_OF_TRANSCRIPTION_DNA_DEPENDENT** | | **103** | **0.38583374** | **1.5887253** | **0.00405954** | **0.126955** | **1** | **3615** | **tags=40%, list=27%, signal=54%** |
| **REGULATION_OF_MITOSIS** | **REGULATION_OF_MITOSIS** | | **34** | **0.47279036** | **1.5882334** | **0.01854714** | **0.12591743** | **1** | **4670** | **tags=59%, list=35%, signal=90%** |
| **REGULATION_OF_CATALYTIC_ACTIVITY** | **REGULATION_OF_CATALYTIC_ACTIVITY** | | **231** | **0.34721974** | **1.5879933** | **0.00122399** | **0.12463068** | **1** | **3962** | **tags=44%, list=30%, signal=62%** |
| **RESPONSE_TO_HYPOXIA** | **RESPONSE_TO_HYPOXIA** | | **25** | **0.5131977** | **1.584642** | **0.02898551** | **0.12653624** | **1** | **3236** | **tags=40%, list=24%, signal=53%** |
| **EMBRYONIC_MORPHOGENESIS** | **EMBRYONIC_MORPHOGENESIS** | | **14** | **0.5913371** | **1.5824065** | **0.03355705** | **0.12738957** | **1** | **3597** | **tags=50%, list=27%, signal=68%** |
| **REGULATION_OF_PROTEIN_STABILITY** | **REGULATION_OF_PROTEIN_STABILITY** | | **14** | **0.58601284** | **1.5748367** | **0.03624382** | **0.13360043** | **1** | **2620** | **tags=43%, list=20%, signal=53%** |
| **CELL_CYCLE_GO_0007049** | **CELL_CYCLE_GO_0007049** | | **260** | **0.34021017** | **1.5693902** | **0.00121951** | **0.13827124** | **1** | **4755** | **tags=48%, list=36%, signal=74%** |
| **VASCULATURE_DEVELOPMENT** | **VASCULATURE_DEVELOPMENT** | | **46** | **0.43468702** | **1.5689211** | **0.01497006** | **0.13715062** | **1** | **3807** | **tags=48%, list=29%, signal=67%** |
| **NUCLEOTIDE_BIOSYNTHETIC_PROCESS** | **NUCLEOTIDE_BIOSYNTHETIC_PROCESS** | | **16** | **0.5639401** | **1.5656868** | **0.02741936** | **0.1392412** | **1** | **3138** | **tags=56%, list=24%, signal=73%** |
| **NEGATIVE_REGULATION_OF_CELLULAR_PROCESS** | **NEGATIVE_REGULATION_OF_CELLULAR_PROCESS** | | **538** | **0.3210202** | **1.5650064** | **0** | **0.13849421** | **1** | **3621** | **tags=35%, list=27%, signal=47%** |
| **NEGATIVE_REGULATION_OF_SECRETION** | **NEGATIVE_REGULATION_OF_SECRETION** | | **9** | **0.6532666** | **1.5562944** | **0.04280822** | **0.14662321** | **1** | **3518** | **tags=56%, list=26%, signal=75%** |
| **MITOTIC_SPINDLE_ORGANIZATION_AND_BIOGENESIS** | **MITOTIC_SPINDLE_ORGANIZATION_AND_BIOGENESIS** | | **9** | **0.6636464** | **1.5479426** | **0.03793104** | **0.1553336** | **1** | **2703** | **tags=67%, list=20%, signal=84%** |
| **NEGATIVE_REGULATION_OF_BIOLOGICAL_PROCESS** | **NEGATIVE_REGULATION_OF_BIOLOGICAL_PROCESS** | | **563** | **0.31521076** | **1.5457423** | **0** | **0.15618956** | **1** | **4147** | **tags=40%, list=31%, signal=56%** |
| **INTRACELLULAR_SIGNALING_CASCADE** | **INTRACELLULAR_SIGNALING_CASCADE** | | **555** | **0.31399104** | **1.5447708** | **0** | **0.15554963** | **1** | **2909** | **tags=30%, list=22%, signal=37%** |
| **G_PROTEIN_SIGNALING_ADENYLATE_CYCLASE_ACTIVATING_PATHWAY** | **G_PROTEIN_SIGNALING_ADENYLATE_CYCLASE_ACTIVATING_PATHWAY** | | **25** | **0.49377978** | **1.5441953** | **0.03733766** | **0.15464185** | **1** | **2977** | **tags=52%, list=22%, signal=67%** |
| **NEGATIVE_REGULATION_OF_MYELOID_CELL_DIFFERENTIATION** | **NEGATIVE_REGULATION_OF_MYELOID_CELL_DIFFERENTIATION** | | **10** | **0.6215169** | **1.5402867** | **0.04821429** | **0.15769228** | **1** | **2895** | **tags=40%, list=22%, signal=51%** |
| **TRANSCRIPTION_INITIATION_FROM_RNA_POLYMERASE_II_PROMOTER** | **TRANSCRIPTION_INITIATION_FROM_RNA_POLYMERASE_II_PROMOTER** | | **18** | **0.54005945** | **1.5337874** | **0.04139073** | **0.16373523** | **1** | **2542** | **tags=39%, list=19%, signal=48%** |
| **INSULIN_RECEPTOR_SIGNALING_PATHWAY** | **INSULIN_RECEPTOR_SIGNALING_PATHWAY** | | **16** | **0.5462267** | **1.5334309** | **0.04792332** | **0.16251162** | **1** | **1950** | **tags=31%, list=15%, signal=37%** |
| **POSITIVE_REGULATION_OF_CYTOKINE_BIOSYNTHETIC_PROCESS** | **POSITIVE_REGULATION_OF_CYTOKINE_BIOSYNTHETIC_PROCESS** | | **21** | **0.5061237** | **1.5254197** | **0.04597701** | **0.1705637** | **1** | **2253** | **tags=24%, list=17%, signal=29%** |
| **SKELETAL_DEVELOPMENT** | **SKELETAL_DEVELOPMENT** | | **91** | **0.37826952** | **1.5240057** | **0.00958904** | **0.17060958** | **1** | **3487** | **tags=40%, list=26%, signal=53%** |
| **REGULATION_OF_HORMONE_SECRETION** | **REGULATION_OF_HORMONE_SECRETION** | | **12** | **0.5848169** | **1.5230812** | **0.03846154** | **0.17007393** | **1** | **4147** | **tags=58%, list=31%, signal=85%** |
| **REGULATION_OF_PROTEIN_POLYMERIZATION** | **REGULATION_OF_PROTEIN_POLYMERIZATION** | | **10** | **0.6194251** | **1.5186492** | **0.05820106** | **0.17414396** | **1** | **541** | **tags=30%, list=4%, signal=31%** |
| **MUSCLE_DEVELOPMENT** | **MUSCLE_DEVELOPMENT** | | **85** | **0.377633** | **1.5163816** | **0.00827586** | **0.17534503** | **1** | **2586** | **tags=34%, list=19%, signal=42%** |
| **REGULATION_OF_IMMUNE_EFFECTOR_PROCESS** | **REGULATION_OF_IMMUNE_EFFECTOR_PROCESS** | | **13** | **0.57063335** | **1.5149622** | **0.04545455** | **0.17538014** | **1** | **3331** | **tags=77%, list=25%, signal=102%** |
| **NEGATIVE_REGULATION_OF_INTRACELLULAR_TRANSPORT** | **NEGATIVE_REGULATION_OF_INTRACELLULAR_TRANSPORT** | | **9** | **0.62741804** | **1.5141509** | **0.05181347** | **0.17497091** | **1** | **3260** | **tags=67%, list=24%, signal=88%** |
| **REGULATION_OF_CELL_CYCLE** | **REGULATION_OF_CELL_CYCLE** | | **153** | **0.34426773** | **1.5136225** | **0.00383142** | **0.1739625** | **1** | **4789** | **tags=52%, list=36%, signal=81%** |
| **NEGATIVE_REGULATION_OF_TRANSPORT** | **NEGATIVE_REGULATION_OF_TRANSPORT** | | **16** | **0.55011725** | **1.512753** | **0.04950495** | **0.1731949** | **1** | **3621** | **tags=56%, list=27%, signal=77%** |
| **PROTEIN_LOCALIZATION** | **PROTEIN_LOCALIZATION** | | **174** | **0.3410519** | **1.5095222** | **0.00127551** | **0.17552057** | **1** | **2720** | **tags=32%, list=20%, signal=39%** |
| **RESPONSE_TO_NUTRIENT** | **RESPONSE_TO_NUTRIENT** | | **16** | **0.52391964** | **1.5081447** | **0.04530744** | **0.1758126** | **1** | **3861** | **tags=69%, list=29%, signal=97%** |
| **NLS_BEARING_SUBSTRATE_IMPORT_INTO_NUCLEUS** | **NLS_BEARING_SUBSTRATE_IMPORT_INTO_NUCLEUS** | | **11** | **0.6037489** | **1.5078983** | **0.0464345** | **0.17454301** | **1** | **2323** | **tags=55%, list=17%, signal=66%** |
| **POSITIVE_REGULATION_OF_BIOLOGICAL_PROCESS** | **POSITIVE_REGULATION_OF_BIOLOGICAL_PROCESS** | | **570** | **0.3050495** | **1.507246** | **0** | **0.17383863** | **1** | **3805** | **tags=35%, list=29%, signal=47%** |
| **HORMONE_SECRETION** | **HORMONE_SECRETION** | | **14** | **0.55485386** | **1.50719** | **0.04297521** | **0.17238401** | **1** | **5257** | **tags=71%, list=39%, signal=118%** |
| **MICROTUBULE_CYTOSKELETON_ORGANIZATION_AND_BIOGENESIS** | **MICROTUBULE_CYTOSKELETON_ORGANIZATION_AND_BIOGENESIS** | | **31** | **0.45555094** | **1.5059035** | **0.04468413** | **0.17238735** | **1** | **2703** | **tags=35%, list=20%, signal=44%** |
| **REGULATION_OF_TYROSINE_PHOSPHORYLATION_OF_STAT_PROTEIN** | **REGULATION_OF_TYROSINE_PHOSPHORYLATION_OF_STAT_PROTEIN** | | **6** | **0.721701** | **1.4986664** | **0.02871622** | **0.18067355** | **1** | **1246** | **tags=50%, list=9%, signal=55%** |
| **DNA_REPAIR** | **DNA_REPAIR** | | **115** | **0.35314992** | **1.4968046** | **0.01494565** | **0.18121088** | **1** | **2972** | **tags=33%, list=22%, signal=42%** |
| **REGULATION_OF_NUCLEOCYTOPLASMIC_TRANSPORT** | **REGULATION_OF_NUCLEOCYTOPLASMIC_TRANSPORT** | | **18** | **0.5210113** | **1.4960511** | **0.0477707** | **0.18065049** | **1** | **5016** | **tags=72%, list=38%, signal=116%** |
| **POSITIVE_REGULATION_OF_TRANSCRIPTIONDNA_DEPENDENT** | **POSITIVE_REGULATION_OF_TRANSCRIPTIONDNA_DEPENDENT** | | **93** | **0.36287293** | **1.4951439** | **0.01751825** | **0.18043567** | **1** | **2900** | **tags=30%, list=22%, signal=38%** |
| **POSITIVE_REGULATION_OF_DNA_METABOLIC_PROCESS** | **POSITIVE_REGULATION_OF_DNA_METABOLIC_PROCESS** | | **10** | **0.59917414** | **1.493134** | **0.04537522** | **0.18137594** | **1** | **4632** | **tags=60%, list=35%, signal=92%** |
| **REGULATION_OF_TRANSFERASE_ACTIVITY** | **REGULATION_OF_TRANSFERASE_ACTIVITY** | | **133** | **0.3457089** | **1.4923592** | **0.00933333** | **0.18066695** | **1** | **3948** | **tags=44%, list=30%, signal=61%** |
| **MACROMOLECULE_LOCALIZATION** | **MACROMOLECULE_LOCALIZATION** | | **190** | **0.33532336** | **1.4893963** | **0.0036855** | **0.18300714** | **1** | **2720** | **tags=31%, list=20%, signal=38%** |
| **PROTEIN_IMPORT_INTO_NUCLEUS_TRANSLOCATION** | **PROTEIN_IMPORT_INTO_NUCLEUS_TRANSLOCATION** | | **11** | **0.59019953** | **1.4886785** | **0.05369128** | **0.18242745** | **1** | **1552** | **tags=36%, list=12%, signal=41%** |
| **NUCLEOSOME_ASSEMBLY** | **NUCLEOSOME_ASSEMBLY** | | **10** | **0.61659795** | **1.4871337** | **0.04950495** | **0.18313225** | **1** | **1787** | **tags=40%, list=13%, signal=46%** |
| **ESTABLISHMENT_OF_PROTEIN_LOCALIZATION** | **ESTABLISHMENT_OF_PROTEIN_LOCALIZATION** | | **156** | **0.33951905** | **1.4856699** | **0.00898588** | **0.18347642** | **1** | **2509** | **tags=29%, list=19%, signal=36%** |
| **JAK_STAT_CASCADE** | **JAK_STAT_CASCADE** | | **26** | **0.4791627** | **1.4855684** | **0.0512** | **0.18212202** | **1** | **2528** | **tags=35%, list=19%, signal=43%** |
| **DNA_METABOLIC_PROCESS** | **DNA_METABOLIC_PROCESS** | | **221** | **0.32568148** | **1.4854296** | **0.00732601** | **0.18081887** | **1** | **3588** | **tags=37%, list=27%, signal=49%** |
| **GLAND_DEVELOPMENT** | **GLAND_DEVELOPMENT** | | **12** | **0.56983745** | **1.4852746** | **0.05374593** | **0.17959891** | **1** | **5016** | **tags=58%, list=38%, signal=93%** |
| **PROTEIN_MODIFICATION_BY_SMALL_PROTEIN_CONJUGATION** | **PROTEIN_MODIFICATION_BY_SMALL_PROTEIN_CONJUGATION** | | **36** | **0.43586832** | **1.4847064** | **0.03633491** | **0.17875548** | **1** | **3144** | **tags=44%, list=24%, signal=58%** |
| **POSITIVE_REGULATION_OF_ANGIOGENESIS** | **POSITIVE_REGULATION_OF_ANGIOGENESIS** | | **9** | **0.624315** | **1.4829868** | **0.05405406** | **0.17935926** | **1** | **3504** | **tags=67%, list=26%, signal=90%** |
| **MUSCLE_CELL_DIFFERENTIATION** | **MUSCLE_CELL_DIFFERENTIATION** | | **19** | **0.5050096** | **1.4804784** | **0.04501608** | **0.18175161** | **1** | **2260** | **tags=32%, list=17%, signal=38%** |
| **POSITIVE_REGULATION_OF_TRANSCRIPTION_FROM_RNA_POLYMERASE_II_PROMOTER** | **POSITIVE_REGULATION_OF_TRANSCRIPTION_FROM_RNA_POLYMERASE_II_PROMOTER** | | **53** | **0.40507796** | **1.4800187** | **0.03264095** | **0.18098368** | **1** | **2900** | **tags=30%, list=22%, signal=38%** |
| **DEVELOPMENTAL_MATURATION** | **DEVELOPMENTAL_MATURATION** | | **17** | **0.50434464** | **1.4796848** | **0.07487521** | **0.18004194** | **1** | **2260** | **tags=35%, list=17%, signal=42%** |
| **REGULATION_OF_KINASE_ACTIVITY** | **REGULATION_OF_KINASE_ACTIVITY** | | **131** | **0.3446845** | **1.4794674** | **0.00909091** | **0.17896198** | **1** | **3948** | **tags=44%, list=30%, signal=61%** |
| **CALCIUM_MEDIATED_SIGNALING** | **CALCIUM_MEDIATED_SIGNALING** | | **13** | **0.56714725** | **1.4756869** | **0.04085258** | **0.18215255** | **1** | **593** | **tags=23%, list=4%, signal=24%** |
| **PROTEIN_UBIQUITINATION** | **PROTEIN_UBIQUITINATION** | | **34** | **0.43280137** | **1.4734929** | **0.04573171** | **0.18383731** | **1** | **3144** | **tags=44%, list=24%, signal=58%** |
| **GENERATION_OF_A_SIGNAL_INVOLVED_IN_CELL_CELL_SIGNALING** | **GENERATION_OF_A_SIGNAL_INVOLVED_IN_CELL_CELL_SIGNALING** | | **24** | **0.48257402** | **1.4727782** | **0.05057096** | **0.18347289** | **1** | **4321** | **tags=58%, list=32%, signal=86%** |
| **POSITIVE_REGULATION_OF_CELLULAR_PROCESS** | **POSITIVE_REGULATION_OF_CELLULAR_PROCESS** | | **540** | **0.29910582** | **1.4717534** | **0** | **0.18334848** | **1** | **3805** | **tags=34%, list=29%, signal=46%** |
| **REGULATION_OF_SECRETION** | **REGULATION_OF_SECRETION** | | **29** | **0.445897** | **1.4711441** | **0.04307692** | **0.18281567** | **1** | **3518** | **tags=48%, list=26%, signal=65%** |
| **REGULATION_OF_PROTEIN_KINASE_ACTIVITY** | **REGULATION_OF_PROTEIN_KINASE_ACTIVITY** | | **130** | **0.34239554** | **1.4707515** | **0.00780234** | **0.18198507** | **1** | **3948** | **tags=43%, list=30%, signal=61%** |
| **INTERFERON_GAMMA_PRODUCTION** | **INTERFERON_GAMMA_PRODUCTION** | | **11** | **0.58623254** | **1.4701921** | **0.05901639** | **0.18135944** | **1** | **2253** | **tags=45%, list=17%, signal=55%** |
| **POSITIVE_REGULATION_OF_DEVELOPMENTAL_PROCESS** | **POSITIVE_REGULATION_OF_DEVELOPMENTAL_PROCESS** | | **186** | **0.33169007** | **1.4696132** | **0.00250313** | **0.18085411** | **1** | **4286** | **tags=44%, list=32%, signal=63%** |
| **MITOTIC_CELL_CYCLE_CHECKPOINT** | **MITOTIC_CELL_CYCLE_CHECKPOINT** | | **19** | **0.5108522** | **1.4686892** | **0.05638474** | **0.18070768** | **1** | **887** | **tags=26%, list=7%, signal=28%** |
| **REGULATION_OF_PROTEIN_IMPORT_INTO_NUCLEUS** | **REGULATION_OF_PROTEIN_IMPORT_INTO_NUCLEUS** | | **13** | **0.5575833** | **1.4667279** | **0.05685619** | **0.18166272** | **1** | **3260** | **tags=54%, list=24%, signal=71%** |
| **REGULATION_OF_MYELOID_CELL_DIFFERENTIATION** | **REGULATION_OF_MYELOID_CELL_DIFFERENTIATION** | | **18** | **0.502851** | **1.4665754** | **0.06420546** | **0.18060821** | **1** | **2895** | **tags=39%, list=22%, signal=50%** |
| **CHROMOSOME_CONDENSATION** | **CHROMOSOME_CONDENSATION** | | **7** | **0.6711522** | **1.4659959** | **0.06514084** | **0.17997873** | **1** | **2860** | **tags=71%, list=21%, signal=91%** |
| **REGULATION_OF_INTRACELLULAR_TRANSPORT** | **REGULATION_OF_INTRACELLULAR_TRANSPORT** | | **21** | **0.4912792** | **1.4655503** | **0.0485133** | **0.17934315** | **1** | **3260** | **tags=48%, list=24%, signal=63%** |
| **INTRACELLULAR_PROTEIN_TRANSPORT** | **INTRACELLULAR_PROTEIN_TRANSPORT** | | **122** | **0.3459977** | **1.464842** | **0.01184211** | **0.17907894** | **1** | **2720** | **tags=30%, list=20%, signal=38%** |
| **CYTOKINE_METABOLIC_PROCESS** | **CYTOKINE_METABOLIC_PROCESS** | | **34** | **0.4296632** | **1.4646628** | **0.03448276** | **0.17807814** | **1** | **1506** | **tags=21%, list=11%, signal=23%** |
| **NEGATIVE_REGULATION_OF_CELL_MIGRATION** | **NEGATIVE_REGULATION_OF_CELL_MIGRATION** | | **14** | **0.5432897** | **1.4541696** | **0.05806452** | **0.18998404** | **1** | **3124** | **tags=50%, list=23%, signal=65%** |
| **MACROMOLECULAR_COMPLEX_ASSEMBLY** | **MACROMOLECULAR_COMPLEX_ASSEMBLY** | | **219** | **0.32060805** | **1.4492096** | **0.00615006** | **0.19510399** | **1** | **2612** | **tags=28%, list=20%, signal=34%** |
| **CELLULAR_COMPONENT_ASSEMBLY** | **CELLULAR_COMPONENT_ASSEMBLY** | | **236** | **0.3165498** | **1.4490424** | **0.00476191** | **0.19405338** | **1** | **2612** | **tags=28%, list=20%, signal=34%** |
| **POSITIVE_REGULATION_OF_CELL_MIGRATION** | **POSITIVE_REGULATION_OF_CELL_MIGRATION** | | **8** | **0.631297** | **1.446132** | **0.09074733** | **0.19626762** | **1** | **4120** | **tags=75%, list=31%, signal=109%** |
| **REGULATION_OF_CELL_CELL_ADHESION** | **REGULATION_OF_CELL_CELL_ADHESION** | | **9** | **0.61081994** | **1.4404963** | **0.08035714** | **0.20243277** | **1** | **3807** | **tags=67%, list=29%, signal=93%** |
| **MAPKKK_CASCADE_GO_0000165** | **MAPKKK_CASCADE_GO_0000165** | | **90** | **0.3562476** | **1.4388921** | **0.01392758** | **0.20315838** | **1** | **2583** | **tags=32%, list=19%, signal=40%** |
| **POSITIVE_REGULATION_OF_RNA_METABOLIC_PROCESS** | **POSITIVE_REGULATION_OF_RNA_METABOLIC_PROCESS** | | **95** | **0.35469663** | **1.4375935** | **0.02319236** | **0.20360962** | **1** | **2900** | **tags=29%, list=22%, signal=37%** |
| **REGULATION_OF_RAS_PROTEIN_SIGNAL_TRANSDUCTION** | **REGULATION_OF_RAS_PROTEIN_SIGNAL_TRANSDUCTION** | | **11** | **0.5737565** | **1.4364079** | **0.07804878** | **0.20380013** | **1** | **2328** | **tags=36%, list=17%, signal=44%** |
| **DNA_PACKAGING** | **DNA_PACKAGING** | | **27** | **0.45660114** | **1.4343346** | **0.0659824** | **0.20545456** | **1** | **3214** | **tags=41%, list=24%, signal=54%** |
| **CELLULAR_PROTEIN_CATABOLIC_PROCESS** | **CELLULAR_PROTEIN_CATABOLIC_PROCESS** | | **49** | **0.39622188** | **1.4337696** | **0.04239766** | **0.20497641** | **1** | **2223** | **tags=29%, list=17%, signal=34%** |
| **REGULATION_OF_CELL_ADHESION** | **REGULATION_OF_CELL_ADHESION** | | **32** | **0.43327928** | **1.4315696** | **0.05384615** | **0.20643142** | **1** | **4120** | **tags=44%, list=31%, signal=63%** |
| **PROTEIN_TRANSPORT** | **PROTEIN_TRANSPORT** | | **134** | **0.33364832** | **1.4303253** | **0.01546392** | **0.20675676** | **1** | **2612** | **tags=29%, list=20%, signal=36%** |
| **CHROMATIN_ASSEMBLY_OR_DISASSEMBLY** | **CHROMATIN_ASSEMBLY_OR_DISASSEMBLY** | | **25** | **0.45828274** | **1.4292288** | **0.06858055** | **0.20705837** | **1** | **3351** | **tags=40%, list=25%, signal=53%** |
| **VIRAL_GENOME_REPLICATION** | **VIRAL_GENOME_REPLICATION** | | **20** | **0.4789968** | **1.4244182** | **0.08510638** | **0.21237336** | **1** | **2902** | **tags=55%, list=22%, signal=70%** |
| **RESPONSE_TO_DNA_DAMAGE_STIMULUS** | **RESPONSE_TO_DNA_DAMAGE_STIMULUS** | | **144** | **0.33178326** | **1.4242853** | **0.00794702** | **0.21125074** | **1** | **3184** | **tags=33%, list=24%, signal=42%** |
| **MAINTENANCE_OF_CELLULAR_LOCALIZATION** | **MAINTENANCE_OF_CELLULAR_LOCALIZATION** | | **10** | **0.58412** | **1.4232752** | **0.09317803** | **0.21138176** | **1** | **3260** | **tags=70%, list=24%, signal=93%** |
| **REGULATION_OF_TRANSPORT** | **REGULATION_OF_TRANSPORT** | | **55** | **0.38621262** | **1.4207852** | **0.05393586** | **0.21350454** | **1** | **3621** | **tags=44%, list=27%, signal=60%** |
| **ESTABLISHMENT_AND_OR_MAINTENANCE_OF_CHROMATIN_ARCHITECTURE** | **ESTABLISHMENT_AND_OR_MAINTENANCE_OF_CHROMATIN_ARCHITECTURE** | | **60** | **0.3837918** | **1.4206917** | **0.03399434** | **0.21236165** | **1** | **3351** | **tags=35%, list=25%, signal=47%** |
| **REGULATION_OF_IMMUNE_RESPONSE** | **REGULATION_OF_IMMUNE_RESPONSE** | | **26** | **0.45113716** | **1.4202163** | **0.06677266** | **0.21162346** | **1** | **3621** | **tags=54%, list=27%, signal=74%** |
| **AGING** | **AGING** |  | **11** | **0.5719728** | **1.4153478** | **0.08376069** | **0.21725133** | **1** | **1061** | **tags=27%, list=8%, signal=30%** |
| **I_KAPPAB_KINASE_NF_KAPPAB_CASCADE** | **I_KAPPAB_KINASE_NF_KAPPAB_CASCADE** | | **88** | **0.3492849** | **1.4144802** | **0.033241** | **0.21712594** | **1** | **3799** | **tags=38%, list=29%, signal=52%** |
| **REGULATION_OF_DNA_METABOLIC_PROCESS** | **REGULATION_OF_DNA_METABOLIC_PROCESS** | | **37** | **0.4095362** | **1.4143264** | **0.07033639** | **0.21605727** | **1** | **2232** | **tags=38%, list=17%, signal=45%** |
| **PEPTIDYL_AMINO_ACID_MODIFICATION** | **PEPTIDYL_AMINO_ACID_MODIFICATION** | | **46** | **0.39397243** | **1.411653** | **0.05805515** | **0.2184035** | **1** | **1657** | **tags=24%, list=12%, signal=27%** |
| **REGULATION_OF_BINDING** | **REGULATION_OF_BINDING** | | **42** | **0.39827406** | **1.4098347** | **0.06550218** | **0.21965481** | **1** | **2977** | **tags=33%, list=22%, signal=43%** |
| **POSITIVE_REGULATION_OF_TRANSLATION** | **POSITIVE_REGULATION_OF_TRANSLATION** | | **28** | **0.4448245** | **1.4073919** | **0.0741325** | **0.22162129** | **1** | **3366** | **tags=29%, list=25%, signal=38%** |
| **REGULATION_OF_IMMUNE_SYSTEM_PROCESS** | **REGULATION_OF_IMMUNE_SYSTEM_PROCESS** | | **55** | **0.3836245** | **1.4064184** | **0.04558405** | **0.22160126** | **1** | **3621** | **tags=44%, list=27%, signal=60%** |
| **CELL_CYCLE_CHECKPOINT_GO_0000075** | **CELL_CYCLE_CHECKPOINT_GO_0000075** | | **42** | **0.39709142** | **1.4055483** | **0.06748466** | **0.22145016** | **1** | **2137** | **tags=31%, list=16%, signal=37%** |
| **SPINDLE_ORGANIZATION_AND_BIOGENESIS** | **SPINDLE_ORGANIZATION_AND_BIOGENESIS** | | **10** | **0.5723853** | **1.4053063** | **0.10797342** | **0.22047794** | **1** | **2703** | **tags=60%, list=20%, signal=75%** |
| **PEPTIDYL_TYROSINE_PHOSPHORYLATION** | **PEPTIDYL_TYROSINE_PHOSPHORYLATION** | | **21** | **0.47229373** | **1.4021761** | **0.07443366** | **0.22351219** | **1** | **1627** | **tags=29%, list=12%, signal=32%** |
| **EMBRYONIC_DEVELOPMENT** | **EMBRYONIC_DEVELOPMENT** | | **46** | **0.39357376** | **1.3979778** | **0.07424243** | **0.22789946** | **1** | **2661** | **tags=39%, list=20%, signal=49%** |
| **REGULATION_OF_CELL_SHAPE** | **REGULATION_OF_CELL_SHAPE** | | **8** | **0.6165171** | **1.3975453** | **0.09847198** | **0.22732173** | **1** | **4383** | **tags=75%, list=33%, signal=112%** |
| **INTERPHASE** | **INTERPHASE** | | **58** | **0.3677692** | **1.3967996** | **0.0605613** | **0.22705868** | **1** | **4749** | **tags=48%, list=36%, signal=75%** |
| **NEGATIVE_REGULATION_OF_CELL_PROLIFERATION** | **NEGATIVE_REGULATION_OF_CELL_PROLIFERATION** | | **138** | **0.32228813** | **1.3961877** | **0.02213542** | **0.22661784** | **1** | **3674** | **tags=38%, list=28%, signal=51%** |
| **PROTEIN_POLYMERIZATION** | **PROTEIN_POLYMERIZATION** | | **16** | **0.500216** | **1.3955089** | **0.09836066** | **0.2262379** | **1** | **541** | **tags=19%, list=4%, signal=20%** |
| **POSITIVE_REGULATION_OF_HYDROLASE_ACTIVITY** | **POSITIVE_REGULATION_OF_HYDROLASE_ACTIVITY** | | **44** | **0.398382** | **1.395412** | **0.06508876** | **0.22513184** | **1** | **3805** | **tags=48%, list=29%, signal=67%** |
| **STRESS_ACTIVATED_PROTEIN_KINASE_SIGNALING_PATHWAY** | **STRESS_ACTIVATED_PROTEIN_KINASE_SIGNALING_PATHWAY** | | **45** | **0.3923482** | **1.3948115** | **0.06807867** | **0.22479697** | **1** | **2439** | **tags=36%, list=18%, signal=43%** |
| **POSITIVE_REGULATION_OF_IMMUNE_RESPONSE** | **POSITIVE_REGULATION_OF_IMMUNE_RESPONSE** | | **22** | **0.4530895** | **1.3937707** | **0.09379968** | **0.22495966** | **1** | **3621** | **tags=59%, list=27%, signal=81%** |
| **REGULATION_OF_CYTOKINE_BIOSYNTHETIC_PROCESS** | **REGULATION_OF_CYTOKINE_BIOSYNTHETIC_PROCESS** | | **31** | **0.42161804** | **1.3925829** | **0.08153846** | **0.22537851** | **1** | **1506** | **tags=19%, list=11%, signal=22%** |
| **ENDOTHELIAL_CELL_PROLIFERATION** | **ENDOTHELIAL_CELL_PROLIFERATION** | | **9** | **0.59601676** | **1.391784** | **0.10286678** | **0.22517478** | **1** | **896** | **tags=33%, list=7%, signal=36%** |
| **REGULATION_OF_TRANSCRIPTION_FACTOR_ACTIVITY** | **REGULATION_OF_TRANSCRIPTION_FACTOR_ACTIVITY** | | **27** | **0.43549508** | **1.3892782** | **0.09060956** | **0.22779244** | **1** | **2087** | **tags=30%, list=16%, signal=35%** |
| **HOMOPHILIC_CELL_ADHESION** | **HOMOPHILIC_CELL_ADHESION** | | **12** | **0.5411076** | **1.3888816** | **0.09390445** | **0.2270717** | **1** | **3557** | **tags=50%, list=27%, signal=68%** |
| **REGULATION_OF_INTERFERON_GAMMA_BIOSYNTHETIC_PROCESS** | **REGULATION_OF_INTERFERON_GAMMA_BIOSYNTHETIC_PROCESS** | | **8** | **0.599814** | **1.3877416** | **0.091354** | **0.22728604** | **1** | **2253** | **tags=38%, list=17%, signal=45%** |
| **PROTEIN_TARGETING** | **PROTEIN_TARGETING** | | **92** | **0.34345564** | **1.3814691** | **0.04011065** | **0.23528337** | **1** | **2720** | **tags=33%, list=20%, signal=41%** |
| **CELL_FATE_COMMITMENT** | **CELL_FATE_COMMITMENT** | | **13** | **0.5280378** | **1.3806766** | **0.1095008** | **0.2351644** | **1** | **3592** | **tags=46%, list=27%, signal=63%** |
| **POSITIVE_REGULATION_OF_IMMUNE_SYSTEM_PROCESS** | **POSITIVE_REGULATION_OF_IMMUNE_SYSTEM_PROCESS** | | **41** | **0.39260647** | **1.3793932** | **0.07969925** | **0.23571049** | **1** | **3621** | **tags=44%, list=27%, signal=60%** |
| **HEART_DEVELOPMENT** | **HEART_DEVELOPMENT** | | **32** | **0.4209026** | **1.3790582** | **0.06687403** | **0.23494487** | **1** | **2773** | **tags=28%, list=21%, signal=35%** |
| **REGULATION_OF_RESPONSE_TO_STIMULUS** | **REGULATION_OF_RESPONSE_TO_STIMULUS** | | **47** | **0.38580358** | **1.3782955** | **0.06746627** | **0.23475374** | **1** | **3621** | **tags=45%, list=27%, signal=61%** |
| **PROTEIN_CATABOLIC_PROCESS** | **PROTEIN_CATABOLIC_PROCESS** | | **58** | **0.37155506** | **1.3766271** | **0.05665723** | **0.23589353** | **1** | **2223** | **tags=28%, list=17%, signal=33%** |
| **MITOTIC_SISTER_CHROMATID_SEGREGATION** | **MITOTIC_SISTER_CHROMATID_SEGREGATION** | | **12** | **0.52424246** | **1.369535** | **0.09417808** | **0.24523133** | **1** | **1845** | **tags=33%, list=14%, signal=39%** |
| **POSITIVE_REGULATION_OF_TRANSFERASE_ACTIVITY** | **POSITIVE_REGULATION_OF_TRANSFERASE_ACTIVITY** | | **68** | **0.35572076** | **1.3673539** | **0.04864091** | **0.24729927** | **1** | **3928** | **tags=46%, list=29%, signal=64%** |
| **REGULATION_OF_HYDROLASE_ACTIVITY** | **REGULATION_OF_HYDROLASE_ACTIVITY** | | **63** | **0.361739** | **1.3671612** | **0.05772006** | **0.24633694** | **1** | **4041** | **tags=49%, list=30%, signal=70%** |
| **CELL_MIGRATION** | **CELL_MIGRATION** | | **82** | **0.34619415** | **1.3663491** | **0.04081633** | **0.24625883** | **1** | **3608** | **tags=37%, list=27%, signal=50%** |
| **DNA_REPLICATION** | **DNA_REPLICATION** | | **85** | **0.34336925** | **1.3647368** | **0.05020353** | **0.24721609** | **1** | **3393** | **tags=36%, list=25%, signal=49%** |
| **PROTEIN_COMPLEX_ASSEMBLY** | **PROTEIN_COMPLEX_ASSEMBLY** | | **149** | **0.3111928** | **1.3643668** | **0.03355705** | **0.24648854** | **1** | **2862** | **tags=30%, list=21%, signal=37%** |
| **FOCAL_ADHESION_FORMATION** | **FOCAL_ADHESION_FORMATION** | | **10** | **0.55520135** | **1.363947** | **0.10915493** | **0.2459011** | **1** | **4155** | **tags=60%, list=31%, signal=87%** |
| **POSITIVE_REGULATION_OF_LYMPHOCYTE_ACTIVATION** | **POSITIVE_REGULATION_OF_LYMPHOCYTE_ACTIVATION** | | **22** | **0.44909903** | **1.3559102** | **0.09677419** | **0.25643033** | **1** | **2815** | **tags=36%, list=21%, signal=46%** |
| **ORGANELLE_ORGANIZATION_AND_BIOGENESIS** | **ORGANELLE_ORGANIZATION_AND_BIOGENESIS** | | **386** | **0.2799319** | **1.3541247** | **0.00227273** | **0.25794548** | **1** | **3749** | **tags=35%, list=28%, signal=48%** |
| **G_PROTEIN_SIGNALING_COUPLED_TO_CAMP_NUCLEOTIDE_SECOND_MESSENGER** | **G_PROTEIN_SIGNALING_COUPLED_TO_CAMP_NUCLEOTIDE_SECOND_MESSENGER** | | **63** | **0.3553802** | **1.3536184** | **0.07344633** | **0.2575127** | **1** | **3985** | **tags=51%, list=30%, signal=72%** |
| **RNA_SPLICINGVIA_TRANSESTERIFICATION_REACTIONS** | **RNA_SPLICINGVIA_TRANSESTERIFICATION_REACTIONS** | | **17** | **0.47914645** | **1.3532858** | **0.11707317** | **0.256692** | **1** | **1550** | **tags=24%, list=12%, signal=27%** |
| **CELL_DIVISION** | **CELL_DIVISION** | | **18** | **0.46897057** | **1.3528525** | **0.11428572** | **0.25618416** | **1** | **4021** | **tags=67%, list=30%, signal=95%** |
| **CELL_MATURATION** | **CELL_MATURATION** | | **15** | **0.4927655** | **1.3525534** | **0.11980033** | **0.2554502** | **1** | **2260** | **tags=33%, list=17%, signal=40%** |
| **POSITIVE_REGULATION_OF_CELLULAR_PROTEIN_METABOLIC_PROCESS** | **POSITIVE_REGULATION_OF_CELLULAR_PROTEIN_METABOLIC_PROCESS** | | **59** | **0.35763454** | **1.3494625** | **0.08115942** | **0.25906104** | **1** | **1627** | **tags=20%, list=12%, signal=23%** |
| **POSITIVE_REGULATION_OF_EPITHELIAL_CELL_PROLIFERATION** | **POSITIVE_REGULATION_OF_EPITHELIAL_CELL_PROLIFERATION** | | **7** | **0.6170993** | **1.3476949** | **0.14031972** | **0.26061317** | **1** | **593** | **tags=29%, list=4%, signal=30%** |
| **REGULATION_OF_RHO_PROTEIN_SIGNAL_TRANSDUCTION** | **REGULATION_OF_RHO_PROTEIN_SIGNAL_TRANSDUCTION** | | **7** | **0.60502523** | **1.3467511** | **0.13356164** | **0.26094308** | **1** | **1454** | **tags=29%, list=11%, signal=32%** |
| **REGULATION_OF_PROGRAMMED_CELL_DEATH** | **REGULATION_OF_PROGRAMMED_CELL_DEATH** | | **296** | **0.28444916** | **1.34584** | **0.01542112** | **0.26109818** | **1** | **4286** | **tags=40%, list=32%, signal=58%** |
| **REGULATION_OF_DNA_BINDING** | **REGULATION_OF_DNA_BINDING** | | **34** | **0.39933926** | **1.3454088** | **0.09785933** | **0.26050827** | **1** | **2811** | **tags=29%, list=21%, signal=37%** |
| **REGULATION_OF_MITOTIC_CELL_CYCLE** | **REGULATION_OF_MITOTIC_CELL_CYCLE** | | **16** | **0.4830804** | **1.3443912** | **0.12106136** | **0.2608747** | **1** | **4651** | **tags=75%, list=35%, signal=115%** |
| **REGULATION_OF_CELL_PROLIFERATION** | **REGULATION_OF_CELL_PROLIFERATION** | | **262** | **0.28863195** | **1.3424045** | **0.01794258** | **0.2627289** | **1** | **3618** | **tags=33%, list=27%, signal=45%** |
| **POSITIVE_REGULATION_OF_MULTICELLULAR_ORGANISMAL_PROCESS** | **POSITIVE_REGULATION_OF_MULTICELLULAR_ORGANISMAL_PROCESS** | | **52** | **0.3656286** | **1.3423339** | **0.08154506** | **0.2616124** | **1** | **3621** | **tags=44%, list=27%, signal=61%** |
| **UBIQUITIN_CYCLE** | **UBIQUITIN_CYCLE** | | **42** | **0.3883554** | **1.3374462** | **0.09552239** | **0.26777765** | **1** | **3144** | **tags=40%, list=24%, signal=53%** |
| **INTERLEUKIN_2_PRODUCTION** | **INTERLEUKIN_2_PRODUCTION** | | **10** | **0.54258406** | **1.3373533** | **0.14527027** | **0.26671514** | **1** | **1429** | **tags=40%, list=11%, signal=45%** |
| **NEGATIVE_REGULATION_OF_CELL_ADHESION** | **NEGATIVE_REGULATION_OF_CELL_ADHESION** | | **16** | **0.48251155** | **1.3371686** | **0.15806451** | **0.2657471** | **1** | **4120** | **tags=50%, list=31%, signal=72%** |
| **REGULATION_OF_APOPTOSIS** | **REGULATION_OF_APOPTOSIS** | | **295** | **0.2859734** | **1.3327789** | **0.01543943** | **0.27127773** | **1** | **4286** | **tags=40%, list=32%, signal=58%** |
| **REGULATION_OF_PHOSPHORYLATION** | **REGULATION_OF_PHOSPHORYLATION** | | **38** | **0.39297235** | **1.3312039** | **0.1051051** | **0.2723831** | **1** | **1627** | **tags=26%, list=12%, signal=30%** |
| **APOPTOTIC_PROGRAM** | **APOPTOTIC_PROGRAM** | | **53** | **0.36060217** | **1.3303959** | **0.10298508** | **0.27241933** | **1** | **2860** | **tags=34%, list=21%, signal=43%** |
| **RNA_SPLICING** | **RNA_SPLICING** | | **59** | **0.35131314** | **1.3298447** | **0.08671328** | **0.272141** | **1** | **1621** | **tags=20%, list=12%, signal=23%** |
| **BASE_EXCISION_REPAIR** | **BASE_EXCISION_REPAIR** | | **15** | **0.48518908** | **1.3265591** | **0.12106136** | **0.27604988** | **1** | **237** | **tags=13%, list=2%, signal=14%** |
| **CYTOKINE_BIOSYNTHETIC_PROCESS** | **CYTOKINE_BIOSYNTHETIC_PROCESS** | | **33** | **0.39436412** | **1.324768** | **0.10575428** | **0.27745563** | **1** | **1506** | **tags=18%, list=11%, signal=20%** |
| **EXTRACELLULAR_STRUCTURE_ORGANIZATION_AND_BIOGENESIS** | **EXTRACELLULAR_STRUCTURE_ORGANIZATION_AND_BIOGENESIS** | | **22** | **0.44094652** | **1.3244035** | **0.11809816** | **0.2768314** | **1** | **2550** | **tags=41%, list=19%, signal=51%** |
| **CELLULAR_LOCALIZATION** | **CELLULAR_LOCALIZATION** | | **310** | **0.28175345** | **1.323976** | **0.0150289** | **0.27621683** | **1** | **3331** | **tags=33%, list=25%, signal=42%** |
| **CAMP_MEDIATED_SIGNALING** | **CAMP_MEDIATED_SIGNALING** | | **64** | **0.35137114** | **1.3237861** | **0.0944206** | **0.27532506** | **1** | **3985** | **tags=50%, list=30%, signal=71%** |
| **POSITIVE_REGULATION_OF_PROTEIN_METABOLIC_PROCESS** | **POSITIVE_REGULATION_OF_PROTEIN_METABOLIC_PROCESS** | | **61** | **0.35086554** | **1.3230413** | **0.0911641** | **0.27535525** | **1** | **3366** | **tags=31%, list=25%, signal=41%** |
| **REGULATION_OF_JAK_STAT_CASCADE** | **REGULATION_OF_JAK_STAT_CASCADE** | | **7** | **0.5992538** | **1.322722** | **0.13240418** | **0.27460343** | **1** | **1246** | **tags=43%, list=9%, signal=47%** |
| **PROTEIN_AUTOPROCESSING** | **PROTEIN_AUTOPROCESSING** | | **24** | **0.42103866** | **1.3212489** | **0.11864407** | **0.27580753** | **1** | **2591** | **tags=29%, list=19%, signal=36%** |
| **JNK_CASCADE** | **JNK_CASCADE** | | **44** | **0.37437612** | **1.3212395** | **0.09715994** | **0.27462354** | **1** | **2439** | **tags=34%, list=18%, signal=42%** |
| **SMOOTH_MUSCLE_CONTRACTION_GO_0006939** | **SMOOTH_MUSCLE_CONTRACTION_GO_0006939** | | **11** | **0.5127421** | **1.3184963** | **0.14853196** | **0.27775517** | **1** | **2855** | **tags=45%, list=21%, signal=58%** |
| **NEGATIVE_REGULATION_OF_TRANSFERASE_ACTIVITY** | **NEGATIVE_REGULATION_OF_TRANSFERASE_ACTIVITY** | | **28** | **0.41555846** | **1.3184963** | **0.12426036** | **0.27656308** | **1** | **3184** | **tags=46%, list=24%, signal=61%** |
| **REGULATION_OF_DEVELOPMENTAL_PROCESS** | **REGULATION_OF_DEVELOPMENTAL_PROCESS** | | **369** | **0.27500224** | **1.318203** | **0.02508552** | **0.2758366** | **1** | **4302** | **tags=40%, list=32%, signal=57%** |
| **MESODERM_DEVELOPMENT** | **MESODERM_DEVELOPMENT** | | **22** | **0.43293616** | **1.3177007** | **0.1312** | **0.27540588** | **1** | **2875** | **tags=41%, list=22%, signal=52%** |
| **CYTOKINESIS** | **CYTOKINESIS** | | **16** | **0.4690985** | **1.316296** | **0.13333334** | **0.2763071** | **1** | **4021** | **tags=69%, list=30%, signal=98%** |
| **DNA_DAMAGE_RESPONSESIGNAL_TRANSDUCTION_RESULTING_IN_INDUCTION_OF_APOPTOSIS** | **DNA_DAMAGE_RESPONSESIGNAL_TRANSDUCTION_RESULTING_IN_INDUCTION_OF_APOPTOSIS** | | **13** | **0.4948653** | **1.3149198** | **0.13752122** | **0.27715135** | **1** | **4242** | **tags=46%, list=32%, signal=68%** |
| **ORGAN_MORPHOGENESIS** | **ORGAN_MORPHOGENESIS** | | **125** | **0.30942094** | **1.3120854** | **0.05997393** | **0.28044972** | **1** | **3162** | **tags=32%, list=24%, signal=42%** |
| **CELL_DEVELOPMENT** | **CELL_DEVELOPMENT** | | **498** | **0.26804516** | **1.3116623** | **0.01314348** | **0.27992377** | **1** | **3064** | **tags=28%, list=23%, signal=34%** |
| **REGULATION_OF_SIGNAL_TRANSDUCTION** | **REGULATION_OF_SIGNAL_TRANSDUCTION** | | **168** | **0.29787764** | **1.3097787** | **0.05006418** | **0.281552** | **1** | **3432** | **tags=33%, list=26%, signal=44%** |
| **LOCOMOTORY_BEHAVIOR** | **LOCOMOTORY_BEHAVIOR** | | **83** | **0.32039216** | **1.3089641** | **0.08743169** | **0.28165007** | **1** | **2810** | **tags=34%, list=21%, signal=42%** |
| **POSITIVE_REGULATION_OF_RESPONSE_TO_STIMULUS** | **POSITIVE_REGULATION_OF_RESPONSE_TO_STIMULUS** | | **33** | **0.38758907** | **1.308308** | **0.11975116** | **0.28158617** | **1** | **3621** | **tags=45%, list=27%, signal=62%** |
| **ORGAN_DEVELOPMENT** | **ORGAN_DEVELOPMENT** | | **493** | **0.26839304** | **1.3068478** | **0.00892857** | **0.28274342** | **1** | **3176** | **tags=29%, list=24%, signal=36%** |
[truncated: 2,212,029 more chars]
