# Supplementary material for: Repeated observation of immune gene sets enrichment in women with non-small cell lung cancer
Source: Oncotarget. 2016 Mar 6;7(15):20282–92. doi: 10.18632/oncotarget.7943 (PMC4991454; doi:10.18632/oncotarget.7943)
Supplement: Supplementary file 3 [file oncotarget-07-20282-s003.docx]

**TABLE S3.- Overrepresented genes related to immune system in all cohorts.**

| **DATASET** | **PROCESS** | **PROBE** | **RANK IN GENE LIST** | **RANK METRIC SCORE** | **RUNNING ES** | **TISSUE** | **STATUS** |
| --- | --- | --- | --- | --- | --- | --- | --- |
| GSE32863 | IMMUNE SYSTEM PROCESS | ACIN1 | 33849 | -0,36956501 | -0,26515216 | TUMOR | Non-Smokers |
| TCGA | IMMUNE SYSTEM PROCESS | ACIN1 | 17096 | -0,25255001 | -0,2557419 | TUMOR | Non-Smokers |
| GSE47115 | IMMUNE SYSTEM PROCESS | ACVR1B | 18676 | -0,36612934 | -0,31805354 | TUMOR | Smokers |
| TCGA | IMMUNE SYSTEM PROCESS | ACVR1B | 18301 | -0,31521824 | -0,16190161 | TUMOR | Non-Smokers |
| TCGA | DEFENSE RESPONSE | ADORA1 | 18891 | -0,10869214 | -0,18644162 | TUMOR | Smokers |
| GSE50081 | CELLULAR DEFENSE RESPONSE | ADORA2A | 18115 | -0,1337069 | -0,31048676 | TUMOR | Smokers |
| GSE47115 | CELLULAR DEFENSE RESPONSE | ADORA2A | 17215 | -0,27998641 | -0,3324168 | TUMOR | Smokers |
| TCGA | CELLULAR DEFENSE RESPONSE | ADORA2A | 15186 | -0,05347211 | -0,3661511 | TUMOR | Smokers |
| GSE50081 | DEFENSE RESPONSE | ADORA2A | 18115 | -0,1337069 | -0,38053384 | TUMOR | Smokers |
| GSE47115 | DEFENSE RESPONSE | ADORA2A | 17215 | -0,27998641 | -0,27429864 | TUMOR | Smokers |
| TCGA | DEFENSE RESPONSE | ADORA2A | 15186 | -0,05347211 | -0,40609413 | TUMOR | Smokers |
| GSE47115 | CELLULAR DEFENSE RESPONSE | ADORA2B | 19988 | -0,51102728 | -0,1391357 | TUMOR | Smokers |
| TCGA | CELLULAR DEFENSE RESPONSE | ADORA2B | 17595 | -0,08406039 | -0,22516167 | TUMOR | Smokers |
| GSE47115 | DEFENSE RESPONSE | ADORA2B | 19988 | -0,51102728 | -0,13015977 | TUMOR | Smokers |
| GSE32863 | DEFENSE RESPONSE | ADORA2B | 29674 | -0,20454824 | -0,40655 | TUMOR | Smokers |
| TCGA | DEFENSE RESPONSE | ADORA2B | 17595 | -0,08406039 | -0,32413965 | TUMOR | Smokers |
| GSE50081 | DEFENSE RESPONSE | ADORA3 | 18674 | -0,15177247 | -0,33355743 | TUMOR | Smokers |
| GSE47115 | DEFENSE RESPONSE | ADORA3 | 17069 | -0,27161032 | -0,29645172 | TUMOR | Smokers |
| GSE32863 | DEFENSE RESPONSE | AFAP1L2 | 36317 | -0,60610408 | -0,0103585 | TUMOR | Non-Smokers |
| GSE32863 | DEFENSE RESPONSE | AFAP1L2 | 35423 | -0,42714548 | -0,23153912 | TUMOR | Smokers |
| TCGA | DEFENSE RESPONSE | AFAP1L2 | 18279 | -0,09569972 | -0,24419725 | TUMOR | Smokers |
| GSE50081 | DEFENSE RESPONSE | AGER | 18085 | -0,13262138 | -0,3894172 | TUMOR | Smokers |
| GSE32863 | DEFENSE RESPONSE | AGER | 31010 | -0,23695116 | -0,38422802 | TUMOR | Smokers |
| GSE50081 | DEFENSE RESPONSE | AHSG | 18364 | -0,14169036 | -0,38726112 | TUMOR | Smokers |
| GSE47115 | DEFENSE RESPONSE | AHSG | 16810 | -0,2587603 | -0,32164454 | TUMOR | Smokers |
| GSE32863 | DEFENSE RESPONSE | AHSG | 30598 | -0,22694153 | -0,39592254 | TUMOR | Smokers |
| GSE47115 | DEFENSE RESPONSE | AIF1 | 15938 | -0,21970488 | -0,33900124 | TUMOR | Smokers |
| GSE32863 | DEFENSE RESPONSE | AIF1 | 29555 | -0,20171337 | -0,4066842 | TUMOR | Smokers |
| TCGA | DEFENSE RESPONSE | AIF1 | 14861 | -0,0502104 | -0,41565585 | TUMOR | Smokers |
| GSE50081 | IMMUNE_RESPONSE | AIM2 | 18660 | -0,15154423 | -0,31449166 | TUMOR | Smokers |
| GSE47115 | IMMUNE_RESPONSE | AIM2 | 20345 | -0,6018095 | -0,16877832 | TUMOR | Smokers |
| GSE32863 | IMMUNE_RESPONSE | AIM2 | 35361 | -0,42275453 | -0,22450857 | TUMOR | Smokers |
| TCGA | IMMUNE_RESPONSE | AIM2 | 19130 | -0,11486514 | -0,16199678 | TUMOR | Smokers |
| GSE50081 | IMMUNE SYSTEM PROCESS | AIM2 | 18660 | -0,15154423 | -0,27862576 | TUMOR | Smokers |
| GSE47115 | IMMUNE SYSTEM PROCESS | AIM2 | 20345 | -0,6018095 | -0,16786128 | TUMOR | Smokers |
| GSE32863 | IMMUNE SYSTEM PROCESS | AIM2 | 35361 | -0,42275453 | -0,19143966 | TUMOR | Smokers |
| TCGA | IMMUNE SYSTEM PROCESS | AIM2 | 19130 | -0,11486514 | -0,14470409 | TUMOR | Smokers |
| TCGA | IMMUNE SYSTEM PROCESS | AKT1 | 16470 | -0,22898208 | -0,30833146 | TUMOR | Non-Smokers |
| GSE32863 | IMMUNE SYSTEM PROCESS | ALAS2 | 32623 | -0,28294176 | -0,36367586 | TUMOR | Smokers |
| TCGA | IMMUNE SYSTEM PROCESS | ALAS2 | 19088 | -0,11366497 | -0,14805894 | TUMOR | Smokers |
| TCGA | IMMUNE SYSTEM PROCESS | ALAS2 | 19523 | -0,44210884 | -0,0791163 | TUMOR | Non-Smokers |
| GSE47115 | DEFENSE RESPONSE | ALOX15 | 15438 | -0,19813862 | -0,35131067 | TUMOR | Smokers |
| GSE32863 | DEFENSE RESPONSE | ALOX15 | 30849 | -0,23292507 | -0,38374388 | TUMOR | Smokers |
| TCGA | DEFENSE RESPONSE | ALOX15 | 16652 | -0,07080727 | -0,36228505 | TUMOR | Smokers |
| GSE32863 | DEFENSE RESPONSE | ALOX5AP | 30343 | -0,24640626 | -0,3139138 | TUMOR | Non-Smokers |
| GSE32863 | DEFENSE RESPONSE | ALOX5AP | 33933 | -0,33524925 | -0,3105356 | TUMOR | Smokers |
| TCGA | DEFENSE RESPONSE | ALOX5AP | 18438 | -0,09883274 | -0,228299 | TUMOR | Smokers |
| GSE50081 | DEFENSE RESPONSE | ANKRD1 | 20205 | -0,2436029 | -0,16892678 | TUMOR | Smokers |
| GSE32863 | DEFENSE RESPONSE | ANKRD1 | 35876 | -0,51916224 | -0,055780075 | TUMOR | Non-Smokers |
| TCGA | DEFENSE RESPONSE | ANKRD1 | 18156 | -0,09339245 | -0,25539142 | TUMOR | Smokers |
| TCGA | DEFENSE RESPONSE | ANXA1 | 18342 | -0,09688476 | -0,23554282 | TUMOR | Smokers |
| GSE32863 | IMMUNE_RESPONSE | ANXA11 | 35473 | -0,47592863 | -0,18823439 | TUMOR | Non-Smokers |
| GSE32863 | IMMUNE SYSTEM PROCESS | ANXA11 | 35473 | -0,47592863 | -0,18325005 | TUMOR | Non-Smokers |
| GSE50081 | DEFENSE RESPONSE | AOAH | 19735 | -0,2022198 | -0,25711542 | TUMOR | Smokers |
| GSE47115 | DEFENSE RESPONSE | AOAH | 14929 | -0,17724663 | -0,34961584 | TUMOR | Smokers |
| GSE32863 | DEFENSE RESPONSE | AOAH | 29154 | -0,21484084 | -0,34617823 | TUMOR | Non-Smokers |
| GSE32863 | DEFENSE RESPONSE | AOAH | 34376 | -0,35625476 | -0,28249994 | TUMOR | Smokers |
| TCGA | DEFENSE RESPONSE | AOC3 | 17995 | -0,09058013 | -0,2643218 | TUMOR | Smokers |
| GSE32863 | DEFENSE RESPONSE | AOX1 | 30753 | -0,25846976 | -0,29895347 | TUMOR | Non-Smokers |
| GSE50081 | DEFENSE RESPONSE | APCS | 19587 | -0,19289148 | -0,28041738 | TUMOR | Smokers |
| GSE47115 | DEFENSE RESPONSE | APCS | 17205 | -0,27952141 | -0,27875593 | TUMOR | Smokers |
| GSE32863 | DEFENSE RESPONSE | APCS | 31446 | -0,24892336 | -0,37587258 | TUMOR | Smokers |
| GSE32863 | IMMUNE_RESPONSE | APOA1 | 30510 | -0,25137258 | -0,32729155 | TUMOR | Non-Smokers |
| GSE32863 | IMMUNE_RESPONSE | APOA1 | 30571 | -0,22635446 | -0,3996013 | TUMOR | Smokers |
| TCGA | IMMUNE_RESPONSE | APOA1 | 18184 | -0,30923137 | -0,16000444 | TUMOR | Non-Smokers |
| GSE32863 | IMMUNE SYSTEM PROCESS | APOA1 | 30510 | -0,25137258 | -0,34544367 | TUMOR | Non-Smokers |
| GSE32863 | IMMUNE SYSTEM PROCESS | APOA1 | 30571 | -0,22635446 | -0,36833155 | TUMOR | Smokers |
| TCGA | IMMUNE SYSTEM PROCESS | APOA1 | 18184 | -0,30923137 | -0,18321544 | TUMOR | Non-Smokers |
| GSE32863 | IMMUNE_RESPONSE | APOA2 | 30974 | -0,26444259 | -0,32074478 | TUMOR | Non-Smokers |
| GSE32863 | IMMUNE_RESPONSE | APOA2 | 32626 | -0,28302121 | -0,3934199 | TUMOR | Smokers |
| TCGA | IMMUNE_RESPONSE | APOA2 | 14872 | -0,17723504 | -0,3752714 | TUMOR | Non-Smokers |
| GSE32863 | IMMUNE SYSTEM PROCESS | APOA2 | 30974 | -0,26444259 | -0,3413419 | TUMOR | Non-Smokers |
| GSE32863 | IMMUNE SYSTEM PROCESS | APOA2 | 32626 | -0,28302121 | -0,35979956 | TUMOR | Smokers |
| TCGA | IMMUNE SYSTEM PROCESS | APOA2 | 14872 | -0,17723504 | -0,36643803 | TUMOR | Non-Smokers |
| GSE32863 | IMMUNE_RESPONSE | APOA4 | 29301 | -0,19637291 | -0,42442608 | TUMOR | Smokers |
| TCGA | IMMUNE_RESPONSE | APOA4 | 19836 | -0,57658923 | -0,06884918 | TUMOR | Non-Smokers |
| GSE32863 | IMMUNE SYSTEM PROCESS | APOA4 | 29301 | -0,19637291 | -0,3990853 | TUMOR | Smokers |
| TCGA | IMMUNE SYSTEM PROCESS | APOA4 | 19836 | -0,57658923 | -0,058337618 | TUMOR | Non-Smokers |
| GSE32863 | DEFENSE RESPONSE | APOA4 | 29301 | -0,19637291 | -0,41291347 | TUMOR | Smokers |
| GSE50081 | IMMUNE_RESPONSE | APOBEC3F | 16283 | -0,08945549 | -0,4063929 | TUMOR | Smokers |
| GSE47115 | IMMUNE_RESPONSE | APOBEC3F | 18232 | -0,33590406 | -0,33886245 | TUMOR | Smokers |
| GSE32863 | IMMUNE_RESPONSE | APOBEC3F | 30981 | -0,26467851 | -0,31597012 | TUMOR | Non-Smokers |
| GSE32863 | IMMUNE_RESPONSE | APOBEC3F | 35439 | -0,42885038 | -0,19460046 | TUMOR | Smokers |
| TCGA | IMMUNE_RESPONSE | APOBEC3F | 19226 | -0,11740696 | -0,113670945 | TUMOR | Smokers |
| TCGA | IMMUNE_RESPONSE | APOBEC3F | 18409 | -0,32302028 | -0,1319578 | TUMOR | Non-Smokers |
| GSE50081 | IMMUNE SYSTEM PROCESS | APOBEC3F | 16283 | -0,08945549 | -0,3556892 | TUMOR | Smokers |
| GSE47115 | IMMUNE SYSTEM PROCESS | APOBEC3F | 18232 | -0,33590406 | -0,33246407 | TUMOR | Smokers |
| GSE32863 | IMMUNE SYSTEM PROCESS | APOBEC3F | 30981 | -0,26467851 | -0,33805504 | TUMOR | Non-Smokers |
| GSE32863 | IMMUNE SYSTEM PROCESS | APOBEC3F | 35439 | -0,42885038 | -0,16978644 | TUMOR | Smokers |
| TCGA | IMMUNE SYSTEM PROCESS | APOBEC3F | 19226 | -0,11740696 | -0,10537686 | TUMOR | Smokers |
| TCGA | IMMUNE SYSTEM PROCESS | APOBEC3F | 18409 | -0,32302028 | -0,15062395 | TUMOR | Non-Smokers |
| GSE47115 | DEFENSE RESPONSE | APOBEC3F | 18232 | -0,33590406 | -0,2481819 | TUMOR | Smokers |
| GSE32863 | DEFENSE RESPONSE | APOBEC3F | 30981 | -0,26467851 | -0,3006498 | TUMOR | Non-Smokers |
| GSE32863 | DEFENSE RESPONSE | APOBEC3F | 35439 | -0,42885038 | -0,22488007 | TUMOR | Smokers |
| TCGA | DEFENSE RESPONSE | APOBEC3F | 19226 | -0,11740696 | -0,17548646 | TUMOR | Smokers |
| GSE47115 | REGULATION OF IMMUNE SYSTEM PROCESS | APOBEC3F | 18232 | -0,33590406 | -0,3306666 | TUMOR | Smokers |
| GSE32863 | REGULATION OF IMMUNE SYSTEM PROCESS | APOBEC3F | 35439 | -0,42885038 | -0,21000494 | TUMOR | Smokers |
| TCGA | REGULATION OF IMMUNE SYSTEM PROCESS | APOBEC3F | 18409 | -0,32302028 | -0,1556984 | TUMOR | Non-Smokers |
| GSE50081 | IMMUNE_RESPONSE | APOBEC3G | 18838 | -0,15702735 | -0,27757844 | TUMOR | Smokers |
| GSE47115 | IMMUNE_RESPONSE | APOBEC3G | 20354 | -0,60454279 | -0,14777336 | TUMOR | Smokers |
| GSE32863 | IMMUNE_RESPONSE | APOBEC3G | 32705 | -0,32262936 | -0,28544447 | TUMOR | Non-Smokers |
| GSE32863 | IMMUNE_RESPONSE | APOBEC3G | 36040 | -0,49948832 | -0,15822105 | TUMOR | Smokers |
| TCGA | IMMUNE_RESPONSE | APOBEC3G | 18947 | -0,11002023 | -0,1894815 | TUMOR | Smokers |
| TCGA | IMMUNE_RESPONSE | APOBEC3G | 18499 | -0,32988682 | -0,12831996 | TUMOR | Non-Smokers |
| GSE50081 | IMMUNE SYSTEM PROCESS | APOBEC3G | 18838 | -0,15702735 | -0,24883683 | TUMOR | Smokers |
| GSE47115 | IMMUNE SYSTEM PROCESS | APOBEC3G | 20354 | -0,60454279 | -0,15290733 | TUMOR | Smokers |
| GSE32863 | IMMUNE SYSTEM PROCESS | APOBEC3G | 32705 | -0,32262936 | -0,28866026 | TUMOR | Non-Smokers |
| GSE32863 | IMMUNE SYSTEM PROCESS | APOBEC3G | 36040 | -0,49948832 | -0,12742029 | TUMOR | Smokers |
| TCGA | IMMUNE SYSTEM PROCESS | APOBEC3G | 18947 | -0,11002023 | -0,16759071 | TUMOR | Smokers |
| TCGA | IMMUNE SYSTEM PROCESS | APOBEC3G | 18499 | -0,32988682 | -0,14947434 | TUMOR | Non-Smokers |
| GSE50081 | DEFENSE RESPONSE | APOBEC3G | 18838 | -0,15702735 | -0,3053895 | TUMOR | Smokers |
| GSE47115 | DEFENSE RESPONSE | APOBEC3G | 20354 | -0,60454279 | -0,069551595 | TUMOR | Smokers |
| GSE32863 | DEFENSE RESPONSE | APOBEC3G | 32705 | -0,32262936 | -0,27213702 | TUMOR | Non-Smokers |
| GSE32863 | DEFENSE RESPONSE | APOBEC3G | 36040 | -0,49948832 | -0,14335862 | TUMOR | Smokers |
| TCGA | DEFENSE RESPONSE | APOBEC3G | 18947 | -0,11002023 | -0,18253152 | TUMOR | Smokers |
| GSE47115 | REGULATION OF IMMUNE SYSTEM PROCESS | APOBEC3G | 20354 | -0,60454279 | -0,094348505 | TUMOR | Smokers |
| GSE10072 | REGULATION OF IMMUNE SYSTEM PROCESS | APOBEC3G | 12564 | -0,50817913 | -0,17500457 | NORMAL | Non-Smokers |
| GSE32863 | REGULATION OF IMMUNE SYSTEM PROCESS | APOBEC3G | 36040 | -0,49948832 | -0,166243 | TUMOR | Smokers |
| TCGA | REGULATION OF IMMUNE SYSTEM PROCESS | APOBEC3G | 18499 | -0,32988682 | -0,13296615 | TUMOR | Non-Smokers |
| GSE50081 | DEFENSE RESPONSE | APOL3 | 20554 | -0,35327926 | -0,04413433 | TUMOR | Smokers |
| GSE32863 | DEFENSE RESPONSE | APOL3 | 36586 | -0,68223792 | -0,022233423 | TUMOR | Smokers |
| TCGA | DEFENSE RESPONSE | APOL3 | 16124 | -0,06405891 | -0,3893154 | TUMOR | Smokers |
| GSE32863 | IMMUNE_RESPONSE | AQP9 | 34765 | -0,37716487 | -0,30686682 | TUMOR | Smokers |
| GSE32863 | IMMUNE SYSTEM PROCESS | AQP9 | 34765 | -0,37716487 | -0,26992336 | TUMOR | Smokers |
| GSE32863 | IMMUNE_RESPONSE | ARHGDIB | 33466 | -0,31473652 | -0,39984903 | TUMOR | Smokers |
| TCGA | IMMUNE_RESPONSE | ARHGDIB | 20301 | -0,19709848 | -0,010618637 | TUMOR | Smokers |
| GSE32863 | IMMUNE SYSTEM PROCESS | ARHGDIB | 33466 | -0,31473652 | -0,34942192 | TUMOR | Smokers |
| TCGA | IMMUNE SYSTEM PROCESS | ARHGDIB | 20301 | -0,19709848 | -0,006768719 | TUMOR | Smokers |
| GSE47115 | IMMUNE SYSTEM PROCESS | AZU1 | 20486 | -0,65468824 | -0,08778739 | TUMOR | Smokers |
| GSE32863 | IMMUNE SYSTEM PROCESS | AZU1 | 29396 | -0,1982764 | -0,39615184 | TUMOR | Smokers |
| TCGA | IMMUNE SYSTEM PROCESS | AZU1 | 17047 | -0,0761435 | -0,31589574 | TUMOR | Smokers |
| GSE47115 | DEFENSE RESPONSE | AZU1 | 20486 | -0,65468824 | -0,03060874 | TUMOR | Smokers |
| GSE32863 | DEFENSE RESPONSE | AZU1 | 29396 | -0,1982764 | -0,40567392 | TUMOR | Smokers |
| TCGA | DEFENSE RESPONSE | AZU1 | 17047 | -0,0761435 | -0,35038614 | TUMOR | Smokers |
| GSE32863 | IMMUNE_RESPONSE | BCAR1 | 34551 | -0,36508149 | -0,3219643 | TUMOR | Smokers |
| GSE32863 | IMMUNE SYSTEM PROCESS | BCAR1 | 34551 | -0,36508149 | -0,2900115 | TUMOR | Smokers |
| GSE32863 | REGULATION OF IMMUNE SYSTEM PROCESS | BCAR1 | 34551 | -0,36508149 | -0,2878529 | TUMOR | Smokers |
| TCGA | IMMUNE_RESPONSE | BCL10 | 16030 | -0,06304125 | -0,38533694 | TUMOR | Smokers |
| TCGA | IMMUNE SYSTEM PROCESS | BCL10 | 16030 | -0,06304125 | -0,34550714 | TUMOR | Smokers |
| TCGA | DEFENSE RESPONSE | BCL10 | 16030 | -0,06304125 | -0,40022576 | TUMOR | Smokers |
| GSE32863 | IMMUNE_RESPONSE | BCL2 | 27306 | -0,1717484 | -0,38124993 | TUMOR | Non-Smokers |
| TCGA | IMMUNE_RESPONSE | BCL2 | 16024 | -0,06300169 | -0,38917074 | TUMOR | Smokers |
| TCGA | IMMUNE SYSTEM PROCESS | BCL2 | 16024 | -0,06300169 | -0,34822157 | TUMOR | Smokers |
| TCGA | DEFENSE RESPONSE | BCL2 | 16024 | -0,06300169 | -0,4037851 | TUMOR | Smokers |
| GSE47115 | CELLULAR DEFENSE RESPONSE | BECN1 | 13817 | -0,13388151 | -0,40628603 | TUMOR | Smokers |
| GSE50081 | IMMUNE_RESPONSE | BLNK | 19595 | -0,19333164 | -0,19278282 | TUMOR | Smokers |
| GSE47115 | IMMUNE_RESPONSE | BLNK | 17558 | -0,29748213 | -0,38503087 | TUMOR | Smokers |
| GSE32863 | IMMUNE_RESPONSE | BLNK | 34890 | -0,42735595 | -0,22328566 | TUMOR | Non-Smokers |
| GSE50081 | IMMUNE SYSTEM PROCESS | BLNK | 19595 | -0,19333164 | -0,14436151 | TUMOR | Smokers |
| GSE47115 | IMMUNE SYSTEM PROCESS | BLNK | 17558 | -0,29748213 | -0,36049435 | TUMOR | Smokers |
| GSE32863 | IMMUNE SYSTEM PROCESS | BLNK | 34890 | -0,42735595 | -0,22072063 | TUMOR | Non-Smokers |
| GSE50081 | DEFENSE RESPONSE | BLNK | 19595 | -0,19333164 | -0,27332333 | TUMOR | Smokers |
| GSE47115 | DEFENSE RESPONSE | BLNK | 17558 | -0,29748213 | -0,26041138 | TUMOR | Smokers |
| GSE32863 | DEFENSE RESPONSE | BLNK | 34890 | -0,42735595 | -0,13527204 | TUMOR | Non-Smokers |
| GSE50081 | IMMUNE_RESPONSE | BST1 | 17647 | -0,12103555 | -0,3719474 | TUMOR | Smokers |
| GSE32863 | IMMUNE_RESPONSE | BST1 | 32511 | -0,31466678 | -0,2920907 | TUMOR | Non-Smokers |
| GSE32863 | IMMUNE_RESPONSE | BST1 | 36555 | -0,64846969 | -0,062394693 | TUMOR | Smokers |
| TCGA | IMMUNE_RESPONSE | BST1 | 15016 | -0,18099928 | -0,3691411 | TUMOR | Non-Smokers |
| GSE50081 | IMMUNE SYSTEM PROCESS | BST1 | 17647 | -0,12103555 | -0,32827085 | TUMOR | Smokers |
| GSE32863 | IMMUNE SYSTEM PROCESS | BST1 | 32511 | -0,31466678 | -0,29170653 | TUMOR | Non-Smokers |
| GSE32863 | IMMUNE SYSTEM PROCESS | BST1 | 36555 | -0,64846969 | -0,045261405 | TUMOR | Smokers |
| TCGA | IMMUNE SYSTEM PROCESS | BST1 | 15285 | -0,05456663 | -0,3710241 | TUMOR | Smokers |
| TCGA | IMMUNE SYSTEM PROCESS | BST1 | 15016 | -0,18099928 | -0,36120173 | TUMOR | Non-Smokers |
| GSE47115 | IMMUNE_RESPONSE | BST2 | 20690 | -0,9063136 | 0,001659017 | TUMOR | Smokers |
| GSE32863 | IMMUNE_RESPONSE | BST2 | 35213 | -0,4109585 | -0,25971976 | TUMOR | Smokers |
| TCGA | IMMUNE_RESPONSE | BST2 | 17549 | -0,08326285 | -0,2989519 | TUMOR | Smokers |
| GSE47115 | IMMUNE SYSTEM PROCESS | BST2 | 20690 | -0,9063136 | 0,001666638 | TUMOR | Smokers |
| GSE32863 | IMMUNE SYSTEM PROCESS | BST2 | 35213 | -0,4109585 | -0,21653266 | TUMOR | Smokers |
| TCGA | IMMUNE SYSTEM PROCESS | BST2 | 17549 | -0,08326285 | -0,272486 | TUMOR | Smokers |
| GSE32863 | IMMUNE_RESPONSE | C1QBP | 36058 | -0,54836065 | -0,09815263 | TUMOR | Non-Smokers |
| GSE32863 | IMMUNE SYSTEM PROCESS | C1QBP | 36058 | -0,54836065 | -0,11232155 | TUMOR | Non-Smokers |
| GSE50081 | IMMUNE_RESPONSE | C2 | 18872 | -0,15812217 | -0,2594158 | TUMOR | Smokers |
| GSE32863 | IMMUNE_RESPONSE | C2 | 31484 | -0,25002426 | -0,40213925 | TUMOR | Smokers |
| GSE50081 | IMMUNE SYSTEM PROCESS | C2 | 18872 | -0,15812217 | -0,23586798 | TUMOR | Smokers |
| GSE32863 | IMMUNE SYSTEM PROCESS | C2 | 31484 | -0,25002426 | -0,37324387 | TUMOR | Smokers |
| GSE50081 | DEFENSE RESPONSE | C2 | 18872 | -0,15812217 | -0,30092674 | TUMOR | Smokers |
| GSE47115 | DEFENSE RESPONSE | C2 | 15569 | -0,20326053 | -0,34342173 | TUMOR | Smokers |
| GSE32863 | DEFENSE RESPONSE | C2 | 31484 | -0,25002426 | -0,3727657 | TUMOR | Smokers |
| GSE50081 | REGULATION OF IMMUNE SYSTEM PROCESS | C2 | 18872 | -0,15812217 | -0,28432325 | TUMOR | Smokers |
| GSE47115 | REGULATION OF IMMUNE SYSTEM PROCESS | C2 | 15569 | -0,20326053 | -0,38059288 | TUMOR | Smokers |
| GSE32863 | REGULATION OF IMMUNE SYSTEM PROCESS | C2 | 31484 | -0,25002426 | -0,326795 | TUMOR | Smokers |
| GSE47115 | DEFENSE RESPONSE | C3AR1 | 17046 | -0,2701858 | -0,3001271 | TUMOR | Smokers |
| GSE32863 | DEFENSE RESPONSE | C3AR1 | 31145 | -0,2402844 | -0,3839437 | TUMOR | Smokers |
| GSE32863 | DEFENSE RESPONSE | C5 | 32141 | -0,26823077 | -0,35606927 | TUMOR | Smokers |
| GSE32863 | IMMUNE_RESPONSE | C5AR1 | 34187 | -0,34625316 | -0,33233753 | TUMOR | Smokers |
| GSE32863 | IMMUNE SYSTEM PROCESS | C5AR1 | 34187 | -0,34625316 | -0,29512355 | TUMOR | Smokers |
| GSE32863 | CELLULAR DEFENSE RESPONSE | C5AR1 | 34187 | -0,34625316 | -0,40322816 | TUMOR | Smokers |
| GSE32863 | DEFENSE RESPONSE | C5AR1 | 34187 | -0,34625316 | -0,2948852 | TUMOR | Smokers |
| TCGA | IMMUNE_RESPONSE | CADM1 | 16287 | -0,22185723 | -0,3173705 | TUMOR | Non-Smokers |
| TCGA | IMMUNE SYSTEM PROCESS | CADM1 | 16287 | -0,22185723 | -0,32658744 | TUMOR | Non-Smokers |
| TCGA | REGULATION OF IMMUNE SYSTEM PROCESS | CADM1 | 16287 | -0,22185723 | -0,39422145 | TUMOR | Non-Smokers |
| GSE32863 | IMMUNE SYSTEM PROCESS | CALCA | 34836 | -0,38251609 | -0,25071964 | TUMOR | Smokers |
| GSE32863 | DEFENSE RESPONSE | CAMP | 36557 | -0,65105379 | -0,0436239 | TUMOR | Smokers |
| TCGA | IMMUNE_RESPONSE | CCBP2 | 19198 | -0,11689977 | -0,13521993 | TUMOR | Smokers |
| TCGA | IMMUNE SYSTEM PROCESS | CCBP2 | 19198 | -0,11689977 | -0,12067702 | TUMOR | Smokers |
| GSE50081 | DEFENSE RESPONSE | CCL11 | 17612 | -0,12027367 | -0,41000617 | TUMOR | Smokers |
| GSE47115 | DEFENSE RESPONSE | CCL11 | 17100 | -0,27318591 | -0,28831547 | TUMOR | Smokers |
| TCGA | DEFENSE RESPONSE | CCL11 | 20061 | -0,15680797 | -0,060938947 | TUMOR | Smokers |
| GSE47115 | DEFENSE RESPONSE | CCL13 | 16892 | -0,26254594 | -0,32101154 | TUMOR | Smokers |
| GSE32863 | DEFENSE RESPONSE | CCL13 | 27843 | -0,18387675 | -0,36594796 | TUMOR | Non-Smokers |
| GSE32863 | DEFENSE RESPONSE | CCL13 | 30733 | -0,23029627 | -0,38442826 | TUMOR | Smokers |
| GSE50081 | IMMUNE_RESPONSE | CCL18 | 19178 | -0,17161134 | -0,23326804 | TUMOR | Smokers |
| GSE47115 | IMMUNE_RESPONSE | CCL18 | 17722 | -0,3069413 | -0,36606678 | TUMOR | Smokers |
| GSE32863 | IMMUNE_RESPONSE | CCL18 | 33722 | -0,32623336 | -0,3705591 | TUMOR | Smokers |
| GSE50081 | IMMUNE SYSTEM PROCESS | CCL18 | 19178 | -0,17161134 | -0,18503611 | TUMOR | Smokers |
| GSE47115 | IMMUNE SYSTEM PROCESS | CCL18 | 17722 | -0,3069413 | -0,34913868 | TUMOR | Smokers |
| GSE32863 | IMMUNE SYSTEM PROCESS | CCL18 | 33722 | -0,32623336 | -0,32948583 | TUMOR | Smokers |
| GSE50081 | IMMUNE_RESPONSE | CCL19 | 16204 | -0,08771534 | -0,41006535 | TUMOR | Smokers |
| GSE32863 | IMMUNE_RESPONSE | CCL19 | 27667 | -0,17976135 | -0,3778814 | TUMOR | Non-Smokers |
| GSE32863 | IMMUNE_RESPONSE | CCL19 | 28678 | -0,18258312 | -0,43614724 | TUMOR | Smokers |
| TCGA | IMMUNE_RESPONSE | CCL19 | 18589 | -0,10188673 | -0,2337834 | TUMOR | Smokers |
| GSE50081 | IMMUNE SYSTEM PROCESS | CCL19 | 16204 | -0,08771534 | -0,36016098 | TUMOR | Smokers |
| GSE32863 | IMMUNE SYSTEM PROCESS | CCL19 | 27667 | -0,17976135 | -0,39487845 | TUMOR | Non-Smokers |
| GSE32863 | IMMUNE SYSTEM PROCESS | CCL19 | 28678 | -0,18258312 | -0,40878502 | TUMOR | Smokers |
| TCGA | IMMUNE SYSTEM PROCESS | CCL19 | 18589 | -0,10188673 | -0,19999443 | TUMOR | Smokers |
| GSE50081 | IMMUNE_RESPONSE | CCL2 | 16146 | -0,08673228 | -0,41092345 | TUMOR | Smokers |
| GSE32863 | IMMUNE_RESPONSE | CCL2 | 29480 | -0,20008646 | -0,42184353 | TUMOR | Smokers |
| TCGA | IMMUNE_RESPONSE | CCL2 | 17476 | -0,26887456 | -0,23268697 | TUMOR | Non-Smokers |
| GSE50081 | IMMUNE SYSTEM PROCESS | CCL2 | 16146 | -0,08673228 | -0,36276323 | TUMOR | Smokers |
| GSE32863 | IMMUNE SYSTEM PROCESS | CCL2 | 29480 | -0,20008646 | -0,395654 | TUMOR | Smokers |
| TCGA | IMMUNE SYSTEM PROCESS | CCL2 | 17476 | -0,26887456 | -0,24360043 | TUMOR | Non-Smokers |
| GSE47115 | IMMUNE_RESPONSE | CCL20 | 20453 | -0,63698548 | -0,08632523 | TUMOR | Smokers |
| GSE32863 | IMMUNE_RESPONSE | CCL20 | 35827 | -0,51302469 | -0,15111805 | TUMOR | Non-Smokers |
| TCGA | IMMUNE_RESPONSE | CCL20 | 17164 | -0,25560442 | -0,23003915 | TUMOR | Non-Smokers |
| GSE47115 | IMMUNE SYSTEM PROCESS | CCL20 | 20453 | -0,63698548 | -0,0945169 | TUMOR | Smokers |
| GSE32863 | IMMUNE SYSTEM PROCESS | CCL20 | 35827 | -0,51302469 | -0,1474637 | TUMOR | Non-Smokers |
| TCGA | IMMUNE SYSTEM PROCESS | CCL20 | 17164 | -0,25560442 | -0,24145329 | TUMOR | Non-Smokers |
| GSE47115 | DEFENSE RESPONSE | CCL20 | 20453 | -0,63698548 | -0,06320024 | TUMOR | Smokers |
| GSE32863 | DEFENSE RESPONSE | CCL20 | 35827 | -0,51302469 | -0,06335475 | TUMOR | Non-Smokers |
| GSE50081 | IMMUNE_RESPONSE | CCL21 | 18785 | -0,15511471 | -0,29469135 | TUMOR | Smokers |
| TCGA | IMMUNE_RESPONSE | CCL21 | 16139 | -0,06424705 | -0,38235912 | TUMOR | Smokers |
| TCGA | IMMUNE_RESPONSE | CCL21 | 16125 | -0,21548536 | -0,32566446 | TUMOR | Non-Smokers |
| GSE50081 | IMMUNE SYSTEM PROCESS | CCL21 | 18785 | -0,15511471 | -0,26563346 | TUMOR | Smokers |
| TCGA | IMMUNE SYSTEM PROCESS | CCL21 | 16139 | -0,06424705 | -0,34482563 | TUMOR | Smokers |
| TCGA | IMMUNE SYSTEM PROCESS | CCL21 | 16125 | -0,21548536 | -0,33367133 | TUMOR | Non-Smokers |
| GSE50081 | DEFENSE RESPONSE | CCL21 | 18785 | -0,15511471 | -0,32100165 | TUMOR | Smokers |
| TCGA | DEFENSE RESPONSE | CCL21 | 16139 | -0,06424705 | -0,38613087 | TUMOR | Smokers |
| GSE50081 | IMMUNE_RESPONSE | CCL22 | 20194 | -0,24201533 | -0,112078846 | TUMOR | Smokers |
| GSE32863 | IMMUNE_RESPONSE | CCL22 | 29124 | -0,19233142 | -0,4306374 | TUMOR | Smokers |
| TCGA | IMMUNE_RESPONSE | CCL22 | 20285 | -0,18896329 | -0,022636382 | TUMOR | Smokers |
| GSE50081 | IMMUNE SYSTEM PROCESS | CCL22 | 20194 | -0,24201533 | -0,085776344 | TUMOR | Smokers |
| GSE32863 | IMMUNE SYSTEM PROCESS | CCL22 | 29124 | -0,19233142 | -0,4052003 | TUMOR | Smokers |
| TCGA | IMMUNE SYSTEM PROCESS | CCL22 | 20285 | -0,18896329 | -0,015286892 | TUMOR | Smokers |
| GSE50081 | DEFENSE RESPONSE | CCL22 | 20194 | -0,24201533 | -0,17780755 | TUMOR | Smokers |
| GSE32863 | DEFENSE RESPONSE | CCL22 | 29124 | -0,19233142 | -0,41776556 | TUMOR | Smokers |
| TCGA | DEFENSE RESPONSE | CCL22 | 20285 | -0,18896329 | -0,020596763 | TUMOR | Smokers |
| GSE50081 | IMMUNE_RESPONSE | CCL23 | 20160 | -0,23742472 | -0,12054248 | TUMOR | Smokers |
| GSE32863 | IMMUNE_RESPONSE | CCL23 | 28667 | -0,2033276 | -0,3589718 | TUMOR | Non-Smokers |
| GSE32863 | IMMUNE_RESPONSE | CCL23 | 35199 | -0,4098435 | -0,26705986 | TUMOR | Smokers |
| TCGA | IMMUNE_RESPONSE | CCL23 | 15847 | -0,20635831 | -0,33811408 | TUMOR | Non-Smokers |
| GSE50081 | IMMUNE SYSTEM PROCESS | CCL23 | 20160 | -0,23742472 | -0,09157591 | TUMOR | Smokers |
| GSE32863 | IMMUNE SYSTEM PROCESS | CCL23 | 28667 | -0,2033276 | -0,37962794 | TUMOR | Non-Smokers |
| GSE32863 | IMMUNE SYSTEM PROCESS | CCL23 | 35199 | -0,4098435 | -0,22188374 | TUMOR | Smokers |
| TCGA | IMMUNE SYSTEM PROCESS | CCL23 | 14980 | -0,05150415 | -0,37380373 | TUMOR | Smokers |
| TCGA | IMMUNE SYSTEM PROCESS | CCL23 | 15847 | -0,20635831 | -0,34187174 | TUMOR | Non-Smokers |
| GSE50081 | DEFENSE RESPONSE | CCL23 | 20160 | -0,23742472 | -0,18549782 | TUMOR | Smokers |
| GSE32863 | DEFENSE RESPONSE | CCL23 | 28667 | -0,2033276 | -0,3618535 | TUMOR | Non-Smokers |
| GSE32863 | DEFENSE RESPONSE | CCL23 | 35199 | -0,4098435 | -0,26021296 | TUMOR | Smokers |
| TCGA | DEFENSE RESPONSE | CCL23 | 14980 | -0,05150415 | -0,41530603 | TUMOR | Smokers |
| GSE50081 | IMMUNE_RESPONSE | CCL24 | 17806 | -0,12454692 | -0,36416197 | TUMOR | Smokers |
| GSE32863 | IMMUNE_RESPONSE | CCL24 | 29146 | -0,2146299 | -0,35235095 | TUMOR | Non-Smokers |
| GSE50081 | IMMUNE SYSTEM PROCESS | CCL24 | 17806 | -0,12454692 | -0,32458705 | TUMOR | Smokers |
| GSE32863 | IMMUNE SYSTEM PROCESS | CCL24 | 29146 | -0,2146299 | -0,373418 | TUMOR | Non-Smokers |
| GSE50081 | DEFENSE RESPONSE | CCL24 | 17806 | -0,12454692 | -0,3907718 | TUMOR | Smokers |
| GSE32863 | DEFENSE RESPONSE | CCL24 | 29146 | -0,2146299 | -0,34966582 | TUMOR | Non-Smokers |
| GSE47115 | IMMUNE_RESPONSE | CCL25 | 20364 | -0,60753709 | -0,13746676 | TUMOR | Smokers |
| TCGA | IMMUNE_RESPONSE | CCL25 | 19425 | -0,12314592 | -0,0842917 | TUMOR | Smokers |
| GSE47115 | IMMUNE SYSTEM PROCESS | CCL25 | 20364 | -0,60753709 | -0,1379107 | TUMOR | Smokers |
| TCGA | IMMUNE SYSTEM PROCESS | CCL25 | 19425 | -0,12314592 | -0,069630444 | TUMOR | Smokers |
| GSE50081 | IMMUNE_RESPONSE | CCL26 | 17715 | -0,12249223 | -0,37013105 | TUMOR | Smokers |
| GSE32863 | IMMUNE_RESPONSE | CCL26 | 29870 | -0,23326422 | -0,35081345 | TUMOR | Non-Smokers |
| GSE50081 | IMMUNE SYSTEM PROCESS | CCL26 | 17715 | -0,12249223 | -0,3278136 | TUMOR | Smokers |
| GSE32863 | IMMUNE SYSTEM PROCESS | CCL26 | 29870 | -0,23326422 | -0,36937952 | TUMOR | Non-Smokers |
| GSE50081 | DEFENSE RESPONSE | CCL26 | 17715 | -0,12249223 | -0,40555528 | TUMOR | Smokers |
| GSE32863 | DEFENSE RESPONSE | CCL26 | 29870 | -0,23326422 | -0,3384541 | TUMOR | Non-Smokers |
| GSE47115 | IMMUNE_RESPONSE | CCL27 | 18386 | -0,34527785 | -0,34022135 | TUMOR | Smokers |
| TCGA | IMMUNE_RESPONSE | CCL27 | 15991 | -0,06252535 | -0,3916613 | TUMOR | Smokers |
| GSE47115 | IMMUNE SYSTEM PROCESS | CCL27 | 18386 | -0,34527785 | -0,33123434 | TUMOR | Smokers |
| TCGA | IMMUNE SYSTEM PROCESS | CCL27 | 15991 | -0,06252535 | -0,34958708 | TUMOR | Smokers |
| GSE47115 | DEFENSE RESPONSE | CCL3 | 17783 | -0,31041637 | -0,26057914 | TUMOR | Smokers |
| GSE32863 | DEFENSE RESPONSE | CCL3 | 32076 | -0,26627573 | -0,3631731 | TUMOR | Smokers |
| GSE47115 | DEFENSE RESPONSE | CCL3L3 | 16581 | -0,24771005 | -0,33297047 | TUMOR | Smokers |
| GSE32863 | DEFENSE RESPONSE | CCL3L3 | 35709 | -0,45520169 | -0,18838757 | TUMOR | Smokers |
| GSE32863 | IMMUNE_RESPONSE | CCL4 | 28241 | -0,1931473 | -0,3659016 | TUMOR | Non-Smokers |
| GSE32863 | IMMUNE SYSTEM PROCESS | CCL4 | 28241 | -0,1931473 | -0,38873288 | TUMOR | Non-Smokers |
| GSE32863 | DEFENSE RESPONSE | CCL4 | 28241 | -0,1931473 | -0,36375904 | TUMOR | Non-Smokers |
| GSE47115 | IMMUNE_RESPONSE | CCL5 | 20552 | -0,70781159 | -0,07858798 | TUMOR | Smokers |
| GSE32863 | IMMUNE_RESPONSE | CCL5 | 34508 | -0,4030425 | -0,25197434 | TUMOR | Non-Smokers |
| GSE32863 | IMMUNE_RESPONSE | CCL5 | 35738 | -0,45817143 | -0,17756452 | TUMOR | Smokers |
| TCGA | IMMUNE_RESPONSE | CCL5 | 15763 | -0,05995391 | -0,4125181 | TUMOR | Smokers |
| GSE47115 | IMMUNE SYSTEM PROCESS | CCL5 | 20552 | -0,70781159 | -0,073516436 | TUMOR | Smokers |
| GSE32863 | IMMUNE SYSTEM PROCESS | CCL5 | 34508 | -0,4030425 | -0,24317795 | TUMOR | Non-Smokers |
| GSE32863 | IMMUNE SYSTEM PROCESS | CCL5 | 35738 | -0,45817143 | -0,1530895 | TUMOR | Smokers |
| TCGA | IMMUNE SYSTEM PROCESS | CCL5 | 15763 | -0,05995391 | -0,3616864 | TUMOR | Smokers |
| GSE47115 | CELLULAR DEFENSE RESPONSE | CCL5 | 20552 | -0,70781159 | 0,008322131 | TUMOR | Smokers |
| GSE32863 | CELLULAR DEFENSE RESPONSE | CCL5 | 34508 | -0,4030425 | -0,24801117 | TUMOR | Non-Smokers |
| GSE32863 | CELLULAR DEFENSE RESPONSE | CCL5 | 35738 | -0,45817143 | -0,26699707 | TUMOR | Smokers |
| TCGA | CELLULAR DEFENSE RESPONSE | CCL5 | 15763 | -0,05995391 | -0,34052473 | TUMOR | Smokers |
| GSE47115 | DEFENSE RESPONSE | CCL5 | 20552 | -0,70781159 | -0,0214056 | TUMOR | Smokers |
| GSE32863 | DEFENSE RESPONSE | CCL5 | 34508 | -0,4030425 | -0,18199223 | TUMOR | Non-Smokers |
| GSE32863 | DEFENSE RESPONSE | CCL5 | 35738 | -0,45817143 | -0,18160187 | TUMOR | Smokers |
| TCGA | DEFENSE RESPONSE | CCL5 | 15763 | -0,05995391 | -0,41717285 | TUMOR | Smokers |
| GSE50081 | IMMUNE_RESPONSE | CCR1 | 15963 | -0,08251755 | -0,41284522 | TUMOR | Smokers |
| GSE47115 | IMMUNE_RESPONSE | CCR1 | 18187 | -0,33303639 | -0,34862596 | TUMOR | Smokers |
| TCGA | IMMUNE_RESPONSE | CCR1 | 16607 | -0,23401666 | -0,2823827 | TUMOR | Non-Smokers |
| GSE50081 | IMMUNE SYSTEM PROCESS | CCR1 | 15963 | -0,08251755 | -0,36714312 | TUMOR | Smokers |
| GSE47115 | IMMUNE SYSTEM PROCESS | CCR1 | 18187 | -0,33303639 | -0,3388565 | TUMOR | Smokers |
| TCGA | IMMUNE SYSTEM PROCESS | CCR1 | 14655 | -0,04806451 | -0,3695653 | TUMOR | Smokers |
| TCGA | IMMUNE SYSTEM PROCESS | CCR1 | 16607 | -0,23401666 | -0,29094976 | TUMOR | Non-Smokers |
| GSE47115 | DEFENSE RESPONSE | CCR1 | 18187 | -0,33303639 | -0,25190648 | TUMOR | Smokers |
| TCGA | DEFENSE RESPONSE | CCR1 | 14655 | -0,04806451 | -0,4175353 | TUMOR | Smokers |
| GSE50081 | IMMUNE_RESPONSE | CCR2 | 20073 | -0,22668095 | -0,1454615 | TUMOR | Smokers |
| GSE32863 | IMMUNE_RESPONSE | CCR2 | 32653 | -0,28390938 | -0,38347924 | TUMOR | Smokers |
| TCGA | IMMUNE_RESPONSE | CCR2 | 16177 | -0,06478719 | -0,37577373 | TUMOR | Smokers |
| TCGA | IMMUNE_RESPONSE | CCR2 | 17495 | -0,26996061 | -0,2269188 | TUMOR | Non-Smokers |
| GSE50081 | IMMUNE SYSTEM PROCESS | CCR2 | 20073 | -0,22668095 | -0,10882419 | TUMOR | Smokers |
| GSE32863 | IMMUNE SYSTEM PROCESS | CCR2 | 32653 | -0,28390938 | -0,3526055 | TUMOR | Smokers |
| TCGA | IMMUNE SYSTEM PROCESS | CCR2 | 16177 | -0,06478719 | -0,34054232 | TUMOR | Smokers |
| TCGA | IMMUNE SYSTEM PROCESS | CCR2 | 17495 | -0,26996061 | -0,23985507 | TUMOR | Non-Smokers |
| GSE50081 | CELLULAR DEFENSE RESPONSE | CCR2 | 20073 | -0,22668095 | -0,020554543 | TUMOR | Smokers |
| GSE32863 | CELLULAR DEFENSE RESPONSE | CCR2 | 26915 | -0,16331188 | -0,4597888 | TUMOR | Non-Smokers |
| GSE32863 | CELLULAR DEFENSE RESPONSE | CCR2 | 32653 | -0,28390938 | -0,4482379 | TUMOR | Smokers |
| TCGA | CELLULAR DEFENSE RESPONSE | CCR2 | 16177 | -0,06478719 | -0,32118842 | TUMOR | Smokers |
| GSE50081 | DEFENSE RESPONSE | CCR2 | 20073 | -0,22668095 | -0,19932044 | TUMOR | Smokers |
| GSE32863 | DEFENSE RESPONSE | CCR2 | 32653 | -0,28390938 | -0,3425379 | TUMOR | Smokers |
| TCGA | DEFENSE RESPONSE | CCR2 | 16177 | -0,06478719 | -0,37615588 | TUMOR | Smokers |
| GSE50081 | CELLULAR DEFENSE RESPONSE | CCR3 | 18587 | -0,14917661 | -0,25385007 | TUMOR | Smokers |
| GSE50081 | DEFENSE RESPONSE | CCR3 | 18587 | -0,14917661 | -0,35853595 | TUMOR | Smokers |
| GSE32863 | IMMUNE_RESPONSE | CCR4 | 34760 | -0,41799283 | -0,2277235 | TUMOR | Non-Smokers |
| GSE32863 | IMMUNE_RESPONSE | CCR4 | 33998 | -0,33840749 | -0,35297114 | TUMOR | Smokers |
| TCGA | IMMUNE_RESPONSE | CCR4 | 17992 | -0,09050895 | -0,27489004 | TUMOR | Smokers |
| GSE32863 | IMMUNE SYSTEM PROCESS | CCR4 | 34760 | -0,41799283 | -0,22829476 | TUMOR | Non-Smokers |
| GSE32863 | IMMUNE SYSTEM PROCESS | CCR4 | 33998 | -0,33840749 | -0,3091042 | TUMOR | Smokers |
| TCGA | IMMUNE SYSTEM PROCESS | CCR4 | 17992 | -0,09050895 | -0,24868117 | TUMOR | Smokers |
| GSE32863 | DEFENSE RESPONSE | CCR4 | 34760 | -0,41799283 | -0,1463761 | TUMOR | Non-Smokers |
| GSE32863 | DEFENSE RESPONSE | CCR4 | 33998 | -0,33840749 | -0,30111933 | TUMOR | Smokers |
| TCGA | DEFENSE RESPONSE | CCR4 | 17992 | -0,09050895 | -0,26969388 | TUMOR | Smokers |
| GSE32863 | IMMUNE_RESPONSE | CCR5 | 34625 | -0,36972216 | -0,3101192 | TUMOR | Smokers |
| TCGA | IMMUNE_RESPONSE | CCR5 | 17318 | -0,07982355 | -0,3408095 | TUMOR | Smokers |
| GSE32863 | IMMUNE SYSTEM PROCESS | CCR5 | 34625 | -0,36972216 | -0,28174105 | TUMOR | Smokers |
| TCGA | IMMUNE SYSTEM PROCESS | CCR5 | 17318 | -0,07982355 | -0,29981953 | TUMOR | Smokers |
| GSE32863 | CELLULAR DEFENSE RESPONSE | CCR5 | 34625 | -0,36972216 | -0,38924634 | TUMOR | Smokers |
| TCGA | CELLULAR DEFENSE RESPONSE | CCR5 | 17318 | -0,07982355 | -0,2627406 | TUMOR | Smokers |
| GSE32863 | DEFENSE RESPONSE | CCR5 | 34625 | -0,36972216 | -0,27714938 | TUMOR | Smokers |
| TCGA | DEFENSE RESPONSE | CCR5 | 17318 | -0,07982355 | -0,33529937 | TUMOR | Smokers |
| GSE50081 | IMMUNE_RESPONSE | CCR6 | 20296 | -0,25663176 | -0,09605745 | TUMOR | Smokers |
| GSE32863 | IMMUNE_RESPONSE | CCR6 | 36240 | -0,58494347 | -0,05038846 | TUMOR | Non-Smokers |
| TCGA | IMMUNE_RESPONSE | CCR6 | 19321 | -0,12011569 | -0,0951424 | TUMOR | Smokers |
| GSE50081 | IMMUNE SYSTEM PROCESS | CCR6 | 20296 | -0,25663176 | -0,07529113 | TUMOR | Smokers |
| GSE32863 | IMMUNE SYSTEM PROCESS | CCR6 | 36240 | -0,58494347 | -0,07308883 | TUMOR | Non-Smokers |
| TCGA | IMMUNE SYSTEM PROCESS | CCR6 | 19321 | -0,12011569 | -0,087618954 | TUMOR | Smokers |
| GSE50081 | CELLULAR DEFENSE RESPONSE | CCR6 | 20296 | -0,25663176 | 0,015032863 | TUMOR | Smokers |
| GSE32863 | CELLULAR DEFENSE RESPONSE | CCR6 | 36240 | -0,58494347 | 0,012607181 | TUMOR | Non-Smokers |
| TCGA | CELLULAR DEFENSE RESPONSE | CCR6 | 19321 | -0,12011569 | -0,070822105 | TUMOR | Smokers |
| GSE50081 | DEFENSE RESPONSE | CCR6 | 20296 | -0,25663176 | -0,1342383 | TUMOR | Smokers |
| GSE32863 | DEFENSE RESPONSE | CCR6 | 36240 | -0,58494347 | -0,018653868 | TUMOR | Non-Smokers |
| TCGA | DEFENSE RESPONSE | CCR6 | 19321 | -0,12011569 | -0,15857734 | TUMOR | Smokers |
| GSE50081 | DEFENSE RESPONSE | CCR7 | 17778 | -0,12390058 | -0,3942375 | TUMOR | Smokers |
| GSE47115 | DEFENSE RESPONSE | CCR7 | 19596 | -0,45249167 | -0,17797042 | TUMOR | Smokers |
| GSE32863 | DEFENSE RESPONSE | CCR7 | 32047 | -0,29781535 | -0,28618392 | TUMOR | Non-Smokers |
| GSE32863 | DEFENSE RESPONSE | CCR7 | 36513 | -0,62163597 | -0,06383035 | TUMOR | Smokers |
| TCGA | DEFENSE RESPONSE | CCR7 | 20293 | -0,19318347 | -0,009275544 | TUMOR | Smokers |
| TCGA | IMMUNE_RESPONSE | CCR8 | 15704 | -0,05927999 | -0,41744673 | TUMOR | Smokers |
| TCGA | IMMUNE SYSTEM PROCESS | CCR8 | 15704 | -0,05927999 | -0,3644762 | TUMOR | Smokers |
| GSE47115 | IMMUNE_RESPONSE | CCR9 | 17657 | -0,30316627 | -0,36837277 | TUMOR | Smokers |
| GSE32863 | IMMUNE_RESPONSE | CCR9 | 30295 | -0,24499844 | -0,32611474 | TUMOR | Non-Smokers |
| TCGA | IMMUNE_RESPONSE | CCR9 | 18205 | -0,09430138 | -0,2732199 | TUMOR | Smokers |
| GSE47115 | IMMUNE SYSTEM PROCESS | CCR9 | 17657 | -0,30316627 | -0,3498919 | TUMOR | Smokers |
| GSE32863 | IMMUNE SYSTEM PROCESS | CCR9 | 30295 | -0,24499844 | -0,3493451 | TUMOR | Non-Smokers |
| TCGA | IMMUNE SYSTEM PROCESS | CCR9 | 18205 | -0,09430138 | -0,23721309 | TUMOR | Smokers |
| GSE47115 | CELLULAR DEFENSE RESPONSE | CCR9 | 17657 | -0,30316627 | -0,26754895 | TUMOR | Smokers |
| GSE32863 | CELLULAR DEFENSE RESPONSE | CCR9 | 30295 | -0,24499844 | -0,34158376 | TUMOR | Non-Smokers |
| TCGA | CELLULAR DEFENSE RESPONSE | CCR9 | 18205 | -0,09430138 | -0,119547285 | TUMOR | Smokers |
| GSE47115 | DEFENSE RESPONSE | CCR9 | 17657 | -0,30316627 | -0,2598988 | TUMOR | Smokers |
| GSE32863 | DEFENSE RESPONSE | CCR9 | 30295 | -0,24499844 | -0,3210705 | TUMOR | Non-Smokers |
| TCGA | DEFENSE RESPONSE | CCR9 | 18205 | -0,09430138 | -0,24634695 | TUMOR | Smokers |
| GSE50081 | IMMUNE_RESPONSE | CCRL1 | 18647 | -0,15109931 | -0,3202164 | TUMOR | Smokers |
| GSE32863 | IMMUNE_RESPONSE | CCRL1 | 27722 | -0,1809613 | -0,36920956 | TUMOR | Non-Smokers |
| TCGA | IMMUNE_RESPONSE | CCRL1 | 16985 | -0,24862345 | -0,27748087 | TUMOR | Non-Smokers |
| GSE50081 | IMMUNE SYSTEM PROCESS | CCRL1 | 18647 | -0,15109931 | -0,28268418 | TUMOR | Smokers |
| GSE32863 | IMMUNE SYSTEM PROCESS | CCRL1 | 27722 | -0,1809613 | -0,389251 | TUMOR | Non-Smokers |
| TCGA | IMMUNE SYSTEM PROCESS | CCRL1 | 16985 | -0,24862345 | -0,2807012 | TUMOR | Non-Smokers |
| GSE50081 | CELLULAR DEFENSE RESPONSE | CD160 | 17756 | -0,12333831 | -0,36403108 | TUMOR | Smokers |
| TCGA | CELLULAR DEFENSE RESPONSE | CD160 | 15178 | -0,05341438 | -0,38231152 | TUMOR | Smokers |
| GSE50081 | DEFENSE RESPONSE | CD160 | 17756 | -0,12333831 | -0,40277448 | TUMOR | Smokers |
| TCGA | DEFENSE RESPONSE | CD160 | 15178 | -0,05341438 | -0,408976 | TUMOR | Smokers |
| GSE50081 | IMMUNE_RESPONSE | CD164 | 17555 | -0,11856083 | -0,37757933 | TUMOR | Smokers |
| GSE32863 | IMMUNE_RESPONSE | CD164 | 29530 | -0,22438212 | -0,35458872 | TUMOR | Non-Smokers |
| GSE50081 | IMMUNE SYSTEM PROCESS | CD164 | 17555 | -0,11856083 | -0,33498847 | TUMOR | Smokers |
| GSE32863 | IMMUNE SYSTEM PROCESS | CD164 | 29530 | -0,22438212 | -0,3752895 | TUMOR | Non-Smokers |
| GSE50081 | CELLULAR DEFENSE RESPONSE | CD19 | 19971 | -0,21844718 | -0,0973009 | TUMOR | Smokers |
| GSE47115 | CELLULAR DEFENSE RESPONSE | CD19 | 17857 | -0,31406561 | -0,25404993 | TUMOR | Smokers |
| GSE32863 | CELLULAR DEFENSE RESPONSE | CD19 | 35135 | -0,44691515 | -0,127384 | TUMOR | Non-Smokers |
| GSE32863 | CELLULAR DEFENSE RESPONSE | CD19 | 36292 | -0,54547024 | -0,1754106 | TUMOR | Smokers |
| TCGA | CELLULAR DEFENSE RESPONSE | CD19 | 16783 | -0,07253515 | -0,30751294 | TUMOR | Smokers |
| GSE50081 | DEFENSE RESPONSE | CD19 | 19971 | -0,21844718 | -0,21177983 | TUMOR | Smokers |
| GSE47115 | DEFENSE RESPONSE | CD19 | 17857 | -0,31406561 | -0,258654 | TUMOR | Smokers |
| GSE32863 | DEFENSE RESPONSE | CD19 | 35135 | -0,44691515 | -0,1267545 | TUMOR | Non-Smokers |
| GSE32863 | DEFENSE RESPONSE | CD19 | 36292 | -0,54547024 | -0,11609238 | TUMOR | Smokers |
| TCGA | DEFENSE RESPONSE | CD19 | 16783 | -0,07253515 | -0,35993966 | TUMOR | Smokers |
| GSE50081 | IMMUNE_RESPONSE | CD1D | 20494 | -0,30509421 | -0,024347296 | TUMOR | Smokers |
| GSE32863 | IMMUNE_RESPONSE | CD1D | 30266 | -0,24418141 | -0,33450407 | TUMOR | Non-Smokers |
| GSE32863 | IMMUNE_RESPONSE | CD1D | 35651 | -0,44989404 | -0,18378724 | TUMOR | Smokers |
| TCGA | IMMUNE_RESPONSE | CD1D | 17499 | -0,08242591 | -0,30731168 | TUMOR | Smokers |
| GSE50081 | IMMUNE SYSTEM PROCESS | CD1D | 20494 | -0,30509421 | -0,016498687 | TUMOR | Smokers |
| GSE32863 | IMMUNE SYSTEM PROCESS | CD1D | 30266 | -0,24418141 | -0,35498327 | TUMOR | Non-Smokers |
| GSE32863 | IMMUNE SYSTEM PROCESS | CD1D | 35651 | -0,44989404 | -0,15708977 | TUMOR | Smokers |
| TCGA | IMMUNE SYSTEM PROCESS | CD1D | 17499 | -0,08242591 | -0,2778916 | TUMOR | Smokers |
| GSE50081 | DEFENSE RESPONSE | CD1D | 20494 | -0,30509421 | -0,06707605 | TUMOR | Smokers |
| GSE32863 | DEFENSE RESPONSE | CD1D | 30266 | -0,24418141 | -0,32870892 | TUMOR | Non-Smokers |
| GSE32863 | DEFENSE RESPONSE | CD1D | 35651 | -0,44989404 | -0,19432846 | TUMOR | Smokers |
| TCGA | DEFENSE RESPONSE | CD1D | 17499 | -0,08242591 | -0,32449195 | TUMOR | Smokers |
| GSE50081 | REGULATION OF IMMUNE SYSTEM PROCESS | CD1D | 20494 | -0,30509421 | -0,096511945 | TUMOR | Smokers |
| GSE10072 | REGULATION OF IMMUNE SYSTEM PROCESS | CD1D | 10795 | -0,31028438 | -0,37719616 | NORMAL | Non-Smokers |
| GSE32863 | REGULATION OF IMMUNE SYSTEM PROCESS | CD1D | 35651 | -0,44989404 | -0,1872784 | TUMOR | Smokers |
| TCGA | REGULATION OF IMMUNE SYSTEM PROCESS | CD1D | 14646 | -0,17034288 | -0,41054294 | TUMOR | Non-Smokers |
| GSE50081 | IMMUNE SYSTEM PROCESS | CD2 | 19029 | -0,16439015 | -0,20366539 | TUMOR | Smokers |
| GSE47115 | IMMUNE SYSTEM PROCESS | CD2 | 20566 | -0,72293133 | -0,05584983 | TUMOR | Smokers |
| GSE32863 | IMMUNE SYSTEM PROCESS | CD2 | 33873 | -0,37083024 | -0,2609482 | TUMOR | Non-Smokers |
| GSE32863 | IMMUNE SYSTEM PROCESS | CD2 | 34906 | -0,38810065 | -0,24722537 | TUMOR | Smokers |
| TCGA | IMMUNE SYSTEM PROCESS | CD2 | 16459 | -0,0681069 | -0,33848882 | TUMOR | Smokers |
| TCGA | IMMUNE SYSTEM PROCESS | CD2 | 16128 | -0,21557638 | -0,33004758 | TUMOR | Non-Smokers |
| GSE47115 | IMMUNE_RESPONSE | CD22 | 19211 | -0,41493407 | -0,3197894 | TUMOR | Smokers |
| TCGA | IMMUNE_RESPONSE | CD22 | 19775 | -0,1363343 | -0,058744185 | TUMOR | Smokers |
| GSE47115 | IMMUNE SYSTEM PROCESS | CD22 | 19211 | -0,41493407 | -0,30371624 | TUMOR | Smokers |
| TCGA | IMMUNE SYSTEM PROCESS | CD22 | 19775 | -0,1363343 | -0,055855006 | TUMOR | Smokers |
| GSE32863 | IMMUNE SYSTEM PROCESS | CD24 | 30987 | -0,26482388 | -0,3347388 | TUMOR | Non-Smokers |
| TCGA | IMMUNE SYSTEM PROCESS | CD24 | 19593 | -0,45980003 | -0,07469829 | TUMOR | Non-Smokers |
| GSE47115 | REGULATION OF IMMUNE SYSTEM PROCESS | CD24 | 15829 | -0,21448231 | -0,3804318 | TUMOR | Smokers |
| GSE10072 | REGULATION OF IMMUNE SYSTEM PROCESS | CD24 | 11528 | -0,37075832 | -0,3545966 | NORMAL | Non-Smokers |
| TCGA | REGULATION OF IMMUNE SYSTEM PROCESS | CD24 | 19593 | -0,45980003 | 0,014285747 | TUMOR | Non-Smokers |
| GSE47115 | IMMUNE_RESPONSE | CD274 | 18656 | -0,36431417 | -0,32797843 | TUMOR | Smokers |
| TCGA | IMMUNE_RESPONSE | CD274 | 17011 | -0,07555757 | -0,35606596 | TUMOR | Smokers |
| TCGA | IMMUNE_RESPONSE | CD274 | 15496 | -0,19557308 | -0,3606365 | TUMOR | Non-Smokers |
| GSE47115 | IMMUNE SYSTEM PROCESS | CD274 | 18656 | -0,36431417 | -0,32176283 | TUMOR | Smokers |
| TCGA | IMMUNE SYSTEM PROCESS | CD274 | 17011 | -0,07555757 | -0,31772944 | TUMOR | Smokers |
| TCGA | IMMUNE SYSTEM PROCESS | CD274 | 15496 | -0,19557308 | -0,35928863 | TUMOR | Non-Smokers |
| GSE50081 | IMMUNE SYSTEM PROCESS | CD276 | 17832 | -0,12508671 | -0,31422737 | TUMOR | Smokers |
| TCGA | IMMUNE SYSTEM PROCESS | CD276 | 19334 | -0,4108938 | -0,098960266 | TUMOR | Non-Smokers |
| TCGA | REGULATION OF IMMUNE SYSTEM PROCESS | CD276 | 19334 | -0,4108938 | -0,01063696 | TUMOR | Non-Smokers |
| GSE32863 | IMMUNE_RESPONSE | CD28 | 29746 | -0,20612307 | -0,41756645 | TUMOR | Smokers |
| TCGA | IMMUNE_RESPONSE | CD28 | 19166 | -0,38979337 | -0,11664348 | TUMOR | Non-Smokers |
| GSE32863 | IMMUNE SYSTEM PROCESS | CD28 | 29746 | -0,20612307 | -0,39436254 | TUMOR | Smokers |
| TCGA | IMMUNE SYSTEM PROCESS | CD28 | 19166 | -0,38979337 | -0,13927786 | TUMOR | Non-Smokers |
| GSE10072 | REGULATION OF IMMUNE SYSTEM PROCESS | CD28 | 12039 | -0,4253512 | -0,27329984 | NORMAL | Non-Smokers |
| GSE32863 | REGULATION OF IMMUNE SYSTEM PROCESS | CD28 | 29746 | -0,20612307 | -0,36661944 | TUMOR | Smokers |
| TCGA | REGULATION OF IMMUNE SYSTEM PROCESS | CD28 | 19166 | -0,38979337 | -0,10319872 | TUMOR | Non-Smokers |
| GSE50081 | CELLULAR DEFENSE RESPONSE | CD300C | 17083 | -0,10781112 | -0,39572725 | TUMOR | Smokers |
| GSE47115 | CELLULAR DEFENSE RESPONSE | CD300C | 14015 | -0,14163919 | -0,3951313 | TUMOR | Smokers |
| GSE32863 | CELLULAR DEFENSE RESPONSE | CD300C | 32454 | -0,27703881 | -0,46272862 | TUMOR | Smokers |
| TCGA | CELLULAR DEFENSE RESPONSE | CD300C | 14056 | -0,04196133 | -0,41678873 | TUMOR | Smokers |
| GSE50081 | DEFENSE RESPONSE | CD300C | 17083 | -0,10781112 | -0,4201773 | TUMOR | Smokers |
| GSE32863 | DEFENSE RESPONSE | CD300C | 32454 | -0,27703881 | -0,34649318 | TUMOR | Smokers |
| TCGA | IMMUNE SYSTEM PROCESS | CD34 | 18818 | -0,10670567 | -0,17679198 | TUMOR | Smokers |
| TCGA | IMMUNE SYSTEM PROCESS | CD34 | 19206 | -0,39415562 | -0,1276369 | TUMOR | Non-Smokers |
| GSE50081 | IMMUNE SYSTEM PROCESS | CD3D | 18055 | -0,13183166 | -0,2973162 | TUMOR | Smokers |
| GSE47115 | IMMUNE SYSTEM PROCESS | CD3D | 20645 | -0,81034368 | -0,039650004 | TUMOR | Smokers |
| GSE32863 | IMMUNE SYSTEM PROCESS | CD3D | 35317 | -0,46166357 | -0,19134885 | TUMOR | Non-Smokers |
| GSE32863 | IMMUNE SYSTEM PROCESS | CD3D | 35803 | -0,46754241 | -0,1483543 | TUMOR | Smokers |
| TCGA | IMMUNE SYSTEM PROCESS | CD3D | 16384 | -0,06714556 | -0,34446663 | TUMOR | Smokers |
| TCGA | IMMUNE SYSTEM PROCESS | CD3D | 17269 | -0,2598204 | -0,24228194 | TUMOR | Non-Smokers |
| GSE50081 | IMMUNE SYSTEM PROCESS | CD3E | 17012 | -0,10606927 | -0,3578824 | TUMOR | Smokers |
| GSE47115 | IMMUNE SYSTEM PROCESS | CD3E | 20309 | -0,5902741 | -0,1737735 | TUMOR | Smokers |
| GSE32863 | IMMUNE SYSTEM PROCESS | CD3E | 33164 | -0,34008241 | -0,2793184 | TUMOR | Non-Smokers |
| GSE32863 | IMMUNE SYSTEM PROCESS | CD3E | 30376 | -0,22141936 | -0,37868628 | TUMOR | Smokers |
| TCGA | IMMUNE SYSTEM PROCESS | CD3E | 16366 | -0,0669226 | -0,34677532 | TUMOR | Smokers |
| TCGA | IMMUNE SYSTEM PROCESS | CD3E | 19324 | -0,4093667 | -0,10560186 | TUMOR | Non-Smokers |
| GSE47115 | REGULATION OF IMMUNE SYSTEM PROCESS | CD3E | 20309 | -0,5902741 | -0,1637211 | TUMOR | Smokers |
| GSE32863 | REGULATION OF IMMUNE SYSTEM PROCESS | CD3E | 30376 | -0,22141936 | -0,36976877 | TUMOR | Smokers |
| TCGA | REGULATION OF IMMUNE SYSTEM PROCESS | CD3E | 19324 | -0,4093667 | -0,04409294 | TUMOR | Non-Smokers |
| GSE50081 | IMMUNE SYSTEM PROCESS | CD4 | 19070 | -0,16615403 | -0,1902913 | TUMOR | Smokers |
| GSE47115 | IMMUNE SYSTEM PROCESS | CD4 | 17483 | -0,29334813 | -0,36063746 | TUMOR | Smokers |
| GSE32863 | IMMUNE SYSTEM PROCESS | CD4 | 32489 | -0,31384572 | -0,295233 | TUMOR | Non-Smokers |
| GSE32863 | IMMUNE SYSTEM PROCESS | CD4 | 30279 | -0,21912935 | -0,38221356 | TUMOR | Smokers |
| TCGA | IMMUNE SYSTEM PROCESS | CD4 | 17175 | -0,07779679 | -0,30395174 | TUMOR | Smokers |
| TCGA | IMMUNE SYSTEM PROCESS | CD4 | 18274 | -0,31377509 | -0,17149521 | TUMOR | Non-Smokers |
| GSE50081 | DEFENSE RESPONSE | CD40 | 20581 | -0,38732716 | -0,016732313 | TUMOR | Smokers |
| GSE47115 | DEFENSE RESPONSE | CD40 | 18328 | -0,34135571 | -0,24685472 | TUMOR | Smokers |
| TCGA | DEFENSE RESPONSE | CD40 | 19589 | -0,1288472 | -0,13375512 | TUMOR | Smokers |
| GSE32863 | IMMUNE_RESPONSE | CD40LG | 35496 | -0,47782484 | -0,17992091 | TUMOR | Non-Smokers |
| TCGA | IMMUNE_RESPONSE | CD40LG | 18642 | -0,103032 | -0,2230309 | TUMOR | Smokers |
| GSE32863 | IMMUNE SYSTEM PROCESS | CD40LG | 35496 | -0,47782484 | -0,17762326 | TUMOR | Non-Smokers |
| TCGA | IMMUNE SYSTEM PROCESS | CD40LG | 18642 | -0,103032 | -0,19289306 | TUMOR | Smokers |
| GSE32863 | DEFENSE RESPONSE | CD40LG | 35496 | -0,47782484 | -0,08865216 | TUMOR | Non-Smokers |
| TCGA | DEFENSE RESPONSE | CD40LG | 18642 | -0,103032 | -0,22590496 | TUMOR | Smokers |
| TCGA | IMMUNE SYSTEM PROCESS | CD47 | 16935 | -0,07441983 | -0,32469267 | TUMOR | Smokers |
| TCGA | IMMUNE SYSTEM PROCESS | CD47 | 14973 | -0,17980039 | -0,3621819 | TUMOR | Non-Smokers |
| TCGA | REGULATION OF IMMUNE SYSTEM PROCESS | CD47 | 14973 | -0,17980039 | -0,39736295 | TUMOR | Non-Smokers |
| GSE47115 | DEFENSE RESPONSE | CD48 | 18406 | -0,3472043 | -0,22624482 | TUMOR | Smokers |
| GSE32863 | DEFENSE RESPONSE | CD48 | 35331 | -0,46264255 | -0,11630848 | TUMOR | Non-Smokers |
| GSE32863 | DEFENSE RESPONSE | CD48 | 33632 | -0,32179782 | -0,32426953 | TUMOR | Smokers |
| TCGA | DEFENSE RESPONSE | CD48 | 17376 | -0,08064424 | -0,328378 | TUMOR | Smokers |
| GSE32863 | CELLULAR DEFENSE RESPONSE | CD5L | 36503 | -0,61636966 | -0,09784791 | TUMOR | Smokers |
| TCGA | CELLULAR DEFENSE RESPONSE | CD5L | 19936 | -0,14590922 | 0,021364477 | TUMOR | Smokers |
| GSE32863 | DEFENSE RESPONSE | CD5L | 36503 | -0,61636966 | -0,0738326 | TUMOR | Smokers |
| TCGA | DEFENSE RESPONSE | CD5L | 19936 | -0,14590922 | -0,09174531 | TUMOR | Smokers |
| GSE50081 | IMMUNE_RESPONSE | CD7 | 19803 | -0,20717789 | -0,17715858 | TUMOR | Smokers |
| GSE47115 | IMMUNE_RESPONSE | CD7 | 20416 | -0,62311423 | -0,10709707 | TUMOR | Smokers |
| GSE32863 | IMMUNE_RESPONSE | CD7 | 34749 | -0,41748804 | -0,23524947 | TUMOR | Non-Smokers |
| GSE32863 | IMMUNE_RESPONSE | CD7 | 29841 | -0,20828032 | -0,40843305 | TUMOR | Smokers |
| TCGA | IMMUNE_RESPONSE | CD7 | 16679 | -0,07115814 | -0,3735672 | TUMOR | Smokers |
| GSE50081 | IMMUNE SYSTEM PROCESS | CD7 | 19803 | -0,20717789 | -0,13554853 | TUMOR | Smokers |
| GSE47115 | IMMUNE SYSTEM PROCESS | CD7 | 20416 | -0,62311423 | -0,10891037 | TUMOR | Smokers |
| GSE32863 | IMMUNE SYSTEM PROCESS | CD7 | 34749 | -0,41748804 | -0,23347111 | TUMOR | Non-Smokers |
| GSE32863 | IMMUNE SYSTEM PROCESS | CD7 | 29841 | -0,20828032 | -0,38824636 | TUMOR | Smokers |
| TCGA | IMMUNE SYSTEM PROCESS | CD7 | 16679 | -0,07115814 | -0,3294882 | TUMOR | Smokers |
| TCGA | IMMUNE_RESPONSE | CD74 | 17423 | -0,08143122 | -0,32496852 | TUMOR | Smokers |
| TCGA | IMMUNE_RESPONSE | CD74 | 18126 | -0,30565846 | -0,17245744 | TUMOR | Non-Smokers |
| TCGA | IMMUNE SYSTEM PROCESS | CD74 | 17423 | -0,08143122 | -0,28971544 | TUMOR | Smokers |
| TCGA | IMMUNE SYSTEM PROCESS | CD74 | 18126 | -0,30565846 | -0,19639108 | TUMOR | Non-Smokers |
| GSE50081 | IMMUNE_RESPONSE | CD79A | 16807 | -0,10130733 | -0,40757722 | TUMOR | Smokers |
| GSE32863 | IMMUNE_RESPONSE | CD79A | 29851 | -0,2329229 | -0,35467288 | TUMOR | Non-Smokers |
| GSE32863 | IMMUNE_RESPONSE | CD79A | 35169 | -0,40712893 | -0,27394035 | TUMOR | Smokers |
| TCGA | IMMUNE_RESPONSE | CD79A | 17802 | -0,08733109 | -0,29455987 | TUMOR | Smokers |
| GSE50081 | IMMUNE SYSTEM PROCESS | CD79A | 16807 | -0,10130733 | -0,35436413 | TUMOR | Smokers |
| GSE32863 | IMMUNE SYSTEM PROCESS | CD79A | 29851 | -0,2329229 | -0,37192684 | TUMOR | Non-Smokers |
| GSE32863 | IMMUNE SYSTEM PROCESS | CD79A | 35169 | -0,40712893 | -0,22677952 | TUMOR | Smokers |
| TCGA | IMMUNE SYSTEM PROCESS | CD79A | 17802 | -0,08733109 | -0,26464897 | TUMOR | Smokers |
| GSE50081 | REGULATION OF IMMUNE SYSTEM PROCESS | CD79A | 16807 | -0,10130733 | -0,46347362 | TUMOR | Smokers |
| GSE47115 | REGULATION OF IMMUNE SYSTEM PROCESS | CD79A | 14785 | -0,17142305 | -0,38923883 | TUMOR | Smokers |
| GSE10072 | REGULATION OF IMMUNE SYSTEM PROCESS | CD79A | 12235 | -0,45417944 | -0,2225477 | NORMAL | Non-Smokers |
| GSE32863 | REGULATION OF IMMUNE SYSTEM PROCESS | CD79A | 35169 | -0,40712893 | -0,22981593 | TUMOR | Smokers |
| GSE50081 | IMMUNE_RESPONSE | CD79B | 18096 | -0,13326603 | -0,3296996 | TUMOR | Smokers |
| GSE47115 | IMMUNE_RESPONSE | CD79B | 19307 | -0,4243114 | -0,3095248 | TUMOR | Smokers |
| GSE32863 | IMMUNE_RESPONSE | CD79B | 33930 | -0,37371859 | -0,26519004 | TUMOR | Non-Smokers |
| GSE32863 | IMMUNE_RESPONSE | CD79B | 36140 | -0,51437175 | -0,14189133 | TUMOR | Smokers |
| TCGA | IMMUNE_RESPONSE | CD79B | 18928 | -0,10959418 | -0,20287137 | TUMOR | Smokers |
| GSE50081 | IMMUNE SYSTEM PROCESS | CD79B | 18096 | -0,13326603 | -0,2869325 | TUMOR | Smokers |
| GSE47115 | IMMUNE SYSTEM PROCESS | CD79B | 19307 | -0,4243114 | -0,29236358 | TUMOR | Smokers |
| GSE32863 | IMMUNE SYSTEM PROCESS | CD79B | 33930 | -0,37371859 | -0,25272617 | TUMOR | Non-Smokers |
| GSE32863 | IMMUNE SYSTEM PROCESS | CD79B | 36140 | -0,51437175 | -0,108877145 | TUMOR | Smokers |
| TCGA | IMMUNE SYSTEM PROCESS | CD79B | 18928 | -0,10959418 | -0,17707747 | TUMOR | Smokers |
| GSE32863 | IMMUNE_RESPONSE | CD83 | 29795 | -0,2074023 | -0,4110999 | TUMOR | Smokers |
| TCGA | IMMUNE_RESPONSE | CD83 | 20131 | -0,16346909 | -0,02727498 | TUMOR | Smokers |
| GSE32863 | IMMUNE SYSTEM PROCESS | CD83 | 29795 | -0,2074023 | -0,38990253 | TUMOR | Smokers |
| TCGA | IMMUNE SYSTEM PROCESS | CD83 | 20131 | -0,16346909 | -0,016537739 | TUMOR | Smokers |
| GSE47115 | DEFENSE RESPONSE | CD83 | 15100 | -0,18423763 | -0,34505472 | TUMOR | Smokers |
| GSE32863 | DEFENSE RESPONSE | CD83 | 29795 | -0,2074023 | -0,3995754 | TUMOR | Smokers |
| TCGA | DEFENSE RESPONSE | CD83 | 20131 | -0,16346909 | -0,04461871 | TUMOR | Smokers |
| GSE50081 | DEFENSE RESPONSE | CD84 | 19919 | -0,2145929 | -0,22607782 | TUMOR | Smokers |
| GSE47115 | DEFENSE RESPONSE | CD84 | 18118 | -0,32932827 | -0,25440785 | TUMOR | Smokers |
| GSE32863 | DEFENSE RESPONSE | CD84 | 33359 | -0,34807998 | -0,24962194 | TUMOR | Non-Smokers |
| GSE32863 | DEFENSE RESPONSE | CD84 | 36298 | -0,54848295 | -0,10718647 | TUMOR | Smokers |
| TCGA | DEFENSE RESPONSE | CD84 | 15255 | -0,0542567 | -0,40619913 | TUMOR | Smokers |
| GSE50081 | IMMUNE_RESPONSE | CD86 | 18887 | -0,15866326 | -0,2534926 | TUMOR | Smokers |
| GSE32863 | IMMUNE_RESPONSE | CD86 | 27384 | -0,17318203 | -0,38012943 | TUMOR | Non-Smokers |
| GSE32863 | IMMUNE_RESPONSE | CD86 | 28400 | -0,17688274 | -0,44208705 | TUMOR | Smokers |
| TCGA | IMMUNE_RESPONSE | CD86 | 15790 | -0,06015815 | -0,40991375 | TUMOR | Smokers |
| GSE50081 | IMMUNE SYSTEM PROCESS | CD86 | 18887 | -0,15866326 | -0,22677317 | TUMOR | Smokers |
| GSE32863 | IMMUNE SYSTEM PROCESS | CD86 | 28400 | -0,17688274 | -0,41377977 | TUMOR | Smokers |
| TCGA | IMMUNE SYSTEM PROCESS | CD86 | 15790 | -0,06015815 | -0,36015525 | TUMOR | Smokers |
| GSE50081 | IMMUNE_RESPONSE | CD96 | 16562 | -0,09555419 | -0,40820783 | TUMOR | Smokers |
| GSE47115 | IMMUNE_RESPONSE | CD96 | 19217 | -0,41535467 | -0,31268686 | TUMOR | Smokers |
| GSE32863 | IMMUNE_RESPONSE | CD96 | 34719 | -0,41569063 | -0,24224508 | TUMOR | Non-Smokers |
| GSE32863 | IMMUNE_RESPONSE | CD96 | 36593 | -0,69081795 | -0,03762506 | TUMOR | Smokers |
| TCGA | IMMUNE_RESPONSE | CD96 | 17514 | -0,08265004 | -0,30265507 | TUMOR | Smokers |
| GSE50081 | IMMUNE SYSTEM PROCESS | CD96 | 16562 | -0,09555419 | -0,3547748 | TUMOR | Smokers |
| GSE47115 | IMMUNE SYSTEM PROCESS | CD96 | 19217 | -0,41535467 | -0,29869676 | TUMOR | Smokers |
| GSE32863 | IMMUNE SYSTEM PROCESS | CD96 | 34719 | -0,41569063 | -0,23811862 | TUMOR | Non-Smokers |
| GSE32863 | IMMUNE SYSTEM PROCESS | CD96 | 36593 | -0,69081795 | -0,027147561 | TUMOR | Smokers |
| TCGA | IMMUNE SYSTEM PROCESS | CD96 | 17514 | -0,08265004 | -0,2747043 | TUMOR | Smokers |
| GSE32863 | IMMUNE_RESPONSE | CD97 | 33985 | -0,37625292 | -0,2596494 | TUMOR | Non-Smokers |
| GSE32863 | IMMUNE SYSTEM PROCESS | CD97 | 33985 | -0,37625292 | -0,24930361 | TUMOR | Non-Smokers |
| GSE32863 | DEFENSE RESPONSE | CD97 | 33985 | -0,37625292 | -0,22850595 | TUMOR | Non-Smokers |
| GSE50081 | IMMUNE SYSTEM PROCESS | CDC42 | 18395 | -0,14264202 | -0,28868937 | TUMOR | Smokers |
| GSE32863 | IMMUNE SYSTEM PROCESS | CDK6 | 30242 | -0,21831676 | -0,3842678 | TUMOR | Smokers |
| TCGA | DEFENSE RESPONSE | CDO1 | 16056 | -0,06331143 | -0,3976449 | TUMOR | Smokers |
| GSE47115 | IMMUNE_RESPONSE | CEACAM8 | 17292 | -0,28430399 | -0,38776115 | TUMOR | Smokers |
| GSE32863 | IMMUNE_RESPONSE | CEACAM8 | 30258 | -0,24395712 | -0,3388688 | TUMOR | Non-Smokers |
| GSE32863 | IMMUNE_RESPONSE | CEACAM8 | 33911 | -0,33414134 | -0,35695088 | TUMOR | Smokers |
| TCGA | IMMUNE_RESPONSE | CEACAM8 | 16947 | -0,07456211 | -0,36276558 | TUMOR | Smokers |
| TCGA | IMMUNE_RESPONSE | CEACAM8 | 16582 | -0,23322846 | -0,28695545 | TUMOR | Non-Smokers |
| GSE47115 | IMMUNE SYSTEM PROCESS | CEACAM8 | 17292 | -0,28430399 | -0,3625226 | TUMOR | Smokers |
| GSE32863 | IMMUNE SYSTEM PROCESS | CEACAM8 | 30258 | -0,24395712 | -0,35797533 | TUMOR | Non-Smokers |
| GSE32863 | IMMUNE SYSTEM PROCESS | CEACAM8 | 33911 | -0,33414134 | -0,31615743 | TUMOR | Smokers |
| TCGA | IMMUNE SYSTEM PROCESS | CEACAM8 | 16947 | -0,07456211 | -0,32173592 | TUMOR | Smokers |
| TCGA | IMMUNE SYSTEM PROCESS | CEACAM8 | 16582 | -0,23322846 | -0,29785785 | TUMOR | Non-Smokers |
| GSE50081 | IMMUNE_RESPONSE | CEBPB | 15940 | -0,08204624 | -0,41520372 | TUMOR | Smokers |
| TCGA | IMMUNE_RESPONSE | CEBPB | 18515 | -0,33065134 | -0,12089634 | TUMOR | Non-Smokers |
| GSE50081 | IMMUNE SYSTEM PROCESS | CEBPB | 15940 | -0,08204624 | -0,36859098 | TUMOR | Smokers |
| TCGA | IMMUNE SYSTEM PROCESS | CEBPB | 18515 | -0,33065134 | -0,13876337 | TUMOR | Non-Smokers |
| GSE50081 | IMMUNE_RESPONSE | CEBPG | 19753 | -0,20360467 | -0,1920023 | TUMOR | Smokers |
| GSE50081 | IMMUNE SYSTEM PROCESS | CEBPG | 19753 | -0,20360467 | -0,14584973 | TUMOR | Smokers |
| GSE50081 | DEFENSE RESPONSE | CEBPG | 19753 | -0,20360467 | -0,24225053 | TUMOR | Smokers |
| GSE50081 | IMMUNE_RESPONSE | CFHR1 | 20559 | -0,35684597 | 0,002256711 | TUMOR | Smokers |
| GSE47115 | IMMUNE_RESPONSE | CFHR1 | 17117 | -0,27459109 | -0,40922284 | TUMOR | Smokers |
| GSE32863 | IMMUNE_RESPONSE | CFHR1 | 30503 | -0,22485685 | -0,40624246 | TUMOR | Smokers |
| TCGA | IMMUNE_RESPONSE | CFHR1 | 17780 | -0,28481853 | -0,21393295 | TUMOR | Non-Smokers |
| GSE50081 | IMMUNE SYSTEM PROCESS | CFHR1 | 20559 | -0,35684597 | 0,002266484 | TUMOR | Smokers |
| GSE47115 | IMMUNE SYSTEM PROCESS | CFHR1 | 17117 | -0,27459109 | -0,3790926 | TUMOR | Smokers |
| GSE32863 | IMMUNE SYSTEM PROCESS | CFHR1 | 30503 | -0,22485685 | -0,3727852 | TUMOR | Smokers |
| TCGA | IMMUNE SYSTEM PROCESS | CFHR1 | 17780 | -0,28481853 | -0,2303761 | TUMOR | Non-Smokers |
| GSE50081 | DEFENSE RESPONSE | CFHR1 | 20559 | -0,35684597 | -0,03060225 | TUMOR | Smokers |
| GSE47115 | DEFENSE RESPONSE | CFHR1 | 17117 | -0,27459109 | -0,2842947 | TUMOR | Smokers |
| GSE32863 | DEFENSE RESPONSE | CFHR1 | 30503 | -0,22485685 | -0,40083322 | TUMOR | Smokers |
| GSE50081 | REGULATION OF IMMUNE SYSTEM PROCESS | CFHR1 | 20559 | -0,35684597 | 0,002235857 | TUMOR | Smokers |
| GSE47115 | REGULATION OF IMMUNE SYSTEM PROCESS | CFHR1 | 17117 | -0,27459109 | -0,3693405 | TUMOR | Smokers |
| GSE32863 | REGULATION OF IMMUNE SYSTEM PROCESS | CFHR1 | 30503 | -0,22485685 | -0,34480736 | TUMOR | Smokers |
| TCGA | REGULATION OF IMMUNE SYSTEM PROCESS | CFHR1 | 17780 | -0,28481853 | -0,3234777 | TUMOR | Non-Smokers |
| GSE50081 | DEFENSE RESPONSE | CFP | 20256 | -0,2512739 | -0,15211639 | TUMOR | Smokers |
| GSE47115 | DEFENSE RESPONSE | CFP | 19580 | -0,45105222 | -0,18515171 | TUMOR | Smokers |
| GSE32863 | DEFENSE RESPONSE | CFP | 30014 | -0,23712371 | -0,33017793 | TUMOR | Non-Smokers |
| GSE47115 | DEFENSE RESPONSE | CHRNA7 | 15584 | -0,20420989 | -0,3405342 | TUMOR | Smokers |
| GSE32863 | DEFENSE RESPONSE | CHRNA7 | 31308 | -0,27473885 | -0,2957315 | TUMOR | Non-Smokers |
| GSE50081 | DEFENSE RESPONSE | CHST2 | 19831 | -0,20819777 | -0,23006135 | TUMOR | Smokers |
| GSE47115 | DEFENSE RESPONSE | CHST2 | 19987 | -0,5108189 | -0,13909806 | TUMOR | Smokers |
| TCGA | DEFENSE RESPONSE | CHST2 | 17638 | -0,0846406 | -0,3108162 | TUMOR | Smokers |
| GSE47115 | IMMUNE_RESPONSE | CHST4 | 18217 | -0,3347297 | -0,3441206 | TUMOR | Smokers |
| GSE32863 | IMMUNE_RESPONSE | CHST4 | 36089 | -0,55281979 | -0,07832452 | TUMOR | Non-Smokers |
| TCGA | IMMUNE_RESPONSE | CHST4 | 16765 | -0,07232861 | -0,37310442 | TUMOR | Smokers |
| GSE47115 | IMMUNE SYSTEM PROCESS | CHST4 | 18217 | -0,3347297 | -0,33603537 | TUMOR | Smokers |
| GSE32863 | IMMUNE SYSTEM PROCESS | CHST4 | 36089 | -0,55281979 | -0,098705925 | TUMOR | Non-Smokers |
| TCGA | IMMUNE SYSTEM PROCESS | CHST4 | 16765 | -0,07232861 | -0,33032835 | TUMOR | Smokers |
| GSE32863 | IMMUNE_RESPONSE | CHUK | 31909 | -0,29360273 | -0,3044202 | TUMOR | Non-Smokers |
| GSE32863 | IMMUNE SYSTEM PROCESS | CHUK | 31909 | -0,29360273 | -0,31962118 | TUMOR | Non-Smokers |
| GSE47115 | IMMUNE_RESPONSE | CIITA | 19993 | -0,51195925 | -0,20039558 | TUMOR | Smokers |
| GSE32863 | IMMUNE_RESPONSE | CIITA | 34432 | -0,35886228 | -0,32556617 | TUMOR | Smokers |
| TCGA | IMMUNE_RESPONSE | CIITA | 18434 | -0,09870848 | -0,25909775 | TUMOR | Smokers |
| TCGA | IMMUNE_RESPONSE | CIITA | 16318 | -0,22276285 | -0,31338182 | TUMOR | Non-Smokers |
| GSE47115 | IMMUNE SYSTEM PROCESS | CIITA | 19993 | -0,51195925 | -0,1931734 | TUMOR | Smokers |
| GSE32863 | IMMUNE SYSTEM PROCESS | CIITA | 34432 | -0,35886228 | -0,29183912 | TUMOR | Smokers |
| TCGA | IMMUNE SYSTEM PROCESS | CIITA | 18434 | -0,09870848 | -0,21629885 | TUMOR | Smokers |
| TCGA | IMMUNE SYSTEM PROCESS | CIITA | 16318 | -0,22276285 | -0,32427162 | TUMOR | Non-Smokers |
| GSE47115 | IMMUNE SYSTEM PROCESS | CKLF | 19788 | -0,47848228 | -0,24021322 | TUMOR | Smokers |
| GSE32863 | IMMUNE SYSTEM PROCESS | CKLF | 30003 | -0,23666932 | -0,36683786 | TUMOR | Non-Smokers |
| GSE32863 | IMMUNE SYSTEM PROCESS | CKLF | 29289 | -0,19617732 | -0,40151066 | TUMOR | Smokers |
| TCGA | DEFENSE RESPONSE | CLEC1A | 18419 | -0,09849074 | -0,23337375 | TUMOR | Smokers |
| GSE32863 | DEFENSE RESPONSE | CLEC1B | 32063 | -0,29849014 | -0,28148317 | TUMOR | Non-Smokers |
| GSE47115 | CELLULAR DEFENSE RESPONSE | CLEC5A | 15093 | -0,18406697 | -0,38446102 | TUMOR | Smokers |
| GSE32863 | CELLULAR DEFENSE RESPONSE | CLEC5A | 26928 | -0,16353998 | -0,44692 | TUMOR | Non-Smokers |
| TCGA | CELLULAR DEFENSE RESPONSE | CLEC5A | 18640 | -0,10293839 | -0,10913828 | TUMOR | Smokers |
| GSE47115 | DEFENSE RESPONSE | CLEC5A | 15093 | -0,18406697 | -0,34798393 | TUMOR | Smokers |
| TCGA | DEFENSE RESPONSE | CLEC5A | 18640 | -0,10293839 | -0,23207894 | TUMOR | Smokers |
| GSE32863 | IMMUNE SYSTEM PROCESS | CLEC7A | 32987 | -0,33302283 | -0,2877839 | TUMOR | Non-Smokers |
| GSE32863 | IMMUNE SYSTEM PROCESS | CLEC7A | 33233 | -0,30502525 | -0,35604364 | TUMOR | Smokers |
| TCGA | IMMUNE SYSTEM PROCESS | CLEC7A | 14457 | -0,04600554 | -0,37552926 | TUMOR | Smokers |
| GSE50081 | IMMUNE_RESPONSE | CMKLR1 | 20217 | -0,24568997 | -0,10292251 | TUMOR | Smokers |
| GSE32863 | IMMUNE_RESPONSE | CMKLR1 | 30173 | -0,24170695 | -0,3456797 | TUMOR | Non-Smokers |
| TCGA | IMMUNE_RESPONSE | CMKLR1 | 17986 | -0,29814667 | -0,18046165 | TUMOR | Non-Smokers |
| GSE50081 | IMMUNE SYSTEM PROCESS | CMKLR1 | 20217 | -0,24568997 | -0,07932208 | TUMOR | Smokers |
| GSE32863 | IMMUNE SYSTEM PROCESS | CMKLR1 | 30173 | -0,24170695 | -0,36204416 | TUMOR | Non-Smokers |
| TCGA | IMMUNE SYSTEM PROCESS | CMKLR1 | 17986 | -0,29814667 | -0,20512733 | TUMOR | Non-Smokers |
| TCGA | IMMUNE_RESPONSE | CNIH | 15778 | -0,20425688 | -0,34999442 | TUMOR | Non-Smokers |
| TCGA | IMMUNE SYSTEM PROCESS | CNIH | 15778 | -0,20425688 | -0,35274106 | TUMOR | Non-Smokers |
| GSE47115 | IMMUNE_RESPONSE | CNR2 | 20667 | -0,84377474 | -0,013297807 | TUMOR | Smokers |
| TCGA | IMMUNE_RESPONSE | CNR2 | 19819 | -0,1389394 | -0,051882707 | TUMOR | Smokers |
| TCGA | IMMUNE_RESPONSE | CNR2 | 19379 | -0,41867971 | -0,0973052 | TUMOR | Non-Smokers |
| GSE47115 | IMMUNE SYSTEM PROCESS | CNR2 | 20667 | -0,84377474 | -0,00874241 | TUMOR | Smokers |
| TCGA | IMMUNE SYSTEM PROCESS | CNR2 | 19819 | -0,1389394 | -0,051468093 | TUMOR | Smokers |
| TCGA | IMMUNE SYSTEM PROCESS | CNR2 | 19379 | -0,41867971 | -0,08681842 | TUMOR | Non-Smokers |
| TCGA | IMMUNE_RESPONSE | COLEC12 | 16453 | -0,06804028 | -0,38060886 | TUMOR | Smokers |
| TCGA | IMMUNE SYSTEM PROCESS | COLEC12 | 16453 | -0,06804028 | -0,3414414 | TUMOR | Smokers |
| TCGA | DEFENSE RESPONSE | COLEC12 | 16453 | -0,06804028 | -0,37359378 | TUMOR | Smokers |
| GSE47115 | IMMUNE_RESPONSE | CRHR1 | 16873 | -0,26159546 | -0,40690008 | TUMOR | Smokers |
| GSE47115 | IMMUNE SYSTEM PROCESS | CRHR1 | 16873 | -0,26159546 | -0,3740277 | TUMOR | Smokers |
| GSE50081 | DEFENSE RESPONSE | CRP | 18447 | -0,14448521 | -0,37465522 | TUMOR | Smokers |
| GSE32863 | DEFENSE RESPONSE | CRP | 33892 | -0,37217355 | -0,23882775 | TUMOR | Non-Smokers |
| GSE32863 | DEFENSE RESPONSE | CRP | 32321 | -0,27384713 | -0,3474384 | TUMOR | Smokers |
| GSE50081 | IMMUNE_RESPONSE | CRTAM | 18766 | -0,15452202 | -0,30675948 | TUMOR | Smokers |
| GSE47115 | IMMUNE_RESPONSE | CRTAM | 18532 | -0,35555837 | -0,32842016 | TUMOR | Smokers |
| GSE32863 | IMMUNE_RESPONSE | CRTAM | 30999 | -0,2652007 | -0,31148732 | TUMOR | Non-Smokers |
| TCGA | IMMUNE_RESPONSE | CRTAM | 16609 | -0,07012694 | -0,37474868 | TUMOR | Smokers |
| GSE50081 | IMMUNE SYSTEM PROCESS | CRTAM | 18766 | -0,15452202 | -0,27429786 | TUMOR | Smokers |
| GSE47115 | IMMUNE SYSTEM PROCESS | CRTAM | 18532 | -0,35555837 | -0,3203511 | TUMOR | Smokers |
| GSE32863 | IMMUNE SYSTEM PROCESS | CRTAM | 30999 | -0,2652007 | -0,33158258 | TUMOR | Non-Smokers |
| TCGA | IMMUNE SYSTEM PROCESS | CRTAM | 16609 | -0,07012694 | -0,33274055 | TUMOR | Smokers |
| GSE50081 | DEFENSE RESPONSE | CRTAM | 18766 | -0,15452202 | -0,33208135 | TUMOR | Smokers |
| GSE47115 | DEFENSE RESPONSE | CRTAM | 18532 | -0,35555837 | -0,21380498 | TUMOR | Smokers |
| GSE32863 | DEFENSE RESPONSE | CRTAM | 30999 | -0,2652007 | -0,2965741 | TUMOR | Non-Smokers |
| TCGA | DEFENSE RESPONSE | CRTAM | 16609 | -0,07012694 | -0,36447307 | TUMOR | Smokers |
| GSE50081 | REGULATION OF IMMUNE SYSTEM PROCESS | CRTAM | 18766 | -0,15452202 | -0,3688426 | TUMOR | Smokers |
| GSE47115 | REGULATION OF IMMUNE SYSTEM PROCESS | CRTAM | 18532 | -0,35555837 | -0,30355006 | TUMOR | Smokers |
| GSE10072 | REGULATION OF IMMUNE SYSTEM PROCESS | CRTAM | 10778 | -0,30959323 | -0,3988137 | NORMAL | Non-Smokers |
| GSE32863 | IMMUNE SYSTEM PROCESS | CSF1 | 34222 | -0,38827518 | -0,2457098 | TUMOR | Non-Smokers |
| GSE32863 | IMMUNE SYSTEM PROCESS | CSF1 | 30398 | -0,22209185 | -0,37617856 | TUMOR | Smokers |
| TCGA | IMMUNE SYSTEM PROCESS | CSF1 | 14536 | -0,04695043 | -0,3727503 | TUMOR | Smokers |
| GSE50081 | DEFENSE RESPONSE | CSF3R | 19683 | -0,19876087 | -0,26239076 | TUMOR | Smokers |
| GSE32863 | DEFENSE RESPONSE | CSF3R | 33027 | -0,33448058 | -0,2581353 | TUMOR | Non-Smokers |
| GSE32863 | DEFENSE RESPONSE | CSF3R | 36416 | -0,58229816 | -0,091339305 | TUMOR | Smokers |
| GSE32863 | IMMUNE_RESPONSE | CST7 | 27684 | -0,18010727 | -0,371572 | TUMOR | Non-Smokers |
| GSE32863 | IMMUNE_RESPONSE | CST7 | 35507 | -0,43493071 | -0,18829204 | TUMOR | Smokers |
| TCGA | IMMUNE_RESPONSE | CST7 | 15839 | -0,06075013 | -0,40439102 | TUMOR | Smokers |
| GSE32863 | IMMUNE SYSTEM PROCESS | CST7 | 27684 | -0,18010727 | -0,39059395 | TUMOR | Non-Smokers |
| GSE32863 | IMMUNE SYSTEM PROCESS | CST7 | 35507 | -0,43493071 | -0,16558671 | TUMOR | Smokers |
| TCGA | IMMUNE SYSTEM PROCESS | CST7 | 15839 | -0,06075013 | -0,3567957 | TUMOR | Smokers |
| GSE47115 | IMMUNE_RESPONSE | CTLA4 | 19397 | -0,43313608 | -0,29086378 | TUMOR | Smokers |
| GSE32863 | IMMUNE_RESPONSE | CTLA4 | 33567 | -0,35688603 | -0,28957602 | TUMOR | Non-Smokers |
| GSE32863 | IMMUNE_RESPONSE | CTLA4 | 34925 | -0,38924107 | -0,2897357 | TUMOR | Smokers |
| TCGA | IMMUNE_RESPONSE | CTLA4 | 16923 | -0,0742076 | -0,3664516 | TUMOR | Smokers |
| GSE47115 | IMMUNE SYSTEM PROCESS | CTLA4 | 19397 | -0,43313608 | -0,2802134 | TUMOR | Smokers |
| GSE32863 | IMMUNE SYSTEM PROCESS | CTLA4 | 33567 | -0,35688603 | -0,28119972 | TUMOR | Non-Smokers |
| GSE32863 | IMMUNE SYSTEM PROCESS | CTLA4 | 34925 | -0,38924107 | -0,2423134 | TUMOR | Smokers |
| TCGA | IMMUNE SYSTEM PROCESS | CTLA4 | 16923 | -0,0742076 | -0,32764268 | TUMOR | Smokers |
| GSE50081 | IMMUNE_RESPONSE | CTSC | 18850 | -0,15729778 | -0,26497307 | TUMOR | Smokers |
| GSE47115 | IMMUNE_RESPONSE | CTSC | 19076 | -0,40249223 | -0,32058972 | TUMOR | Smokers |
| GSE32863 | IMMUNE_RESPONSE | CTSC | 33585 | -0,35803393 | -0,28336087 | TUMOR | Non-Smokers |
| GSE50081 | IMMUNE SYSTEM PROCESS | CTSC | 18850 | -0,15729778 | -0,23968479 | TUMOR | Smokers |
| GSE47115 | IMMUNE SYSTEM PROCESS | CTSC | 19076 | -0,40249223 | -0,30240688 | TUMOR | Smokers |
| GSE32863 | IMMUNE SYSTEM PROCESS | CTSC | 33585 | -0,35803393 | -0,27699772 | TUMOR | Non-Smokers |
| GSE32863 | IMMUNE SYSTEM PROCESS | CTSE | 29283 | -0,2181714 | -0,3714538 | TUMOR | Non-Smokers |
| TCGA | IMMUNE SYSTEM PROCESS | CTSE | 15350 | -0,19158739 | -0,35866997 | TUMOR | Non-Smokers |
| TCGA | IMMUNE_RESPONSE | CTSG | 17481 | -0,08209326 | -0,3118048 | TUMOR | Smokers |
| TCGA | IMMUNE_RESPONSE | CTSG | 18715 | -0,34574679 | -0,122465186 | TUMOR | Non-Smokers |
| TCGA | IMMUNE SYSTEM PROCESS | CTSG | 17481 | -0,08209326 | -0,28091872 | TUMOR | Smokers |
| TCGA | IMMUNE SYSTEM PROCESS | CTSG | 18715 | -0,34574679 | -0,14296594 | TUMOR | Non-Smokers |
| GSE50081 | IMMUNE_RESPONSE | CTSS | 17818 | -0,12479383 | -0,3542584 | TUMOR | Smokers |
| GSE32863 | IMMUNE_RESPONSE | CTSS | 31665 | -0,28543916 | -0,31408557 | TUMOR | Non-Smokers |
| GSE50081 | IMMUNE SYSTEM PROCESS | CTSS | 17818 | -0,12479383 | -0,31742474 | TUMOR | Smokers |
| GSE32863 | IMMUNE SYSTEM PROCESS | CTSS | 31665 | -0,28543916 | -0,32436946 | TUMOR | Non-Smokers |
| GSE47115 | IMMUNE_RESPONSE | CTSW | 20144 | -0,54744482 | -0,17942795 | TUMOR | Smokers |
| GSE32863 | IMMUNE_RESPONSE | CTSW | 28740 | -0,18402086 | -0,4343734 | TUMOR | Smokers |
| TCGA | IMMUNE_RESPONSE | CTSW | 15612 | -0,05828534 | -0,42832184 | TUMOR | Smokers |
| GSE47115 | IMMUNE SYSTEM PROCESS | CTSW | 20144 | -0,54744482 | -0,18022741 | TUMOR | Smokers |
| GSE32863 | IMMUNE SYSTEM PROCESS | CTSW | 28740 | -0,18402086 | -0,40790564 | TUMOR | Smokers |
| TCGA | IMMUNE SYSTEM PROCESS | CTSW | 15612 | -0,05828534 | -0,37396836 | TUMOR | Smokers |
| GSE50081 | IMMUNE_RESPONSE | CX3CL1 | 20107 | -0,23036355 | -0,12788282 | TUMOR | Smokers |
| GSE32863 | IMMUNE_RESPONSE | CX3CL1 | 31243 | -0,2430314 | -0,4048518 | TUMOR | Smokers |
| TCGA | IMMUNE_RESPONSE | CX3CL1 | 20337 | -0,21613264 | 0,001638514 | TUMOR | Smokers |
| TCGA | IMMUNE_RESPONSE | CX3CL1 | 16340 | -0,22356114 | -0,30333373 | TUMOR | Non-Smokers |
| GSE50081 | IMMUNE SYSTEM PROCESS | CX3CL1 | 20107 | -0,23036355 | -0,09629854 | TUMOR | Smokers |
| GSE32863 | IMMUNE SYSTEM PROCESS | CX3CL1 | 31243 | -0,2430314 | -0,37701002 | TUMOR | Smokers |
| TCGA | IMMUNE SYSTEM PROCESS | CX3CL1 | 20337 | -0,21613264 | 0,001646535 | TUMOR | Smokers |
| TCGA | IMMUNE SYSTEM PROCESS | CX3CL1 | 16340 | -0,22356114 | -0,3175699 | TUMOR | Non-Smokers |
| GSE50081 | DEFENSE RESPONSE | CX3CL1 | 20107 | -0,23036355 | -0,19207844 | TUMOR | Smokers |
| GSE32863 | DEFENSE RESPONSE | CX3CL1 | 31243 | -0,2430314 | -0,3825987 | TUMOR | Smokers |
| TCGA | DEFENSE RESPONSE | CX3CL1 | 20337 | -0,21613264 | 0,001641251 | TUMOR | Smokers |
| TCGA | CELLULAR DEFENSE RESPONSE | CX3CR1 | 17139 | -0,07730263 | -0,278617 | TUMOR | Smokers |
| TCGA | DEFENSE RESPONSE | CX3CR1 | 17139 | -0,07730263 | -0,345559 | TUMOR | Smokers |
| GSE50081 | DEFENSE RESPONSE | CXCL1 | 19164 | -0,17111731 | -0,30863348 | TUMOR | Smokers |
| GSE32863 | DEFENSE RESPONSE | CXCL1 | 30414 | -0,24845429 | -0,3115794 | TUMOR | Non-Smokers |
| GSE32863 | DEFENSE RESPONSE | CXCL1 | 36429 | -0,5869258 | -0,08199167 | TUMOR | Smokers |
| GSE50081 | DEFENSE RESPONSE | CXCL10 | 20310 | -0,25882417 | -0,12491925 | TUMOR | Smokers |
| GSE47115 | DEFENSE RESPONSE | CXCL10 | 19858 | -0,48807684 | -0,14177631 | TUMOR | Smokers |
| GSE32863 | DEFENSE RESPONSE | CXCL10 | 29989 | -0,23622508 | -0,33358064 | TUMOR | Non-Smokers |
| GSE32863 | DEFENSE RESPONSE | CXCL10 | 31517 | -0,25078082 | -0,36950913 | TUMOR | Smokers |
| TCGA | DEFENSE RESPONSE | CXCL10 | 16167 | -0,06460553 | -0,3796217 | TUMOR | Smokers |
| GSE50081 | DEFENSE RESPONSE | CXCL11 | 20465 | -0,29458374 | -0,0774386 | TUMOR | Smokers |
| GSE47115 | DEFENSE RESPONSE | CXCL11 | 19599 | -0,45270434 | -0,17015 | TUMOR | Smokers |
| GSE32863 | DEFENSE RESPONSE | CXCL11 | 34312 | -0,3928048 | -0,21104369 | TUMOR | Non-Smokers |
| TCGA | DEFENSE RESPONSE | CXCL11 | 18685 | -0,10396319 | -0,22171411 | TUMOR | Smokers |
| GSE32863 | IMMUNE_RESPONSE | CXCL12 | 35721 | -0,5000214 | -0,17673187 | TUMOR | Non-Smokers |
| GSE32863 | IMMUNE_RESPONSE | CXCL12 | 29592 | -0,20259963 | -0,42109272 | TUMOR | Smokers |
| TCGA | IMMUNE_RESPONSE | CXCL12 | 18936 | -0,10980236 | -0,19610901 | TUMOR | Smokers |
| GSE32863 | IMMUNE SYSTEM PROCESS | CXCL12 | 35721 | -0,5000214 | -0,16450663 | TUMOR | Non-Smokers |
| GSE32863 | IMMUNE SYSTEM PROCESS | CXCL12 | 29592 | -0,20259963 | -0,39589092 | TUMOR | Smokers |
| TCGA | IMMUNE SYSTEM PROCESS | CXCL12 | 18936 | -0,10980236 | -0,17226438 | TUMOR | Smokers |
| GSE50081 | IMMUNE_RESPONSE | CXCL13 | 20432 | -0,2851465 | -0,04636223 | TUMOR | Smokers |
| GSE47115 | IMMUNE_RESPONSE | CXCL13 | 19989 | -0,51106018 | -0,20930444 | TUMOR | Smokers |
| GSE32863 | IMMUNE_RESPONSE | CXCL13 | 35184 | -0,45018047 | -0,20654291 | TUMOR | Non-Smokers |
| GSE32863 | IMMUNE_RESPONSE | CXCL13 | 36638 | -0,73338652 | 0,001754728 | TUMOR | Smokers |
| TCGA | IMMUNE_RESPONSE | CXCL13 | 19619 | -0,12984692 | -0,077180795 | TUMOR | Smokers |
| GSE50081 | IMMUNE SYSTEM PROCESS | CXCL13 | 20432 | -0,2851465 | -0,03192357 | TUMOR | Smokers |
| GSE47115 | IMMUNE SYSTEM PROCESS | CXCL13 | 19989 | -0,51106018 | -0,1995154 | TUMOR | Smokers |
| GSE32863 | IMMUNE SYSTEM PROCESS | CXCL13 | 35184 | -0,45018047 | -0,19971228 | TUMOR | Non-Smokers |
| GSE32863 | IMMUNE SYSTEM PROCESS | CXCL13 | 36638 | -0,73338652 | 0,001759515 | TUMOR | Smokers |
| TCGA | IMMUNE SYSTEM PROCESS | CXCL13 | 19619 | -0,12984692 | -0,06712419 | TUMOR | Smokers |
| GSE32863 | DEFENSE RESPONSE | CXCL2 | 34135 | -0,3833181 | -0,21294145 | TUMOR | Non-Smokers |
| GSE32863 | DEFENSE RESPONSE | CXCL2 | 30538 | -0,22569712 | -0,3980451 | TUMOR | Smokers |
| TCGA | DEFENSE RESPONSE | CXCL2 | 17746 | -0,0864196 | -0,30055547 | TUMOR | Smokers |
| GSE50081 | DEFENSE RESPONSE | CXCL6 | 20456 | -0,29201379 | -0,099665105 | TUMOR | Smokers |
| GSE32863 | DEFENSE RESPONSE | CXCL6 | 33004 | -0,33377385 | -0,26900804 | TUMOR | Non-Smokers |
| GSE32863 | DEFENSE RESPONSE | CXCL6 | 29090 | -0,19157007 | -0,420031 | TUMOR | Smokers |
| TCGA | DEFENSE RESPONSE | CXCL6 | 18871 | -0,10820051 | -0,19206217 | TUMOR | Smokers |
| GSE50081 | CELLULAR DEFENSE RESPONSE | CXCL9 | 17591 | -0,11955754 | -0,37834662 | TUMOR | Smokers |
| GSE47115 | CELLULAR DEFENSE RESPONSE | CXCL9 | 19713 | -0,46755695 | -0,16350996 | TUMOR | Smokers |
| GSE32863 | CELLULAR DEFENSE RESPONSE | CXCL9 | 34649 | -0,41118851 | -0,18572205 | TUMOR | Non-Smokers |
| GSE32863 | CELLULAR DEFENSE RESPONSE | CXCL9 | 32995 | -0,29610017 | -0,4163544 | TUMOR | Smokers |
| TCGA | CELLULAR DEFENSE RESPONSE | CXCL9 | 17811 | -0,08746977 | -0,15652576 | TUMOR | Smokers |
| GSE50081 | DEFENSE RESPONSE | CXCL9 | 17591 | -0,11955754 | -0,4136512 | TUMOR | Smokers |
| GSE47115 | DEFENSE RESPONSE | CXCL9 | 19713 | -0,46755695 | -0,14327471 | TUMOR | Smokers |
| GSE32863 | DEFENSE RESPONSE | CXCL9 | 34649 | -0,41118851 | -0,1647958 | TUMOR | Non-Smokers |
| GSE32863 | DEFENSE RESPONSE | CXCL9 | 32995 | -0,29610017 | -0,33250543 | TUMOR | Smokers |
| TCGA | DEFENSE RESPONSE | CXCL9 | 17811 | -0,08746977 | -0,28785348 | TUMOR | Smokers |
| TCGA | IMMUNE SYSTEM PROCESS | CXCR2 | 19335 | -0,41105431 | -0,091855556 | TUMOR | Non-Smokers |
| GSE32863 | IMMUNE_RESPONSE | CXCR4 | 29787 | -0,231281 | -0,35729206 | TUMOR | Non-Smokers |
| GSE32863 | IMMUNE_RESPONSE | CXCR4 | 33878 | -0,33291805 | -0,36233136 | TUMOR | Smokers |
| TCGA | IMMUNE_RESPONSE | CXCR4 | 17771 | -0,08685505 | -0,2987251 | TUMOR | Smokers |
| TCGA | IMMUNE_RESPONSE | CXCR4 | 15929 | -0,20903829 | -0,33706132 | TUMOR | Non-Smokers |
| GSE32863 | IMMUNE SYSTEM PROCESS | CXCR4 | 29787 | -0,231281 | -0,3762839 | TUMOR | Non-Smokers |
| GSE32863 | IMMUNE SYSTEM PROCESS | CXCR4 | 33878 | -0,33291805 | -0,3199192 | TUMOR | Smokers |
| TCGA | IMMUNE SYSTEM PROCESS | CXCR4 | 17771 | -0,08685505 | -0,2672581 | TUMOR | Smokers |
| TCGA | IMMUNE SYSTEM PROCESS | CXCR4 | 15929 | -0,20903829 | -0,3424017 | TUMOR | Non-Smokers |
| GSE32863 | DEFENSE RESPONSE | CXCR4 | 29787 | -0,231281 | -0,340199 | TUMOR | Non-Smokers |
| GSE32863 | DEFENSE RESPONSE | CXCR4 | 33878 | -0,33291805 | -0,3201021 | TUMOR | Smokers |
| TCGA | DEFENSE RESPONSE | CXCR4 | 17771 | -0,08685505 | -0,2965027 | TUMOR | Smokers |
| GSE47115 | DEFENSE RESPONSE | CYBB | 14913 | -0,17688189 | -0,35198286 | TUMOR | Smokers |
| GSE32863 | DEFENSE RESPONSE | CYBB | 33008 | -0,33397987 | -0,26337013 | TUMOR | Non-Smokers |
| GSE32863 | DEFENSE RESPONSE | CYBB | 32909 | -0,29295409 | -0,33505476 | TUMOR | Smokers |
| TCGA | DEFENSE RESPONSE | CYBB | 14495 | -0,04632651 | -0,42118293 | TUMOR | Smokers |
| GSE32863 | DEFENSE RESPONSE | CYSLTR1 | 30666 | -0,22880131 | -0,39021742 | TUMOR | Smokers |
| TCGA | DEFENSE RESPONSE | CYSLTR1 | 19507 | -0,12564407 | -0,14514866 | TUMOR | Smokers |
| GSE32863 | IMMUNE_RESPONSE | DEFA1 | 35019 | -0,3953858 | -0,27747974 | TUMOR | Smokers |
| GSE32863 | IMMUNE SYSTEM PROCESS | DEFA1 | 35019 | -0,3953858 | -0,23388124 | TUMOR | Smokers |
| GSE32863 | IMMUNE_RESPONSE | DEFB1 | 35964 | -0,53280115 | -0,10583563 | TUMOR | Non-Smokers |
| TCGA | IMMUNE_RESPONSE | DEFB1 | 16951 | -0,24704033 | -0,28195715 | TUMOR | Non-Smokers |
| GSE32863 | IMMUNE SYSTEM PROCESS | DEFB1 | 35964 | -0,53280115 | -0,11691661 | TUMOR | Non-Smokers |
| TCGA | IMMUNE SYSTEM PROCESS | DEFB1 | 16951 | -0,24704033 | -0,28331053 | TUMOR | Non-Smokers |
| GSE32863 | DEFENSE RESPONSE | DEFB1 | 35964 | -0,53280115 | -0,049042076 | TUMOR | Non-Smokers |
| GSE32863 | IMMUNE_RESPONSE | DEFB103A | 28855 | -0,20777474 | -0,35635486 | TUMOR | Non-Smokers |
| GSE32863 | IMMUNE SYSTEM PROCESS | DEFB103A | 28855 | -0,20777474 | -0,3766373 | TUMOR | Non-Smokers |
| GSE32863 | DEFENSE RESPONSE | DEFB103A | 28855 | -0,20777474 | -0,35990337 | TUMOR | Non-Smokers |
| GSE50081 | IMMUNE_RESPONSE | DEFB127 | 19248 | -0,17504613 | -0,2220704 | TUMOR | Smokers |
| GSE50081 | IMMUNE SYSTEM PROCESS | DEFB127 | 19248 | -0,17504613 | -0,16696233 | TUMOR | Smokers |
| GSE50081 | DEFENSE RESPONSE | DEFB127 | 19248 | -0,17504613 | -0,29267853 | TUMOR | Smokers |
| TCGA | IMMUNE_RESPONSE | DEFB4A | 18524 | -0,10068384 | -0,25699753 | TUMOR | Smokers |
| TCGA | IMMUNE SYSTEM PROCESS | DEFB4A | 18524 | -0,10068384 | -0,21600546 | TUMOR | Smokers |
| TCGA | IMMUNE_RESPONSE | DMBT1 | 19411 | -0,12264493 | -0,091620155 | TUMOR | Smokers |
| TCGA | IMMUNE_RESPONSE | DMBT1 | 19799 | -0,54794008 | -0,08129345 | TUMOR | Non-Smokers |
| TCGA | IMMUNE SYSTEM PROCESS | DMBT1 | 19411 | -0,12264493 | -0,07477154 | TUMOR | Smokers |
| TCGA | IMMUNE SYSTEM PROCESS | DMBT1 | 19799 | -0,54794008 | -0,066462114 | TUMOR | Non-Smokers |
| TCGA | DEFENSE RESPONSE | DMBT1 | 19411 | -0,12264493 | -0,15559584 | TUMOR | Smokers |
| GSE50081 | IMMUNE SYSTEM PROCESS | DOCK2 | 18942 | -0,16108796 | -0,2195118 | TUMOR | Smokers |
| GSE32863 | IMMUNE SYSTEM PROCESS | DOCK2 | 31393 | -0,24735954 | -0,37424296 | TUMOR | Smokers |
| TCGA | IMMUNE SYSTEM PROCESS | DOCK2 | 14489 | -0,04628406 | -0,37489983 | TUMOR | Smokers |
| TCGA | IMMUNE SYSTEM PROCESS | DOCK2 | 19862 | -0,62694734 | -0,048780095 | TUMOR | Non-Smokers |
| TCGA | IMMUNE_RESPONSE | DPP4 | 19547 | -0,12720424 | -0,082063176 | TUMOR | Smokers |
| TCGA | IMMUNE SYSTEM PROCESS | DPP4 | 19547 | -0,12720424 | -0,069686696 | TUMOR | Smokers |
| GSE50081 | IMMUNE_RESPONSE | EBI3 | 17542 | -0,11818061 | -0,38192996 | TUMOR | Smokers |
| GSE32863 | IMMUNE_RESPONSE | EBI3 | 35861 | -0,51674151 | -0,14237995 | TUMOR | Non-Smokers |
| GSE32863 | IMMUNE_RESPONSE | EBI3 | 30680 | -0,22898385 | -0,39399385 | TUMOR | Smokers |
| TCGA | IMMUNE_RESPONSE | EBI3 | 18689 | -0,10401662 | -0,2050904 | TUMOR | Smokers |
| TCGA | IMMUNE_RESPONSE | EBI3 | 18206 | -0,31035295 | -0,15338883 | TUMOR | Non-Smokers |
| GSE50081 | IMMUNE SYSTEM PROCESS | EBI3 | 17542 | -0,11818061 | -0,3380349 | TUMOR | Smokers |
| GSE32863 | IMMUNE SYSTEM PROCESS | EBI3 | 35861 | -0,51674151 | -0,14163174 | TUMOR | Non-Smokers |
| GSE32863 | IMMUNE SYSTEM PROCESS | EBI3 | 30680 | -0,22898385 | -0,36493778 | TUMOR | Smokers |
| TCGA | IMMUNE SYSTEM PROCESS | EBI3 | 18689 | -0,10401662 | -0,18047583 | TUMOR | Smokers |
| TCGA | IMMUNE SYSTEM PROCESS | EBI3 | 18206 | -0,31035295 | -0,17892538 | TUMOR | Non-Smokers |
| GSE47115 | REGULATION OF IMMUNE SYSTEM PROCESS | EBI3 | 15533 | -0,20214912 | -0,39093286 | TUMOR | Smokers |
| GSE10072 | REGULATION OF IMMUNE SYSTEM PROCESS | EBI3 | 13030 | -0,63567775 | -0,08183444 | NORMAL | Non-Smokers |
| GSE32863 | REGULATION OF IMMUNE SYSTEM PROCESS | EBI3 | 30680 | -0,22898385 | -0,32070744 | TUMOR | Smokers |
| TCGA | REGULATION OF IMMUNE SYSTEM PROCESS | EBI3 | 18206 | -0,31035295 | -0,17215979 | TUMOR | Non-Smokers |
| TCGA | IMMUNE SYSTEM PROCESS | ELF4 | 16599 | -0,23372574 | -0,2946365 | TUMOR | Non-Smokers |
| GSE32863 | IMMUNE SYSTEM PROCESS | ERAP2 | 35514 | -0,4794611 | -0,17183769 | TUMOR | Non-Smokers |
| TCGA | IMMUNE SYSTEM PROCESS | ERAP2 | 19369 | -0,12135401 | -0,08425838 | TUMOR | Smokers |
| GSE32863 | IMMUNE_RESPONSE | EREG | 29026 | -0,21194814 | -0,3530933 | TUMOR | Non-Smokers |
| TCGA | IMMUNE_RESPONSE | EREG | 15915 | -0,06172859 | -0,40010893 | TUMOR | Smokers |
| TCGA | IMMUNE_RESPONSE | EREG | 14927 | -0,17869386 | -0,36914328 | TUMOR | Non-Smokers |
| GSE32863 | IMMUNE SYSTEM PROCESS | EREG | 29026 | -0,21194814 | -0,37576494 | TUMOR | Non-Smokers |
| TCGA | IMMUNE SYSTEM PROCESS | EREG | 15915 | -0,06172859 | -0,35471058 | TUMOR | Smokers |
| TCGA | IMMUNE SYSTEM PROCESS | EREG | 14927 | -0,17869386 | -0,36298794 | TUMOR | Non-Smokers |
| GSE47115 | DEFENSE RESPONSE | EREG | 15837 | -0,21497288 | -0,33795622 | TUMOR | Smokers |
| GSE32863 | DEFENSE RESPONSE | EREG | 29026 | -0,21194814 | -0,35007614 | TUMOR | Non-Smokers |
| TCGA | DEFENSE RESPONSE | EREG | 15915 | -0,06172859 | -0,40603715 | TUMOR | Smokers |
| GSE47115 | REGULATION OF IMMUNE SYSTEM PROCESS | EREG | 15837 | -0,21497288 | -0,368043 | TUMOR | Smokers |
| GSE10072 | REGULATION OF IMMUNE SYSTEM PROCESS | EREG | 11023 | -0,32635924 | -0,37030154 | NORMAL | Non-Smokers |
| TCGA | REGULATION OF IMMUNE SYSTEM PROCESS | EREG | 14927 | -0,17869386 | -0,40992993 | TUMOR | Non-Smokers |
| GSE32863 | IMMUNE_RESPONSE | ETS1 | 36086 | -0,55233228 | -0,08858586 | TUMOR | Non-Smokers |
| GSE32863 | IMMUNE_RESPONSE | ETS1 | 34017 | -0,33914834 | -0,347113 | TUMOR | Smokers |
| TCGA | IMMUNE_RESPONSE | ETS1 | 17109 | -0,07697114 | -0,34589154 | TUMOR | Smokers |
| TCGA | IMMUNE_RESPONSE | ETS1 | 17310 | -0,26149192 | -0,23094542 | TUMOR | Non-Smokers |
| GSE32863 | IMMUNE SYSTEM PROCESS | ETS1 | 36086 | -0,55233228 | -0,10586051 | TUMOR | Non-Smokers |
| GSE32863 | IMMUNE SYSTEM PROCESS | ETS1 | 34017 | -0,33914834 | -0,30488807 | TUMOR | Smokers |
| TCGA | IMMUNE SYSTEM PROCESS | ETS1 | 17109 | -0,07697114 | -0,30436647 | TUMOR | Smokers |
| TCGA | IMMUNE SYSTEM PROCESS | ETS1 | 17310 | -0,26149192 | -0,23980822 | TUMOR | Non-Smokers |
| GSE50081 | CELLULAR DEFENSE RESPONSE | FAIM3 | 18415 | -0,14335008 | -0,29912174 | TUMOR | Smokers |
| GSE47115 | CELLULAR DEFENSE RESPONSE | FAIM3 | 17343 | -0,2869834 | -0,29655057 | TUMOR | Smokers |
| GSE32863 | CELLULAR DEFENSE RESPONSE | FAIM3 | 34035 | -0,37835491 | -0,2676532 | TUMOR | Non-Smokers |
| GSE32863 | CELLULAR DEFENSE RESPONSE | FAIM3 | 36677 | -0,81305879 | 0,000682275 | TUMOR | Smokers |
| TCGA | CELLULAR DEFENSE RESPONSE | FAIM3 | 19508 | -0,1256457 | -0,002652754 | TUMOR | Smokers |
| GSE50081 | DEFENSE RESPONSE | FAIM3 | 18415 | -0,14335008 | -0,37869152 | TUMOR | Smokers |
| GSE47115 | DEFENSE RESPONSE | FAIM3 | 17343 | -0,2869834 | -0,26554388 | TUMOR | Smokers |
| GSE32863 | DEFENSE RESPONSE | FAIM3 | 34035 | -0,37835491 | -0,22337039 | TUMOR | Non-Smokers |
| GSE32863 | DEFENSE RESPONSE | FAIM3 | 36677 | -0,81305879 | 6,86E-04 | TUMOR | Smokers |
| TCGA | DEFENSE RESPONSE | FAIM3 | 19508 | -0,1256457 | -0,13755894 | TUMOR | Smokers |
| GSE50081 | IMMUNE_RESPONSE | FCAR | 17303 | -0,11241621 | -0,4137423 | TUMOR | Smokers |
| GSE32863 | IMMUNE_RESPONSE | FCAR | 31141 | -0,26988062 | -0,31031716 | TUMOR | Non-Smokers |
| TCGA | IMMUNE_RESPONSE | FCAR | 19179 | -0,39180332 | -0,10755297 | TUMOR | Non-Smokers |
| GSE50081 | IMMUNE SYSTEM PROCESS | FCAR | 17303 | -0,11241621 | -0,36206597 | TUMOR | Smokers |
| GSE32863 | IMMUNE SYSTEM PROCESS | FCAR | 31141 | -0,26988062 | -0,33193868 | TUMOR | Non-Smokers |
| TCGA | IMMUNE SYSTEM PROCESS | FCAR | 19179 | -0,39180332 | -0,13311967 | TUMOR | Non-Smokers |
| GSE50081 | IMMUNE_RESPONSE | FCGR1A | 17846 | -0,12545162 | -0,3503565 | TUMOR | Smokers |
| GSE47115 | IMMUNE_RESPONSE | FCGR1A | 20406 | -0,62106955 | -0,117679186 | TUMOR | Smokers |
| GSE32863 | IMMUNE_RESPONSE | FCGR1A | 35437 | -0,42849267 | -0,20260467 | TUMOR | Smokers |
| TCGA | IMMUNE_RESPONSE | FCGR1A | 14890 | -0,17775774 | -0,37173533 | TUMOR | Non-Smokers |
| GSE50081 | IMMUNE SYSTEM PROCESS | FCGR1A | 17846 | -0,12545162 | -0,3110188 | TUMOR | Smokers |
| GSE47115 | IMMUNE SYSTEM PROCESS | FCGR1A | 20406 | -0,62106955 | -0,12429281 | TUMOR | Smokers |
| GSE32863 | IMMUNE SYSTEM PROCESS | FCGR1A | 35437 | -0,42849267 | -0,1757159 | TUMOR | Smokers |
| TCGA | IMMUNE SYSTEM PROCESS | FCGR1A | 14890 | -0,17775774 | -0,36423516 | TUMOR | Non-Smokers |
| GSE50081 | IMMUNE_RESPONSE | FCGR2B | 18111 | -0,13361736 | -0,3248198 | TUMOR | Smokers |
| GSE32863 | IMMUNE_RESPONSE | FCGR2B | 36339 | -0,55692226 | -0,13689011 | TUMOR | Smokers |
| TCGA | IMMUNE_RESPONSE | FCGR2B | 18665 | -0,10348648 | -0,21068364 | TUMOR | Smokers |
| TCGA | IMMUNE_RESPONSE | FCGR2B | 16237 | -0,22014104 | -0,32036957 | TUMOR | Non-Smokers |
| GSE50081 | IMMUNE SYSTEM PROCESS | FCGR2B | 18111 | -0,13361736 | -0,28352264 | TUMOR | Smokers |
| GSE32863 | IMMUNE SYSTEM PROCESS | FCGR2B | 36339 | -0,55692226 | -0,09901898 | TUMOR | Smokers |
| TCGA | IMMUNE SYSTEM PROCESS | FCGR2B | 18665 | -0,10348648 | -0,18421867 | TUMOR | Smokers |
| TCGA | IMMUNE SYSTEM PROCESS | FCGR2B | 16237 | -0,22014104 | -0,3279158 | TUMOR | Non-Smokers |
| GSE32863 | IMMUNE_RESPONSE | FCGR3A | 36547 | -0,64503628 | -0,07434747 | TUMOR | Smokers |
| GSE32863 | IMMUNE SYSTEM PROCESS | FCGR3A | 36547 | -0,64503628 | -0,054076552 | TUMOR | Smokers |
| GSE50081 | IMMUNE_RESPONSE | FCGR3B | 17999 | -0,12977071 | -0,34172982 | TUMOR | Smokers |
| GSE47115 | IMMUNE_RESPONSE | FCGR3B | 17199 | -0,27920219 | -0,39837286 | TUMOR | Smokers |
| GSE32863 | IMMUNE_RESPONSE | FCGR3B | 29952 | -0,21123119 | -0,40353698 | TUMOR | Smokers |
| TCGA | IMMUNE_RESPONSE | FCGR3B | 15050 | -0,18191288 | -0,36631662 | TUMOR | Non-Smokers |
| GSE50081 | IMMUNE SYSTEM PROCESS | FCGR3B | 17999 | -0,12977071 | -0,29865125 | TUMOR | Smokers |
| GSE47115 | IMMUNE SYSTEM PROCESS | FCGR3B | 17199 | -0,27920219 | -0,36886242 | TUMOR | Smokers |
| GSE32863 | IMMUNE SYSTEM PROCESS | FCGR3B | 29952 | -0,21123119 | -0,3853945 | TUMOR | Smokers |
| TCGA | IMMUNE SYSTEM PROCESS | FCGR3B | 15050 | -0,18191288 | -0,3597454 | TUMOR | Non-Smokers |
| GSE32863 | IMMUNE_RESPONSE | FCGRT | 30015 | -0,2124725 | -0,40125772 | TUMOR | Smokers |
| TCGA | IMMUNE_RESPONSE | FCGRT | 16528 | -0,23115028 | -0,29003245 | TUMOR | Non-Smokers |
| GSE32863 | IMMUNE SYSTEM PROCESS | FCGRT | 30015 | -0,2124725 | -0,38414738 | TUMOR | Smokers |
| TCGA | IMMUNE SYSTEM PROCESS | FCGRT | 16528 | -0,23115028 | -0,29917815 | TUMOR | Non-Smokers |
| GSE50081 | IMMUNE_RESPONSE | FCN1 | 19221 | -0,17380787 | -0,22808746 | TUMOR | Smokers |
| GSE32863 | IMMUNE_RESPONSE | FCN1 | 33638 | -0,36085641 | -0,27805272 | TUMOR | Non-Smokers |
| GSE32863 | IMMUNE_RESPONSE | FCN1 | 33854 | -0,33186746 | -0,3679357 | TUMOR | Smokers |
| GSE50081 | IMMUNE SYSTEM PROCESS | FCN1 | 19221 | -0,17380787 | -0,17643724 | TUMOR | Smokers |
| GSE32863 | IMMUNE SYSTEM PROCESS | FCN1 | 33638 | -0,36085641 | -0,26902005 | TUMOR | Non-Smokers |
| GSE32863 | IMMUNE SYSTEM PROCESS | FCN1 | 33854 | -0,33186746 | -0,3239114 | TUMOR | Smokers |
| GSE32863 | IMMUNE_RESPONSE | FCN2 | 28458 | -0,19834036 | -0,35706294 | TUMOR | Non-Smokers |
| TCGA | IMMUNE_RESPONSE | FCN2 | 15721 | -0,20233771 | -0,35220137 | TUMOR | Non-Smokers |
| GSE32863 | IMMUNE SYSTEM PROCESS | FCN2 | 28458 | -0,19834036 | -0,37921843 | TUMOR | Non-Smokers |
| TCGA | IMMUNE SYSTEM PROCESS | FCN2 | 15721 | -0,20233771 | -0,35340717 | TUMOR | Non-Smokers |
| GSE47115 | CELLULAR DEFENSE RESPONSE | FOSL1 | 17041 | -0,27007872 | -0,34466425 | TUMOR | Smokers |
| GSE32863 | CELLULAR DEFENSE RESPONSE | FOSL1 | 34594 | -0,40776196 | -0,21742782 | TUMOR | Non-Smokers |
| GSE32863 | CELLULAR DEFENSE RESPONSE | FOSL1 | 33220 | -0,30450413 | -0,40113008 | TUMOR | Smokers |
| TCGA | CELLULAR DEFENSE RESPONSE | FOSL1 | 14569 | -0,04728473 | -0,40028462 | TUMOR | Smokers |
| GSE47115 | DEFENSE RESPONSE | FOSL1 | 17041 | -0,27007872 | -0,30465737 | TUMOR | Smokers |
| GSE32863 | DEFENSE RESPONSE | FOSL1 | 34594 | -0,40776196 | -0,17035651 | TUMOR | Non-Smokers |
| GSE32863 | DEFENSE RESPONSE | FOSL1 | 33220 | -0,30450413 | -0,32866475 | TUMOR | Smokers |
| TCGA | DEFENSE RESPONSE | FOSL1 | 14569 | -0,04728473 | -0,41621065 | TUMOR | Smokers |
| GSE47115 | DEFENSE RESPONSE | FOXN1 | 16804 | -0,25836557 | -0,3259261 | TUMOR | Smokers |
| GSE32863 | IMMUNE SYSTEM PROCESS | FOXO3 | 30643 | -0,25524449 | -0,3357197 | TUMOR | Non-Smokers |
| TCGA | IMMUNE SYSTEM PROCESS | FOXO3 | 14973 | -0,05141446 | -0,37592584 | TUMOR | Smokers |
| GSE50081 | IMMUNE_RESPONSE | FOXP3 | 17400 | -0,11486582 | -0,38974437 | TUMOR | Smokers |
| GSE47115 | IMMUNE_RESPONSE | FOXP3 | 19753 | -0,47197708 | -0,2678684 | TUMOR | Smokers |
| TCGA | IMMUNE_RESPONSE | FOXP3 | 15645 | -0,05862542 | -0,42611468 | TUMOR | Smokers |
| GSE50081 | IMMUNE SYSTEM PROCESS | FOXP3 | 17400 | -0,11486582 | -0,345588 | TUMOR | Smokers |
| GSE47115 | IMMUNE SYSTEM PROCESS | FOXP3 | 19753 | -0,47197708 | -0,25680476 | TUMOR | Smokers |
| TCGA | IMMUNE SYSTEM PROCESS | FOXP3 | 15645 | -0,05862542 | -0,3728086 | TUMOR | Smokers |
| GSE50081 | REGULATION OF IMMUNE SYSTEM PROCESS | FOXP3 | 17400 | -0,11486582 | -0,42684954 | TUMOR | Smokers |
| GSE47115 | REGULATION OF IMMUNE SYSTEM PROCESS | FOXP3 | 19753 | -0,47197708 | -0,25850946 | TUMOR | Smokers |
| GSE50081 | IMMUNE_RESPONSE | FYB | 19910 | -0,21414046 | -0,15604843 | TUMOR | Smokers |
| GSE32863 | IMMUNE_RESPONSE | FYB | 36551 | -0,70195127 | -0,011267264 | TUMOR | Non-Smokers |
| GSE50081 | IMMUNE SYSTEM PROCESS | FYB | 19910 | -0,21414046 | -0,12136818 | TUMOR | Smokers |
| GSE32863 | IMMUNE SYSTEM PROCESS | FYB | 36551 | -0,70195127 | -0,031005705 | TUMOR | Non-Smokers |
| TCGA | IMMUNE SYSTEM PROCESS | FYB | 14447 | -0,04589283 | -0,3772432 | TUMOR | Smokers |
| GSE32863 | IMMUNE_RESPONSE | FYN | 33906 | -0,37266228 | -0,27153337 | TUMOR | Non-Smokers |
| TCGA | IMMUNE_RESPONSE | FYN | 19737 | -0,13470003 | -0,06573491 | TUMOR | Smokers |
| TCGA | IMMUNE_RESPONSE | FYN | 17536 | -0,27293357 | -0,21543378 | TUMOR | Non-Smokers |
| GSE32863 | IMMUNE SYSTEM PROCESS | FYN | 33906 | -0,37266228 | -0,25696775 | TUMOR | Non-Smokers |
| TCGA | IMMUNE SYSTEM PROCESS | FYN | 19737 | -0,13470003 | -0,060418785 | TUMOR | Smokers |
| TCGA | IMMUNE SYSTEM PROCESS | FYN | 17536 | -0,27293357 | -0,232447 | TUMOR | Non-Smokers |
| GSE47115 | REGULATION OF IMMUNE SYSTEM PROCESS | FYN | 15472 | -0,19930254 | -0,39999682 | TUMOR | Smokers |
| TCGA | REGULATION OF IMMUNE SYSTEM PROCESS | FYN | 17536 | -0,27293357 | -0,33471668 | TUMOR | Non-Smokers |
| GSE32863 | CELLULAR DEFENSE RESPONSE | GAGE8 | 33722 | -0,36424771 | -0,28966925 | TUMOR | Non-Smokers |
| GSE32863 | DEFENSE RESPONSE | GAGE8 | 33722 | -0,36424771 | -0,2533177 | TUMOR | Non-Smokers |
| GSE50081 | DEFENSE RESPONSE | GATA3 | 18831 | -0,15691388 | -0,31113598 | TUMOR | Smokers |
| GSE32863 | DEFENSE RESPONSE | GATA3 | 32485 | -0,31376442 | -0,27715322 | TUMOR | Non-Smokers |
| GSE32863 | DEFENSE RESPONSE | GATA3 | 31837 | -0,25957826 | -0,3697361 | TUMOR | Smokers |
| TCGA | DEFENSE RESPONSE | GATA3 | 20010 | -0,15165994 | -0,06792399 | TUMOR | Smokers |
| GSE50081 | IMMUNE_RESPONSE | GBP2 | 20342 | -0,26477629 | -0,07635909 | TUMOR | Smokers |
| GSE47115 | IMMUNE_RESPONSE | GBP2 | 17583 | -0,29884982 | -0,38091612 | TUMOR | Smokers |
| GSE32863 | IMMUNE_RESPONSE | GBP2 | 34132 | -0,34406763 | -0,33734164 | TUMOR | Smokers |
| TCGA | IMMUNE_RESPONSE | GBP2 | 19013 | -0,11148605 | -0,17830074 | TUMOR | Smokers |
| GSE50081 | IMMUNE SYSTEM PROCESS | GBP2 | 20342 | -0,26477629 | -0,061362084 | TUMOR | Smokers |
| GSE47115 | IMMUNE SYSTEM PROCESS | GBP2 | 17583 | -0,29884982 | -0,35788292 | TUMOR | Smokers |
| GSE32863 | IMMUNE SYSTEM PROCESS | GBP2 | 34132 | -0,34406763 | -0,29844883 | TUMOR | Smokers |
| TCGA | IMMUNE SYSTEM PROCESS | GBP2 | 19013 | -0,11148605 | -0,16035828 | TUMOR | Smokers |
| GSE50081 | DEFENSE RESPONSE | GHRL | 20512 | -0,31707919 | -0,055712257 | TUMOR | Smokers |
| GSE32863 | DEFENSE RESPONSE | GHRL | 35353 | -0,42205054 | -0,24369732 | TUMOR | Smokers |
| TCGA | DEFENSE RESPONSE | GHRL | 16338 | -0,06664108 | -0,3720333 | TUMOR | Smokers |
| GSE47115 | DEFENSE RESPONSE | GHSR | 19389 | -0,43265581 | -0,19161327 | TUMOR | Smokers |
| TCGA | DEFENSE RESPONSE | GHSR | 20150 | -0,16563679 | -0,035508625 | TUMOR | Smokers |
| GSE50081 | CELLULAR DEFENSE RESPONSE | GNLY | 18650 | -0,15115546 | -0,20235369 | TUMOR | Smokers |
| GSE32863 | CELLULAR DEFENSE RESPONSE | GNLY | 27816 | -0,18309517 | -0,41218683 | TUMOR | Non-Smokers |
| GSE50081 | DEFENSE RESPONSE | GNLY | 18650 | -0,15115546 | -0,34414655 | TUMOR | Smokers |
| GSE32863 | DEFENSE RESPONSE | GNLY | 27816 | -0,18309517 | -0,3715514 | TUMOR | Non-Smokers |
| GSE47115 | IMMUNE_RESPONSE | GPR183 | 19898 | -0,49329031 | -0,2230267 | TUMOR | Smokers |
| TCGA | IMMUNE_RESPONSE | GPR183 | 19152 | -0,11555783 | -0,14055498 | TUMOR | Smokers |
| TCGA | IMMUNE_RESPONSE | GPR183 | 15467 | -0,19476658 | -0,36405373 | TUMOR | Non-Smokers |
| GSE47115 | IMMUNE SYSTEM PROCESS | GPR183 | 19898 | -0,49329031 | -0,21440463 | TUMOR | Smokers |
| TCGA | IMMUNE SYSTEM PROCESS | GPR183 | 19152 | -0,11555783 | -0,123927966 | TUMOR | Smokers |
| TCGA | IMMUNE SYSTEM PROCESS | GPR183 | 15467 | -0,19476658 | -0,36123678 | TUMOR | Non-Smokers |
| GSE50081 | IMMUNE_RESPONSE | GPR65 | 16426 | -0,09219699 | -0,40951785 | TUMOR | Smokers |
| GSE47115 | IMMUNE_RESPONSE | GPR65 | 18521 | -0,35498643 | -0,3342211 | TUMOR | Smokers |
| GSE32863 | IMMUNE_RESPONSE | GPR65 | 34922 | -0,42947358 | -0,21612123 | TUMOR | Non-Smokers |
| GSE32863 | IMMUNE_RESPONSE | GPR65 | 31635 | -0,25380194 | -0,40149885 | TUMOR | Smokers |
| TCGA | IMMUNE_RESPONSE | GPR65 | 15128 | -0,18419124 | -0,36108258 | TUMOR | Non-Smokers |
| GSE50081 | IMMUNE SYSTEM PROCESS | GPR65 | 16426 | -0,09219699 | -0,35985655 | TUMOR | Smokers |
| GSE47115 | IMMUNE SYSTEM PROCESS | GPR65 | 18521 | -0,35498643 | -0,32436758 | TUMOR | Smokers |
| GSE32863 | IMMUNE SYSTEM PROCESS | GPR65 | 34922 | -0,42947358 | -0,2159718 | TUMOR | Non-Smokers |
| GSE32863 | IMMUNE SYSTEM PROCESS | GPR65 | 31635 | -0,25380194 | -0,37384158 | TUMOR | Smokers |
| TCGA | IMMUNE SYSTEM PROCESS | GPR65 | 14774 | -0,04932199 | -0,3684648 | TUMOR | Smokers |
| TCGA | IMMUNE SYSTEM PROCESS | GPR65 | 15128 | -0,18419124 | -0,35727862 | TUMOR | Non-Smokers |
| GSE32863 | DEFENSE RESPONSE | GPR68 | 27826 | -0,18334027 | -0,3686582 | TUMOR | Non-Smokers |
| TCGA | DEFENSE RESPONSE | GPR68 | 19987 | -0,15000413 | -0,08517125 | TUMOR | Smokers |
| GSE50081 | IMMUNE_RESPONSE | GTPBP1 | 18981 | -0,16261533 | -0,23775703 | TUMOR | Smokers |
| TCGA | IMMUNE_RESPONSE | GTPBP1 | 18537 | -0,10091375 | -0,251059 | TUMOR | Smokers |
| GSE50081 | IMMUNE SYSTEM PROCESS | GTPBP1 | 18981 | -0,16261533 | -0,20639354 | TUMOR | Smokers |
| TCGA | IMMUNE SYSTEM PROCESS | GTPBP1 | 18537 | -0,10091375 | -0,21185972 | TUMOR | Smokers |
| GSE50081 | IMMUNE_RESPONSE | GZMA | 18485 | -0,14600413 | -0,33107877 | TUMOR | Smokers |
| GSE47115 | IMMUNE_RESPONSE | GZMA | 19777 | -0,47644189 | -0,2605637 | TUMOR | Smokers |
| GSE32863 | IMMUNE_RESPONSE | GZMA | 32532 | -0,31563663 | -0,286749 | TUMOR | Non-Smokers |
| GSE32863 | IMMUNE_RESPONSE | GZMA | 36521 | -0,62961721 | -0,0857424 | TUMOR | Smokers |
| TCGA | IMMUNE_RESPONSE | GZMA | 15653 | -0,20019275 | -0,35380086 | TUMOR | Non-Smokers |
| GSE50081 | IMMUNE SYSTEM PROCESS | GZMA | 18485 | -0,14600413 | -0,28859463 | TUMOR | Smokers |
| GSE47115 | IMMUNE SYSTEM PROCESS | GZMA | 19777 | -0,47644189 | -0,2518933 | TUMOR | Smokers |
| GSE32863 | IMMUNE SYSTEM PROCESS | GZMA | 32532 | -0,31563663 | -0,28813994 | TUMOR | Non-Smokers |
| GSE32863 | IMMUNE SYSTEM PROCESS | GZMA | 36521 | -0,62961721 | -0,062349226 | TUMOR | Smokers |
| TCGA | IMMUNE SYSTEM PROCESS | GZMA | 15653 | -0,20019275 | -0,35347745 | TUMOR | Non-Smokers |
| GSE32863 | IMMUNE_RESPONSE | HAMP | 35951 | -0,48735216 | -0,16516271 | TUMOR | Smokers |
| GSE32863 | IMMUNE SYSTEM PROCESS | HAMP | 35951 | -0,48735216 | -0,13887829 | TUMOR | Smokers |
| GSE32863 | IMMUNE SYSTEM PROCESS | HCLS1 | 35163 | -0,44878 | -0,20503351 | TUMOR | Non-Smokers |
| TCGA | IMMUNE SYSTEM PROCESS | HCLS1 | 17665 | -0,08513943 | -0,27422056 | TUMOR | Smokers |
| TCGA | IMMUNE SYSTEM PROCESS | HCLS1 | 18277 | -0,31381661 | -0,16617347 | TUMOR | Non-Smokers |
| GSE47115 | DEFENSE RESPONSE | HCP5 | 16246 | -0,23142558 | -0,34185436 | TUMOR | Smokers |
| GSE32863 | DEFENSE RESPONSE | HCP5 | 32765 | -0,32479247 | -0,26819327 | TUMOR | Non-Smokers |
| GSE32863 | DEFENSE RESPONSE | HCP5 | 30268 | -0,21883006 | -0,4018273 | TUMOR | Smokers |
| TCGA | DEFENSE RESPONSE | HCP5 | 16162 | -0,06456117 | -0,3833253 | TUMOR | Smokers |
| GSE32863 | IMMUNE SYSTEM PROCESS | HDAC4 | 28261 | -0,19374247 | -0,38672847 | TUMOR | Non-Smokers |
| GSE32863 | DEFENSE RESPONSE | HDAC4 | 28261 | -0,19374247 | -0,3576169 | TUMOR | Non-Smokers |
| GSE47115 | IMMUNE SYSTEM PROCESS | HDAC5 | 19620 | -0,45627782 | -0,26826814 | TUMOR | Smokers |
| TCGA | IMMUNE SYSTEM PROCESS | HDAC5 | 19239 | -0,11758058 | -0,100447536 | TUMOR | Smokers |
| GSE47115 | DEFENSE RESPONSE | HDAC5 | 19620 | -0,45627782 | -0,16314688 | TUMOR | Smokers |
| TCGA | DEFENSE RESPONSE | HDAC5 | 19239 | -0,11758058 | -0,16898079 | TUMOR | Smokers |
| TCGA | IMMUNE SYSTEM PROCESS | HDAC7 | 19911 | -0,14478473 | -0,03564606 | TUMOR | Smokers |
| TCGA | DEFENSE RESPONSE | HDAC7 | 19911 | -0,14478473 | -0,09936529 | TUMOR | Smokers |
| TCGA | IMMUNE SYSTEM PROCESS | HDAC9 | 15657 | -0,05878692 | -0,3649683 | TUMOR | Smokers |
| TCGA | IMMUNE SYSTEM PROCESS | HDAC9 | 16498 | -0,23015442 | -0,30573443 | TUMOR | Non-Smokers |
| GSE47115 | DEFENSE RESPONSE | HDAC9 | 14933 | -0,17739812 | -0,34665963 | TUMOR | Smokers |
| TCGA | DEFENSE RESPONSE | HDAC9 | 15657 | -0,05878692 | -0,41922224 | TUMOR | Smokers |
| GSE32863 | IMMUNE SYSTEM PROCESS | HELLS | 32400 | -0,31083161 | -0,30508047 | TUMOR | Non-Smokers |
| GSE47115 | DEFENSE RESPONSE | HLA-B | 18657 | -0,36433512 | -0,21349335 | TUMOR | Smokers |
| GSE32863 | DEFENSE RESPONSE | HLA-B | 31036 | -0,26635784 | -0,293 | TUMOR | Non-Smokers |
| TCGA | DEFENSE RESPONSE | HLA-B | 15974 | -0,06238956 | -0,40515345 | TUMOR | Smokers |
| GSE47115 | CELLULAR DEFENSE RESPONSE | HLA-G | 16671 | -0,25233686 | -0,36552036 | TUMOR | Smokers |
| GSE32863 | CELLULAR DEFENSE RESPONSE | HLA-G | 27911 | -0,18561901 | -0,39977407 | TUMOR | Non-Smokers |
| GSE47115 | DEFENSE RESPONSE | HLA-G | 16671 | -0,25233686 | -0,3285011 | TUMOR | Smokers |
| GSE32863 | DEFENSE RESPONSE | HLA-G | 27911 | -0,18561901 | -0,36460745 | TUMOR | Non-Smokers |
| GSE32863 | DEFENSE RESPONSE | HLA-G | 28890 | -0,18710193 | -0,4240567 | TUMOR | Smokers |
| GSE32863 | DEFENSE RESPONSE | HP | 28260 | -0,19371134 | -0,36093524 | TUMOR | Non-Smokers |
| GSE32863 | DEFENSE RESPONSE | HRH1 | 31363 | -0,24647653 | -0,37772638 | TUMOR | Smokers |
| TCGA | DEFENSE RESPONSE | HRH1 | 16782 | -0,07251729 | -0,3643212 | TUMOR | Smokers |
| GSE50081 | IMMUNE_RESPONSE | HRH2 | 16090 | -0,08571842 | -0,41183868 | TUMOR | Smokers |
| GSE47115 | IMMUNE_RESPONSE | HRH2 | 16813 | -0,25879824 | -0,41329437 | TUMOR | Smokers |
| GSE50081 | IMMUNE SYSTEM PROCESS | HRH2 | 16090 | -0,08571842 | -0,3654121 | TUMOR | Smokers |
| GSE47115 | IMMUNE SYSTEM PROCESS | HRH2 | 16813 | -0,25879824 | -0,37779492 | TUMOR | Smokers |
| GSE50081 | IMMUNE SYSTEM PROCESS | ICOSLG | 16024 | -0,08418843 | -0,3675162 | TUMOR | Smokers |
| GSE32863 | IMMUNE SYSTEM PROCESS | ICOSLG | 36416 | -0,63443232 | -0,05414746 | TUMOR | Non-Smokers |
| GSE32863 | IMMUNE SYSTEM PROCESS | ICOSLG | 28969 | -0,18893428 | -0,40632087 | TUMOR | Smokers |
| TCGA | IMMUNE SYSTEM PROCESS | ICOSLG | 17910 | -0,0892507 | -0,2531802 | TUMOR | Smokers |
| GSE10072 | REGULATION OF IMMUNE SYSTEM PROCESS | ICOSLG | 12783 | -0,55282712 | -0,1100494 | NORMAL | Non-Smokers |
| GSE32863 | REGULATION OF IMMUNE SYSTEM PROCESS | ICOSLG | 28969 | -0,18893428 | -0,3831142 | TUMOR | Smokers |
| GSE50081 | IMMUNE SYSTEM PROCESS | IFI16 | 19223 | -0,17391241 | -0,1711506 | TUMOR | Smokers |
| GSE32863 | IMMUNE SYSTEM PROCESS | IFI16 | 32402 | -0,31088081 | -0,3010536 | TUMOR | Non-Smokers |
| TCGA | IMMUNE SYSTEM PROCESS | IFI16 | 17098 | -0,07679553 | -0,30748633 | TUMOR | Smokers |
| TCGA | IMMUNE SYSTEM PROCESS | IFI16 | 17580 | -0,27487814 | -0,22512043 | TUMOR | Non-Smokers |
| TCGA | IMMUNE_RESPONSE | IFI6 | 18230 | -0,3114202 | -0,1468486 | TUMOR | Non-Smokers |
| TCGA | IMMUNE SYSTEM PROCESS | IFI6 | 18230 | -0,3114202 | -0,17471917 | TUMOR | Non-Smokers |
| TCGA | IMMUNE SYSTEM PROCESS | IFITM2 | 15402 | -0,05601405 | -0,36628157 | TUMOR | Smokers |
| GSE50081 | IMMUNE_RESPONSE | IFITM3 | 16979 | -0,10523618 | -0,41158155 | TUMOR | Smokers |
| GSE50081 | IMMUNE SYSTEM PROCESS | IFITM3 | 16979 | -0,10523618 | -0,35956022 | TUMOR | Smokers |
| GSE32863 | IMMUNE_RESPONSE | IFNK | 34674 | -0,41276526 | -0,24879585 | TUMOR | Non-Smokers |
| GSE32863 | IMMUNE_RESPONSE | IFNK | 29071 | -0,19110861 | -0,43281364 | TUMOR | Smokers |
| TCGA | IMMUNE_RESPONSE | IFNK | 16575 | -0,06958997 | -0,37765095 | TUMOR | Smokers |
| TCGA | IMMUNE_RESPONSE | IFNK | 16016 | -0,21228954 | -0,3361826 | TUMOR | Non-Smokers |
| GSE32863 | IMMUNE SYSTEM PROCESS | IFNK | 34674 | -0,41276526 | -0,24233036 | TUMOR | Non-Smokers |
| GSE32863 | IMMUNE SYSTEM PROCESS | IFNK | 29071 | -0,19110861 | -0,40644252 | TUMOR | Smokers |
| TCGA | IMMUNE SYSTEM PROCESS | IFNK | 16575 | -0,06958997 | -0,33439118 | TUMOR | Smokers |
| TCGA | IMMUNE SYSTEM PROCESS | IFNK | 16016 | -0,21228954 | -0,34313124 | TUMOR | Non-Smokers |
| GSE32863 | DEFENSE RESPONSE | IFNK | 34674 | -0,41276526 | -0,1583848 | TUMOR | Non-Smokers |
| GSE32863 | DEFENSE RESPONSE | IFNK | 29071 | -0,19110861 | -0,42269555 | TUMOR | Smokers |
| TCGA | DEFENSE RESPONSE | IFNK | 16575 | -0,06958997 | -0,3670677 | TUMOR | Smokers |
| GSE32863 | REGULATION OF IMMUNE SYSTEM PROCESS | IFNK | 29071 | -0,19110861 | -0,37377062 | TUMOR | Smokers |
| TCGA | REGULATION OF IMMUNE SYSTEM PROCESS | IFNK | 16016 | -0,21228954 | -0,41709486 | TUMOR | Non-Smokers |
| GSE50081 | IMMUNE_RESPONSE | IGSF6 | 19330 | -0,17860791 | -0,21122958 | TUMOR | Smokers |
| GSE47115 | IMMUNE_RESPONSE | IGSF6 | 20443 | -0,63395858 | -0,097152695 | TUMOR | Smokers |
| GSE32863 | IMMUNE_RESPONSE | IGSF6 | 35780 | -0,50706506 | -0,15943034 | TUMOR | Non-Smokers |
| GSE32863 | IMMUNE_RESPONSE | IGSF6 | 35343 | -0,42118481 | -0,23195991 | TUMOR | Smokers |
| TCGA | IMMUNE_RESPONSE | IGSF6 | 17949 | -0,29570159 | -0,19340791 | TUMOR | Non-Smokers |
| GSE50081 | IMMUNE SYSTEM PROCESS | IGSF6 | 19330 | -0,17860791 | -0,15451732 | TUMOR | Smokers |
| GSE47115 | IMMUNE SYSTEM PROCESS | IGSF6 | 20443 | -0,63395858 | -0,10214949 | TUMOR | Smokers |
| GSE32863 | IMMUNE SYSTEM PROCESS | IGSF6 | 35780 | -0,50706506 | -0,15288983 | TUMOR | Non-Smokers |
| GSE32863 | IMMUNE SYSTEM PROCESS | IGSF6 | 35343 | -0,42118481 | -0,19684464 | TUMOR | Smokers |
| TCGA | IMMUNE SYSTEM PROCESS | IGSF6 | 14508 | -0,04652205 | -0,37361062 | TUMOR | Smokers |
| TCGA | IMMUNE SYSTEM PROCESS | IGSF6 | 17949 | -0,29570159 | -0,21361786 | TUMOR | Non-Smokers |
| GSE50081 | IMMUNE_RESPONSE | IKBKG | 18548 | -0,14785039 | -0,3279607 | TUMOR | Smokers |
| GSE32863 | IMMUNE_RESPONSE | IKBKG | 30602 | -0,25393447 | -0,325048 | TUMOR | Non-Smokers |
| GSE32863 | IMMUNE_RESPONSE | IKBKG | 33693 | -0,32476646 | -0,38203633 | TUMOR | Smokers |
| TCGA | IMMUNE_RESPONSE | IKBKG | 18040 | -0,3013052 | -0,17569898 | TUMOR | Non-Smokers |
| GSE50081 | IMMUNE SYSTEM PROCESS | IKBKG | 18548 | -0,14785039 | -0,28711298 | TUMOR | Smokers |
| GSE32863 | IMMUNE SYSTEM PROCESS | IKBKG | 30602 | -0,25393447 | -0,34463337 | TUMOR | Non-Smokers |
| GSE32863 | IMMUNE SYSTEM PROCESS | IKBKG | 33693 | -0,32476646 | -0,33780524 | TUMOR | Smokers |
| TCGA | IMMUNE SYSTEM PROCESS | IKBKG | 14289 | -0,04448063 | -0,37585574 | TUMOR | Smokers |
| TCGA | IMMUNE SYSTEM PROCESS | IKBKG | 18040 | -0,3013052 | -0,2026304 | TUMOR | Non-Smokers |
| GSE50081 | REGULATION OF IMMUNE SYSTEM PROCESS | IKBKG | 18548 | -0,14785039 | -0,40240225 | TUMOR | Smokers |
| GSE32863 | REGULATION OF IMMUNE SYSTEM PROCESS | IKBKG | 33693 | -0,32476646 | -0,3089777 | TUMOR | Smokers |
| TCGA | REGULATION OF IMMUNE SYSTEM PROCESS | IKBKG | 18040 | -0,3013052 | -0,2399612 | TUMOR | Non-Smokers |
| GSE50081 | IMMUNE_RESPONSE | IL10 | 20558 | -0,35592818 | -0,012609664 | TUMOR | Smokers |
| GSE32863 | IMMUNE_RESPONSE | IL10 | 32294 | -0,30663052 | -0,30369997 | TUMOR | Non-Smokers |
| GSE32863 | IMMUNE_RESPONSE | IL10 | 32067 | -0,26596862 | -0,3937085 | TUMOR | Smokers |
| GSE50081 | IMMUNE SYSTEM PROCESS | IL10 | 20558 | -0,35592818 | -0,008682135 | TUMOR | Smokers |
| GSE32863 | IMMUNE SYSTEM PROCESS | IL10 | 32294 | -0,30663052 | -0,31032157 | TUMOR | Non-Smokers |
| GSE32863 | IMMUNE SYSTEM PROCESS | IL10 | 32067 | -0,26596862 | -0,36388326 | TUMOR | Smokers |
| GSE32863 | IMMUNE_RESPONSE | IL10RB | 31728 | -0,28757948 | -0,31041905 | TUMOR | Non-Smokers |
| TCGA | IMMUNE_RESPONSE | IL10RB | 17091 | -0,07671978 | -0,35003147 | TUMOR | Smokers |
| TCGA | IMMUNE_RESPONSE | IL10RB | 15798 | -0,20479058 | -0,34078032 | TUMOR | Non-Smokers |
| GSE32863 | IMMUNE SYSTEM PROCESS | IL10RB | 31728 | -0,28757948 | -0,32232326 | TUMOR | Non-Smokers |
| TCGA | IMMUNE SYSTEM PROCESS | IL10RB | 17091 | -0,07671978 | -0,3107975 | TUMOR | Smokers |
| TCGA | IMMUNE SYSTEM PROCESS | IL10RB | 15798 | -0,20479058 | -0,34659028 | TUMOR | Non-Smokers |
| GSE32863 | DEFENSE RESPONSE | IL10RB | 31728 | -0,28757948 | -0,29260573 | TUMOR | Non-Smokers |
| TCGA | DEFENSE RESPONSE | IL10RB | 17091 | -0,07671978 | -0,34789068 | TUMOR | Smokers |
| TCGA | IMMUNE_RESPONSE | IL12A | 17821 | -0,28723505 | -0,20885693 | TUMOR | Non-Smokers |
| TCGA | IMMUNE SYSTEM PROCESS | IL12A | 17821 | -0,28723505 | -0,22245005 | TUMOR | Non-Smokers |
| TCGA | REGULATION OF IMMUNE SYSTEM PROCESS | IL12A | 17821 | -0,28723505 | -0,27807638 | TUMOR | Non-Smokers |
| GSE47115 | IMMUNE_RESPONSE | IL12B | 17217 | -0,28000337 | -0,39424986 | TUMOR | Smokers |
| TCGA | IMMUNE_RESPONSE | IL12B | 18805 | -0,10641748 | -0,20390995 | TUMOR | Smokers |
| GSE47115 | IMMUNE SYSTEM PROCESS | IL12B | 17217 | -0,28000337 | -0,36614674 | TUMOR | Smokers |
| TCGA | IMMUNE SYSTEM PROCESS | IL12B | 18805 | -0,10641748 | -0,18121003 | TUMOR | Smokers |
| GSE47115 | DEFENSE RESPONSE | IL12B | 17217 | -0,28000337 | -0,26945004 | TUMOR | Smokers |
| TCGA | DEFENSE RESPONSE | IL12B | 18805 | -0,10641748 | -0,20839848 | TUMOR | Smokers |
| GSE47115 | REGULATION OF IMMUNE SYSTEM PROCESS | IL12B | 17217 | -0,28000337 | -0,357555 | TUMOR | Smokers |
| GSE10072 | REGULATION OF IMMUNE SYSTEM PROCESS | IL12B | 10550 | -0,29135081 | -0,4260547 | NORMAL | Non-Smokers |
| GSE32863 | DEFENSE RESPONSE | IL13 | 34955 | -0,39213866 | -0,2734058 | TUMOR | Smokers |
| GSE50081 | IMMUNE_RESPONSE | IL15 | 16738 | -0,0993776 | -0,41265246 | TUMOR | Smokers |
| GSE47115 | IMMUNE_RESPONSE | IL15 | 17897 | -0,31650382 | -0,3578138 | TUMOR | Smokers |
| GSE32863 | IMMUNE_RESPONSE | IL15 | 36380 | -0,57115108 | -0,1272902 | TUMOR | Smokers |
| TCGA | IMMUNE_RESPONSE | IL15 | 15782 | -0,20433901 | -0,34508753 | TUMOR | Non-Smokers |
| GSE50081 | IMMUNE SYSTEM PROCESS | IL15 | 16738 | -0,0993776 | -0,3603477 | TUMOR | Smokers |
| GSE47115 | IMMUNE SYSTEM PROCESS | IL15 | 17897 | -0,31650382 | -0,34564134 | TUMOR | Smokers |
| GSE32863 | IMMUNE SYSTEM PROCESS | IL15 | 36380 | -0,57115108 | -0,09218492 | TUMOR | Smokers |
| TCGA | IMMUNE SYSTEM PROCESS | IL15 | 15782 | -0,20433901 | -0,3493627 | TUMOR | Non-Smokers |
| GSE50081 | IMMUNE_RESPONSE | IL16 | 16739 | -0,09940996 | -0,40851098 | TUMOR | Smokers |
| GSE32863 | IMMUNE_RESPONSE | IL16 | 35373 | -0,42350274 | -0,21687871 | TUMOR | Smokers |
| TCGA | IMMUNE_RESPONSE | IL16 | 19271 | -0,11859258 | -0,100487076 | TUMOR | Smokers |
| GSE50081 | IMMUNE SYSTEM PROCESS | IL16 | 16739 | -0,09940996 | -0,35729763 | TUMOR | Smokers |
| GSE32863 | IMMUNE SYSTEM PROCESS | IL16 | 35373 | -0,42350274 | -0,18585937 | TUMOR | Smokers |
| TCGA | IMMUNE SYSTEM PROCESS | IL16 | 19271 | -0,11859258 | -0,090821534 | TUMOR | Smokers |
| GSE50081 | IMMUNE_RESPONSE | IL17A | 20369 | -0,26898247 | -0,055200115 | TUMOR | Smokers |
| GSE32863 | IMMUNE_RESPONSE | IL17A | 29769 | -0,20668985 | -0,41429874 | TUMOR | Smokers |
| TCGA | IMMUNE_RESPONSE | IL17A | 15654 | -0,0587604 | -0,42270702 | TUMOR | Smokers |
| TCGA | IMMUNE_RESPONSE | IL17A | 15317 | -0,19063236 | -0,36129168 | TUMOR | Non-Smokers |
| GSE50081 | IMMUNE SYSTEM PROCESS | IL17A | 20369 | -0,26898247 | -0,046107672 | TUMOR | Smokers |
| GSE32863 | IMMUNE SYSTEM PROCESS | IL17A | 29769 | -0,20668985 | -0,39209625 | TUMOR | Smokers |
| TCGA | IMMUNE SYSTEM PROCESS | IL17A | 15654 | -0,0587604 | -0,37044513 | TUMOR | Smokers |
| TCGA | IMMUNE SYSTEM PROCESS | IL17A | 15317 | -0,19063236 | -0,36034462 | TUMOR | Non-Smokers |
| GSE47115 | DEFENSE RESPONSE | IL17C | 16989 | -0,2671172 | -0,31638953 | TUMOR | Smokers |
| GSE47115 | DEFENSE RESPONSE | IL17RB | 20675 | -0,85850376 | 0,0023949 | TUMOR | Smokers |
| GSE32863 | DEFENSE RESPONSE | IL17RB | 29457 | -0,22254977 | -0,34691525 | TUMOR | Non-Smokers |
| GSE50081 | IMMUNE_RESPONSE | IL18 | 17815 | -0,12470292 | -0,35935923 | TUMOR | Smokers |
| GSE32863 | IMMUNE_RESPONSE | IL18 | 35260 | -0,45574355 | -0,2000947 | TUMOR | Non-Smokers |
| TCGA | IMMUNE_RESPONSE | IL18 | 17904 | -0,29327092 | -0,19848995 | TUMOR | Non-Smokers |
| GSE50081 | IMMUNE SYSTEM PROCESS | IL18 | 17815 | -0,12470292 | -0,3211551 | TUMOR | Smokers |
| GSE32863 | IMMUNE SYSTEM PROCESS | IL18 | 35260 | -0,45574355 | -0,1958303 | TUMOR | Non-Smokers |
| TCGA | IMMUNE SYSTEM PROCESS | IL18 | 17904 | -0,29327092 | -0,21647827 | TUMOR | Non-Smokers |
| GSE10072 | REGULATION OF IMMUNE SYSTEM PROCESS | IL18 | 11385 | -0,3582184 | -0,37116447 | NORMAL | Non-Smokers |
| TCGA | REGULATION OF IMMUNE SYSTEM PROCESS | IL18 | 17904 | -0,29327092 | -0,25801262 | TUMOR | Non-Smokers |
| GSE50081 | IMMUNE_RESPONSE | IL18BP | 17940 | -0,12815815 | -0,3442909 | TUMOR | Smokers |
| GSE32863 | IMMUNE_RESPONSE | IL18BP | 28769 | -0,20575449 | -0,35790154 | TUMOR | Non-Smokers |
| GSE32863 | IMMUNE_RESPONSE | IL18BP | 30504 | -0,22487798 | -0,4020309 | TUMOR | Smokers |
| TCGA | IMMUNE_RESPONSE | IL18BP | 17511 | -0,27108264 | -0,22097014 | TUMOR | Non-Smokers |
| GSE50081 | IMMUNE SYSTEM PROCESS | IL18BP | 17940 | -0,12815815 | -0,3077606 | TUMOR | Smokers |
| GSE32863 | IMMUNE SYSTEM PROCESS | IL18BP | 28769 | -0,20575449 | -0,37701052 | TUMOR | Non-Smokers |
| GSE32863 | IMMUNE SYSTEM PROCESS | IL18BP | 30504 | -0,22487798 | -0,36966157 | TUMOR | Smokers |
| TCGA | IMMUNE SYSTEM PROCESS | IL18BP | 14574 | -0,04732137 | -0,37011042 | TUMOR | Smokers |
| TCGA | IMMUNE SYSTEM PROCESS | IL18BP | 17511 | -0,27108264 | -0,23593687 | TUMOR | Non-Smokers |
| GSE50081 | DEFENSE RESPONSE | IL18RAP | 19181 | -0,17178079 | -0,29617184 | TUMOR | Smokers |
| GSE32863 | DEFENSE RESPONSE | IL18RAP | 35283 | -0,45809886 | -0,12294256 | TUMOR | Non-Smokers |
| GSE32863 | DEFENSE RESPONSE | IL18RAP | 33329 | -0,30915618 | -0,32653138 | TUMOR | Smokers |
| GSE32863 | DEFENSE RESPONSE | IL1A | 35535 | -0,48109603 | -0,08145502 | TUMOR | Non-Smokers |
| GSE32863 | DEFENSE RESPONSE | IL1A | 35794 | -0,46498105 | -0,14487039 | TUMOR | Smokers |
| TCGA | DEFENSE RESPONSE | IL1A | 17027 | -0,07587597 | -0,35404056 | TUMOR | Smokers |
| GSE50081 | IMMUNE_RESPONSE | IL1R2 | 17856 | -0,12576897 | -0,3455584 | TUMOR | Smokers |
| GSE32863 | IMMUNE_RESPONSE | IL1R2 | 35927 | -0,52626073 | -0,11479116 | TUMOR | Non-Smokers |
| GSE32863 | IMMUNE_RESPONSE | IL1R2 | 36456 | -0,59875697 | -0,107259475 | TUMOR | Smokers |
| GSE50081 | IMMUNE SYSTEM PROCESS | IL1R2 | 17856 | -0,12576897 | -0,30760342 | TUMOR | Smokers |
| GSE32863 | IMMUNE SYSTEM PROCESS | IL1R2 | 35927 | -0,52626073 | -0,12287555 | TUMOR | Non-Smokers |
| GSE32863 | IMMUNE SYSTEM PROCESS | IL1R2 | 36456 | -0,59875697 | -0,077857606 | TUMOR | Smokers |
| GSE47115 | DEFENSE RESPONSE | IL1RAP | 15492 | -0,20008861 | -0,34685454 | TUMOR | Smokers |
| GSE32863 | DEFENSE RESPONSE | IL1RAP | 29689 | -0,22892992 | -0,3414983 | TUMOR | Non-Smokers |
| TCGA | DEFENSE RESPONSE | IL1RAP | 18790 | -0,10620874 | -0,21413033 | TUMOR | Smokers |
| GSE47115 | CELLULAR DEFENSE RESPONSE | IL1RL2 | 19178 | -0,41119829 | -0,2057852 | TUMOR | Smokers |
| GSE32863 | CELLULAR DEFENSE RESPONSE | IL1RL2 | 28927 | -0,20943874 | -0,37921706 | TUMOR | Non-Smokers |
| GSE47115 | DEFENSE RESPONSE | IL1RL2 | 19178 | -0,41119829 | -0,20393945 | TUMOR | Smokers |
| GSE32863 | DEFENSE RESPONSE | IL1RL2 | 28927 | -0,20943874 | -0,35466897 | TUMOR | Non-Smokers |
| GSE50081 | IMMUNE_RESPONSE | IL2 | 17529 | -0,1179432 | -0,38626477 | TUMOR | Smokers |
| GSE47115 | IMMUNE_RESPONSE | IL2 | 17618 | -0,30090716 | -0,37725294 | TUMOR | Smokers |
| GSE32863 | IMMUNE_RESPONSE | IL2 | 29679 | -0,20469753 | -0,41961712 | TUMOR | Smokers |
| GSE50081 | IMMUNE SYSTEM PROCESS | IL2 | 17529 | -0,1179432 | -0,34106967 | TUMOR | Smokers |
| GSE47115 | IMMUNE SYSTEM PROCESS | IL2 | 17618 | -0,30090716 | -0,3557356 | TUMOR | Smokers |
| GSE32863 | IMMUNE SYSTEM PROCESS | IL2 | 29679 | -0,20469753 | -0,39541152 | TUMOR | Smokers |
| TCGA | IMMUNE SYSTEM PROCESS | IL2 | 14286 | -0,04441683 | -0,37784722 | TUMOR | Smokers |
| GSE50081 | IMMUNE SYSTEM PROCESS | IL21 | 16121 | -0,08631372 | -0,3642419 | TUMOR | Smokers |
| TCGA | IMMUNE SYSTEM PROCESS | IL21 | 18153 | -0,30699793 | -0,19241473 | TUMOR | Non-Smokers |
| TCGA | REGULATION OF IMMUNE SYSTEM PROCESS | IL21 | 18153 | -0,30699793 | -0,1951477 | TUMOR | Non-Smokers |
| GSE47115 | IMMUNE_RESPONSE | IL27 | 20346 | -0,60233253 | -0,1581246 | TUMOR | Smokers |
| GSE47115 | IMMUNE SYSTEM PROCESS | IL27 | 20346 | -0,60233253 | -0,16022675 | TUMOR | Smokers |
| GSE47115 | REGULATION OF IMMUNE SYSTEM PROCESS | IL27 | 20346 | -0,60233253 | -0,12980214 | TUMOR | Smokers |
| GSE47115 | IMMUNE_RESPONSE | IL27RA | 20161 | -0,54930556 | -0,1704929 | TUMOR | Smokers |
| TCGA | IMMUNE_RESPONSE | IL27RA | 18962 | -0,11026191 | -0,18303697 | TUMOR | Smokers |
| GSE47115 | IMMUNE SYSTEM PROCESS | IL27RA | 20161 | -0,54930556 | -0,1740493 | TUMOR | Smokers |
| TCGA | IMMUNE SYSTEM PROCESS | IL27RA | 18962 | -0,11026191 | -0,16310525 | TUMOR | Smokers |
| GSE47115 | IMMUNE_RESPONSE | IL28RA | 19371 | -0,43071616 | -0,30498078 | TUMOR | Smokers |
| TCGA | IMMUNE_RESPONSE | IL28RA | 20002 | -0,15115553 | -0,04190145 | TUMOR | Smokers |
| TCGA | IMMUNE_RESPONSE | IL28RA | 16337 | -0,22336292 | -0,30876747 | TUMOR | Non-Smokers |
| GSE47115 | IMMUNE SYSTEM PROCESS | IL28RA | 19371 | -0,43071616 | -0,2899925 | TUMOR | Smokers |
| TCGA | IMMUNE SYSTEM PROCESS | IL28RA | 20002 | -0,15115553 | -0,033029646 | TUMOR | Smokers |
| TCGA | IMMUNE SYSTEM PROCESS | IL28RA | 16337 | -0,22336292 | -0,32133168 | TUMOR | Non-Smokers |
| GSE47115 | DEFENSE RESPONSE | IL28RA | 19371 | -0,43071616 | -0,19834986 | TUMOR | Smokers |
| TCGA | DEFENSE RESPONSE | IL28RA | 20002 | -0,15115553 | -0,07673695 | TUMOR | Smokers |
| GSE47115 | REGULATION OF IMMUNE SYSTEM PROCESS | IL28RA | 19371 | -0,43071616 | -0,29549202 | TUMOR | Smokers |
| TCGA | REGULATION OF IMMUNE SYSTEM PROCESS | IL28RA | 16337 | -0,22336292 | -0,3782612 | TUMOR | Non-Smokers |
| GSE47115 | IMMUNE_RESPONSE | IL29 | 19673 | -0,4633587 | -0,2723615 | TUMOR | Smokers |
| TCGA | IMMUNE_RESPONSE | IL29 | 19048 | -0,37765807 | -0,13001406 | TUMOR | Non-Smokers |
| GSE47115 | IMMUNE SYSTEM PROCESS | IL29 | 19673 | -0,4633587 | -0,26494414 | TUMOR | Smokers |
| TCGA | IMMUNE SYSTEM PROCESS | IL29 | 14542 | -0,04703027 | -0,37078863 | TUMOR | Smokers |
| TCGA | IMMUNE SYSTEM PROCESS | IL29 | 19048 | -0,37765807 | -0,15341969 | TUMOR | Non-Smokers |
| GSE47115 | DEFENSE RESPONSE | IL29 | 19673 | -0,4633587 | -0,14954643 | TUMOR | Smokers |
| TCGA | DEFENSE RESPONSE | IL29 | 14542 | -0,04703027 | -0,41777366 | TUMOR | Smokers |
| GSE47115 | REGULATION OF IMMUNE SYSTEM PROCESS | IL29 | 19673 | -0,4633587 | -0,2826291 | TUMOR | Smokers |
| TCGA | REGULATION OF IMMUNE SYSTEM PROCESS | IL29 | 19048 | -0,37765807 | -0,12946153 | TUMOR | Non-Smokers |
| GSE50081 | IMMUNE_RESPONSE | IL2RA | 19060 | -0,16575505 | -0,23467793 | TUMOR | Smokers |
| GSE32863 | IMMUNE_RESPONSE | IL2RA | 36167 | -0,56877846 | -0,05932993 | TUMOR | Non-Smokers |
| GSE32863 | IMMUNE_RESPONSE | IL2RA | 33719 | -0,32612532 | -0,37661403 | TUMOR | Smokers |
| TCGA | IMMUNE_RESPONSE | IL2RA | 17088 | -0,25203717 | -0,25153312 | TUMOR | Non-Smokers |
| GSE50081 | IMMUNE SYSTEM PROCESS | IL2RA | 19060 | -0,16575505 | -0,19494577 | TUMOR | Smokers |
| GSE32863 | IMMUNE SYSTEM PROCESS | IL2RA | 36167 | -0,56877846 | -0,07873823 | TUMOR | Non-Smokers |
| GSE32863 | IMMUNE SYSTEM PROCESS | IL2RA | 33719 | -0,32612532 | -0,33396238 | TUMOR | Smokers |
| TCGA | IMMUNE SYSTEM PROCESS | IL2RA | 17088 | -0,25203717 | -0,25974897 | TUMOR | Non-Smokers |
| GSE50081 | IMMUNE_RESPONSE | IL2RG | 20098 | -0,22926778 | -0,13708743 | TUMOR | Smokers |
| GSE47115 | IMMUNE_RESPONSE | IL2RG | 20563 | -0,71729839 | -0,066388786 | TUMOR | Smokers |
| GSE32863 | IMMUNE_RESPONSE | IL2RG | 35113 | -0,44512275 | -0,21302441 | TUMOR | Non-Smokers |
| GSE32863 | IMMUNE_RESPONSE | IL2RG | 34428 | -0,35845882 | -0,3322048 | TUMOR | Smokers |
| TCGA | IMMUNE_RESPONSE | IL2RG | 17382 | -0,08072976 | -0,32825518 | TUMOR | Smokers |
| TCGA | IMMUNE_RESPONSE | IL2RG | 18181 | -0,30882749 | -0,16755943 | TUMOR | Non-Smokers |
| GSE50081 | IMMUNE SYSTEM PROCESS | IL2RG | 20098 | -0,22926778 | -0,10297232 | TUMOR | Smokers |
| GSE47115 | IMMUNE SYSTEM PROCESS | IL2RG | 20563 | -0,71729839 | -0,06491491 | TUMOR | Smokers |
| GSE32863 | IMMUNE SYSTEM PROCESS | IL2RG | 35113 | -0,44512275 | -0,2153894 | TUMOR | Non-Smokers |
| GSE32863 | IMMUNE SYSTEM PROCESS | IL2RG | 34428 | -0,35845882 | -0,29674143 | TUMOR | Smokers |
| TCGA | IMMUNE SYSTEM PROCESS | IL2RG | 17382 | -0,08072976 | -0,29154828 | TUMOR | Smokers |
| TCGA | IMMUNE SYSTEM PROCESS | IL2RG | 18181 | -0,30882749 | -0,18845794 | TUMOR | Non-Smokers |
| TCGA | IMMUNE SYSTEM PROCESS | IL31RA | 15284 | -0,05453731 | -0,37358952 | TUMOR | Smokers |
| GSE50081 | IMMUNE_RESPONSE | IL32 | 19954 | -0,2170226 | -0,14911656 | TUMOR | Smokers |
| GSE47115 | IMMUNE_RESPONSE | IL32 | 20666 | -0,84037155 | -0,028222017 | TUMOR | Smokers |
| GSE32863 | IMMUNE_RESPONSE | IL32 | 34122 | -0,3826057 | -0,25623858 | TUMOR | Non-Smokers |
| GSE32863 | IMMUNE_RESPONSE | IL32 | 29896 | -0,20977646 | -0,40598494 | TUMOR | Smokers |
| TCGA | IMMUNE_RESPONSE | IL32 | 17958 | -0,09007832 | -0,27911207 | TUMOR | Smokers |
| GSE50081 | IMMUNE SYSTEM PROCESS | IL32 | 19954 | -0,2170226 | -0,11682811 | TUMOR | Smokers |
| GSE47115 | IMMUNE SYSTEM PROCESS | IL32 | 20666 | -0,84037155 | -0,01943721 | TUMOR | Smokers |
| GSE32863 | IMMUNE SYSTEM PROCESS | IL32 | 34122 | -0,3826057 | -0,2480522 | TUMOR | Non-Smokers |
| GSE32863 | IMMUNE SYSTEM PROCESS | IL32 | 29896 | -0,20977646 | -0,3868168 | TUMOR | Smokers |
| TCGA | IMMUNE SYSTEM PROCESS | IL32 | 17958 | -0,09007832 | -0,25129005 | TUMOR | Smokers |
| GSE50081 | DEFENSE RESPONSE | IL32 | 19954 | -0,2170226 | -0,21939819 | TUMOR | Smokers |
| GSE47115 | DEFENSE RESPONSE | IL32 | 20666 | -0,84037155 | -0,012230032 | TUMOR | Smokers |
| GSE32863 | DEFENSE RESPONSE | IL32 | 34122 | -0,3826057 | -0,21917742 | TUMOR | Non-Smokers |
| GSE32863 | DEFENSE RESPONSE | IL32 | 29896 | -0,20977646 | -0,39886105 | TUMOR | Smokers |
| TCGA | DEFENSE RESPONSE | IL32 | 17958 | -0,09007832 | -0,2735197 | TUMOR | Smokers |
| GSE50081 | IMMUNE_RESPONSE | IL4 | 18798 | -0,15568779 | -0,288794 | TUMOR | Smokers |
| GSE32863 | IMMUNE_RESPONSE | IL4 | 27778 | -0,18217731 | -0,367318 | TUMOR | Non-Smokers |
| GSE50081 | IMMUNE SYSTEM PROCESS | IL4 | 18798 | -0,15568779 | -0,2614479 | TUMOR | Smokers |
| GSE32863 | IMMUNE SYSTEM PROCESS | IL4 | 27778 | -0,18217731 | -0,38838693 | TUMOR | Non-Smokers |
| GSE50081 | CELLULAR DEFENSE RESPONSE | IL4 | 18798 | -0,15568779 | -0,18136375 | TUMOR | Smokers |
| GSE47115 | CELLULAR DEFENSE RESPONSE | IL4 | 14665 | -0,16649385 | -0,4031953 | TUMOR | Smokers |
| GSE32863 | CELLULAR DEFENSE RESPONSE | IL4 | 27778 | -0,18217731 | -0,44068056 | TUMOR | Non-Smokers |
| TCGA | CELLULAR DEFENSE RESPONSE | IL4 | 13996 | -0,04134645 | -0,42683637 | TUMOR | Smokers |
| GSE50081 | DEFENSE RESPONSE | IL4 | 18798 | -0,15568779 | -0,31560132 | TUMOR | Smokers |
| GSE32863 | DEFENSE RESPONSE | IL4 | 27778 | -0,18217731 | -0,37682003 | TUMOR | Non-Smokers |
| GSE50081 | REGULATION OF IMMUNE SYSTEM PROCESS | IL4 | 18798 | -0,15568779 | -0,32590967 | TUMOR | Smokers |
| GSE47115 | REGULATION OF IMMUNE SYSTEM PROCESS | IL4 | 14665 | -0,16649385 | -0,3936276 | TUMOR | Smokers |
| GSE10072 | REGULATION OF IMMUNE SYSTEM PROCESS | IL4 | 11776 | -0,39500636 | -0,34416014 | NORMAL | Non-Smokers |
| GSE50081 | IMMUNE_RESPONSE | IL4R | 20464 | -0,29399323 | -0,035635054 | TUMOR | Smokers |
| GSE32863 | IMMUNE_RESPONSE | IL4R | 35416 | -0,46966392 | -0,19558024 | TUMOR | Non-Smokers |
| TCGA | IMMUNE_RESPONSE | IL4R | 17451 | -0,08173231 | -0,31568053 | TUMOR | Smokers |
| GSE50081 | IMMUNE SYSTEM PROCESS | IL4R | 20464 | -0,29399323 | -0,024430694 | TUMOR | Smokers |
| GSE32863 | IMMUNE SYSTEM PROCESS | IL4R | 35416 | -0,46966392 | -0,18791755 | TUMOR | Non-Smokers |
| TCGA | IMMUNE SYSTEM PROCESS | IL4R | 17451 | -0,08173231 | -0,28333148 | TUMOR | Smokers |
| GSE32863 | DEFENSE RESPONSE | IL5 | 35793 | -0,46481332 | -0,15253678 | TUMOR | Smokers |
| GSE50081 | IMMUNE_RESPONSE | IL6 | 19566 | -0,19204009 | -0,19946356 | TUMOR | Smokers |
| GSE32863 | IMMUNE_RESPONSE | IL6 | 28583 | -0,18084992 | -0,4369893 | TUMOR | Smokers |
| GSE50081 | IMMUNE SYSTEM PROCESS | IL6 | 19566 | -0,19204009 | -0,14891374 | TUMOR | Smokers |
| GSE32863 | IMMUNE SYSTEM PROCESS | IL6 | 28583 | -0,18084992 | -0,4112861 | TUMOR | Smokers |
| TCGA | IMMUNE_RESPONSE | IL6R | 17961 | -0,29666087 | -0,1866223 | TUMOR | Non-Smokers |
| TCGA | IMMUNE SYSTEM PROCESS | IL6R | 17961 | -0,29666087 | -0,20905298 | TUMOR | Non-Smokers |
| GSE32863 | IMMUNE_RESPONSE | IL7 | 36475 | -0,6629408 | -0,022309965 | TUMOR | Non-Smokers |
| GSE32863 | IMMUNE SYSTEM PROCESS | IL7 | 36475 | -0,6629408 | -0,047096044 | TUMOR | Non-Smokers |
| GSE32863 | IMMUNE SYSTEM PROCESS | IL7 | 28107 | -0,17108992 | -0,41797814 | TUMOR | Smokers |
| TCGA | IMMUNE SYSTEM PROCESS | IL7 | 14670 | -0,04817061 | -0,36799905 | TUMOR | Smokers |
| GSE32863 | REGULATION OF IMMUNE SYSTEM PROCESS | IL7 | 28107 | -0,17108992 | -0,40570718 | TUMOR | Smokers |
| GSE50081 | IMMUNE_RESPONSE | IL7R | 17037 | -0,10661183 | -0,40993622 | TUMOR | Smokers |
| GSE47115 | IMMUNE_RESPONSE | IL7R | 18156 | -0,33157313 | -0,35305262 | TUMOR | Smokers |
| GSE32863 | IMMUNE_RESPONSE | IL7R | 36647 | -0,82418543 | 0,001508068 | TUMOR | Non-Smokers |
| TCGA | IMMUNE_RESPONSE | IL7R | 18658 | -0,10330164 | -0,2170867 | TUMOR | Smokers |
| GSE50081 | IMMUNE SYSTEM PROCESS | IL7R | 17037 | -0,10661183 | -0,35579383 | TUMOR | Smokers |
| GSE47115 | IMMUNE SYSTEM PROCESS | IL7R | 18156 | -0,33157313 | -0,34160712 | TUMOR | Smokers |
| GSE32863 | IMMUNE SYSTEM PROCESS | IL7R | 36647 | -0,82418543 | -0,013516211 | TUMOR | Non-Smokers |
| TCGA | IMMUNE SYSTEM PROCESS | IL7R | 18658 | -0,10330164 | -0,18878472 | TUMOR | Smokers |
| GSE32863 | IMMUNE SYSTEM PROCESS | IL8 | 30435 | -0,24903992 | -0,34668782 | TUMOR | Non-Smokers |
| GSE32863 | DEFENSE RESPONSE | IL8 | 30435 | -0,24903992 | -0,30786285 | TUMOR | Non-Smokers |
| GSE32863 | IMMUNE SYSTEM PROCESS | INHA | 28595 | -0,20156905 | -0,380328 | TUMOR | Non-Smokers |
| TCGA | IMMUNE SYSTEM PROCESS | INHA | 18089 | -0,30390587 | -0,19983277 | TUMOR | Non-Smokers |
| TCGA | REGULATION OF IMMUNE SYSTEM PROCESS | INHA | 18089 | -0,30390587 | -0,21730344 | TUMOR | Non-Smokers |
| GSE47115 | IMMUNE SYSTEM PROCESS | INHBA | 18396 | -0,34609386 | -0,32728878 | TUMOR | Smokers |
| GSE32863 | IMMUNE SYSTEM PROCESS | INHBA | 32239 | -0,30465138 | -0,31684566 | TUMOR | Non-Smokers |
| GSE32863 | IMMUNE SYSTEM PROCESS | INHBA | 33979 | -0,33757061 | -0,3133101 | TUMOR | Smokers |
| TCGA | IMMUNE SYSTEM PROCESS | INHBA | 18329 | -0,09661731 | -0,22975513 | TUMOR | Smokers |
| GSE47115 | DEFENSE RESPONSE | INHBA | 18396 | -0,34609386 | -0,23187782 | TUMOR | Smokers |
| GSE32863 | DEFENSE RESPONSE | INHBA | 32239 | -0,30465138 | -0,28106782 | TUMOR | Non-Smokers |
| GSE32863 | DEFENSE RESPONSE | INHBA | 33979 | -0,33757061 | -0,30620486 | TUMOR | Smokers |
| TCGA | DEFENSE RESPONSE | INHBA | 18329 | -0,09661731 | -0,24079832 | TUMOR | Smokers |
| GSE47115 | REGULATION OF IMMUNE SYSTEM PROCESS | INHBA | 18396 | -0,34609386 | -0,3180662 | TUMOR | Smokers |
| GSE32863 | REGULATION OF IMMUNE SYSTEM PROCESS | INHBA | 33979 | -0,33757061 | -0,29538298 | TUMOR | Smokers |
| TCGA | DEFENSE RESPONSE | INHBB | 19813 | -0,13846894 | -0,11179789 | TUMOR | Smokers |
| TCGA | IMMUNE SYSTEM PROCESS | INS | 16551 | -0,06936646 | -0,33651546 | TUMOR | Smokers |
| GSE32863 | DEFENSE RESPONSE | IRAK2 | 33894 | -0,37220043 | -0,23248033 | TUMOR | Non-Smokers |
| GSE32863 | DEFENSE RESPONSE | IRAK2 | 30682 | -0,22901754 | -0,38685313 | TUMOR | Smokers |
| GSE50081 | IMMUNE_RESPONSE | IRF8 | 17362 | -0,11402538 | -0,40232188 | TUMOR | Smokers |
| GSE32863 | IMMUNE_RESPONSE | IRF8 | 32428 | -0,31162778 | -0,29571435 | TUMOR | Non-Smokers |
| GSE32863 | IMMUNE_RESPONSE | IRF8 | 35921 | -0,48375916 | -0,17349482 | TUMOR | Smokers |
| TCGA | IMMUNE_RESPONSE | IRF8 | 17324 | -0,07987975 | -0,33588538 | TUMOR | Smokers |
| TCGA | IMMUNE_RESPONSE | IRF8 | 16525 | -0,23103377 | -0,29565412 | TUMOR | Non-Smokers |
| GSE50081 | IMMUNE SYSTEM PROCESS | IRF8 | 17362 | -0,11402538 | -0,35439104 | TUMOR | Smokers |
| GSE32863 | IMMUNE SYSTEM PROCESS | IRF8 | 32428 | -0,31162778 | -0,29767674 | TUMOR | Non-Smokers |
| GSE32863 | IMMUNE SYSTEM PROCESS | IRF8 | 35921 | -0,48375916 | -0,1448507 | TUMOR | Smokers |
| TCGA | IMMUNE SYSTEM PROCESS | IRF8 | 17324 | -0,07987975 | -0,29631346 | TUMOR | Smokers |
| TCGA | IMMUNE SYSTEM PROCESS | IRF8 | 16525 | -0,23103377 | -0,30307108 | TUMOR | Non-Smokers |
| GSE50081 | IMMUNE SYSTEM PROCESS | ITGB2 | 19311 | -0,17805581 | -0,15911047 | TUMOR | Smokers |
| GSE47115 | IMMUNE SYSTEM PROCESS | ITGB2 | 18301 | -0,33959469 | -0,33149308 | TUMOR | Smokers |
| GSE32863 | IMMUNE SYSTEM PROCESS | ITGB2 | 31430 | -0,27846852 | -0,3290407 | TUMOR | Non-Smokers |
| GSE32863 | IMMUNE SYSTEM PROCESS | ITGB2 | 36028 | -0,49755579 | -0,13405606 | TUMOR | Smokers |
| TCGA | IMMUNE SYSTEM PROCESS | ITGB2 | 15200 | -0,05362329 | -0,37460005 | TUMOR | Smokers |
| GSE50081 | CELLULAR DEFENSE RESPONSE | ITK | 17321 | -0,11278625 | -0,3868705 | TUMOR | Smokers |
| GSE47115 | CELLULAR DEFENSE RESPONSE | ITK | 17527 | -0,29566747 | -0,28363225 | TUMOR | Smokers |
| GSE32863 | CELLULAR DEFENSE RESPONSE | ITK | 33071 | -0,33631805 | -0,3013236 | TUMOR | Non-Smokers |
| GSE32863 | CELLULAR DEFENSE RESPONSE | ITK | 35286 | -0,41731831 | -0,37801465 | TUMOR | Smokers |
| TCGA | CELLULAR DEFENSE RESPONSE | ITK | 17837 | -0,08801709 | -0,13058856 | TUMOR | Smokers |
| GSE50081 | DEFENSE RESPONSE | ITK | 17321 | -0,11278625 | -0,41873202 | TUMOR | Smokers |
| GSE47115 | DEFENSE RESPONSE | ITK | 17527 | -0,29566747 | -0,26414824 | TUMOR | Smokers |
| GSE32863 | DEFENSE RESPONSE | ITK | 33071 | -0,33631805 | -0,25355506 | TUMOR | Non-Smokers |
| GSE32863 | DEFENSE RESPONSE | ITK | 35286 | -0,41731831 | -0,24884464 | TUMOR | Smokers |
| TCGA | DEFENSE RESPONSE | ITK | 17837 | -0,08801709 | -0,2784292 | TUMOR | Smokers |
| GSE47115 | DEFENSE RESPONSE | KCNN4 | 18393 | -0,34585556 | -0,24393362 | TUMOR | Smokers |
| GSE32863 | DEFENSE RESPONSE | KCNN4 | 34478 | -0,40164337 | -0,19501284 | TUMOR | Non-Smokers |
| GSE32863 | DEFENSE RESPONSE | KCNN4 | 36111 | -0,50926584 | -0,1286085 | TUMOR | Smokers |
| TCGA | DEFENSE RESPONSE | KCNN4 | 20161 | -0,16741718 | -0,025893064 | TUMOR | Smokers |
| GSE47115 | IMMUNE_RESPONSE | KIR2DL1 | 16875 | -0,26172981 | -0,40231955 | TUMOR | Smokers |
| GSE32863 | IMMUNE_RESPONSE | KIR2DL1 | 30108 | -0,2398635 | -0,34843537 | TUMOR | Non-Smokers |
| TCGA | IMMUNE_RESPONSE | KIR2DL1 | 16460 | -0,06811184 | -0,37649637 | TUMOR | Smokers |
| TCGA | IMMUNE_RESPONSE | KIR2DL1 | 19413 | -0,42372513 | -0,08849326 | TUMOR | Non-Smokers |
| GSE47115 | IMMUNE SYSTEM PROCESS | KIR2DL1 | 16875 | -0,26172981 | -0,3707593 | TUMOR | Smokers |
| GSE32863 | IMMUNE SYSTEM PROCESS | KIR2DL1 | 30108 | -0,2398635 | -0,36343715 | TUMOR | Non-Smokers |
| TCGA | IMMUNE SYSTEM PROCESS | KIR2DL1 | 16460 | -0,06811184 | -0,33528656 | TUMOR | Smokers |
| TCGA | IMMUNE SYSTEM PROCESS | KIR2DL1 | 19413 | -0,42372513 | -0,0811826 | TUMOR | Non-Smokers |
| GSE32863 | IMMUNE_RESPONSE | KIR2DL3 | 27869 | -0,18439615 | -0,3663447 | TUMOR | Non-Smokers |
| GSE32863 | IMMUNE_RESPONSE | KIR2DL3 | 31693 | -0,25526673 | -0,39828104 | TUMOR | Smokers |
| TCGA | IMMUNE_RESPONSE | KIR2DL3 | 15521 | -0,19630779 | -0,35208943 | TUMOR | Non-Smokers |
| GSE32863 | IMMUNE SYSTEM PROCESS | KIR2DL3 | 27869 | -0,18439615 | -0,38845605 | TUMOR | Non-Smokers |
| GSE32863 | IMMUNE SYSTEM PROCESS | KIR2DL3 | 31693 | -0,25526673 | -0,36829284 | TUMOR | Smokers |
| TCGA | IMMUNE SYSTEM PROCESS | KIR2DL3 | 15521 | -0,19630779 | -0,35368156 | TUMOR | Non-Smokers |
| GSE47115 | CELLULAR DEFENSE RESPONSE | KIR2DL4 | 16729 | -0,25493503 | -0,3495051 | TUMOR | Smokers |
| GSE32863 | CELLULAR DEFENSE RESPONSE | KIR2DL4 | 29015 | -0,21162781 | -0,3645146 | TUMOR | Non-Smokers |
| TCGA | CELLULAR DEFENSE RESPONSE | KIR2DL4 | 14793 | -0,04955507 | -0,39596632 | TUMOR | Smokers |
| GSE47115 | DEFENSE RESPONSE | KIR2DL4 | 16729 | -0,25493503 | -0,32682812 | TUMOR | Smokers |
| GSE32863 | DEFENSE RESPONSE | KIR2DL4 | 29015 | -0,21162781 | -0,35343185 | TUMOR | Non-Smokers |
| TCGA | DEFENSE RESPONSE | KIR2DL4 | 14793 | -0,04955507 | -0,41840273 | TUMOR | Smokers |
| GSE47115 | CELLULAR DEFENSE RESPONSE | KIR2DS3 | 13948 | -0,13862048 | -0,4023681 | TUMOR | Smokers |
| GSE32863 | CELLULAR DEFENSE RESPONSE | KIR2DS3 | 31599 | -0,28346738 | -0,31301925 | TUMOR | Non-Smokers |
| GSE32863 | DEFENSE RESPONSE | KIR2DS3 | 31599 | -0,28346738 | -0,29401854 | TUMOR | Non-Smokers |
| GSE47115 | CELLULAR DEFENSE RESPONSE | KIR3DL2 | 14693 | -0,16783346 | -0,39214262 | TUMOR | Smokers |
| TCGA | CELLULAR DEFENSE RESPONSE | KIR3DL2 | 16081 | -0,06356475 | -0,33650944 | TUMOR | Smokers |
| GSE32863 | DEFENSE RESPONSE | KIR3DL2 | 29694 | -0,20489222 | -0,40369323 | TUMOR | Smokers |
| TCGA | DEFENSE RESPONSE | KIR3DL2 | 16081 | -0,06356475 | -0,394999 | TUMOR | Smokers |
| GSE47115 | IMMUNE SYSTEM PROCESS | KIRREL3 | 19752 | -0,47184542 | -0,26278704 | TUMOR | Smokers |
| TCGA | IMMUNE SYSTEM PROCESS | KIRREL3 | 20108 | -0,16121554 | -0,023125604 | TUMOR | Smokers |
| GSE32863 | CELLULAR DEFENSE RESPONSE | KLRC2 | 27780 | -0,18219967 | -0,42600593 | TUMOR | Non-Smokers |
| GSE32863 | CELLULAR DEFENSE RESPONSE | KLRC2 | 31721 | -0,25595906 | -0,48134014 | TUMOR | Smokers |
| GSE32863 | DEFENSE RESPONSE | KLRC2 | 27780 | -0,18219967 | -0,37372684 | TUMOR | Non-Smokers |
| GSE32863 | DEFENSE RESPONSE | KLRC2 | 31721 | -0,25595906 | -0,37085995 | TUMOR | Smokers |
| GSE50081 | CELLULAR DEFENSE RESPONSE | KLRC3 | 17072 | -0,10735983 | -0,41472822 | TUMOR | Smokers |
| GSE32863 | CELLULAR DEFENSE RESPONSE | KLRC3 | 35477 | -0,43163726 | -0,32325494 | TUMOR | Smokers |
| GSE50081 | DEFENSE RESPONSE | KLRC3 | 17072 | -0,10735983 | -0,42383394 | TUMOR | Smokers |
| GSE32863 | DEFENSE RESPONSE | KLRC3 | 35477 | -0,43163726 | -0,21877883 | TUMOR | Smokers |
| GSE50081 | CELLULAR DEFENSE RESPONSE | KLRC4 | 19432 | -0,18432267 | -0,14783296 | TUMOR | Smokers |
| TCGA | CELLULAR DEFENSE RESPONSE | KLRC4 | 15328 | -0,05500067 | -0,35611528 | TUMOR | Smokers |
| GSE50081 | DEFENSE RESPONSE | KLRC4 | 19432 | -0,18432267 | -0,28760445 | TUMOR | Smokers |
| TCGA | DEFENSE RESPONSE | KLRC4 | 15328 | -0,05500067 | -0,40645814 | TUMOR | Smokers |
| GSE47115 | CELLULAR DEFENSE RESPONSE | KLRG1 | 15599 | -0,20460582 | -0,36503613 | TUMOR | Smokers |
| GSE32863 | CELLULAR DEFENSE RESPONSE | KLRG1 | 35065 | -0,43974173 | -0,16156329 | TUMOR | Non-Smokers |
| GSE47115 | DEFENSE RESPONSE | KLRG1 | 15599 | -0,20460582 | -0,33763978 | TUMOR | Smokers |
| GSE32863 | DEFENSE RESPONSE | KLRG1 | 35065 | -0,43974173 | -0,13251548 | TUMOR | Non-Smokers |
| GSE50081 | DEFENSE RESPONSE | KNG1 | 16950 | -0,10448965 | -0,42614284 | TUMOR | Smokers |
| GSE47115 | DEFENSE RESPONSE | KNG1 | 17466 | -0,29272476 | -0,26638702 | TUMOR | Smokers |
| GSE50081 | IMMUNE_RESPONSE | KRT1 | 16280 | -0,08936449 | -0,41002157 | TUMOR | Smokers |
| GSE32863 | IMMUNE_RESPONSE | KRT1 | 33158 | -0,33981061 | -0,29149666 | TUMOR | Non-Smokers |
| GSE50081 | IMMUNE SYSTEM PROCESS | KRT1 | 16280 | -0,08936449 | -0,3583353 | TUMOR | Smokers |
| GSE32863 | IMMUNE SYSTEM PROCESS | KRT1 | 33158 | -0,33981061 | -0,28361613 | TUMOR | Non-Smokers |
| GSE32863 | DEFENSE RESPONSE | KRT1 | 33158 | -0,33981061 | -0,25009507 | TUMOR | Non-Smokers |
| GSE50081 | REGULATION OF IMMUNE SYSTEM PROCESS | KRT1 | 16280 | -0,08936449 | -0,46682465 | TUMOR | Smokers |
| GSE32863 | DEFENSE RESPONSE | LALBA | 33502 | -0,31639627 | -0,32603502 | TUMOR | Smokers |
| GSE50081 | IMMUNE_RESPONSE | LAT | 17370 | -0,11417565 | -0,39310712 | TUMOR | Smokers |
| GSE47115 | IMMUNE_RESPONSE | LAT | 19828 | -0,48271644 | -0,25446555 | TUMOR | Smokers |
| GSE32863 | IMMUNE_RESPONSE | LAT | 30604 | -0,25401187 | -0,3203353 | TUMOR | Non-Smokers |
| GSE32863 | IMMUNE_RESPONSE | LAT | 36405 | -0,57912856 | -0,1171022 | TUMOR | Smokers |
| TCGA | IMMUNE_RESPONSE | LAT | 16895 | -0,07392161 | -0,36991602 | TUMOR | Smokers |
| TCGA | IMMUNE_RESPONSE | LAT | 15170 | -0,18536049 | -0,35857996 | TUMOR | Non-Smokers |
| GSE50081 | IMMUNE SYSTEM PROCESS | LAT | 17370 | -0,11417565 | -0,3476835 | TUMOR | Smokers |
| GSE47115 | IMMUNE SYSTEM PROCESS | LAT | 19828 | -0,48271644 | -0,23600657 | TUMOR | Smokers |
| GSE32863 | IMMUNE SYSTEM PROCESS | LAT | 30604 | -0,25401187 | -0,34134817 | TUMOR | Non-Smokers |
| GSE32863 | IMMUNE SYSTEM PROCESS | LAT | 36405 | -0,57912856 | -0,08480024 | TUMOR | Smokers |
| TCGA | IMMUNE SYSTEM PROCESS | LAT | 16895 | -0,07392161 | -0,3297845 | TUMOR | Smokers |
| TCGA | IMMUNE SYSTEM PROCESS | LAT | 15170 | -0,18536049 | -0,35617188 | TUMOR | Non-Smokers |
| GSE47115 | REGULATION OF IMMUNE SYSTEM PROCESS | LAT | 19828 | -0,48271644 | -0,23351197 | TUMOR | Smokers |
| GSE10072 | REGULATION OF IMMUNE SYSTEM PROCESS | LAT | 13152 | -0,72180808 | 0,012663974 | NORMAL | Non-Smokers |
| GSE32863 | REGULATION OF IMMUNE SYSTEM PROCESS | LAT | 36405 | -0,57912856 | -0,075344905 | TUMOR | Smokers |
| TCGA | REGULATION OF IMMUNE SYSTEM PROCESS | LAT | 15170 | -0,18536049 | -0,3919595 | TUMOR | Non-Smokers |
| GSE50081 | IMMUNE_RESPONSE | LAT2 | 18929 | -0,16068736 | -0,24880956 | TUMOR | Smokers |
| GSE47115 | IMMUNE_RESPONSE | LAT2 | 17620 | -0,30097213 | -0,3719783 | TUMOR | Smokers |
| GSE32863 | IMMUNE_RESPONSE | LAT2 | 31942 | -0,29471555 | -0,29979792 | TUMOR | Non-Smokers |
| GSE32863 | IMMUNE_RESPONSE | LAT2 | 36494 | -0,61152703 | -0,096821144 | TUMOR | Smokers |
| TCGA | IMMUNE_RESPONSE | LAT2 | 19027 | -0,11203656 | -0,17169163 | TUMOR | Smokers |
| GSE50081 | IMMUNE SYSTEM PROCESS | LAT2 | 18929 | -0,16068736 | -0,22386304 | TUMOR | Smokers |
| GSE47115 | IMMUNE SYSTEM PROCESS | LAT2 | 17620 | -0,30097213 | -0,3519698 | TUMOR | Smokers |
| GSE32863 | IMMUNE SYSTEM PROCESS | LAT2 | 31942 | -0,29471555 | -0,31665727 | TUMOR | Non-Smokers |
| GSE32863 | IMMUNE SYSTEM PROCESS | LAT2 | 36494 | -0,61152703 | -0,07038024 | TUMOR | Smokers |
| TCGA | IMMUNE SYSTEM PROCESS | LAT2 | 19027 | -0,11203656 | -0,15573949 | TUMOR | Smokers |
| GSE50081 | REGULATION OF IMMUNE SYSTEM PROCESS | LAT2 | 18929 | -0,16068736 | -0,24117835 | TUMOR | Smokers |
| GSE47115 | REGULATION OF IMMUNE SYSTEM PROCESS | LAT2 | 17620 | -0,30097213 | -0,35919547 | TUMOR | Smokers |
| GSE10072 | REGULATION OF IMMUNE SYSTEM PROCESS | LAT2 | 12366 | -0,47327101 | -0,19753475 | NORMAL | Non-Smokers |
| GSE32863 | REGULATION OF IMMUNE SYSTEM PROCESS | LAT2 | 36494 | -0,61152703 | -0,0390269 | TUMOR | Smokers |
| GSE50081 | IMMUNE_RESPONSE | LAX1 | 18368 | -0,14179316 | -0,3314709 | TUMOR | Smokers |
| GSE32863 | IMMUNE_RESPONSE | LAX1 | 33802 | -0,36753333 | -0,27566347 | TUMOR | Non-Smokers |
| TCGA | IMMUNE_RESPONSE | LAX1 | 16406 | -0,06740408 | -0,38273048 | TUMOR | Smokers |
| TCGA | IMMUNE_RESPONSE | LAX1 | 16231 | -0,21983947 | -0,32556593 | TUMOR | Non-Smokers |
| GSE50081 | IMMUNE SYSTEM PROCESS | LAX1 | 18368 | -0,14179316 | -0,2917849 | TUMOR | Smokers |
| GSE32863 | IMMUNE SYSTEM PROCESS | LAX1 | 33802 | -0,36753333 | -0,2687074 | TUMOR | Non-Smokers |
| TCGA | IMMUNE SYSTEM PROCESS | LAX1 | 16406 | -0,06740408 | -0,34234536 | TUMOR | Smokers |
| TCGA | IMMUNE SYSTEM PROCESS | LAX1 | 16231 | -0,21983947 | -0,33146498 | TUMOR | Non-Smokers |
| GSE10072 | REGULATION OF IMMUNE SYSTEM PROCESS | LAX1 | 11825 | -0,40091869 | -0,318288 | NORMAL | Non-Smokers |
| TCGA | REGULATION OF IMMUNE SYSTEM PROCESS | LAX1 | 16231 | -0,21983947 | -0,40975454 | TUMOR | Non-Smokers |
| GSE50081 | CELLULAR DEFENSE RESPONSE | LBP | 18516 | -0,14703582 | -0,27740914 | TUMOR | Smokers |
| GSE47115 | CELLULAR DEFENSE RESPONSE | LBP | 16240 | -0,23123084 | -0,36329705 | TUMOR | Smokers |
| GSE32863 | CELLULAR DEFENSE RESPONSE | LBP | 30186 | -0,24216025 | -0,3584059 | TUMOR | Non-Smokers |
| GSE50081 | DEFENSE RESPONSE | LBP | 18516 | -0,14703582 | -0,37233773 | TUMOR | Smokers |
| GSE47115 | DEFENSE RESPONSE | LBP | 16240 | -0,23123084 | -0,3456578 | TUMOR | Smokers |
| GSE32863 | DEFENSE RESPONSE | LBP | 30186 | -0,24216025 | -0,3307231 | TUMOR | Non-Smokers |
| GSE50081 | IMMUNE SYSTEM PROCESS | LCK | 17630 | -0,12069941 | -0,33119613 | TUMOR | Smokers |
| GSE47115 | IMMUNE SYSTEM PROCESS | LCK | 20505 | -0,66562283 | -0,08023301 | TUMOR | Smokers |
| GSE32863 | IMMUNE SYSTEM PROCESS | LCK | 32255 | -0,3053363 | -0,31327596 | TUMOR | Non-Smokers |
| GSE47115 | REGULATION OF IMMUNE SYSTEM PROCESS | LCK | 20505 | -0,66562283 | 0,010601362 | TUMOR | Smokers |
| GSE32863 | REGULATION OF IMMUNE SYSTEM PROCESS | LCK | 27753 | -0,16375627 | -0,40690473 | TUMOR | Smokers |
| GSE50081 | IMMUNE_RESPONSE | LCP2 | 19441 | -0,18486968 | -0,20138113 | TUMOR | Smokers |
| GSE47115 | IMMUNE_RESPONSE | LCP2 | 19508 | -0,4438481 | -0,28838092 | TUMOR | Smokers |
| TCGA | IMMUNE_RESPONSE | LCP2 | 15755 | -0,05987126 | -0,41605267 | TUMOR | Smokers |
| GSE50081 | IMMUNE SYSTEM PROCESS | LCP2 | 19441 | -0,18486968 | -0,14869656 | TUMOR | Smokers |
| GSE47115 | IMMUNE SYSTEM PROCESS | LCP2 | 19508 | -0,4438481 | -0,2799798 | TUMOR | Smokers |
| TCGA | IMMUNE SYSTEM PROCESS | LCP2 | 15755 | -0,05987126 | -0,3641559 | TUMOR | Smokers |
| GSE50081 | IMMUNE SYSTEM PROCESS | LDB1 | 20023 | -0,22206675 | -0,11336499 | TUMOR | Smokers |
| GSE32863 | IMMUNE SYSTEM PROCESS | LDB1 | 35576 | -0,44281644 | -0,16130494 | TUMOR | Smokers |
| GSE32863 | DEFENSE RESPONSE | LGALS3BP | 29138 | -0,1927013 | -0,41494516 | TUMOR | Smokers |
| GSE32863 | IMMUNE SYSTEM PROCESS | LIG1 | 30042 | -0,23799214 | -0,36477864 | TUMOR | Non-Smokers |
| TCGA | IMMUNE SYSTEM PROCESS | LIG1 | 16375 | -0,22511473 | -0,31148037 | TUMOR | Non-Smokers |
| TCGA | IMMUNE SYSTEM PROCESS | LIG3 | 15522 | -0,19632021 | -0,35028833 | TUMOR | Non-Smokers |
| GSE50081 | DEFENSE RESPONSE | LILRA1 | 18592 | -0,14929688 | -0,35298863 | TUMOR | Smokers |
| GSE47115 | DEFENSE RESPONSE | LILRA1 | 18803 | -0,3772237 | -0,21398273 | TUMOR | Smokers |
| GSE50081 | DEFENSE RESPONSE | LILRA2 | 19517 | -0,18921535 | -0,28444993 | TUMOR | Smokers |
| GSE32863 | DEFENSE RESPONSE | LILRA2 | 33885 | -0,33319291 | -0,3147732 | TUMOR | Smokers |
| TCGA | DEFENSE RESPONSE | LILRA2 | 14905 | -0,05063083 | -0,41473633 | TUMOR | Smokers |
| GSE50081 | DEFENSE RESPONSE | LILRA3 | 17535 | -0,11808734 | -0,41554993 | TUMOR | Smokers |
| GSE47115 | DEFENSE RESPONSE | LILRA3 | 17097 | -0,27302551 | -0,292996 | TUMOR | Smokers |
| GSE32863 | DEFENSE RESPONSE | LILRA3 | 36328 | -0,60777855 | -2,23E-04 | TUMOR | Non-Smokers |
| GSE50081 | IMMUNE_RESPONSE | LILRB2 | 19781 | -0,20564461 | -0,18475954 | TUMOR | Smokers |
| GSE32863 | IMMUNE_RESPONSE | LILRB2 | 28386 | -0,1966389 | -0,36252666 | TUMOR | Non-Smokers |
| TCGA | IMMUNE_RESPONSE | LILRB2 | 17882 | -0,29193544 | -0,20468259 | TUMOR | Non-Smokers |
| GSE50081 | IMMUNE SYSTEM PROCESS | LILRB2 | 19781 | -0,20564461 | -0,14087047 | TUMOR | Smokers |
| GSE32863 | IMMUNE SYSTEM PROCESS | LILRB2 | 28386 | -0,1966389 | -0,38245404 | TUMOR | Non-Smokers |
| TCGA | IMMUNE SYSTEM PROCESS | LILRB2 | 17882 | -0,29193544 | -0,2204731 | TUMOR | Non-Smokers |
| GSE50081 | CELLULAR DEFENSE RESPONSE | LILRB2 | 19781 | -0,20564461 | -0,12759167 | TUMOR | Smokers |
| GSE47115 | CELLULAR DEFENSE RESPONSE | LILRB2 | 15188 | -0,1870815 | -0,37523264 | TUMOR | Smokers |
| GSE32863 | CELLULAR DEFENSE RESPONSE | LILRB2 | 28386 | -0,1966389 | -0,38138136 | TUMOR | Non-Smokers |
| GSE50081 | DEFENSE RESPONSE | LILRB2 | 19781 | -0,20564461 | -0,23566489 | TUMOR | Smokers |
| GSE47115 | DEFENSE RESPONSE | LILRB2 | 15188 | -0,1870815 | -0,3460349 | TUMOR | Smokers |
| GSE32863 | DEFENSE RESPONSE | LILRB2 | 28386 | -0,1966389 | -0,35765192 | TUMOR | Non-Smokers |
| GSE32863 | DEFENSE RESPONSE | LILRB3 | 35975 | -0,53433162 | -0,040164746 | TUMOR | Non-Smokers |
| GSE32863 | DEFENSE RESPONSE | LILRB3 | 33713 | -0,3259775 | -0,3210904 | TUMOR | Smokers |
| GSE50081 | IMMUNE SYSTEM PROCESS | LRMP | 16550 | -0,09525801 | -0,3571646 | TUMOR | Smokers |
| GSE32863 | IMMUNE SYSTEM PROCESS | LRMP | 31317 | -0,27496299 | -0,32959372 | TUMOR | Non-Smokers |
| GSE32863 | IMMUNE SYSTEM PROCESS | LRMP | 32483 | -0,27793196 | -0,3637853 | TUMOR | Smokers |
| TCGA | IMMUNE SYSTEM PROCESS | LRMP | 18086 | -0,09205259 | -0,24466516 | TUMOR | Smokers |
| GSE47115 | CELLULAR DEFENSE RESPONSE | LSP1 | 14228 | -0,14976326 | -0,3943603 | TUMOR | Smokers |
| GSE32863 | CELLULAR DEFENSE RESPONSE | LSP1 | 29684 | -0,22887582 | -0,36427477 | TUMOR | Non-Smokers |
| TCGA | CELLULAR DEFENSE RESPONSE | LSP1 | 19482 | -0,12471025 | -0,04020463 | TUMOR | Smokers |
| GSE32863 | DEFENSE RESPONSE | LSP1 | 29684 | -0,22887582 | -0,3453095 | TUMOR | Non-Smokers |
| TCGA | DEFENSE RESPONSE | LSP1 | 19482 | -0,12471025 | -0,15154451 | TUMOR | Smokers |
| GSE50081 | IMMUNE SYSTEM PROCESS | LST1 | 18948 | -0,16119373 | -0,21481246 | TUMOR | Smokers |
| TCGA | IMMUNE SYSTEM PROCESS | LST1 | 17669 | -0,08518293 | -0,2703654 | TUMOR | Smokers |
| GSE47115 | REGULATION OF IMMUNE SYSTEM PROCESS | LST1 | 16400 | -0,23934658 | -0,38107723 | TUMOR | Smokers |
| GSE50081 | IMMUNE_RESPONSE | LTB4R | 19360 | -0,17987671 | -0,20515844 | TUMOR | Smokers |
| GSE47115 | IMMUNE_RESPONSE | LTB4R | 18460 | -0,35118005 | -0,33757207 | TUMOR | Smokers |
| GSE32863 | IMMUNE_RESPONSE | LTB4R | 31457 | -0,27960587 | -0,31373644 | TUMOR | Non-Smokers |
| TCGA | IMMUNE_RESPONSE | LTB4R | 17236 | -0,07874648 | -0,3419562 | TUMOR | Smokers |
| GSE50081 | IMMUNE SYSTEM PROCESS | LTB4R | 19360 | -0,17987671 | -0,15042719 | TUMOR | Smokers |
| GSE47115 | IMMUNE SYSTEM PROCESS | LTB4R | 18460 | -0,35118005 | -0,32592583 | TUMOR | Smokers |
| GSE32863 | IMMUNE SYSTEM PROCESS | LTB4R | 31457 | -0,27960587 | -0,32610893 | TUMOR | Non-Smokers |
| TCGA | IMMUNE SYSTEM PROCESS | LTB4R | 17236 | -0,07874648 | -0,2995313 | TUMOR | Smokers |
| GSE50081 | DEFENSE RESPONSE | LTB4R | 19360 | -0,17987671 | -0,29120913 | TUMOR | Smokers |
| GSE47115 | DEFENSE RESPONSE | LTB4R | 18460 | -0,35118005 | -0,21655364 | TUMOR | Smokers |
| GSE32863 | DEFENSE RESPONSE | LTB4R | 31457 | -0,27960587 | -0,29500413 | TUMOR | Non-Smokers |
| TCGA | DEFENSE RESPONSE | LTB4R | 17236 | -0,07874648 | -0,3360921 | TUMOR | Smokers |
| GSE32863 | IMMUNE_RESPONSE | LTF | 36262 | -0,59022999 | -0,03994996 | TUMOR | Non-Smokers |
| GSE32863 | IMMUNE_RESPONSE | LTF | 31755 | -0,25670955 | -0,39514586 | TUMOR | Smokers |
| TCGA | IMMUNE_RESPONSE | LTF | 16857 | -0,07347477 | -0,37286538 | TUMOR | Smokers |
| GSE32863 | IMMUNE SYSTEM PROCESS | LTF | 36262 | -0,59022999 | -0,065968625 | TUMOR | Non-Smokers |
| GSE32863 | IMMUNE SYSTEM PROCESS | LTF | 31755 | -0,25670955 | -0,36640376 | TUMOR | Smokers |
| TCGA | IMMUNE SYSTEM PROCESS | LTF | 16857 | -0,07347477 | -0,33141395 | TUMOR | Smokers |
| GSE47115 | IMMUNE_RESPONSE | LY75 | 17168 | -0,2774727 | -0,4018473 | TUMOR | Smokers |
| GSE32863 | IMMUNE_RESPONSE | LY75 | 31823 | -0,29086921 | -0,30756852 | TUMOR | Non-Smokers |
| GSE32863 | IMMUNE_RESPONSE | LY75 | 34990 | -0,39371735 | -0,2841169 | TUMOR | Smokers |
| GSE47115 | IMMUNE SYSTEM PROCESS | LY75 | 17168 | -0,2774727 | -0,37093073 | TUMOR | Smokers |
| GSE32863 | IMMUNE SYSTEM PROCESS | LY75 | 31823 | -0,29086921 | -0,32111374 | TUMOR | Non-Smokers |
| GSE32863 | IMMUNE SYSTEM PROCESS | LY75 | 34990 | -0,39371735 | -0,23860368 | TUMOR | Smokers |
| GSE47115 | DEFENSE RESPONSE | LY75 | 17168 | -0,2774727 | -0,2818854 | TUMOR | Smokers |
| GSE32863 | DEFENSE RESPONSE | LY75 | 31823 | -0,29086921 | -0,29020354 | TUMOR | Non-Smokers |
| GSE32863 | DEFENSE RESPONSE | LY75 | 34990 | -0,39371735 | -0,26784742 | TUMOR | Smokers |
| GSE47115 | IMMUNE_RESPONSE | LY86 | 17286 | -0,2839472 | -0,3925458 | TUMOR | Smokers |
| GSE32863 | IMMUNE_RESPONSE | LY86 | 34602 | -0,3681595 | -0,31644025 | TUMOR | Smokers |
| GSE47115 | IMMUNE SYSTEM PROCESS | LY86 | 17286 | -0,2839472 | -0,36588106 | TUMOR | Smokers |
| GSE32863 | IMMUNE SYSTEM PROCESS | LY86 | 34602 | -0,3681595 | -0,28627196 | TUMOR | Smokers |
| GSE50081 | CELLULAR DEFENSE RESPONSE | LY96 | 17844 | -0,12539586 | -0,3455976 | TUMOR | Smokers |
| GSE47115 | CELLULAR DEFENSE RESPONSE | LY96 | 19627 | -0,4567363 | -0,19382769 | TUMOR | Smokers |
| GSE32863 | CELLULAR DEFENSE RESPONSE | LY96 | 36384 | -0,57194138 | -0,13781743 | TUMOR | Smokers |
| GSE50081 | DEFENSE RESPONSE | LY96 | 17844 | -0,12539586 | -0,38776454 | TUMOR | Smokers |
| GSE47115 | DEFENSE RESPONSE | LY96 | 19627 | -0,4567363 | -0,15545145 | TUMOR | Smokers |
| GSE32863 | DEFENSE RESPONSE | LY96 | 36384 | -0,57194138 | -0,10008923 | TUMOR | Smokers |
| GSE47115 | IMMUNE SYSTEM PROCESS | LYN | 18914 | -0,38866559 | -0,31987384 | TUMOR | Smokers |
| GSE32863 | IMMUNE SYSTEM PROCESS | LYN | 36128 | -0,55963171 | -0,09245206 | TUMOR | Non-Smokers |
| TCGA | IMMUNE SYSTEM PROCESS | LYN | 16719 | -0,23832227 | -0,28426182 | TUMOR | Non-Smokers |
| GSE47115 | CELLULAR DEFENSE RESPONSE | LYST | 17263 | -0,28285965 | -0,31386137 | TUMOR | Smokers |
| TCGA | CELLULAR DEFENSE RESPONSE | LYST | 17612 | -0,08425226 | -0,1999435 | TUMOR | Smokers |
| GSE47115 | DEFENSE RESPONSE | LYST | 17263 | -0,28285965 | -0,2667021 | TUMOR | Smokers |
| TCGA | DEFENSE RESPONSE | LYST | 17612 | -0,08425226 | -0,31984618 | TUMOR | Smokers |
| GSE50081 | DEFENSE RESPONSE | LYZ | 17511 | -0,11753155 | -0,41896352 | TUMOR | Smokers |
| GSE47115 | DEFENSE RESPONSE | LYZ | 18425 | -0,3482058 | -0,22103421 | TUMOR | Smokers |
| GSE32863 | DEFENSE RESPONSE | LYZ | 30302 | -0,24514745 | -0,3170364 | TUMOR | Non-Smokers |
| GSE32863 | DEFENSE RESPONSE | LYZ | 36048 | -0,50020635 | -0,13530356 | TUMOR | Smokers |
| TCGA | IMMUNE_RESPONSE | MADCAM1 | 19216 | -0,11715278 | -0,12847815 | TUMOR | Smokers |
| TCGA | IMMUNE SYSTEM PROCESS | MADCAM1 | 19216 | -0,11715278 | -0,11601725 | TUMOR | Smokers |
| GSE50081 | IMMUNE SYSTEM PROCESS | MAFB | 16163 | -0,0870242 | -0,36088148 | TUMOR | Smokers |
| GSE32863 | IMMUNE SYSTEM PROCESS | MAFB | 29810 | -0,23184979 | -0,37386498 | TUMOR | Non-Smokers |
| GSE32863 | IMMUNE SYSTEM PROCESS | MAFB | 30237 | -0,21826231 | -0,38719037 | TUMOR | Smokers |
| TCGA | IMMUNE SYSTEM PROCESS | MAFB | 15340 | -0,05514901 | -0,36587176 | TUMOR | Smokers |
| GSE32863 | IMMUNE SYSTEM PROCESS | MAL | 30316 | -0,2455792 | -0,34669214 | TUMOR | Non-Smokers |
| GSE32863 | IMMUNE SYSTEM PROCESS | MAL | 31681 | -0,25501543 | -0,37153625 | TUMOR | Smokers |
| TCGA | IMMUNE SYSTEM PROCESS | MAL | 19378 | -0,12158597 | -0,07894117 | TUMOR | Smokers |
| TCGA | IMMUNE_RESPONSE | MALT1 | 17000 | -0,07545236 | -0,36046192 | TUMOR | Smokers |
| TCGA | IMMUNE SYSTEM PROCESS | MALT1 | 17000 | -0,07545236 | -0,32078284 | TUMOR | Smokers |
| GSE47115 | REGULATION OF IMMUNE SYSTEM PROCESS | MALT1 | 14976 | -0,17905657 | -0,38783506 | TUMOR | Smokers |
| GSE50081 | IMMUNE SYSTEM PROCESS | MAP4K1 | 18885 | -0,1586367 | -0,23159195 | TUMOR | Smokers |
| GSE47115 | IMMUNE SYSTEM PROCESS | MAP4K1 | 19785 | -0,47783664 | -0,24617991 | TUMOR | Smokers |
| GSE32863 | IMMUNE SYSTEM PROCESS | MAP4K1 | 32184 | -0,30267939 | -0,31933442 | TUMOR | Non-Smokers |
| GSE32863 | IMMUNE SYSTEM PROCESS | MAP4K1 | 34682 | -0,3724837 | -0,2729084 | TUMOR | Smokers |
| TCGA | IMMUNE SYSTEM PROCESS | MAP4K1 | 19145 | -0,11530178 | -0,12906154 | TUMOR | Smokers |
| GSE32863 | IMMUNE_RESPONSE | MAP4K2 | 35885 | -0,52058798 | -0,1332959 | TUMOR | Non-Smokers |
| GSE32863 | IMMUNE_RESPONSE | MAP4K2 | 28285 | -0,17469627 | -0,44559807 | TUMOR | Smokers |
| TCGA | IMMUNE_RESPONSE | MAP4K2 | 15942 | -0,06196642 | -0,3933265 | TUMOR | Smokers |
| TCGA | IMMUNE_RESPONSE | MAP4K2 | 15099 | -0,18343461 | -0,364218 | TUMOR | Non-Smokers |
| GSE32863 | IMMUNE SYSTEM PROCESS | MAP4K2 | 35885 | -0,52058798 | -0,13547473 | TUMOR | Non-Smokers |
| GSE32863 | IMMUNE SYSTEM PROCESS | MAP4K2 | 28285 | -0,17469627 | -0,4155757 | TUMOR | Smokers |
| TCGA | IMMUNE SYSTEM PROCESS | MAP4K2 | 15942 | -0,06196642 | -0,35013196 | TUMOR | Smokers |
| TCGA | IMMUNE SYSTEM PROCESS | MAP4K2 | 15099 | -0,18343461 | -0,35903004 | TUMOR | Non-Smokers |
| TCGA | IMMUNE_RESPONSE | MBL2 | 19253 | -0,11798663 | -0,107322074 | TUMOR | Smokers |
| TCGA | IMMUNE SYSTEM PROCESS | MBL2 | 19253 | -0,11798663 | -0,095549 | TUMOR | Smokers |
| TCGA | DEFENSE RESPONSE | MBL2 | 19253 | -0,11798663 | -0,16250035 | TUMOR | Smokers |
| GSE50081 | DEFENSE RESPONSE | MEFV | 19745 | -0,20320226 | -0,24973983 | TUMOR | Smokers |
| TCGA | DEFENSE RESPONSE | MEFV | 14657 | -0,0480744 | -0,41468105 | TUMOR | Smokers |
| TCGA | DEFENSE RESPONSE | MGLL | 19806 | -0,13810164 | -0,11986378 | TUMOR | Smokers |
| GSE32863 | IMMUNE SYSTEM PROCESS | MIA3 | 28186 | -0,17287642 | -0,41530854 | TUMOR | Smokers |
| GSE32863 | REGULATION OF IMMUNE SYSTEM PROCESS | MIA3 | 28186 | -0,17287642 | -0,39689022 | TUMOR | Smokers |
| GSE50081 | CELLULAR DEFENSE RESPONSE | MICA | 16833 | -0,10198031 | -0,44138986 | TUMOR | Smokers |
| GSE32863 | CELLULAR DEFENSE RESPONSE | MICA | 32863 | -0,29132664 | -0,43352762 | TUMOR | Smokers |
| TCGA | CELLULAR DEFENSE RESPONSE | MICA | 16458 | -0,06809802 | -0,31395248 | TUMOR | Smokers |
| GSE32863 | DEFENSE RESPONSE | MICA | 32863 | -0,29132664 | -0,3386499 | TUMOR | Smokers |
| TCGA | DEFENSE RESPONSE | MICA | 16458 | -0,06809802 | -0,3696792 | TUMOR | Smokers |
| GSE50081 | CELLULAR DEFENSE RESPONSE | MICB | 18617 | -0,15015726 | -0,22811912 | TUMOR | Smokers |
| GSE47115 | CELLULAR DEFENSE RESPONSE | MICB | 20109 | -0,53610539 | -0,1054636 | TUMOR | Smokers |
| GSE32863 | CELLULAR DEFENSE RESPONSE | MICB | 27761 | -0,18192363 | -0,45494407 | TUMOR | Non-Smokers |
| TCGA | CELLULAR DEFENSE RESPONSE | MICB | 16805 | -0,07277007 | -0,28608507 | TUMOR | Smokers |
| GSE50081 | DEFENSE RESPONSE | MICB | 18617 | -0,15015726 | -0,34839037 | TUMOR | Smokers |
| GSE47115 | DEFENSE RESPONSE | MICB | 20109 | -0,53610539 | -0,1079949 | TUMOR | Smokers |
| GSE32863 | DEFENSE RESPONSE | MICB | 27761 | -0,18192363 | -0,37950116 | TUMOR | Non-Smokers |
| TCGA | DEFENSE RESPONSE | MICB | 16805 | -0,07277007 | -0,3565885 | TUMOR | Smokers |
| GSE47115 | DEFENSE RESPONSE | MLF2 | 16260 | -0,23227237 | -0,33431613 | TUMOR | Smokers |
| GSE32863 | IMMUNE SYSTEM PROCESS | MMP9 | 36699 | -1,15022099 | 8,24509E-05 | TUMOR | Non-Smokers |
| GSE32863 | IMMUNE SYSTEM PROCESS | MMP9 | 28137 | -0,17168538 | -0,41639048 | TUMOR | Smokers |
| TCGA | IMMUNE SYSTEM PROCESS | MMP9 | 16615 | -0,07017861 | -0,32969058 | TUMOR | Smokers |
| GSE50081 | CELLULAR DEFENSE RESPONSE | MNDA | 19171 | -0,17128167 | -0,16850136 | TUMOR | Smokers |
| TCGA | CELLULAR DEFENSE RESPONSE | MNDA | 15731 | -0,05961191 | -0,35750443 | TUMOR | Smokers |
| GSE50081 | DEFENSE RESPONSE | MNDA | 19171 | -0,17128167 | -0,3023386 | TUMOR | Smokers |
| TCGA | DEFENSE RESPONSE | MNDA | 15731 | -0,05961191 | -0,41925246 | TUMOR | Smokers |
| GSE47115 | IMMUNE_RESPONSE | MNX1 | 17793 | -0,31090277 | -0,3639835 | TUMOR | Smokers |
| TCGA | IMMUNE_RESPONSE | MNX1 | 18557 | -0,10129273 | -0,23884149 | TUMOR | Smokers |
| GSE47115 | IMMUNE SYSTEM PROCESS | MNX1 | 17793 | -0,31090277 | -0,3486294 | TUMOR | Smokers |
| TCGA | IMMUNE SYSTEM PROCESS | MNX1 | 18557 | -0,10129273 | -0,20323801 | TUMOR | Smokers |
| TCGA | DEFENSE RESPONSE | MPO | 17825 | -0,08776157 | -0,2831988 | TUMOR | Smokers |
| GSE47115 | IMMUNE_RESPONSE | MR1 | 17452 | -0,29231721 | -0,38516885 | TUMOR | Smokers |
| GSE32863 | IMMUNE_RESPONSE | MR1 | 28442 | -0,17754239 | -0,43653765 | TUMOR | Smokers |
| GSE47115 | IMMUNE SYSTEM PROCESS | MR1 | 17452 | -0,29231721 | -0,36288503 | TUMOR | Smokers |
| GSE32863 | IMMUNE SYSTEM PROCESS | MR1 | 28442 | -0,17754239 | -0,4099499 | TUMOR | Smokers |
| GSE47115 | IMMUNE_RESPONSE | MS4A1 | 16836 | -0,25993457 | -0,40977034 | TUMOR | Smokers |
| GSE32863 | IMMUNE_RESPONSE | MS4A1 | 29240 | -0,21698263 | -0,3508518 | TUMOR | Non-Smokers |
| TCGA | IMMUNE_RESPONSE | MS4A1 | 18282 | -0,09574739 | -0,27079383 | TUMOR | Smokers |
| GSE47115 | IMMUNE SYSTEM PROCESS | MS4A1 | 16836 | -0,25993457 | -0,3755787 | TUMOR | Smokers |
| GSE32863 | IMMUNE SYSTEM PROCESS | MS4A1 | 29240 | -0,21698263 | -0,37314457 | TUMOR | Non-Smokers |
| TCGA | IMMUNE SYSTEM PROCESS | MS4A1 | 18282 | -0,09574739 | -0,23200262 | TUMOR | Smokers |
| TCGA | IMMUNE_RESPONSE | MS4A2 | 19225 | -0,11740267 | -0,12127331 | TUMOR | Smokers |
| TCGA | IMMUNE SYSTEM PROCESS | MS4A2 | 19225 | -0,11740267 | -0,11089672 | TUMOR | Smokers |
| GSE32863 | DEFENSE RESPONSE | MST1R | 35488 | -0,43265128 | -0,21191992 | TUMOR | Smokers |
| GSE47115 | DEFENSE RESPONSE | MX1 | 18395 | -0,34596455 | -0,23793128 | TUMOR | Smokers |
| GSE32863 | DEFENSE RESPONSE | MX1 | 35977 | -0,53454596 | -0,031036755 | TUMOR | Non-Smokers |
| TCGA | DEFENSE RESPONSE | MX1 | 17650 | -0,08493197 | -0,30623296 | TUMOR | Smokers |
| GSE47115 | DEFENSE RESPONSE | MX2 | 20094 | -0,53272843 | -0,11668754 | TUMOR | Smokers |
| GSE32863 | DEFENSE RESPONSE | MX2 | 35396 | -0,4682675 | -0,09411923 | TUMOR | Non-Smokers |
| TCGA | DEFENSE RESPONSE | MX2 | 17178 | -0,07786776 | -0,33801362 | TUMOR | Smokers |
| GSE50081 | IMMUNE SYSTEM PROCESS | MYH9 | 16771 | -0,10028581 | -0,35574803 | TUMOR | Smokers |
| TCGA | IMMUNE SYSTEM PROCESS | MYH9 | 18422 | -0,0985036 | -0,22039081 | TUMOR | Smokers |
| GSE47115 | CELLULAR DEFENSE RESPONSE | NCF1 | 15779 | -0,21223693 | -0,358068 | TUMOR | Smokers |
| GSE32863 | CELLULAR DEFENSE RESPONSE | NCF1 | 28154 | -0,19125915 | -0,39094484 | TUMOR | Non-Smokers |
| GSE32863 | CELLULAR DEFENSE RESPONSE | NCF1 | 36124 | -0,51159728 | -0,20907502 | TUMOR | Smokers |
| TCGA | CELLULAR DEFENSE RESPONSE | NCF1 | 17430 | -0,08154388 | -0,243035 | TUMOR | Smokers |
| GSE47115 | DEFENSE RESPONSE | NCF1 | 15779 | -0,21223693 | -0,33893022 | TUMOR | Smokers |
| GSE32863 | DEFENSE RESPONSE | NCF1 | 28154 | -0,19125915 | -0,36470705 | TUMOR | Non-Smokers |
| GSE32863 | DEFENSE RESPONSE | NCF1 | 36124 | -0,51159728 | -0,12050284 | TUMOR | Smokers |
| TCGA | DEFENSE RESPONSE | NCF1 | 17430 | -0,08154388 | -0,32608855 | TUMOR | Smokers |
| GSE32863 | CELLULAR DEFENSE RESPONSE | NCF2 | 30562 | -0,25280377 | -0,32844335 | TUMOR | Non-Smokers |
| GSE32863 | CELLULAR DEFENSE RESPONSE | NCF2 | 35372 | -0,42345712 | -0,35066217 | TUMOR | Smokers |
| TCGA | CELLULAR DEFENSE RESPONSE | NCF2 | 15016 | -0,05190588 | -0,39087316 | TUMOR | Smokers |
| GSE32863 | DEFENSE RESPONSE | NCF2 | 30562 | -0,25280377 | -0,30699077 | TUMOR | Non-Smokers |
| GSE32863 | DEFENSE RESPONSE | NCF2 | 35372 | -0,42345712 | -0,23720953 | TUMOR | Smokers |
| TCGA | DEFENSE RESPONSE | NCF2 | 15016 | -0,05190588 | -0,41072974 | TUMOR | Smokers |
| GSE50081 | IMMUNE_RESPONSE | NCF4 | 17344 | -0,11320082 | -0,4062383 | TUMOR | Smokers |
| GSE47115 | IMMUNE_RESPONSE | NCF4 | 18840 | -0,38043642 | -0,33017936 | TUMOR | Smokers |
| GSE32863 | IMMUNE_RESPONSE | NCF4 | 35229 | -0,41221169 | -0,25241104 | TUMOR | Smokers |
| TCGA | IMMUNE_RESPONSE | NCF4 | 17678 | -0,08535305 | -0,2997809 | TUMOR | Smokers |
| GSE50081 | IMMUNE SYSTEM PROCESS | NCF4 | 17344 | -0,11320082 | -0,35705197 | TUMOR | Smokers |
| GSE47115 | IMMUNE SYSTEM PROCESS | NCF4 | 18840 | -0,38043642 | -0,32122174 | TUMOR | Smokers |
| GSE32863 | IMMUNE SYSTEM PROCESS | NCF4 | 35229 | -0,41221169 | -0,21121916 | TUMOR | Smokers |
| TCGA | IMMUNE SYSTEM PROCESS | NCF4 | 17678 | -0,08535305 | -0,26675165 | TUMOR | Smokers |
| GSE50081 | IMMUNE SYSTEM PROCESS | NCK1 | 18821 | -0,15648563 | -0,25773057 | TUMOR | Smokers |
| GSE32863 | IMMUNE SYSTEM PROCESS | NCK1 | 31557 | -0,28212994 | -0,32515082 | TUMOR | Non-Smokers |
| GSE32863 | IMMUNE SYSTEM PROCESS | NCK2 | 35131 | -0,44646603 | -0,21003413 | TUMOR | Non-Smokers |
| GSE32863 | IMMUNE SYSTEM PROCESS | NCOA6 | 33591 | -0,358354 | -0,2724617 | TUMOR | Non-Smokers |
| TCGA | IMMUNE SYSTEM PROCESS | NCOA6 | 16118 | -0,21533337 | -0,3370889 | TUMOR | Non-Smokers |
| GSE50081 | IMMUNE_RESPONSE | NCR1 | 18093 | -0,13293827 | -0,34075373 | TUMOR | Smokers |
| GSE50081 | IMMUNE SYSTEM PROCESS | NCR1 | 18093 | -0,13293827 | -0,29506037 | TUMOR | Smokers |
| TCGA | IMMUNE SYSTEM PROCESS | NCR1 | 14304 | -0,0445682 | -0,3723164 | TUMOR | Smokers |
| GSE50081 | CELLULAR DEFENSE RESPONSE | NCR1 | 18093 | -0,13293827 | -0,33363342 | TUMOR | Smokers |
| GSE32863 | CELLULAR DEFENSE RESPONSE | NCR1 | 26853 | -0,16192062 | -0,4713021 | TUMOR | Non-Smokers |
| TCGA | CELLULAR DEFENSE RESPONSE | NCR1 | 14304 | -0,0445682 | -0,40188384 | TUMOR | Smokers |
| GSE50081 | DEFENSE RESPONSE | NCR1 | 18093 | -0,13293827 | -0,38464656 | TUMOR | Smokers |
| GSE50081 | REGULATION OF IMMUNE SYSTEM PROCESS | NCR1 | 18093 | -0,13293827 | -0,42253816 | TUMOR | Smokers |
| TCGA | REGULATION OF IMMUNE SYSTEM PROCESS | NCR1 | 14556 | -0,16769692 | -0,42010832 | TUMOR | Non-Smokers |
| GSE32863 | CELLULAR DEFENSE RESPONSE | NCR2 | 32267 | -0,30574846 | -0,3065492 | TUMOR | Non-Smokers |
| GSE32863 | DEFENSE RESPONSE | NCR2 | 32267 | -0,30574846 | -0,27657208 | TUMOR | Non-Smokers |
| GSE50081 | IMMUNE_RESPONSE | NFAM1 | 17364 | -0,11407686 | -0,39761844 | TUMOR | Smokers |
| GSE32863 | IMMUNE_RESPONSE | NFAM1 | 34776 | -0,37759444 | -0,30006933 | TUMOR | Smokers |
| TCGA | IMMUNE_RESPONSE | NFAM1 | 18400 | -0,09816105 | -0,26385072 | TUMOR | Smokers |
| TCGA | IMMUNE_RESPONSE | NFAM1 | 17003 | -0,24915384 | -0,26596108 | TUMOR | Non-Smokers |
| GSE50081 | IMMUNE SYSTEM PROCESS | NFAM1 | 17364 | -0,11407686 | -0,35094026 | TUMOR | Smokers |
| GSE32863 | IMMUNE SYSTEM PROCESS | NFAM1 | 34776 | -0,37759444 | -0,25968567 | TUMOR | Smokers |
| TCGA | IMMUNE SYSTEM PROCESS | NFAM1 | 18400 | -0,09816105 | -0,22397423 | TUMOR | Smokers |
| TCGA | IMMUNE SYSTEM PROCESS | NFAM1 | 17003 | -0,24915384 | -0,27290973 | TUMOR | Non-Smokers |
| GSE50081 | REGULATION OF IMMUNE SYSTEM PROCESS | NFAM1 | 17364 | -0,11407686 | -0,45793578 | TUMOR | Smokers |
| GSE32863 | REGULATION OF IMMUNE SYSTEM PROCESS | NFAM1 | 34776 | -0,37759444 | -0,270059 | TUMOR | Smokers |
| TCGA | REGULATION OF IMMUNE SYSTEM PROCESS | NFAM1 | 17003 | -0,24915384 | -0,37236673 | TUMOR | Non-Smokers |
| GSE47115 | DEFENSE RESPONSE | NFATC3 | 17937 | -0,31941372 | -0,25137 | TUMOR | Smokers |
| GSE32863 | DEFENSE RESPONSE | NFATC3 | 35050 | -0,39774802 | -0,2629087 | TUMOR | Smokers |
| TCGA | DEFENSE RESPONSE | NFATC4 | 18196 | -0,09412907 | -0,2516454 | TUMOR | Smokers |
| GSE32863 | DEFENSE RESPONSE | NFRKB | 30193 | -0,21711656 | -0,40340447 | TUMOR | Smokers |
| TCGA | IMMUNE SYSTEM PROCESS | NLRC3 | 18225 | -0,09466632 | -0,23371029 | TUMOR | Smokers |
| GSE32863 | DEFENSE RESPONSE | NLRC4 | 36344 | -0,61070102 | 0,009824926 | TUMOR | Non-Smokers |
| TCGA | DEFENSE RESPONSE | NLRC4 | 15883 | -0,06130127 | -0,41200098 | TUMOR | Smokers |
| GSE32863 | DEFENSE RESPONSE | NLRP3 | 35753 | -0,45972627 | -0,16681713 | TUMOR | Smokers |
| TCGA | DEFENSE RESPONSE | NLRP3 | 16845 | -0,07326663 | -0,35410267 | TUMOR | Smokers |
| GSE50081 | DEFENSE RESPONSE | NMI | 17467 | -0,11641851 | -0,4213736 | TUMOR | Smokers |
| GSE32863 | DEFENSE RESPONSE | NMI | 34755 | -0,41773501 | -0,1534255 | TUMOR | Non-Smokers |
| TCGA | DEFENSE RESPONSE | NOD1 | 18697 | -0,10415392 | -0,21596976 | TUMOR | Smokers |
| GSE32863 | DEFENSE RESPONSE | NOD2 | 29974 | -0,23578204 | -0,3372424 | TUMOR | Non-Smokers |
| GSE32863 | DEFENSE RESPONSE | NOD2 | 31328 | -0,24544792 | -0,3808571 | TUMOR | Smokers |
| TCGA | IMMUNE SYSTEM PROCESS | NOTCH2 | 18018 | -0,09099463 | -0,24565035 | TUMOR | Smokers |
| TCGA | DEFENSE RESPONSE | NOX4 | 18121 | -0,0927108 | -0,25934166 | TUMOR | Smokers |
| GSE47115 | IMMUNE_RESPONSE | ODZ1 | 20030 | -0,5181762 | -0,19298707 | TUMOR | Smokers |
| GSE32863 | IMMUNE_RESPONSE | ODZ1 | 34253 | -0,38968816 | -0,25253108 | TUMOR | Non-Smokers |
| TCGA | IMMUNE_RESPONSE | ODZ1 | 18387 | -0,3210071 | -0,13888706 | TUMOR | Non-Smokers |
| GSE47115 | IMMUNE SYSTEM PROCESS | ODZ1 | 20030 | -0,5181762 | -0,18837024 | TUMOR | Smokers |
| GSE32863 | IMMUNE SYSTEM PROCESS | ODZ1 | 34253 | -0,38968816 | -0,24145235 | TUMOR | Non-Smokers |
| TCGA | IMMUNE SYSTEM PROCESS | ODZ1 | 18387 | -0,3210071 | -0,15513295 | TUMOR | Non-Smokers |
| GSE32863 | IMMUNE_RESPONSE | OPRD1 | 29217 | -0,19468132 | -0,4295139 | TUMOR | Smokers |
| GSE32863 | IMMUNE SYSTEM PROCESS | OPRD1 | 29217 | -0,19468132 | -0,40502495 | TUMOR | Smokers |
| TCGA | IMMUNE_RESPONSE | OPRK1 | 20094 | -0,15980376 | -0,036072396 | TUMOR | Smokers |
| TCGA | IMMUNE SYSTEM PROCESS | OPRK1 | 20094 | -0,15980376 | -0,030056532 | TUMOR | Smokers |
| GSE47115 | DEFENSE RESPONSE | OR2H2 | 19293 | -0,42260656 | -0,20211983 | TUMOR | Smokers |
| GSE32863 | DEFENSE RESPONSE | OR2H2 | 29331 | -0,1970026 | -0,40718666 | TUMOR | Smokers |
| TCGA | DEFENSE RESPONSE | OR2H2 | 14507 | -0,04646441 | -0,41892335 | TUMOR | Smokers |
| GSE50081 | DEFENSE RESPONSE | P2RY11 | 19630 | -0,19518229 | -0,26748392 | TUMOR | Smokers |
| GSE47115 | DEFENSE RESPONSE | P2RY11 | 20273 | -0,57814324 | -0,0762153 | TUMOR | Smokers |
| TCGA | DEFENSE RESPONSE | PARP4 | 15913 | -0,06170439 | -0,40971616 | TUMOR | Smokers |
| GSE47115 | IMMUNE_RESPONSE | PAX5 | 19512 | -0,44410837 | -0,2806722 | TUMOR | Smokers |
| GSE32863 | IMMUNE_RESPONSE | PAX5 | 35913 | -0,52413672 | -0,12425528 | TUMOR | Non-Smokers |
| TCGA | IMMUNE_RESPONSE | PAX5 | 19869 | -0,14178866 | -0,045134664 | TUMOR | Smokers |
| GSE47115 | IMMUNE SYSTEM PROCESS | PAX5 | 19512 | -0,44410837 | -0,2744978 | TUMOR | Smokers |
| GSE32863 | IMMUNE SYSTEM PROCESS | PAX5 | 35913 | -0,52413672 | -0,1293814 | TUMOR | Non-Smokers |
| TCGA | IMMUNE SYSTEM PROCESS | PAX5 | 19869 | -0,14178866 | -0,047246564 | TUMOR | Smokers |
| GSE47115 | IMMUNE_RESPONSE | PDCD1 | 20607 | -0,76554728 | -0,054946497 | TUMOR | Smokers |
| GSE32863 | IMMUNE_RESPONSE | PDCD1 | 36134 | -0,56076515 | -0,06906651 | TUMOR | Non-Smokers |
| GSE32863 | IMMUNE_RESPONSE | PDCD1 | 32638 | -0,2834748 | -0,3884125 | TUMOR | Smokers |
| TCGA | IMMUNE_RESPONSE | PDCD1 | 19133 | -0,11494883 | -0,1546529 | TUMOR | Smokers |
| GSE47115 | IMMUNE SYSTEM PROCESS | PDCD1 | 20607 | -0,76554728 | -0,04810734 | TUMOR | Smokers |
| GSE32863 | IMMUNE SYSTEM PROCESS | PDCD1 | 36134 | -0,56076515 | -0,08527631 | TUMOR | Non-Smokers |
| GSE32863 | IMMUNE SYSTEM PROCESS | PDCD1 | 32638 | -0,2834748 | -0,3561643 | TUMOR | Smokers |
| TCGA | IMMUNE SYSTEM PROCESS | PDCD1 | 19133 | -0,11494883 | -0,13939959 | TUMOR | Smokers |
| GSE50081 | IMMUNE SYSTEM PROCESS | PF4 | 17471 | -0,11652859 | -0,34188005 | TUMOR | Smokers |
| GSE32863 | IMMUNE SYSTEM PROCESS | PF4 | 33734 | -0,32670024 | -0,32525015 | TUMOR | Smokers |
| GSE47115 | DEFENSE RESPONSE | PGLYRP2 | 20196 | -0,5594312 | -0,102413505 | TUMOR | Smokers |
| GSE47115 | DEFENSE RESPONSE | PGLYRP3 | 16893 | -0,26261321 | -0,3164182 | TUMOR | Smokers |
| TCGA | DEFENSE RESPONSE | PGLYRP3 | 17154 | -0,0775115 | -0,34157324 | TUMOR | Smokers |
| GSE47115 | DEFENSE RESPONSE | PGLYRP4 | 16339 | -0,23635776 | -0,32983133 | TUMOR | Smokers |
| TCGA | DEFENSE RESPONSE | PGLYRP4 | 20119 | -0,16270214 | -0,053946044 | TUMOR | Smokers |
| GSE50081 | DEFENSE RESPONSE | PLA2G2D | 18661 | -0,1515526 | -0,3388071 | TUMOR | Smokers |
| GSE32863 | DEFENSE RESPONSE | PLA2G2D | 34439 | -0,39937794 | -0,20084915 | TUMOR | Non-Smokers |
| GSE32863 | DEFENSE RESPONSE | PLA2G2D | 29225 | -0,19483243 | -0,41409296 | TUMOR | Smokers |
| TCGA | DEFENSE RESPONSE | PLA2G2D | 16335 | -0,06660112 | -0,3759593 | TUMOR | Smokers |
| GSE32863 | DEFENSE RESPONSE | PLA2G2E | 28704 | -0,20410956 | -0,3593456 | TUMOR | Non-Smokers |
| GSE50081 | DEFENSE RESPONSE | PLA2G7 | 17000 | -0,1058266 | -0,42447773 | TUMOR | Smokers |
| GSE47115 | DEFENSE RESPONSE | PLA2G7 | 17005 | -0,26795989 | -0,31243587 | TUMOR | Smokers |
| GSE32863 | DEFENSE RESPONSE | PLA2G7 | 33844 | -0,36947352 | -0,25031015 | TUMOR | Non-Smokers |
| GSE32863 | DEFENSE RESPONSE | PLA2G7 | 35741 | -0,45863366 | -0,174095 | TUMOR | Smokers |
| GSE47115 | IMMUNE_RESPONSE | POU2AF1 | 17851 | -0,31371886 | -0,36121607 | TUMOR | Smokers |
| GSE32863 | IMMUNE_RESPONSE | POU2AF1 | 28417 | -0,19730061 | -0,35966742 | TUMOR | Non-Smokers |
| GSE32863 | IMMUNE_RESPONSE | POU2AF1 | 31297 | -0,24471992 | -0,40172184 | TUMOR | Smokers |
| TCGA | IMMUNE_RESPONSE | POU2AF1 | 17828 | -0,08782069 | -0,29011464 | TUMOR | Smokers |
| GSE47115 | IMMUNE SYSTEM PROCESS | POU2AF1 | 17851 | -0,31371886 | -0,34744713 | TUMOR | Smokers |
| GSE32863 | IMMUNE SYSTEM PROCESS | POU2AF1 | 28417 | -0,19730061 | -0,38070557 | TUMOR | Non-Smokers |
| GSE32863 | IMMUNE SYSTEM PROCESS | POU2AF1 | 31297 | -0,24471992 | -0,37506756 | TUMOR | Smokers |
| TCGA | IMMUNE SYSTEM PROCESS | POU2AF1 | 17828 | -0,08782069 | -0,26176736 | TUMOR | Smokers |
| GSE50081 | IMMUNE_RESPONSE | POU2F2 | 19804 | -0,20720379 | -0,16852638 | TUMOR | Smokers |
| GSE47115 | IMMUNE_RESPONSE | POU2F2 | 19984 | -0,51029766 | -0,21814859 | TUMOR | Smokers |
| GSE32863 | IMMUNE_RESPONSE | POU2F2 | 32387 | -0,31025255 | -0,3004329 | TUMOR | Non-Smokers |
| GSE32863 | IMMUNE_RESPONSE | POU2F2 | 30341 | -0,22050934 | -0,40603918 | TUMOR | Smokers |
| TCGA | IMMUNE_RESPONSE | POU2F2 | 18555 | -0,10119139 | -0,24535076 | TUMOR | Smokers |
| TCGA | IMMUNE_RESPONSE | POU2F2 | 17543 | -0,27330157 | -0,20897204 | TUMOR | Non-Smokers |
| GSE50081 | IMMUNE SYSTEM PROCESS | POU2F2 | 19804 | -0,20720379 | -0,12919119 | TUMOR | Smokers |
| GSE47115 | IMMUNE SYSTEM PROCESS | POU2F2 | 19984 | -0,51029766 | -0,20579699 | TUMOR | Smokers |
| GSE32863 | IMMUNE SYSTEM PROCESS | POU2F2 | 32387 | -0,31025255 | -0,3088043 | TUMOR | Non-Smokers |
| GSE32863 | IMMUNE SYSTEM PROCESS | POU2F2 | 30341 | -0,22050934 | -0,3808273 | TUMOR | Smokers |
| TCGA | IMMUNE SYSTEM PROCESS | POU2F2 | 18555 | -0,10119139 | -0,20795037 | TUMOR | Smokers |
| TCGA | IMMUNE SYSTEM PROCESS | POU2F2 | 17543 | -0,27330157 | -0,22803012 | TUMOR | Non-Smokers |
| GSE47115 | IMMUNE_RESPONSE | PRELID1 | 19516 | -0,44516456 | -0,27294475 | TUMOR | Smokers |
| TCGA | IMMUNE_RESPONSE | PRELID1 | 17008 | -0,24931954 | -0,25999135 | TUMOR | Non-Smokers |
| GSE47115 | IMMUNE SYSTEM PROCESS | PRELID1 | 19516 | -0,44516456 | -0,26900244 | TUMOR | Smokers |
| TCGA | IMMUNE SYSTEM PROCESS | PRELID1 | 17008 | -0,24931954 | -0,26880506 | TUMOR | Non-Smokers |
| GSE50081 | IMMUNE SYSTEM PROCESS | PREX1 | 19145 | -0,16988443 | -0,18872483 | TUMOR | Smokers |
| GSE47115 | IMMUNE SYSTEM PROCESS | PREX1 | 17164 | -0,27739802 | -0,3743006 | TUMOR | Smokers |
| GSE32863 | IMMUNE SYSTEM PROCESS | PREX1 | 35671 | -0,49419707 | -0,16968073 | TUMOR | Non-Smokers |
| GSE50081 | CELLULAR DEFENSE RESPONSE | PRF1 | 20058 | -0,22481951 | -0,060847357 | TUMOR | Smokers |
| GSE47115 | CELLULAR DEFENSE RESPONSE | PRF1 | 20210 | -0,56404728 | -0,027301366 | TUMOR | Smokers |
| GSE32863 | CELLULAR DEFENSE RESPONSE | PRF1 | 35610 | -0,44556463 | -0,2956359 | TUMOR | Smokers |
| GSE50081 | DEFENSE RESPONSE | PRF1 | 20058 | -0,22481951 | -0,20735377 | TUMOR | Smokers |
| GSE47115 | DEFENSE RESPONSE | PRF1 | 20210 | -0,56404728 | -0,08329706 | TUMOR | Smokers |
| GSE32863 | DEFENSE RESPONSE | PRF1 | 35610 | -0,44556463 | -0,20064838 | TUMOR | Smokers |
| GSE50081 | IMMUNE SYSTEM PROCESS | PRG3 | 15916 | -0,08148856 | -0,36997512 | TUMOR | Smokers |
| GSE50081 | IMMUNE_RESPONSE | PRKRA | 18095 | -0,1332498 | -0,33525154 | TUMOR | Smokers |
| GSE50081 | IMMUNE SYSTEM PROCESS | PRKRA | 18095 | -0,1332498 | -0,29102132 | TUMOR | Smokers |
| GSE32863 | IMMUNE SYSTEM PROCESS | PRL | 28039 | -0,1884881 | -0,38572678 | TUMOR | Non-Smokers |
| TCGA | DEFENSE RESPONSE | PSG3 | 15171 | -0,05336764 | -0,41190407 | TUMOR | Smokers |
| TCGA | DEFENSE RESPONSE | PSG8 | 18835 | -0,10721828 | -0,19685717 | TUMOR | Smokers |
| GSE47115 | IMMUNE_RESPONSE | PSMB10 | 19884 | -0,49146071 | -0,23111737 | TUMOR | Smokers |
| GSE32863 | IMMUNE_RESPONSE | PSMB10 | 36588 | -0,68446851 | -0,0504532 | TUMOR | Smokers |
| TCGA | IMMUNE_RESPONSE | PSMB10 | 16369 | -0,22486088 | -0,2991913 | TUMOR | Non-Smokers |
| GSE47115 | IMMUNE SYSTEM PROCESS | PSMB10 | 19884 | -0,49146071 | -0,2200198 | TUMOR | Smokers |
| GSE32863 | IMMUNE SYSTEM PROCESS | PSMB10 | 36588 | -0,68446851 | -0,03663341 | TUMOR | Smokers |
| TCGA | IMMUNE SYSTEM PROCESS | PSMB10 | 16369 | -0,22486088 | -0,31511554 | TUMOR | Non-Smokers |
| GSE50081 | IMMUNE_RESPONSE | PTAFR | 18583 | -0,14900972 | -0,32342076 | TUMOR | Smokers |
| GSE47115 | IMMUNE_RESPONSE | PTAFR | 20075 | -0,52814388 | -0,18579264 | TUMOR | Smokers |
| GSE32863 | IMMUNE_RESPONSE | PTAFR | 30269 | -0,24423748 | -0,33000118 | TUMOR | Non-Smokers |
| GSE32863 | IMMUNE_RESPONSE | PTAFR | 36634 | -0,72518706 | -0,011898068 | TUMOR | Smokers |
| TCGA | IMMUNE_RESPONSE | PTAFR | 19781 | -0,53226727 | -0,09399551 | TUMOR | Non-Smokers |
| GSE50081 | IMMUNE SYSTEM PROCESS | PTAFR | 18583 | -0,14900972 | -0,28421623 | TUMOR | Smokers |
| GSE47115 | IMMUNE SYSTEM PROCESS | PTAFR | 20075 | -0,52814388 | -0,18383291 | TUMOR | Smokers |
| GSE32863 | IMMUNE SYSTEM PROCESS | PTAFR | 30269 | -0,24423748 | -0,351853 | TUMOR | Non-Smokers |
| GSE32863 | IMMUNE SYSTEM PROCESS | PTAFR | 36634 | -0,72518706 | -0,008345117 | TUMOR | Smokers |
| TCGA | IMMUNE SYSTEM PROCESS | PTAFR | 19781 | -0,53226727 | -0,07506325 | TUMOR | Non-Smokers |
| GSE50081 | DEFENSE RESPONSE | PTAFR | 18583 | -0,14900972 | -0,3641277 | TUMOR | Smokers |
| GSE47115 | DEFENSE RESPONSE | PTAFR | 20075 | -0,52814388 | -0,1251256 | TUMOR | Smokers |
| GSE32863 | DEFENSE RESPONSE | PTAFR | 30269 | -0,24423748 | -0,3245806 | TUMOR | Non-Smokers |
| GSE32863 | DEFENSE RESPONSE | PTAFR | 36634 | -0,72518706 | -0,011566706 | TUMOR | Smokers |
| GSE50081 | IMMUNE_RESPONSE | PTGER4 | 18734 | -0,1535393 | -0,3116762 | TUMOR | Smokers |
| GSE32863 | IMMUNE_RESPONSE | PTGER4 | 27873 | -0,1844912 | -0,36298415 | TUMOR | Non-Smokers |
| GSE32863 | IMMUNE_RESPONSE | PTGER4 | 35243 | -0,41367772 | -0,24502003 | TUMOR | Smokers |
| TCGA | IMMUNE_RESPONSE | PTGER4 | 17180 | -0,07788435 | -0,3443242 | TUMOR | Smokers |
| GSE50081 | IMMUNE SYSTEM PROCESS | PTGER4 | 18734 | -0,1535393 | -0,2775115 | TUMOR | Smokers |
| GSE32863 | IMMUNE SYSTEM PROCESS | PTGER4 | 27873 | -0,1844912 | -0,38613248 | TUMOR | Non-Smokers |
| GSE32863 | IMMUNE SYSTEM PROCESS | PTGER4 | 35243 | -0,41367772 | -0,20583032 | TUMOR | Smokers |
| TCGA | IMMUNE SYSTEM PROCESS | PTGER4 | 17180 | -0,07788435 | -0,30048957 | TUMOR | Smokers |
| GSE32863 | IMMUNE_RESPONSE | PTPRC | 30601 | -0,22708097 | -0,39614365 | TUMOR | Smokers |
| GSE32863 | IMMUNE SYSTEM PROCESS | PTPRC | 30601 | -0,22708097 | -0,36597446 | TUMOR | Smokers |
| TCGA | IMMUNE SYSTEM PROCESS | PTPRC | 15144 | -0,05317973 | -0,37437716 | TUMOR | Smokers |
| GSE47115 | DEFENSE RESPONSE | PTPRC | 16612 | -0,24910653 | -0,33007973 | TUMOR | Smokers |
| GSE32863 | DEFENSE RESPONSE | PTPRC | 30601 | -0,22708097 | -0,39223343 | TUMOR | Smokers |
| TCGA | DEFENSE RESPONSE | PTPRC | 15144 | -0,05317973 | -0,4138345 | TUMOR | Smokers |
| GSE47115 | REGULATION OF IMMUNE SYSTEM PROCESS | PTPRC | 16612 | -0,24910653 | -0,37654263 | TUMOR | Smokers |
| GSE32863 | REGULATION OF IMMUNE SYSTEM PROCESS | PTPRC | 30601 | -0,22708097 | -0,33307695 | TUMOR | Smokers |
| GSE50081 | DEFENSE RESPONSE | PTPRCAP | 18529 | -0,1473612 | -0,36725774 | TUMOR | Smokers |
| GSE47115 | DEFENSE RESPONSE | PTPRCAP | 20462 | -0,64038402 | -0,05239041 | TUMOR | Smokers |
| GSE32863 | DEFENSE RESPONSE | PTPRCAP | 32624 | -0,31954342 | -0,2754674 | TUMOR | Non-Smokers |
| GSE32863 | DEFENSE RESPONSE | PTPRCAP | 36544 | -0,64441389 | -0,05402885 | TUMOR | Smokers |
| TCGA | DEFENSE RESPONSE | PTPRCAP | 17655 | -0,08497389 | -0,301299 | TUMOR | Smokers |
| GSE50081 | DEFENSE RESPONSE | PTX3 | 17988 | -0,12948619 | -0,38980517 | TUMOR | Smokers |
| GSE32863 | DEFENSE RESPONSE | PTX3 | 34562 | -0,36592594 | -0,2815437 | TUMOR | Smokers |
| TCGA | DEFENSE RESPONSE | PTX3 | 15009 | -0,05185086 | -0,4135667 | TUMOR | Smokers |
| TCGA | IMMUNE_RESPONSE | PYDC1 | 16098 | -0,06379325 | -0,38453308 | TUMOR | Smokers |
| TCGA | IMMUNE SYSTEM PROCESS | PYDC1 | 16098 | -0,06379325 | -0,3458506 | TUMOR | Smokers |
| TCGA | DEFENSE RESPONSE | PYDC1 | 16098 | -0,06379325 | -0,3919414 | TUMOR | Smokers |
| GSE32863 | IMMUNE SYSTEM PROCESS | RAB3D | 32979 | -0,29540247 | -0,35332623 | TUMOR | Smokers |
| GSE50081 | IMMUNE_RESPONSE | RAG1 | 17061 | -0,10708705 | -0,4066032 | TUMOR | Smokers |
| GSE50081 | IMMUNE SYSTEM PROCESS | RAG1 | 17061 | -0,10708705 | -0,3536414 | TUMOR | Smokers |
| GSE32863 | IMMUNE SYSTEM PROCESS | RASGRP4 | 32753 | -0,32433566 | -0,28572237 | TUMOR | Non-Smokers |
| TCGA | IMMUNE SYSTEM PROCESS | RASGRP4 | 16743 | -0,23910065 | -0,2813056 | TUMOR | Non-Smokers |
| TCGA | DEFENSE RESPONSE | RELA | 15781 | -0,06007994 | -0,41438928 | TUMOR | Smokers |
| GSE32863 | IMMUNE_RESPONSE | RFX1 | 34779 | -0,37774009 | -0,29304975 | TUMOR | Smokers |
| TCGA | IMMUNE_RESPONSE | RFX1 | 16073 | -0,21391091 | -0,32840395 | TUMOR | Non-Smokers |
| GSE32863 | IMMUNE SYSTEM PROCESS | RFX1 | 34779 | -0,37774009 | -0,25449365 | TUMOR | Smokers |
| TCGA | IMMUNE SYSTEM PROCESS | RFX1 | 15096 | -0,05271078 | -0,37706292 | TUMOR | Smokers |
| TCGA | IMMUNE SYSTEM PROCESS | RFX1 | 16073 | -0,21391091 | -0,33856025 | TUMOR | Non-Smokers |
| GSE32863 | IMMUNE_RESPONSE | RGS1 | 27524 | -0,17650263 | -0,37734243 | TUMOR | Non-Smokers |
| GSE32863 | IMMUNE_RESPONSE | RGS1 | 33557 | -0,31888381 | -0,38441706 | TUMOR | Smokers |
| GSE32863 | IMMUNE SYSTEM PROCESS | RGS1 | 33557 | -0,31888381 | -0,33860558 | TUMOR | Smokers |
| TCGA | IMMUNE SYSTEM PROCESS | RGS1 | 15287 | -0,05457744 | -0,36850804 | TUMOR | Smokers |
| GSE50081 | DEFENSE RESPONSE | RIPK2 | 17304 | -0,11246037 | -0,4222854 | TUMOR | Smokers |
| GSE47115 | DEFENSE RESPONSE | RIPK2 | 16259 | -0,23224194 | -0,3383788 | TUMOR | Smokers |
| GSE32863 | DEFENSE RESPONSE | RIPK2 | 34571 | -0,40618911 | -0,1767367 | TUMOR | Non-Smokers |
| GSE47115 | DEFENSE RESPONSE | RNASE6 | 16319 | -0,23522882 | -0,33303672 | TUMOR | Smokers |
| GSE32863 | DEFENSE RESPONSE | RNASE6 | 28880 | -0,2083361 | -0,35699373 | TUMOR | Non-Smokers |
| GSE32863 | DEFENSE RESPONSE | RNASE6 | 33079 | -0,29961988 | -0,32984322 | TUMOR | Smokers |
| TCGA | DEFENSE RESPONSE | RNASE6 | 16471 | -0,06834009 | -0,366148 | TUMOR | Smokers |
| GSE50081 | IMMUNE SYSTEM PROCESS | RPS19 | 16466 | -0,09310347 | -0,35892144 | TUMOR | Smokers |
| GSE32863 | IMMUNE SYSTEM PROCESS | RPS19 | 28274 | -0,19393207 | -0,38452917 | TUMOR | Non-Smokers |
| GSE50081 | IMMUNE_RESPONSE | RSAD2 | 18770 | -0,15458053 | -0,30046675 | TUMOR | Smokers |
| GSE32863 | IMMUNE_RESPONSE | RSAD2 | 30613 | -0,25432503 | -0,31580868 | TUMOR | Non-Smokers |
| TCGA | IMMUNE_RESPONSE | RSAD2 | 15826 | -0,06058182 | -0,40772888 | TUMOR | Smokers |
| GSE50081 | IMMUNE SYSTEM PROCESS | RSAD2 | 18770 | -0,15458053 | -0,26970288 | TUMOR | Smokers |
| GSE32863 | IMMUNE SYSTEM PROCESS | RSAD2 | 30613 | -0,25432503 | -0,33825132 | TUMOR | Non-Smokers |
| TCGA | IMMUNE SYSTEM PROCESS | RSAD2 | 15826 | -0,06058182 | -0,35905316 | TUMOR | Smokers |
| GSE50081 | DEFENSE RESPONSE | RSAD2 | 18770 | -0,15458053 | -0,3262817 | TUMOR | Smokers |
| GSE32863 | DEFENSE RESPONSE | RSAD2 | 30613 | -0,25432503 | -0,304007 | TUMOR | Non-Smokers |
| TCGA | DEFENSE RESPONSE | RSAD2 | 15826 | -0,06058182 | -0,41291842 | TUMOR | Smokers |
| GSE32863 | IMMUNE SYSTEM PROCESS | RUNX1 | 28621 | -0,1815078 | -0,40978187 | TUMOR | Smokers |
| TCGA | IMMUNE SYSTEM PROCESS | RUNX1 | 19902 | -0,14440382 | -0,04205395 | TUMOR | Smokers |
| GSE50081 | DEFENSE RESPONSE | S100A12 | 20294 | -0,25657895 | -0,14406227 | TUMOR | Smokers |
| GSE32863 | DEFENSE RESPONSE | S100A12 | 34485 | -0,40203795 | -0,1882916 | TUMOR | Non-Smokers |
| GSE32863 | DEFENSE RESPONSE | S100A12 | 34256 | -0,3496964 | -0,2909857 | TUMOR | Smokers |
| TCGA | DEFENSE RESPONSE | S100A12 | 14797 | -0,04961123 | -0,41555515 | TUMOR | Smokers |
| GSE50081 | DEFENSE RESPONSE | S100A7 | 20598 | -0,46427748 | 3,44E-04 | TUMOR | Smokers |
| GSE32863 | DEFENSE RESPONSE | S100A7 | 28065 | -0,18907599 | -0,36556786 | TUMOR | Non-Smokers |
| TCGA | DEFENSE RESPONSE | S100A7 | 16202 | -0,06511126 | -0,37341657 | TUMOR | Smokers |
| GSE50081 | DEFENSE RESPONSE | S100A8 | 20457 | -0,29208633 | -0,08842802 | TUMOR | Smokers |
| GSE47115 | DEFENSE RESPONSE | S100A8 | 15332 | -0,19326809 | -0,3496441 | TUMOR | Smokers |
| GSE32863 | DEFENSE RESPONSE | S100A8 | 33886 | -0,3719393 | -0,24506494 | TUMOR | Non-Smokers |
| GSE32863 | DEFENSE RESPONSE | S100A8 | 35540 | -0,43781272 | -0,20610106 | TUMOR | Smokers |
| GSE50081 | DEFENSE RESPONSE | S100A9 | 18371 | -0,14194465 | -0,3820949 | TUMOR | Smokers |
| GSE47115 | DEFENSE RESPONSE | S100A9 | 17037 | -0,26965272 | -0,30923462 | TUMOR | Smokers |
| GSE32863 | DEFENSE RESPONSE | S100A9 | 29553 | -0,22539 | -0,34566197 | TUMOR | Non-Smokers |
| TCGA | DEFENSE RESPONSE | S100A9 | 16853 | -0,0733739 | -0,35001865 | TUMOR | Smokers |
| GSE47115 | IMMUNE_RESPONSE | S1PR4 | 19375 | -0,43121329 | -0,2975001 | TUMOR | Smokers |
| GSE32863 | IMMUNE_RESPONSE | S1PR4 | 28349 | -0,17602487 | -0,44402882 | TUMOR | Smokers |
| GSE47115 | IMMUNE SYSTEM PROCESS | S1PR4 | 19375 | -0,43121329 | -0,28467396 | TUMOR | Smokers |
| GSE32863 | IMMUNE SYSTEM PROCESS | S1PR4 | 28349 | -0,17602487 | -0,41486236 | TUMOR | Smokers |
| GSE50081 | IMMUNE SYSTEM PROCESS | SAA1 | 19189 | -0,17229055 | -0,18024263 | TUMOR | Smokers |
| GSE47115 | IMMUNE SYSTEM PROCESS | SAA1 | 19052 | -0,4001576 | -0,30638102 | TUMOR | Smokers |
| GSE32863 | IMMUNE SYSTEM PROCESS | SAA1 | 36523 | -0,68780565 | -0,03941799 | TUMOR | Non-Smokers |
| GSE32863 | IMMUNE SYSTEM PROCESS | SAA1 | 34767 | -0,37724468 | -0,26471075 | TUMOR | Smokers |
| TCGA | IMMUNE SYSTEM PROCESS | SAA1 | 15333 | -0,0550438 | -0,36816522 | TUMOR | Smokers |
| GSE47115 | IMMUNE SYSTEM PROCESS | SART1 | 20409 | -0,62143546 | -0,11651419 | TUMOR | Smokers |
| GSE32863 | IMMUNE SYSTEM PROCESS | SART1 | 29118 | -0,21404095 | -0,3754749 | TUMOR | Non-Smokers |
| GSE47115 | REGULATION OF IMMUNE SYSTEM PROCESS | SART1 | 20409 | -0,62143546 | -0,024208719 | TUMOR | Smokers |
| TCGA | IMMUNE SYSTEM PROCESS | SCG2 | 19278 | -0,40326834 | -0,11037573 | TUMOR | Non-Smokers |
| GSE32863 | IMMUNE SYSTEM PROCESS | SCIN | 29730 | -0,22993605 | -0,37776083 | TUMOR | Non-Smokers |
| GSE32863 | IMMUNE SYSTEM PROCESS | SCIN | 31927 | -0,26232585 | -0,3674603 | TUMOR | Smokers |
| TCGA | IMMUNE SYSTEM PROCESS | SCIN | 19237 | -0,39735037 | -0,12230349 | TUMOR | Non-Smokers |
| GSE50081 | IMMUNE_RESPONSE | SECTM1 | 17607 | -0,12012665 | -0,37507662 | TUMOR | Smokers |
| TCGA | IMMUNE_RESPONSE | SECTM1 | 18602 | -0,1021331 | -0,2277659 | TUMOR | Smokers |
| GSE50081 | IMMUNE SYSTEM PROCESS | SECTM1 | 17607 | -0,12012665 | -0,3338155 | TUMOR | Smokers |
| TCGA | IMMUNE SYSTEM PROCESS | SECTM1 | 18602 | -0,1021331 | -0,19579136 | TUMOR | Smokers |
| TCGA | DEFENSE RESPONSE | SELE | 18072 | -0,091842 | -0,26255435 | TUMOR | Smokers |
| TCGA | IMMUNE_RESPONSE | SEMA3C | 19141 | -0,11519939 | -0,14754106 | TUMOR | Smokers |
| TCGA | IMMUNE_RESPONSE | SEMA3C | 18361 | -0,31918272 | -0,14556286 | TUMOR | Non-Smokers |
| TCGA | IMMUNE SYSTEM PROCESS | SEMA3C | 19141 | -0,11519939 | -0,13433275 | TUMOR | Smokers |
| TCGA | IMMUNE SYSTEM PROCESS | SEMA3C | 18361 | -0,31918272 | -0,15940256 | TUMOR | Non-Smokers |
| GSE50081 | IMMUNE_RESPONSE | SEMA4D | 20363 | -0,2683461 | -0,066160776 | TUMOR | Smokers |
| GSE47115 | IMMUNE_RESPONSE | SEMA4D | 19863 | -0,48914939 | -0,2388341 | TUMOR | Smokers |
| GSE32863 | IMMUNE_RESPONSE | SEMA4D | 36272 | -0,59237194 | -0,02914247 | TUMOR | Non-Smokers |
| GSE32863 | IMMUNE_RESPONSE | SEMA4D | 29252 | -0,19534416 | -0,4267877 | TUMOR | Smokers |
| GSE50081 | IMMUNE SYSTEM PROCESS | SEMA4D | 20363 | -0,2683461 | -0,05411415 | TUMOR | Smokers |
| GSE47115 | IMMUNE SYSTEM PROCESS | SEMA4D | 19863 | -0,48914939 | -0,22526865 | TUMOR | Smokers |
| GSE32863 | IMMUNE SYSTEM PROCESS | SEMA4D | 36272 | -0,59237194 | -0,058490638 | TUMOR | Non-Smokers |
| GSE32863 | IMMUNE SYSTEM PROCESS | SEMA4D | 29252 | -0,19534416 | -0,4032461 | TUMOR | Smokers |
| TCGA | IMMUNE SYSTEM PROCESS | SEMA4D | 14750 | -0,04900992 | -0,36963618 | TUMOR | Smokers |
| TCGA | IMMUNE_RESPONSE | SEMA7A | 16996 | -0,24898277 | -0,2718249 | TUMOR | Non-Smokers |
| TCGA | IMMUNE SYSTEM PROCESS | SEMA7A | 15229 | -0,05397179 | -0,37345952 | TUMOR | Smokers |
| TCGA | IMMUNE SYSTEM PROCESS | SEMA7A | 16996 | -0,24898277 | -0,27690923 | TUMOR | Non-Smokers |
| GSE50081 | IMMUNE_RESPONSE | SFTPD | 16488 | -0,09367735 | -0,4086076 | TUMOR | Smokers |
| GSE50081 | IMMUNE SYSTEM PROCESS | SFTPD | 16488 | -0,09367735 | -0,3570819 | TUMOR | Smokers |
| GSE47115 | DEFENSE RESPONSE | SIGIRR | 15759 | -0,21140592 | -0,34171376 | TUMOR | Smokers |
| GSE32863 | DEFENSE RESPONSE | SIGIRR | 35242 | -0,41367283 | -0,25454512 | TUMOR | Smokers |
| GSE50081 | IMMUNE SYSTEM PROCESS | SIRPG | 17952 | -0,1285304 | -0,3003665 | TUMOR | Smokers |
| GSE47115 | IMMUNE SYSTEM PROCESS | SIRPG | 20358 | -0,606574 | -0,1453661 | TUMOR | Smokers |
| GSE32863 | IMMUNE SYSTEM PROCESS | SIRPG | 36122 | -0,51149815 | -0,11555473 | TUMOR | Smokers |
| TCGA | IMMUNE SYSTEM PROCESS | SIRPG | 14294 | -0,04450871 | -0,37396276 | TUMOR | Smokers |
| TCGA | IMMUNE SYSTEM PROCESS | SIRPG | 17815 | -0,28675091 | -0,2271589 | TUMOR | Non-Smokers |
| GSE47115 | REGULATION OF IMMUNE SYSTEM PROCESS | SIRPG | 20358 | -0,606574 | -0,058580983 | TUMOR | Smokers |
| GSE10072 | REGULATION OF IMMUNE SYSTEM PROCESS | SIRPG | 11853 | -0,40351319 | -0,29064202 | NORMAL | Non-Smokers |
| GSE32863 | REGULATION OF IMMUNE SYSTEM PROCESS | SIRPG | 36122 | -0,51149815 | -0,104316205 | TUMOR | Smokers |
| TCGA | REGULATION OF IMMUNE SYSTEM PROCESS | SIRPG | 17815 | -0,28675091 | -0,30152896 | TUMOR | Non-Smokers |
| GSE50081 | IMMUNE SYSTEM PROCESS | SIT1 | 17951 | -0,12852226 | -0,30431 | TUMOR | Smokers |
| GSE47115 | IMMUNE SYSTEM PROCESS | SIT1 | 19902 | -0,49367383 | -0,20829439 | TUMOR | Smokers |
| GSE32863 | IMMUNE SYSTEM PROCESS | SIT1 | 35040 | -0,39701003 | -0,22891633 | TUMOR | Smokers |
| GSE47115 | REGULATION OF IMMUNE SYSTEM PROCESS | SIT1 | 19902 | -0,49367383 | -0,1790154 | TUMOR | Smokers |
| GSE10072 | REGULATION OF IMMUNE SYSTEM PROCESS | SIT1 | 12112 | -0,43459955 | -0,24675937 | NORMAL | Non-Smokers |
| GSE32863 | REGULATION OF IMMUNE SYSTEM PROCESS | SIT1 | 35040 | -0,39701003 | -0,25210026 | TUMOR | Smokers |
| GSE47115 | IMMUNE_RESPONSE | SKAP1 | 20657 | -0,8278138 | -0,042695656 | TUMOR | Smokers |
| GSE32863 | IMMUNE_RESPONSE | SKAP1 | 27681 | -0,18003941 | -0,37487814 | TUMOR | Non-Smokers |
| GSE47115 | IMMUNE SYSTEM PROCESS | SKAP1 | 20657 | -0,8278138 | -0,029696722 | TUMOR | Smokers |
| GSE32863 | IMMUNE SYSTEM PROCESS | SKAP1 | 27681 | -0,18003941 | -0,39288783 | TUMOR | Non-Smokers |
| GSE47115 | IMMUNE_RESPONSE | SLA2 | 17929 | -0,31866044 | -0,35369024 | TUMOR | Smokers |
| GSE32863 | IMMUNE_RESPONSE | SLA2 | 33534 | -0,31778592 | -0,38978598 | TUMOR | Smokers |
| TCGA | IMMUNE_RESPONSE | SLA2 | 15655 | -0,05876601 | -0,4189018 | TUMOR | Smokers |
| GSE47115 | IMMUNE SYSTEM PROCESS | SLA2 | 17929 | -0,31866044 | -0,34312195 | TUMOR | Smokers |
| GSE32863 | IMMUNE SYSTEM PROCESS | SLA2 | 33534 | -0,31778592 | -0,3424303 | TUMOR | Smokers |
| TCGA | IMMUNE SYSTEM PROCESS | SLA2 | 15655 | -0,05876601 | -0,36768225 | TUMOR | Smokers |
| GSE47115 | REGULATION OF IMMUNE SYSTEM PROCESS | SLA2 | 17929 | -0,31866044 | -0,35523838 | TUMOR | Smokers |
| GSE32863 | REGULATION OF IMMUNE SYSTEM PROCESS | SLA2 | 33534 | -0,31778592 | -0,3252282 | TUMOR | Smokers |
| GSE32863 | IMMUNE SYSTEM PROCESS | SNRK | 28767 | -0,20571688 | -0,3796664 | TUMOR | Non-Smokers |
| TCGA | IMMUNE SYSTEM PROCESS | SNRK | 15102 | -0,05275969 | -0,3748319 | TUMOR | Smokers |
| GSE32863 | DEFENSE RESPONSE | SOCS6 | 34817 | -0,38086581 | -0,27611148 | TUMOR | Smokers |
| TCGA | IMMUNE SYSTEM PROCESS | SOD1 | 19069 | -0,38004839 | -0,14787385 | TUMOR | Non-Smokers |
| GSE50081 | DEFENSE RESPONSE | SP140 | 20228 | -0,2482245 | -0,16045748 | TUMOR | Smokers |
| GSE47115 | DEFENSE RESPONSE | SP140 | 19107 | -0,40521365 | -0,20771018 | TUMOR | Smokers |
| GSE32863 | DEFENSE RESPONSE | SP140 | 35342 | -0,46355051 | -0,10068499 | TUMOR | Non-Smokers |
| GSE32863 | DEFENSE RESPONSE | SP140 | 34354 | -0,35483 | -0,2877974 | TUMOR | Smokers |
| TCGA | DEFENSE RESPONSE | SP140 | 17780 | -0,08700703 | -0,2916449 | TUMOR | Smokers |
| GSE32863 | IMMUNE SYSTEM PROCESS | SPACA3 | 30429 | -0,22325961 | -0,373902 | TUMOR | Smokers |
| GSE32863 | DEFENSE RESPONSE | SPACA3 | 30429 | -0,22325961 | -0,40253723 | TUMOR | Smokers |
| GSE32863 | REGULATION OF IMMUNE SYSTEM PROCESS | SPACA3 | 30429 | -0,22325961 | -0,35705203 | TUMOR | Smokers |
| GSE50081 | IMMUNE SYSTEM PROCESS | SPI1 | 19046 | -0,1650099 | -0,19939092 | TUMOR | Smokers |
| GSE47115 | IMMUNE SYSTEM PROCESS | SPI1 | 18115 | -0,32902706 | -0,343849 | TUMOR | Smokers |
| GSE32863 | IMMUNE SYSTEM PROCESS | SPI1 | 33063 | -0,33597279 | -0,2854639 | TUMOR | Non-Smokers |
| GSE32863 | IMMUNE SYSTEM PROCESS | SPI1 | 34678 | -0,37223414 | -0,2779999 | TUMOR | Smokers |
| TCGA | IMMUNE SYSTEM PROCESS | SPI1 | 14576 | -0,047362 | -0,3679336 | TUMOR | Smokers |
| TCGA | IMMUNE SYSTEM PROCESS | SPI1 | 16939 | -0,24656886 | -0,2870178 | TUMOR | Non-Smokers |
| GSE32863 | IMMUNE_RESPONSE | SPINK5 | 33495 | -0,31615984 | -0,39469564 | TUMOR | Smokers |
| GSE32863 | IMMUNE SYSTEM PROCESS | SPINK5 | 33495 | -0,31615984 | -0,34579995 | TUMOR | Smokers |
| GSE10072 | REGULATION OF IMMUNE SYSTEM PROCESS | SPINK5 | 13113 | -0,68586177 | -0,03756566 | NORMAL | Non-Smokers |
| GSE32863 | REGULATION OF IMMUNE SYSTEM PROCESS | SPINK5 | 33495 | -0,31615984 | -0,3443122 | TUMOR | Smokers |
| GSE50081 | CELLULAR DEFENSE RESPONSE | SPN | 16925 | -0,10392888 | -0,42703125 | TUMOR | Smokers |
| GSE47115 | CELLULAR DEFENSE RESPONSE | SPN | 20205 | -0,56242663 | -0,06864362 | TUMOR | Smokers |
| GSE32863 | CELLULAR DEFENSE RESPONSE | SPN | 35748 | -0,50299698 | -0,06610487 | TUMOR | Non-Smokers |
| TCGA | CELLULAR DEFENSE RESPONSE | SPN | 17636 | -0,08461098 | -0,17495918 | TUMOR | Smokers |
| GSE50081 | DEFENSE RESPONSE | SPN | 16925 | -0,10392888 | -0,4289842 | TUMOR | Smokers |
| GSE47115 | DEFENSE RESPONSE | SPN | 20205 | -0,56242663 | -0,09296721 | TUMOR | Smokers |
| GSE32863 | DEFENSE RESPONSE | SPN | 35748 | -0,50299698 | -0,07865789 | TUMOR | Non-Smokers |
| TCGA | DEFENSE RESPONSE | SPN | 17636 | -0,08461098 | -0,31587923 | TUMOR | Smokers |
| GSE50081 | IMMUNE_RESPONSE | ST6GAL1 | 16084 | -0,08563094 | -0,41516447 | TUMOR | Smokers |
| GSE47115 | IMMUNE_RESPONSE | ST6GAL1 | 18965 | -0,39276773 | -0,32234108 | TUMOR | Smokers |
| GSE32863 | IMMUNE_RESPONSE | ST6GAL1 | 30217 | -0,24297649 | -0,34232453 | TUMOR | Non-Smokers |
| GSE50081 | IMMUNE SYSTEM PROCESS | ST6GAL1 | 16084 | -0,08563094 | -0,36779574 | TUMOR | Smokers |
| GSE47115 | IMMUNE SYSTEM PROCESS | ST6GAL1 | 18965 | -0,39276773 | -0,31235772 | TUMOR | Smokers |
| GSE32863 | IMMUNE SYSTEM PROCESS | ST6GAL1 | 30217 | -0,24297649 | -0,36005735 | TUMOR | Non-Smokers |
| GSE32863 | DEFENSE RESPONSE | STAB1 | 34366 | -0,39612284 | -0,20571359 | TUMOR | Non-Smokers |
| GSE32863 | DEFENSE RESPONSE | STAB1 | 32120 | -0,26749319 | -0,35994285 | TUMOR | Smokers |
| GSE50081 | IMMUNE SYSTEM PROCESS | SYK | 20388 | -0,27510634 | -0,03855379 | TUMOR | Smokers |
| GSE47115 | IMMUNE SYSTEM PROCESS | SYK | 19219 | -0,41568363 | -0,293477 | TUMOR | Smokers |
| GSE32863 | IMMUNE SYSTEM PROCESS | SYK | 36567 | -0,715011 | -0,02209325 | TUMOR | Non-Smokers |
| GSE32863 | IMMUNE SYSTEM PROCESS | SYK | 33350 | -0,30990511 | -0,3506327 | TUMOR | Smokers |
| GSE47115 | DEFENSE RESPONSE | TACR1 | 20481 | -0,65210915 | -0,04186428 | TUMOR | Smokers |
| GSE47115 | IMMUNE_RESPONSE | TAPBP | 17417 | -0,29014441 | -0,3886801 | TUMOR | Smokers |
| TCGA | IMMUNE_RESPONSE | TAPBP | 16158 | -0,06452069 | -0,37907505 | TUMOR | Smokers |
| GSE47115 | IMMUNE SYSTEM PROCESS | TAPBP | 17417 | -0,29014441 | -0,36492348 | TUMOR | Smokers |
| TCGA | IMMUNE SYSTEM PROCESS | TAPBP | 16158 | -0,06452069 | -0,34269023 | TUMOR | Smokers |
| GSE32863 | IMMUNE_RESPONSE | TARBP2 | 28431 | -0,17733142 | -0,43958852 | TUMOR | Smokers |
| GSE32863 | IMMUNE SYSTEM PROCESS | TARBP2 | 28431 | -0,17733142 | -0,41214117 | TUMOR | Smokers |
| GSE32863 | REGULATION OF IMMUNE SYSTEM PROCESS | TARBP2 | 28431 | -0,17733142 | -0,39232227 | TUMOR | Smokers |
| GSE50081 | IMMUNE SYSTEM PROCESS | TAZ | 15895 | -0,08108972 | -0,37148997 | TUMOR | Smokers |
| GSE32863 | IMMUNE SYSTEM PROCESS | TAZ | 34842 | -0,42320618 | -0,22500205 | TUMOR | Non-Smokers |
| TCGA | IMMUNE SYSTEM PROCESS | TAZ | 17026 | -0,24986586 | -0,26535586 | TUMOR | Non-Smokers |
| GSE32863 | IMMUNE_RESPONSE | TCF7 | 29915 | -0,23433203 | -0,347647 | TUMOR | Non-Smokers |
| GSE32863 | IMMUNE_RESPONSE | TCF7 | 28778 | -0,18482256 | -0,43192652 | TUMOR | Smokers |
| GSE32863 | IMMUNE SYSTEM PROCESS | TCF7 | 29915 | -0,23433203 | -0,36753294 | TUMOR | Non-Smokers |
| GSE32863 | IMMUNE SYSTEM PROCESS | TCF7 | 28778 | -0,18482256 | -0,4063554 | TUMOR | Smokers |
| GSE47115 | CELLULAR DEFENSE RESPONSE | TCIRG1 | 15544 | -0,20259172 | -0,37749034 | TUMOR | Smokers |
| GSE32863 | CELLULAR DEFENSE RESPONSE | TCIRG1 | 35338 | -0,46305743 | -0,09553151 | TUMOR | Non-Smokers |
| GSE32863 | CELLULAR DEFENSE RESPONSE | TCIRG1 | 36565 | -0,66007596 | -0,05326043 | TUMOR | Smokers |
| GSE47115 | DEFENSE RESPONSE | TCIRG1 | 15544 | -0,20259172 | -0,34580383 | TUMOR | Smokers |
| GSE32863 | DEFENSE RESPONSE | TCIRG1 | 35338 | -0,46305743 | -0,10854212 | TUMOR | Non-Smokers |
| GSE32863 | DEFENSE RESPONSE | TCIRG1 | 36565 | -0,66007596 | -0,032932978 | TUMOR | Smokers |
| GSE32863 | DEFENSE RESPONSE | TFF3 | 29785 | -0,20713373 | -0,402748 | TUMOR | Smokers |
| TCGA | IMMUNE_RESPONSE | TGFB1 | 19645 | -0,13101834 | -0,069938436 | TUMOR | Smokers |
| TCGA | IMMUNE SYSTEM PROCESS | TGFB1 | 19645 | -0,13101834 | -0,062211655 | TUMOR | Smokers |
| TCGA | DEFENSE RESPONSE | TGFB1 | 19645 | -0,13101834 | -0,12857662 | TUMOR | Smokers |
| GSE32863 | IMMUNE_RESPONSE | TGFB2 | 28027 | -0,18828544 | -0,36366567 | TUMOR | Non-Smokers |
| GSE32863 | IMMUNE_RESPONSE | TGFB2 | 28913 | -0,1875605 | -0,432088 | TUMOR | Smokers |
| TCGA | IMMUNE_RESPONSE | TGFB2 | 18394 | -0,09801845 | -0,26995862 | TUMOR | Smokers |
| GSE32863 | IMMUNE SYSTEM PROCESS | TGFB2 | 28027 | -0,18828544 | -0,38788256 | TUMOR | Non-Smokers |
| GSE32863 | IMMUNE SYSTEM PROCESS | TGFB2 | 28913 | -0,1875605 | -0,40743342 | TUMOR | Smokers |
| TCGA | IMMUNE SYSTEM PROCESS | TGFB2 | 18394 | -0,09801845 | -0,22833979 | TUMOR | Smokers |
| GSE32863 | REGULATION OF IMMUNE SYSTEM PROCESS | TGFB2 | 28913 | -0,1875605 | -0,39357567 | TUMOR | Smokers |
| GSE47115 | IMMUNE_RESPONSE | THY1 | 16800 | -0,25831237 | -0,41728628 | TUMOR | Smokers |
| GSE32863 | IMMUNE_RESPONSE | THY1 | 33256 | -0,34405082 | -0,28773597 | TUMOR | Non-Smokers |
| TCGA | IMMUNE_RESPONSE | THY1 | 17840 | -0,08804098 | -0,28496 | TUMOR | Smokers |
| GSE47115 | IMMUNE SYSTEM PROCESS | THY1 | 16800 | -0,25831237 | -0,38048694 | TUMOR | Smokers |
| GSE32863 | IMMUNE SYSTEM PROCESS | THY1 | 33256 | -0,34405082 | -0,27733284 | TUMOR | Non-Smokers |
| TCGA | IMMUNE SYSTEM PROCESS | THY1 | 17840 | -0,08804098 | -0,25817692 | TUMOR | Smokers |
| GSE47115 | REGULATION OF IMMUNE SYSTEM PROCESS | THY1 | 16800 | -0,25831237 | -0,3703012 | TUMOR | Smokers |
| GSE32863 | DEFENSE RESPONSE | TLR3 | 28988 | -0,18938975 | -0,4235961 | TUMOR | Smokers |
| GSE50081 | IMMUNE SYSTEM PROCESS | TLR4 | 16263 | -0,08896815 | -0,36028886 | TUMOR | Smokers |
| GSE32863 | IMMUNE SYSTEM PROCESS | TLR4 | 31202 | -0,27158797 | -0,33004606 | TUMOR | Non-Smokers |
| GSE32863 | IMMUNE SYSTEM PROCESS | TLR4 | 33283 | -0,30721059 | -0,35312325 | TUMOR | Smokers |
| GSE50081 | DEFENSE RESPONSE | TLR6 | 20441 | -0,28708255 | -0,11021191 | TUMOR | Smokers |
| GSE32863 | DEFENSE RESPONSE | TLR6 | 31874 | -0,29257953 | -0,28656453 | TUMOR | Non-Smokers |
| GSE32863 | DEFENSE RESPONSE | TLR6 | 32847 | -0,29070115 | -0,3430415 | TUMOR | Smokers |
| GSE50081 | IMMUNE_RESPONSE | TLR7 | 17767 | -0,1235308 | -0,36748654 | TUMOR | Smokers |
| GSE32863 | IMMUNE_RESPONSE | TLR7 | 32015 | -0,26463479 | -0,39729124 | TUMOR | Smokers |
| TCGA | IMMUNE_RESPONSE | TLR7 | 17333 | -0,08005045 | -0,33109915 | TUMOR | Smokers |
| GSE50081 | IMMUNE SYSTEM PROCESS | TLR7 | 17767 | -0,1235308 | -0,32653615 | TUMOR | Smokers |
| GSE32863 | IMMUNE SYSTEM PROCESS | TLR7 | 32015 | -0,26463479 | -0,36617583 | TUMOR | Smokers |
| TCGA | IMMUNE SYSTEM PROCESS | TLR7 | 17333 | -0,08005045 | -0,29294905 | TUMOR | Smokers |
| GSE50081 | DEFENSE RESPONSE | TLR7 | 17767 | -0,1235308 | -0,3985131 | TUMOR | Smokers |
| GSE32863 | DEFENSE RESPONSE | TLR7 | 32015 | -0,26463479 | -0,36591676 | TUMOR | Smokers |
| TCGA | DEFENSE RESPONSE | TLR7 | 17333 | -0,08005045 | -0,33116022 | TUMOR | Smokers |
| GSE50081 | IMMUNE_RESPONSE | TLR8 | 20316 | -0,25986499 | -0,08616341 | TUMOR | Smokers |
| GSE32863 | IMMUNE_RESPONSE | TLR8 | 29326 | -0,19694574 | -0,4213957 | TUMOR | Smokers |
| GSE50081 | IMMUNE SYSTEM PROCESS | TLR8 | 20316 | -0,25986499 | -0,06825414 | TUMOR | Smokers |
| GSE32863 | IMMUNE SYSTEM PROCESS | TLR8 | 29326 | -0,19694574 | -0,39700934 | TUMOR | Smokers |
| GSE50081 | DEFENSE RESPONSE | TLR8 | 20316 | -0,25986499 | -0,11516731 | TUMOR | Smokers |
| GSE32863 | DEFENSE RESPONSE | TLR8 | 29326 | -0,19694574 | -0,41032496 | TUMOR | Smokers |
| GSE50081 | REGULATION OF IMMUNE SYSTEM PROCESS | TLR8 | 20316 | -0,25986499 | -0,1749953 | TUMOR | Smokers |
| GSE32863 | REGULATION OF IMMUNE SYSTEM PROCESS | TLR8 | 29326 | -0,19694574 | -0,36823368 | TUMOR | Smokers |
| GSE32863 | IMMUNE SYSTEM PROCESS | TM7SF4 | 36279 | -0,54262376 | -0,10513313 | TUMOR | Smokers |
| TCGA | IMMUNE SYSTEM PROCESS | TM7SF4 | 18513 | -0,33047861 | -0,14442724 | TUMOR | Non-Smokers |
| GSE32863 | IMMUNE_RESPONSE | TNFAIP1 | 28998 | -0,21114703 | -0,35630816 | TUMOR | Non-Smokers |
| GSE32863 | IMMUNE SYSTEM PROCESS | TNFAIP1 | 28998 | -0,21114703 | -0,37778687 | TUMOR | Non-Smokers |
| GSE32863 | DEFENSE RESPONSE | TNFAIP6 | 34877 | -0,42605448 | -0,14226227 | TUMOR | Non-Smokers |
| TCGA | DEFENSE RESPONSE | TNFAIP6 | 19107 | -0,11418658 | -0,17670904 | TUMOR | Smokers |
| GSE50081 | IMMUNE_RESPONSE | TNFRSF14 | 18835 | -0,15693168 | -0,28402215 | TUMOR | Smokers |
| GSE32863 | IMMUNE_RESPONSE | TNFRSF14 | 35326 | -0,41963997 | -0,23940927 | TUMOR | Smokers |
| TCGA | IMMUNE_RESPONSE | TNFRSF14 | 17056 | -0,07628789 | -0,35331097 | TUMOR | Smokers |
| TCGA | IMMUNE_RESPONSE | TNFRSF14 | 17107 | -0,25302172 | -0,23987152 | TUMOR | Non-Smokers |
| GSE50081 | IMMUNE SYSTEM PROCESS | TNFRSF14 | 18835 | -0,15693168 | -0,25355616 | TUMOR | Smokers |
| GSE32863 | IMMUNE SYSTEM PROCESS | TNFRSF14 | 35326 | -0,41963997 | -0,2022553 | TUMOR | Smokers |
| TCGA | IMMUNE SYSTEM PROCESS | TNFRSF14 | 17056 | -0,07628789 | -0,3127082 | TUMOR | Smokers |
| TCGA | IMMUNE SYSTEM PROCESS | TNFRSF14 | 17107 | -0,25302172 | -0,24745788 | TUMOR | Non-Smokers |
| GSE47115 | DEFENSE RESPONSE | TNFRSF1A | 16113 | -0,22654647 | -0,34354356 | TUMOR | Smokers |
| GSE32863 | DEFENSE RESPONSE | TNFRSF1A | 29226 | -0,2167073 | -0,34441504 | TUMOR | Non-Smokers |
| GSE32863 | DEFENSE RESPONSE | TNFRSF1A | 32227 | -0,27099478 | -0,3539339 | TUMOR | Smokers |
| GSE47115 | IMMUNE_RESPONSE | TNFRSF4 | 17119 | -0,27470413 | -0,40441284 | TUMOR | Smokers |
| TCGA | IMMUNE_RESPONSE | TNFRSF4 | 16626 | -0,23475137 | -0,27748632 | TUMOR | Non-Smokers |
| GSE47115 | IMMUNE SYSTEM PROCESS | TNFRSF4 | 17119 | -0,27470413 | -0,37565976 | TUMOR | Smokers |
| TCGA | IMMUNE SYSTEM PROCESS | TNFRSF4 | 16626 | -0,23475137 | -0,28781295 | TUMOR | Non-Smokers |
| GSE32863 | IMMUNE_RESPONSE | TNFSF13 | 36057 | -0,50103414 | -0,14927626 | TUMOR | Smokers |
| TCGA | IMMUNE_RESPONSE | TNFSF13 | 17104 | -0,25289762 | -0,24603473 | TUMOR | Non-Smokers |
| GSE32863 | IMMUNE SYSTEM PROCESS | TNFSF13 | 36057 | -0,50103414 | -0,12090048 | TUMOR | Smokers |
| TCGA | IMMUNE SYSTEM PROCESS | TNFSF13 | 17104 | -0,25289762 | -0,25172883 | TUMOR | Non-Smokers |
| GSE47115 | REGULATION OF IMMUNE SYSTEM PROCESS | TNFSF13 | 14595 | -0,16329749 | -0,4001449 | TUMOR | Smokers |
| GSE32863 | REGULATION OF IMMUNE SYSTEM PROCESS | TNFSF13 | 36057 | -0,50103414 | -0,1349558 | TUMOR | Smokers |
| TCGA | REGULATION OF IMMUNE SYSTEM PROCESS | TNFSF13 | 17104 | -0,25289762 | -0,35654354 | TUMOR | Non-Smokers |
| GSE32863 | DEFENSE RESPONSE | TNIP1 | 32652 | -0,2838878 | -0,34721887 | TUMOR | Smokers |
| GSE47115 | IMMUNE SYSTEM PROCESS | TPD52 | 19051 | -0,40011275 | -0,31145298 | TUMOR | Smokers |
| GSE32863 | IMMUNE SYSTEM PROCESS | TPD52 | 32927 | -0,29386878 | -0,35602763 | TUMOR | Smokers |
| GSE50081 | DEFENSE RESPONSE | TPSAB1 | 17698 | -0,12211871 | -0,4094821 | TUMOR | Smokers |
| TCGA | DEFENSE RESPONSE | TPSAB1 | 18829 | -0,10690114 | -0,20308506 | TUMOR | Smokers |
| GSE50081 | DEFENSE RESPONSE | TPST1 | 17295 | -0,11228035 | -0,4262191 | TUMOR | Smokers |
| TCGA | DEFENSE RESPONSE | TPST1 | 19843 | -0,14008476 | -0,10477845 | TUMOR | Smokers |
| GSE47115 | IMMUNE_RESPONSE | TRAF2 | 18051 | -0,3252193 | -0,35384238 | TUMOR | Smokers |
| TCGA | IMMUNE_RESPONSE | TRAF2 | 19266 | -0,4022426 | -0,10197084 | TUMOR | Non-Smokers |
| GSE47115 | IMMUNE SYSTEM PROCESS | TRAF2 | 18051 | -0,3252193 | -0,3449312 | TUMOR | Smokers |
| TCGA | IMMUNE SYSTEM PROCESS | TRAF2 | 19266 | -0,4022426 | -0,11678324 | TUMOR | Non-Smokers |
| GSE47115 | REGULATION OF IMMUNE SYSTEM PROCESS | TRAF2 | 18051 | -0,3252193 | -0,34184077 | TUMOR | Smokers |
| GSE10072 | REGULATION OF IMMUNE SYSTEM PROCESS | TRAF2 | 10556 | -0,29234776 | -0,40492734 | NORMAL | Non-Smokers |
| TCGA | REGULATION OF IMMUNE SYSTEM PROCESS | TRAF2 | 19266 | -0,4022426 | -0,07499987 | TUMOR | Non-Smokers |
| GSE50081 | IMMUNE_RESPONSE | TRAF6 | 19820 | -0,20783569 | -0,16060367 | TUMOR | Smokers |
| TCGA | IMMUNE_RESPONSE | TRAF6 | 17143 | -0,25455999 | -0,23535004 | TUMOR | Non-Smokers |
| GSE50081 | IMMUNE SYSTEM PROCESS | TRAF6 | 19820 | -0,20783569 | -0,12355348 | TUMOR | Smokers |
| TCGA | IMMUNE SYSTEM PROCESS | TRAF6 | 17143 | -0,25455999 | -0,24484822 | TUMOR | Non-Smokers |
| GSE50081 | REGULATION OF IMMUNE SYSTEM PROCESS | TRAF6 | 19820 | -0,20783569 | -0,22511198 | TUMOR | Smokers |
| TCGA | REGULATION OF IMMUNE SYSTEM PROCESS | TRAF6 | 17143 | -0,25455999 | -0,3374534 | TUMOR | Non-Smokers |
| GSE47115 | IMMUNE_RESPONSE | TRAT1 | 18928 | -0,38972649 | -0,32753146 | TUMOR | Smokers |
| GSE32863 | IMMUNE_RESPONSE | TRAT1 | 32300 | -0,27324918 | -0,3898092 | TUMOR | Smokers |
| TCGA | IMMUNE_RESPONSE | TRAT1 | 19036 | -0,11230837 | -0,16481665 | TUMOR | Smokers |
| GSE47115 | IMMUNE SYSTEM PROCESS | TRAT1 | 18928 | -0,38972649 | -0,31557134 | TUMOR | Smokers |
| GSE32863 | IMMUNE SYSTEM PROCESS | TRAT1 | 32300 | -0,27324918 | -0,36264315 | TUMOR | Smokers |
| TCGA | IMMUNE SYSTEM PROCESS | TRAT1 | 19036 | -0,11230837 | -0,15085846 | TUMOR | Smokers |
| GSE47115 | CELLULAR DEFENSE RESPONSE | TRAT1 | 18928 | -0,38972649 | -0,25387695 | TUMOR | Smokers |
| GSE32863 | CELLULAR DEFENSE RESPONSE | TRAT1 | 32300 | -0,27324918 | -0,47796586 | TUMOR | Smokers |
| TCGA | CELLULAR DEFENSE RESPONSE | TRAT1 | 19036 | -0,11230837 | -0,09391723 | TUMOR | Smokers |
| GSE47115 | DEFENSE RESPONSE | TRAT1 | 18928 | -0,38972649 | -0,21322699 | TUMOR | Smokers |
| GSE32863 | DEFENSE RESPONSE | TRAT1 | 32300 | -0,27324918 | -0,3514046 | TUMOR | Smokers |
| TCGA | DEFENSE RESPONSE | TRAT1 | 19036 | -0,11230837 | -0,18012469 | TUMOR | Smokers |
| GSE47115 | REGULATION OF IMMUNE SYSTEM PROCESS | TRAT1 | 18928 | -0,38972649 | -0,2995969 | TUMOR | Smokers |
| GSE10072 | REGULATION OF IMMUNE SYSTEM PROCESS | TRAT1 | 12781 | -0,55261058 | -0,15063837 | NORMAL | Non-Smokers |
| GSE32863 | REGULATION OF IMMUNE SYSTEM PROCESS | TRAT1 | 32300 | -0,27324918 | -0,33173957 | TUMOR | Smokers |
| TCGA | IMMUNE_RESPONSE | TREM1 | 18151 | -0,09329516 | -0,27669442 | TUMOR | Smokers |
| TCGA | IMMUNE_RESPONSE | TREM1 | 19154 | -0,38867149 | -0,12573512 | TUMOR | Non-Smokers |
| TCGA | IMMUNE SYSTEM PROCESS | TREM1 | 18151 | -0,09329516 | -0,24347189 | TUMOR | Smokers |
| TCGA | IMMUNE SYSTEM PROCESS | TREM1 | 19154 | -0,38867149 | -0,14545247 | TUMOR | Non-Smokers |
| GSE47115 | IMMUNE_RESPONSE | TREM2 | 20367 | -0,60802132 | -0,12681003 | TUMOR | Smokers |
| GSE32863 | IMMUNE_RESPONSE | TREM2 | 35382 | -0,42444447 | -0,20914896 | TUMOR | Smokers |
| TCGA | IMMUNE_RESPONSE | TREM2 | 17027 | -0,24991438 | -0,25471953 | TUMOR | Non-Smokers |
| GSE47115 | IMMUNE SYSTEM PROCESS | TREM2 | 20367 | -0,60802132 | -0,13030209 | TUMOR | Smokers |
| GSE32863 | IMMUNE SYSTEM PROCESS | TREM2 | 35382 | -0,42444447 | -0,18018353 | TUMOR | Smokers |
| TCGA | IMMUNE SYSTEM PROCESS | TREM2 | 17027 | -0,24991438 | -0,2610363 | TUMOR | Non-Smokers |
| GSE50081 | IMMUNE_RESPONSE | TRIM22 | 17328 | -0,112845 | -0,41021845 | TUMOR | Smokers |
| TCGA | IMMUNE_RESPONSE | TRIM22 | 17441 | -0,08164416 | -0,320526 | TUMOR | Smokers |
| GSE50081 | IMMUNE SYSTEM PROCESS | TRIM22 | 17328 | -0,112845 | -0,35978612 | TUMOR | Smokers |
| TCGA | IMMUNE SYSTEM PROCESS | TRIM22 | 17441 | -0,08164416 | -0,28672507 | TUMOR | Smokers |
| GSE47115 | CELLULAR DEFENSE RESPONSE | TYROBP | 17871 | -0,31500283 | -0,23148248 | TUMOR | Smokers |
| GSE32863 | CELLULAR DEFENSE RESPONSE | TYROBP | 35789 | -0,46456781 | -0,23580883 | TUMOR | Smokers |
| GSE47115 | DEFENSE RESPONSE | TYROBP | 17871 | -0,31500283 | -0,25377974 | TUMOR | Smokers |
| GSE32863 | DEFENSE RESPONSE | TYROBP | 35789 | -0,46456781 | -0,16011807 | TUMOR | Smokers |
| TCGA | IMMUNE_RESPONSE | UBE2N | 16457 | -0,22841558 | -0,29796416 | TUMOR | Non-Smokers |
| TCGA | IMMUNE SYSTEM PROCESS | UBE2N | 16457 | -0,22841558 | -0,31167543 | TUMOR | Non-Smokers |
| TCGA | REGULATION OF IMMUNE SYSTEM PROCESS | UBE2N | 16457 | -0,22841558 | -0,36541757 | TUMOR | Non-Smokers |
| GSE47115 | CELLULAR DEFENSE RESPONSE | UMOD | 15029 | -0,18175104 | -0,39496732 | TUMOR | Smokers |
| GSE32863 | CELLULAR DEFENSE RESPONSE | UMOD | 30729 | -0,25769725 | -0,31217924 | TUMOR | Non-Smokers |
| GSE47115 | DEFENSE RESPONSE | UMOD | 15029 | -0,18175104 | -0,3481241 | TUMOR | Smokers |
| GSE32863 | DEFENSE RESPONSE | UMOD | 30729 | -0,25769725 | -0,30274922 | TUMOR | Non-Smokers |
| TCGA | CELLULAR DEFENSE RESPONSE | VEZF1 | 14161 | -0,04295281 | -0,40865028 | TUMOR | Smokers |
| GSE50081 | IMMUNE_RESPONSE | VIPR1 | 18954 | -0,16156028 | -0,24325621 | TUMOR | Smokers |
| TCGA | IMMUNE_RESPONSE | VIPR1 | 15870 | -0,06114808 | -0,40192118 | TUMOR | Smokers |
| TCGA | IMMUNE_RESPONSE | VIPR1 | 15519 | -0,19616143 | -0,35689926 | TUMOR | Non-Smokers |
| GSE50081 | IMMUNE SYSTEM PROCESS | VIPR1 | 18954 | -0,16156028 | -0,21010187 | TUMOR | Smokers |
| TCGA | IMMUNE SYSTEM PROCESS | VIPR1 | 15870 | -0,06114808 | -0,35541755 | TUMOR | Smokers |
| TCGA | IMMUNE SYSTEM PROCESS | VIPR1 | 15519 | -0,19616143 | -0,35702342 | TUMOR | Non-Smokers |
| GSE50081 | IMMUNE_RESPONSE | VTN | 17410 | -0,11512362 | -0,38538975 | TUMOR | Smokers |
| GSE32863 | IMMUNE_RESPONSE | VTN | 28291 | -0,19432843 | -0,36361876 | TUMOR | Non-Smokers |
| GSE32863 | IMMUNE_RESPONSE | VTN | 32298 | -0,27315003 | -0,39489925 | TUMOR | Smokers |
| TCGA | IMMUNE_RESPONSE | VTN | 16057 | -0,21331003 | -0,332937 | TUMOR | Non-Smokers |
| GSE50081 | IMMUNE SYSTEM PROCESS | VTN | 17410 | -0,11512362 | -0,34249926 | TUMOR | Smokers |
| GSE32863 | IMMUNE SYSTEM PROCESS | VTN | 28291 | -0,19432843 | -0,38243464 | TUMOR | Non-Smokers |
| GSE32863 | IMMUNE SYSTEM PROCESS | VTN | 32298 | -0,27315003 | -0,36641124 | TUMOR | Smokers |
| TCGA | IMMUNE SYSTEM PROCESS | VTN | 16057 | -0,21331003 | -0,3414903 | TUMOR | Non-Smokers |
| GSE32863 | IMMUNE_RESPONSE | WAS | 35753 | -0,50383455 | -0,1681798 | TUMOR | Non-Smokers |
| GSE32863 | IMMUNE_RESPONSE | WAS | 34113 | -0,3431159 | -0,34329188 | TUMOR | Smokers |
| TCGA | IMMUNE_RESPONSE | WAS | 17907 | -0,08918812 | -0,2824621 | TUMOR | Smokers |
| TCGA | IMMUNE_RESPONSE | WAS | 16668 | -0,23642665 | -0,27371925 | TUMOR | Non-Smokers |
| GSE32863 | IMMUNE SYSTEM PROCESS | WAS | 35753 | -0,50383455 | -0,15878801 | TUMOR | Non-Smokers |
| GSE32863 | IMMUNE SYSTEM PROCESS | WAS | 34113 | -0,3431159 | -0,30273333 | TUMOR | Smokers |
| TCGA | IMMUNE SYSTEM PROCESS | WAS | 17907 | -0,08918812 | -0,25727654 | TUMOR | Smokers |
| TCGA | IMMUNE SYSTEM PROCESS | WAS | 16668 | -0,23642665 | -0,2858236 | TUMOR | Non-Smokers |
| GSE47115 | DEFENSE RESPONSE | WAS | 15489 | -0,19999504 | -0,3502565 | TUMOR | Smokers |
| GSE32863 | DEFENSE RESPONSE | WAS | 35753 | -0,50383455 | -0,07013823 | TUMOR | Non-Smokers |
| GSE32863 | DEFENSE RESPONSE | WAS | 34113 | -0,3431159 | -0,2985907 | TUMOR | Smokers |
| TCGA | DEFENSE RESPONSE | WAS | 17907 | -0,08918812 | -0,27647388 | TUMOR | Smokers |
| GSE47115 | DEFENSE RESPONSE | WFDC12 | 19538 | -0,44653428 | -0,191037 | TUMOR | Smokers |
| GSE32863 | DEFENSE RESPONSE | WFDC12 | 31848 | -0,25996369 | -0,36572435 | TUMOR | Smokers |
| GSE50081 | IMMUNE_RESPONSE | XBP1 | 18839 | -0,15705106 | -0,2710356 | TUMOR | Smokers |
| TCGA | IMMUNE_RESPONSE | XBP1 | 19876 | -2,86075139 | -1,78178E-08 | TUMOR | Non-Smokers |
| GSE50081 | IMMUNE SYSTEM PROCESS | XBP1 | 18839 | -0,15705106 | -0,24401826 | TUMOR | Smokers |
| TCGA | IMMUNE SYSTEM PROCESS | XBP1 | 19876 | -2,86075139 | 5,36E-07 | TUMOR | Non-Smokers |
| GSE47115 | DEFENSE RESPONSE | XCR1 | 16496 | -0,24349631 | -0,33319739 | TUMOR | Smokers |
| TCGA | DEFENSE RESPONSE | XCR1 | 19789 | -0,13706577 | -0,12741007 | TUMOR | Smokers |
| GSE50081 | IMMUNE_RESPONSE | ZAP70 | 19261 | -0,17581129 | -0,21533468 | TUMOR | Smokers |
| GSE47115 | IMMUNE_RESPONSE | ZAP70 | 19844 | -0,48565304 | -0,24660754 | TUMOR | Smokers |
| GSE32863 | IMMUNE_RESPONSE | ZAP70 | 27495 | -0,17561308 | -0,37986842 | TUMOR | Non-Smokers |
| GSE32863 | IMMUNE_RESPONSE | ZAP70 | 36611 | -0,70560974 | -0,024876341 | TUMOR | Smokers |
| GSE50081 | IMMUNE SYSTEM PROCESS | ZAP70 | 19261 | -0,17581129 | -0,16215937 | TUMOR | Smokers |
| GSE47115 | IMMUNE SYSTEM PROCESS | ZAP70 | 19844 | -0,48565304 | -0,23058623 | TUMOR | Smokers |
| GSE32863 | IMMUNE SYSTEM PROCESS | ZAP70 | 36611 | -0,70560974 | -0,01781359 | TUMOR | Smokers |
| GSE47115 | REGULATION OF IMMUNE SYSTEM PROCESS | ZAP70 | 19844 | -0,48565304 | -0,20548457 | TUMOR | Smokers |
| GSE32863 | REGULATION OF IMMUNE SYSTEM PROCESS | ZAP70 | 36611 | -0,70560974 | 0,00248386 | TUMOR | Smokers |
| TCGA | IMMUNE_RESPONSE | ZEB1 | 15937 | -0,06194923 | -0,39714035 | TUMOR | Smokers |
| TCGA | IMMUNE SYSTEM PROCESS | ZEB1 | 15937 | -0,06194923 | -0,35284573 | TUMOR | Smokers |
| GSE47115 | CELLULAR DEFENSE RESPONSE | ZNF148 | 19104 | -0,40482444 | -0,23253334 | TUMOR | Smokers |
| GSE32863 | CELLULAR DEFENSE RESPONSE | ZNF148 | 36109 | -0,55623579 | -0,031045208 | TUMOR | Non-Smokers |
| GSE47115 | DEFENSE RESPONSE | ZNF148 | 19104 | -0,40482444 | -0,21469995 | TUMOR | Smokers |
| GSE32863 | DEFENSE RESPONSE | ZNF148 | 36109 | -0,55623579 | -0,025104878 | TUMOR | Non-Smokers |
| TCGA | IMMUNE SYSTEM PROCESS | ZNF675 | 18188 | -0,09400357 | -0,24084839 | TUMOR | Smokers |
| TCGA | IMMUNE SYSTEM PROCESS | ZNF675 | 15826 | -0,20572619 | -0,34441552 | TUMOR | Non-Smokers |
